# Imine as a linchpin approach for *meta*-C–H functionalization

**DOI:** 10.1038/s41467-021-21633-2

**Published:** 2021-03-02

**Authors:** Sukdev Bag, Sadhan Jana, Sukumar Pradhan, Suman Bhowmick, Nupur Goswami, Soumya Kumar Sinha, Debabrata Maiti

**Affiliations:** Department of Chemistry, Indian Institute of Technology Bombay, Powai, Mumbai, India

**Keywords:** Catalytic mechanisms, Homogeneous catalysis, Synthetic chemistry methodology

## Abstract

Despite the widespread applications of C–H functionalization, controlling site selectivity remains a significant challenge. Covalently attached directing groups (DGs) served as ancillary ligands to ensure *ortho*-, *meta*- and *para*-C–H functionalization over the last two decades. These covalently linked DGs necessitate two extra steps for a single C–H functionalization: introduction of DG prior to C–H activation and removal of DG post-functionalization. Here we report a temporary directing group (TDG) for *meta*-C–H functionalization via reversible imine formation. By overruling facile *ortho*-C–H bond activation by imine-*N* atom, a suitably designed pyrimidine-based TDG successfully delivered selective *meta*-C–C bond formation. Application of this temporary directing group strategy for streamlining the synthesis of complex organic molecules without any necessary pre-functionalization at the *meta* position has been explored.

## Abstract (web summary)

Site-selective C–H functionalization still faces some challenges, such as the introduction and removal of an appropriate directing group. Here, the authors introduce a temporary directing group for selective *meta*-C–H functionalization of 2-arylbenzaldehydes via reversible imine formation.

## Introduction

**Fig. 1:**
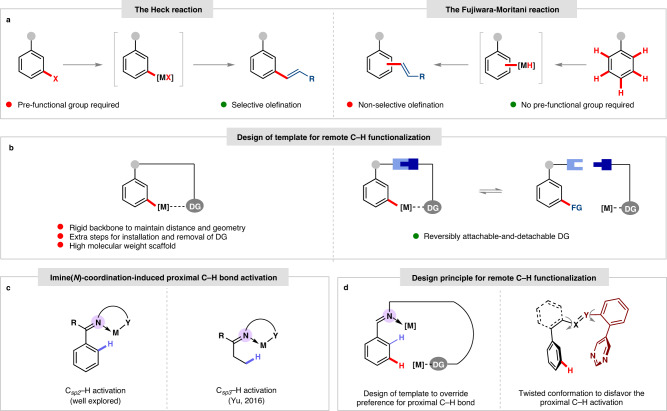
Temporary directing group (TDG) assisted remote C–H functionalization. **a** The Heck reaction vs The Fujiwara-Moritani reaction. **b** Covalent template directed remote C–H functionalization vs TDG; X=Y, imine; M, Metal; DG, Directing Group; FG, Functional Group. **c** TDG-assisted *ortho*-C–H and C(*sp*^*3*^)–H functionalization. **d** Challenges associated with the TDG promoted *meta*-C–H functionalization.

Evident from several paradigms of chemical transformations observed in nature, selective and efficient reactions relied either on inherent electronic nature of a molecule or through direct interaction with a metal center^1–4^. A classic example of C–C bond formation reaction is the Heck olefination that can be realized with aryl halides and olefins via organopalladium intermediate (Fig. 1a)^5^. Alternatively, arene has been used as the coupling partner with activated olefins via inert C–H bond activated organopalladium intermediate, namely the Fujiwara-Moritani reaction (Fig. 1a)^6,7^. Discovery of the proximity-driven C–H functionalization reactions by omitting the requirement of prefunctionalization has drawn a significant attention in the recent years. Innate and directing group (DG) assisted strategies have been developed for selective C–H functionalization and greatly applied in the late-stage diversification of natural products and pharmaceuticals^8–10^. Template-directed approaches successfully promoted *meta*- and *para*-C–H bond functionalization of arene substrates^11–15^. To achieve exclusive *meta*-C–H bond functionalization, various well-designed template-based assembly has been discovered by adopting distance and geometry correlation (Fig. 1b). However, additional steps for installation and removal of covalently attached directing group indicate substantial limitation. In addition, a high molecular weight scaffold comprising of significant number of chemically equivalent C–H bonds is prone to deliver less selective functional group incorporation. Alternative approach by overruling the extra-steps associated with covalently linked template-based assembly is highly desirable^16–27^. We envisioned the development of a temporary DG, which can be designed to bind substrate reversibly and can also accommodate a metal center via strong coordination (Fig. 1b).

An interesting substrate-cum-template-design can evolve around reversible imine formation with aldehyde or amine substrates, which are ubiquitous and inexpensive reagent. While *ortho*-C–H functionalization with the aid of transient directing group (TDG) approach has been well appreciated in the community (Fig. 1c), the remote C(*sp*^*2*^)–H functionalization via in situ imine formation remains yet an undiscovered domain^26,28–36^. The reason behind this unsolved problem is the selectivity issue arising from imine-*N* atom, which is prone to deliver the undesired *ortho*-C–H functionalization^37^ via stable 5-6-membered *endo*- or *exo*-carbometallation (Fig. 1c)^38^. Undermining the thermodynamically stable intermediate and favoring large metallacycle transition state (TS), an important factor is the formation of a suitable template-assembly, which will disfavor the *ortho*-carbometallation with imine-*N* atom (Fig. 1d).

Here, we demonstrate that the biphenyl pyrimidyl-based amine and aldehyde act as TDGs for *meta*-C–H olefination of 2-arylbenzaldehyde and 2-arylaniline derivatives, respectively, through reversible imine formation.

## Results

### Template optimization

**Fig. 2:**
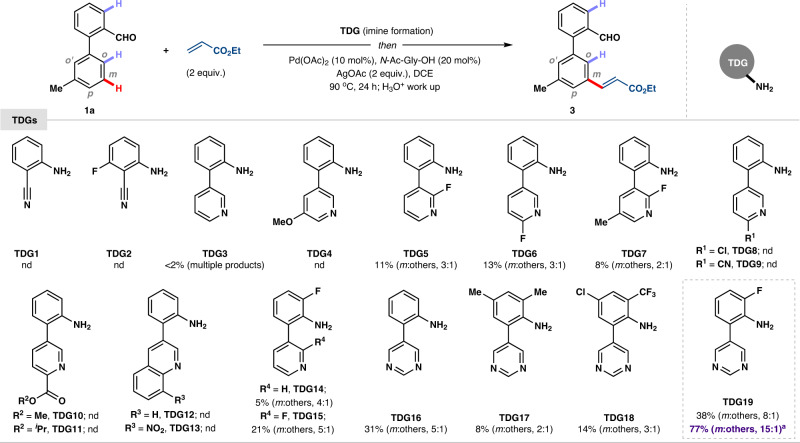
TDG evaluation for *meta*-C–H functionalization of biphenyl aldehyde. Imine formation: 1a (0.2 mmol) and TDG (0.2 mmol) in ^*i*^PrOH (2 mL) at 80 °C for 2 h; Olefination: Pd(OAc)_2_ (10 mol%), *N*-Ac-Gly-OH (20 mol%), AgOAc (2 equiv.) and ethyl acrylate (2 equiv.) in 1,2-dichloroethane (DCE, 2 mL) at 90 °C for 24 h. ^a^Imine formation: 1a (0.2 mmol) and TDG19 (0.19 mmol) in ^*i*^PrOH (2 mL) at 80 °C for 2 h; Olefination: Pd(OAc)_2_ (10 mol%), *N*-Form-Gly-OH (20 mol%), Ag_2_CO_3_ (25 mol%), Cu(OAc)_2_ (3.5 equiv.) and ethyl acrylate (3 equiv.) in DCE (2 mL) at 100 °C for 24 h. Ratios of *meta*:others were shown in parenthesis; nd, not detected.

Phenyl ring on both side of imine linchpin was anticipated to be the productive combination in terms of rigid geometry and sufficient stability in reaction medium to deliver the desired remote C–H functionalization. In this regard, adopting distance and geometry correlation, biphenyl aldehyde was investigated as a substrate in substrate-cum-template-design cognition. We have investigated biphenyl aldehyde with 2-amino benzonitrile as the TDG (TDG1 and TDG2) using palladium (II) acetate as catalyst and mono-protected amino acid (MPAA) *N*-Ac-Gly-OH as ligand (Fig. 2) (see the Supplementary Information). These experiments resulted in either no product formation or trace amount of product formation with very poor selectivity perhaps due to the deactivation of palladium catalyst or formation of bidentate chelate with imine-*N* and *π*-bond of nitrile in ‘side-on’ fashion. In addition, competitive coordination from imine and nitrile can be detrimental as both have equivalent coordinating ability leading to the multiple product formation. To overcome these issues, we decided to synthesize heterocycle-based 2-(pyridin-3-yl)aniline temporary directing groups. A macrocyclic TS with imine adduct having two biphenyls at two end of imine linchpin and palladium may promote a twisted conformation resulting in an unfavorable coordination mode with imine-*N* and can undermine the formation of *ortho*-selective products. Pyridine-based biphenyl templates (TDG3 and TDG4) have either provided trace amount of olefination product or was found completely inactive (Fig. 2). After further modification of TDGs, it was found that electron-deficient fluoro-substituted pyridine-based templates (TDG5-TDG7) could be significantly better than electron-rich pyridine-based ligand and delivered the desired olefination product at distal *meta*-position. Other variation of electron-deficient substitutions on pyridine ring such as chloro (TDG8) and nitrile (TDG9) did not provide any olefinated product. We anticipated that bidentate coordination with palladium may serve as an active catalyst pocket and free amine on another phenyl ring of biphenyl template (TDG10 and TDG11) can act as a reversible binding site to deliver the desired product by confining the catalyst. However, none of the bidentate ligands provided expected olefination product probably due to the formation of unreactive palladium complex. Quinoline-based templates (TDG12 and TDG13) were unsuccessful to deliver the desired product. Further fine-tuning of TDG showed that *ortho*-substitution with fluorine atom with respect to amine (TDG14 and TDG15) can deliver the desired product in better yield and selectivity compared to the unsubstituted TDGs (TDG3 and TDG5). Subsequently, we thought to focus on more electron-deficient monodentate ligand. Encouragingly, pyrimidine-based ligand (TDG16) provided promising yield in 5:1 *meta*-selectivity^39–43^. In contrast, *ortho*-methyl (TDG17) and trifluoromethyl substitution (TDG18) with respect to amine on pyrimidine-based templates resulted in poor yield (8% and 14%) and selectivity (*m*:others; 2:1 & 3:1). Such an observation suggests proper conformation of macrocyclic TS is the key factor for *meta*-C–H functionalization that might be disturbed by steric hindrance arising from *ortho* substitutions. Finally, pyrimidine-based ligand with *ortho*- fluorine substitution (TDG19) provided promising result in terms of yield (38%) and selectivity (*m*:others; 8:1). An *ortho*-fluoro-substitution (TDG19) resulted in efficient olefination. Fluorine atom helps to prevent undesired *ortho*-C–H functionalization as well as it may assist a proper conformation of macrocyclic TS^44^. The presence of an extra electron-withdrawing nitrogen atom in pyrimidine (compared to pyridine) served as a *σ*-donor and strong *π*-acceptor ligand. These combined effects facilitate a strong binding with palladium, which helps in C–H activation followed by 1,2-migratory insertion in alkene to deliver the desired olefination product.

### Reaction optimization

With the optimal directing group TDG19, we have tested several parameters to achieve synthetically useful yield and *meta*-selectivity. The use of 1,1,1,3,3,3-hexafluoroisopropanol (HFIP) as a solvent led to decomposition of imine and subsequently 1,2-dichloroethane (DCE) provided a superior result. Different proportionate mixture of DCE and HFIP failed to deliver the desired product. We tested several MPAA ancillary ligands and *N*-Form-Gly-OH is found to be most suitable (see the Supplementary Information). Catalytic amount of silver(I) carbonate in conjunction with copper(II) acetate as an oxidant delivered 77% yield and 15:1 *meta*- selectivity. To utilize the TDG either as catalytic amount or for single step olefination, we intended to examine the solvent medium as it plays a major role in two steps; imine formation and C–H activation. We have then evaluated the olefination reaction with the two best solvents, DCE and HFIP, by varying TDG19 from catalytic to stoichiometric amount keeping other optimized reagents intact (see Supplementary Table 12 in the Supplementary Information). It was found that DCE provided lower yield with the gradual increase in the amount of TDG19 whereas HFIP delivered comparably higher yields. The combination of two solvents also did not provide a superior result. Due to the acidic nature of HFIP, it forms a reversible imine intermediate, which might have less sustainability to complete a catalytic cycle and it eventually delivers olefination product with lower selectivity. The observed lower selectivity for *meta*-olefination in case of catalytic amount of TDG19 is due to the formation of competitive *ortho*-olefination products with aldehedye substrate **1a**. Although isopropanol is efficient for imine formation, olefination reaction does not work in this solvent.

### Scope of *meta*-C–H olefination

**Fig. 3:**
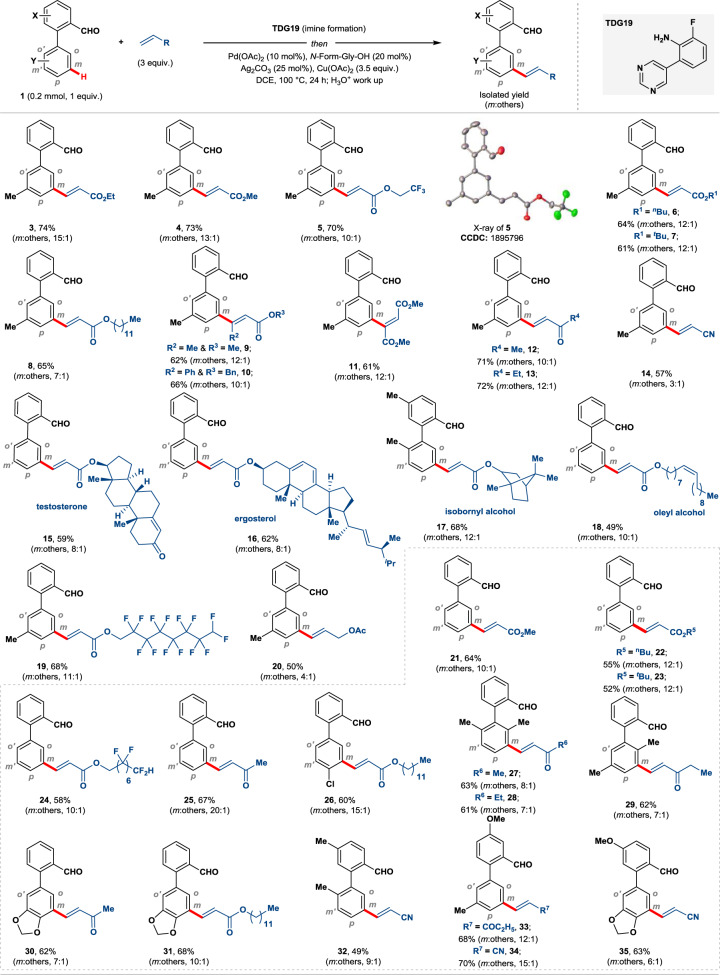
Scope with a variety of alkenes. Combination of various alkenes and arenes are shown in dotted box. The isolated yields are reported and ratios of *meta*:others are shown in parenthesis.

Using the optimized conditions and TDG19 as a temporary directing group, we tested C–C bond formation scope with different alkene coupling partners (Fig. 3). These included both short chain and long-chain olefins (**3**–**8**), *β*-substituted (**9** and **10**) and *α*, *β*-dicarboxylates (**11**), which consistently gave C–C bond formation products. The regioselectivity of **5** was confirmed by X-ray structures. Other important class of activated olefins such as vinyl ketone (**12** and **13**) and acrylonitrile (**14**) were utilized successfully in this reaction. Natural products and bioactive molecules appended acrylates that include testosterone (**15**), ergosterol (**16**), isobornyl alcohol (**17**) and oleyl alcohol (**18**) displayed useful coupling efficiencies under this protocol. Acrylate containing perfluoroalkene chain (**19**) were also coupled with 2-phenylbenzaldehyde exclusively at *meta* position. Notably, unactivated alkene such as allyl acetate (**20**) provided alkenylation product over facile formation of allylation product. The compatibility of different alkene partners with differently substituted arenes were tested successfully under present protocol (**21**–**35**).

**Fig. 4:**
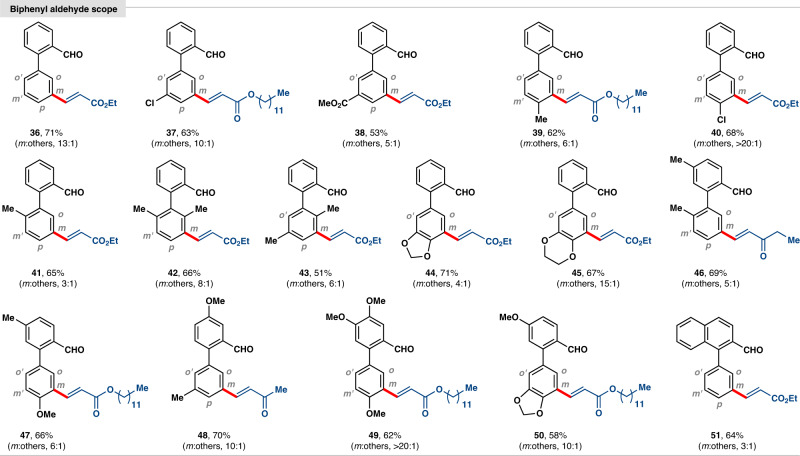
Biphenyl aldehyde substrates for selective *meta*-C–H functionalization. The yields are isolated and ratios of *meta*:others are shown in parenthesis.

After establishing scope with a variety of alkenes, we aimed to verify the reactivity with differently substituted 2-phenylbenzaldehydes (Fig. 4). Electron-neutral, electron-donating and electron-withdrawing substituents-containing substrates were all tolerated and provided the *meta*-selective products. Although biphenyl aldehyde with carboxylate unit (**38**) is prone to deliver multiple products, the current protocol provided *meta*-selective C–C bond formation. Sterically encumbered *para*-substituted methyl and chloro-containing arenes (**39** and **40**) delivered the desired product with excellent *meta*-selectivity. Interestingly, substrates bearing sterically demanding *di*-methyl substitutions (**42** and **43**) also provided *meta*-selective olefination by overriding the steric bias. Notably, electron-rich heterocycle-containing arenes (**44** and **45**) delivered mono-olefination in synthetically useful yield and *meta*- selectivity. Versatile biphenyl derivatives (**46**–**50**) were also found to be competent coupling partners with different alkenes. 1-Phenyl 2-naphthaldehyde (**51**) provided site selective olefination undermining any other possibilities. For some cases, a small amount of acid (from aldehyde) product was detected in the reaction mixture as a side product.

**Fig. 5:**
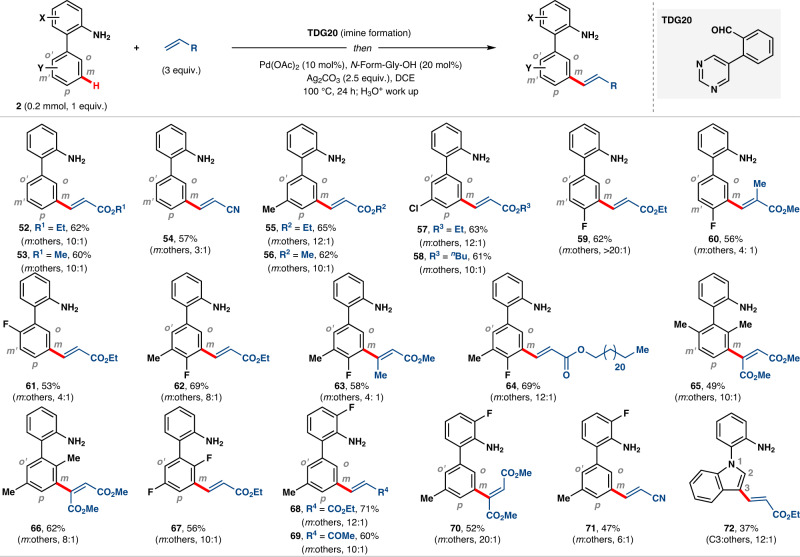
Biphenyl amine substrates for selective *meta*-C–H functionalization. The isolated yields are reported and ratios of *meta*:others are shown in parenthesis.

We next turned our attention to 2-phenylaniline substrates utilizing aldehyde group on pyrimidine-based TDG (TDG20) (Fig. 5). While electron-donating amine group is *ortho*- and *para*- directing, overruling the electronic bias and reaching to the *meta*-C–H bond of other phenyl ring is a significant challenge. Although, 2-phenylaniline delivered the *meta*-olefination product under the present optimal reaction conditions, we experienced several difficulties such as formation of less selective olefinated product and mostly, generation of C–N coupled cyclized product.

After systematic investigations, it was found that copper (II) acetate was responsible for facile formation of C–N coupled side product. Accordingly, exclusion of copper (II) acetate and use of silver (I) carbonate provided excellent *meta*-selective olefination product (**52**; *m*:others, 10:1) (see the Supplementary Information). With this modified reaction conditions, electron-rich (**55** and **56**) and electron-deficient biphenylamine (**57**–**64**) were evaluated and the desired olefination products were obtained with significant *meta*-selectivity. Interestingly, template geometry overrode the steric experience from di-substituted arene (**65**–**67**) and delivered preparatively useful yield of *meta*-selective olefination product. Both arene substituted biphenylamine was coupled with different alkene partners such as ethyl acrylate (**68**), methyl vinyl ketone (**69**), dimethyl fumarate (**70**) and acrylonitrile (**71**). Indole was functionalized selectively at C3 position with the aid of *N*-substituted 2-arylamine as a linchpin arene (**72**).

### Synthetic diversification

**Fig. 6:**
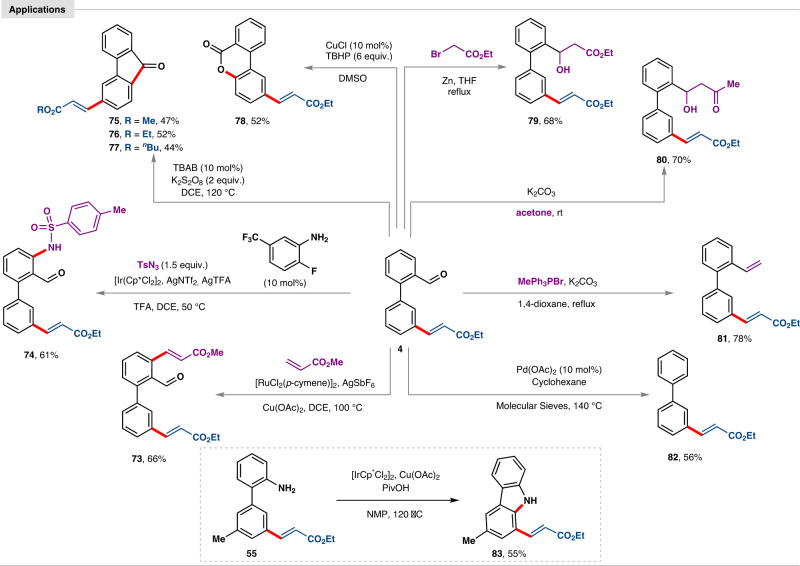
Post synthetic applications. The diverse utilities of aldehyde group are presented. Synthesis of carbazole from obtained *meta*-olefinated 2-phenylaniline is shown in the dotted box.

In addition to the *meta*-C–H functionalization as the stepping stone for mono selective C–C bond formation, *ortho*-transformation can also be promoted efficiently (**73** and **74**) (Fig. 6). The *meta*-olefinated products of 2-phenyl benzaldehyde were expediently elaborated into corresponding fluorenone (**75**–**77**) and benzo[*c*]chromenone (**78**) via oxidative cyclization. Furthermore, aldehyde group was converted to *β*-secondary alcohols of ester (**79**) and carbonyl (**80**). From substituted benzaldehyde (**36**) corresponding styrene derivative (**81**) has been synthesized using a Wittig reagent. Decarbonylation can be promoted to obtain unsubstituted 3-phenyl cinnamate (**82**). *Meta*-olefinated 2-phenylaniline was successfully converted into cyclized carbazole moiety (**83**) present in numerous natural products.

**Fig. 7:**
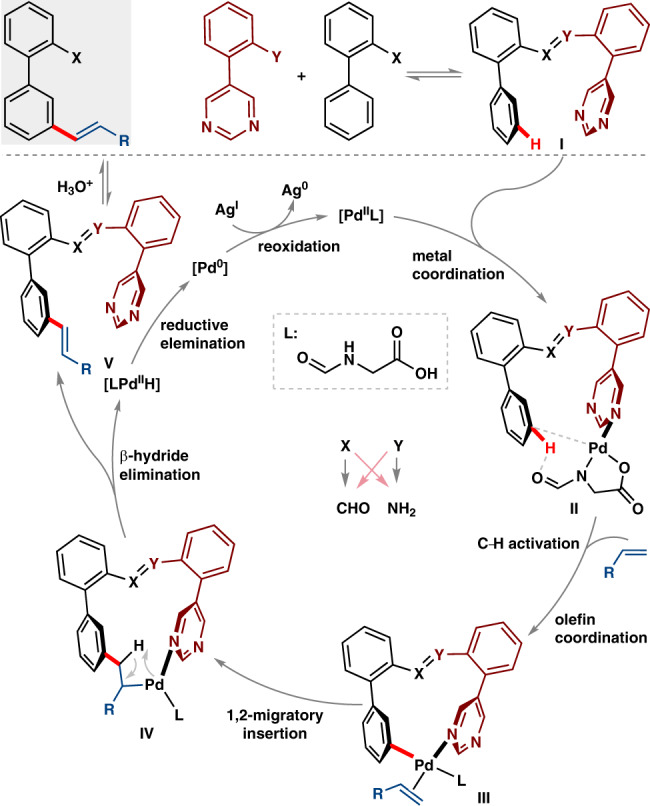
Plausible catalytic cycle for *meta*-C–H olefination. Reversible imine formation then involvement of palladium and olefin in catalytic cycle has been depicted.

We have outlined a plausible catalytic cycle in Fig. 7. The *N*-atom of pyrimidine (**I**) coordinates with palladium bound MPAA ligand (L) (i.e., [Pd^II^L]) to form intermediate **II**. The two hinged biphenyls in imine linchpin are assumed to be in a twisted conformation which brings palladium to the close proximity of target *meta*-C–H bond. With the assistance of MPAA ligand (i.e., *N*-Form-Gly-OH), palladium activates the *meta*-C–H bond via concerted metalation-deprotonation (CMD) pathway to form carbopalladation intermediate. Olefin coordination with palladium (intermediate **III**) and subsequent 1,2-migratory insertion of palladium lead to the macrocyclic intermediate **IV**. *β*-Hydride elimination from intermediate **IV** delivers the olefination product. Ratio of regioisomers formation varies with olefin coupling partners (Michael acceptor) might be due to the weak coordination of olefin with palladium in prior or during the C–H activation step. The steric and electronic properties of substitution in olefin partially determines the regioselectivity.

In summary, we have developed a temporary directing group approach to obtain selective *meta*-C–H functionalization on synthetically important aldehyde and amine. Template design facilitated the formation of macrocyclic transition state to enable proximity-induced reactivity and selectivity. An array of substrate variation irrespective of electronic and steric demands on both aldehyde and amine were explored. The capability of this transformation to install complex molecules at the remote position and post-synthetic manipulation of prevalent aromatic aldehyde and amine groups render this strategy amenable towards practical applications.

## Methods

### *Meta-*C–H olefination of 2-phenylbenzaldehyde derivatives

An oven-dried screw capped reaction tube with a magnetic stir-bar was charged with 2-phenylbenzaldehyde (0.2 mmol) (viscous biphenyl aldehyde was weighed first), and TDG19 (0.19 mmol, 36 mg) under air, followed by isopropyl alcohol (2 mL). The reaction mixture was stirred at 80 °C for 2 h. The mixture was allowed to cool down and almost full conversion was observed in thin layer chromatography. Next, solvent was concentrated in vacuo and washed with pentane. The dry crude solid residue was subjected to Pd(OAc)_2_ (10 mol%, 0.02 mmol, 4.5 mg), *N*-formyl glycine (*N*-Form-Gly-OH; 20 mol%, 8.3 mg), Ag_2_CO_3_ (25 mol%, 0.05 mmol, 14 mg) and Cu(OAc)_2_ (3.5 equiv., 0.7 mmol, 127 mg) in the same reaction tube. Solvent 1,2-dichloroethane (DCE, 2 mL) was added in the reaction tube followed by addition of liquid alkene (3 equiv., 0.6 mmol) by micropipette under air (solid alkenes were weighed before adding solvent). The reaction tube was screwed by a cap fitted with a rubber septum and was vigorously stirred in a preheated oil bath at 100 °C. The reaction mixture was taken out after 24 h, diluted with 10 mL ethyl acetate and filtered through a celite pad. Next, the filtrate mixture was treated with 1 (M) HCl solution and stirred for ten minutes. Organic part was washed one more time with NH_4_Cl solution and concentrated under vacuum. The crude mixture was purified by column chromatography using silica gel (100–200 mesh size) and petroleum ether/ethyl acetate as the eluent.

Full experimental procedures are provided in the Supplementary Information.

## Supplementary information

Supplementary Information

## Data Availability

The authors declare that the data supporting the findings of this study are available within the article and Supplementary Information file, or from the corresponding author upon reasonable request. Crystallographic parameters for compound **5** is available free of charge from the Cambridge Crystallographic Data Centre (www.ccdc.cam.ac.uk/data_request/cif) under CCDC 1895796.
