# Supplementary material for: Imine as a linchpin approach for meta-C–H functionalization
Source: Nat Commun. 2021 Mar 2;12:1393. doi: 10.1038/s41467-021-21633-2 (PMC7925593; doi:10.1038/s41467-021-21633-2)
Supplement: Supplementary file 1 — Supplementary Information [file 41467_2021_21633_MOESM1_ESM.pdf]

Supplementary Information

Imine as a linchpin approach for *meta*-C–H functionalization

Sukdev Bag[1,2], Sadhan Jana[1,2], Sukumar Pradhan[1,2], Suman Bhowmick[1], Nupur Goswami[1], Soumya Kumar Sinha[1] & Debabrata Maiti[1]*

[1]Department of Chemistry, Indian Institute of Technology Bombay, Powai, Mumbai 400076, India

[2]These authors contributed equally.

*e-mail: dmaiti@iitb.ac.in

Table of Contents

1.	General considerations	S2
2.	Experimental section	S3-S93
2.1	Synthesis of amine-based temporary directing groups (TDGs)	S3-S13
2.2	Procedure for synthesis of TDG20	S13
2.3	Synthesis of 2-phenylbenzyldehyde derivatives	S14-S22
2.4	Synthesis of 2-phenylaniline derivatives	S23-S29
2.5	Synthesis of acrylate from bioactive alcohols	S29-S31
2.6	TDG variation for *meta*-C–H olefination	S32-S33
2.7	Optimization details for *meta*-C–H olefination of 2-phenylbenzyldehyde	S34-S43
2.8	Procedure for *meta*-olefination of 2-phenylbenzaldehyde substrates	S43
2.9	Characterization data for *meta*-olefination products of 2-phenylbenzaldehydes	S44-S71
2.10	Optimization details of *meta*-C–H olefination of 2-phenylaniline	S72-S73
2.11	Procedure for *meta*-olefination of 2-phenylaniline substrates	S73
2.12	Characterization data for *meta*-olefination products of 2-phenylanilines	S74-S85
2.13	Post-synthetic applications	S86-S94
3.	NMR Spectra	S95-S237
4.	Supplementary References	S238

1. General considerations

Reagent information: Unless otherwise stated, all reactions were carried out under air atmosphere in screw cap reaction tubes. **TDG1** and **TDG2** are commercially available and purchased from Sigma Aldrich. Palladium(II) acetate was purchased from Alfa-Aesar. All the solvents were bought from Merck, TCI and Spectrochem insure sealed bottle and were used as received. Benzyldehydes, aniline derivatives, heterocycles, boronic acids, activated olefins and other reagents were bought from Sigma Aldrich, Alfa-Aesar, TCI and Spectrochem. For column chromatography, silica gel (100–200 mesh) from SRL Co. and neutral alumina from Merck was used. A gradient elution using petroleum-ether and ethyl acetate was performed, based on Merck aluminium TLC sheets (silica gel $60F_{254}$).

Analytical Information: All isolated compounds were characterized by ^{1}H NMR, ^{13}C NMR spectroscopy, gas chromatography (GC), high resolution mass spectrometry (HRMS), infrared spectroscopy (IR), etc. Copies of the ^{1}H NMR, ^{13}C NMR can be found in the Supplementary Information. Unless otherwise stated, all Nuclear Magnetic Resonance spectra were recorded on a Bruker 400 MHz and 500 MHz instrument. Chemical shifts were quoted in parts per million (ppm) referenced to 0.0 ppm for TMS. All ^{1}H NMR experiments were measured relative to the signals for residual chloroform (7.26 ppm) in the deuterated solvent, unless otherwise stated. All ^{13}C NMR spectra were reported in ppm relative to deuterochloroform (77.23 ppm), unless otherwise stated, and all were obtained with ^{1}H decoupling. The following abbreviations (or combinations thereof) were used to explain multiplicities: s = singlet, d = doublet, t = triplet, q = quartet, m = multiplet, br = broad. Coupling constants, J, were reported in Hertz unit (Hz). NMR of the crude reaction mixtures were performed by using 1,3,5-trimethoxybenzene as the internal standard. All GCMS analysis was done by Agilent 7890A GC system connected with 5975C inert XL EI/CI MSD (with triple axis detector). High-resolution mass spectra (HRMS) were recorded on a Q-TOF micromass (YA-105) mass spectrometer and a Bruker Maxis Impact (282001.00081) in ESI mode. X-ray crystallography was recorded at Department of Chemistry, IIT Bombay.

Description of Reaction Tube:

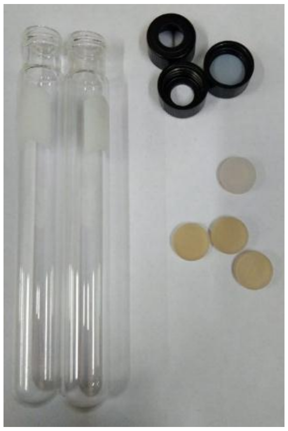

Pictorial description of reaction tube for *meta*-olefination: Fisherbrand Disposable Borosilicate Glass Tubes (16*125mm) with Threaded End (Fisher Scientific Order No. 1495935A) [left]; Kimble Black Phenolic Screw Thread Closures with Open Tops (Fisher Scientific Order No. 033407E);Thermo Scientific National PTFE/Silicone Septa for Sample Screw Thread Caps (Fisher Scientific Order No. 03394A).

2. Experimental section

2.1 General procedure A: Synthesis of amine-based temporary directing groups (TDGs)

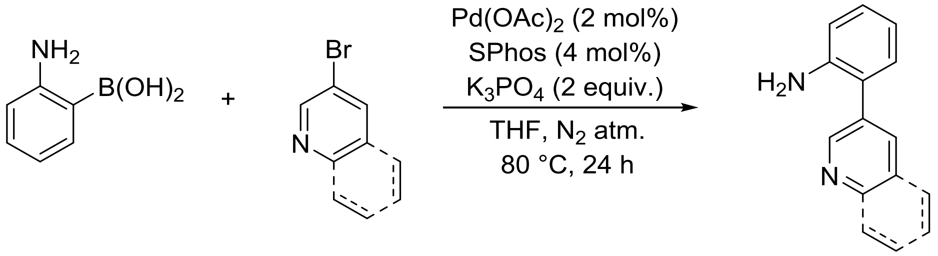

2-(pyridin-3-yl)aniline/2-(quinoline-3-yl)aniline/2-(pyrimidin-5-yl)aniline derivatives were prepared by the Suzuki cross-coupling reaction condition. A clean, oven-dried screw cap reaction tube with previously placed magnetic stir–bar was charged with 3-bromopyridine/3-bromoquinoline/5-bromopyrimidine (1 equiv., 4.0 mmol), 2-aminophenyl boronic acid (1.25 equiv., 5.0 mmol), palladium (II) acetate (2 mol%, 0.08 mmol, 18 mg), SPhos (4 mol%, 0.16 mmol, 66 mg) and K_3PO_4 (2 equiv., 8.0 mmol, 1700 mg). The cap was fitted with a rubber septum and the reaction tube was evacuated and back filled with nitrogen and this sequence was repeated three additional times. Under the positive flow of nitrogen 10 mL THF was added to the reaction mixture. The reaction mixture was vigorously stirred at 80 °C temperature for 24 h. Next, the reaction was allowed to cool at room temperature and dried using rotary evaporator. The reaction mixture was extracted thrice with ethyl acetate (3x20 mL) and brine solution (3x10 mL). The organic layer was collected and dried over anhydrous Na_2SO_4. The solvent was evaporated under reduced pressure. The crude mixture was purified by column chromatography using silica gel and petroleum-ether/ethyl acetate as the eluent.

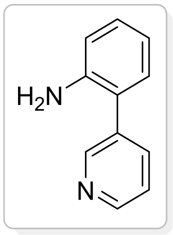

2-(pyridin-3-yl)aniline **:** Compound **TDG3** was prepared by general procedure A (4.0 mmol scale).
Eluent: petroleum ether/ethyl acetate (80/20, v/v).
Physical State: colorless oil.
Yield: 90% (613 mg isolated).
^{1}H NMR (400 MHz, $CDCl_3$) δ (ppm) 8.66 (d, J = 2.2 Hz, 1H), 8.52 (dd, J = 4.8, 1.1 Hz, 1H), 7.75 (dt, J = 7.8, 1.8 Hz, 1H), 7.30 (dd, J = 7.8, 4.9 Hz, 1H), 7.14 (td, J = 8.4, 1.2 Hz, 1H), 7.05 (dd, J = 7.6, 1.4 Hz, 1H), 6.80 (t, J = 8.4 Hz, 1H), 6.73 (d, J = 8.0 Hz, 1H), 3.72 (s, 2H).
^{13}C NMR (101 MHz, $CDCl_3$) δ (ppm) 149.97, 148.28, 143.97, 136.61, 135.37, 130.56, 129.39, 123.60, 118.80, 115.91.
IR (thin film, cm^{-1}): 1028, 1127, 1158, 1191, 1257, 1299, 1339, 1362, 1411, 1450, 1497, 1577, 1618, 2854, 2927, 3029, 3060, 3206, 3330, 3446.

HRMS (*m/z*): [M+H$^+$] calcd for $C_{11}H_{11}N_2$: 171.0917; found, 171.0918.
TLC: R_f = 0.6 (70:30 petroleum ether:EtOAc).

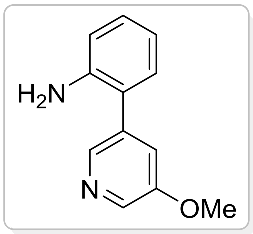

***2-(5-methoxypyridin-3-yl)aniline*:** Compound **TDG4** was prepared by general procedure A (4.0 mmol scale).
Eluent: petroleum ether/ethyl acetate (75/25, v/v).
Physical State: brown solid.
Yield: 89% (997 mg isolated).
^{1}H NMR (500 MHz, $CDCl_3$) δ (ppm) 8.27 (d, *J* = 10.2 Hz, 2H), 7.31 (s, 1H), 7.21 – 7.14 (m, 1H), 7.10 (dd, *J* = 11.8, 7.6 Hz, 1H), 6.86 – 6.73 (m, 2H), 3.87 (S, 3H), 3.77 (s, 2H).
^{13}C NMR (126 MHz, $CDCl_3$) δ (ppm) 155.90, 144.04, 142.25, 136.41, 136.09, 130.68, 129.60, 123.59, 121.27, 118.98, 116.09, 55.82.
IR (thin film, cm^{-1}): 1110, 1398, 1484, 1552, 1578, 1734, 2855, 2924, 3347.
HRMS (*m/z*): [M+Na$^+$] calcd for $C_{12}H_{12}NaN_2O$: 223.0841; found, 223.0844.
TLC: R_f = 0.25 (70:30 petroleum ether:EtOAc).

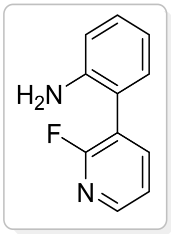

***2-(2-fluoropyridin-3-yl)aniline*:** Compound **TDG5** was prepared by general procedure A (4.0 mmol scale).
Eluent: petroleum ether/ethyl acetate (80/20, v/v).
Physical State: yellow oil.
Yield: 90% (677 mg isolated).
^{1}H NMR (500 MHz, $CDCl_3$) δ (ppm) 8.24 (d, *J* = 4.5 Hz, 1H), 7.84 (t, *J* = 8.4 Hz, 1H), 7.29 (t, *J* = 6.1 Hz, 1H), 7.23 (t, *J* = 7.6 Hz, 1H), 7.11 (d, *J* = 7.5 Hz, 1H), 6.86 (t, *J* = 7.5 Hz, 1H), 6.81 (d, *J* = 8.0 Hz, 1H), 3.66 (s, 2H).
^{13}C NMR (126 MHz, $CDCl_3$) δ (ppm) 147.21, 147.09, 144.27, 142.81, 142.77, 131.17, 130.05, 122.04, 122.00, 119.01, 116.36.
^{19}F NMR (471 MHz, $CDCl_3$) δ (ppm) -74.21.
IR (thin film, cm^{-1}): 1002, 1045, 1116, 1159, 1202, 1244, 1308, 1374, 1422, 1457, 1500, 1567, 1599, 1622, 1732, 2928, 3234, 3368, 3462.
HRMS (*m/z*): [M+H$^+$] calcd for $C_{11}H_{10}FN_2$: 189.0779; found: 189.0777.
TLC: R_f = 0.25 (70:30 petroleum ether:EtOAc).

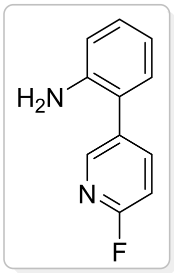

***2-(6-fluoropyridin-3-yl)aniline*:** Compound **TDG6** was prepared by general procedure A (4.0 mmol scale).
Eluent: petroleum ether/ethyl acetate (80:20, v/v).
Physical State: yellow oil.
Yield: 95% (715 mg isolated).
^{1}H NMR (500 MHz, CDCl$_3$) δ (ppm) 8.31 (s, 1H), 7.91 (td, *J* = 8.1, 2.3 Hz, 1H), 7.21 (t, *J* = 7.7 Hz, 1H), 7.08 (d, *J* = 7.5 Hz, 1H), 7.02 (dd, *J* = 8.4, 2.8 Hz, 1H), 6.85 (t, *J* = 7.4 Hz, 1H), 6.79 (d, *J* = 8.0 Hz, 1H), 3.68 (s, 2H).
^{13}C NMR (126 MHz, CDCl$_3$) δ (ppm) 148.04, 147.92, 143.96, 142.16, 142.10, 130.78, 129.75, 119.24, 116.17, 109.89, 109.59.
^{19}F NMR (471 MHz, CDCl$_3$) δ (ppm) -68.45.
IR (thin film, cm^{-1}): 1000, 1040, 1110, 1155, 1200, 1237, 1301, 1364, 1412, 1442, 1527, 1568, 1570, 1601, 1711, 2920, 3219, 3328, 3451.
HRMS (ESI): [M+H$^+$] calcd for $C_{11}H_{10}FN_2$: 189.0779; found, 189.0778.
TLC: R$_f$ = 0.4 (70:30petroleum ether:EtOAc).

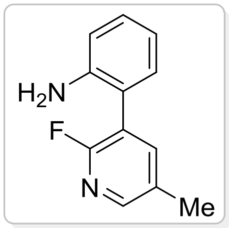

***2-(2-fluoro-5-methylpyridin-3-yl)aniline*:** Compound **TDG7** was prepared by general procedure A (4.0 mmol scale).
Eluent: petroleum ether/ethyl acetate (80/20, v/v).
Physical State: brown oil.
Yield: 79% (369 mg isolated).
^{1}H NMR (500 MHz, CDCl$_3$) δ (ppm) 8.04 (s, 1H), 7.65 (dd, *J* = 9.1, 2.1 Hz, 1H), 7.26 – 7.21 (m, 1H), 7.11 (d, *J* = 7.4 Hz, 1H), 6.86 (t, *J* = 7.8, Hz, 1H), 6.81 (d, *J* = 8.0 Hz, 1H), 3.71 (s, 2H), 2.38 (s, 3H).
^{13}C NMR (126 MHz, CDCl$_3$) δ (ppm) 159.12 (d, *J* = 237.0 Hz), 146.51 (d, *J* = 14.1 Hz), 144.11, 143.17 (d, *J* = 4.6 Hz), 131.39 (d, *J* = 4.8 Hz), 130.94, 129.74, 120.66 (d, *J* = 32.2 Hz), 119.72 (d, *J* = 4.1 Hz), 118.77, 116.13, 17.42.
^{19}F NMR (471 MHz, CDCl$_3$) δ (ppm) -74.21.

IR (thin film, cm^{-1}): 1049, 1159, 1203, 1242, 1303, 1431, 1498, 1578, 1620, 2855, 2926, 3030, 3232, 3361, 3464.
HRMS (*m/z*): [M+H$^+$] calcd for $C_{12}H_{11}FN_2$: 203.0976; found, 203.0979.
TLC: R_f = 0.7 (70:30 petroleum ether:EtOAc).

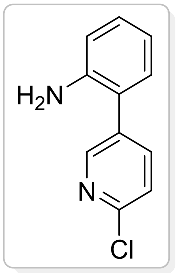

***2-(6-chloropyridin-3-yl)aniline*:** Compound **TDG8** was prepared by general procedure A (4.0 mmol scale).
Eluent: petroleum ether/ethyl acetate (80/20, v/v).
Physical State: Brown Solid.
Yield: 55% (450 mg isolated).
^{1}H NMR (500 MHz, $CDCl_3$) δ (ppm) 8.50 (d, *J* = 2.3 Hz, 1H), 7.81 (dd, *J* = 8.2, 2.5 Hz, 1H), 7.42 (d, *J* = 8.2 Hz, 1H), 7.23 (td, *J* = 8.0, 1.4 Hz, 1H), 7.10 (dd, *J* = 7.6, 1.3 Hz, 1H), 6.87 (t, *J* = 7.5 Hz, 1H), 6.81 (d, *J* = 8.0 Hz, 1H), 3.73 (s, 2H).
^{13}C NMR (126 MHz, $CDCl_3$) δ (ppm) 150.17, 149.90, 143.75, 139.43, 134.20, 130.47, 129.74, 124.29, 122.33, 119.10, 116.09.
IR (thin film, cm^{-1}): 999, 1106, 1139, 1216, 1300, 1360, 1457, 1496, 1552, 1583, 1620, 2924, 3028, 3224, 3348, 3453.
HRMS (*m/z*): [M+H$^+$] calcd for $C_{11}H_{10}ClN_2$: 207.0497; found, 207.0527.
TLC: R_f = 0.5 (70:30 petroleum ether:EtOAc).

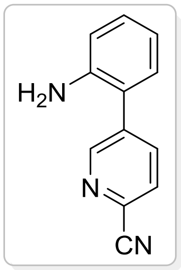

5-(2-aminophenyl)picolinonitrile: Compound **TDG9** was prepared by general procedure A (4.0 mmol scale).
Eluent: petroleum ether/ethyl acetate (70/30, v/v).
Physical State: brown solid.
Yield: 72% (562 mg isolated).
^{1}H NMR (500 MHz, $CDCl_3$) δ (ppm) 8.87 (s, 1H), 8.02 (d, *J* = 8.0 Hz, 1H), 7.79 (d, *J* = 8.0 Hz, 1H), 7.29 – 7.25 (m, 1H), 7.12 (d, *J* = 7.6 Hz, 1H), 6.91 (t, *J* = 7.5 Hz, 1H), 6.84 (d, *J* = 8.1 Hz, 1H), 3.88 (s, 2H).

^{13}C NMR (126 MHz, $CDCl_3$) δ (ppm) 151.49, 143.74, 139.20, 137.27, 131.92, 130.50, 130.45, 128.47, 121.76, 119.40, 117.32, 116.53.
IR (thin film, cm^{-1}): 1000, 1030, 1160, 1201, 1304, 1364, 1467, 1497, 1576, 1623, 2234, 2853, 2925, 3058, 3231, 3367, 3444.
HRMS (ESI): [$M+H^+$] calcd for $C_{12}H_{10}N_3$: 196.0796; found, 196.0808.
TLC: R_f = 0.3 (65:35 petroleum ether:EtOAc).

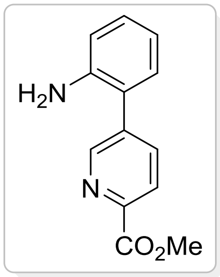

***Methyl-5-(2-aminophenyl)picolinate*:** Compound **TDG10** was prepared by general procedure A (4.0 mmol scale).
Eluent: petroleum ether/ethyl acetate (70/30, v/v).
Physical State: yellow solid.
Yield: 82% (749 mg isolated).
^{1}H NMR (500 MHz, $CDCl_3$) δ (ppm) 8.87 (d, J = 1.5 Hz, 1H), 8.21 (d, J = 8.0 Hz, 1H), 7.99 (dd, J = 8.0, 2.1 Hz, 1H), 7.23 (td, J = 7.6 Hz, 1H), 7.13 (dd, J = 7.6, 1.2 Hz, 1H), 6.88 (t, J = 7.9 Hz, 1H), 6.81 (d, J = 8.0 Hz, 1H), 4.04 (s, 3H), 3.77 (s, 2H).
^{13}C NMR (126 MHz, $CDCl_3$) δ (ppm) 165.58, 150.11, 146.33, 143.75, 138.92, 137.35, 130.50, 130.04, 125.14, 122.51, 119.16, 116.21, 52.95.
IR (thin film, cm^{-1}): 1002, 1031, 1135, 1195, 1235, 1311, 1371, 1436, 1498, 1626, 1723, 2851, 2951, 3029, 3229, 3361, 3443.
HRMS (ESI): [$M+H^+$] calcd for $C_{13}H_{13}N_2O_2$: 229.0972; found, 229.0965.
TLC: R_f = 0.45 (60:40 petroleum ether:EtOAc).

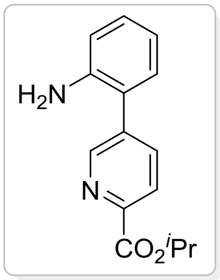

***Isopropyl 5-(2-aminophenyl)picolinate*:** Compound **TDG11** was prepared by general procedure A (4.0 mmol scale).
Eluent: petroleum ether/ethyl acetate (70/30, v/v).
Physical State: yellow oil.
Yield: 86% (882 mg isolated).

^{1}H NMR (500 MHz, $CDCl_3$) δ (ppm) 8.87 (s, 1H), 8.18 (d, *J* = 8.0 Hz, 1H), 7.95 (dd, *J* = 8.0, 1.9 Hz, 1H), 7.21 (t, *J* = 7.7 Hz, 1H), 7.10 (d, *J* = 7.5 Hz, 1H), 6.86 (t, *J* = 7.4 Hz, 1H), 6.78 (d, *J* = 8.0 Hz, 1H), 5.36 (dt, *J* = 12.5, 6.2 Hz, 1H), 3.75 (s, 2H), 1.43 (d, *J* = 6.3 Hz, 6H).
^{13}C NMR (126 MHz, $CDCl_3$) δ (ppm) 164.80, 150.32, 147.22, 143.91, 138.73, 137.42, 130.66, 130.12, 125.23, 122.81, 119.29, 116.32, 69.77, 22.05.
IR (thin film, cm^{-1}): 1044, 1101, 1141, 1182, 1235, 1304, 1373, 1452, 1500, 1626, 1732, 2938, 2982, 3230, 3369, 3446.
HRMS (ESI): [$M+H^+$] calcd for $C_{15}H_{17}N_2O_2$: 257.1278; found, 257.1278.
TLC: R_f = 0.3 (70:30 petroleum ether:EtOAc).

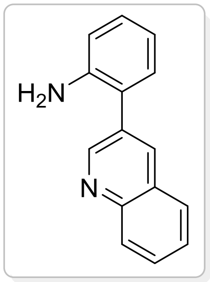

***2-(quinolin-3-yl)aniline*:** Compound **TDG12** was prepared by general procedure A (4.0 mmol scale).
Eluent: petroleum ether/ethyl acetate (75/25, v/v).
Physical State: brown solid.
Yield: 70% (616 mg isolated).
^{1}H NMR (500 MHz, $CDCl_3$) δ (ppm) 9.05 (d, *J* = 2.1 Hz, 1H), 8.28 (d, *J* = 1.5 Hz, 1H), 8.17 (d, *J* = 8.5 Hz, 1H), 7.86 (d, *J* = 8.1 Hz, 1H), 7.76 (td, *J* = 8.5, 1.5 Hz, 1H), 7.60 (t, *J* = 7.5 Hz, 1H), 7.28 – 7.21 (m, 2H), 6.92 (td, *J* = 7.5, 0.9 Hz, 1H), 6.85 (d, *J* = 8.0 Hz, 1H), 3.85 (s, 2H).
^{13}C NMR (126 MHz, $CDCl_3$) δ (ppm. 151.55, 147.16, 144.05, 135.47, 132.49, 130.87, 129.55, 129.46, 129.27, 127.96, 127.86, 127.03, 123.76, 119.09, 116.00.
IR (thin film, cm^{-1}): 1028, 1127, 1158, 1191, 1257, 1299, 1339, 1362, 1411, 1450, 1497, 1577, 1618, 2854, 2927, 3029, 3060, 3206, 3330, 3446.
HRMS (*m/z*): [$M+H^+$] calcd for $C_{15}H_{13}N_2$: 221.1073; found, 221.1075
TLC: R_f = 0.5 (70:30 petroleum ether:EtOAc).

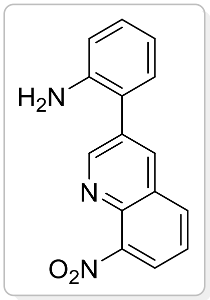

***2-(8-nitroquinolin-3-yl)aniline*:** Compound **TDG13** was prepared by general procedure A (4.0 mmol scale).
Eluent: petroleum ether/ethyl acetate (70/30, v/v).
Physical State: colorless oil.

Yield: 85% (902 mg isolated).
^{1}H NMR (400 MHz, CDCl$_3$) δ (ppm) 9.20 (d, *J* = 2.1 Hz, 1H), 8.37 (d, *J* = 2.1 Hz, 1H), 8.06 (d, *J* = 7.7 Hz, 2H), 7.65 (t, *J* = 7.9 Hz, 1H), 7.29 – 7.24 (m, 1H), 7.19 (dd, *J* = 7.6, 1.5 Hz, 1H), 6.92 (td, *J* = 7.5, 1.0 Hz, 1H), 6.85 (dd, *J* = 8.1, 0.8 Hz, 1H), 3.73 (d, *J* = 2.7 Hz, 2H).
^{13}C NMR (101 MHz, CDCl$_3$) δ (ppm) 154.14, 144.15, 138.54, 135.53, 134.77, 132.28, 131.00, 130.25, 129.14, 125.95, 123.94, 122.76, 121.58, 119.59, 116.52.
IR (thin film, cm^{-1}): 1028, 1127, 1158 1191, 1257, 1299, 1339, 1345, 1362, 1385, 1411, 1450, 1497, 1577, 1560, 1515, 1618, 2854, 2927, 3029, 3060, 3206, 3330, 3446.
HRMS (ESI): [M+H$^+$] calcd for $C_{15}H_{12}N_3O_2$: 266.0924; found, 266.0929.
TLC: R$_f$ = 0.3 (70:30 petroleum ether:EtOAc).

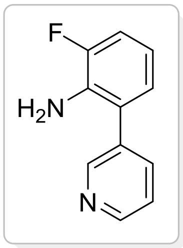

***2-fluoro-6-(pyridin-3-yl)aniline*:** Compound **TDG14** was prepared by general procedure A (4.0 mmol scale).
Eluent: petroleum ether/ethyl acetate (75:25, v/v).
Physical State: white solid.
Yield: 70% (527 mg isolated).
^{1}H NMR (400 MHz, CDCl$_3$) δ (ppm) 7.59 (d, *J* = 7.6 Hz, 1H), 7.41 (t, *J* = 7.3 Hz, 1H), 7.37 – 7.30 (m, 2H), 7.20 (ddd, *J* = 7.4, 3.5, 1.4 Hz, 1H), 6.61 (d, *J* = 8.4 Hz, 2H).
^{13}C NMR (101 MHz, CDCl$_3$) δ (ppm) 157.49, 142.95 (d, *J* = 31.8 Hz), 135.99 (d, *J* = 17.6 Hz), 132.43 (d, *J* = 3.6 Hz), 130.98 (d, *J* = 6.1 Hz), 128.85, 128.28, 126.23, 119.98 (d, *J* = 7.0 Hz), 103.15.
IR (thin film, cm^{-1}): 1002, 1036, 1110, 1172, 1247, 1471, 1589, 1735, 2851, 2925, 3050.
^{19}F NMR (471 MHz, CDCl$_3$) δ (ppm) -75.19.
HRMS (ESI): [M+Na$^+$] calcd for $C_{11}H_9FN_2Na$: 211.0642; found, 211.0638.
TLC: R$_f$ = 0.35 (70:30 petroleum ether:EtOAc).

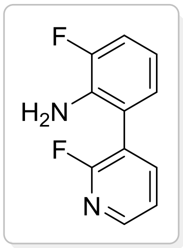

***2-fluoro-6-(2-fluoropyridin-3-yl)aniline*:** Compound **TDG15** was prepared by general procedure A (4.0 mmol scale).
Eluent: petroleum ether/ethyl acetate (75:25, v/v).
Physical State: white solid.

Yield: 75% (618 mg isolated).
^{1}H NMR (400 MHz, CDCl$_3$) δ (ppm) 8.24 (dd, *J* = 3.2, 1.2 Hz, 1H), 7.83 (ddd, *J* = 9.4, 7.4, 2.0 Hz, 1H), 7.29 (ddd, *J* = 7.1, 4.9, 1.9 Hz, 1H), 7.05 (ddd, *J* = 10.8, 8.1, 1.3 Hz, 1H), 6.90 (d, *J* = 7.6 Hz, 1H), 6.79 – 6.72 (m, 1H), 3.77 (s, 2H).
^{13}C NMR (101 MHz, CDCl$_3$) δ (ppm) 160.51 (d, *J* = 239.6 Hz), 151.83 (d, *J* = 239.1 Hz), 147.34 (d, *J* = 14.4 Hz), 142.45 (d, *J* = 4.6 Hz), 133.06 (d, *J* = 13.3 Hz), 126.14 (d, *J* = 2.2 Hz), 121.89 (d, *J* = 4.4 Hz), 121.41, 120.55 (dd, *J* = 3.4 , 3.3 Hz), 117.98 (d, *J* = 7.8 Hz), 115.27 (d, *J* = 19.1 Hz).
^{19}F NMR (471 MHz, CDCl$_3$) δ (ppm) -68.37, -133.89.
IR (thin film, cm^{-1}): 1028, 1067, 1114, 1143, 1180, 1208, 1244, 1274, 1426, 1476, 1568, 1602, 1631, 2926, 3063, 3362.
HRMS (**ESI**): [M+H$^+$] calcd for $C_{11}H_9F_2N_2$: 207.0734; found, 207.0728.
TLC: R$_f$ = 0.3 (70:30 petroleum ether:EtOAc).

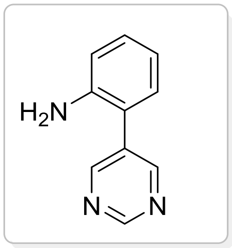

***2-(pyrimidin-5-yl)aniline*:** Compound **TDG16** was prepared by general procedure A (4.0 mmol scale).
Eluent: petroleum ether/ethyl acetate (70:30, v/v).
Physical State: yellow solid.
Yield: 85% (582 mg isolated).
^{1}H NMR (400 MHz, CDCl$_3$) δ (ppm) 9.19 (s, 1H), 8.88 (s, 2H), 7.25 (t, *J* = 7.2 Hz, 1H), 7.09 (d, *J* =7.4 Hz, 1H), 6.88 (t, *J* = 7.3 Hz, 1H), 6.82 (d, *J* = 8.0 Hz, 1H), 3.79 (s, 2H).
^{13}C NMR (126 MHz, CDCl$_3$) δ (ppm) 190.63, 158.04, 156.71, 137.22, 134.19, 133.77, 132.28, 131.31, 130.57, 129.61.
IR (thin film, cm^{-1}): 1000, 1057, 1108, 1160, 1258, 1302, 1408, 1457, 1497, 1550, 1577, 1629, 1895, 2331, 2859, 2936, 3036, 3227, 3341, 3436.
HRMS (*m/z*): [M+H$^+$] calcd for $C_{10}H_{10}N_3$: 172.0869; found, 172.0868
TLC: R$_f$ = 0.3 (70:30 petroleum ether:EtOAc).

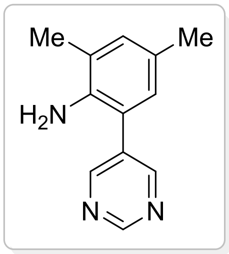

***2,4-dimethyl-6-(pyrimidin-5-yl)aniline*:** Compound **TDG17** was prepared by general procedure A (4.0 mmol scale).
Eluent: petroleum ether/ethyl acetate (70:30, v/v).

Physical State: yellow solid.
Yield: 76% (606 mg isolated).
^{1}H NMR (400 MHz, $CDCl_3$) δ (ppm) 9.20 (s, 1H), 8.86 (s, 2H), 6.98 (d, *J* = 0.6 Hz, 1H), 6.78 (d, *J* = 1.3 Hz, 1H), 3.54 (s, 2H), 2.27 (s, 3H), 2.21 (s, 3H).
^{13}C NMR (126 MHz, $CDCl_3$) δ (ppm) 157.32, 157.12, 139.52, 133.90, 132.16, 128.71, 128.01, 123.30, 119.80, 20.31, 17.80.
IR (thin film, cm^{-1}): 1184.29, 1412.78, 1484.66, 1552.02, 1627.14, 1734.21, 2855.33, 2924.64, 3367.73.
HRMS (ESI): [$M+H^+$] calcd for $C_{12}H_{14}N_3$: 200.1182; found, 200.1178.
TLC: R_f = 0.26 (70:30 petroleum ether:EtOAc).

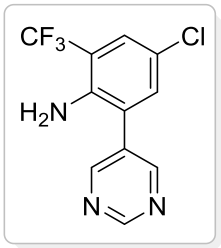

***4-chloro-2-(pyrimidin-5-yl)-6-(trifluoromethyl)aniline*:** Compound **TDG18** was prepared by general procedure A (4.0 mmol scale).
Eluent: petroleum ether/ethyl acetate (75:25, v/v).
Physical State: colorless oil.
Yield: 70% (766 mg isolated).
^{1}H NMR (400 MHz, $CDCl_3$) δ (ppm) 9.28 (s, 1H), 8.83 (s, 2H), 7.53 (d, *J* = 2.4 Hz, 1H), 7.21 (d, *J* = 2.4 Hz, 1H), 4.21 (s, 2H).
^{13}C NMR (126 MHz, $CDCl_3$) δ (ppm) 158.77, 157.37, 140.96, 134.02, 131.29, 127.69, 127.65, 125.14, 123.54, 122.98, 116.19 (q, *J* = 272 Hz, CF_3).
IR (thin film, cm^{-1}): 1019, 1132, 1213, 1242, 1345, 1430, 1499, 1578, 1620, 2877, 2825, 2930, 3131, 3261, 3474.
HRMS (*m/z*): [$M+H^+$] calcd for $C_{11}H_8ClF_3N_3$: 274.0353; found, 274.0359.
TLC: R_f = 0.2 (70:30 petroleum ether:EtOAc).

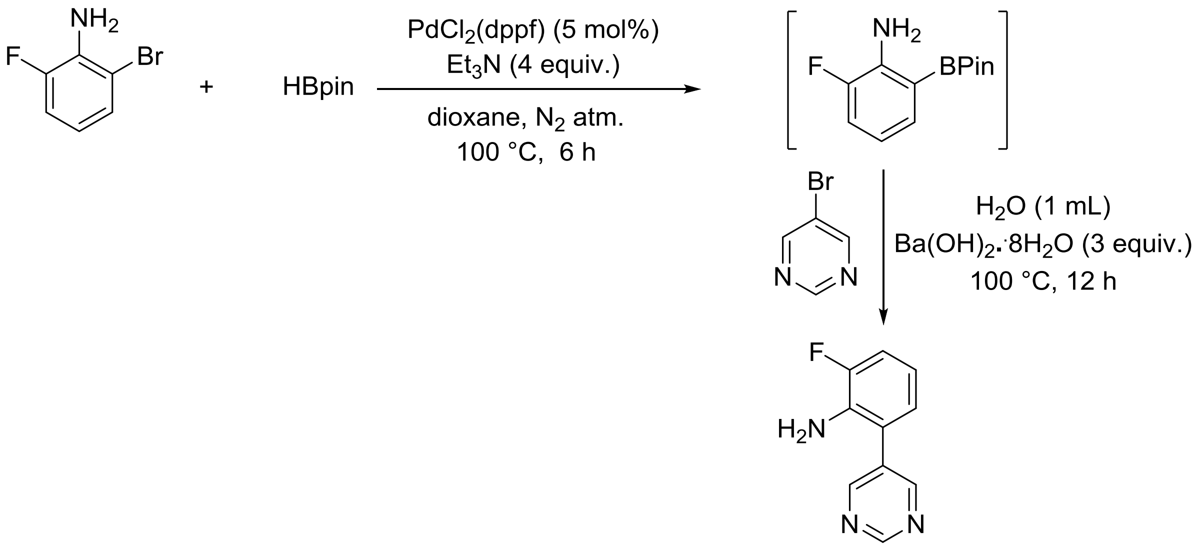

TDG19

Procedure for synthesis of TDG19: To a solution of 2-fluoro-6-(pyrimidin-5-yl)aniline (1.0 equiv., 2.5 mmol, 285 µL) in dioxane (10 mL) were added Et_3N (4.0 equiv., 10.0 mmol, 1.4 mL), $PdCl_2$(dppf) (5 mol%, 0.125 mmol, 92 mg), and pinacolborane (3.0 equiv., 1.0 mL, 7.5 mmol), dropwise. The mixture was stirrer at 100 °C for 6 h, then cool to room temperature, and water (1.0 mL), $Ba(OH)_2.8H_2O$ (3.0 equiv., 7.5 mmol, 2.37 g), and 5-bromopyrimidine (0.92 equiv., 2.3 mmol, 366 mg) were successively added. The mixture was stirrer at 100 °C for 12 h before addition of water (25 mL) at room temperature. The mixture was filtered through Celite. The eluent was extracted with ethyl acetate and the organic layer was purified by column chromatography using silica gel deactivated with 10 mol% Et_3N in hexane. 2-fluoro-6-(pyrimidin-5-yl)aniline was isolated as white solid.

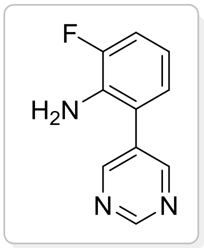

***2-fluoro-6-(pyrimidin-5-yl)aniline*:** Compound **TDG19** was prepared by above procedure (2.5 mmol scale).

Eluent: petroleum ether/ethyl acetate (70/30, v/v).

Physical State: white solid.

Yield: 81% (383 mg isolated).

^{1}H NMR (400 MHz, $CDCl_3$) δ (ppm) 9.17 (s, 1H), 8.85 (s, 2H), 7.06 (ddd, *J* = 10.7, 8.1, 1.4 Hz, 1H), 6.88 (d, *J* = 7.7 Hz, 1H), 6.79 (td, *J* = 7.9, 5.1 Hz, 1H), 3.86 (s, 2H).

^{13}C NMR (101 MHz, $CDCl_3$) δ (ppm) 157.70, 156.79, 150.65 (s), 132.81 (d, *J* = 13.4 Hz), 132.39 (d, *J* = 3.2 Hz), 125.63 (d, *J* = 3.2 Hz), 121.89 (d, *J* = 3.5 Hz), 118.54 (d, *J* = 7.8 Hz), 115.74 (d, *J* = 19.2 Hz).

^{19}F NMR (471 MHz, $CDCl_3$) δ (ppm) -133.51.

IR (thin film, cm^{-1}): 1062, 1182, 1215, 1246, 1277, 1299, 1344, 1412, 1488, 1555, 1580, 1652, 2855, 2925, 3219, 3339, 3433.

HRMS (ESI): [M+H$^+$] calcd for $C_{10}H_9FN_3$: 190.0771; found, 190.0775.
TLC: R_f = 0.4 (70/30 petroleum ether:EtOAc).

2.2 Procedure for synthesis of TDG20

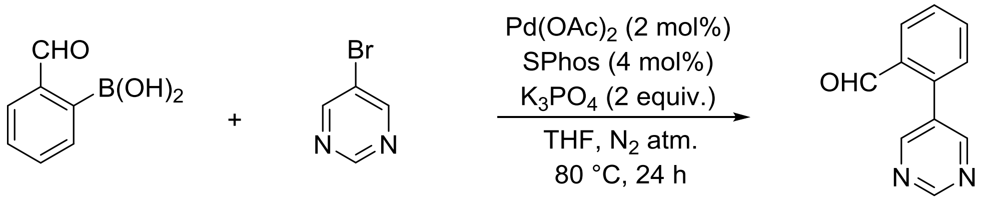

TDG20

2-(pyrimidin-5-yl)benzaldehyde was prepared by the Suzuki cross-coupling reaction condition. A clean, oven-dried screw cap reaction tube with previously placed magnetic stir–bar was charged with 5-bromopyrimidine (1 equiv., 4.0 mmol, 636 mg), (2-formylphenyl)boronic acid (1.25 equiv., 5.0 mmol, 750 mg), palladium (II) acetate (2 mol%, 0.08 mmol, 18 mg), SPhos (4 mol%, 0.16 mmol, 66 mg) and K_3PO_4 (2 equiv., 8.0 mmol, 1700 mg). The cap was fitted with a rubber septum and the reaction tube was evacuated and back filled with nitrogen and this sequence was repeated three additional times. Under the positive flow of nitrogen 15 mL THF was added to the reaction mixture. The reaction mixture was vigorously stirred at 80 °C temperature for 24 h. Next, the reaction was allowed to cool at room temperature and dried using rotary evaporator. The reaction mixture was extracted thrice with ethyl acetate (3x20 mL) and brine solution (3x10 mL). The organic layer was collected and dried over anhydrous Na_2SO_4. The solvent was evaporated under reduced pressure. The crude mixture was purified by column chromatography using silica gel and petroleum-ether/ethyl acetate as the eluent.

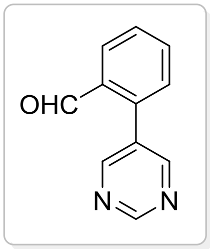

***2-(pyrimidin-5-yl)benzaldehyde*:** Compound **TDG20** was prepared by above procedure.
Eluent: petroleum ether/ethyl acetate (70/30, v/v).
Physical State: white solid.
Yield: 87% (641 mg isolated).
^{1}H NMR (500 MHz, $CDCl_3$) δ (ppm).10.01 (s, 1H), 9.29 (s, 1H), 8.79 (s, 2H), 8.08 (dd, *J* = 7.7, 1.1 Hz, 1H), 7.76 (td, *J* = 7.5, 1.3 Hz, 1H), 7.67 (t, *J* = 7.5 Hz, 1H), 7.42 (d, *J* = 7.6 Hz, 1H).
^{13}C NMR (126 MHz, $CDCl_3$) δ (ppm). 190.63, 158.04, 156.71, 137.22, 134.19, 133.77, 132.28, 131.31, 130.57, 129.61.
IR (thin film, cm^{-1}): 1000, 1055, 1200, 1266, 1355, 1416, 1549, 1597, 1654, 1689, 2764, 2869, 2923, 3042.
HRMS (*m/z*): [M+H$^+$] calcd for $C_{11}H_9N_2O$: 185.0709; found, 185.0710.
TLC: R_f = 0.3 (70:30 petroleum ether:EtOAc).

2.3 General procedure B: Synthesis of 2-phenylbenzyldehyde derivatives

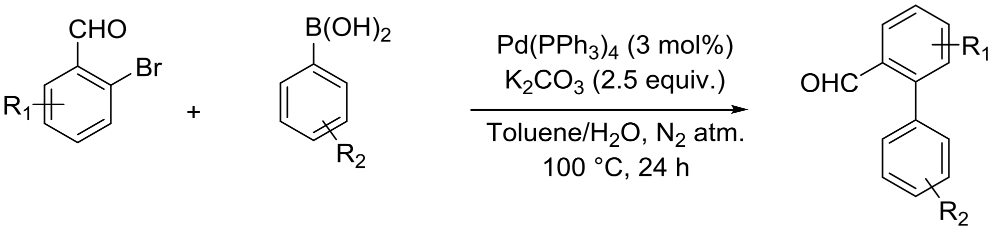

Under an inert atmosphere, into a three-necked flask was charged with (1.0 equiv., 4.0 mmol, 740 mg) of 2-bromobenzaldehyde, (1.25 equiv., 5.0 mmol, 609 mg) of arylboronic acid, (2.0 equiv., 8.0 mmol, 1.7 g) of potassium carbonate, 8 mL of toluene and 8 mL of ion exchanged water to obtain a mixed solution, and argon was bubbled through this mixed solution for 20 minutes while stirring at room temperature. Subsequently, to this mixed solution was added 139 mg (3 mol%, 0.12 mmol) of tetrakis(triphenylphosphine)palladium, further, argon was bubbled through the solution for 10 minutes while stirring at room temperature. The mixed solution was heated up to 100 °C and reacted for 25 hours. After cooling to room temperature, the organic phase was evaporated under reduced pressure. The crude mixture was purified by column chromatography using silica gel and petroleum-ether/ethyl acetate (90/10, v/v) as the eluent.

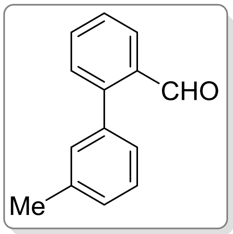

***3'-methyl-[1,1'-biphenyl]-2-carbaldehyde*:** Compound **1a** was prepared by general procedure B (4.0 mmol scale).
Eluent: petroleum ether
Physical State: yellow oil.
Yield: 97% (761 mg isolated).
^{1}H NMR (500 MHz, $CDCl_3$) δ (ppm) 10.02 (s, 1H), 8.05 (dd, *J* = 7.8, 1.0 Hz, 1H), 7.66 (td, *J* = 7.5, 1.4 Hz, 1H), 7.52 (t, *J* = 7.6 Hz, 1H), 7.47 (d, *J* = 7.7 Hz, 1H), 7.39 (t, *J* = 7.5 Hz, 1H), 7.28 (d, *J* = 3.7 Hz, 1H), 7.24 – 7.20 (m, 2H), 2.45 (s, 3H).
^{13}C NMR (126 MHz, $CDCl_3$) δ (ppm) 192.65, 146.19, 138.18, 137.71, 133.75, 133.52, 130.85, 130.75, 128.87, 128.32, 127.67, 127.47, 127.28, 21.44.
IR (thin film, cm^{-1}): 3032, 2922, 2847, 2751, 1692, 1648, 1597, 1471, 1392, 1257, 1197, 1160.76, 1103, 1035, 1000.
HRMS (m/z): [M+H+] calcd for $C_{14}H_{13}O$: 197.0960; found, 197.0963.
TLC: R_f = 0.7 (98:2 petroleum ether:EtOAc).

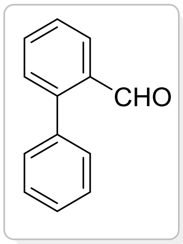

***[1,1'-biphenyl]-2-carbaldehyde*:** Compound **1b** was prepared by general procedure B (4.0 mmol scale).
Eluent: petroleum ether.
Physical State: yellow oil.
Yield: 95% (692 mg isolated).
^{1}H NMR (400 MHz, $CDCl_3$) δ (ppm) 9.96 (s, 1H), 8.01 (d, *J* = 7.8 Hz, 1H), 7.56 (t, *J* = 7.5 Hz, 1H), 7.46 – 7.34 (m, 5H), 7.33 – 7.28 (m, 2H).
^{13}C NMR (101 MHz, $CDCl_3$) δ (ppm) 191.89, 145.65, 137.51, 133.49, 133.34, 130.59, 129.90, 128.24, 127.92, 127.56, 127.34.
IR (thin film, cm^{-1}): 3028, 2920, 2848, 2761, 1592, 1638, 1588, 1471, 1392, 1257, 1187, 1170, 1100, 1033, 1070.
HRMS (*m/z*): [M+H$^+$] calcd for $C_{13}H_{11}O$: 183.0804; found, 183.0805.
TLC: R_f = 0.7 (98:2 petroleum ether:EtOAc).

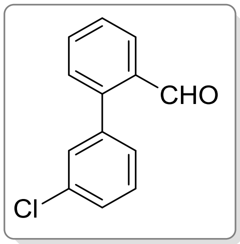

***3'-chloro-[1,1'-biphenyl]-2-carbaldehyde*:** Compound **1c** was prepared by general procedure B (4.0 mmol scale).
Eluent: petroleum ether.
Physical State: colorless oil.
Yield: 87% (479 mg isolated).
^{1}H NMR (500 MHz, $CDCl_3$) δ (ppm) 9.98 (s, 1H), 8.03 (dd, *J* = 7.8, 1.2 Hz, 1H), 7.65 (td, *J* = 7.5, 1.4 Hz, 1H), 7.53 (t, *J* = 7.6 Hz, 1H), 7.43 – 7.39 (m, 4H), 7.25 (t, *J* = 1.8 Hz, 1H).
^{13}C NMR (126 MHz, $CDCl_3$) δ (ppm) 192.01, 144.50, 139.84, 134.73, 133.91, 133.86, 130.86, 130.08, 129.82, 128.58, 128.54, 128.49, 128.07.
IR (thin film, cm^{-1}): 1023, 1080, 1104, 1161, 1195, 1253, 1393, 1464, 1560, 1593, 1691, 1947, 2753, 2849, 3063.
HRMS (*m/z*): [M+H$^+$] calcd for $C_{13}H_{10}ClO$: 217.0414; found, 217.0420.
TLC: R_f = 0.7 (98:2 petroleum ether:EtOAc).

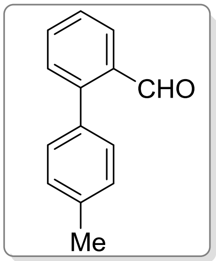

***4'-methyl-[1,1'-biphenyl]-2-carbaldehyde*:** Compound **1d** was prepared by general procedure B (0.4 mmol scale).
Eluent: petroleum ether/ethyl acetate (98/2, v/v).
Physical State: yellow oil.
Yield: 95% (745 mg isolated).
^{1}H NMR (500 MHz, $CDCl_3$) δ (ppm) 10.00 (s, 1H), 8.02 (dd, J = 7.8, 1.0 Hz, 1H), 7.66 – 7.59 (m, 1H), 7.48 (t, J = 7.5 Hz, 1H), 7.44 (d, J = 7.7 Hz, 1H), 7.28 (s, 4H), 2.44 (s, 3H).
^{13}C NMR (126 MHz, $CDCl_3$) δ (ppm) 192.84, 146.21, 138.25, 134.99, 133.93, 133.73, 130.98, 130.22, 129.35, 127.74, 127.73, 21.38.
IR (thin film, cm^{-1}): 3026, 2848, 2922, 2751, 1910, 1690, 1596, 1475, 1392, 1448, 1516, 1160, 1113, 11043, 1005, 958.
HRMS (*m/z*): [M+Na$^+$] calcd for $C_{14}H_{12}NaO$: 219.0780; found, 219.0783.
TLC: R$_f$ = 0.5 (95:5 petroleum ether:EtOAc).

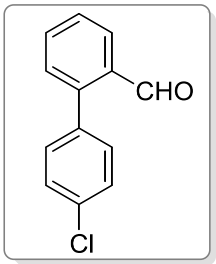

4'-chloro-[1,1'-biphenyl]-2-carbaldehyde: Compound **1e** was prepared by general procedure B (4.0 mmol scale).
Eluent: petroleum ether/ethyl acetate (98/2, v/v).
Physical State: yellow oil.
Yield: 85% (736 mg isolated).
^{1}H NMR (500 MHz, $CDCl_3$) δ (ppm) 9.97 (s, 1H), 8.03 (dd, J = 7.8, 1.1 Hz, 1H), 7.65 (td, J = 7.5, 1.4 Hz, 1H), 7.52 (t, J = 7.6 Hz, 1H), 7.47 – 7.44 (m, 2H), 7.41 (d, J = 7.7 Hz, 1H), 7.34 – 7.30 (m, 2H).
^{13}C NMR (126 MHz, $CDCl_3$) δ (ppm) 192.13, 144.73, 136.45, 134.71, 133.90, 131.48, 130.89, 128.88, 128.35, 128.14.
IR (thin film, cm^{-1}): 1000, 1033, 1085, 1119, 1176, 1300, 1379, 1458, 1473, 1598, 1697, 2725, 2826, 3019, 3061.
HRMS (*m/z*): [M+H$^+$] calcd for $C_{13}H_{10}ClO$: 217.0414; found, 217.0420.
TLC: R$_f$ = 0.5 (95:5 petroleum ether:EtOAc).

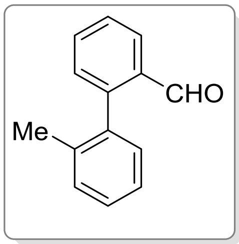

***2'-methyl-[1,1'-biphenyl]-2-carbaldehyde* :** Compound **1f** was prepared by general procedure B (4.0 mmol scale).
Eluent: petroleum ether/ethyl acetate (98/2, v/v).
Physical State: colorless oil.
Yield: 87% (683 mg isolated).
^{1}H NMR (500 MHz, $CDCl_3$) δ (ppm) 9.81 (s, 1H), 8.08 (d, *J* = 7.8 Hz, 1H), 7.66 (t, *J* = 7.8 Hz, 1H), 7.52 (t, *J* = 7.6 Hz, 1H), 7.37 – 7.32 (m, 3H), 7.29 (t, *J* = 7.3 Hz, 1H), 7.22 (d, *J* = 7.5 Hz, 1H), 2.14 (s, 3H).
^{13}C NMR (126 MHz, $CDCl_3$) δ (ppm) 192.18, 145.69, 137.55, 136.18, 133.89, 133.77, 130.82, 130.24, 130.15, 128.35, 127.88, 127.13, 125.75, 20.36.
IR (thin film, cm^{-1}): 3020, 3062, 2923, 2844, 2748, 1693, 1596, 1474, 1448, 1391, 1253, 1194, 1160, 1120, 1098, 1036, 1006.
HRMS (ESI): [M-H$^+$] calcd for $C_{14}H_{11}O$: 195.0810; found, 195.0786.
TLC: R_f = 0.5 (95:5 petroleum ether:EtOAc).

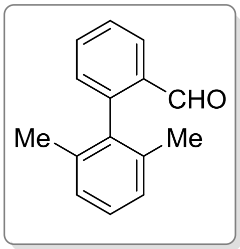

***2',6'-dimethyl-[1,1'-biphenyl]-2-carbaldehyde* :** Compound **1g** was prepared by general procedure B (4.0 mmol scale).
Eluent: petroleum ether/ethyl acetate (98/2, v/v).
Physical State: colorless oil.
Yield: 96% (807 mg isolated).
^{1}H NMR (500 MHz, $CDCl_3$) δ (ppm) 9.66 (s, 1H), 8.06 (dd, *J* = 7.8, 1.0 Hz, 1H), 7.68 (td, *J* = 7.5, 1.3 Hz, 1H), 7.51 (t, *J* = 7.6 Hz, 1H), 7.22 (dd, *J* = 7.4, 2.6 Hz, 2H), 7.14 (d, *J* = 7.6 Hz, 2H), 1.98 (s, 6H).
^{13}C NMR (126 MHz, $CDCl_3$) δ (ppm) 192.38, 145.26, 137.28, 136.46, 134.57, 133.77, 130.62, 128.17, 127.97, 127.61, 127.47, 21.06.
IR (thin film, cm^{-1}): 3022, 2923, 2841, 2745, 1734, 1693, 1651, 1597, 1462, 1390, 1256, 1195, 1159, 1107, 1045, 1004.
HRMS (ESI): [M+Na$^+$] calcd for $C_{15}H_{14}NaO$: 233.0937; found, 233.0934.
TLC: R_f = 0.5 (95:5 petroleum ether:EtOAc).

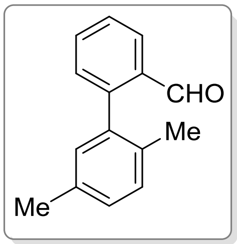

***4'-methoxy-2',5'-dimethyl-[1,1'-biphenyl]-2-carbaldehyde*:** Compound **1h** was prepared by general procedure B (4.0 mmol scale).
Eluent: petroleum ether/ethyl acetate (97/3, v/v).
Physical State: colorless oil.
Yield: 92% (774 mg isolated).
^{1}H NMR (500 MHz, $CDCl_3$) δ (ppm) 9.79 (s, 1H), 8.04 (dd, *J* = 7.8, 1.0 Hz, 1H), 7.63 (td, *J* = 7.5, 1.3 Hz, 1H), 7.49 (t, *J* = 7.6 Hz, 1H), 7.31 (dd, *J* = 7.6, 0.5 Hz, 1H), 7.19 (d, *J* = 7.8 Hz, 1H), 7.15 (dd, *J* = 7.8, 0.9 Hz, 1H), 7.03 (s, 1H), 2.36 (s, 3H), 2.07 (s, 3H).
^{13}C NMR (126 MHz, $CDCl_3$) δ (ppm) 192.49, 146.02, 137.43, 135.26, 133.96, 133.78, 133.12, 131.01, 130.87, 130.10, 129.12, 127.82, 127.07, 20.99, 19.91.
IR (thin film, cm^{-1}): 3018, 2922, 2840, 2747, 1693, 1597, 1501, 1476, 1447, 1390, 1257, 1197, 1138.
HRMS (*m/z*): [M+Na^+] calcd for $C_{15}H_{14}NaO$: 233.0937; found, 233.0928.
TLC: R_f = 0.4 (95:5 petroleum ether:EtOAc).

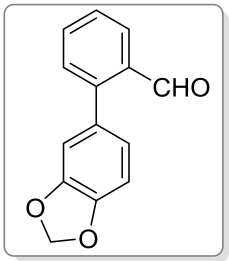

***2-(benzo[d][1,3]dioxol-5-yl)benzaldehyde*:** Compound **1i** was prepared by general procedure B (4.0 mmol scale).
Eluent: petroleum ether/ethyl acetate (98/2, v/v).
Physical State: colorless oil.
Yield: 97% (878 mg isolated).
^{1}H NMR (500 MHz, $CDCl_3$) δ (ppm) 9.97 (s, 1H), 7.96 (dd, *J* = 7.8, 0.6 Hz, 1H), 7.60 – 7.52 (m, 1H), 7.42 (dt, *J* = 14.2, 3.6 Hz, 1H), 7.37 (d, *J* = 7.7 Hz, 1H), 6.90 – 6.81 (m, 2H), 6.81 – 6.72 (m, 1H), 5.99 (dd, *J* = 2.5, 1.5 Hz, 2H).
^{13}C NMR (126 MHz, $CDCl_3$) δ (ppm) 192.39, 147.93, 147.83, 145.53, 133.90, 133.56, 131.58, 130.74, 127.65, 127.61, 124.15, 110.30, 108.25, 101.50.
IR (thin film, cm^{-1}): 3067, 2896, 2852, 2752, 1850, 1689, 1596, 1503, 1472, 1393, 1341, 1245, 1221, 1193, 1108, 1037.
HRMS (ESI): [M+H^+] calcd for $C_{14}H_{10}NaO_3$: 249.0522; found, 249.0516.
TLC: R_f = 0.5 (98:2 petroleum ether:EtOAc).

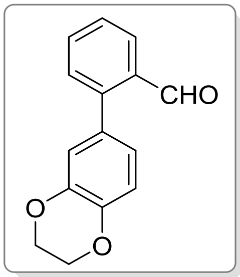

***2-(2,3-dihydrobenzo[b][1,4]dioxin-6-yl)benzaldehyde*:** Compound **1j** was prepared by general procedure B (4.0 mmol scale).
Eluent: petroleum ether/ethyl acetate (95/5, v/v).
Physical State: colorless oil.
Yield: 92% (884 mg isolated).
^{1}H NMR (400 MHz, CDCl$_3$) δ (ppm) 10.00 (s, 1H), 7.97 (d, *J* = 7.7 Hz, 1H), 7.57 (t, *J* = 7.3 Hz, 1H), 7.43 (d, *J* = 7.6 Hz, 1H), 7.39 (d, *J* = 7.8 Hz, 1H), 6.91 (t, *J* = 7.8 Hz, 2H), 6.80 (dd, *J* = 8.2, 1.7 Hz, 1H), 4.28 (s, 4H).
^{13}C NMR (101 MHz, CDCl$_3$) δ (ppm) 192.59, 145.44, 143.81, 143.47, 133.72, 133.51, 130.98, 130.70, 127.50, 127.47, 123.46, 118.92, 117.25, 64.43, 64.37.
IR (thin film, cm^{-1}): 1068, 1100, 1127, 1195, 1224, 1245, 1281, 1311, 1391, 1416, 1450, 1475, 1509, 1583, 1652, 1690, 2750, 2926.
HRMS (*m/z*): [M+H$^+$] calcd for $C_{15}H_{13}O_3$: 241.0859; found, 241.0862.
TLC: R$_f$ = 0.5 (90:10 petroleum ether:EtOAc).

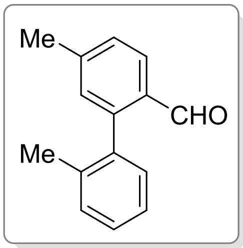

***2',5-dimethyl-[1,1'-biphenyl]-2-carbaldehyde*:** Compound **1k** was prepared by general procedure B (4.0 mmol scale).
Eluent: petroleum ether/ethyl acetate (97/3, v/v).
Physical State: brown oil.
Yield: 88% (740 mg isolated).
^{1}H NMR (500 MHz, CDCl$_3$) δ (ppm) 9.94 (d, *J* = 0.6 Hz, 1H), 7.93 (d, *J* = 8.0 Hz, 1H), 7.30 – 7.26 (m, 5H), 7.24 (s, 1H), 2.46 (s, 3H), 2.43 (s, 3H).
^{13}C NMR (126 MHz, CDCl$_3$) δ (ppm) 192.32, 146.19, 144.50, 137.93, 134.98, 131.52, 131.40, 130.00, 129.11, 128.49, 127.71, 21.85, 21.21.
IR (thin film, cm^{-1}): 1038, 1121, 1182, 1208, 1256, 1394, 1450, 1488, 1514, 1602, 1682, 2752, 2847, 2921, 2954, 3026.
HRMS (*m/z*): [M+Na$^+$] calcd for $C_{15}H_{14}NaO$: 233.0936; found, 233.0939
TLC: R$_f$ = 0.4 (95:5 petroleum ether:EtOAc).

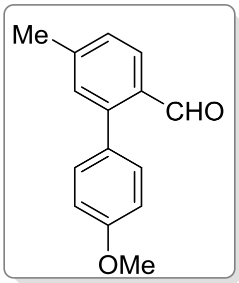

***4'-methoxy-5-methyl-[1,1'-biphenyl]-2-carbaldehyde*:** Compound **1l** was prepared by general procedure B (4.0 mmol scale).
Eluent: petroleum ether/ethyl acetate (97/3, v/v).
Physical State: brown oil.
Yield: 95% (1075 mg isolated).
^{1}H NMR (500 MHz, CDCl$_3$) δ (ppm) 9.94 (s, 1H), 7.91 (d, J = 7.9 Hz, 1H), 7.29 (d, J = 8.5 Hz, 2H), 7.27 (d, J = 4.5 Hz, 1H), 7.22 (s, 1H), 6.99 (d, J = 8.5 Hz, 2H), 3.87 (s, 3H), 2.45 (s, 3H).
^{13}C NMR (126 MHz, CDCl$_3$) δ (ppm) 192.55, 159.78, 145.99, 144.67, 131.66, 131.55, 131.41, 130.35, 128.50, 127.91, 114.02, 55.56, 22.01.
IR (thin film, cm^{-1}): 3354, 2957, 2934, 2839, 2752, 2544, 2297, 2052, 1893, 1682, 1603, 1514, 1462, 1394, 1295, 1266, 1244, 1209, 1120, 1028.
HRMS (*m/z*): [M+Na$^+$] calcd for $C_{15}H_{14}NaO_2$: 249.0886; found, 249.0889.
TLC: R$_f$ = 0.6 (95:5 petroleum ether:EtOAc).

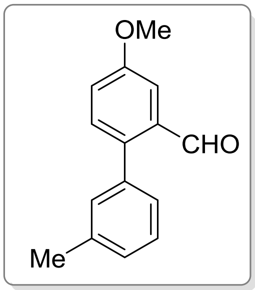

***3',4-dimethoxy-[1,1'-biphenyl]-2-carbaldehyde*:** Compound **1m** was prepared by general procedure B (4.0 mmol scale).
Eluent: petroleum ether/ethyl acetate (95/5, v/v).
Physical State: pale yellow oil.
Yield: 89% (805 mg isolated).
^{1}H NMR (500 MHz, CDCl$_3$) δ (ppm) 9.95 (s, 1H), 7.51 (s, 1H), 7.35 (dd, J = 8.6, 2.0 Hz, 2H), 7.25 – 7.12 (m, 4H), 3.90 (s, 3H), 2.42 (s, 3H).
^{13}C NMR (126 MHz, CDCl$_3$) δ (ppm) 192.66, 159.26, 139.51, 138.28, 137.61, 134.68, 132.24, 131.16, 128.72, 128.44, 127.59, 121.57, 109.93, 55.78, 21.61.
IR (thin film, cm^{-1}): 3343, 2959, 2877, 2738, 2594, 2349, 2036, 1915, 1702, 1548, 1462, 1387, 1245, 1149, 1038.
HRMS (*m/z*): [M+H$^+$] calcd for $C_{15}H_{15}O_4$: 243.1015; found, 243.1020
TLC: R$_f$ = 0.7 (90:10 petroleum ether:EtOAc).

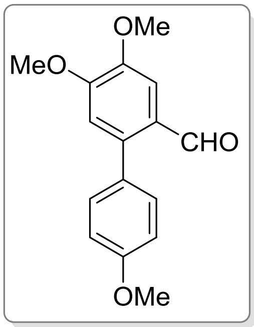

***4,4',5-trimethoxy-[1,1'-biphenyl]-2-carbaldehyde*:** Compound **1n** was prepared by general procedure B (4.0 mmol scale).
Eluent: petroleum ether/ethyl acetate (96/4, v/v).
Physical State: yellow oil.
Yield: 93% (643 mg isolated).
^{1}H NMR (400 MHz, $CDCl_3$) δ (ppm) 9.81 (s, 1H), 7.50 (s, 1H), 7.29 (d, *J* = 8.0 Hz, 2H), 6.97 (d, *J* = 8.0 Hz, 2H), 6.82 (s, 1H), 3.96 (s, 6H), 3.85 (s, 3H).
^{13}C NMR (101 MHz, $CDCl_3$) δ (ppm) 191.44, 159.73, 153.56, 148.68, 141.40, 131.48, 129.99, 127.08, 113.97, 112.76, 108.74, 56.34, 56.25, 55.54.
IR (thin film, cm^{-1}): 1042, 1135, 1177, 1213, 1247, 1277, 1349, 1396, 1440, 1463, 1501, 1596, 1672, 1735, 2850, 2928, 3002, 3076.
HRMS (*m/z*): [M+H$^+$] calcd for $C_{16}H_{17}O_4$: 273.1121; found, 273.1125.
TLC: R_f = 0.7 (90:10 petroleum ether:EtOAc).

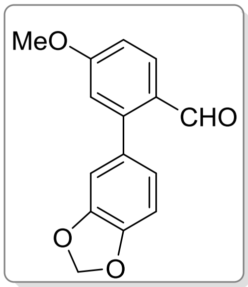

***2-(benzo[d][1,3]dioxol-5-yl)-4-methoxybenzaldehyde*:** Compound **1o** was prepared by general procedure B (4.0 mmol scale).
Eluent: petroleum ether/ethyl acetate (95/5, v/v).
Physical State: colorless oil.
Yield: 98% (1004 mg isolated).
^{1}H NMR (400 MHz, $CDCl_3$) δ (ppm) 9.85 (s, 1H), 7.98 (d, *J* = 8.7 Hz, 1H), 6.96 (ddd, *J* = 8.7, 2.5, 0.6 Hz, 1H), 6.90 – 6.85 (m, 2H), 6.84 (d, *J* = 2.5 Hz, 1H), 6.80 (dd, *J* = 7.9, 1.8 Hz, 1H), 6.03 (s, 2H), 3.89 (s, 3H).
^{13}C NMR (101 MHz, $CDCl_3$) δ (ppm) 191.05, 163.50, 148.07, 147.78, 131.58, 130.04, 127.49, 123.87, 115.14, 113.81, 110.20, 108.17, 101.45, 55.62.
IR (thin film, cm^{-1}): 3072, 3008, 2900, 2844, 2760, 1678, 1593, 1483, 1448, 1397, 1338, 1298, 1232, 1178, 1099, 1036.
HRMS (*m/z*): [M+Na$^+$] calcd for $C_{15}H_{12}NaO_4$: 279.0627; found, 279.0629.

TLC: R_f = 0.7 (90:10 petroleum ether:EtOAc).

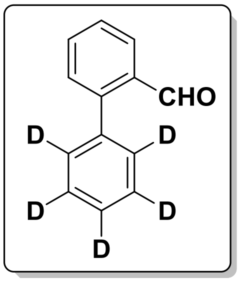

***[1,1'-biphenyl]-2',3',4',5',6'-d5-2-carbaldehyde*:** Compound **1b-d$_5$** was prepared by general procedure B using d_5-chlorobenzene (10 mmol scale).
Eluent: petroleum ether.
Physical State: colorless oil.
Yield: 91% (1.7 g isolated).
^{1}H NMR (500 MHz, $CDCl_3$) δ (ppm) 9.99 (d, *J* = 0.8 Hz, 1H), 8.04 (dd, *J* = 7.8, 1.5 Hz, 1H), 7.64 (td, *J* = 7.5, 1.5 Hz, 1H), 7.50 (tt, *J* = 7.5, 1.1 Hz, 1H), 7.46 (dd, *J* = 7.6, 1.3 Hz, 1H).
^{13}C NMR (126 MHz, $CDCl_3$) δ (ppm) 192.67, 146.14, 137.77, 133.92, 133.75, 130.97, 129.86 (t, *J* = 129.96 Hz), 128.11 (t, *J* = 129.96 Hz), 127.92, 127.76.
HRMS (*m/z*): [$M+H^+$] calcd for $C_{13}H_6D_5O$: 188.1118; found, 188.1124.
TLC: R_f = 0.7 (98:2 petroleum ether:EtOAc).

2.4 General procedure C: Synthesis of 2-phenylaniline derivatives

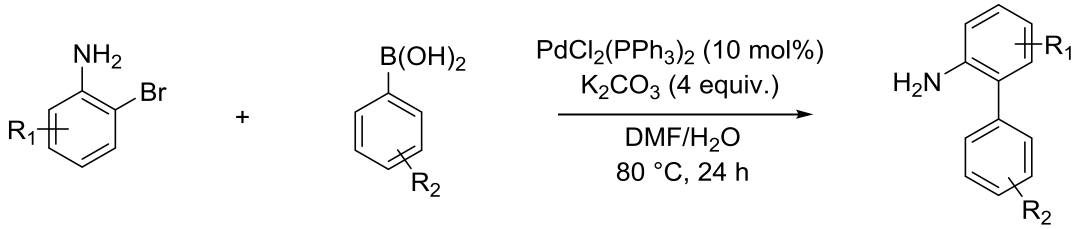

To a clean RB flask containing, arylboronic acid (4.35 mmol, 1.5 equiv.), K_2CO_3 (11.62 mmol, 4.0 equiv.) and $PdCl_2(PPh_3)_2$ (10 mol%) were added DMF/H_2O (13 mL/3 mL). To this resulting mixture 2-bromoanilines (2.9 mmol, 1.0 equiv.) was added and stirred at 80 °C for 24 h under nitrogen atmosphere. After completion of the reaction (monitor by TLC), water was added and extracted with ethyl acetate for 2-3 times. The combined organic layer was washed with saturated NaCl and dried over anhydrous Na_2SO_4. The organic layer was concentrated under vacuum, and the crude residue was purified by silica gel column chromatography (15%, petroleum ether/ethyl acetate) to afford the corresponding coupling product 2-aminobiaryls.

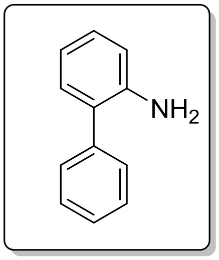

***[1,1'-biphenyl]-2-amine*:** Compound **2a** was prepared by general procedure C (2.9 mmol scale).
Eluent: petroleum ether/ethyl acetate (95/5, v/v).
Physical State: white solid.
Yield: 90% (447 mg isolated).
^{1}H NMR (500 MHz, $CDCl_3$) δ (ppm) 7.54 – 7.49 (m, 4H), 7.43 – 7.39 (m, 1H), 7.22 (ddd, *J* = 15.5, 7.8, 1.5 Hz, 2H), 6.90 (td, *J* = 7.5, 1.1 Hz, 1H), 6.83 (dd, *J* = 7.9, 0.8 Hz, 1H), 3.7 (s, 2H).
^{13}C NMR (126 MHz, $CDCl_3$) δ (ppm) 143.56, 139.59, 130.51, 129.15, 128.87, 128.56, 127.69, 127.22, 118.70, 115.66.
IR (thin film, cm^{-1}): 1027, 1082, 1173, 1244, 1307, 1416, 1459, 1493, 1592, 1719, 1784, 1859, 1933, 2097, 2332, 2542, 2640, 2925, 3043, 3394.
HRMS (*m/z*): [M+H$^+$] calcd for $C_{12}H_{12}N$: 170.0964; found, 170.0962.
TLC: R_f = 0.6 (90:10 petroleum ether:EtOAc).

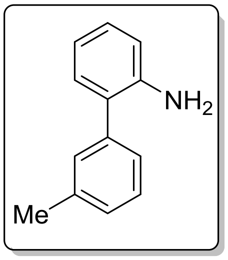

3'-methyl-[1,1'-biphenyl]-2-amine: Compound **2b** was prepared by general procedure C (2.9 mmol scale).
Eluent: petroleum ether/ethyl acetate (95/5, v/v).
Physical State: colorless oil.
Yield: 95% (505 mg isolated).
^{1}H NMR (400 MHz, $CDCl_3$) δ (ppm) 7.35 (t, *J* = 7.5 Hz, 1H), 7.27 (d, *J* = 7.0 Hz, 2H), 7.20 – 7.12 (m, 3H), 6.83 (td, *J* = 7.5, 1.1 Hz, 1H), 6.78 (dd, *J* = 7.9, 0.7 Hz, 1H), 3.75 (s, 2H), 2.41 (s, 3H).
^{13}C NMR (126 MHz, $CDCl_3$) δ (ppm). 143.51, 139.48, 138.48, 130.42, 129.83, 128.71, 128.41, 127.92, 127.81, 126.09, 118.62, 115.57, 21.50.
IR (thin film, cm^{-1}): 3468, 3378, 3021, 2922, 2856, 1792, 1692, 1614, 1478, 1448, 1295, 1250, 1215, 1157, 1094.
HRMS (ESI): [$M+H^+$] calcd for $C_{13}H_{14}N$: 184.1120; found, 184.1123.
TLC: R_f = 0.7 (90:10 petroleum ether:EtOAc).

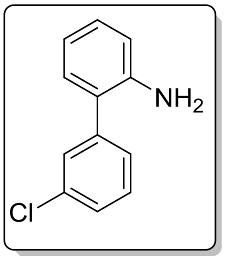

3'-chloro-[1,1'-biphenyl]-2-amine: Compound **2c** was prepared by general procedure C (2.9 mmol scale).
Eluent: petroleum ether/ethyl acetate (95/5, v/v).
Physical State: yellow oil.
Yield: 92% (543 mg isolated).
^{1}H NMR (400 MHz, $CDCl_3$) δ (ppm) 7.48 – 7.46 (m, 1H), 7.41 – 7.36 (m, 2H), 7.35 – 7.32 (m, 1H), 7.18 (td, *J* = 7.6, 1.5 Hz 1H), 7.11 (dd, *J* = 7.6, 1.5 Hz, 1H), 6.84 (td, *J* = 7.5, 1.1 Hz, 1H), 6.77 (dd, *J* = 8.0, 0.9 Hz, 1H), 3.71 (s, 2H).
^{13}C NMR (101 MHz, $CDCl_3$) δ (ppm) 143.42, 141.41, 134.65, 130.34, 130.07, 129.23, 129.01, 127.32, 127.29, 126.12, 118.75, 115.77.
IR (thin film, cm^{-1}): 3470, 3376, 3211, 3061, 3026, 2926, 1785, 1693, 1615, 1560, 1469, 1497, 1406, 1295, 1258, 1158, 1098, 1079, 1050, 1018.
HRMS (*m/z*): [$M+H^+$] calcd for $C_{12}H_{11}ClN$: 204.0574; found, 204.0576.
TLC: R_f = 0.6 (90:10 petroleum ether:EtOAc).

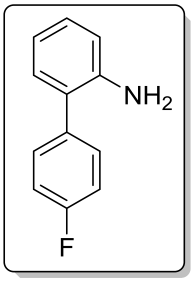

***4'-fluoro-[1,1'-biphenyl]-2-amine*:** Compound **2d** was prepared by general procedure C (2.9 mmol scale).
Eluent: petroleum ether/ethyl acetate (95/5, v/v).
Physical State: yellow oil.
Yield: 85% (461 mg isolated).
^{1}H NMR (400 MHz, $CDCl_3$) δ (ppm) 7.46 – 7.41 (m, 2H), 7.20 – 7.17 (m, 1H), 7.17 – 7.13 (m, 2H), 7.13 – 7.10 (m, 1H), 6.84 (td, *J* = 7.4, 1.1 Hz, 1H), 6.78 (dd, *J* = 8.0, 1.0 Hz, 1H), 3.72 (s, 2H).
^{13}C NMR (101 MHz, $CDCl_3$) δ (ppm) 162.06 (d, *J* = 246.1 Hz), 143.54, 135.39 (d, *J* = 3.3 Hz), 130.77 (d, *J* = 7.9 Hz), 130.49, 128.67, 126.62, 118.74, 115.84, 115.65 (d, *J* = 5.2 Hz), 77.39, 77.07, 76.75.
^{19}F NMR (471 MHz, $CDCl_3$) δ (ppm) -115.07.
IR (thin film, cm^{-1}): 3468, 3378, 3207, 2928, 2031, 1900, 1615, 1511, 1487, 1451, 1402, 1293, 1293, 1220, 1157, 1093, 1055, 1007.
HRMS (ESI): [M+H$^+$] calcd for $C_{12}H_{11}FN$: 188.0870; found, 188.0870.
TLC: R_f = 0.6 (90:10 petroleum ether:EtOAc).

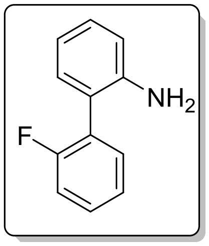

***2'-fluoro-[1,1'-biphenyl]-2-amine*:** Compound **2e** was prepared by general procedure D (2.9 mmol scale).
Eluent: petroleum ether/ethyl acetate (95/5, v/v).
Physical State: yellow oil.
Yield: 85% (461 mg isolated).
^{1}H NMR (500 MHz, $CDCl_3$) δ (ppm) 7.43 – 7.36 (m, 2H), 7.29 – 7.19 (m, 3H), 7.16 (d, *J* = 7.5 Hz, 1H), 6.88 (t, *J* = 7.5 Hz, 1H), 6.83 (d, *J* = 8.0 Hz, 1H), 3.64 (s, 2H).
^{13}C NMR (126 MHz, $CDCl_3$) δ (ppm) 143.63, 131.50 (d, *J* = 3.6 Hz), 130.53, 128.92 (d, *J* = 8.2 Hz), 128.69, 124.04, 121.04, 118.01, 115.64, 115.47, 115.25.
^{19}F NMR (471 MHz, $CDCl_3$) δ (ppm) **-**112.43.
IR (thin film, cm^{-1}): 3447, 3352, 3212, 2907, 2066, 1879, 1625, 1581, 1487, 1419, 1289, 1229, 1137, 1087, 946.
HRMS (*m/z*): [M+H$^+$] calcd for $C_{12}H_{11}FN$: 188.0870; found, 188.0875.

TLC: R_f = 0.7 (90:10 petroleum ether:EtOAc).

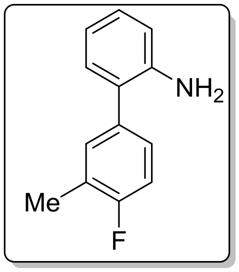

***4'-fluoro-3'-methyl-[1,1'-biphenyl]-2-amine*:** Compound **2f** was prepared by general procedure C (2.9 mmol scale).
Eluent: petroleum ether/ethyl acetate (97/3, v/v).
Physical State: yellow oil.
Yield: 87% (508 mg isolated).
^{1}H NMR (400 MHz, $CDCl_3$) δ (ppm) 7.28 – 7.21 (m, 2H), 7.15 (td, *J* = 7.7, 1.0 Hz, 1H), 7.11 – 7.04 (m, 2H), 6.81 (td, *J* = 5.1, 2.6 Hz, 1H), 6.76 (dd, *J* = 8.0, 0.9 Hz, 1H), 3.70 (s, 2H), 2.32 (d, *J* = 1.8 Hz, 3H).
^{13}C NMR (101 MHz, $CDCl_3$) δ (ppm) 160.62 (d, *J* = 245.0 Hz), 143.52, 132.21 (d, *J* = 5.2 Hz), 130.42, 128.50, 127.95 (d, *J* = 8.0 Hz), 126.86, 118.65, 115.29 (d, *J* = 22.3 Hz), 115.18, 14.6.
^{19}F NMR (471 MHz, $CDCl_3$) δ (ppm) **-**112.43.
IR (thin film, cm^{-1}): 3465, 3377, 3208, 3027, 2925, 1898, 1783, 1614, 1486, 1451, 1395, 1298, 1225, 1169, 1118, 1054, 1050, 988, 936.
HRMS (ESI): [$M+H^+$] calcd for $C_{13}H_{13}FN$: 202.1027; found, 202.1026.
TLC: R_f = 0.7 (90:10 petroleum ether:EtOAc).

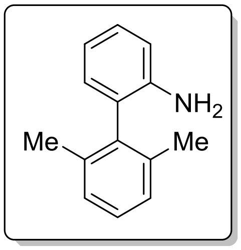

***2',6'-dimethyl-[1,1'-biphenyl]-2-amine*:** Compound **2g** was prepared by general procedure C (2.9 mmol scale).
Eluent: petroleum ether/ethyl acetate (95/5, v/v).
Physical State: greenish oil.
Yield: 96% (549 mg isolated).
^{1}H NMR (400 MHz, $CDCl_3$) δ (ppm) 7.22 – 7.16 (m, 2H), 7.16 – 7.13 (m, 2H), 6.93 (dd, *J* = 7.5, 1.6 Hz, 1H), 6.83 (td, *J* = 7.4, 1.1 Hz, 1H), 6.79 (dd, *J* = 8.0, 1.1 Hz, 1H), 3.39 (s, 2H), 2.06 (s, 6H).
^{13}C NMR (126 MHz, $CDCl_3$) δ (ppm) 143.55, 138.09, 137.36, 129.85, 128.34, 127.82, 127.69, 126.27, 118.68, 115.23, 20.39.
IR (thin film, cm^{-1}): 3468, 3378, 3061, 3019, 2855, 2736, 2609, 1930, 1785, 1612, 1449, 1496, 1377, 1100, 1041, 1002.

HRMS (*m/z*): [M+H$^+$] calcd for $C_{14}H_{16}N$: 198.1277; found, 198.1279.
TLC: R_f = 0.7 (90:10 petroleum ether:EtOAc).

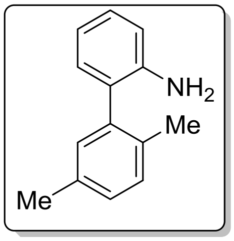

***2',5'-dimethyl-[1,1'-biphenyl]-2-amine*:** Compound **2h** was prepared by general procedure C (2.9 mmol scale).
Eluent: petroleum ether/ethyl acetate (95/5, v/v).
Physical State: brown oil.
Yield: 94% (538 mg isolated).
^{1}H NMR (500 MHz, CDCl$_3$) δ (ppm) 7.27 – 7.21 (m, 2H), 7.16 (d, *J* = 7.8 Hz, 1H), 7.13 – 7.06 (m, 2H), 6.88 (t, *J* = 7.4, 0.7 Hz, 1H), 6.82 (d, *J* = 8.0 Hz, 1H), 3.55 (s, 2H), 2.41 (s, 3H), 2.21 (s, 3H).
^{13}C NMR (126 MHz, CDCl$_3$) δ (ppm) 143.70, 138.52, 135.63, 133.77, 130.77, 130.24, 130.13, 128.46, 128.31, 127.69, 118.25, 115.07, 20.97, 19.23.
IR (thin film, cm^{-1}): 3442, 3312, 3092, 3001, 2863, 2749, 2621, 1910, 1775, 1633, 1458, 1401, 1377, 1138, 1045, 997.
HRMS (*m/z*): [M+H$^+$] calcd for $C_{14}H_{16}N$: 198.1277; found, 198.1278.
TLC: R_f = 0.8 (90:10 petroleum ether:EtOAc).

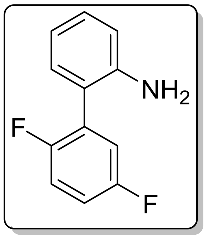

***2',5'-difluoro-[1,1'-biphenyl]-2-amine*:** Compound **2i** was prepared by general procedure C (2.9 mmol scale).
Eluent: petroleum ether/ethyl acetate (96/4, v/v).
Physical State: brown oil.
Yield: 85% (505 mg isolated).
^{1}H NMR (400 MHz, CDCl$_3$) δ (ppm) 7.22 (td, *J* = 7.5, 2.1, 1H), 7.17 – 7.01 (m, 4H), 6.84 (td, *J* = 7.5, 1.1 Hz, 1H), 6.80 (dd, *J* = 7.8, 1.1 Hz, 1H 1H), 3.69 (s, 2H).
^{13}C NMR (101 MHz, CDCl$_3$) δ (ppm) 160.05 (dd, *J* = 243.3, 2.4 Hz), 144.00, 130.87 (d, *J* = 0.8 Hz), 129.62, 120.41, 118.60, 118.20 (dd, *J* = 23.6, 4.0 Hz), 117.65 (ddd, *J* = 34.4, 24.5, 6.4 Hz), 117.25, 116.87, 115.93, 115.55.
^{19}F NMR (471 MHz, CDCl$_3$) δ (ppm) -118.59, -120.41.
IR (thin film, cm^{-1}): 3445, 3397, 3228, 3072, 2952, 1828, 1763, 1604, 1446, 1459, 1351, 1277, 1295, 1169, 1198, 1044, 916.

HRMS (ESI): [M+H$^+$] calcd for $C_{12}H_{10}F_2N$: 206.0776; found, 206.0773.
TLC: R$_f$ = 0.6 (90:10 petroleum ether:EtOAc).

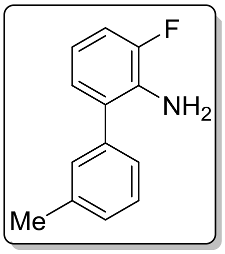

***3-fluoro-3'-methyl-[1,1'-biphenyl]-2-amine*:** Compound **2j** was prepared by general procedure C (2.9 mmol scale).
Eluent: petroleum ether/ethyl acetate (95/5, v/v).
Physical State: yellow oil.
Yield: 75% (437 mg isolated).
^{1}H NMR (500 MHz, CDCl$_3$) δ (ppm) 7.39 (t, *J* = 7.5 Hz, 1H), 7.33 – 7.28 (m, 2H), 7.23 (d, *J* = 7.5 Hz, 1H), 7.06 – 7.00 (m, 1H), 6.96 (dd, *J* = 7.9, 5.3 Hz, 1H), 6.80 – 6.74 (m, 1H), 3.87 (s, 2H), 2.45 (s, 3H).
^{13}C NMR (126 MHz, CDCl$_3$) δ (ppm) 151.78 (d, *J* = 238.3 Hz), 138.65, 138.31 (d, *J* = 3.0 Hz), 132.20 (d, *J* = 12.5 Hz), 129.71, 129.70 (d, *J* = 3.3 Hz), 128.83, 128.32, 125.92, 125.51 (d, *J* = 2.9 Hz), 117.65 (d, *J* = 7.8 Hz), 113.96 (d, *J* = 19.0 Hz), 21.50.
^{19}F NMR (471 MHz, CDCl$_3$) δ (ppm) -134.33.
IR (thin film, cm^{-1}): 3480, 3389, 3037, 3198, 2921, 1946, 1892, 1800, 1625, 1601, 1573, 1476, 1411, 1327, 1269, 1210, 1139, 1095, 1067.
HRMS (ESI): [M+H$^+$] calcd for $C_{13}H_{13}FN$: 202.1027; found, 202.1025.
TLC: R$_f$ = 0.7 (90:10 petroleum ether:EtOAc).

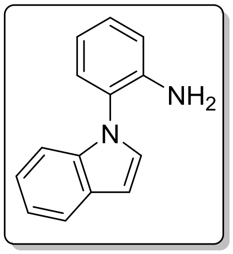

***2-(1H-indol-1-yl)aniline*:** Compound **2k** was prepared by modified literature procedure (3.0 mmol scale).[1]
Eluent: petroleum ether/ethyl acetate (90/10, v/v).
Physical State: yellow oil.
Yield: 76% (713 mg isolated).
^{1}H NMR (400 MHz, CDCl$_3$) δ (ppm) 7.75 – 7.72 (m, 1H), 7.31 – 7.27 (m, 1H), 7.24 (q, *J* = 2.5 Hz, 2H), 7.21 (d, *J* = 1.8 Hz, 2H), 7.20 (s, 1H), 6.91 – 6.85 (m, 2H), 6.73 (dd, *J* = 3.2, 0.5 Hz, 1H), 3.59 (s, 2H).
^{13}C NMR (101 MHz, CDCl$_3$) δ (ppm) 143.18, 136.41, 129.21, 128.67, 128.65, 128.60, 124.90, 122.26, 121.00, 120.20, 118.57, 116.29, 110.79, 103.25.

IR (thin film, cm^{-1}): 1010, 1063, 1136, 1229, 1260, 1311, 1331, 1453, 1512, 1618, 1699, 1781, 2918, 3052, 3204, 3372, 3468.
HRMS (*m/z*): [M+H$^+$] calcd for $C_{14}H_{13}N_2$: 209.1073; found, 209.1070.
TLC: R_f = 0.7 (80:20 petroleum ether:EtOAc).

2.5 Synthesis of acrylate from bioactive alcohols:

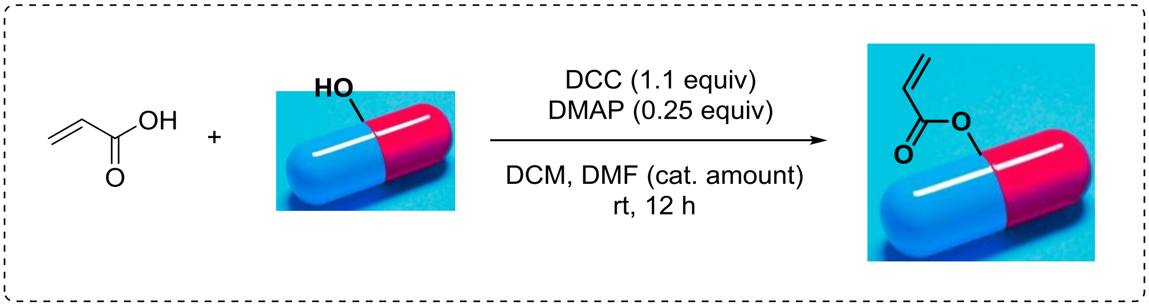

An oven-dried 20 mL reaction tube was charged with a magnetic stir-bar, acrylic acid (2 mmol), alcohol (2.2 mmol), DCC (2.2 mmol) and DMAP (0.5 mmol). Then 8 mL of dry DCM and two drops of DMF were added. The reaction mixture was stirred vigorously at room temperature along. The reaction was carried out for overnight and the reaction mixture was diluted with 10 mL DCM and filtered through a cotton pad to filter off solid part. Next, the solvent was dried completely and in semi-solid mixture, ethyl acetate (10 mL) and brine solution (10 mL) were added and ethyl acetate part was collected. Aqueous part was washed three additional times with ethyl acetate (10 mL). The desired ester was isolated by column chromatography using silica gel (100-200 mesh size) and petroleum ether/ethyl acetate as the eluent.

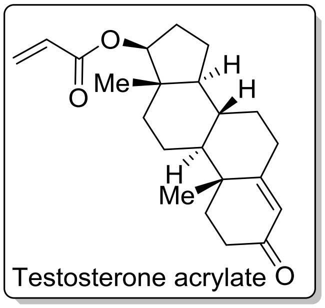

***(8R,9S,10R,13S,14S,17S)-10,13-dimethyl-3-oxo-2,3,6,7,8,9,10,11,12,13,14,15,16,17-tetradecahydro-1H-cyclopenta[a]phenanthren-17-yl acrylate*:** The compound was prepared by above depicted procedure (2 mmol scale).
Eluent: petroleum ether/ethyl acetate (80/20, v/v) [a little amount of DCC-acrylate intermediate has been observed in the main compound which was almost inseparable. The desired product was collected by several times pentane wash].
Physical State: colorless solid.
Yield: 62% (424 mg isolated).
^{1}H NMR (400 MHz, $CDCl_3$) δ (ppm) 6.36 (ddd, *J* = 17.4, 1.6, 0.6 Hz, 1H), 6.10 (ddd, *J* = 17.3, 10.4, 0.6 Hz, 1H), 5.78 (ddd, *J* = 10.4, 1.6, 0.6 Hz, 1H), 5.71 (d, *J* = 0.8 Hz, 1H), 4.66 (dd, *J* = 9.2,

7.8 Hz, 1H), 2.51 – 2.24 (m, 4H), 2.19 (dddd, *J* = 15.7, 9.4, 7.1, 3.2 Hz, 1H), 2.00 (ddd, *J* = 13.4, 5.0, 3.2 Hz, 1H), 1.91 – 1.84 (m, 1H), 1.71 (dd, *J* = 8.7, 5.2 Hz, 1H), 1.66 – 1.61 (m, 1H), 1.54 – 1.48 (m, 1H), 1.46 – 1.40 (m, 1H), 1.36 – 1.32 (m, 1H), 1.29 – 1.18 (m, 3H), 1.17 (s, 3H), 1.16 – 0.87 (m, 4H), 0.85 (s, 3H).
^{13}C NMR (101 MHz, CDCl$_3$) δ (ppm) 199.55, 171.06, 166.35, 130.43, 128.98, 124.12, 82.71, 53.87, 50.46, 42.86, 38.78, 36.81, 35.87, 35.59, 34.09, 32.90, 31.67, 31.00, 27.69, 26.44, 20.71, 17.57, 12.23.
HRMS (*m/z*): [M+H$^+$] calcd for $C_{22}H_{31}O_3$: 343.2268; found, 343.2272.
TLC: R$_f$ = 0.6 (70:30 petroleum ether:EtOAc).

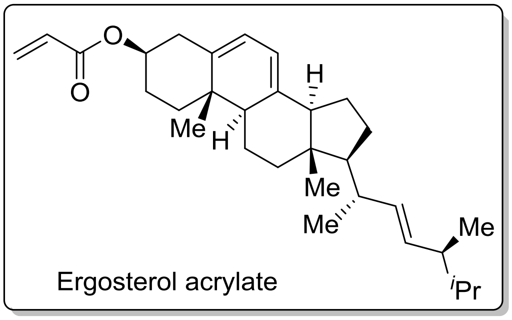

***(3R,9R,10S,13S,14S,17S)-17-((2S,5S,E)-5,6-dimethylhept-3-en-2-yl)-10,13-dimethyl-2,3,4,9,10,11,12,13,14,15,16,17-dodecahydro-1H-cyclopenta[a]phenanthren-3-yl acrylate*:** The compound was prepared by above depicted procedure (2 mmol scale).
Eluent: petroleum ether/ethyl acetate (98/2, v/v).
Physical State: colorless oil.
Yield: 74% (666.5 mg isolated).
^{1}H NMR (500 MHz, CDCl$_3$) δ (ppm) 6.39 (dd, *J* = 17.3, 1.5 Hz, 1H), 6.11 (dd, *J* = 17.3, 10.4 Hz, 1H), 5.81 (dd, *J* = 10.4, 1.5 Hz, 1H), 5.58 (dd, *J* = 5.8, 2.5 Hz, 1H), 5.38 (dt, *J* = 5.6, 2.8 Hz, 1H), 5.28 – 5.09 (m, 2H), 4.79 (tt, *J* = 11.5, 4.6 Hz, 1H), 2.54 (ddd, *J* = 14.5, 5.0, 2.4 Hz, 1H), 2.41 (ddd, *J* = 14.2, 11.8, 2.3 Hz, 1H), 2.09 – 1.96 (m, 4H), 1.94 – 1.83 (m, 4H), 1.78 – 1.66 (m, 3H), 1.60 (ddd, *J* = 14.7, 9.6, 3.2 Hz, 2H), 1.47 (h, *J* = 6.6 Hz, 1H), 1.42 – 1.31 (m, 3H), 1.27 (ddd, *J* = 17.6, 10.5, 6.4 Hz, 2H), 1.03 (d, *J* = 6.6 Hz, 3H), 0.96 (s, 3H), 0.91 (d, *J* = 6.8 Hz, 3H), 0.83 (t, *J* = 7.2 Hz, 6H), 0.63 (s, 3H).
^{13}C NMR (126 MHz, CDCl$_3$) δ (ppm) 165.92, 141.79, 138.70, 135.77, 132.18, 130.63, 129.17, 120.46, 116.50, 73.18, 55.90, 54.73, 46.24, 43.02, 40.66, 39.23, 38.12, 37.31, 36.84, 33.30, 28.50, 28.31, 23.20, 21.32, 21.23, 20.17, 19.86, 17.82, 16.40, 12.27.
HRMS (*m/z*): [M+H$^+$] calcd for $C_{31}H_{47}O_2$: 451.3571; found, 451.3576.
TLC: R$_f$ = 0.6 (95:5 petroleum ether:EtOAc).

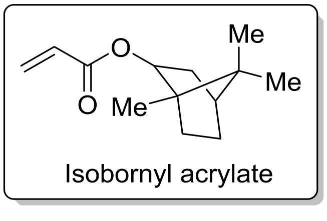

Isobornyl acrylate was purchased from Sigma Aldrich (Product Number: 392103, CAS: 5888-33-5)

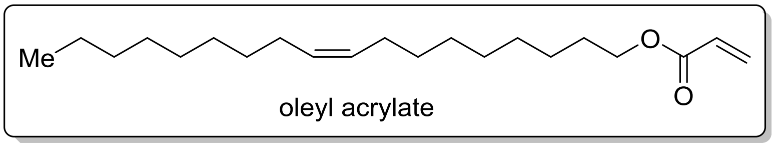

***(Z)-octadec-9-en-1-yl acrylate*:** The compound was prepared by above depicted procedure (2 mmol scale).
Eluent: petroleum ether/ethyl acetate (97/3, v/v).
Physical State: colorless oil.
Yield: 76% (490 mg isolated).
^{1}H NMR (500 MHz, $CDCl_3$) δ (ppm) 6.39 (d, *J* = 17.3 Hz, 1H), 6.12 (dd, *J* = 17.3, 10.4 Hz, 1H), 5.81 (d, *J* = 10.4 Hz, 1H), 5.41 – 5.30 (m, 2H), 4.14 (t, *J* = 6.8 Hz, 2H), 2.01 (q, *J* = 6.4 Hz, 4H), 1.66 (p, *J* = 6.9 Hz, 2H), 1.29 (dd, *J* = 21.6, 10.5 Hz, 22H), 0.88 (t, *J* = 6.8 Hz, 3H).
^{13}C NMR (126 MHz, $CDCl_3$) δ (ppm) 166.62, 130.69, 130.23, 130.04, 128.88, 64.97, 32.15, 30.01, 29.97, 29.77, 29.65, 29.57, 29.47, 29.44, 28.85, 27.46, 27.43, 26.16, 22.93, 14.36.
HRMS (*m/z*): $[M+H^+]$ calcd for $C_{21}H_{39}O_2$: 323.2945; found, 323.2949.
TLC: R_f = 0.3 (95:5 petroleum ether:EtOAc).

2.6 Temporary Directing Group (TDG) variation for *meta*-C–H olefination

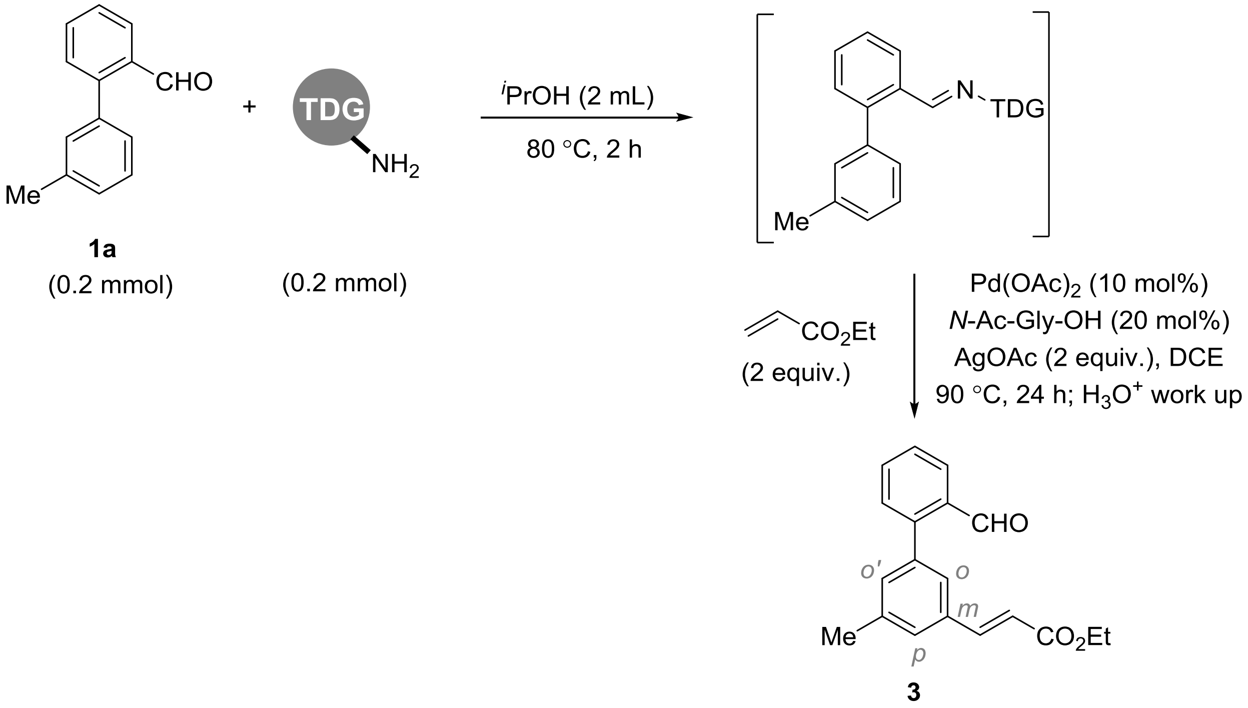

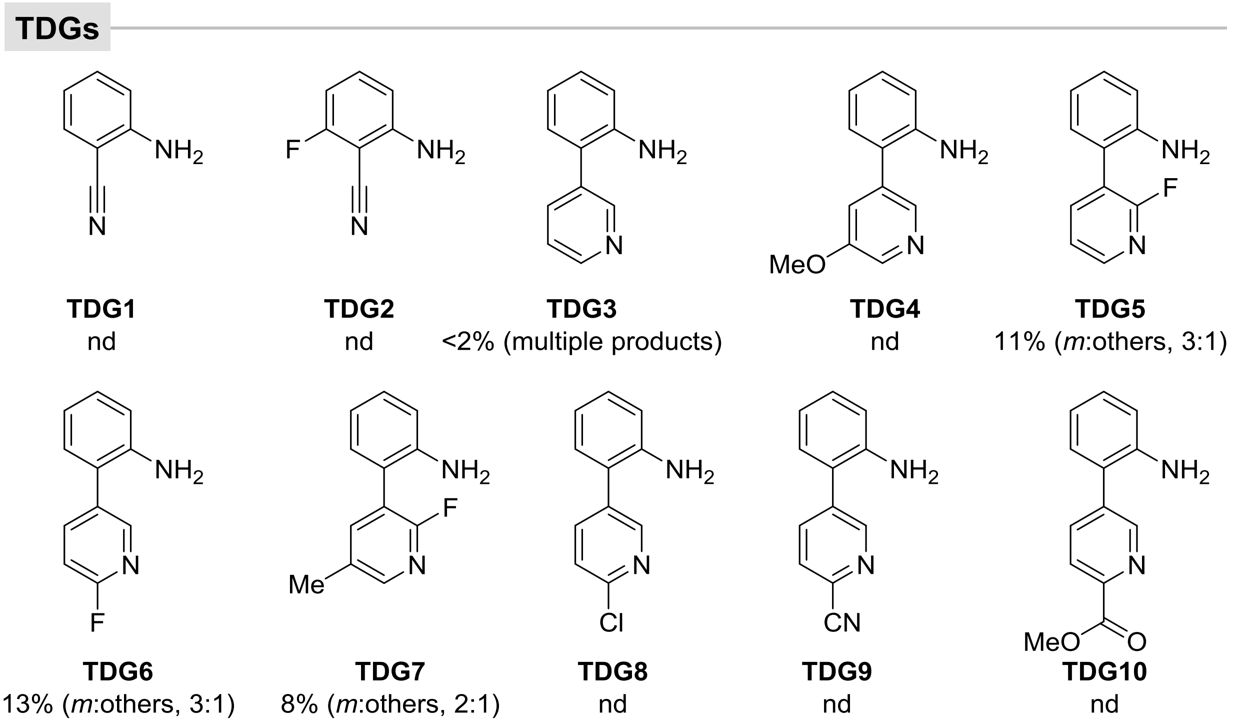

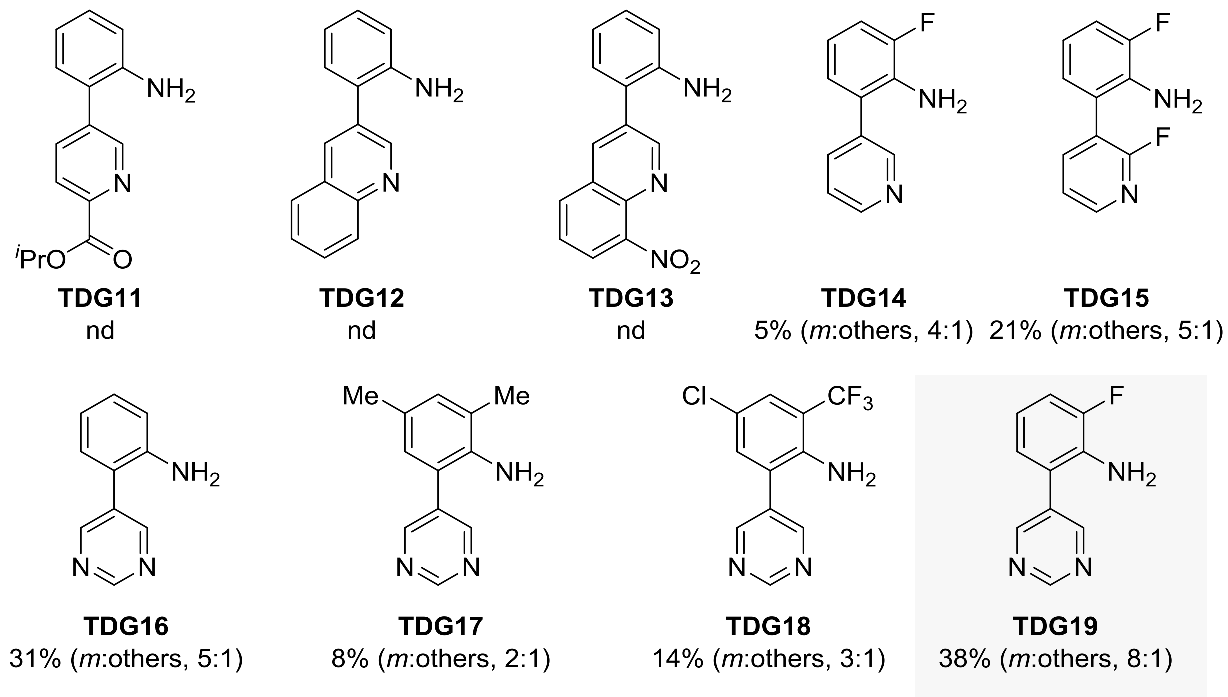

TDG11 nd; **TDG12** nd; **TDG13** nd; **TDG14** 5% (*m*:others, 4:1); **TDG15** 21% (*m*:others, 5:1)

TDG16 31% (*m*:others, 5:1); **TDG17** 8% (*m*:others, 2:1); **TDG18** 14% (*m*:others, 3:1); **TDG19** 38% (*m*:others, 8:1)

Reagents and reaction conditions: Step1: 1a (0.2 mmol), **TDG** (0.2 mmol) in ^{i}PrOH (2 mL) at 80 °C for 2h; concentrated under vacuum, the crude mixture was used for next step; **Step2:** $Pd(OAc)_2$ (10 mol%, 0.02 mmol), *N*-Ac-Gly-OH (20 mol%, 0.04 mmol), AgOAc (2 equiv., 0.4 mmol), ethyl acrylate (2 equiv., 0.4 mmol), DCE (2 mL) at 90 °C for 24 h.

Yield and selectivity are based on ^{1}H NMR of the crude reaction mixture using 1,3,5-trimethoxybenzene (TMB) as internal standard. Doublet of olefin proton in ^{1}H NMR was used to measure the selectivity. Ratios of *meta*:others are shown in parenthesis. nd, not detected.

2.7 Optimization details for *meta*-C–H olefination of 2-phenylbenzyldehyde

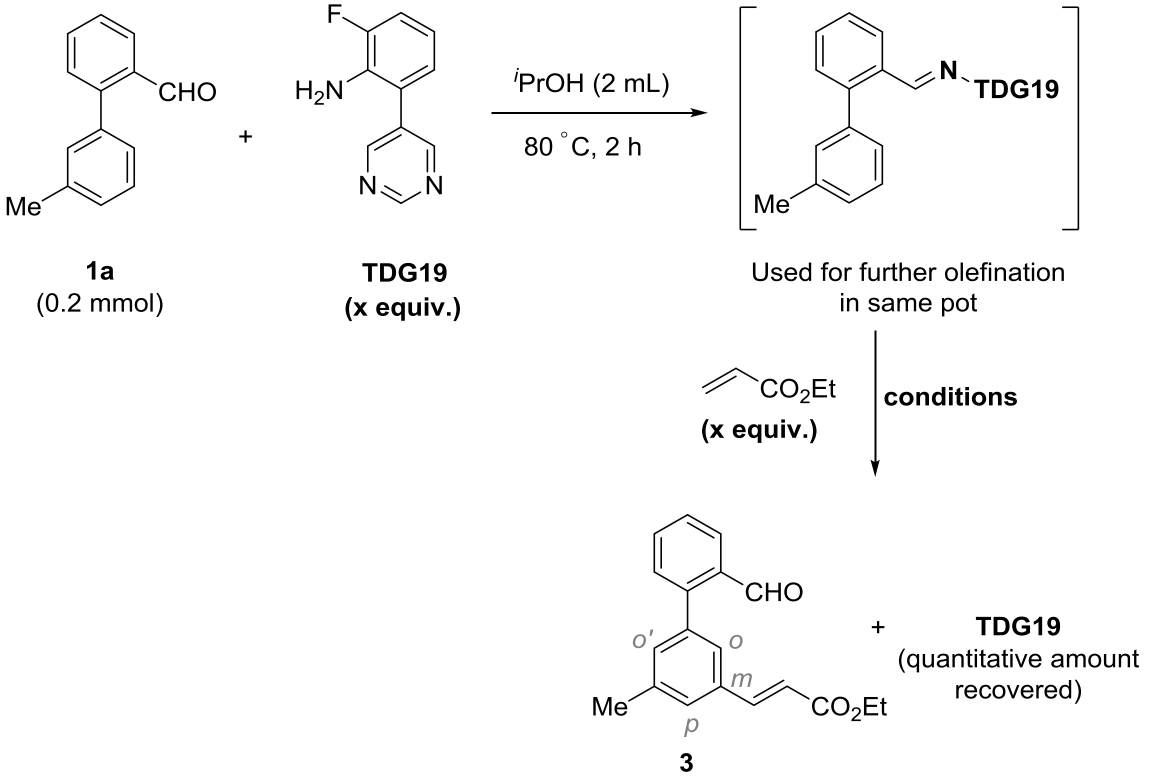

Supplementary Table 1: Optimization of imine intermediate: Different conditions

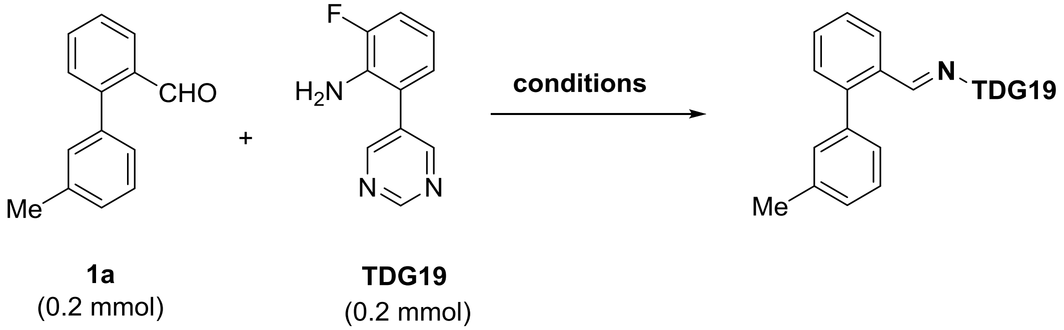

Entry	Conditions	Yield[a]
1	MeCN, 80 °C	65%
2	DCM, rt	62%
3	DCM + 4 Å MS, rt	64%
4	Toluene, rt	40%
5	Toluene, 80 °C	45%
6	^{i}PrOH + cat. CH_3COOH, 70 °C	48%
7	MeOH + cat. *p*-TSA, 70 °C	nd
**8**	**^{i}PrOH, 80 °C, 2 h; dried, then decant with PE/diethyl ether**	**95%**

[a]Yield was measured by ^{1}H NMR of the crude reaction mixture using 1,3,5-trimethoxybenzene.

Supplementary Table 2: Optimization of TDG19 amount

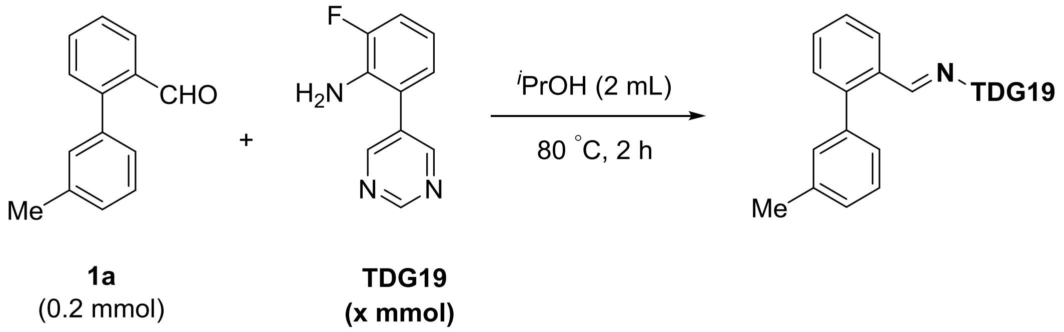

Entry	1a (mmol)	TDG19 (mmol)	Yield (%)
1	0.2	0.18	89
**2**	**0.2**	**0.19**	**95**[a]
3	0.2	0.2	95[b]
4	0.2	0.21	96[b]
5	0.2	0.22	96[b]

[a]**TDG19** was fully consumed. [b]Some amount of **TDG19** was left. *Note:* As free amine was detrimental for this reaction, we have selected **entry 2** for further use.

Optimization for *meta*-C–H olefination of 2-phenylbenzyldehyde:

Supplementary Table 3: Solvent optimization

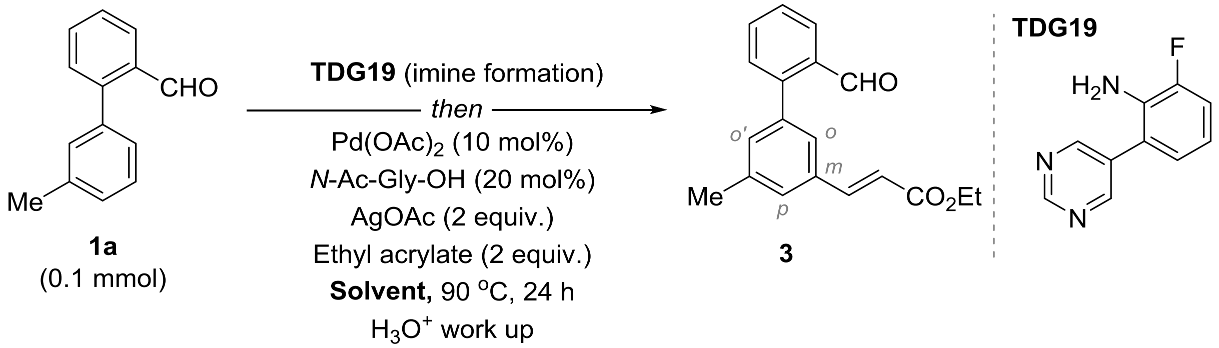

Entry	Solvent (1 mL)	Yield (*m*:others)[a]
1	HFIP	20% (5:1)
**2**	**DCE**	**38% (8:1)**
3	DCE (1 mL):HFIP (10 µL)	15% (5:1)
4	DCE (1 mL):HFIP (20 µL)	15% (5:1)
5	DCE (1 mL):HFIP (30 µL)	11% (5:1)
6	DCE (1 mL):HFIP (40 µL)	15% (5:1)
7	DCE (1 mL):HFIP (50 µL)	17% (5:1)

8	DCE (1 mL):HFIP (75 µL)	13% (5:1)
9	DCE (0.9 mL):HFIP (100 µL)	9% (4:1)
10	DCE (0.8 mL):HFIP (200 µL)	12% (5:1)
11	DCE (0.7 mL):HFIP (300 µL)	8% (4:1)
12	TFE	18% (3:1)
13	MeCN	11% (3:1)
14	1,4-Dioxane	5% (2:1)
15	TFT	3% (1:1)
16	^{i}PrOH	nd
17	DCM	8% (3:1)
18	THF	2% (1:1)
19	DMF	nd
20	$CHCl_3$	15% (4:1)
21	1,2-dibromoethane	13% (2:1)
22	2-Ethoxy ethanol	nd
23	EtOAc	6% (4:1)
24	1,2,3-TCP	20% (5:1)
25	Acetone	nd
26	Isoamyl alcohol	nd
27	CCl_4	nd
28	Cyclohexane	nd

[a]Yield and selectivity are based on ^{1}H NMR of the crude reaction mixture using 1,3,5-trimethoxybenzene (TMB) as internal standard. Doublet of olefin proton in ^{1}H NMR was used to measure the selectivity. Ratios of *meta*:others are shown in parenthesis. nd, not detected.

Supplementary Table 4: Catalyst optimization

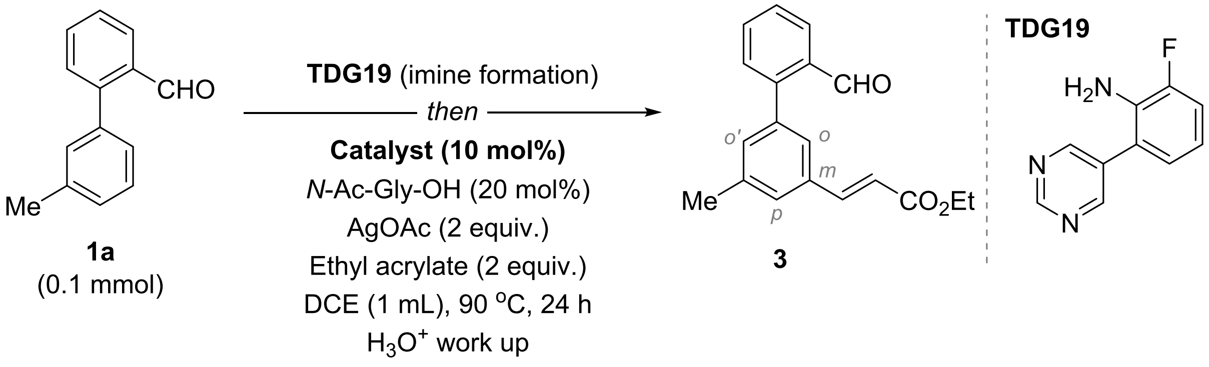

Entry	Catalyst	Yield (*m*:others)[a]
1	**$Pd(OAc)_2$**	**38% (8:1)**
2	$Pd(acac)_2$	18% (6:1)

3	$Pd(TFA)_2$	19% (3:1)
4	$Pd(OPiv)_2$	15% (5:1)
5	$Pd(CH_3CN)_2Cl_2$	8% (3:1)
6	$Pd(PPh_3)_4$	nd
7	$Pd_2(dba)_3$	nd
8	$PdCl_2(PPh_3)_2$	nd
9	$Pd(PhCN)_2Cl_2$	trace
10	$Pd(COD)Cl_2$	nd

[a]Yield and selectivity are based on ^1H NMR of the crude reaction mixture using 1,3,5-trimethoxybenzene (TMB) as internal standard. Doublet of olefin proton in ^1H NMR was used to measure the selectivity. Ratios of *meta*:others are shown in parenthesis. nd, not detected.

Supplementary Table 5: Ligand optimization

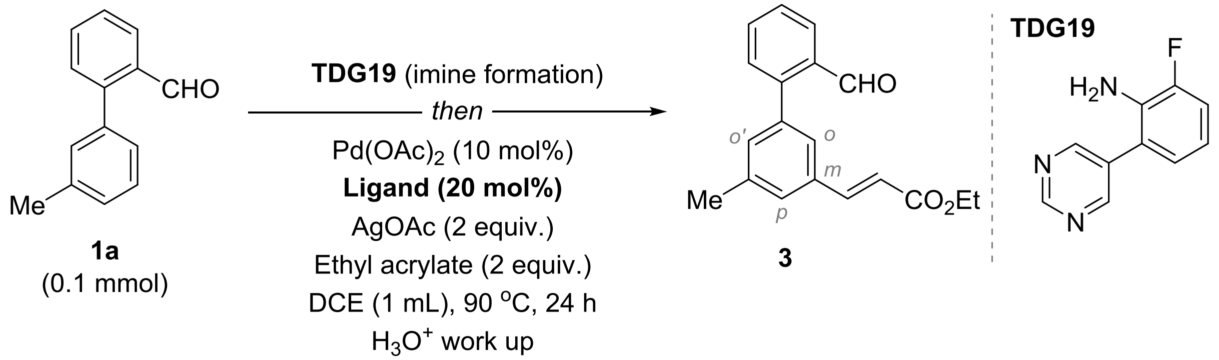

Entry	Ligand	Yield (*m*:others)[a]
1	*N*-Ac-Gly-OH	38% (8:1)
**2**	***N*-Form-Gly-OH**	**44% (12:1)**
3	2-Hydroxy-5-nitro pyridine	5% (2:1)
4	*N*-Ac-Nle-OH	18% (7:1)
5	*N*-Ac-Leu-OH	21% (8:1)
6	*N*-Ac-Val-OH	22% (5:1)
7	*N*-Ac-Ala-OH	18% (9:1)
8	*N*-Ac-Phg-OH	3% (2:1)
9	*N*-Ac-Trp-OH	2% (2:1)
10	Carbobenzoxy-Val-OH	nd
11	*N*-Boc-L-Alanine	10% (5:1)
12	*N*-Fmoc-L-Leucine	1%
13	*N*-Ac-L-Glutamic acid	3%
14	*N*-Boc-L-Phenylalanine	9% (4:1)
15	*N*-Ac-L-Histidine	1%
16	*N*-Boc-L-*tert*-Leucine	nd
17	*N*-Benzoyl-Leucine	2% (1:1)
18	*N*-Boc-L-Isoleucine	8% (2:1)
19	*N*-Benzoyl-Valine	2% (2:1)

20	*N*-Benzoyl-Phenylalanine	nd
21	2-hydroxypyridine	trace
22	2-Hydroxy-3-nitro pyridine	5% (2:1)
23	2-Hydroxy-6-methyl pyridine	nd
24	5-Chloro-2-hydroxy pyridine	trace
25	5-Chloro-2-hydroxy-3-nitro pyridine	nd
26	2-Hydroxy-4-methyl-5-nitro pyridine	trace
27	3-Methyl-2-hydroxy-5-nitro pyridine	trace
28	2-hydroxy-5-methyl-3-nitro pyridine	trace
29	2-hydroxy-5-trifluromethyl pyridine	16% (4:1)
30	2-hydroxy-4-methoxy-3-nitrile pyridine	8% (3:1)

[a]Yield and selectivity are based on ^{1}H NMR of the crude reaction mixture using 1,3,5-trimethoxybenzene (TMB) as internal standard. Doublet of olefin proton in ^{1}H NMR was used to measure the selectivity. Ratios of *meta*:others are shown in parenthesis. nd, not detected.

Supplementary Table 6: Oxidant optimization

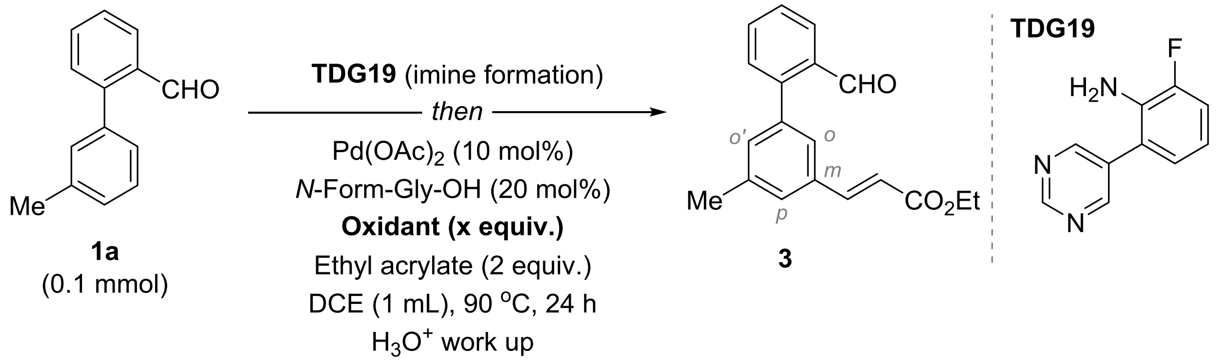

Entry	Oxidant (2 equiv.)	Yield (*m*:others)[a]
1	AgTFA	12% (1:1)
2	Ag_2CO_3	48% (12:1)
3	AgOAc	44% (12:1)
4	CuOTf	nd
5	Ag_2SO_4	22% (6:1)
7	$AgNO_3$	nd
8	$Cu(OAc)_2$	15% (6:1)
12	$Cu_2Cr_2O_5$	16% (2:1)
15	$CuCl_2$	nd
21	Ag_2CO_3:$Cu(OAc)_2$ (1:1)	43% (8:1)
22	Ag_2CO_3:$Cu(OAc)_2$ (1:2)	49% (8:1)
**23**	**Ag_2CO_3:$Cu(OAc)_2$ (1:3)**	**62% (12:1)**
24	Ag_2CO_3:$Cu(OAc)_2$ (2:1)	42% (7:1)
25	Ag_2CO_3:$Cu(OAc)_2$ (2:2)	44% (8:1)
26	Ag_2CO_3:$Cu(OAc)_2$ (2:3)	51% (10:1)

27	Ag_2CO_3:$Cu(OAc)_2$ (3:1)	46% (10:1)
28	Ag_2CO_3:$Cu(OAc)_2$ (3:2)	44% (5:1)
29	Ag_2CO_3:$Cu(OAc)_2$ (3:3)	38% (5:1)

[a]Yield and selectivity are based on ^{1}H NMR of the crude reaction mixture using 1,3,5-trimethoxybenzene (TMB) as internal standard. Doublet of olefin proton in ^{1}H NMR was used to measure the selectivity. Ratios of *meta*:others are shown in parenthesis. nd, not detected.

Supplementary Table 7: Optimization of Ag_2CO_3 loading

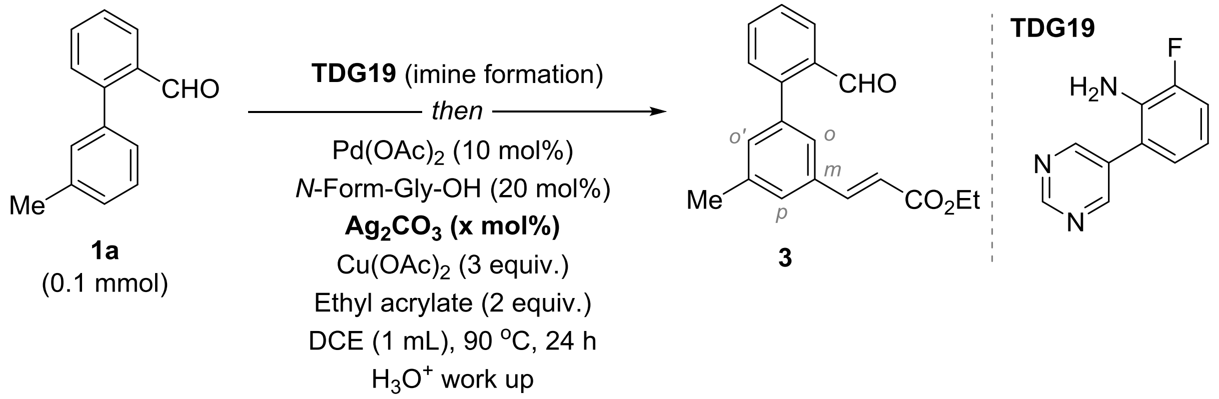

Entry	Ag_2CO_3 (x mol%)	Yield (*m*:others)[a]
1	Without Ag_2CO_3	15% (6:1)
2	7 mol%	22% (10:1)
3	15 mol%	32% (13:1)
4	20 mol%	55% (14:1)
5	**25 mol%**	**65% (15:1)**
6	50 mol%	63% (15:1)
7	75 mol%	58% (14:1)
8	100 mol%	62% (12:1)

[a]Yield and selectivity are based on ^{1}H NMR of the crude reaction mixture using 1,3,5-trimethoxybenzene (TMB) as internal standard. Doublet of olefin proton in ^{1}H NMR was used to measure the selectivity. Ratios of *meta*:others are shown in parenthesis. nd, not detected.

Supplementary Table 8: Optimization of $Pd(OAc)_2$ loading

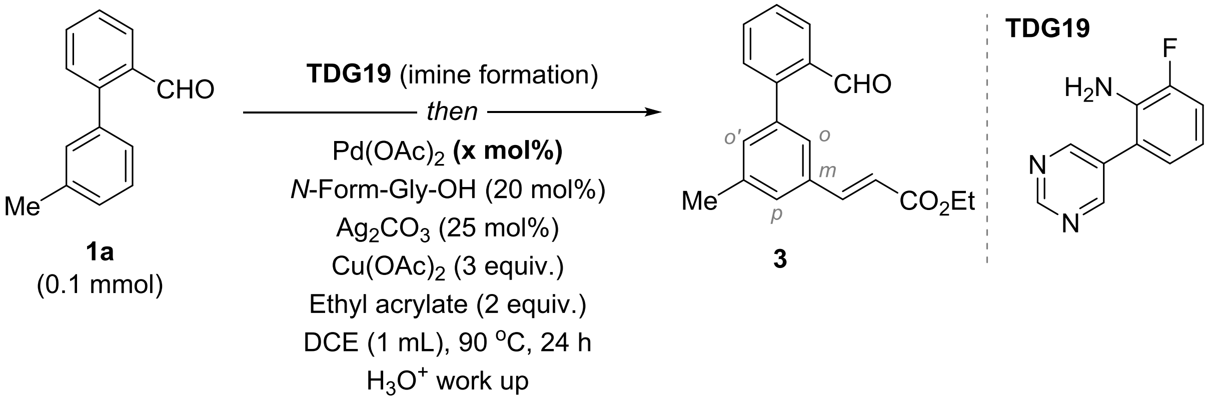

Entry	$Pd(OAc)_2$	Yield (*m*:others)[a]
1	1 mol%	5% (5:1)
2	2 mol%	8% (6:1)
3	3 mol%	16% (5:1)
4	4 mol%	19% (11:1)
5	5 mol%	26% (14:1)
6	6 mol%	38% (13:1)
7	7 mol%	53% (12:1)
8	8 mol%	62% (15:1)
9	9 mol%	63% (15:1)
**10**	**10 mol%**	**65% (15:1)**

[a]Yield and selectivity are based on ^1H NMR of the crude reaction mixture using 1,3,5-trimethoxybenzene (TMB) as internal standard. Doublet of olefin proton in ^1H NMR was used to measure the selectivity. Ratios of *meta*:others are shown in parenthesis. nd, not detected.

Supplementary Table 9: Optimization of $Cu(OAc)_2$ loading

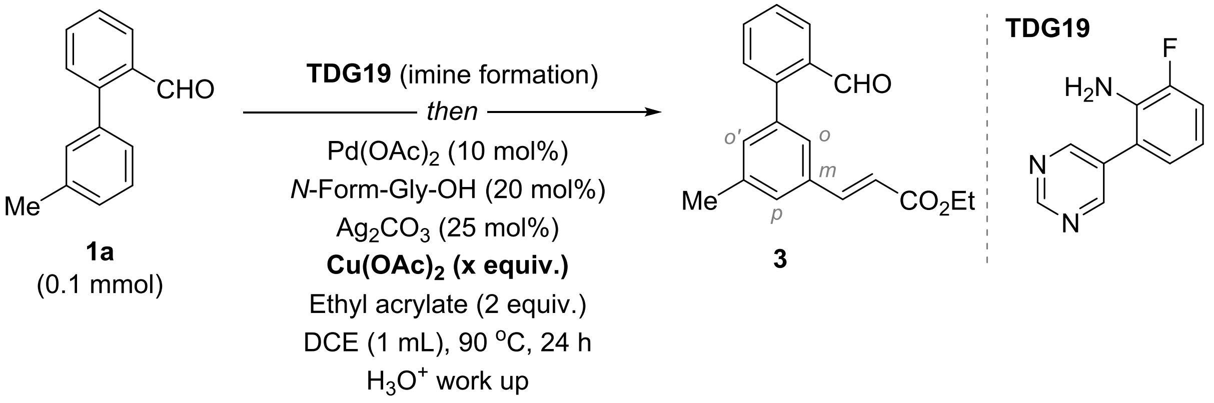

Entry	$Cu(OAc)_2$ (x equiv.)	Yield (*m*:others)[a]
1	0.5	43% (12:1)
2	1	48% (13:1)
3	1.5	50% (15:1)
4	2	55% (14:1)
5	2.5	62% (15:1)
6	3	65% (15:1)
7	**3.5**	**68% (15:1)**
8	4	66% (14:1)
9	5	59% (11:1)

[a]Yield and selectivity are based on ^{1}H NMR of the crude reaction mixture using 1,3,5-trimethoxybenzene (TMB) as internal standard. Doublet of olefin proton in ^{1}H NMR was used to measure the selectivity. Ratios of *meta*:others are shown in parenthesis. nd, not detected.

Supplementary Table 10: Temperature optimization

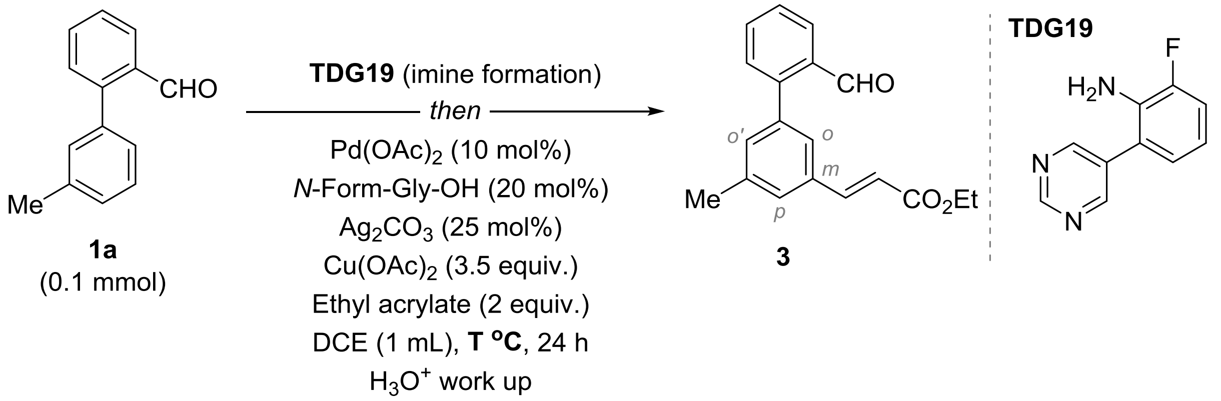

Entry	Temperature (°C)	Yield (*m*:others)[a]
1	RT	nd
2	60	33% (15:1)
3	70	47% (15:1)
4	80	61% (15:1)
5	90	68% (15:1)
**6**	**100**	**72% (15:1)**
7	110	69% (11:1)
8	120	64% (9:1)
9	130	53% (8:1)

[a]Yield and selectivity are based on ^{1}H NMR of the crude reaction mixture using 1,3,5-trimethoxybenzene (TMB) as internal standard. Doublet of olefin proton in ^{1}H NMR was used to measure the selectivity. Ratios of *meta*:others are shown in parenthesis. nd, not detected.

Supplementary Table 11: Optimization of olefin amount

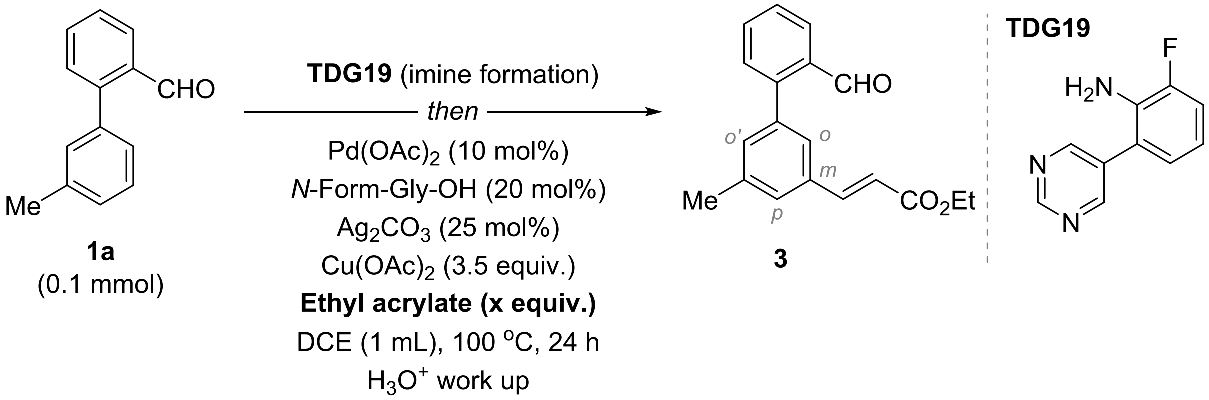

Entry	Ethyl acrylate (x equiv.)	Yield (*m*:others)[a]
1	1	43% (15:1)
2	1.5	58% (15:1)
3	2	72% (15:1)
4	2.5	74% (15:1)
**5**	**3**	**77% (15:1)**
6	3.5	76% (15:1)

[a]Yield and selectivity are based on ^{1}H NMR of the crude reaction mixture using 1,3,5-trimethoxybenzene (TMB) as internal standard. Doublet of olefin proton in ^{1}H NMR was used to measure the selectivity. Ratios of *meta*:others are shown in parenthesis. nd, not detected.

Supplementary Table 12: Optimization of TDG19 amount in a single step

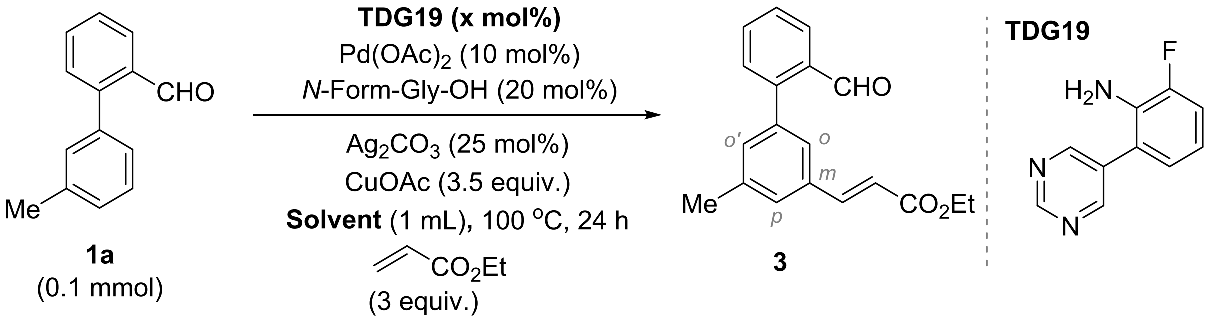

Entry[a]	TDG19 (x mol%)	Solvent	Yield[b]
1	10	DCE (1 mL)	8% (multiple products)
2	20	DCE (1 mL)	15% (multiple products)
3	30	DCE (1 mL)	22% (1:1)
4	50	DCE (1 mL)	33% (3:1)
5	100	DCE (1 mL)	42% (5:1)
6	10	HFIP (1 mL)	12% (multiple products)
7	20	HFIP (1 mL)	16% (multiple products)

8	30	HFIP (1 mL)	24% (multiple products)
9	50	HFIP (1 mL)	35% (1:1)
10	100	HFIP (1 mL)	44% (3:1)
11	100	[DCE (0.5 mL) + HFIP (0.5 mL)]	38% (3:1)
12	100	^{i}PrOH (1 mL)	nd

[a]Substrate and **TDG19** were taken in the respective solvent medium and stirred for 5 minute at room temperature. Then, the remaining reagents were added and stirred at 100 °C for 24 h. [b]Yields are based on ^{1}H NMR of the crude reaction mixture using 1,3,5-trimethoxybenzene as an internal standard. Ratio of *meta*:others are shown in parenthesis. nd, not detected.

2.8 General procedure D: Procedure for *meta*-olefination of 2-phenylbenzaldehyde substrates:

An oven-dried screw capped reaction tube with a magnetic stir-bar was charged with 2-phenylbenzaldehyde (0.2 mmol) (viscous biphenyl aldehyde was weighed first), and **TDG19** (0.19 mmol, 36 mg) under air, followed by isopropyl alcohol (2 mL). The reaction mixture was stirred at 80 °C for 2 hours. The mixture was allowed to cool down and almost full conversion was observed in thin layer chromatography. Next, solvent was concentrated in vacuo and washed with pentane. The dry crude solid residue was subjected to $Pd(OAc)_2$ (10 mol%, 0.02 mmol, 4.5 mg), *N*-formyl glycine (*N*-Form-Gly-OH; 20 mol%, 8.3 mg), Ag_2CO_3 (25 mol%, 0.05 mmol, 14 mg) and $Cu(OAc)_2$ (3.5 equiv., 0.7 mmol, 127 mg) in the same reaction tube. Solvent 1,2-dichloroethane (DCE, 2 mL) was added in the reaction tube followed by addition of liquid alkene (3 equiv., 0.6 mmol) by micropipette under air (solid alkenes were weighed before adding solvent). The reaction tube was screwed by a cap fitted with a rubber septum and was vigorously stirred in a preheated oil bath at 100 °C. The reaction mixture was taken out after 24 h, diluted with 10 mL ethyl acetate and filtered through a celite pad. Next, the filtrate mixture was treated with 1 (M) HCl solution and stirred for ten minutes. Organic part was washed one more time with NH_4Cl solution and concentrated under vacuum. The crude mixture was purified by column chromatography using silica gel (100-200 mesh size) and petroleum ether/ethyl acetate as the eluent.

2.9 Characterization data for *meta*-olefination products of 2-phenylbenzaldehydes

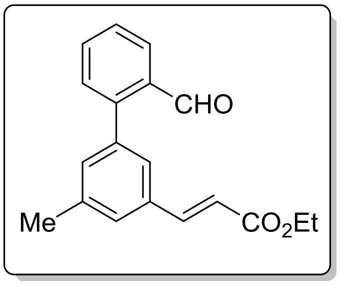

Ethyl (E)-3-(2'-formyl-5-methyl-[1,1'-biphenyl]-3-yl)acrylate: Compound **3** was prepared by general procedure D (0.2 mmol scale).
Eluent: petroleum ether/ethyl acetate (98/2, v/v).
Physical State: colorless oil.
Yield: 74% (43.6 mg isolated; *m*:others = 15:1).
^{1}H NMR (400 MHz, $CDCl_3$) δ (ppm) 9.98 (s, 1H), 8.03 (dd, *J* = 7.8, 1.1 Hz, 1H), 7.70 (d, *J* = 16.0 Hz, 1H), 7.65 (td, *J* = 7.5, 1.5 Hz, 1H), 7.54 – 7.49 (m, 1H), 7.45 – 7.42 (m, 1H), 7.41 (s, 1H), 7.34 (s, 1H), 7.21 (s, 1H), 6.47 (d, *J* = 16.0 Hz, 1H), 4.27 (q, *J* = 7.1 Hz, 2H), 2.44 (s, 3H), 1.34 (t, *J* = 7.1 Hz, 3H).
^{13}C NMR (101 MHz, $CDCl_3$) δ (ppm) 192.34, 167.01, 145.44, 144.14, 139.07, 138.71, 134.95, 133.96, 133.87, 132.82, 130.88, 128.58, 128.28, 127.94, 126.96, 119.41, 60.80, 21.54, 14.52.
IR (thin film, cm^{-1}): 1037, 1095, 1177, 1262, 1367, 1464, 1596, 1639, 1694.91, 2752, 2854, 2925.
HRMS (*m/z*): [M+Na^+] calcd for $C_{19}H_{18}NaO_3$: 317.1148; found, 317.1152.
TLC: R_f = 0.5 (95:5 petroleum ether:EtOAc).

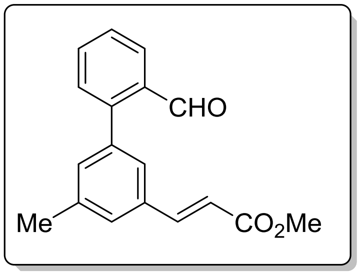

***Methyl (E)-3-(2'-formyl-5-methyl-[1,1'-biphenyl]-3-yl)acrylate*:** Compound **4** was prepared by general procedure D (0.2 mmol scale).
Eluent: petroleum ether/ethyl acetate (95/5, v/v).
Physical State: yellow oil.
Yield: 73% (41 mg isolated; *m*:others = 13:1).
^{1}H NMR (400 MHz, $CDCl_3$) δ (ppm) 9.97 (s, 1H), 8.04 (dd, *J* = 7.8, 1.2 Hz, 1H), 7.71 (d, *J* = 16.0 Hz, 1H), 7.67 – 7.63 (m, 1H), 7.52 (t, *J* = 7.6 Hz, 1H), 7.45 – 7.41 (m, 2H), 7.34 (s, 1H), 7.21 (s, 1H), 6.48 (d, *J* = 16.0 Hz, 1H), 3.81 (s, 3H), 2.44 (s, 3H).
^{13}C NMR (126 MHz, $CDCl_3$) δ (ppm) 192.32, 167.46, 145.40, 144.46, 139.09, 138.74, 134.86, 133.95, 133.88, 132.91, 130.88, 128.59, 128.30, 127.95, 126.96, 118.90, 51.99, 21.54.
IR (thin film, cm^{-1}): 1170, 1214, 1281, 1393, 1437, 1596, 1639, 1693, 2755, 2855, 2953, 3022.
HRMS (ESI): [M+Na^+] calcd for $C_{18}H_{16}NaO_3$: 303.0997; found, 303.0993.

TLC: R_f = 0.6 (90:10 petroleum ether:EtOAc).

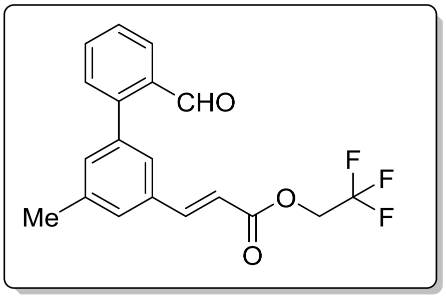

***2,2,2-trifluoroethyl (E)-3-(2'-formyl-5-methyl-[1,1'-biphenyl]-3-yl)acrylate*:** Compound **5** was prepared by general procedure D (0.2 mmol scale).
Eluent: petroleum ether/ethyl acetate (98/2, v/v).
Physical State: colorless solid.
Yield: 70% (49 mg isolated; *m*:others = 10:1).
^{1}H NMR (500 MHz, $CDCl_3$) δ (ppm) 9.97 (s, 1H), 8.04 (dd, *J* = 7.8, 1.2 Hz, 1H), 7.80 (d, *J* = 16.0 Hz, 1H), 7.66 (td, *J* = 7.5, 1.4 Hz, 1H), 7.53 (t, *J* = 7.6 Hz, 1H), 7.43 (dd, *J* = 9.6, 1.5 Hz, 2H), 7.37 (s, 1H), 7.25 (s, 1H), 6.53 (d, *J* = 16.0 Hz, 1H), 4.59 (q, *J* = 8.4 Hz, 2H), 2.45 (s, 3H).
^{13}C NMR (126 MHz, $CDCl_3$) δ (ppm) 192.24, 165.21, 146.75, 145.19, 139.25, 138.91, 134.33, 133.92, 133.47, 130.88, 128.83, 128.40, 128.05, 127.21, 123.3 (q, *J* = 270.6 Hz, CF_3), 117.00, 60.6 (q, *J* = 36.6 Hz, CF_3), 21.53.
IR (thin film, cm^{-1}): 1077, 1149, 1278, 1407, 1596, 1638, 1693, 1734, 2753, 2854, 2924.
^{19}F NMR (471 MHz, $CDCl_3$) δ (ppm) -73.70.
HRMS (*m/z*): [M+Na$^+$] calcd for $C_{19}H_{15}F_3NaO_3$: 371.0865; found, 371.0864.
TLC: R_f = 0.6 (90:10 petroleum ether:EtOAc).

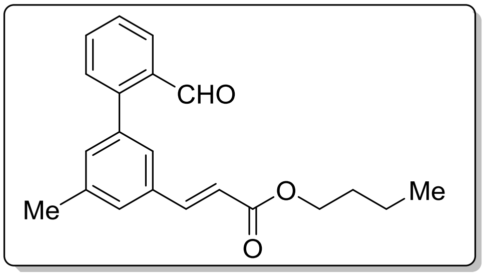

***Butyl (E)-3-(2'-formyl-5-methyl-[1,1'-biphenyl]-3-yl)acrylate*:** Compound **6** was prepared by general procedure D (0.2 mmol scale).
Eluent: petroleum ether/ethyl acetate (97/3, v/v).
Physical State: colorless oil.
Yield: 64% (41 mg isolated; *m*:others = 12:1).
^{1}H NMR (500 MHz, $CDCl_3$) δ (ppm) 9.98 (s, 1H), 8.03 (dd, *J* = 7.8, 1.1 Hz, 1H), 7.69 (d, *J* = 16.0 Hz, 1H), 7.65 (td, *J* = 7.5, 1.3 Hz, 1H), 7.52 (t, *J* = 7.6 Hz, 1H), 7.45 – 7.41 (m, 2H), 7.34 (s, 1H), 7.20 (s, 1H), 6.48 (d, *J* = 16.0 Hz, 1H), 4.21 (t, *J* = 6.7 Hz, 2H), 2.44 (s, 3H), 1.71 – 1.66 (m, 2H), 1.46 – 1.41 (m, 2H), 0.96 (t, *J* = 7.4 Hz, 3H).

^{13}C NMR (126 MHz, CDCl$_3$) δ (ppm) 192.32, 167.11, 145.46, 144.11, 139.07, 138.75, 135.00, 134.01, 133.86, 132.80, 130.89, 128.60, 128.29, 127.96, 126.96, 119.48, 64.73, 31.00, 21.53, 19.43, 13.94.
IR (thin film, cm^{-1}): 1175, 1215, 1262, 1392, 1459, 1596, 1640, 1696, 2854, 2926, 3021.
HRMS (ESI): [M+Na$^+$] calcd for $C_{21}H_{22}NaO_3$: 345.1461; found: 345.1461.
TLC: R_f = 0.5 (90:10 petroleum ether:EtOAc).

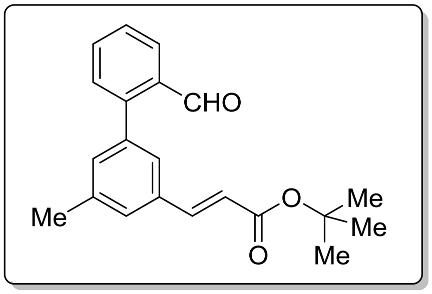

Tert-butyl (E)-3-(2'-formyl-5-methyl-[1,1'-biphenyl]-3-yl)acrylate: Compound **7** was prepared by general procedure D (0.2 mmol scale).
Eluent: petroleum ether/ethyl acetate (98/2, v/v).
Physical State: yellow oil.
Yield: 61% (39 mg isolated; *m*:others = 12:1).
^{1}H NMR (400 MHz, CDCl$_3$) δ (ppm) 9.97 (s, 1H), 8.03 (dd, J = 7.8, 1.1 Hz, 1H), 7.64 (td, J = 7.5, 1.4 Hz, 1H), 7.59 (d, J = 16.0 Hz, 1H), 7.53 – 7.49 (m, 1H), 7.43 (dd, J = 7.7, 0.8 Hz, 1H), 7.39 (s, 1H), 7.32 (s, 1H), 7.19 (s, 1H), 6.43 – 6.38 (m, 1H), 2.43 (s, 3H), 1.53 (s, 9H).
^{13}C NMR (126 MHz, CDCl$_3$) δ (ppm) 192.40, 166.32, 145.55, 143.07, 138.99, 138.62, 135.15, 133.96, 133.85, 132.56, 130.88, 128.53, 128.23, 127.89, 126.86, 121.35, 80.87, 28.41, 21.54.
IR (thin film, cm^{-1}): 1151, 1214, 1259, 1297, 1368, 1393, 1596, 1638, 1696, 2858, 2927, 2980, 3021.
HRMS (ESI): [M+Na$^+$] calcd for $C_{21}H_{22}NaO_3$: 345.1461; found, 345.1461.
TLC: R_f = 0.7 (90:10 petroleum ether:EtOAc).

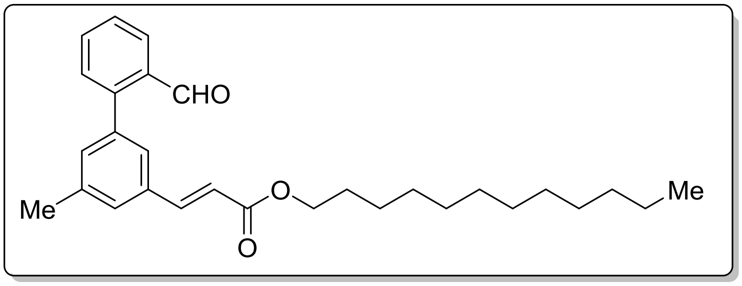

***Dodecyl (E)-3-(2'-formyl-5-methyl-[1,1'-biphenyl]-3-yl)acrylate*:** Compound **8** was prepared by general procedure D (0.2 mmol scale).
Eluent: petroleum ether/ethyl acetate (97/3, v/v).
Physical State: colorless oil.
Yield: 65% (56 mg isolated; *m*:others = 7:1).
^{1}H NMR (400 MHz, CDCl$_3$) δ (ppm) 9.98 (s, 1H), 8.03 (dd, J = 7.8, 1.2 Hz, 1H), 7.69 (d, J = 16.0 Hz, 1H), 7.67 – 7.63 (m, 1H), 7.52 (t, J = 7.5 Hz, 1H), 7.43 (dd, J = 9.5, 1.4 Hz, 2H), 7.34 (s,

1H), 7.20 (s, 1H), 6.48 (d, J = 16.0 Hz, 1H), 4.20 (t, J = 6.7 Hz, 2H), 2.44 (s, 3H), 1.72 – 1.67 (m, 2H), 1.45 – 1.36 (m, 2H), 1.25 (s, 16H), 0.87 (t, J = 6.9 Hz, 3H).
^{13}C NMR (126 MHz, $CDCl_3$) δ (ppm) 192.17, 166.93, 145.25, 143.90, 138.85, 138.48, 134.75, 133.73, 133.67, 132.61, 130.67, 128.40, 128.08, 127.72, 126.73, 119.21, 64.84, 31.92, 29.66, 29.64, 29.60, 29.55, 29.35, 29.30, 28.72, 25.99, 22.70, 21.33, 14.13.
IR (thin film, cm^{-1}): 1168, 1262, 1295, 1393, 1466, 1596, 1641, 1696, 2854, 2925.
HRMS (ESI): [$M+K^+$] calcd for $C_{29}H_{38}KO_3$: 473.2455; found, 473.2453.
TLC: R_f = 0.6 (90:10 petroleum ether:EtOAc).

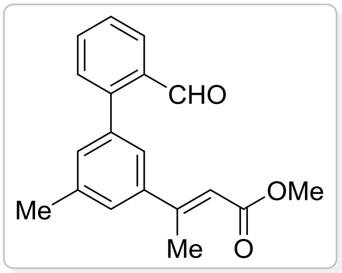

Methyl (E)-3-(2'-formyl-5-methyl-[1,1'-biphenyl]-3-yl)but-2-enoate**:** Compound **9** was prepared by general procedure D (0.2 mmol scale).
Eluent: petroleum ether/ethyl acetate (97/3, v/v).
Physical State: Yellow oil.
Yield: 62% (36 mg isolated; *m*:others = 12:1).
^{1}H NMR (500 MHz, $CDCl_3$) δ (ppm) 9.98 (s, 1H), 8.03 (d, J = 7.7 Hz, 1H), 7.65 (t, J = 7.5 Hz, 1H), 7.51 (t, J = 7.5 Hz, 1H), 7.44 (d, J = 7.6 Hz, 1H), 7.35 (s, 1H), 7.27 (s, 1H), 7.19 (s, 1H), 6.17 (s, 1H), 3.76 (s, 3H), 2.60 (s, 3H), 2.45 (s, 3H).
^{13}C NMR (126 MHz, $CDCl_3$) δ (ppm) 192.47, 167.32, 155.51, 145.79, 142.74, 138.67, 138.32, 133.98, 133.84, 131.67, 130.95, 128.19, 127.88, 127.05, 125.40, 117.55, 51.40, 21.66, 18.34.
IR (thin film, cm^{-1}): 1168, 1214, 1437, 1596, 1629, 1696, 2855, 2926, 3021.
HRMS (ESI): [$M+Na^+$] calcd for $C_{19}H_{18}NaO_3$: 317.1150; found, 317.1148
TLC: R_f = 0.6 (90:10 petroleum ether:EtOAc).

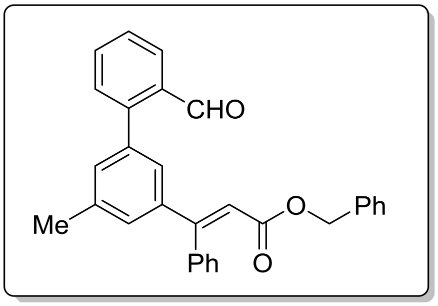

Benzyl (E)-3-(2'-formyl-5-methyl-[1,1'-biphenyl]-3-yl)-3-phenylacrylate**:** Compound **10** was prepared by general procedure D (0.2 mmol scale).
Eluent: petroleum ether/ethyl acetate (97/3, v/v).
Physical State: colorless oil.
Yield: 66% (57 mg isolated; *m*:others = 10:1).

^{1}H NMR (500 MHz, CDCl$_3$) δ (ppm) 9.93 (s, 1H), 7.99 (dd, *J* = 7.8, 1.1 Hz, 1H), 7.60 (td, *J* = 7.5, 1.4 Hz, 1H), 7.48 (t, *J* = 7.6 Hz, 1H), 7.39 – 7.36 (m, 5H), 7.32 – 7.29 (m, 3H), 7.23 (dd, *J* = 6.6, 3.0 Hz, 2H), 7.20 – 7.18 (m, 4H), 7.08 (s, 1H), 6.43 (s, 1H), 5.05 (s, 2H), 2.38 (s, 3H)
^{13}C NMR (126 MHz, CDCl$_3$) δ (ppm) 192.30, 165.91, 156.72, 145.60, 141.39, 138.73, 138.57, 138.14, 136.00, 133.87, 133.80, 132.13, 130.90, 129.34, 129.11, 128.91, 128.64, 128.46, 128.30, 128.27, 128.16, 127.83, 127.39, 117.94, 66.30, 21.58.
IR (thin film, cm^{-1}): 697, 822, 864, 1004, 1155, 1197, 1269, 1377, 1455, 1496, 1594, 1693, 1721, 2750, 2856, 2924, 3032.
HRMS (ESI): [M+Na$^+$] calcd for $C_{30}H_{24}NaO_3$: 455.1624, found 455.1630.
TLC: R$_f$ = 0.5 (90:10 petroleum ether:EtOAc).

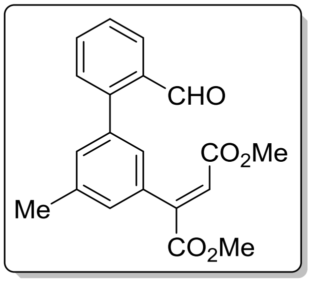

***Dimethyl 2-(2'-formyl-5-methyl-[1,1'-biphenyl]-3-yl)fumarate*:** Compound **11** was prepared by general procedure D (0.2 mmol scale).
Eluent: petroleum ether/ethyl acetate (97/3, v/v).
Physical State: white semi-solid.
Yield: 61% (35 mg isolated; *m*:others = 12:1).
^{1}H NMR (500 MHz, CDCl$_3$) δ (ppm) 9.95 (s, 1H), 8.03 (dd, *J* = 7.8, 1.1 Hz, 1H), 7.65 (td, *J* = 7.5, 1.4 Hz, 1H), 7.52 (t, *J* = 7.6 Hz, 1H), 7.41 (dd, *J* = 7.7, 0.7 Hz, 1H), 7.35 (s, 1H), 7.29 (s, 1H), 7.25 (s, 1H), 6.35 (s, 1H), 3.95 (s, 3H), 3.79 (s, 3H), 2.44 (s, 3H).
^{13}C NMR (126 MHz, CDCl$_3$) δ (ppm) 192.16, 168.39, 165.50, 148.66, 145.08, 139.36, 138.94, 133.93, 133.90, 133.75, 133.26, 130.92, 128.43, 128.00, 127.35, 125.53, 118.14, 53.09, 52.35, 21.62.
IR (thin film, cm^{-1}): 1169, 1196, 1257, 1348, 1394, 1436, 1595, 1626, 1692, 1723, 2755, 2854, 2924, 2953.
HRMS (ESI): [M+Na$^+$] calcd for $C_{20}H_{18}NaO_5$: 361.1044; found, 361.1046.
TLC: R$_f$ = 0.5 (90:10 petroleum ether:EtOAc).

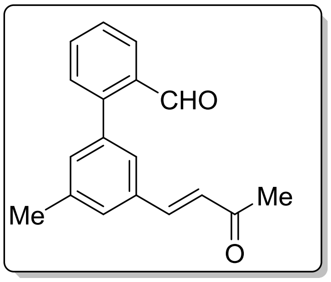

(E)-3'-methyl-5'-(3-oxobut-1-en-1-yl)-[1,1'-biphenyl]-2-carbaldehyde: Compound **12** was prepared by general procedure D (0.2 mmol scale).
Eluent: petroleum ether/ethyl acetate (97/3, v/v).

Physical State: colourless oil.
Yield: 71% (37 mg isolated; *m*:others = 10:1).
^{1}H NMR (400 MHz, CDCl$_3$) δ (ppm) 9.98 (s, 1H), 8.04 (dd, *J* = 7.8, 1.1 Hz, 1H), 7.66 (td, *J* = 7.5, 1.4 Hz, 1H), 7.55 – 7.51 (m, 2H), 7.44 (d, *J* = 6.5 Hz, 2H), 7.35 (s, 1H), 7.23 (s, 1H), 6.75 (d, *J* = 16.3 Hz, 1H), 2.45 (s, 3H), 2.39 (s, 3H).
^{13}C NMR (126 MHz, CDCl$_3$) δ (ppm) 198.36, 192.32, 145.34, 142.93, 139.20, 138.83, 134.93, 133.91, 133.08, 130.88, 128.75, 128.34, 128.02, 128.01, 127.24, 127.22, 27.92, 21.56.
IR (thin film, cm^{-1}): 704, 765, 822, 871, 979, 1108, 1194, 1260, 1359, 1393, 1456, 1596, 1692, 2751, 2853, 2926.
HRMS (ESI): [M+Na$^+$] calcd for $C_{18}H_{16}NaO_2$: 287.1047, found 287.1052.
TLC: R$_f$ = 0.5 (90:10 petroleum ether:EtOAc).

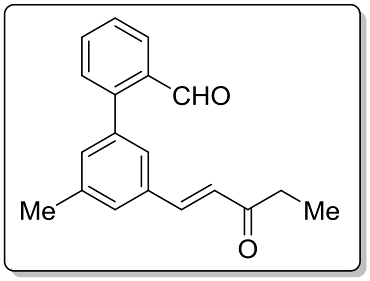

(E)-3'-methyl-5'-(3-oxopent-1-en-1-yl)-[1,1'-biphenyl]-2-carbaldehyde: Compound **13** was prepared by general procedure D (0.2 mmol scale).
Eluent: petroleum ether/ethyl acetate (97/3, v/v).
Physical State: colorless oil.
Yield: 72% (40 mg isolated; *m*:others = 12:1).
^{1}H NMR (400 MHz, CDCl$_3$) δ (ppm) 9.99 (s, 1H), 8.04 (d, *J* = 6.7 Hz, 1H), 7.65 (td, *J* = 7.6, 1.3 Hz, 1H), 7.56 (d, *J* =16.2 1H), 7.52 (t, *J* = 7.6 Hz, 1H), 7.47 – 7.41 (m, 2H), 7.36 (s, 1H), 7.22 (s, 1H), 6.78 (d, *J* = 16.2 Hz, 1H), 2.70 (q, *J* = 7.3 Hz, 2H), 2.45 (s, 3H), 1.17 (t, *J* = 7.3 Hz, 3H).
^{13}C NMR (126 MHz, CDCl$_3$) δ (ppm) 200.80, 192.21, 145.22, 141.55, 138.94, 138.54, 134.85, 133.72, 132.75, 130.68, 128.61, 128.12, 127.75, 126.92, 126.64, 34.30, 21.36, 8.19.
IR (thin film, cm^{-1}): 1041, 1120, 1192, 1261, 1459, 1614, 1666, 1693, 2851, 2918.
HRMS (ESI): [M+Na$^+$] calcd for $C_{19}H_{18}NaO_2$: 301.1199; found 301.1198.
TLC: R$_f$ = 0.5 (90:10 petroleum ether:EtOAc).

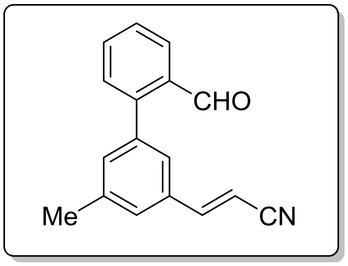

***(E)-3-(2'-formyl-5-methyl-[1,1'-biphenyl]-3-yl)acrylonitrile*:** Compound **14** was prepared by general procedure D (0.2 mmol scale).
Eluent: petroleum ether/ethyl acetate (97/3, v/v).
Physical State: colorless oil.

Yield: 57% (28 mg isolated; *m*:others = 3:1).
^{1}H NMR (400 MHz, $CDCl_3$) δ (ppm) 9.95 (s, 1H), 8.04 (dd, *J* = 7.8, 1.3 Hz, 1H), 7.66 (ddd, *J* = 7.5, 4.5, 1.4 Hz, 1H), 7.59 – 7.51 (m, 2H), 7.45 – 7.40 (m, 2H), 7.34 (s, 1H), 7.26 (s, 1H), 5.93 (d, *J* = 16.6 Hz, 1H), 2.45 (s, 3H).
^{13}C NMR (126 MHz, $CDCl_3$) δ (ppm) 192.10, 150.26, 148.44, 144.88, 139.44, 139.11, 133.97, 133.75, 130.98, 130.84, 128.53, 128.17, 127.82, 126.19, 118.14, 97.45, 96.37, 21.52
IR (thin film, cm^{-1}): 701, 766, 821, 870, 967, 1041, 1108, 1194, 1261, 1394, 1453, 1595, 1620, 1691, 2217, 2752, 2854, 2925, 3065.
HRMS (ESI): [$M+Na^+$] calcd for $C_{17}H_{13}NaNO$: 270.0896, found 270.0892.
TLC: R_f = 0.5 (90:10 petroleum ether:EtOAc).

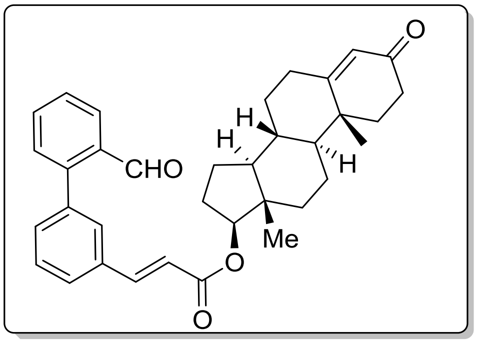

***(8R,9S,10R,13S,14S,17S)-10,13-dimethyl-3-oxo-2,3,6,7,8,9,10,11,12,13,14,15,16,17-tetradecahydro-1H-cyclopenta[a]phenanthren-17-yl (E)-3-(2'-formyl-[1,1'-biphenyl]-3-yl)acrylate*:** Compound **15** was prepared by general procedure D (0.2 mmol scale).
Eluent: petroleum ether/ethyl acetate (95/5, v/v).
Physical State: colorless oil.
Yield: 59% (62 mg isolated; *m*:others = 8:1).
^{1}H NMR (400 MHz, $CDCl_3$) δ (ppm) 9.94 (s, 1H), 8.00 (d, *J* = 7.7 Hz, 1H), 7.66 – 7.59 (m, 2H), 7.52 – 7.48 (m, 2H), 7.47 – 7.41 (m, 2H), 7.41 – 7.38 (m, 1H), 7.31 (d, *J* = 7.5 Hz, 1H), 6.49 – 6.43 (m, 1H), 6.13 – 5.96 (m, 1H), 3.96 – 3.81 (m, 1H), 2.50 – 2.09 (m, 2H), 2.06 – 1.79 (m, 4H), 1.75 – 1.49 (m, 6H), 1.45 – 1.25 (m, 4H), 1.29 – 0.98 (m, 7H), 1.01 – 0.78 (m, 2H).
^{13}C NMR (101 MHz, $CDCl_3$) δ (ppm) 192.23, 192.06, 164.70, 145.25, 143.68, 139.70, 138.29, 135.34, 133.74, 133.67, 130.98, 130.72, 128.93, 128.08, 127.71, 127.63, 123.91, 122.45, 119.56, 82.72, 53.70, 50.29, 48.44, 42.74, 38.63, 35.41, 33.97, 33.13, 31.49, 27.61, 25.53, 24.87, 20.54, 17.40, 12.18.
IR (thin film, cm^{-1}): 1041, 1091, 1196, 1253, 1343, 1393, 1449, 1542, 1597, 1619, 1655, 1692, 1946, 2752, 2854, 2930, 3061, 3281.
HRMS (ESI): [$M+Na^+$] calcd for $C_{35}H_{38}NaO_4$: 545.2662; found 545.2666
TLC: R_f = 0.4 (90:10 petroleum ether:EtOAc).

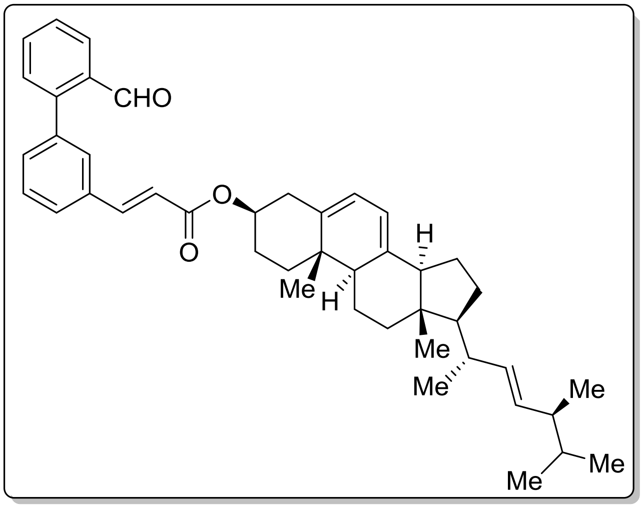

(3R,9R,10S,13S,14S,17S)-17-((2S,5S,E)-5,6-dimethylhept-3-en-2-yl)-10,13-dimethyl-2,3,4,9,10,11,12,13,14,15,16,17-dodecahydro-1H-cyclopenta[a]phenanthren-3-yl (E)-3-(2'-formyl-[1,1'-biphenyl]-3-yl)acrylate: Compound **16** was prepared by general procedure D (0.2 mmol scale).

Eluent: petroleum ether/ethyl acetate (95/5, v/v).

Physical State: colorless oil.

Yield: 62% (78 mg isolated; *m*:others = 8:1).

^{1}H NMR (400 MHz, $CDCl_3$) δ (ppm) 9.98 (s, 1H), 8.04 (d, *J* = 7.8 Hz, 1H), 7.72 (d, *J* = 16.0 Hz, 1H), 7.66 (td, *J* = 7. 6, 2.3 Hz, 2H), 7.61 (d, *J* = 7.9 Hz, 1H), 7.53 (s, 1H), 7.49 (d, *J* = 7.8 Hz, 1H), 7.44 (d, *J* = 7.6 Hz, 1H), 7.39 (d, *J* = 7.9 Hz, 1H), 6.49 (d, *J* = 16.0 Hz, 1H), 5.60 (d, *J* = 3.5 Hz, 1H), 5.42 – 5.37 (m, 1H), 5.21 (t, *J* = 15.1, 2H), 4.85 (ddd, *J* = 15.8, 11.3, 4.5 Hz, 1H), 2.62 – 2.56 (m, 1H), 2.45 (t, *J* = 13.0 Hz, 1H), 2.07 – 1.98 (m, 4H), 1.96 – 1.82 (m, 4H), 1.73 – 1.60 (m, 6H), 1.50 – 1.43 (m, 2H), 1.41 – 1.29 (m, 4H), 1.04 (d, *J* = 6.6 Hz, 3H), 0.98 (s, 3H), 0.92 (d, *J* = 6.8 Hz, 3H), 0.82 (d, *J* = 6.1 Hz, 4H), 0.64 (s, 3H).

^{13}C NMR (101 MHz, $CDCl_3$) δ (ppm) 192.03, 166.33, 145.02, 143.69, 141.67, 138.51, 135.58, 134.84, 133.74, 131.98, 130.72, 129.44, 129.01, 128.20, 127.86, 127.65, 120.30, 119.77, 116.33, 73.10, 55.71, 54.54, 46.05, 42.82, 40.46, 39.04, 37.94, 37.13, 36.75, 33.10, 28.31, 28.22, 23.01, 21.13, 21.05, 19.98, 19.67, 17.62, 16.21, 12.08.

IR (thin film, cm^{-1}): 1012, 1170, 1255, 1371, 1457, 1597, 1638, 1692, 2870, 2957.

HRMS (ESI): [$M+K^+$] calcd for $C_{44}H_{54}KO_3$: 669.3705; found 669.3709.

TLC: R_f = 0.4 (90:10 petroleum ether:EtOAc).

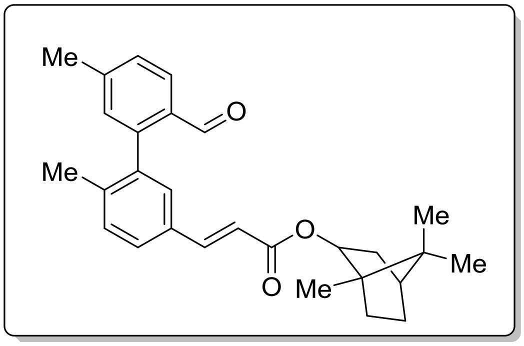

***(4R)-1,7,7-trimethylbicyclo[2.2.1]heptan-2-yl (E)-3-(2'-formyl-5',6-dimethyl-[1,1'-biphenyl]-3-yl)acrylate*:** Compound **17** was prepared by general procedure D (0.2 mmol scale).
Eluent: petroleum ether/ethyl acetate (95/5, v/v).
Physical State: yellow oil.
Yield: 68% (57 mg isolated; *m*:others = 12:1).
^{1}H NMR (400 MHz, CDCl$_3$) δ (ppm) 9.92 (s, 1H), 7.98 (d, *J* = 15.9 Hz, 1H), 7.94 (d, *J* = 8.0 Hz, 1H), 7.55 (d, *J* = 1.3 Hz, 1H), 7.31 (d, *J* = 8.7 Hz, 1H), 7.28 (d, *J* = 3.4 Hz, 1H), 7.27 – 7.26 (m, 1H), 7.23 (s, 1H), 6.34 (d, *J* = 15.9 Hz, 1H), 4.80 (dd, *J* = 7.6, 4.0 Hz, 1H), 2.49 (s, 3H), 2.47 (s, 3H), 1.89 – 1.83 (m, 2H), 1.78 – 1.69 (m, 3H), 1.23 – 1.12 (m, 2H), 1.05 (s, 3H), 0.89 (s, 3H), 0.86 (s, 3H).
^{13}C NMR (126 MHz, CDCl$_3$) δ (ppm) 192.10, 166.54, 145.59, 144.93, 141.51, 137.62, 136.36, 133.85, 131.69, 131.59, 131.53, 131.00, 129.07, 128.14, 127.80, 120.84, 81.46, 49.12, 47.22, 45.30, 39.10, 33.97, 27.28, 22.05, 20.35, 20.22, 19.68, 11.74.
IR (thin film, cm^{-1}): 821, 980, 1017, 1054, 1109, 1174, 1260, 1304, 1391, 1457, 1604, 1636, 1710, 2751, 2928, 2955.
HRMS (ESI): [M+Na^{+}] calcd for $C_{28}H_{32}NaO_3$: 439.2297; found: 438.2293.
TLC: R$_f$ = 0.5 (90:10 petroleum ether:EtOAc).

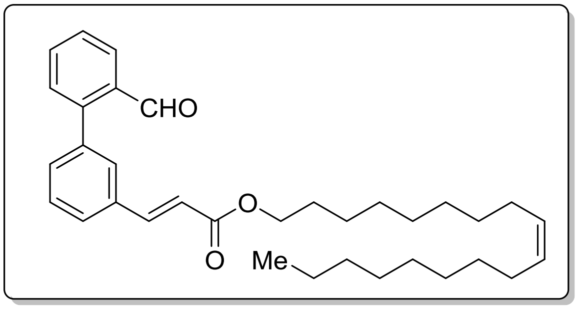

***(Z)-octadec-9-en-1-yl (E)-3-(2'-formyl-[1,1'-biphenyl]-3-yl)acrylate*:** Compound **18** was prepared by general procedure D (0.2 mmol scale).
Eluent: petroleum ether/ethyl acetate (97/3, v/v).
Physical State: colorless oil.
Yield: 49% (48 mg isolated; *m*:others = 10:1).
^{1}H NMR (500 MHz, CDCl$_3$) δ (ppm) 10.00 (s, 1H), 8.07 (dd, *J* = 7.8, 1.1 Hz, 1H), 7.75 (d, *J* = 16.0 Hz, 1H), 7.68 (td, *J* = 7.5, 1.3 Hz, 1H), 7.63 (d, *J* = 7.8 Hz, 1H), 7.58 – 7.55 (m, 2H), 7.52 (t, *J* = 7.6 Hz, 1H), 7.47 (d, *J* = 7.6 Hz, 1H), 7.41 (d, *J* = 7.6 Hz, 1H), 6.52 (d, *J* = 16.0 Hz, 1H), 5.37

(t, *J* = 7.5 Hz, 2H), 4.23 (t, *J* = 6.7 Hz, 2H), 2.10 – 1.97 (m, 4H), 1.72 (dd, *J* = 14.4, 6.8 Hz, 2H), 1.41 (d, *J* = 8.2 Hz, 2H), 1.38 – 1.28 (m, 20H), 0.90 (t, *J* = 6.9 Hz, 3H).
^{13}C NMR (126 MHz, $CDCl_3$) δ (ppm) 191.97, 166.82, 145.01, 143.71, 138.58, 134.84, 133.73, 133.71, 131.74, 130.71, 129.99, 129.80, 129.39, 128.99, 128.19, 127.87, 127.66, 119.45, 64.86, 31.91, 29.77, 29.74, 29.53, 29.44, 29.32, 29.27, 29.23, 28.72, 27.22, 27.20, 25.98, 22.69, 14.12.
IR (thin film, cm^{-1}): 1103, 1166, 1258, 1300, 1392, 1466, 1597, 1639, 1694, 2750, 2853, 2924, 3004.
HRMS (ESI): [$M+Na^+$] calcd for $C_{34}H_{46}NaO_3$: 525.3339; found 525.3338.
TLC: R_f = 0.4 (90:10 petroleum ether:EtOAc).

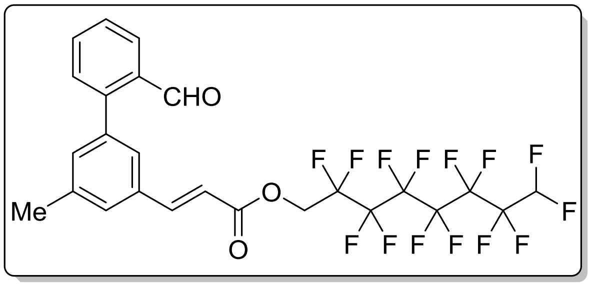

***1,1,2,2,3,3,4,4,5,5,6,6,7,7-tetradecafluoroheptyl (E)-3-(2'-formyl-5-methyl-[1,1'-biphenyl]-3-yl)acrylate*:** Compound **19** was prepared by general procedure D (0.2 mmol scale).
Eluent: petroleum ether/ethyl acetate (97/3, v/v).
Physical State: yellow oil.
Yield: 68% (84 mg isolated; *m*:others = 11:1)
^{1}H NMR (400 MHz, $CDCl_3$) δ (ppm) 9.97 (s, 1H), 8.04 (d, *J* = 6.8 Hz, 1H), 7.79 (d, *J* = 16.0 Hz, 1H), 7.66 (td, *J* = 7.5, 1.3 Hz, 1H), 7.53 (t, *J* = 7.6 Hz, 1H), 7.43 (d, *J* = 8.0 Hz, 2H), 7.37 (s, 1H), 7.25 (s, 1H), 6.52 (d, *J* = 16.0 Hz, 1H), 6.06 (tt, *J* = 8.0, 5.1 Hz, 1H), 4.72 (t, *J* = 13.6 Hz, 2H), 2.45 (s, 3H).
^{13}C NMR (126 MHz, $CDCl_3$) δ (ppm) 192.24, 165.22, 146.84, 145.20, 139.25, 138.91, 134.31, 133.93, 133.50, 132.08, 130.88, 128.86, 128.40, 128.12, 128.03, 127.79, 127.20, 116.92, 107.78, 104.30, 60.11, 59.90, 59.68.
^{19}F NMR (471 MHz, $CDCl_3$) δ (ppm) -119.37, -119.40, -119.43, -122.12, -123.34, -123.41, -123.42, -129.41, -136.95, -136.96, -137.07.
IR (thin film, cm^{-1}): 670, 695, 761, 797, 853, 982, 1018, 1139, 1194, 1260, 1396, 1452, 1597, 1638, 1693, 1735, 2856, 2925, 2964.
HRMS (ESI): [$M+H^+$] calcd for $C_{25}H_{17}F_{14}O_5$: 631.1044; found, 631.1046.
TLC: R_f = 0.6 (90:10 petroleum ether:EtOAc).

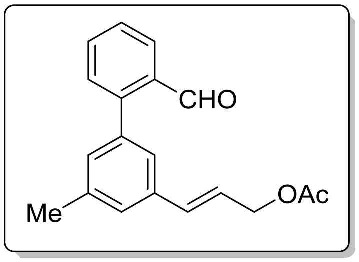

(E)-3-(2'-formyl-5-methyl-[1,1'-biphenyl]-3-yl)allyl acetate: Compound **20** was prepared by general procedure D (0.2 mmol scale).
Eluent: petroleum ether/ethyl acetate (97/3, v/v).
Physical State: colorless oil.
Yield: 50% (29 mg isolated; *m*:others = 4:1).
^{1}H NMR (400 MHz, $CDCl_3$) δ (ppm) 9.98 (s, 1H), 8.02 (d, *J* = 7.8 Hz, 1H), 7.64 (d, *J* = 15.9 Hz, 1H), 7.50 (t, *J* = 7.5 Hz, 1H), 7.43 (d, *J* = 7.5 Hz, 1H), 7.28 (s, 1H), 7.20 (s, 1H), 7.09 (s, 1H), 6.66 (d, *J* = 15.9 Hz, 1H), 6.37 – 6.29 (m, 1H), 4.74 (d, *J* = 6.3 Hz, 2H), 2.41 (s, 3H), 2.10 (s, 3H).
^{13}C NMR (101 MHz, $CDCl_3$) δ (ppm) 192.70, 171.07, 146.04, 138.70, 138.32, 136.61, 133.94, 133.79, 133.67, 130.87, 130.77, 128.05, 127.72, 127.29, 125.74, 124.41, 65.12, 21.56, 21.20.
IR (thin film, cm^{-1}): 705, 767, 821, 868, 966, 1026, 1106, 1228, 1379, 1450, 1596, 1693, 1739, 2752, 2853, 2926.
HRMS (ESI): [M+Na^{+}] calcd for $C_{19}H_{18}NaO_3$: 317.1154, found 317.1159.
TLC: R_f = 0.3 (90:10 petroleum ether:EtOAc).

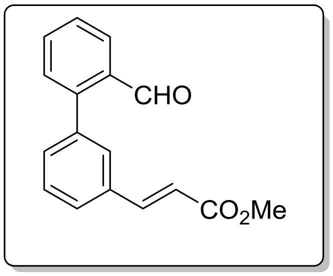

***Methyl (E)-3-(2'-formyl-[1,1'-biphenyl]-3-yl)acrylate*:** Compound **21** was prepared by general procedure D (0.2 mmol scale).
Eluent: petroleum ether/ethyl acetate (95/5, v/v).
Physical State: colorless oil.
Yield: 64% (34 mg isolated; *m*:others = 10:1)
^{1}H NMR (400 MHz, $CDCl_3$) δ (ppm) 9.98 (s, 1H), 8.05 (d, *J* = 7.8 Hz, 1H), 7.74 (d, *J* = 16.0 Hz, 1H), 7.67 (t, *J* = 7.5 Hz, 1H), 7.61 (d, *J* = 7.5 Hz, 1H), 7.55 – 7.48 (m, 3H), 7.44 (d, *J* = 7.7 Hz, 1H), 7.40 (d, *J* = 7.5 Hz, 1H), 6.50 (d, *J* = 16.0 Hz, 1H), 3.82 (s, 3H).
^{13}C NMR (126 MHz, $CDCl_3$) δ (ppm) 192.18, 167.39, 145.19, 144.28, 138.84, 134.97, 133.94, 132.06, 130.93, 129.63, 129.22, 128.43, 128.11, 127.87, 119.16, 52.03
IR (thin film, cm^{-1}): 1034, 1103, 1170, 1195, 1274, 1322, 1394, 1435, 1596, 1639, 1692, 1718, 2752, 2852, 2951, 3062.
HRMS (ESI): [M+Na^{+}] calcd for $C_{17}H_{14}NaO_3$: 289.0835; found: 289.0833.
TLC: R_f = 0.5 (90:10 petroleum ether:EtOAc).

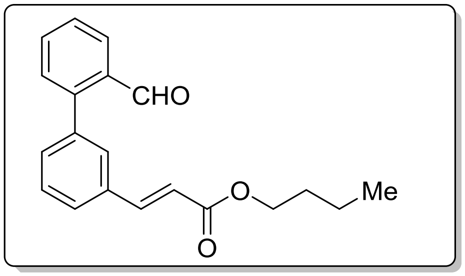

Butyl (E)-3-(2'-formyl-[1,1'-biphenyl]-3-yl)acrylate: Compound **22** was prepared by general procedure D (0.2 mmol scale).
Eluent: petroleum ether/ethyl acetate (97/3, v/v).
Physical State: colorless oil.
Yield: 55% (34 mg isolated; *m*:others = 12:1)
^{1}H NMR (400 MHz, CDCl$_3$) δ (ppm) 9.98 (s, 1H), 8.04 (dd, *J* = 7.8, 1.2 Hz, 1H), 7.72 (d, *J* = 16.0 Hz, 1H), 7.66 (td, *J* = 7.5, 1.4 Hz, 1H), 7.61 (d, *J* = 7.8 Hz, 1H), 7.55 – 7.48 (m, 3H), 7.44 (d, *J* = 7.0 Hz, 1H), 7.39 (d, *J* = 7.6 Hz, 1H), 6.50 (d, *J* = 16.0 Hz, 1H), 4.22 (t, *J* = 6.7 Hz, 2H), 1.73 – 1.65 (m, 3H), 1.47 – 1.41 (m, 2H), 0.96 (t, *J* = 7.4 Hz, 3H).
^{13}C NMR (126 MHz, CDCl$_3$) δ (ppm) 192.34, 166.46, 142.73, 136.66, 135.30, 130.65, 130.24, 129.33, 127.04, 126.46, 120.18, 60.73, 14.12.
IR (thin film, cm^{-1}): 1025, 1064, 1171, 1259, 1301, 1392, 1467, 1597, 1639, 1693, 2752, 2854, 2927, 2959, 3060.
HRMS (ESI): [M+H$^+$] calcd for for $C_{20}H_{21}O_3$: 309.1485, found 309.1489.
TLC: R_f = 0.6 (90:10 petroleum ether:EtOAc).

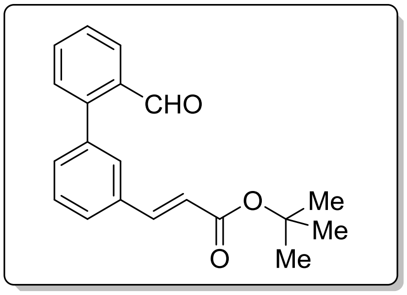

Butyl (E)-3-(2'-formyl-[1,1'-biphenyl]-3-yl)acrylate: Compound **23** was prepared by general procedure D (0.2 mmol scale).
Eluent: petroleum ether/ethyl acetate (97/3, v/v).
Physical State: colorless oil.
Yield: 52% (32 mg isolated; *m*:others = 12:1)
^{1}H NMR (400 MHz, CDCl$_3$) δ (ppm) 9.97 (s, 1H), 8.04 (dd, *J* = 7.8, 1.2 Hz, 1H), 7.68 – 7.60 (m, 2H), 7.58 (d, *J* = 7.8 Hz, 1H), 7.55 – 7.51 (m, 2H), 7.48 (t, *J* = 7.7 Hz, 1H), 7.44 (d, *J* = 7.6 Hz, 1H), 7.37 (d, *J* = 7.5 Hz, 1H), 6.42 (d, *J* = 16.0 Hz, 1H), 1.53 (s, 9H).
^{13}C NMR (126 MHz, CDCl$_3$) δ (ppm) 192.30, 166.26, 145.33, 142.90, 138.68, 135.21, 133.93, 131.70, 130.93, 129.51, 129.16, 128.36, 128.01, 127.81, 121.56, 80.97, 28.39.
IR (thin film, cm^{-1}): 696, 764, 801, 848, 980, 1030, 1103, 1150, 1196, 1257, 1298, 1323, 1368, 1392, 1473, 1597, 1638, 1695, 2851.
HRMS (ESI): [M+H$^+$] calcd for $C_{20}H_{21}O_3$: 309.1485, found 309.1490.
TLC: R_f = 0.6 (90:10 petroleum ether:EtOAc).

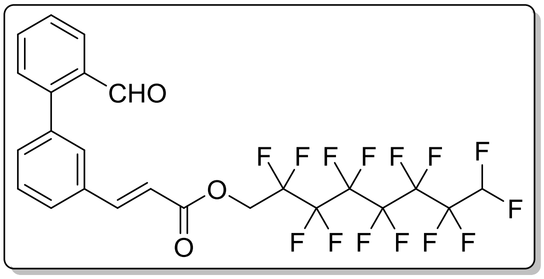

2,2,3,3,4,4,5,5,6,6,7,7,8,8-tetradecafluorooctyl (E)-3-(2'-formyl-[1,1'-biphenyl]-3-yl)acrylate: Compound **24** was prepared by general procedure D (0.2 mmol scale).
Eluent: petroleum ether/ethyl acetate (97/3, v/v).
Physical State: colorless oil.
Yield: 58% (71 mg isolated; *m*:others = 10:1)
^{1}H NMR (500 MHz, CDCl$_3$) δ (ppm) 10.00 (s, 1H), 8.07 (dd, *J* = 7.8, 1.0 Hz, 1H), 7.85 (d, *J* = 16.0 Hz, 1H), 7.69 (td, *J* = 7.5, 1.3 Hz, 1H), 7.66 (d, *J* = 7.8 Hz, 1H), 7.59 (d, *J* = 4.4 Hz, 1H), 7.55 (t, *J* = 7.8 Hz, 2H), 7.49 – 7.44 (m, 2H), 6.59 – 6.54 (m, 1H), 6.20 – 5.97 (m, 1H), 4.75 (t, *J* = 13.6 Hz, 2H).
^{13}C NMR (126 MHz, CDCl$_3$) δ (ppm) 191.88, 164.94, 146.42, 144.76, 138.81, 134.18, 133.7 (d, *J* = 6.65 Hz, C-F), 132.42, 130.72, 129.62, 129.11, 128.31, 127.9 (d, *J* = 3.5 Hz, C-F), 116.96, 107. 5, 59.71 (t, *J* = 26.5 Hz, C-F).
^{19}F NMR (471 MHz, CDCl$_3$) δ (ppm) -119.39, -122.15, -123.33, -123.47, -129.39, -137.00.
IR (thin film, cm^{-1}): 1082, 1140, 1195, 1261, 1301, 1395, 1598, 1638, 1694, 1735, 2756, 2852.
HRMS (ESI): [M+Na$^+$] calcd for $C_{23}H_{14}F_{12}NaO_3$: 589.0644; found 589.0636.
TLC: R$_f$ = 0.4 (90:10 petroleum ether:EtOAc).

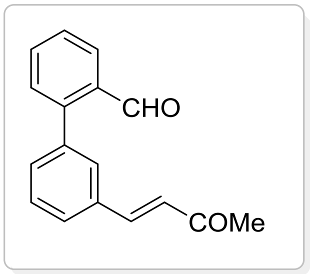

***(E)-3'-(3-oxobut-1-en-1-yl)-[1,1'-biphenyl]-2-carbaldehyde*:** Compound **25** was prepared by general procedure D (0.2 mmol scale).
Eluent: petroleum ether/ethyl acetate (98/2, v/v).
Physical State: Colorless oil.
Yield: 67% (34 mg isolated; *m*:others = 20:1)
^{1}H NMR (400 MHz, CDCl$_3$) δ (ppm) 9.97 (s, 1H), 8.04 (dd, *J* = 7.8, 1.1 Hz, 1H), 7.67 (td, *J* = 7.5, 1.3 Hz, 1H), 7.63 (d, *J* = 7.8 Hz, 1H), 7.56 (d, *J* = 16.3 Hz, 1H), 7.54 (s, 1H), 7.50 (t, *J* = 7.8 Hz, 2H), 7.44 (d, *J* = 7.6 Hz, 1H), 7.41 (d, *J* = 7.6 Hz, 1H), 6.77 (d, *J* = 16.3 Hz, 1H), 2.39 (s, 3H).
^{13}C NMR (101 MHz, CDCl$_3$) δ (ppm) 198.17, 191.98, 144.91, 142.53, 138.69, 134.78, 133.77, 133.70, 132.00, 130.72, 129.67, 129.11, 128.26, 127.95, 127.93, 127.82, 27.74.
IR (thin film, cm^{-1}): 1104, 1197. 1258, 1359, 1394, 1414, 1473, 1597, 1690, 2751, 2849.

HRMS (ESI): [M+Na$^+$] calcd for $C_{17}H_{14}NaO_2$: 273.0886, found 273.0888.
TLC: R$_f$ = 0.6 (90:10 petroleum ether:EtOAc).

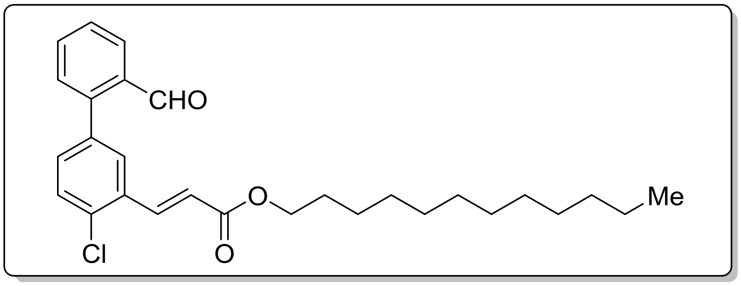

***(E)-4'-chloro-3'-(3-oxopentadec-1-en-1-yl)-[1,1'-biphenyl]-2-carbaldehyde*:** Compound **26** was prepared by general procedure D (0.2 mmol scale).
Eluent: petroleum ether/ethyl acetate (98/2, v/v).
Physical State: yellow oil.
Yield: 60% (53 mg isolated; *m*:others =15:1).
^{1}H NMR (400 MHz, CDCl$_3$) δ (ppm) 10.01 (s, 1H), 8.15 (d, *J* = 16.0 Hz, 1H), 8.07 (d, *J* = 7.7 Hz, 1H), 7.69 (td, *J* = 7.7, 2.0 Hz, 1H), 7.65 (d, *J* = 2.0 Hz, 1H), 7.61 – 7.53 (m, 2H), 7.44 (d, *J* = 7.6 Hz, 1H), 7.35 (dd, *J* = 8.2, 2.0 Hz, 1H), 6.49 (d, *J* = 16.0 Hz, 1H), 4.24 (t, *J* = 6.7 Hz, 2H), 1.76 – 1.69 (m, 2H), 1.45 – 1.39 (m, 2H), 1.33 – 1.24 (m, 16H), 0.90 (t, *J* = 6.9 Hz, 3H).
^{13}C NMR (126 MHz, CDCl$_3$) δ (ppm) 191.80, 166.55, 144.01, 139.94, 137.23, 135.17, 134.04, 133.87, 133.20, 132.44, 130.88, 130.40, 129.08, 128.72, 128.48, 122.11, 65.25, 32.12, 29.86, 29.84, 29.79, 29.75, 29.56, 29.49, 28.88, 26.17, 22.90, 14.33.
IR (thin film, cm^{-1}): 3503, 2925, 2854, 2751, 1711, 1639, 1598, 1465, 1390, 1321, 1275, 1176.
HRMS (ESI): [M+Na$^+$] calcd for $C_{28}H_{35}ClNaO_3$: 477.2166; found, 477.2168.
TLC: R$_f$ = 0.6 (90:10 petroleum ether:EtOAc).

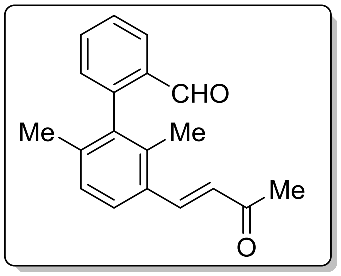

***(E)-2',6'-dimethyl-3'-(3-oxobut-1-en-1-yl)-[1,1'-biphenyl]-2-carbaldehyde*:** Compound **27** was prepared by general procedure D (0.2 mmol scale).
Eluent: petroleum ether/ethyl acetate (96/4, v/v).
Physical State: colorless oil.
Yield: 63% (34 mg isolated; *m*:others = 8:1).
^{1}H NMR (400 MHz, CDCl$_3$) δ (ppm) 9.65 (s, 1H), 8.06 (dd, *J* = 7.8, 1.0 Hz, 1H), 7.85 (d, *J* = 16.0 Hz, 1H), 7.70 (td, *J* = 7.5, 1.4 Hz, 1H), 7.56 (d, *J* = 7.9 Hz, 1H), 7.54 (t, *J* = 7.6 Hz, 1H), 7.21 – 7.17 (m, 2H), 6.67 (d, *J* = 16.0 Hz, 1H), 2.40 (s, 3H), 2.04 (s, 3H), 1.97 (s, 3H).

^{13}C NMR (101 MHz, CDCl$_3$) δ (ppm**)** 198.34, 191.79, 144.50, 141.13, 139.01, 138.32, 135.99, 134.59, 133.68, 131.73, 130.48, 128.38, 128.15, 127.95, 127.84, 126.36, 27.86, 21.40, 17.38.
IR (thin film, cm^{-1}): 1043.32, 1113, 1195, 1253, 1360, 1448, 1597, 1695, 2746, 2923, 2987.
HRMS (ESI): [M+Na$^+$] calcd for $C_{19}H_{18}NaO_2$: 01.1199; found 301.1198.
TLC: R$_f$ = 0.5 (90:10 petroleum ether:EtOAc).

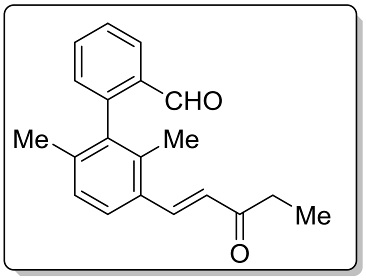

(E)-2',6'-dimethyl-3'-(3-oxopent-1-en-1-yl)-[1,1'-biphenyl]-2-carbaldehyde: Compound **28** was prepared by general procedure D (0.2 mmol scale).
Eluent: petroleum ether/ethyl acetate (97/3, v/v).
Physical State: colorless oil.
Yield: 61% (36 mg isolated; *m*:others = 7:1).
^{1}H NMR (400 MHz, CDCl$_3$) δ (ppm) 9.65 (s, 1H), 8.05 (d, *J* = 7.8 Hz, 1H), 7.89 (d, *J* = 15.9 Hz, 1H), 7.70 (td, *J* = 7.5, 1.3 Hz, 1H), 7.56 (d, *J* = 7.8 Hz, 1H), 7.53 (t, *J* = 7.5 Hz, 1H), 7.19 (d, *J* = 4.7 Hz, 1H), 7.17 (d, *J* = 5.3 Hz, 1H), 6.69 (d, *J* = 15.9 Hz, 1H), 2.71 (q, *J* = 7.3 Hz, 2H), 2.05 (s, 3H), 1.97 (s, 3H), 1.18 (t, *J* = 7.3 Hz, 3H).
^{13}C NMR (126 MHz, CDCl$_3$) δ (ppm) 201.03, 192.08, 145.40, 140.00, 138.66, 135.38, 134.37, 133.93, 133.85, 132.92, 132.80, 130.82, 128.03, 128.00, 127.32, 127.17, 34.46, 20.91, 16.61, 8.23.
IR (thin film, cm^{-1}): 1046, 1122, 1195, 1265, 1392, 1456, 1598, 1695, 2745, 2855, 2926.
HRMS (ESI): [M+Na$^+$] calcd for $C_{20}H_{20}NaO_2$: 315.1356; found 315.1354.
TLC: R$_f$ = 0.5 (90:10 petroleum ether:EtOAc).

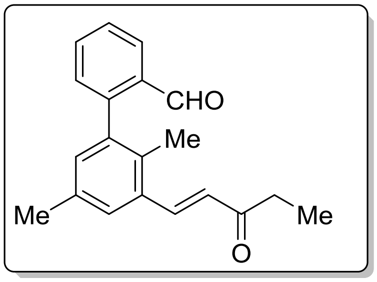

(E)-2',5'-dimethyl-3'-(3-oxopent-1-en-1-yl)-[1,1'-biphenyl]-2-carbaldehyde: Compound **29** was prepared by general procedure D (0.2 mmol scale).
Eluent: petroleum ether/ethyl acetate (97/3, v/v).
Physical State: colorless oil.
Yield: 62% (36 mg isolated; *m*:others = 7:1).
^{1}H NMR (400 MHz, CDCl$_3$) δ (ppm) 9.75 (s, 1H), 8.02 (d, *J* = 7.6 Hz, 1H), 7.89 (d, *J* = 15.6 Hz, H), 7.65 (t, *J* = 7.4 Hz, 1H), 7.52 (t, *J* = 5.9 Hz, 1H), 7.45 (s, 1H), 7.28 (d, *J* = 9.1 Hz, 1H), 7.06

(s, 1H), 6.71 (d, J = 15.6 Hz, 1H), 2.72 (q, J = 7.3 Hz, 2H), 2.35 (s, 3H), 2.10 (s, 3H), 1.19 (t, J = 7.6 Hz, 3H).
^{13}C NMR (101 MHz, CDCl$_3$) δ (ppm) 200.90, 191.83, 144.58, 140.00, 138.82, 138.27, 136.06, 134.58, 133.68, 131.93, 130.48, 128.12, 127.87, 127.79, 127.29, 126.28, 34.40, 21.39, 17.40, 8.26.
IR (thin film, cm^{-1}): 1036, 1121, 1194, 1258, 1356, 1449, 1608, 1667, 1695, 2745, 2853, 2938, 2977.
HRMS (ESI): [M+Na$^+$] calcd for $C_{20}H_{20}NaO_2$: 315.1356; found 315.1354.
TLC: R$_f$ = 0.4 (90:10 petroleum ether:EtOAc).

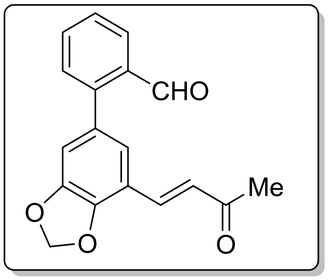

(E)-2-(7-(3-oxobut-1-en-1-yl)benzo[d][1,3]dioxol-5-yl)benzaldehyde: Compound **30** was prepared by general procedure D (0.2 mmol scale).
Eluent: petroleum ether/ethyl acetate (956/4, v/v).
Physical State: Colorless oil.
Yield: 62% (36 mg isolated; *m*:others = 7:1).
^{1}H NMR (400 MHz, CDCl$_3$) δ (ppm) 10.00 (s, 1H), 8.01 (dd, J = 7.8, 1.2 Hz, 1H), 7.63 (td, J = 7.5, 1.4 Hz, 1H), 7.53 – 7.48 (m, 1H), 7.46 (d, J = 16.4 Hz, 1H), 7.41 (dd, J = 7.7, 0.7 Hz, 1H), 6.96 – 6.88 (m, 3H), 6.17 (s, 2H), 2.38 (s, 3H).
^{13}C NMR (101 MHz, CDCl$_3$) δ (ppm) 198.43, 192.04, 148.46, 146.85, 144.65, 136.91, 133.85, 133.71, 132.06, 130.65, 130.14, 128.03, 127.94, 124.37, 116.97, 111.39, 102.25, 27.77.
IR (thin film, cm^{-1}): 1040, 1059, 1197, 1229, 1258, 1363, 1427, 1464, 1595, 1690, 2924, 3064.
HRMS (ESI): [M+Na$^+$] calcd for $C_{18}H_{14}NaO_4$: 317.0784; found 317.0789.
TLC: R$_f$ = 0.4 (90:10 petroleum ether:EtOAc).

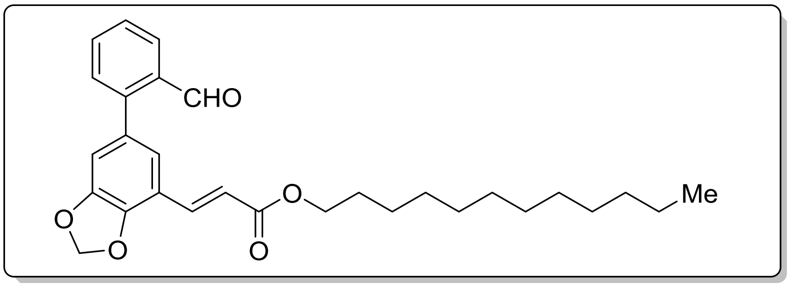

Dodecyl (E)-3-(6-(2-formylphenyl)benzo[d][1,3]dioxol-4-yl)acrylate: Compound **31** was prepared by general procedure D (0.2 mmol scale).
Eluent: petroleum ether/ethyl acetate (95/5, v/v).
Physical State: Yellow oil.
Yield: 68% (63 mg isolated; *m*:others = 10:1).

^{1}H NMR (400 MHz, $CDCl_3$) δ (ppm) 10.01 (s, 1H), 8.01 (dd, *J* = 7.8, 1.2 Hz, 1H), 7.66 – 7.58 (m, 2H), 7.49 (dd, *J* = 9.7, 5.4 Hz, 1H), 7.40 (dd, *J* = 7.7, 0.8 Hz, 1H), 6.88 (dd, *J* = 12.9, 1.6 Hz, 2H), 6.68 (d, *J* = 16.1 Hz, 1H), 6.16 (s, 2H), 4.19 (t, *J* = 6.7 Hz, 2H), 1.74 – 1.61 (m, 2H), 1.43 – 1.36 (m, 2H), 1.28 – 1.24 (m, 16H), 0.87 (t, *J* = 6.8 Hz, 3H).
^{13}C NMR (126 MHz, $CDCl_3$) δ (ppm) 192.24, 167.23, 148.62, 146.89, 144.95, 138.48, 134.07, 133.87, 132.13, 130.86, 128.17, 128.09, 124.66, 122.23, 117.26, 111.39, 102.41, 65.07, 32.12, 29.86, 29.84, 29.80, 29.74, 29.55, 29.50, 28.93, 26.18, 22.89, 14.32.
IR (thin film, cm^{-1}): 1078, 1168, 1228, 1276, 1302, 1361, 1391, 1426, 1465, 1597, 1638, 1694, 2750, 2854, 2924, 3064.
HRMS (ESI): [$M+K^+$] calcd for $C_{29}H_{36}KO_5$: 503.2192; found, 503.2194.
TLC: R_f = 0.4 (90:10 petroleum ether:EtOAc).

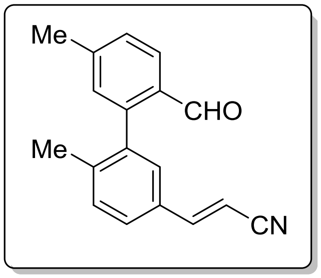

***(E)-3-(2'-formyl-5',6-dimethyl-[1,1'-biphenyl]-3-yl)acrylonitrile*:** Compound **32** was prepared by general procedure D (0.2 mmol scale).
Eluent: petroleum ether/ethyl acetate (96/4, v/v).
Physical State: Colorless oil.
Yield: 49% (26 mg isolated; *m*:others = 9:1).
^{1}H NMR (400 MHz, $CDCl_3$) δ (ppm) 9.90 (s, 1H), 7.94 (d, *J* = 8.0 Hz, 1H), 7.74 (d, *J* = 16.6 Hz, 1H), 7.45 (s, 1H), 7.32 (d, *J* = 0.9 Hz, 2H), 7.27 (d, *J* = 0.7 Hz, 1H), 7.20 (s, 1H), 5.84 (d, *J* = 16.6 Hz, 1H), 2.48 (s, 3H), 2.47 (s, 3H).
^{13}C NMR (101 MHz, $CDCl_3$) δ (ppm) 191.70, 147.92, 144.83, 140.42, 137.06, 136.58, 132.72, 132.38, 131.45, 131.27, 131.07, 129.10, 128.26, 127.66, 126.81, 98.23, 21.72, 19.42.
IR (thin film, cm^{-1}): 1034, 1112, 1210, 1257, 1395, 1466, 1603, 1687, 2218, 2757, 2854, 2922.
HRMS (ESI): [$M+Na^+$] calcd for $C_{18}H_{15}NNaO$: 284.1045; found 284.1047.
TLC: R_f = 0.4 (90:10 petroleum ether:EtOAc).

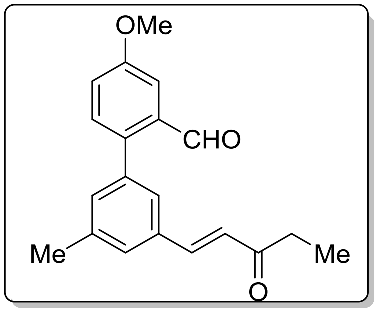

***(E)-4-methoxy-3'-methyl-5'-(3-oxopent-1-en-1-yl)-[1,1'-biphenyl]-2-carbaldehyde*:** Compound **33** was prepared by general procedure D (0.2 mmol scale).
Eluent: petroleum ether/ethyl acetate (95/5, v/v).
Physical State: Colorless oil.
Yield: 68% (42 mg isolated; *m*:others = 12:1).
^{1}H NMR (400 MHz, $CDCl_3$) δ (ppm) 9.94 (s, 1H), 7.57 (d, *J* = 16.2 Hz, 1H), 7.52 (d, *J* = 2.8 Hz, 1H), 7.41 (s, 1H), 7.36 (d, *J* = 8.5 Hz, 1H), 7.32 (s, 1H), 7.21 (dd, *J* = 8.5, 2.8 Hz, 1H), 7.18 (s, 1H), 6.77 (d, *J* = 16.2 Hz, 1H), 3.91 (s, 3H), 2.70 (q, *J* = 7.3 Hz, 2H), 2.43 (s, 3H), 1.17 (t, *J* = 7.3 Hz, 3H).
^{13}C NMR (126 MHz, $CDCl_3$) δ (ppm) 200.82, 192.08, 159.38, 141.65, 138.87, 138.33, 138.27, 134.80, 134.55, 132.94, 132.00, 128.30, 127.11, 126.58, 121.51, 110.04, 55.68, 34.27, 21.35, 8.20.
IR (thin film, cm^{-1}): 1035, 1119, 1164, 1189, 1229, 1276, 1308, 1393, 1462, 1499, 1607, 1688, 2853, 2936, 2972, 3570, 3855.
HRMS (ESI): [$M+H^+$] calcd for $C_{20}H_{21}O_3$: 309.1485; found 309.1483.
TLC: R_f = 0.4 (90:10 petroleum ether:EtOAc).

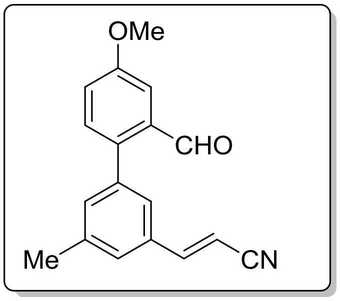

(E)-3-(2'-formyl-4'-methoxy-5-methyl-[1,1'-biphenyl]-3-yl)acrylonitrile: Compound **34** was prepared by general procedure D (0.2 mmol scale).
Eluent: petroleum ether/ethyl acetate (95/5, v/v).
Physical State: Colorless oil.
Yield: 70% (39 mg isolated; *m*:others = 15:1).
^{1}H NMR (400 MHz, $CDCl_3$) δ (ppm) 9.91 (s, 1H), 7.52 (d, *J* = 2.8 Hz, 1H), 7.41 (d, *J* = 16.7 Hz, 1H), 7.34 (d, *J* = 8.5 Hz, 1H), 7.31 (s, 1H), 7.22 (s, 3H), 5.92 (d, *J* = 16.6 Hz, 1H), 3.91 (s, 3H), 2.44 (s, 3H).
^{13}C NMR (101 MHz, $CDCl_3$) δ (ppm) 191.78, 159.53, 150.14, 139.17, 138.62, 137.76, 134.55, 133.75, 133.71, 131.95, 127.32, 126.18, 121.50, 117.98, 110.25, 97.14, 55.69, 21.32.
IR (thin film, cm^{-1}): 1033, 1075, 1163, 1230, 1278, 1310, 1395, 1464, 1499, 1604, 1686, 2218, 2761, 2851, 2927, 2954.
HRMS (ESI): [$M+Na^+$] calcd for $C_{18}H_{15}NNaO_2$: 300 0994; found 300.0995.
TLC: R_f = 0.5 (90:10 petroleum ether:EtOAc).

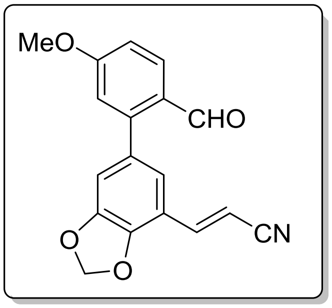

***(E)-3-(6-(2-formyl-5-methoxyphenyl)benzo[d][1,3]dioxol-4-yl)acrylonitrile*:** Compound **35** was prepared by general procedure D (0.2 mmol scale).
Eluent: petroleum ether/ethyl acetate (95/5, v/v).
Physical State: colorless oil.
Yield: 63% (39 mg isolated; *m*:others = 6:1).
^{1}H NMR (400 MHz, $CDCl_3$) δ (ppm) 9.83 (s, 1H), 8.00 (d, *J* = 8.7 Hz, 1H), 7.33 – 7.27 (m, 1H), 7.01 (dd, *J* = 8.5, 2.3 Hz, 1H), 6.90 (t, *J* = 2.5 Hz, 1H), 6.83 – 6.79 (m, 2H), 6.21 (d, *J* = 16.7 Hz, 1H), 6.18 (s 2H), 3.91 (s, 3H).
^{13}C NMR (126 MHz, $CDCl_3$) δ (ppm) 190.41, 163.65, 148.46, 146.69, 146.61, 144.73, 132.37, 130.64, 127.41, 123.64, 118.08, 116.15, 115.41, 114.07, 111.97, 102.52, 100.29, 55.71.
IR (thin film, cm^{-1}): 1028, 1117, 1241, 1295, 1444, 1478, 1595, 1679, 2217, 2853, 2925.
HRMS (ESI): [M+H$^+$] calcd for $C_{18}H_{14}NO_4$: 308.0917; found 308.0921.
TLC: R_f = 0.4 (90:10 petroleum ether:EtOAc).

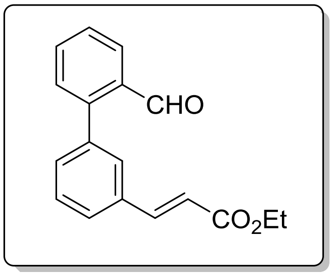

***Ethyl (E)-3-(2'-formyl-[1,1'-biphenyl]-3-yl)acrylate*:** Compound **36** was prepared by general procedure D (0.2 mmol scale).
Eluent: petroleum ether/ethyl acetate (98/2, v/v).
Physical State: colorless oil.
Yield: 71% (39 mg isolated; *m*:others = 13:1).
^{1}H NMR (500 MHz, $CDCl_3$) δ (ppm) 9.98 (s, 1H), 8.04 (d, *J* = 7.8 Hz, 1H), 7.73 (d, *J* = 16.0 Hz, 1H), 7.66 (t, *J* = 7.5 Hz, 1H), 7.61 (d, *J* = 7.8 Hz, 1H), 7.54 (d, *J* = 6.6 Hz, 2H), 7.50 (t, *J* = 7.8 Hz, 1H), 7.44 (d, *J* = 7.6 Hz, 1H), 7.39 (d, *J* = 7.6 Hz, 1H), 6.49 (d, *J* = 16.0 Hz, 1H), 4.27 (q, *J* = 7.1 Hz, 2H), 1.34 (t, *J* = 7.1 Hz, 3H).
^{13}C NMR (126 MHz, $CDCl_3$) δ (ppm) 192.22, 166.96, 145.23, 143.97, 138.81, 135.05, 133.95, 133.93, 131.97, 130.93, 129.62, 129.21, 128.41, 128.09, 127.85, 119.65, 60.87, 14.51.
IR (thin film, cm^{-1}): 1036, 1100, 1179, 1262, 1367, 1467, 1597, 1639, 2856, 2926.
HRMS (ESI): [M+Na$^+$] calcd for $C_{18}H_{16}NaO_3$: 303.0997; found: 303.0993.
TLC: R_f = 0.5 (95:5 petroleum ether:EtOAc).

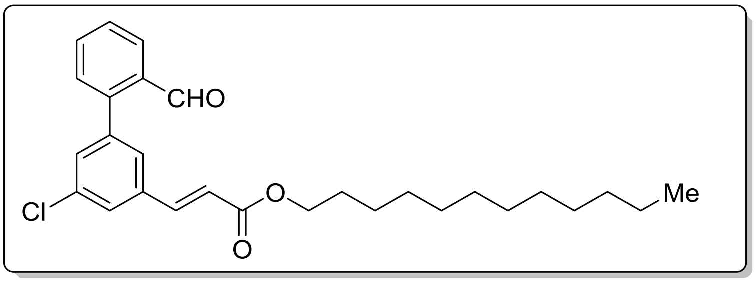

Ethyl (E)-3-(5-chloro-2'-formyl-[1,1'-biphenyl]-3-yl)acrylate**:** Compound **37** was prepared by general procedure D (0.2 mmol scale).
Eluent: petroleum ether/ethyl acetate (96/4, v/v).
Physical State: colorless oil.
Yield: 63% (57 mg isolated; *m*:others = 10:1).
^{1}H NMR (400 MHz, CDCl$_3$) δ (ppm) 9.98 (s, 1H), 8.05 (dd, *J* = 7.8, 1.1 Hz, 1H), 7.67 (ddd, *J* = 7.5, 5.9, 1.4 Hz, 2H), 7.60 (d, *J* = 13.8 Hz, 1H), 7.58 – 7.55 (m, 1H), 7.43 – 7.38 (m, 3H), 6.49 (d, *J* = 16.0 Hz, 1H), 4.20 (t, *J* = 6.7 Hz, 2H), 1.73 – 1.66 (m, 2H), 1.42 – 1.34 (m, 2H), 1.27 – 1.24 (m, 16H), 0.87 (t, *J* = 6.8 Hz, 3H).
^{13}C NMR (126 MHz, CDCl$_3$) δ (ppm) 191.62, 166.63, 143.66, 142.41, 140.51, 136.61, 135.34, 134.06, 133.86, 131.34, 130.83, 128.92, 128.45, 127.95, 127.51, 121.09, 65.26, 32.13, 29.86, 29.84, 29.80, 29.75, 29.56, 29.49, 28.89, 26.18, 22.90, 14.33.
IR (thin film, cm^{-1}): 3503, 2925, 2854, 2751, 1711, 1639, 1598, 1465, 1390, 1321, 1275, 1176, 981.
HRMS (ESI): [M+H$^+$] calcd for $C_{28}H_{36}ClO_3$: 454.2282; found 454.2285.
TLC: R$_f$ = 0.4 (90:10 petroleum ether:EtOAc).

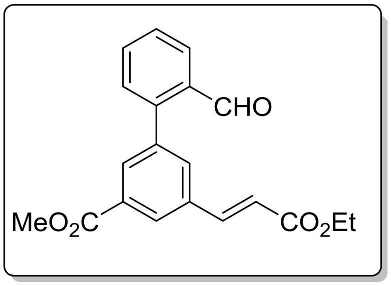

Methyl (E)-5-(3-ethoxy-3-oxoprop-1-en-1-yl)-2'-formyl-[1,1'-biphenyl]-3-carboxylate**:** Compound **38** was prepared by general procedure D (0.2 mmol scale).
Eluent: petroleum ether/ethyl acetate (96/4, v/v).
Physical State: colorless oil.
Yield: 53% (36 mg isolated; *m*:others = 5:1)
^{1}H NMR (500 MHz, CDCl$_3$) δ (ppm) 9.96 (s, 1H), 8.28 (s, 1H), 8.10 – 8.05 (m, 2H), 7.75 (d, *J* = 16.1 Hz, 1H), 7.71 – 7.66 (m, 2H), 7.57 (t, *J* = 7.5 Hz, 1H), 7.44 (d, *J* = 7.6 Hz, 1H), 6.57 (d, *J* = 16.1 Hz, 1H), 4.28 (q, *J* = 7.1 Hz, 2H), 3.96 (s, 3H), 1.34 (t, *J* = 7.1 Hz, 3H).

^{13}C NMR (101 MHz, CDCl$_3$) δ (ppm) 191.64, 166.63, 166.27, 144.04, 142.83, 139.32, 135.44, 134.09, 133.88, 133.54, 132.32, 131.44, 131.02, 128.86, 128.64, 128.59, 120.89, 61.00, 52.78, 14.49.
IR (thin film, cm^{-1}): 1924, 1775, 1667, 1450, 1434, 1392, 1352, 1345, 1341, 1325, 1312, 1296, 1298, 1289, 1287, 1280, 1198.
HRMS (ESI): [M+H$^+$] calcd for $C_{20}H_{19}O_5$: 339.1227; found 339.1229.
TLC: R$_f$ = 0.4 (90:10 petroleum ether:EtOAc).

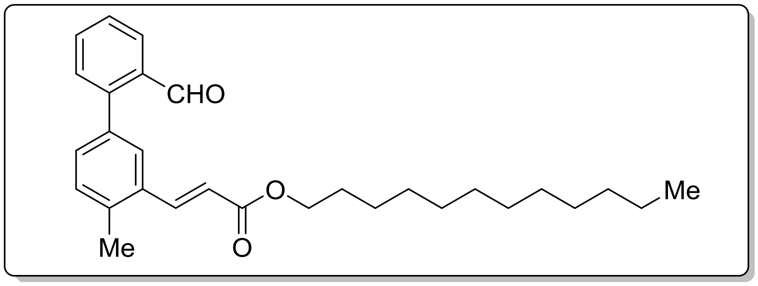

***Dodecyl (E)-3-(2'-formyl-4-methyl-[1,1'-biphenyl]-3-yl)acrylate*:** Compound **39** was prepared by general procedure D (0.2 mmol scale).
Eluent: petroleum ether/ethyl acetate (97/3, v/v).
Physical State: yellow oil.
Yield: 62% (54 mg isolated; *m*:others = 6:1).
^{1}H NMR (400 MHz, CDCl$_3$) δ (ppm) 9.99 (s, 1H), 8.04 – 7.98 (m, 2H), 7.65 (td, *J* = 7.5, 1.4 Hz, 1H), 7.56 (s, 1H), 7.51 (t, *J* = 7.6 Hz, 1H), 7.44 (d, *J* = 7.9 Hz, 1H), 7.29 (dd, *J* = 6.5, 4.8 Hz, 2H), 6.39 (d, *J* = 15.9 Hz, 1H), 2.51 (s, 3H), 1.72 – 1.65 (m, 2H), 1.42 – 1.36 (m, 2H), 1.27 – 1.24 (m, 16H), 0.89 – 0.87 (m, 3H).
^{13}C NMR (126 MHz, CDCl$_3$) δ (ppm) 192.40, 167.13, 163.09, 145.39, 141.79, 137.81, 136.17, 133.96, 133.89, 131.62, 131.13, 130.94, 130.25, 129.37, 128.17, 128.07, 127.76, 120.57, 65.07, 32.13, 29.86, 29.84, 29.80, 29.76, 29.56, 29.50, 28.93, 26.20, 22.90, 19.78, 14.33.
IR (thin film, cm^{-1}): 1045, 1174, 1215, 1251, 1376, 1467, 1597, 1637, 1697, 1716, 2855, 2926, 3022.
HRMS (ESI): [M+ Na$^+$] calcd for $C_{29}H_{38}NaO_3$: 457.2716; found, 457.2713.
TLC: R$_f$ = 0.6 (90:10 petroleum ether:EtOAc).

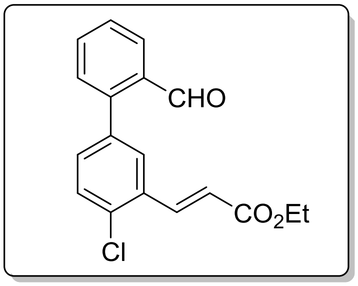

Ethyl (E)-3-(4-chloro-2'-formyl-[1,1'-biphenyl]-3-yl)acrylate: Compound **40** was prepared by general procedure D (0.2 mmol scale).
Eluent: petroleum ether/ethyl acetate (97/3, v/v).

Physical State: yellow oil.
Yield: 68% (43 mg isolated; *m*:others > 20:1).
^{1}H NMR (400 MHz, $CDCl_3$) δ (ppm) 9.75 (s, 1H), 8.12 (d, *J* = 16.0 Hz, 1H), 7.67 (d, *J* = 7.5 Hz, 1H), 7.62 (d, *J* = 8.2 Hz, 1H), 7.55 (t, *J* = 7.2 Hz, 1H), 7.5-7.52 (m. 2H), (7.42 (d, *J* = 7.6 Hz, 1H), 7.32 (dd, *J* = 8.2, 2.1 Hz, 1H), 6.46 (d, *J* = 16.0 Hz, 1H), 4.28 (q, *J* = 7.1 Hz, 2H), 1.34 (t, *J* = 7.1 Hz, 3H).
^{13}C NMR (126 MHz, $CDCl_3$) δ (ppm) 191.83, 166.48, 144.02, 139.99, 137.27, 135.19, 134.08, 133.90, 133.23, 132.48, 130.90, 130.44, 129.13, 128.75, 128.52, 122.14, 61.06, 14.52.
IR (thin film, cm^{-1}): 2926, 2854, 2753, 1715, 1639, 1598, 1465, 1392, 1367, 1274, 1094, 1043, 980.
HRMS (ESI): [M+Na$^+$] calcd for $C_{18}H_{16}ClNaO_3$: 315.0782; found, 315.0781.
TLC: R_f = 0.6 (90:10 petroleum ether:EtOAc).

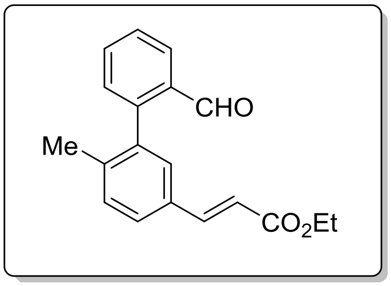

***Ethyl (E)-3-(2'-formyl-6-methyl-[1,1'-biphenyl]-3-yl)acrylate*:** Compound **41** was prepared by general procedure D (0.2 mmol scale).
Eluent: petroleum ether/ethyl acetate (97/3, v/v).
Physical State: yellow oil.
Yield: 65% (38 mg isolated; *m*:others = 3:1)
^{1}H NMR (400 MHz, $CDCl_3$) δ (ppm) 9.76 (s, 1H), 8.06 – 8.01 (m, 2H), 7.65 (t, *J* = 4.5 Hz, 2H), 7.51 (dd, *J* = 6.9, 5.0 Hz, 2H), 7.37 (d, *J* = 1.7 Hz, 1H), 7.34 – 7.30 (m, 2H), 6.42 (d, *J* = 16.0 Hz, 1H), 4.25 (q, *J* = 7.1 Hz, 2H), 2.11 (s, 3H), 1.32 (t, *J* = 7.1 Hz, 3H).
^{13}C NMR (101 MHz, $CDCl_3$) δ (ppm) 191.86, 166.93, 144.61, 143.80, 142.38, 138.80, 138.31, 133.94, 130.72, 130.66, 129.64, 128.22, 127.80, 127.50, 120.80, 118.28, 60.51, 20.36, 14.31.
IR (thin film, cm^{-1}): 1036, 1096, 1174, 1265, 1318, 1366, 1392, 1448, 1597, 1637, 1711, 2750, 2843, 2936, 2984.
HRMS (*m/z*): [M+Na$^+$] calcd for $C_{19}H_{18}NaO_3$: 317.1148; found, 317.1148.
TLC: R_f = 0.6 (90:10 petroleum ether:EtOAc).

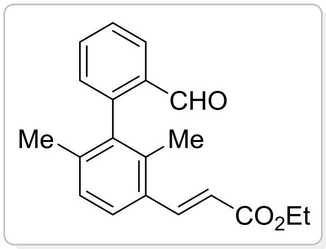

Ethyl (E)-3-(2'-formyl-2,6-dimethyl-[1,1'-biphenyl]-3-yl)acrylate: Compound **42** was prepared by general procedure D (0.2 mmol scale).

Eluent: petroleum ether/ethyl acetate (93/7, v/v).
Physical State: Yellow oil.
Yield: 66% (43 mg isolated; *m*:others = 8:1).
^{1}H NMR (400 MHz, CDCl$_3$) δ (ppm) 9.65 (s, 1H), 8.05 (dd, *J* = 7.8, 1.1 Hz, 1H), 8.00 (d, *J* = 15.8 Hz, 1H), 7.69 (td, *J* = 7.5, 1.4 Hz, 1H), 7.56 – 7.51 (m, 2H), 7.18 (t, *J* = 7.8 Hz, 2H), 6.38 (d, *J* = 15.8 Hz, 1H), 4.27 (q, *J* = 7.1, Hz, 2H), 2.04 (s, 3H), 1.96 (s, 3H), 1.34 (t, *J* = 7.1 Hz, 3H).
^{13}C NMR (101 MHz, CDCl$_3$) δ (ppm) 191.81, 167.03, 144.63, 142.50, 138.65, 138.17, 135.76, 134.54, 133.70, 131.83, 130.49, 128.08, 127.83, 127.72, 126.35, 119.63, 60.52, 21.36, 17.41, 14.35.
IR (thin film, cm^{-1}): 982, 1038, 1095, 1163, 1253, 1311, 1366, 1390, 1447, 1597, 1632, 1697, 2744, 2852, 2926, 2980.
HRMS (*m/z*): [M+Na$^+$] calcd for $C_{20}H_{20}NaO_3$: 331.1305; found, 331.1302.
TLC: R$_f$ = 0.5 (90:10 petroleum ether:EtOAc).

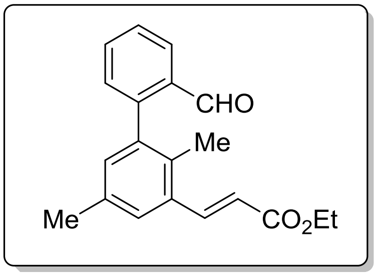

***Ethyl (E)-3-(2'-formyl-2,5-dimethyl-[1,1'-biphenyl]-3-yl)acrylate*:** Compound **43** was prepared by general procedure D (0.2 mmol scale).
Eluent: petroleum ether/ethyl acetate (97/3, v/v).
Physical State: colorless oil.
Yield: 51% (31 mg isolated; *m*:others = 6:1).
^{1}H NMR (500 MHz, CDCl$_3$) δ (ppm) 9.79 (s, 1H), 8.09 – 7.98 (m, 2H), 7.67 (td, *J* = 7.5, 1.4 Hz, 1H), 7.54 (t, *J* = 7.6 Hz, 1H), 7.46 (s, 1H), 7.31 (d, *J* = 7.6 Hz, 1H), 7.06 (s, 1H), 6.44 (d, *J* = 15.8, 1H), 4.31 (q, *J* = 7.1 Hz, 2H), 2.37 (s, 3H), 2.12 (s, 3H), 1.38 (t, *J* = 7.1, 3H).
^{13}C NMR (126 MHz, CDCl$_3$) δ (ppm) 192.05, 166.91, 145.41, 142.54, 138.58, 135.34, 134.26, 133.95, 133.80, 132.67, 130.82, 127.99, 127.28, 120.46, 60.58, 20.88, 16.60, 14.34.
IR (thin film, cm^{-1}): 1039, 1095, 1177, 1262, 1301, 1367, 1391, 1456, 1598, 1635, 1698, 2748, 2854, 2925.
HRMS (*m/z*): [M+Na$^+$] calcd for $C_{20}H_{20}NaO_3$: 331.1305; found, 331.1305.
TLC: R$_f$ = 0.5 (90:10 petroleum ether:EtOAc).

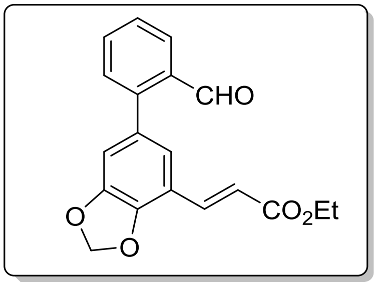

***Ethyl (E)-3-(6-(2-formylphenyl)benzo[d][1,3]dioxol-4-yl)acrylate*:** Compound **44** was prepared by general procedure D (0.2 mmol scale).
Eluent: petroleum ether/ethyl acetate (65/4, v/v).
Physical State: Yellow oil.
Yield: 71% (46 mg isolated; *m*:others = 4:1).
^{1}H NMR (500 MHz, $CDCl_3$) δ (ppm) 10.03 (s, 1H), 8.03 (dd, *J* = 7.8, 1.3 Hz, 1H), 7.67 – 7.65 (m, 1H), 7.64 – 7.62 (m, 1H), 7.52 (t, *J* = 7.1 Hz, 1H), 7.43 (d, *J* = 7.7 Hz, 1H), 6.92 (d, *J* = 1.6 Hz, 1H), 6.89 (d, *J* = 1.7 Hz, 1H), 6.71 (d, *J* = 16.1 Hz, 1H), 6.19 (s, 2H), 4.29 (q, *J* = 7.1 Hz, 2H), 1.37 (t, *J* = 7.1 Hz, 3H).
^{13}C NMR (126 MHz, $CDCl_3$) δ (ppm) 192.0, 166.9, 148.4, 146.6, 144.7, 138.3, 133.8, 133.6, 131.9, 130.6, 127.9, 127.8, 124.4, 122.0, 117.0, 111.2, 102.2, 60.6, 14.3.
IR (thin film, cm^{-1}): 1078, 1228, 1276, 1302, 1391, 1465, 1597, 1638, 1694, 2750, 2854, 2924, 3065.
HRMS (ESI): [M+Na$^+$] calcd for $C_{19}H_{16}NaO_5$: 347.0897; found, 347.0892.
TLC: R_f = 0.5 (90:10 petroleum ether:EtOAc).

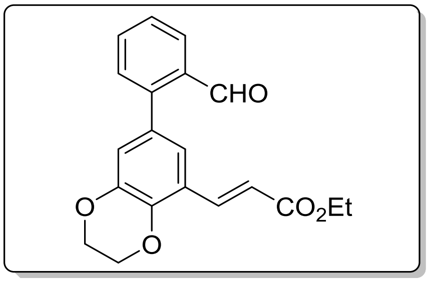

***Ethyl (E)-3-(7-(2-formylphenyl)-2,3-dihydrobenzo[b][1,4]dioxin-5-yl)acrylate*:** Compound **45** was prepared by general procedure D (0.2 mmol scale).
Eluent: petroleum ether/ethyl acetate (96/4, v/v).
Physical State: Colorless oil.
Yield: 67% (45 mg isolated; *m*:others = 15:1).
^{1}H NMR (500 MHz, $CDCl_3$) δ (ppm) 10.04 (s, 1H), 8.03 (dd, *J* = 7.8, 1.1 Hz, 1H), 7.94 (d, *J* = 16.2 Hz, 1H), 7.65 (td, *J* = 7.5, 1.4 Hz, 1H), 7.51 (t, *J* = 7.6 Hz, 1H), 7.44 (d, *J* = 7.54 Hz, 1H), 7.10 (d, *J* = 2.0 Hz, 1H), 6.96 (d, *J* = 2.1 Hz, 1H), 6.58 (d, *J* = 16.2 Hz, 1H), 4.46 – 4.42 (m, 2H), 4.37 – 4.35 (m, 2H), 4.28 (t, *J* = 7.1 Hz, 2H), 1.35 (t, *J* = 7.1 Hz, 3H).
^{13}C NMR (101 MHz, $CDCl_3$) δ (ppm) 192.26, 167.14, 144.76, 143.77, 142.87, 138.53, 133.73, 133.65, 130.63, 127.85, 127.74, 123.68, 122.67, 120.34, 64.59, 64.07, 60.53, 14.34.
IR (thin film, cm^{-1}): 1072, 1226, 1251, 1314, 1395, 1425, 1567, 1619, 1699, 2751, 2847, 2922, 3065.
HRMS (ESI): [M+H$^+$] calcd for $C_{20}H_{19}O_5$: 338.1159; found 338.1163.
TLC: R_f = 0.5 (90:10 petroleum ether:EtOAc).

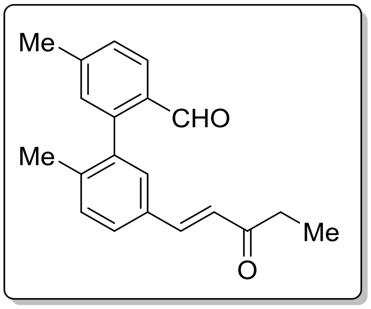

***(E)-2',5-dimethyl-5'-(3-oxopent-1-en-1-yl)-[1,1'-biphenyl]-2-carbaldehyde*:** Compound **46** was prepared by general procedure D (0.2 mmol scale).
Eluent: petroleum ether/ethyl acetate (96/4, v/v).
Physical State: Colorless oil.
Yield: 69% (41 mg isolated; *m*:others = 5:1).
^{1}H NMR (500 MHz, $CDCl_3$) δ (ppm) 9.96 (s, 1H), 7.97 (d, *J* = 8.0 Hz, 1H), 7.93 (d, *J* = 15.9 Hz, 1H), 7.59 (s, 1H), 7.35 (d, *J* = 6.8 Hz, 1H), 7.32 (d, *J* = 4.7 Hz, 1H), 7.29 (d, *J* = 1.9 Hz, 1H), 7.26 (s, 1H), 6.72 (d, *J* = 16.0 Hz, 1H), 2.72 (q, *J* = 7.3 Hz, 2H), 2.53 (s, 3H), 2.50 (s, 3H), 1.20 (t, *J* = 7.3 Hz, 3H).
^{13}C NMR (126 MHz, $CDCl_3$) δ (ppm) 200.70, 191.93, 145.31, 144.77, 139.01, 136.16, 133.76, 131.52, 131.32, 130.90, 129.98, 129.09, 128.90, 127.98, 127.66, 127.49, 34.76, 21.87, 19.59, 8.18.
IR (thin film, cm^{-1}): 1039, 1120, 1189, 1257, 1394, 1458, 1603, 1687, 2754, 2856, 2930, 2974, 3028.
HRMS (ESI): [$M+Na^+$] calcd for $C_{20}H_{20}NaO_2$: 315.1356; found 315.1354.
TLC: R_f = 0.4 (90:10 petroleum ether:EtOAc).

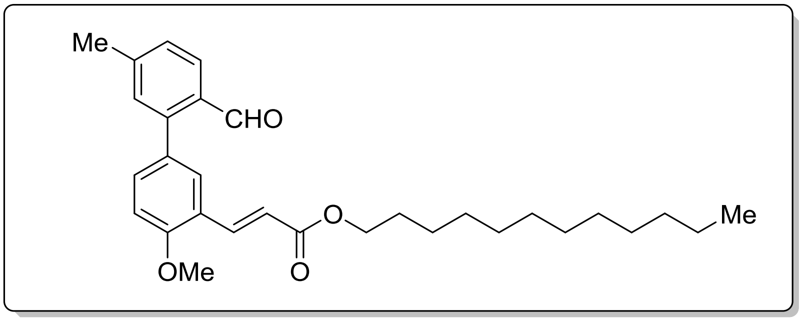

Dodecyl (E)-3-(2'-formyl-4-methoxy-5'-methyl-[1,1'-biphenyl]-3-yl)acrylate: Compound **47** was prepared by general procedure D (0.2 mmol scale).
Eluent: petroleum ether/ethyl acetate (97/3, v/v).
Physical State: Colorless oil.
Yield: 66% (61 mg isolated; *m*:others = 6:1).
^{1}H NMR (400 MHz, $CDCl_3$) δ (ppm) 9.94 (s, 1H), 8.01 (d, *J* = 16.2 Hz, 1H), 7.93 (d, *J* = 8.0 Hz, 1H), 7.53 (d, *J* = 2.2 Hz, 1H), 7.33 (dd, *J* = 8.5, 2.3 Hz, 1H), 7.30 (d, *J* = 7.9 Hz, 1H), 7.22 (s, 1H), 7.00 (d, *J* = 8.5 Hz, 1H), 6.56 (d, *J* = 16.2 Hz, 1H), 4.19 (t, *J* = 6.7 Hz, 2H), 3.96 (s, 3H), 2.46 (s, 3H), 1.73 – 1.65 (m, 2H), 1.43 – 1.36 (m, 2H), 1.28 – 1.24 (m, 16H), 0.87 (t, *J* = 6.8 Hz, 3H).

^{13}C NMR (126 MHz, CDCl$_3$) δ (ppm) 192.14, 167.57, 158.39, 145.23, 144.89, 139.51, 133.13, 131.71, 131.55, 130.57, 130.31, 128.88, 128.21, 123.80, 119.95, 111.24, 64.92, 55.96, 32.12, 29.86, 29.83, 29.80, 29.75, 29.55, 29.51, 28.96, 26.19, 22.89, 22.04, 14.32.
IR (thin film, cm^{-1}): 1115, 1166, 1214, 1251, 1305, 1392, 1464, 1501, 1604, 1633, 1687, 2753, 2854, 2925, 3020.
HRMS (ESI): [M+Na$^+$] calcd for $C_{30}H_{40}NaO_4$: 487.2817; found, 487.2819.
TLC: R$_f$ = 0.7 (90:10 petroleum ether:EtOAc).

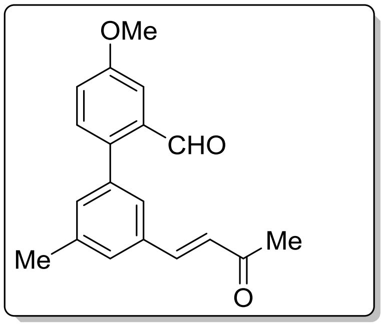

(E)-4-methoxy-3'-methyl-5'-(3-oxobut-1-en-1-yl)-[1,1'-biphenyl]-2-carbaldehyde: Compound **48** was prepared by general procedure D (0.2 mmol scale).
Eluent: petroleum ether/ethyl acetate (95/5, v/v).
Physical State: Colorless oil.
Yield: 70% (41 mg isolated; *m*:others = 10:1).
^{1}H NMR (400 MHz, CDCl$_3$) δ (ppm) 9.93 (s, 1H), 7.53 (d, *J* = 16.3 Hz, 1H), 7.51 (d, *J* = 4.8 Hz, 1H), 7.41 (s, 1H), 7.36 (d, *J* = 8.7 Hz, 1H), 7.32 (s, 1H), 7.22 (d, *J* = 8.7 Hz, 1H), 7.19 (d, *J* = 8.7 Hz, 1H), 6.74 (d, *J* = 16.3 Hz, 1H), 3.91 (s, 3H), 2.44 (s, 3H), 2.39 (s, 3H).
^{13}C NMR (101 MHz, CDCl$_3$) δ (ppm) 198.27, 192.03, 159.40, 142.86, 138.92, 138.32, 138.23, 134.63, 134.55, 133.07, 131.98, 128.23, 127.70, 127.22, 121.49, 110.09, 55.67, 27.68.
IR (thin film, cm^{-1}): 1033, 1111, 1164, 1229, 1276, 1310, 1359, 1394 1438, 1465, 1498, 1605, 1686, 2756, 2853, 2928, 3007.
HRMS (ESI): [M+Na$^+$] calcd for $C_{19}H_{18}NaO_3$: 317.1148; found 317.1149.
TLC: R$_f$ = 0.4 (90:10 petroleum ether:EtOAc).

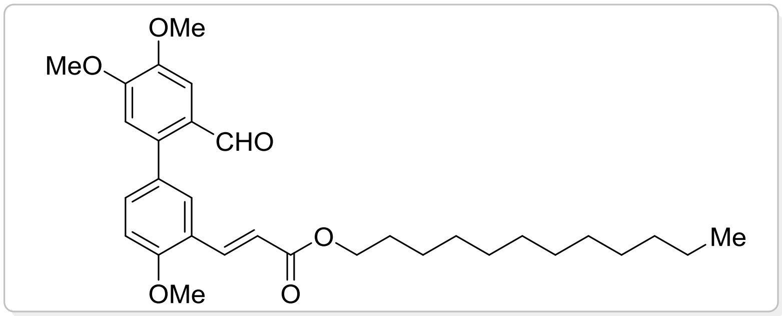

***Ethyl (E)-3-(2'-formyl-4,4',5'-trimethoxy-[1,1'-biphenyl]-3-yl)acrylate*:** Compound **49** was prepared by general procedure D (0.2 mmol scale).

Eluent: petroleum ether/ethyl acetate (95/5, v/v).
Physical State:Ccolorless oil.
Yield: 62% (63 mg isolated; *m*:others > 20:1).
^{1}H NMR (500 MHz, CDCl$_3$) δ (ppm) 9.82 (s, 1H), 8.01 (d, *J* = 16.2 Hz, 1H), 7.52 (d, *J* = 2.2 Hz, 2H), 7.33 (dd, *J* = 8.4, 2.2 Hz, 1H), 7.00 (d, *J* = 8.5 Hz, 1H), 6.82 (s, 1H), 6.57 (d, *J* = 16.2 Hz, 1H), 4.20 (t, *J* = 6.8 Hz, 2H), 3.98 (s, 6H), 3.96 (s, 3H), 1.73 – 1.67 (m, 2H), 1.43 – 1.35 (m, 2H), 1.27 – 1.22 (m, 16H), 0.87 (t, *J* = 6.9 Hz, 3H).
^{13}C NMR (126 MHz, CDCl$_3$) δ (ppm) 191.14, 167.57, 158.39, 153.74, 149.00, 140.68, 139.48, 133.26, 130.46, 130.22, 127.21, 123.78, 120.06, 112.72, 111.22, 108.95, 64.95, 56.49, 56.37, 55.99, 32.12, 29.86, 29.84, 29.80, 29.76, 29.55, 29.51, 28.96, 26.19, 22.89, 14.33.
IR (thin film, cm^{-1}): 1115, 1166, 1214, 1251, 1305, 1392, 1464, 1501 1604, 1633, 1687, 2753, 2854, 2925, 3020.
HRMS (ESI): [M+H$^+$] calcd for $C_{31}H_{43}O_6$: 511.3054; found 511.3054
TLC: R$_f$ = 0.5 (90:10 petroleum ether:EtOAc).

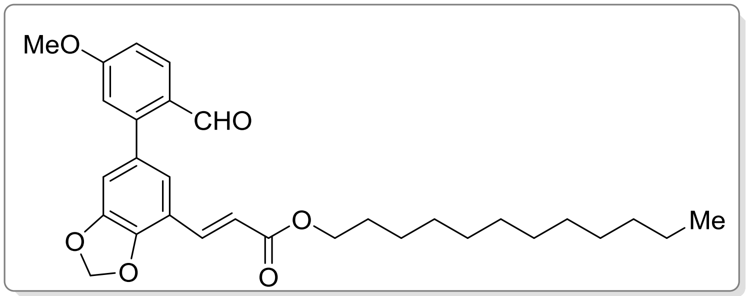

***Dodecyl (E)-3-(6-(2-formyl-5-methoxyphenyl)benzo[d][1,3]dioxol-4-yl)acrylate*:** Compound **50** was prepared by general procedure D (0.2 mmol scale).
Eluent: petroleum ether/ethyl acetate (95/5, v/v).
Physical State: yellow oil.
Yield: 58% (57 mg isolated; *m*:others = 10:1).
^{1}H NMR (500 MHz, CDCl$_3$) δ (ppm) 9.86 (s, 1H), 8.00 (d, *J* = 8.7 Hz, 1H), 7.61 (d, *J* = 16.1 Hz, 1H), 7.00 (dd, *J* = 8.8, 2.3 Hz, 1H), 6.91 (d, *J* = 1.4 Hz, 1H), 6.86 (d, *J* = 1.5 Hz, 1H), 6.83 (d, *J* = 2.5 Hz, 1H), 6.68 (d, *J* = 16.1 Hz, 1H), 6.16 (s, 2H), 4.20 (t, *J* = 6.7 Hz, 2H), 3.91 (s, 3H), 1.72 – 1.66 (m, 2H), 1.41 – 1.37 (m, 2H), 1.26 – 1.23 (m, 16H), 0.87 (t, *J* = 6.9 Hz, 3H).
^{13}C NMR (126 MHz, CDCl$_3$) δ (ppm) 190.84, 167.25, 163.82, 148.52, 147.47, 146.91, 138.49, 132.16, 130.56, 127.70, 124.40, 122.21, 117.21, 115.44, 114.25, 111.33, 102.40, 65.08, 55.87, 32.13, 29.86, 29.84, 29.81, 29.75, 29.56, 29.51, 28.94, 26.19, 22.90, 14.33.
IR (thin film, cm^{-1}): 1030, 1117, 1175, 1240, 1295, 1396, 1444, 1467, 1596, 1637, 1683, 1712, 2854, 2925.
HRMS (ESI): [M+Na$^+$] calcd for $C_{30}H_{38}NaO_6$: 517.2564; found, 517.2561.
TLC: R$_f$ = 0.4 (90:10 petroleum ether:EtOAc).

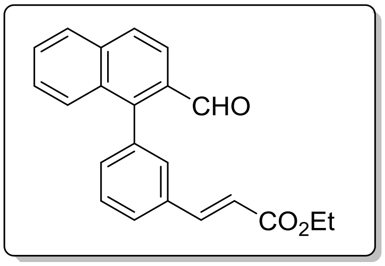

Ethyl (E)-3-(3-(2-formylnaphthalen-1-yl)phenyl)acrylate: Compound **51** was prepared by general procedure D (0.2 mmol scale).
Eluent: petroleum ether/ethyl acetate (98/2, v/v).
Physical State: colorless oil.
Yield: 64% (42 mg isolated; *m*:others = 3:1).
^{1}H NMR (400 MHz, $CDCl_3$) δ (ppm) 9.89 (s, 1H), 8.08 (d, *J* = 8.66 Hz, 1H), 7.96 (t, *J* = 7.51, 2H), 7.75 (d, *J* = 16.0 Hz, 1H), 7.70 (d, *J* = 8.3 Hz, 1H), 7.67 – 7.62 (m, 2H), 7.61 (t, *J* = 4.1 Hz, 1H), 7.56 (d, *J* = 2.9 Hz, 1H), 7.50 – 7.47 (m, 1H), 7.45 (dd, *J* = 9.4, 4.2 Hz, 1H), 6.50 (d, *J* = 16.0 Hz, 1H), 4.26 (q, *J* = 7.15 Hz, 2H), 0.88 (t, *J* = 6.5 Hz, 3H).
^{13}C NMR (126 MHz, $CDCl_3$) δ (ppm) 192.38, 166.76, 145.48, 143.59, 136.13, 136.06, 134.72, 132.68, 132.26, 131.24, 130.38, 128.96, 128.72, 128.36, 127.94, 127.44, 127.13, 122.19, 119.60, 60.66, 14.25.
IR (thin film, cm^{-1}): 1036, 1100, 1179, 1262, 1367, 1467, 1597, 1639, 2856, 2926.
HRMS (ESI): [M+Na^{+}] calcd for $C_{22}H_{18}NaO_3$: 353.1148; found 353.1143.
TLC: R_f = 0.4 (90:10 petroleum ether:EtOAc).

2.10 Optimization details for *meta*-C–H olefination of 2-phenylaniline

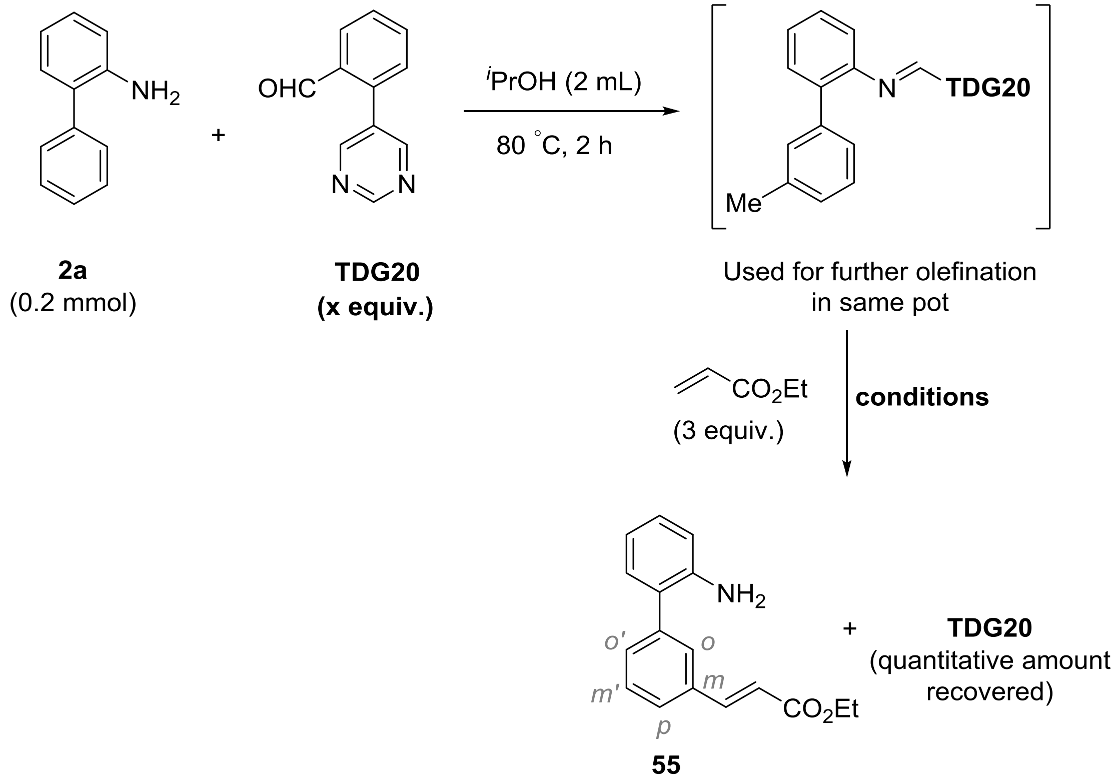

Supplementary Table 13: Optimization of TDG20 amount

Entry	2a (mmol)	TDG20 (mmol)	Yield (%)
1	0.2	0.18	86
2	0.2	0.19	91
3	0.2	0.2	94
4	0.2	0.21	96
**5**	**0.2**	**0.22**	**97**

[a]Yield was measured by ^{1}H NMR of the crude reaction mixture using 1,3,5-trimethoxybenzene.

Supplementary Table 14: Optimization of *meta*-C–H olefination of 2-phenylaniline

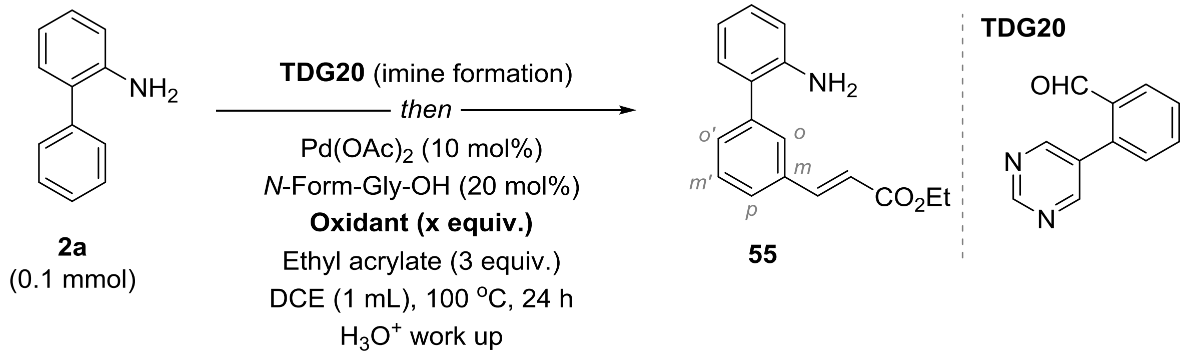

Entry	Oxidant	Yield (*m*:others)[a]
1	Ag_2CO_3 (25 mol%) + $Cu(OAc)_2$ (3.5 equiv.)	31% (5:1)[b]
2	Ag_2CO_3 (25 mol%) + CuOAc (3.5 equiv.)	7% (7:1)[c]
3	Ag_2CO_3 (25 mol%)	18% (7:1)
4	Ag_2CO_3 (50 mol%	32% (8:1)
5	Ag_2CO_3 (75 mol%)	45% (7:1)
6	Ag_2CO_3 (1 equiv.)	52% (10:1)
7	Ag_2CO_3 (1.5 equiv.)	57% (10:1)
8	Ag_2CO_3 (2 equiv.)	58% (10:1)
9	**Ag_2CO_3 (2.5 equiv.)**	**65% (10:1)**
10	AgOAc (3 equiv)	61% (10:1)
11	AgTFA (2.5 equiv.)	33% (4:1)
12	Ag_2CO_3 (1 equiv.) + AgOAc (1 equiv.)	35% (10:1)
13	Ag_2CO_3 (2.5 equiv.) + AgOAc (1 equiv.)	45% (10:1)
14	Ag_2CO_3 (2.5 equiv.) + AgOAc (2.5 equiv.)	37% (10:1)

[a]Yield and selectivity are based on ^{1}H NMR of the crude reaction mixture using 1,3,5-trimethoxybenzene (TMB) as internal standard. Doublet of olefin proton in ^{1}H NMR was used to measure the selectivity. Ratios of *meta*:others are shown in parenthesis. [b]27% C–N coupled carbazole product was observed. [c]21% C–N coupled carbazole product was observed. *Note:* C–N coupled carbazole product was observed when copper salt was used.

2.11 General procedure E: Procedure for *meta*-olefination of 2-phenylaniline substrates

An oven-dried screw capped reaction tube with a magnetic stir-bar was charged with 2-phenylaniline (0.2 mmol) (viscous 2-phenylaniline was weighed first) and **TDG20** (0.22 mmol, 40.5 mg) under air, followed by isopropyl alcohol (2 mL). The reaction mixture was stirred at 80 °C for 2 hours. The mixture was allowed to cool down. Next, solvent was concentrated in vacuo and washed with pentane. The dry crude residue was subjected to $Pd(OAc)_2$ (10 mol%, 0.02 mmol, 4.5 mg), *N*-formyl glycine (*N*-Form-Gly-OH; 20 mol%, 8.3 mg), Ag_2CO_3 (2.5 equiv, 0.5 mmol, 138 mg) in the same reaction tube. Solvent 1,2-dichloroethane (DCE, 2 mL) was added in the reaction tube followed by addition of liquid alkene (3 equiv., 0.6 mmol) by micropipette under air (solid alkenes were weighed before adding solvent). The reaction tube was screwed by a cap fitted with a rubber septum and was vigorously stirred in a preheated oil bath at 100 °C. The reaction mixture was taken out after 24 h, diluted with 10 mL ethyl acetate and filtered through a celite pad. Next, the filtrate mixture was treated with 1 (M) HCl solution and stirred for ten minutes. Organic layer was separated and concentrated under vacuum. The crude mixture was purified by column chromatography using silica gel (100-200 mesh size) and petroleum ether/ethyl acetate as the eluent.

2.12 Characterization data for *meta*-olefination products of 2-phenylanilines

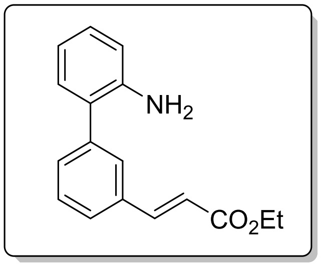

***Ethyl (E)-3-(2'-amino-[1,1'-biphenyl]-3-yl)acrylate*:** Compound **52** was prepared by general procedure E (0.2 mmol scale).
Eluent: petroleum ether/ethyl acetate (92/8, v/v).
Physical State: colorless oil.
Yield: 62% (33 mg isolated; *m*:others = 10:1).
^{1}H NMR (400 MHz, CDCl$_3$) δ (ppm) 7.72 (d, *J* = 16.0 Hz, 1H), 7.63 (s, 1H), 7.52 – 7.44 (m, 3H), 7.18 (ddd, *J* = 7.9, 7.5, 1.6 Hz, 1H), 7.12 (dd, *J* = 7.6, 1.4 Hz, 1H), 6.84 (td, *J* = 7.5, 1.1 Hz, 1H), 6.78 (dd, *J* = 8.0, 0.9 Hz, 1H), 6.48 (d, *J* = 16.0 Hz, 1H), 4.27 (q, *J* = 7.1 Hz, 2H), 3.74 (s, 2H), 1.34 (t, *J* = 7.1 Hz, 3H).
^{13}C NMR (126 MHz, CDCl$_3$) δ (ppm) 167.16, 144.51, 143.66, 140.48, 135.28, 131.15, 130.57, 129.60, 129.07, 128.94, 127.03, 126.93, 119.00, 118.97, 115.95, 60.77, 14.53.
IR (thin film, cm^{-1}): 1036, 1094, 1179, 1270, 1314, 1367, 1415, 1455, 1478, 1638, 1708, 2928, 2981, 3373.
HRMS (*m/z*): [M+H$^+$] calcd for $C_{17}H_{18}NO_2$: 268.1332; found, 268.1331.
TLC: R_f = 0.5 (80:20 petroleum ether:EtOAc).

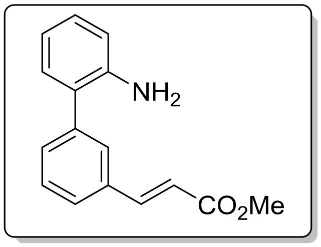

***Methyl (E)-3-(2'-amino-[1,1'-biphenyl]-3-yl)acrylate*:** Compound **53** was prepared by general procedure E (0.2 mmol scale).
Eluent: petroleum ether/ethyl acetate (90/10, v/v).
Physical State: yellow semi-solid.
Yield: 60% (30 mg isolated; *m*:others = 10:1)
^{1}H NMR (400 MHz, CDCl$_3$) δ (ppm) 7.73 (d, *J* = 7.6 Hz, 1H), 7.63 (d, *J* = 16.0 Hz, 1H), 7.53 – 7.46 (m, 3H), 7.18 (td, *J* = 7.6, 2.1 Hz, 1H), 7.12 (dd, *J* = 7.5, 1.1 Hz, 1H), 6.84 (td, *J* = 7.5, 1.1 Hz, 1H), 6.78 (dd, *J* = 8.0, 0.9 Hz, 1H), 6.48 (d, *J* = 16.0 Hz, 1H), 3.81 (s, 3H).
^{13}C NMR (126 MHz, CDCl$_3$) δ (ppm) 167.40, 144.61, 143.45, 140.29, 134.98, 131.04, 130.36, 129.41, 128.89, 128.75, 126.88, 126.69, 118.80, 118.27, 115.75, 51.78.
IR (thin film, cm^{-1}): 1037, 1170, 1273, 1320, 1435, 1499, 1637, 1715, 2853, 2924, 3025, 3219, 3375, 3467.
HRMS (ESI): [M+H$^+$] calcd for $C_{16}H_{16}NO_2$: 254.1176; found, 254.1175.

TLC: R_f = 0.5 (85:15 petroleum ether:EtOAc).

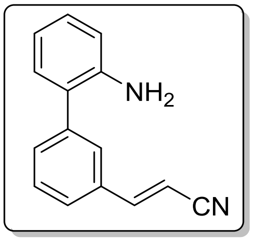

(E)-3-(2'-amino-[1,1'-biphenyl]-3-yl)acrylonitrile: Compound **54** was prepared by general procedure E (0.2 mmol scale).
Eluent: petroleum ether/ethyl acetate (92/8, v/v).
Physical State: colorless oil.
Yield: 57% (25 mg isolated; *m*:others = 3:1).
^{1}H NMR (400 MHz, $CDCl_3$) δ (ppm) 7.57 – 7.54 (m, 2H), 7.51 (d, *J* = 16.7 Hz, 1H), 7.48 – 7.45 (m, 1H), 7.44 – 7.41 (m, 1H), 7.20 (td, *J* = 7.6, 1.5 Hz, 1H), 7.10 (dd, *J* = 7.6, 1.5 Hz, 1H), 6.85 (td, *J* = 7.6, 1.5 Hz, 1H), 6.79 (dd, *J* = 7.2, 2.0 Hz,1H), 5.92 (d, *J* = 16.7 Hz, 1H), 3.72 (s, 2H).
^{13}C NMR (101 MHz, $CDCl_3$) δ (ppm) 150.38, 148.78, 143.43, 140.62, 134.11, 131.96, 130.31, 129.65, 129.12, 128.05, 127.25, 126.20, 118.89, 118.12, 115.86, 96.81.
IR (thin film, cm^{-1}): 1158, 1190, 1298, 1413, 1452, 1481, 1498, 1525, 1579, 1618, 1699, 2217, 2921, 3058, 3210, 3370, 3461.
HRMS (ESI): [M+H$^+$] calcd for $C_{15}H_{13}N_2$: 221.1073; found 221.1078.
TLC: R_f = 0.4 (85:15 petroleum ether:EtOAc).

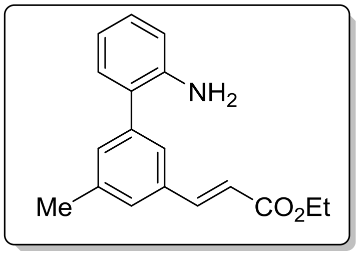

***Ethyl (E)-3-(2'-amino-5-methyl-[1,1'-biphenyl]-3-yl)acrylate*:** Compound **55** was prepared by general procedure E (0.2 mmol scale).
Eluent: petroleum ether/ethyl acetate (92/8, v/v).
Physical State: colorless oil.
Yield: 65% (37 mg isolated; *m*:others = 12:1).
^{1}H NMR (400 MHz, $CDCl_3$) δ (ppm) 7.67 (d, *J* = 16.0 Hz, 1H), 7.47 – 7.41 (m, 1H), 7.30 (t, *J* = 8.9 Hz, 2H), 7.17 (ddd, *J* = 7.9, 7.4, 1.6 Hz, 1H), 7.11 (dd, *J* = 7.6, 1.5 Hz, 1H), 6.82 (tt, *J* = 8.9, 4.5 Hz, 1H), 6.77 (ddd, *J* = 8.4, 5.9, 2.1 Hz, 1H), 6.45 (d, *J* = 16.0 Hz, 1H), 4.26 (q, *J* = 7.1 Hz, 2H), 2.41 (s, 3H), 1.33 (t, *J* = 7.1 Hz, 3H).
^{13}C NMR (101 MHz, $CDCl_3$) δ (ppm) 167.04, 144.49, 143.44, 140.17, 139.16, 134.98, 131.80, 130.31, 128.76, 127.57, 126.88, 125.93, 118.74, 118.51, 115.69, 60.53, 21.37, 14.33

IR (thin film, cm^{-1}): 1037, 1095, 1175, 1286, 1335, 1367, 1460, 1498, 1616, 1638, 1708, 1897, 2854, 2924, 2958, 3374, 3471.
HRMS (*m/z*): [M+H$^+$] calcd for $C_{18}H_{20}NO_2$: 282.1489; found, 282.1490.
TLC: R_f = 0.5 (80:20 petroleum ether:EtOAc).

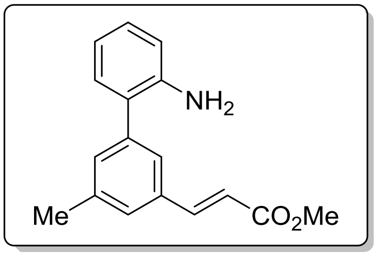

***Methyl (E)-3-(2'-amino-5-methyl-[1,1'-biphenyl]-3-yl)acrylate*:** Compound **56** was prepared by general procedure E (0.2 mmol scale).
Eluent: petroleum ether/ethyl acetate (90/10, v/v).
Physical State: colorless oil.
Yield: 62% (33 mg isolated; *m*:others = 10:1).
^{1}H NMR (400 MHz, CDCl$_3$) δ (ppm) 7.72 (d, *J* = 16.0 Hz, 1H), 7.45 (s, 1H), 7.33 (d, *J* = 9.8 Hz, 2H), 7.19 (t, *J* = 7.6 Hz, 1H), 7.13 (d, *J* = 6.4 Hz, 1H), 6.85 (t, *J* = 7.4 Hz, 1H), 6.80 (d, *J* = 8.0 Hz, 1H), 6.48 (d, *J* = 16.0 Hz, 1H), 3.83 (s, 3H), 2.44 (s, 3H).
^{13}C NMR (101 MHz, CDCl$_3$) δ (ppm) 167.46, 144.78, 143.42, 140.20, 139.18, 134.92, 131.87, 130.30, 128.77, 127.60, 125.94, 118.75, 118.04, 115.70, 51.72, 21.35.
IR (thin film, cm^{-1}): 1035, 1152, 1264, 1390, 1412, 1547, 1601, 1720, 2867, 2945, 3017, 3264, 3382, 3442.
HRMS (*m/z*): [M+H$^+$] calcd for $C_{17}H_{18}NO_2$: 268.1332; found, 268.1332.
TLC: R_f = 0.5 (80:20 petroleum ether:EtOAc).

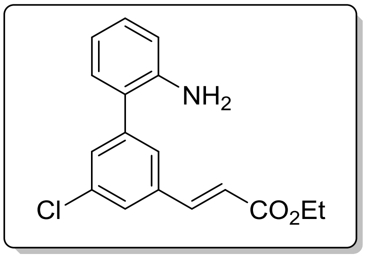

***Ethyl (E)-3-(2'-amino-5-chloro-[1,1'-biphenyl]-3-yl)acrylate*:** Compound **57** was prepared by general procedure E (0.2 mmol scale).
Eluent: petroleum ether/ethyl acetate (91/9, v/v).
Physical State: colorless oil.
Yield: 63% (38 mg isolated; *m*:others = 12:1).
^{1}H NMR (500 MHz, CDCl$_3$) δ (ppm) 7.66 (d, *J* = 7.6 Hz, 1H), 7.52 (d, *J* = 16.3 Hz, 3H), 7.22 (t, *J* = 7.5 Hz, 1H), 7.12 (d, *J* = 7.6 Hz, 1H), 6.87 (t, *J* = 7.4 Hz, 1H), 6.81 (d, *J* = 7.6 Hz, 1H), 6.49 (d, *J* = 16.3 Hz, 1H), 4.29 (q, *J* = 7.1 Hz, 2H), 1.36 (t, *J* = 7.1 Hz, 3H).
^{13}C NMR (126 MHz, CDCl$_3$) δ (ppm) 166.55, 143.35, 142.79, 141.96, 136.69, 135.29, 130.64, 130.24, 129.35, 127.02, 126.42, 125.32, 120.16, 118.90, 115.91, 60.76, 14.29.

IR (thin film, cm^{-1}): 1037, 1111, 1180, 1273, 1316, 1367, 1459, 1498, 1566, 1640, 1711, 2854, 2925, 3065, 3377, 3471.
HRMS (*m/z*): [M+H$^+$] calcd for $C_{17}H_{17}ClNO_2$: 302.0942; found, 302.0941.
TLC: R$_f$ = 0.45 (85:15 petroleum ether:EtOAc).

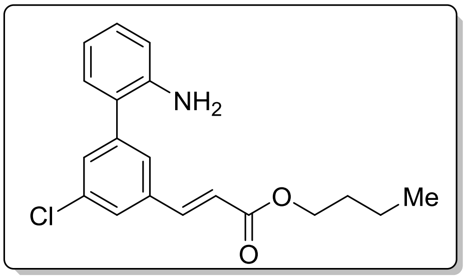

***Butyl (E)-3-(2'-amino-5-chloro-[1,1'-biphenyl]-3-yl)acrylate*:** Compound **58** was prepared by general procedure E (0.2 mmol scale).
Eluent: petroleum ether/ethyl acetate (85/15, v/v).
Physical State: colorless oil.
Yield: 61% (40 mg isolated; *m*:others = 10:1).
^{1}H NMR (500 MHz, CDCl$_3$) δ (ppm) 7.65 (d, *J* = 7.7 Hz, 1H), 7.51 (d, *J* = 16.8 Hz, 1H), 7.23 (t, *J* = 7.7 Hz, 1H), 7.13 (d, *J* = 7.6 Hz, 1H), 6.90 (t, *J* = 7.4 Hz, 1H), 6.85 (d, *J* = 7.7 Hz, 1H), 6.49 (d, *J* = 16.8 Hz, 1H), 4.23 (t, *J* = 6.7 Hz, 2H), 1.72 – 1.69 (m, 2H), 1.48 – 1.44 (m, 2H), 0.97 (t, *J* = 7.2 Hz, 3H).
^{13}C NMR (126 MHz, CDCl$_3$) δ (ppm) 166.65, 143.35, 142.75, 141.95, 136.70, 135.29, 130.63, 130.24, 129.34, 127.02, 126.43, 125.33, 120.50, 120.17, 118.90, 115.91, 64.67, 30.72, 19.19, 13.74.
IR (thin film, cm^{-1}): 1026, 1065, 1176, 1275, 1458, 1498, 1526, 1566, 1640, 1714, 2854, 2926, 2960, 3067, 3379, 3465.
HRMS (*m/z*): [M+H$^+$] calcd for $C_{19}H_{21}ClNO_2$: 330.1255; found, 330.1255.
TLC: R$_f$ = 0.5 (80:20 petroleum ether:EtOAc).

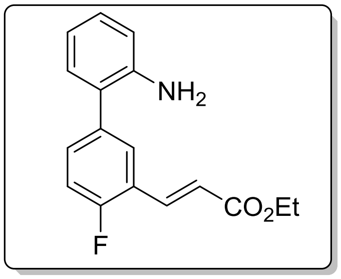

***Ethyl (E)-3-(2'-amino-4-fluoro-[1,1'-biphenyl]-3-yl)acrylate*:** Compound **59** was prepared by general procedure E (0.2 mmol scale).
Eluent: petroleum ether/ethyl acetate (92/8, v/v).
Physical State: colorless oil.
Yield: 62% (35 mg isolated; *m*:others > 20:1).
^{1}H NMR (500 MHz, CDCl$_3$) δ (ppm) 7.86 (d, *J* = 16.2 Hz, 1H), 7.66 (dd, *J* = 7.1, 2.2 Hz, 1H), 7.49 – 7.45 (m, 1H), 7.22 – 7.18 (m, 2H), 7.11 (dd, *J* = 7.6, 1.5 Hz, 1H), 6.86 (td, *J* = 7.5, 1.2 Hz,

1H), 6.80 (dd, *J* = 7.6, 1.5 Hz, 1H), 6.59 (d, *J* = 16.2 Hz, 1H), 4.30 (q, *J* = 7.1 Hz, 2H), 1.36 (t, *J* = 7.2 Hz, 3H).
^{13}C NMR (126 MHz, CDCl$_3$) δ (ppm) 166.75, 160.50 (d, *J* = 254.5 Hz), 159.49, 143.46, 136.94, 132.37 (d, *J* = 8.7 Hz), 130.36, 129.70 (d, *J* = 3.1 Hz), 128.98, 125.84, 122.86 (d, *J* = 11.8 Hz), 121.29 (d, *J* = 6.5 Hz), 118.86, 116.62 (d, *J* = 22.1 Hz), 115.79, 60.68, 14.30.
^{19}F NMR (471 MHz, CDCl$_3$) δ (ppm) -116.52.
IR (thin film, cm^{-1}): 982, 1032, 1105, 1177, 1226, 1257, 1278, 1367, 1397, 1484, 1637, 1708, 1898, 2853, 2927, 3373.
HRMS (ESI): [M+H$^+$] calcd for $C_{17}H_{17}FNO_2$: 286.1238; found, 286.1237.
TLC: R$_f$ = 0.5 (85:15 petroleum ether:EtOAc).

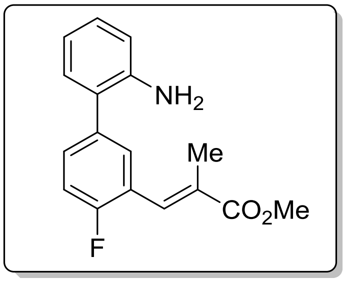

***Ethyl (E)-3-(2'-amino-4-fluoro-5-methyl-[1,1'-biphenyl]-3-yl)but-2-enoate*:** Compound **60** was prepared by general procedure E (0.2 mmol scale).
Eluent: petroleum ether/ethyl acetate (98/8, v/v).
Physical State: colorless oil.
Yield: 56% (33 mg isolated; *m*:others = 4:1).
^{1}H NMR (500 MHz, CDCl$_3$) δ (ppm) 7.34 – 7.32 (m, 1H), 7.18 – 7.14 (m, 2H), 7.11 (t, *J* = 7.6 Hz, 2H), 6.84 (t, *J* = 6.8 Hz, 1H), 6.78 (d, *J* = 7.6 Hz, 1H), 6.30 (s, 1H), 3.78 (s, 3H), 3.72 (s, 3H).
^{13}C NMR (126 MHz, CDCl$_3$) δ (ppm) 167.08, 160.36 (d, *J* = 231.1 Hz), 143.51, 139.27, 138.26, 135.36 (d, *J* = 3.8 Hz), 131.95 (d, *J* = 4.6 Hz), 130.43, 128.98 (d, *J* = 8.0 Hz), 128.59, 126.76, 126.07 (d, *J* = 16.0 Hz), 118.68, 115.75 (d, *J* = 20.2 Hz), 114.07, 52.04, 14.13.
^{19}F NMR (471 MHz, CDCl$_3$) δ (ppm) -119.11.
IR (thin film, cm^{-1}): 1140, 1229, 1275, 1450, 1488, 1619, 1719, 2854, 2925, 3385, 3466.
HRMS (*m/z*): [M+Na$^+$] calcd for $C_{17}H_{16}FNNaO_2$: 308.1057; found, 308.1058.
TLC: R$_f$ = 0.35 (85:15 petroleum ether:EtOAc).

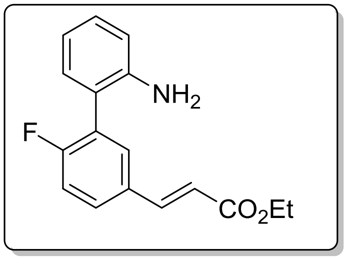

***Ethyl (E)-3-(2'-amino-6-fluoro-[1,1'-biphenyl]-3-yl)acrylate*:** Compound **61** was prepared by general procedure E (0.2 mmol scale).
Eluent: petroleum ether/ethyl acetate (93/7, v/v).
Physical State: yellow oil.

Yield: 53% (30 mg isolated; *m*:others = 4:1).

^{1}H NMR (500 MHz, CDCl$_3$) δ (ppm) 7.90 (d, *J* = 16.2 Hz, 1H), 7.60 – 7.57 (m, 1H), 7.42 (td, *J* = 7.4, 1.7 Hz, 1H), 7.27 – 7.21 (m, 2H), 7.14 (d, *J* = 7.6 Hz, 1H), 6.87 (d, *J* = 7.5 Hz, 1H), 6.84 (d, *J* = 8.0 Hz, 1H), 6.59 (d, *J* = 16.2 Hz, 1H), 4.30 (q, *J* = 7.1 Hz, 2H), 1.37 (t, *J* = 6.4 Hz, 3H).

^{13}C NMR (101 MHz, CDCl3) δ (ppm) 166.78, 159.50, 143.40 (d, *J* = 79.9 Hz), 137.13 (d, *J* = 3.6), 133.73 (d, *J* = 4.3 Hz), 130.94, 129.56, 129.45, 128.34 (d, *J* = 2.9 Hz), 124.73 (d, *J* = 4.3 Hz), 123.30 (d, *J* = 12.7 Hz), 121.27 (d, *J* = 6.5 Hz), 121.00, 118.76, 116.02, 60.69, 14.31.

^{19}F NMR (471 MHz, CDCl$_3$) δ (ppm) -116.17

IR (thin film, cm^{-1}): 1035, 1095, 1174, 1210, 1263, 1314, 1367, 1438, 1501, 1637, 1708, 2854, 2924, 2958, 3378, 3468.

HRMS (*m/z*): [M+H$^+$] calcd for $C_{17}H_{17}FNO_2$: 286.1238; found, 286.1237.

TLC: R$_f$ = 0.5 (80:20 petroleum ether:EtOAc).

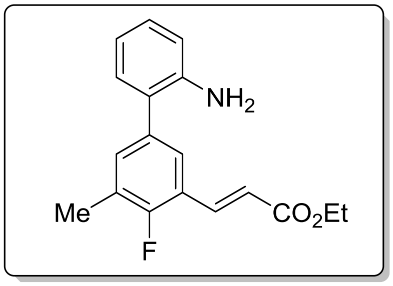

***Ethyl (E)-3-(2'-amino-4-fluoro-5-methyl-[1,1'-biphenyl]-3-yl)acrylate*:** Compound **62** was prepared by general procedure E (0.2 mmol scale).

Eluent: petroleum ether/ethyl acetate (92/8, v/v).

Physical State: yellow oil.

Yield: 69% (42 mg isolated; *m*:others = 8:1).

^{1}H NMR (500 MHz, CDCl$_3$) δ (ppm) 7.87 (d, *J* = 16.2 Hz, 1H), 7.48 (dd, *J* = 6.4, 1.7 Hz, 1H), 7.32 (d, *J* = 5.8 Hz, 1H), 7.21 – 7.17 (m, 1H), 7.10 (dd, *J* = 7.5, 1.2 Hz, 1H), 6.85 (t, *J* = 7.3 Hz, 1H), 6.80 (d, *J* = 8.0 Hz, 1H), 6.57 (d, *J* = 16.2 Hz, 1H), 4.29 (q, *J* = 7.1 Hz, 2H), 2.36 (s, 3H), 1.36 (t, *J* = 7.1 Hz, 3H).

^{13}C NMR (126 MHz, CDCl$_3$) δ (ppm) 166.86, 160.12 (d, *J* = 5.4 Hz), 158.10, 143.40, 137.31 (d, *J* = 3.7, Hz), 135.33 (d, *J*= 4.3 Hz) 133.91 (d, *J* = 5.7 Hz), 130.32, 128.84, 126.95 (d, *J* = 2.7 Hz), 126.18 (d, *J* = 16.5 Hz), 122.52 (d, *J* = 12.7 Hz), 120.94 (d, *J* = 6.3 Hz), 118.82, 115.74, 60.62, 14.68 (d, *J* = 4.66 Hz), 14.31.

^{19}F NMR (471 MHz, CDCl$_3$) δ (ppm) -120.75.

IR (thin film, cm^{-1}): 1040, 1095, 1180, 1273, 1298, 1367, 1475, 1638, 1710, 2854, 2925, 2958, 3373, 3469.

HRMS (ESI): [M+H$^+$] calcd for $C_{18}H_{19}FNO_2$: 300.1394; found, 300.1394.

TLC: R$_f$ = 0.5 (80:20 petroleum ether:EtOAc).

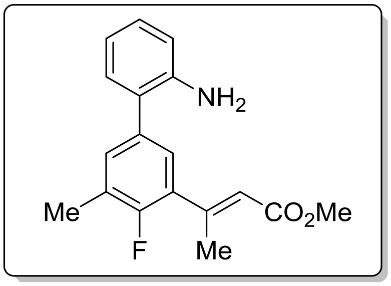

***Ethyl (E)-3-(2'-amino-4-fluoro-5-methyl-[1,1'-biphenyl]-3-yl)but-2-enoate*:** Compound **63** was prepared by general procedure E (0.2 mmol scale).
Eluent: petroleum ether/ethyl acetate (90/10, v/v).
Physical State: colorless oil.
Yield: 58% (34 mg isolated; *m*:others = 4:1).
^{1}H NMR (500 MHz, $CDCl_3$) δ (ppm) 7.21 – 7.18 (m, 2H), 7.11 (d, *J* = 6.7 Hz, 2H), 6.84 (t, *J* = 7.2 Hz, 1H), 6.78 (d, *J* = 6.7 Hz, 1H), 6.06 (s, 1H), 3.78 (s, 3H), 2.58 (s, 3H), 2.36 (s, 3H).
^{13}C NMR (126 MHz, $CDCl_3$) δ (ppm) 166.86, 160.12, 158.10, 143.40, 137.31, 135.33, 133.9 (d, *J* =25.4 Hz, C-F), 130.32, 128.84, 126.95, 126.41, 122.52, 120.98 (d, *J* =18.7 Hz, C-F), 118.82, 115.74, 60.62, 14.68, 14.65.
^{19}F NMR (471 MHz, $CDCl_3$) δ (ppm) -119.56, -120.50.
IR (thin film, cm^{-1}): 1036, 1120, 1171, 1260, 1336, 1457, 1618, 1721, 2854, 2926.
HRMS (*m/z*): [M+H$^+$] calcd for $C_{18}H_{19}FNO_2$: 300.1394; found, 300.1398.
TLC: R_f = 0.5 (80:20 petroleum ether:EtOAc).

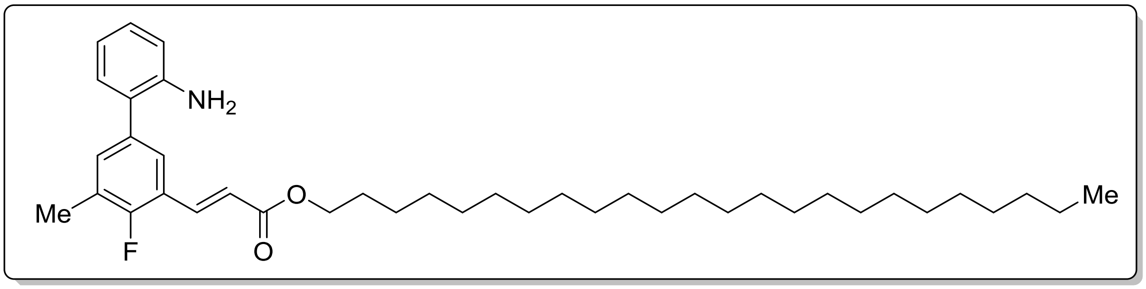

Tricosyl (E)-3-(2'-amino-4-fluoro-5-methyl-[1,1'-biphenyl]-3-yl)acrylate: Compound **64** was prepared by general procedure E (0.2 mmol scale).
Eluent: petroleum ether/ethyl acetate (92/8, v/v).
Physical State: colorless oil.
Yield: 69% (82 mg isolated; *m*:others = 12:1).
^{1}H NMR (500 MHz, $CDCl_3$) δ (ppm) 7.87 (d, *J* = 16.2 Hz, 1H), 7.48 (d, *J* = 7.5 Hz, 1H), 7.31 (t, *J* = 7.5 Hz, 1H), 7.19 (dd, *J* = 11.0, 4.4 Hz, 1H), 7.10 (dd, *J* = 7.5, 1.2 Hz, 1H), 6.85 (t, *J* = 7.3 Hz, 1H), 6.79 (d, *J* = 9.3 Hz, 1H), 6.57 (d, *J* = 16.2 Hz, 1H), 4.22 (t, *J* = 6.7 Hz, 2H), 2.36 (s, 3H), 1.77 – 1.67 (m, 4H), 1.45 – 1.37 (m, 4H),1.29 – 1.28 (m, 36H), 0.91 (t, *J* = 6.8 Hz, 3H).
^{13}C NMR (126 MHz, $CDCl_3$) δ (ppm) 166.96, 157.21, 137.28, 135.41 (d, *J* = 25.4 Hz), 133.96, 133.88, 130.32, 128.84, 128.25, 128.11, 126.96 (d, *J* = 2.7 Hz), 126.18 (d, *J* = 18.2 Hz), 122.50 (d, *J* = 14.7 Hz), 122.50 (d, *J* = 14.7 Hz), 120.97 (d, *J* = 6.0 Hz), 118.87 (d, *J* = 6.8 Hz), 115.79, 115.75, 64.86, 31.94, 29.67, 29.60, 29.55, 29.37, 29.30, 28.71, 25.98, 14.65 (d, *J* = 4.2 Hz).
^{19}F NMR (471 MHz, $CDCl_3$) δ (ppm) -120.73.

IR (thin film, cm^{-1}): 1175, 1273, 1298, 1379, 1467, 1638, 1715, 2852, 2920, 3377.
HRMS (*m/z*): [M+H$^+$] calcd for $C_{39}H_{61}FNO_3$: 594.4680; found, 594.4682.
TLC: R$_f$ = 0.5 (80:20 petroleum ether:EtOAc).

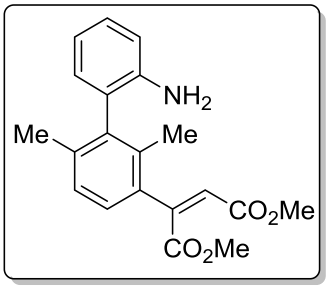

***Dimethyl 2-(2'-amino-4-fluoro-2,6-dimethyl-[1,1'-biphenyl]-3-yl)maleate*:** Compound **65** was prepared by general procedure E (0.2 mmol scale).
Eluent: petroleum ether/ethyl acetate (90/10, v/v).
Physical State: colorless oil.
Yield: 49% (33 mg isolated; *m*:others = 10:1)
^{1}H NMR (500 MHz, CDCl$_3$) δ (ppm) 7.27 – 7.24 (m, 1H), 7.19 (t, *J* = 7.5 Hz, 2H), 6.92 (d, *J* = 6.2 Hz, 1H), 6.86 (t, *J* = 6.9 Hz, 1H), 6.83 (d, *J* = 7.8 Hz, 1H), 6.07 (s, 1H), 3.87 (s, 3H), 3.82 (s, 3H), 2.07 (s, 6H).
^{13}C NMR (126 MHz, CDCl$_3$) δ (ppm) 168.11, 165.48, 148.37, 138.83, 135.30, 133.22, 129.67, 128.51, 127.91, 127.76, 126.00, 123.47, 52.63, 52.07, 20.41, 17.66.
IR (thin film, cm^{-1}): 1168, 1198, 1226, 1256, 1343, 1435, 1497, 1616, 1727, 2854, 2924, 3382, 3473.
HRMS (*m/z*): [M+Na$^+$] calcd for $C_{20}H_{21}NNaO_4$: 362.1363; found, 362.1361.
TLC: R$_f$ = 0.4 (80:20 petroleum ether:EtOAc).

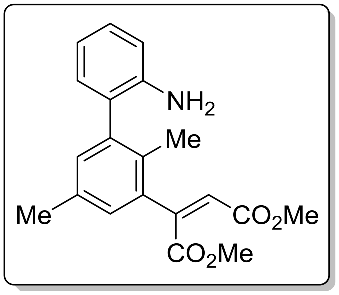

***Dimethyl 2-(2'-amino-2,5-dimethyl-[1,1'-biphenyl]-3-yl)maleate*:** Compound **66** was prepared by general procedure E (0.2 mmol scale).
Eluent: petroleum ether/ethyl acetate (90/10, v/v).
Physical State: colorless oil.
Yield: 62% (42 mg isolated; *m*:others = 8:1)
^{1}H NMR (500 MHz, CDCl$_3$) δ (ppm) 7.19 (d, *J* = 14.3 Hz, 2H), 7.15 (s, 1H), 7.09 (s, 1H), 7.01 (d, *J* = 7.0 Hz, 1H), 6.83 (t, *J* = 7.1 Hz, 1H), 6.79 (d, *J* = 7.8 Hz, 1H), 6.08 (s, 1H), 3.88 (s, 3H), 3.83 (s, 3H), 2.36 (s, 3H), 2.13 (s, 3H).

^{13}C NMR (126 MHz, CDCl$_3$) δ (ppm) 167.96, 165.43, 148.41, 143.55, 139.81, 139.30, 135.83, 135.68, 131.98, 130.07, 128.72, 128.55, 123.67, 118.44, 115.16, 114.08, 52.66, 52.11, 20.78, 16.69.
IR (thin film, cm^{-1}): 1168.16, 1198.40, 1226.73, 1256.04, 1343.56, 1435.95, 1497.52, 1616.11, 1727.89, 2854.67, 2924.89, 3382.68, 3473.87.
HRMS (*m/z*): [M+Na$^+$] calcd for $C_{20}H_{21}NNaO_4$: 362.1363; found, 362.1363.
TLC: R$_f$ = 0.4 (80:20 petroleum ether:EtOAc).

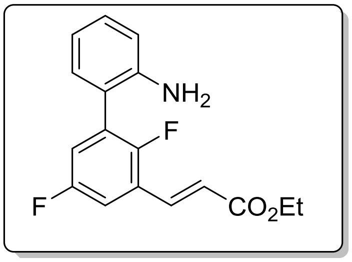

Ethyl (E)-3-(2'-amino-2,5-difluoro-[1,1'-biphenyl]-3-yl)acrylate: Compound **67** was prepared by general procedure E (0.2 mmol scale).
Eluent: petroleum ether/ethyl acetate (95/5, v/v).
Physical State: yellow oil.
Yield: 56% (34 mg isolated; *m*:others = 10:1).
^{1}H NMR (400 MHz, CDCl$_3$) δ (ppm) 7.82 (d, *J* = 16.2 Hz, 1H), 7.26 – 7.22 (m, 2H), 7.12 – 7.09 (m, 2H), 6.85 – 6.80 (m, 2H), 6.54 (d, *J* = 16.2 Hz, 1H), 4.29 (q, *J* = 7.1 Hz, 2H), 1.34 (t, *J* = 7.1 Hz, 3H).
^{13}C NMR (101 MHz, CDCl$_3$) δ (ppm) 166.78, 159.50, 143.40 (d, *J* = 79.9 Hz), 137.13 (d, *J* = 3.6), 133.73 (d, *J* = 4.3 Hz), 130.94, 129.56, 129.45, 128.34 (d, *J* = 2.9 Hz), 124.73 (d, *J* = 4.3 Hz), 123.30, 121.27 (d, *J* = 6.5 Hz), 121.00, 118.76, 116.02, 60.69, 14.31.
^{19}F NMR (471 MHz, CDCl$_3$) δ (ppm) -117.50, -121.74.
IR (thin film, cm^{-1}): 1036, 1095, 1178, 1271, 1302, 1345, 1369, 1448, 1620, 1712, 2855, 2925, 2958, 3071, 3234, 3377, 3479.
HRMS (*m/z*): [M+H$^+$] calcd for $C_{17}H_{16}F_2NO_2$: 304.1144; found, 304.1143.
TLC: R$_f$ = 0.5 (80:20 petroleum ether:EtOAc).

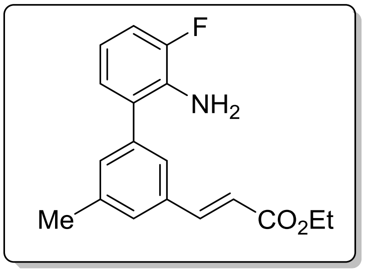

Ethyl (E)-3-(2'-amino-3'-fluoro-5-methyl-[1,1'-biphenyl]-3-yl)acrylate: Compound **68** was prepared by general procedure E (0.2 mmol scale).
Eluent: petroleum ether/ethyl acetate (92/8, v/v).
Physical State: colorless oil.
Yield: 71% (42 mg isolated; *m*:others = 12:1).

^{1}H NMR (500 MHz, CDCl$_3$) δ (ppm) 7.72 (d, *J* = 16.0 Hz, 1H), 7.45 (s, 1H), 7.37 (s, 1H), 7.32 (s, 1H), 7.03 (t, *J* = 7.2 Hz, 1H), 6.93 (d, *J* = 7.1 Hz, 1H), 6.79 – 6.75 (m, 1H), 6.49 (d, *J* = 16.0 Hz, 1H), 4.29 (q, *J* = 7.1 Hz, 2H), 3.83 (s, 2H), 2.45 (s, 3H), 1.36 (t, *J* = 7.1 Hz, 3H).
^{13}C NMR (126 MHz, CDCl$_3$) δ (ppm) 166.95, 151.77 (d, *J* = 238.5 Hz), 144.27, 139.01 (d, *J* = 3.0 Hz), 135.14, 132.19 (d, *J* = 12.6 Hz), 131.53, 129.62, 128.80, 128.29, 127.91, 125.48 (d, *J* = 2.9 Hz), 118.74, 117.81 (d, *J* = 7.9 Hz), 114.29 (d, *J* = 19.0 Hz), 60.55, 21.36, 14.32.
^{19}F NMR (471 MHz, CDCl$_3$) δ (ppm) -134.18.
IR (thin film, cm^{-1}): 1038, 1096, 1172, 1215, 1272, 1367, 1477, 1597, 1629, 1710, 2856, 2927, 3383.
HRMS (ESI): [M+Na$^+$] calcd for $C_{18}H_{18}FNNaO_2$: 322.1214; found, 322.1215.
TLC: R$_f$ = 0.4 (80:20 petroleum ether:EtOAc).

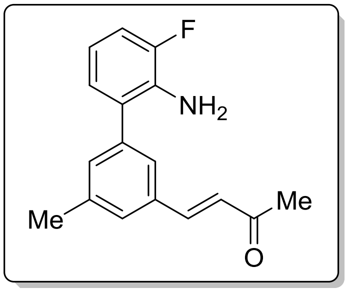

***(E)-4-(2'-amino-3'-fluoro-5-methyl-[1,1'-biphenyl]-3-yl)but-3-en-2-one*:** Compound **69** was prepared by general procedure E (0.2 mmol scale).
Eluent: petroleum ether/ethyl acetate (91/9, v/v).
Physical State: colorless oil.
Yield: 60% (32 mg isolated; *m*:others = 10:1).
^{1}H NMR (400 MHz, CDCl$_3$) δ (ppm) 7.52 (d, *J* = 16.3 Hz, 1H), 7.44 (s, 1H), 7.36 (s, 1H), 7.31 (s, 1H), 7.01 (ddd, *J* = 10.9, 8.1, 1.3 Hz, 1H), 6.91 (d, *J* = 7.7 Hz, 1H), 6.77 – 6.72 (m, 2H), 3.81 (s, 2H), 2.43 (s, 3H), 2.38 (s, 3H).
^{13}C NMR (101 MHz, CDCl$_3$) δ (ppm) 198.36, 151.77 (d, *J* = 238.85 Hz, C-F), 143.11, 139.45, 139.11 (d, *J* = 3.04 Hz, C-F), 135.09, 132.23 (d, *J* = 13.00 Hz, C-F), 131.85, 128.67 (d, *J* = 3.27 Hz, C-F), 128.08, 127.43, 126.00, 125.40 (d, *J* = 2.89, C-F), 117.86 (d, *J* = 7.58 Hz, C-F), 114.34 (d, *J* = 19.06 Hz), 27.69, 21.38.
^{9}F NMR (471 MHz, CDCl$_3$) δ (ppm) -134.33.
IR (thin film, cm^{-1}): 1064, 1172, 1215, 1254, 1360, 1433, 1477, 1625, 1668, 2856, 2924, 3020, 3371.
HRMS (ESI): [M+H$^+$] calcd for $C_{17}H_{17}FNO$: 270.1289; found 270.1284.
TLC: R$_f$ = 0.4 (80:20 petroleum ether:EtOAc).

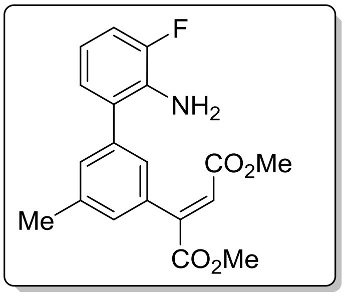

***Dmethyl 2-(2'-amino-3'-fluoro-5-methyl-[1,1'-biphenyl]-3-yl)maleate*:** Compound **70** was prepared by general procedure E (0.2 mmol scale).

Eluent: petroleum ether/ethyl acetate (93/7, v/v).

Physical State: colorless solid.

Yield: 52% (30 mg isolated; *m*:others = 20:1).

^{1}H NMR (400 MHz, $CDCl_3$) δ (ppm) 7.36 (d, *J* = 14.6 Hz, 2H), 7.27 (s, 1H), 7.00 (ddd, *J* = 10.8, 8.1, 1.3 Hz, 1H), 6.89 (d, *J* = 7.6 Hz, 1H), 6.76 – 6.71 (m, 1H), 6.35 (s, 1H), 3.96 (s, 3H), 3.79 (s, 3H), 2.42 (s, 3H).

^{13}C NMR (101 MHz, $CDCl_3$) δ (ppm) 168.11, 165.48, 148.37, 139. 18 (d, *J* = 34.3 Hz), 138.83, 135.30, 133.22, 129.67, 128.51, 127.91, 127.76, 126.00, 123.47, 120.64, 118.80, 115.33, 52.63, 52.07, 20.41

^{19}F NMR (471 MHz, $CDCl_3$) δ (ppm).-134.18.

IR (thin film, cm^{-1}): 1021, 1048, 1168, 1197, 1269, 1348, 1436, 1478, 1597, 1626, 1722, 2854, 2925, 3383, 3473.

HRMS (ESI): [M+Na^+] calcd for $C_{19}H_{18}FNNaO_4$: 366.1112; found, 366.1112.

TLC: R_f = 0.5 (80:20 petroleum ether:EtOAc).

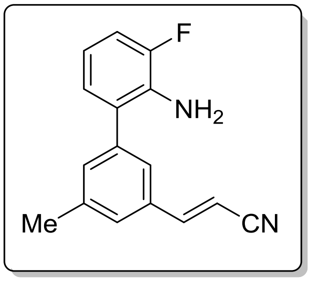

***(E)-3-(2'-amino-3'-fluoro-5-methyl-[1,1'-biphenyl]-3-yl)acrylonitrile*:** Compound **71** was prepared by general procedure E (0.2 mmol scale).

Eluent: petroleum ether/ethyl acetate (92/8, v/v).

Physical State: colorless oil.

Yield: 47% (24 mg isolated; *m*:others = 6:1).

^{1}H NMR (500 MHz, $CDCl_3$) δ (ppm) 7.43 (d, *J* = 16.6 Hz, 1H), 7.37 (s, 1H), 7.29 (s, 1H), 7.06 – 7.02 (m, 2H), 6.91 (d, *J* = 7.6 Hz, 1H), 6.79 – 6.75 (m, 1H), 5.92 (d, *J* = 16.6 Hz, 1H), 3.77 (s, 2H), 2.46 (s, 3H).

^{13}C NMR (126 MHz, $CDCl_3$) δ (ppm) 150.32, 139.72, 134.13, 134.09, 132.61, 132.37, 128.79 (d, *J* = 114.96 Hz, C-F), 127.33, 125.50 (d, *J* = 3.76 Hz, C-F), 125.14, 118.13, 114.71, 114.56, 96.80, 21.35.

^{19}F NMR (471 MHz, CDCl$_3$) δ (ppm) -120.08.
IR (thin film, cm^{-1}): 1040, 1066, 1140, 1173, 1217, 1272, 1436, 1477, 1595, 1622, 2218, 2854, 2923, 3380, 3482
HRMS (ESI): [M+H$^+$] calcd for $C_{16}H_{14}FN_2$: 253.1136; found 253.1139
TLC: R_f = 0.4 (80:20 petroleum ether:EtOAc).

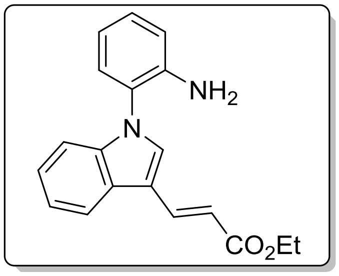

***Ethyl (E)-3-(1-(2-aminophenyl)-1H-indol-3-yl)acrylate*:** Compound **72** was prepared by general procedure E (0.2 mmol scale).
Eluent: petroleum ether/ethyl acetate (90/10, v/v).
Physical State: colorless oil.
Yield: 37% (23 mg isolated; *m*:others = 12:1).
^{1}H NMR (400 MHz, CDCl$_3$) δ (ppm) 7.99 (dd, *J* = 6.5, 2.0 Hz, 1H), 7.95 (d, *J* = 16.0 Hz, 1H), 7.51 (s, 1H), 7.33 – 7.30 (m, 1H), 7.28 (dd, *J* = 7.2, 2.0 Hz, 2H), 7.18 (td, *J* = 7.9, 1.5 Hz, 2H), 6.91 – 6.89 (m, 1H), 6.86 (dd, *J* = 7.6, 1.5 Hz, 1H), 6.51 (d, *J* = 16.0 Hz, 1H), 4.29 (q, *J* = 7.1 Hz, 2H), 3.60 (s, 2H), 1.36 (t, *J* = 7.1 Hz, 3H).
^{13}C NMR (101 MHz, CDCl$_3$) δ (ppm) 168.14, 142.88, 137.76, 137.62, 132.55, 129.87, 128.44, 126.03, 124.60, 123.68, 123.59, 121.92, 120.65, 118.73, 116.48, 114.02, 111.51, 60.17, 14.45.
IR (thin film, cm^{-1}): 1042, 1161, 1215, 1370, 1466, 1625, 1704, 2851, 2927.
HRMS (ESI): [M+H$^+$] calcd for $C_{19}H_{19}N_2O_2$: 307.1441; found 307.1443.
TLC: R_f = 0.5 (80:20 petroleum ether:EtOAc).

2.13 Post-synthetic applications

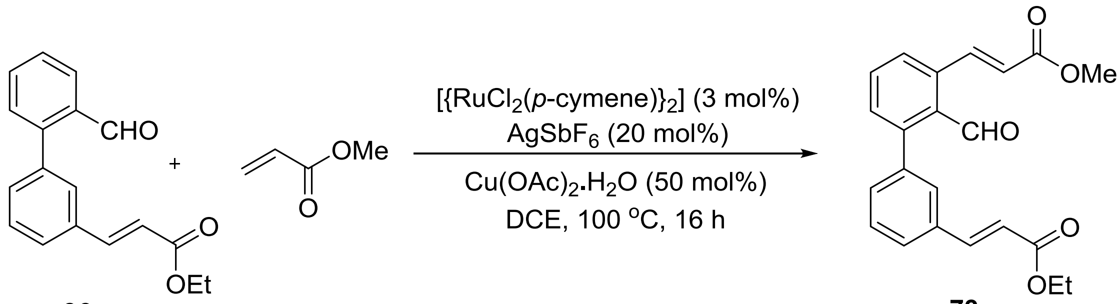

36 73

Procedure: A modified procedure was followed from the literature report.[2] A 25 mL two-neck round bottom flask containing [{$RuCl_2$(*p*-cymene)}$_2$] (0.007 mmol, 4.4 mg), $AgSbF_6$ (0.071 mmol, 24.5 mg) and $Cu(OAc)_2$ (0.1785 mmol, 32.3 mg) was evacuated and purged with nitrogen gas three times. To the flask or tube were then added aldehyde (**36**, 0.357 mmol), methyl acrylate (1.785 mmol, 170 µL) and 1,2-dichloroethane (7.0 mL) *via* syringes and allowed the reaction mixture to stir at room temperature for 5 min. Then, the reaction mixture was allowed to stir at 100 °C for 16 h under open atmosphere. After cooling to ambient temperature, the reaction mixture was diluted with CH_2Cl_2, filtered through Celite and silica gel, and the filtrate was concentrated. The crude residue was purified through a silica gel column using hexanes and ethyl acetate as eluent to give desired product.

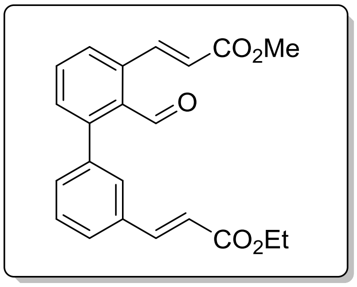

***Ethyl (E)-3-(3'-chloro-2'-formyl-[1,1'-biphenyl]-3-yl)acrylate*:** Compound **73** was prepared by above procedure.
Eluent: petroleum ether/ethyl acetate (90/10, v/v).
Physical State: yellow oil.
Yield: 66% (85 mg isolated).
^{1}H NMR (500 MHz, $CDCl_3$) δ (ppm) 9.99 (s, 1H), 8.38 (d, *J* = 15.9 Hz, 1H), 7.71 (d, *J* = 16.0 Hz, 1H), 7.63 (d, *J* = 4.9 Hz, 2H), 7.61 (d, *J* = 7.7 Hz, 1H), 7.51 – 7.49 (m, 2H), 7.47 (dd, *J* = 7.5, 3.3 Hz, 1H), 7.35 (d, *J* = 7.7 Hz, 1H), 6.48 (d, *J* = 16.0 Hz, 1H), 6.37 (d, *J* = 15.9 Hz, 1H), 4.27 (q, *J* = 7.1 Hz, 2H), 3.83 (s, 3H), 1.33 (t, *J* = 7.1 Hz, 3H).
^{13}C NMR (126 MHz, $CDCl_3$) δ (ppm) 193.29, 167.07, 166.90, 146.22, 144.00, 143.77, 138.86, 136.42, 135.14, 133.00, 132.70, 132.14, 131.84, 129.48, 129.33, 128.06, 122.07, 119.82, 115.51, 60.89, 52.09, 14.50.
IR (thin film, cm^{-1}): 695, 755, 795, 864, 978, 1035, 1094, 1167, 1263, 1311, 1367, 1411, 1435, 1576, 1637, 1707, 2760, 2852, 2952, 2982.
HRMS (ESI): [M+Na$^+$] calcd. for $C_{22}H_{20}NaO_5$: 387.1203, found 387.1200.
TLC: R_f = 0.4 (85:15 petroleum ether:EtOAc).

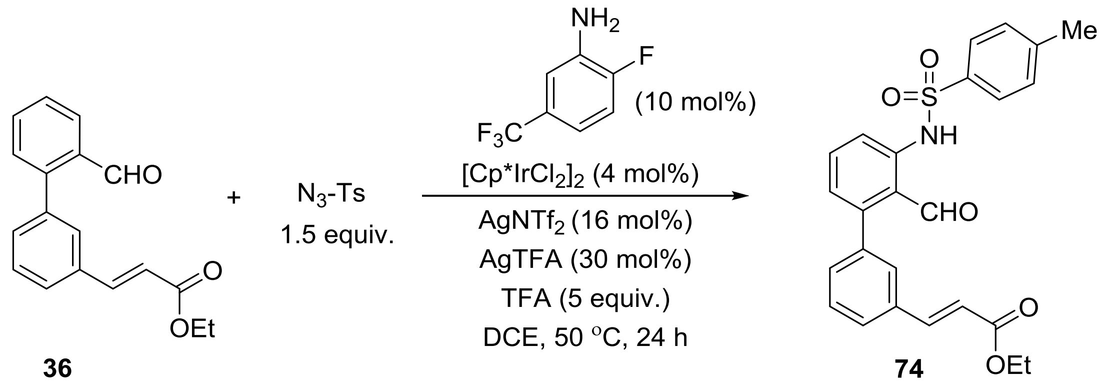

36 **74**

Procedure: A modified procedure was followed from the literature report.[3] To a solution of compound **36** (0.2 mmol) and tosyl azide (0.3 mmol, 59.2 mg) in 1.0 mL of DCE were added $[Cp^*IrCl_2]_2$ (0.008 mmol, 6.4 mg), $AgNTf_2$ (0.032 mmol, 12.4 mg), AgTFA (0.06 mmol, 13.2 mg), 2-fluoro-5-(trifluoromethyl)aniline (0.002 mmol, 3.6 mg), and TFA (1.0 mmol, 77 μL). The mixture was stirred at 50 °C for 24 hours. Upon completion, the reaction mixture was cooled to room temperature and concentrated in vacuo. The crude residue was purified by column chromatography on silica gel using petroleum ether/EtOAc as eluent to afford the desired product.

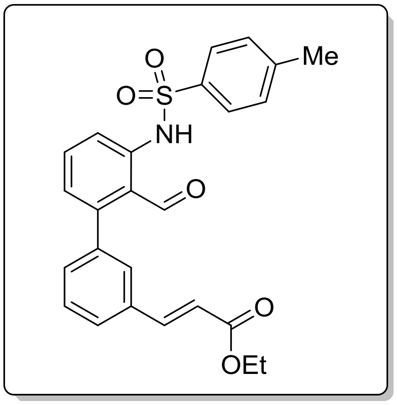

Ethyl (E)-3-(2'-formyl-3'-((4-methylphenyl)sulfonamido)-[1,1'-biphenyl]-3-yl)acrylate: Compound **74** was prepared by above procedure.

Eluent: petroleum ether/ethyl acetate (70/30, v/v).

Physical State: yellow oil.

Yield: 61% (54 mg isolated).

^{1}H NMR (500 MHz, $CDCl_3$) δ (ppm) 11.35 (s, 1H), 9.73 (s, 1H), 7.81 (d, *J* = 8.3 Hz, 2H), 7.72 (d, *J* = 8.4 Hz, 1H), 7.68 (d, *J* = 16.0 Hz, 1H), 7.58 (d, *J* = 7.9 Hz, 1H), 7.54 – 7.50 (m, 1H), 7.46 (t, *J* = 7.7 Hz, 1H), 7.41 (s, 1H), 7.27 (d, *J* = 9.4 Hz, 3H), 7.02 – 7.00 (m, 1H), 6.46 (d, *J* = 16.0 Hz, 1H), 4.27 (q, *J* = 7.1 Hz, 2H), 2.39 (s, 3H), 1.33 (t, *J* = 7.1 Hz, 3H).

^{13}C NMR (126 MHz, $CDCl_3$) δ (ppm) 195.09, 166.84, 148.20, 144.41, 143.63, 140.92, 138.39, 136.73, 135.49, 135.08, 131.85, 130.01, 129.54, 129.26, 129.15, 128.14, 127.59, 126.22, 125.18, 119.89, 119.42, 117.46, 60.91, 21.79, 14.50.

IR (thin film, cm^{-1}): 797, 896, 986, 1036, 1091, 1164, 1278, 1304, 1381, 1464, 1661, 1713, 2858, 2925.

HRMS (ESI): $[M+Na^+]$ calcd for $C_{25}H_{23}NNaO_5S$: 472.1189, found 472.1188.

TLC: R_f = 0.4 (70:30 petroleum ether:EtOAc).

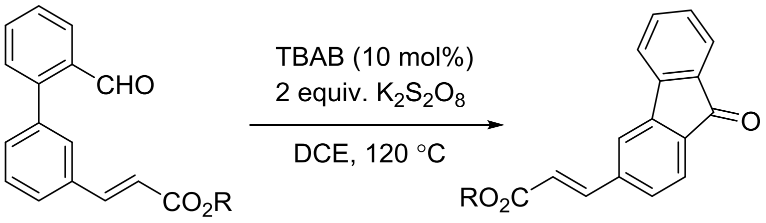

Procedure: A modified procedure was followed from the literature report.[4] To a 10 mL Schleck tube, Tetrabutyl ammonium bromide (TBAB, 10 mol%, 6.45 mg), $K_2S_2O_8$ (2.0 equiv., 108 mg) and the tube was purged with argon for three times, followed by addition of aldehyde (0.2 mmol) and DCE (2.0 mL). The reaction mixture was stirred at 120 ºC under argon for 36 h. The solution was then cooled to room temperature, and DCE was removed under vacuum. The crude product was purified by column chromatography on silica gel (eluent: petroleum ether/ethyl acetate) to afford the desired product.

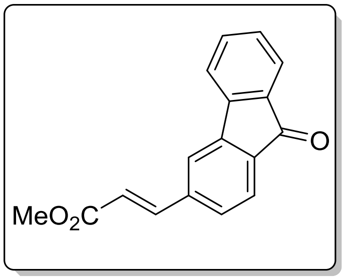

***Methyl (E)-3-(9-oxo-9H-fluoren-1-yl)acrylate*:** Compound **75** was prepared by above procedure (0.2 mmol scale).
Eluent: petroleum ether/ethyl acetate (95/5, v/v).
Physical State: yellow oil.
Yield: 47% (25 mg isolated).
^{1}H NMR (500 MHz, $CDCl_3$) δ (ppm) 8.82 (d, *J* = 16.3 Hz, 1H), 7.67 (d, *J* = 7.3 Hz, 1H), 7.54 – 7.50 (m, 4H), 7.49 – 7.45 (m, 1H), 7.33 (t, *J* = 7.1 Hz, 1H), 6.57 (d, *J* = 16.2 Hz, 1H), 3.87 (s, 3H).
^{13}C NMR (126 MHz, $CDCl_3$) δ (ppm) 193.99, 166.94, 145.09, 143.42, 139.02, 134.76, 134.35, 134.30, 134.02, 130.64, 129.44, 126.30, 124.32, 121.96, 121.33, 120.29, 51.93.
IR (thin film, cm^{-1}): 666, 756, 800, 872, 924, 1036, 1098, 1183, 1233, 1286, 1308, 1367, 1468, 1607, 1638, 1707, 2852, 2925.
HRMS (ESI): [$M+Na^+$] calcd. for $C_{17}H_{12}NaO_3$: 287.067; found 287.0680.
TLC: R_f = 0.4 (95:5 petroleum ether:EtOAc).

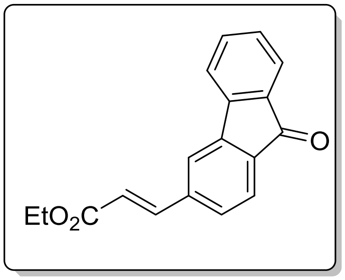

Ethyl (E)-3-(9-oxo-9H-fluoren-3-yl)acrylate: Compound **76** was prepared by the above procedure (0.2 mmol scale).
Eluent: petroleum ether/ethyl acetate (95/5, v/v).
Physical State: yellow oil.
Yield: 52% (52 mg isolated).
^{1}H NMR (500 MHz, $CDCl_3$) δ (ppm) 8.85 (t, *J* = 7.4 Hz, 1H), 7.69 (d, *J* = 16.2 Hz, 1H), 7.57 – 7.54 (m, 3H), 7.54 – 7.52 (m, 1H), 7.51 – 7.48 (m, 1H), 7.35 (td, *J* = 7.4, 2.3 Hz, 1H), 6.58 (d, *J* = 16.2 Hz, 1H), 4.33 (q, *J* = 7.1 Hz, 2H), 1.40 (t, *J* = 7.1 Hz, 3H)
^{13}C NMR (126 MHz, $CDCl_3$) δ (ppm) 194.05, 166.52, 145.12, 143.47, 138.78, 134.75, 134.50, 134.35, 134.08, 130.67, 129.45, 126.36, 124.34, 122.52, 121.25, 120.28, 60.76, 14.32.
IR (thin film, cm^{-1}): 666, 756, 800, 872, 924, 1036, 1098, 1183, 1233, 1286, 1308, 1367, 1468, 1607, 1638, 1707, 2852, 2925.
HRMS (ESI): [M+Na$^+$] calcd. for $C_{18}H_{14}NaO_3$: 301.0835, found 301.0831.
TLC: R_f = 0.4 (95:5 petroleum ether:EtOAc).

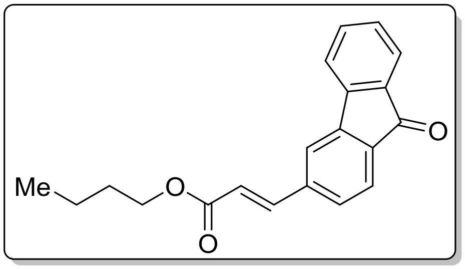

Butyl (E)-3-(9-oxo-9H-fluoren-3-yl)acrylate: Compound **77** was prepared by above procedure (0.2 mmol scale).
Eluent: petroleum ether/ethyl acetate (95/5, v/v).
Physical State: yellow oil.
Yield: 44% (27 mg isolated).
^{1}H NMR (500 MHz, $CDCl_3$) δ (ppm) 8.81 (d, *J* = 16.2 Hz, 1H), 7.66 (d, *J* = 7.4 Hz, 1H), 7.50 (ddd, *J* = 13.3, 10.9, 7.2 Hz, 5H), 7.32 (td, *J* = 7.2, 1.2 Hz, 1H), 6.56 (d, *J* = 16.2 Hz, 1H), 4.25 (t, *J* = 6.7 Hz, 2H), 1.76 – 1.69 (m, 2H), 1.47 (dd, *J* = 15.0, 7.5 Hz, 2H), 0.98 (t, *J* = 7.4 Hz, 3H).
^{13}C NMR (101 MHz, $CDCl_3$) δ (ppm) 194.21, 166.79, 145.31, 143.67, 138.96, 134.93, 134.72, 134.52, 134.29, 130.87, 129.64, 126.57, 124.54, 122.73, 121.43, 120.48, 64.86, 30.97, 19.42, 13.99.
IR (thin film, cm^{-1}): 667, 755, 800, 872, 923, 990, 1024, 1069, 1167, 1232, 1276, 1306, 1382, 1420, 1468, 1582, 1607, 1637, 1704, 2307, 2873, 2933, 2959, 3056.
HRMS (ESI): [M+Na$^+$] calcd. for $C_{20}H_{18}NaO_3$: 329.1148, found 329.1146.
TLC: R_f = 0.25 (95:5 petroleum ether:EtOAc).

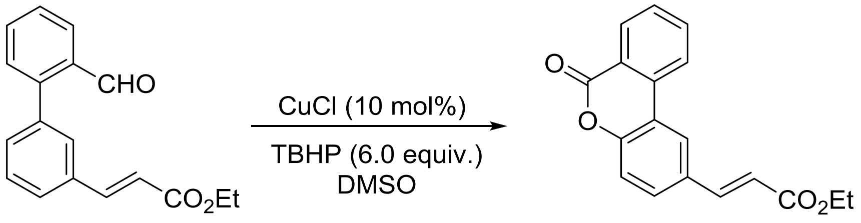

36 **78**

Procedure: A modified procedure was followed from the literature report.[5] The substrate **4** (0.25 mmol) and CuCl (5 mol%, 2.5 mg) were taken in a single neck round bottomed flask and then 2.0 mL DMSO was added. The reaction mixture was stirred and then TBHP (70% in water) (6.0 equiv., 144 μL) was added drop wise. The stirring was continued at room temperature for 4h. After completion of the reaction, the reaction mixture was diluted with water and extracted with ethyl acetate (3 x 20 mL). The combined organic layer was evaporated under reduced pressure and the crude product was purified by column chromatography using silica gel (60 -120 mesh) and hexane/ethyl acetate as eluent.

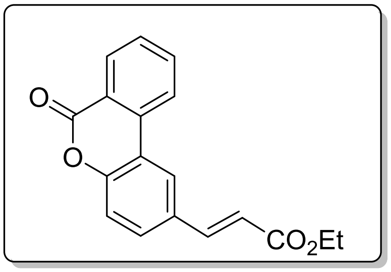

***Ethyl (E)-3-(6-oxo-6H-benzo[c]chromen-2-yl)acrylate*:** Compound **78** was prepared by above procedure.
Eluent: petroleum ether/ethyl acetate (95/5, v/v).
Physical State: yellow oil.
Yield: 52% (30 mg isolated).
^{1}H NMR (400 MHz, $CDCl_3$) δ (ppm) 8.43 (d, *J* = 7.0 Hz, 1H), 8.20 (s, 1H), 8.17 (d, *J* = 8.1 Hz, 1H), 7.88 (t, *J* = 7.7 Hz, 1H), 7.78 (d, *J* = 16.0 Hz, 1H), 7.68 (d, *J* = 8.7 Hz, 1H), 7.63 (t, *J* = 7.9 Hz, 1H), 7.40 (d, *J* = 8.5 Hz, 1H), 6.51 (d, *J* = 16.0 Hz, 1H), 4.29 (q, *J* = 7.3 Hz, 2H), 1.36 (t, *J* = 7.3 Hz, 3H).
^{13}C NMR (126 MHz, $CDCl_3$) δ (ppm) 166.63, 166.27, 144.04, 142.83, 139.32, 135.44, 134.09, 133.88, 133.54, 132.32, 131.44, 131.02, 128.86, 128.64, 128.59, 120.89, 61.00, 52.78, 14.49
IR (thin film, cm^{-1}): 627, 747, 804, 869, 971, 1014, 1098, 1177, 1256, 1337, 1498, 1622, 1740, 2812, 2947.
HRMS (ESI): [$M+H^+$] calcd. for $C_{18}H_{15}O_4$: 295.0965; found: 295.0965.
TLC: R_f = 0.5 (90:10 petroleum ether:EtOAc).

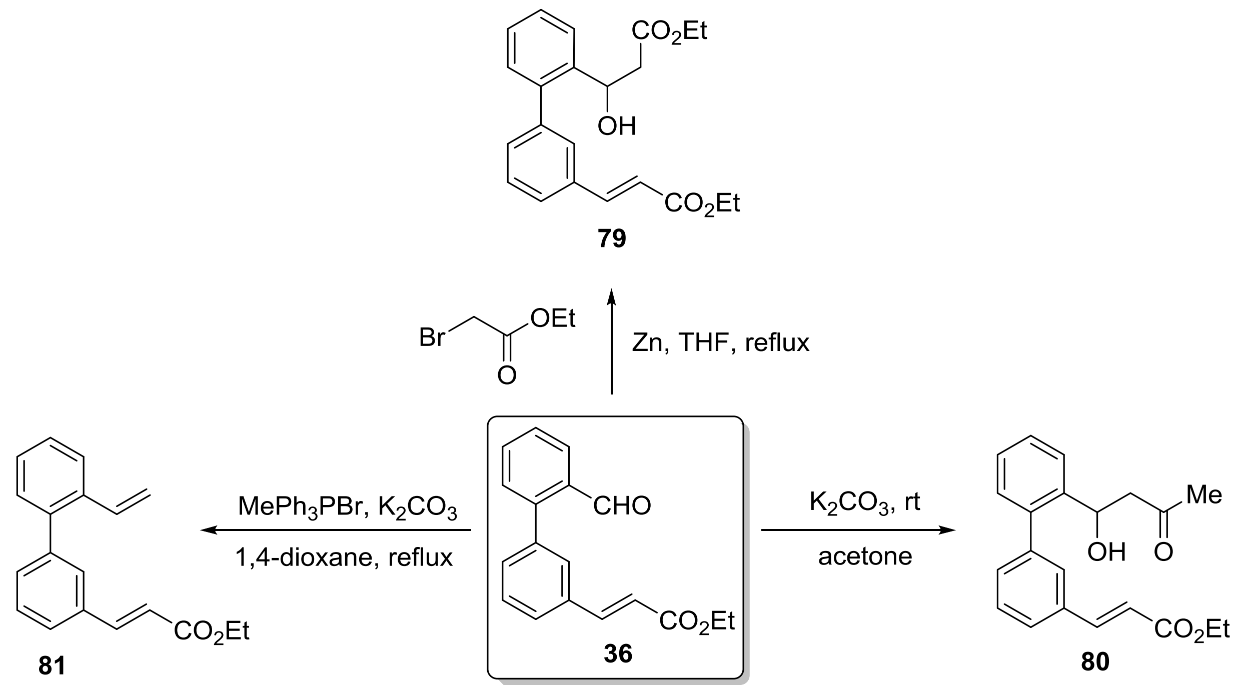

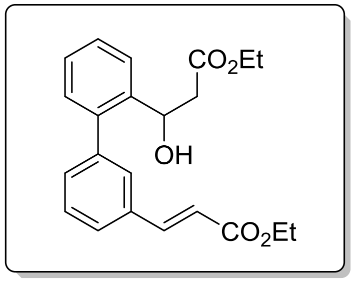

***Ethyl (E)-3-(2'-(3-ethoxy-1-hydroxy-3-oxopropyl)-[1,1'-biphenyl]-3-yl)acrylate*:** Compound **79** was prepared from the literature report (0.2 mmol scale).[6]

Eluent: petroleum ether/ethyl acetate (90/10, v/v).

Physical State: colorless oil.

Yield: 68% (50 mg isolated).

^{1}H NMR (500 MHz, CDCl$_3$) δ (ppm) 7.71 (d, *J* = 16.0 Hz, 1H), 7.65 (d, *J* = 7.8 Hz, 1H), 7.53 (d, *J* = 7.7 Hz, 1H), 7.45 (dd, *J* = 16.2, 8.5 Hz, 3H), 7.35 (t, *J* = 8.2 Hz, 2H), 7.21 (d, *J* = 7.5 Hz, 1H), 6.47 (d, *J* = 16.0 Hz, 1H), 5.25 (d, *J* = 9.6 Hz, 1H), 4.26 (q, *J* = 7.1 Hz, 2H), 4.14 (q, *J* = 7.0 Hz, 2H), 3.36 (s, 1H), 2.93 (dd, *J* = 17.6, 9.8 Hz, 1H), 2.77 (dd, *J* = 17.6, 2.4 Hz, 1H), 1.33 (t, *J* = 7.1 Hz, 3H), 1.23 (t, *J* = 7.1 Hz, 3H).

^{13}C NMR (126 MHz, CDCl$_3$) δ (ppm) 166.88, 166.69, 144.18, 141.28, 139.64, 139.44, 134.71, 130.95, 130.09, 128.93, 128.79, 128.36, 127.76, 126.96, 126.12, 118.95, 66.21, 61.56, 60.56, 50.65, 49.66, 14.31, 14.06.

IR (thin film, cm^{-1}): 698, 760, 801, 864, 915, 983, 1034, 1095, 1178, 1263, 1317, 1367, 1414, 1474, 1638, 1709, 2932, 2980, 3503.

HRMS (ESI): [M+H$^+$] calcd. for $C_{22}H_{24}O_5$: 368.1624, found 368.1632.

TLC: R_f = 0.4 (85:15 petroleum ether:EtOAc).

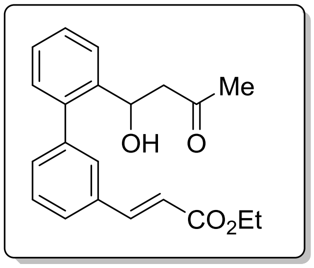

***Ethyl (E)-3-(2'-(1-hydroxy-3-oxobutyl)-[1,1'-biphenyl]-3-yl)acrylate*:** Compound **80** was prepared from the literature report (0.2 mmol scale).[6]
Eluent: petroleum ether/ethyl acetate (90/10, v/v).
Physical State: colorless oil.
Yield: 70% (51 mg isolated).
^{1}H NMR (400 MHz, $CDCl_3$) δ (ppm) 7.71 (d, *J* = 16.0 Hz, 1H), 7.65 (d, *J* = 7.7 Hz, 1H), 7.53 (d, *J* = 7.8 Hz, 1H), 7.45 (dd, *J* = 14.5, 6.8 Hz, 3H), 7.37 – 7.32 (m, 2H), 7.20 (d, *J* = 7.3 Hz, 1H), 6.46 (d, *J* = 16.0 Hz, 1H), 5.24 – 5.19 (m, 1H), 4.27 (q, *J* = 7.1 Hz, 2H), 3.33 (s, 1H), 2.80 (dd, *J* = 17.7, 9.8 Hz, 1H), 2.63 (dd, *J* = 17.7, 2.3 Hz, 1H), 2.07 (s, 3H), 1.33 (t, *J* = 7.1 Hz, 3H).
^{13}C NMR (101 MHz, $CDCl_3$) δ (ppm) 209.32, 167.11, 144.41, 141.63, 139.84, 139.79, 134.85, 131.17, 130.23, 129.13, 128.98, 128.54, 127.84, 127.13, 126.37, 119.12, 66.46, 60.80, 51.23, 30.74, 14.52.
IR (thin film, cm^{-1}): 698, 762, 802, 864, 982, 1036, 1094, 1177, 1270, 1317, 1366, 1415, 1474, 1637, 1707, 2930, 2980, 3488.
HRMS (ESI): $[M+H^+]$ calcd. for $C_{21}H_{22}O_4$: 338.1519, found 338.1522.
TLC: R_f = 0.5 (85:15 petroleum ether:EtOAc).

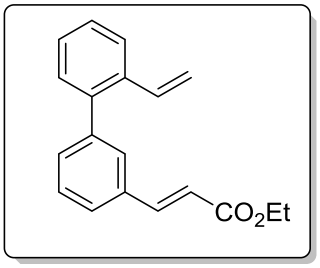

Ethyl (E)-3-(2'-vinyl-[1,1'-biphenyl]-3-yl)acrylate: Compound **81** was prepared from the literature report (0.2 mmol scale).[6]
Eluent: petroleum ether/ethyl acetate (97/3, v/v).
Physical State: colorless oil.
Yield: 78% (43 mg isolated).
^{1}H NMR (500 MHz, $CDCl_3$) δ (ppm) 7.72 (d, *J* = 16.0 Hz, 1H), 7.67 – 7.63 (m, 1H), 7.52 (d, *J* = 8.1 Hz, 2H), 7.43 (t, *J* = 7.6 Hz, 1H), 7.39 – 7.32 (m, 3H), 7.30 – 7.26 (m, 1H), 6.67 (dd, *J* = 17.5, 11.0 Hz, 1H), 6.46 (d, *J* = 16.0 Hz, 1H), 5.71 (dd, *J* = 17.5, 0.9 Hz, 1H), 5.21 (dd, *J* = 11.0, 1.0 Hz, 1H), 4.27 (q, *J* = 7.1 Hz, 2H), 1.34 (t, *J* = 7.1 Hz, 3H).

^{13}C NMR (126 MHz, CDCl$_3$) δ (ppm) 167.21, 144.66, 141.78, 140.16, 136.00, 135.77, 134.53, 131.86, 130.17, 129.64, 128.84, 128.06, 127.99, 126.88, 126.08, 118.85, 115.41, 60.76, 14.53.
IR (thin film, cm^{-1}): 697, 762, 802, 865, 910, 985, 1039, 1094, 1178, 1264, 1318, 1367, 1471, 1639, 1712, 2981.
HRMS (ESI): [M+Na$^+$] calcd. for $C_{19}H_{18}NaO_2$: 301.0835, found 301.0831.
TLC: R$_f$ = 0.5 (95:5 petroleum ether:EtOAc).

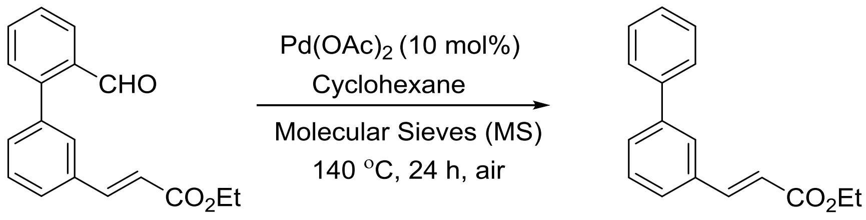

36 **82**

Procedure: A modified procedure was followed from the literature report.[7] A clean, oven-dried screw-cap reaction tube with a magnetic stir-bar was charged with molecular sieves (4 Å, 150 mg), aldehyde **36** (0.2 mmol, 56 mg), and palladium acetate (10 mol%, 4.5 mg). Cyclohexane (1.5 mL) was added to this mixture with a syringe. The screw cap was closed tightly, and the tube was placed in a preheated oil bath at the required temperature. The reaction mixture was stirred vigorously at 140 °C for 24 hours. After the completion of the reaction, the solvent was evaporated under vacuum and the residue was purified by column chromatography on silica gel using petroleum ether/ethyl acetate to obtain the desired product.

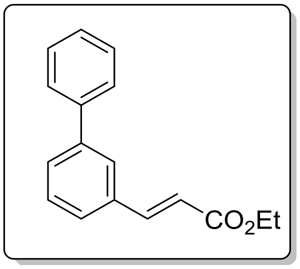

Ethyl (E)-3-([1,1'-biphenyl]-3-yl)acrylate: Compound **82** was prepared by above procedure.
Eluent: petroleum ether/ethyl acetate (95/5, v/v).
Physical State: yellow oil.
Yield: 56% (28 mg isolated).
^{1}H NMR (500 MHz, CDCl$_3$) δ (ppm) 7.81 – 7.75 (m, 2H), 7.65 – 7.60 (m, 3H), 7.56 – 7.53 (m, 1H), 7.49 (t, J = 7.5 Hz, 3H), 7.41 (t, J = 7.5 Hz, 1H), 6.54 (d, J = 16.0 Hz, 1H), 4.31 (q, J = 7.1 Hz, 2H), 1.38 (t, J = 7.1 Hz, 3H).
^{13}C NMR (126 MHz, CDCl$_3$) δ (ppm) 166.99, 144.53, 141.97, 140.45, 134.98, 129.33, 129.04, 128.90, 127.70, 127.15, 126.89, 126.82, 118.65, 60.57, 14.34.
IR (thin film, cm^{-1}): 1032, 1124, 1189, 1248, 1367, 1439, 1552, 2812, 2957.
HRMS (ESI): [M+Na$^+$] calcd. for $C_{17}H_{16}NaO_2$: 303.1043; found: 275.1038.
TLC: R$_f$ = 0.4 (95:5 petroleum ether:EtOAc).

Post-synthetic applications of 55

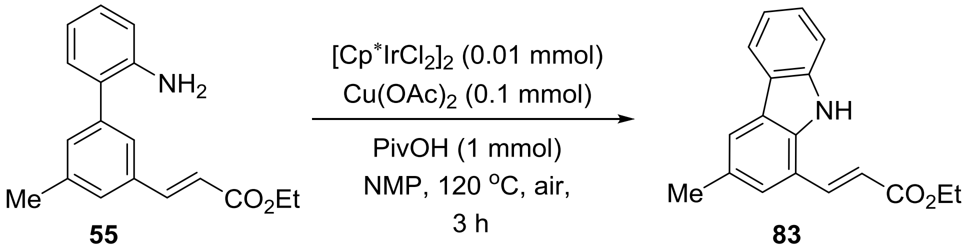

55 **83**

Procedure: The procedure was followed from the modified literature report.[8] To a 20 mL two-necked flask with a reflux condenser and a rubber cup were added **55** (0.5 mmol), $[Cp^*IrCl_2]_2$ (0.01 mmol), $Cu(OAc)_2$ (0.1 mmol), PivOH (1.0 mmol) in NMP (3 mL). The resulting mixture was stirred under air at 120 °C for 3 hours. After cooling, the reaction mixture was extracted with ethyl acetate (100 mL), washed with aqueous $NaHCO_3$ (100 mL, three times), and dried over Na_2SO_4. Purification by column chromatography on silica gel using petroleum ether-ethyl acetate as eluent.

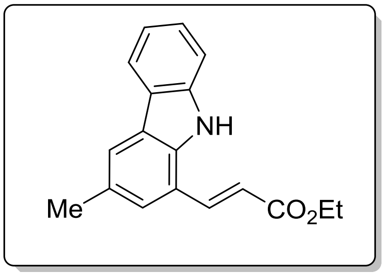

***Ethyl (E)-3-(3-methyl-9H-carbazol-1-yl)acrylate**:* Compound **83** was prepared by above procedure (0.5 mmol scale).

Eluent: petroleum ether/ethyl acetate (80/20, v/v).

Physical State: pale yellow oil.

Yield: 55% (77 mg isolated).

^{1}H NMR (400 MHz, $CDCl_3$) δ (ppm) 11.47 (s, 1H), 7.69 (d, *J* = 16.0 Hz, 1H), 7.32 (s, 1H), 7.30 (s, 1H), 7.17 (ddd, *J* = 7.9, 7.4, 1.6 Hz, 1H), 7.11 (dd, *J* = 7.6, 1.5 Hz, 1H), 6.83 (td, *J* = 7.5, 1.1 Hz, 1H), 6.77 (dd, *J* = 7.6, 1.5 Hz, 1H), 6.46 (d, *J* = 16.0 Hz, 1H), 4.26 (q, *J* = 7.1 Hz, 2H), 2.41 (s, 3H), 1.33 (t, *J* = 7.3 Hz, 3H).

^{13}C NMR (101 MHz, $CDCl_3$) δ (ppm) 167.04, 144.49, 143.44, 140.17, 139.16, 134.98, 131.80, 130.31, 128.76, 127.57, 126.88, 125.93, 118.74, 118.51, 115.69, 60.53, 21.37, 14.33.

IR (thin film, cm^{-1}): 1031, 1115, 1167, 1284, 1305, 1377, 1445, 1495, 1608, 1787, 1917, 2874, 2914, 3341.

HRMS (*m/z*): $[M+H^+]$ calcd for $C_{18}H_{18}NO_2$: 280.1333; found, 280.1334.

TLC: R_f = 0.4 (90:10 petroleum ether:EtOAc).

3. NMR Spectra:

2-(pyridin-3-yl)aniline (TDG3): ^{1}H NMR

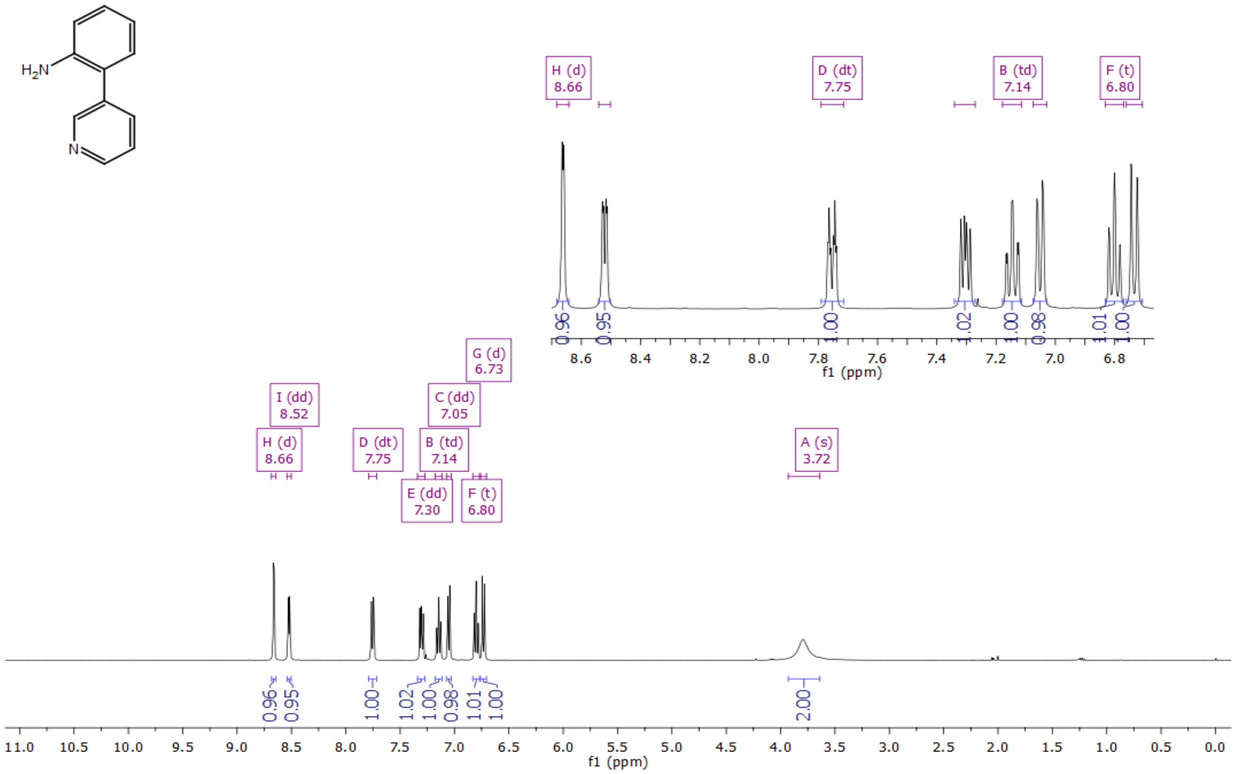

^{13}C NMR

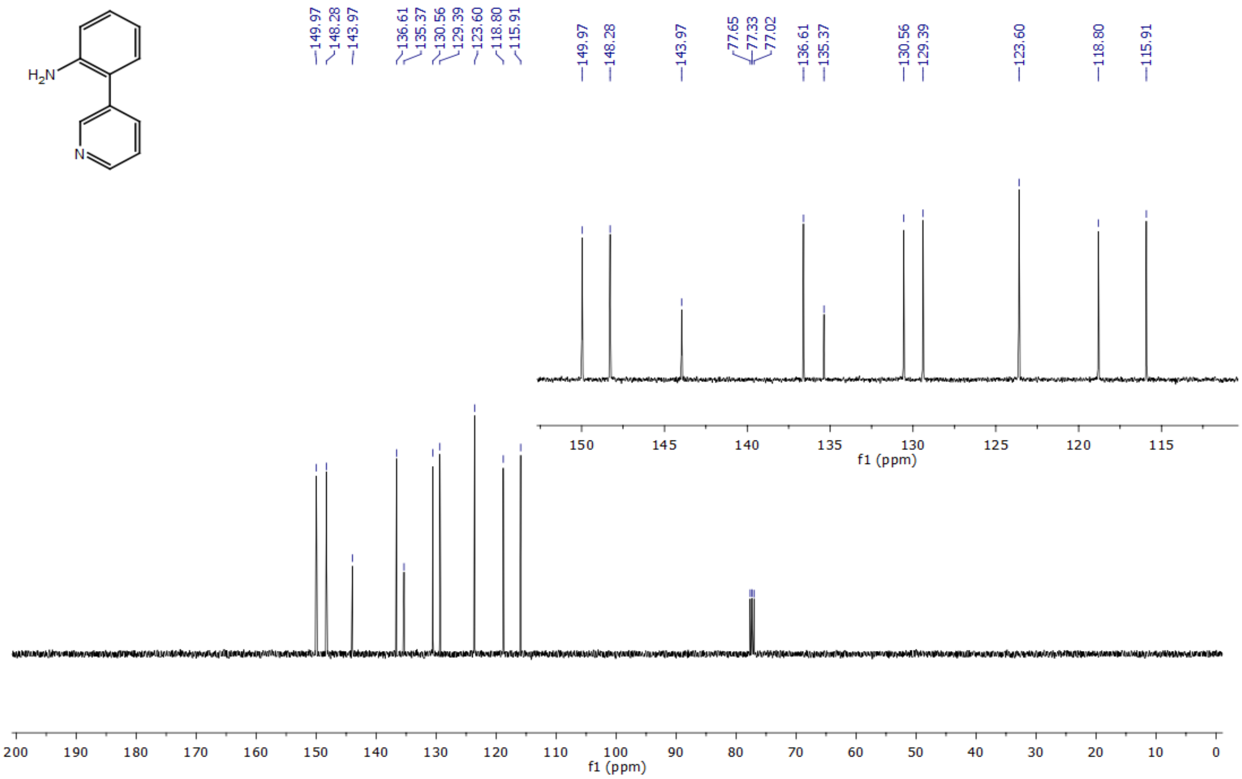

2-(5-methoxypyridin-3-yl)aniline **(TDG4)**

^{1}H NMR

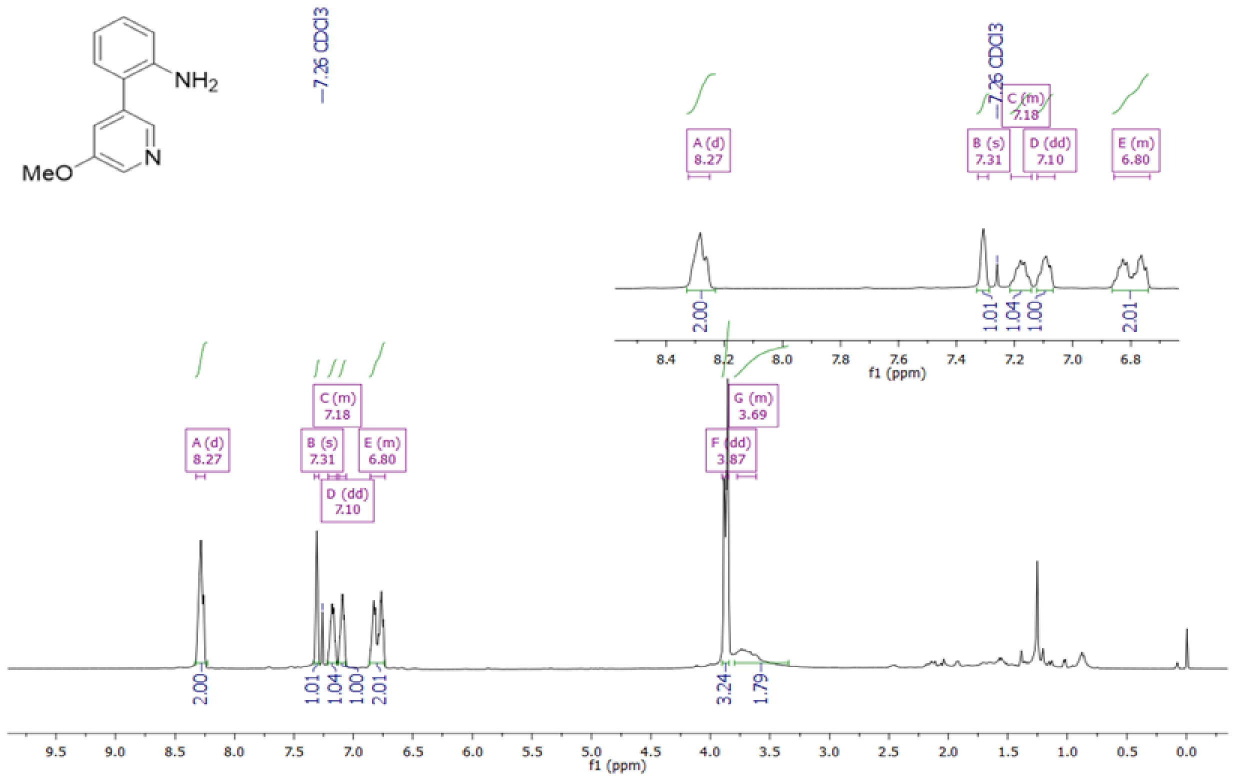

^{13}C NMR

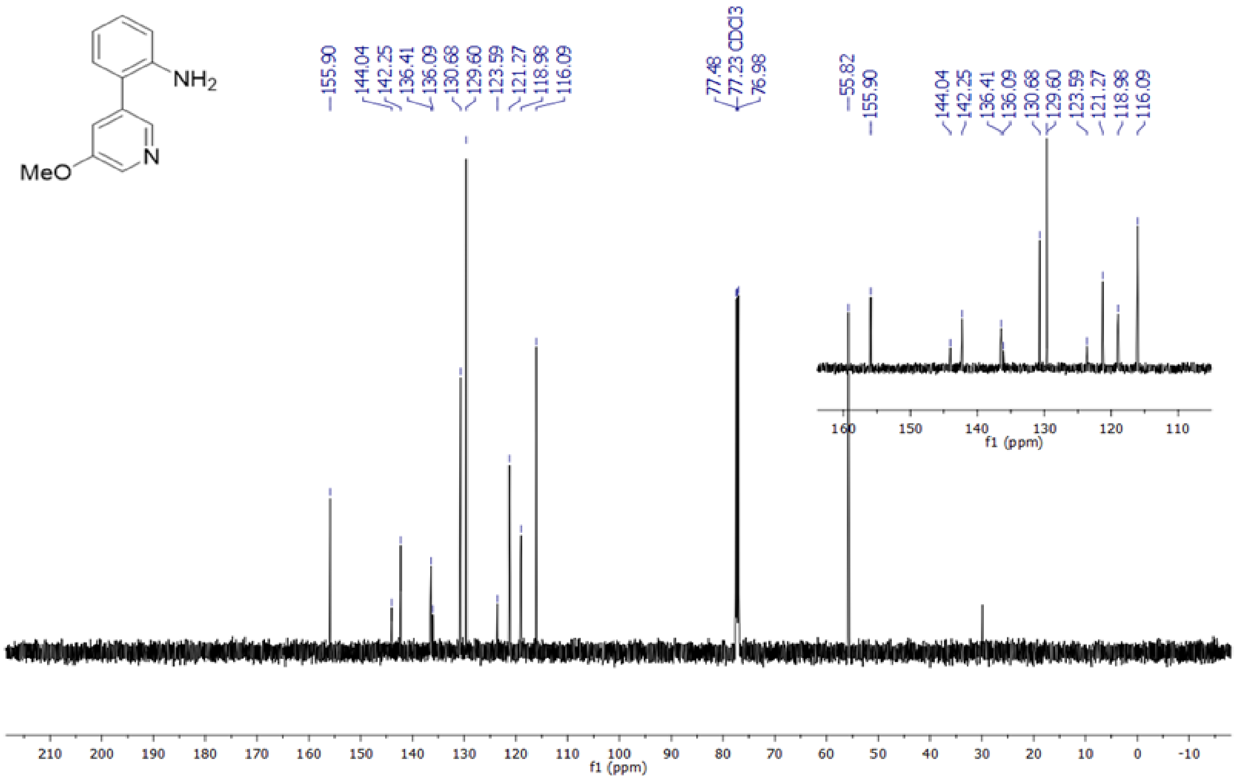

2-(2-fluoropyridin-3-yl)aniline **(TDG5)**

^{1}H NMR

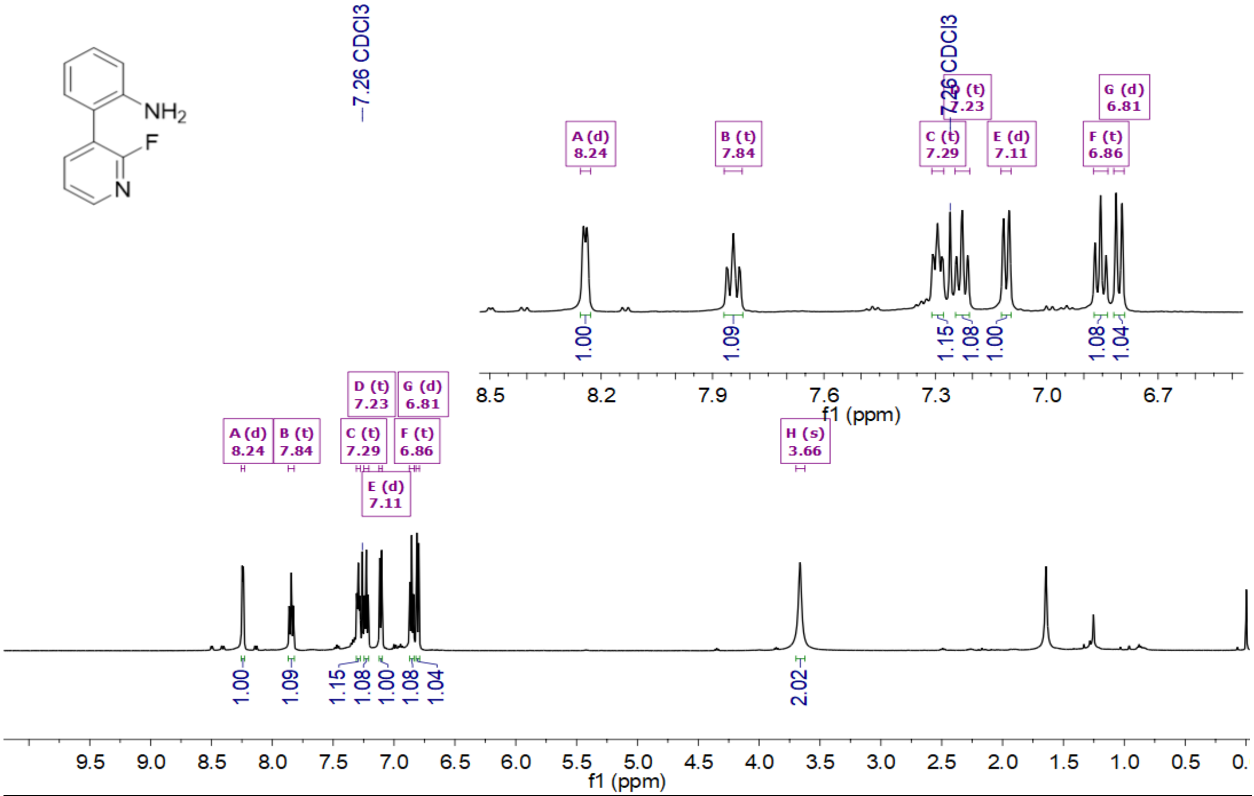

^{13}C NMR

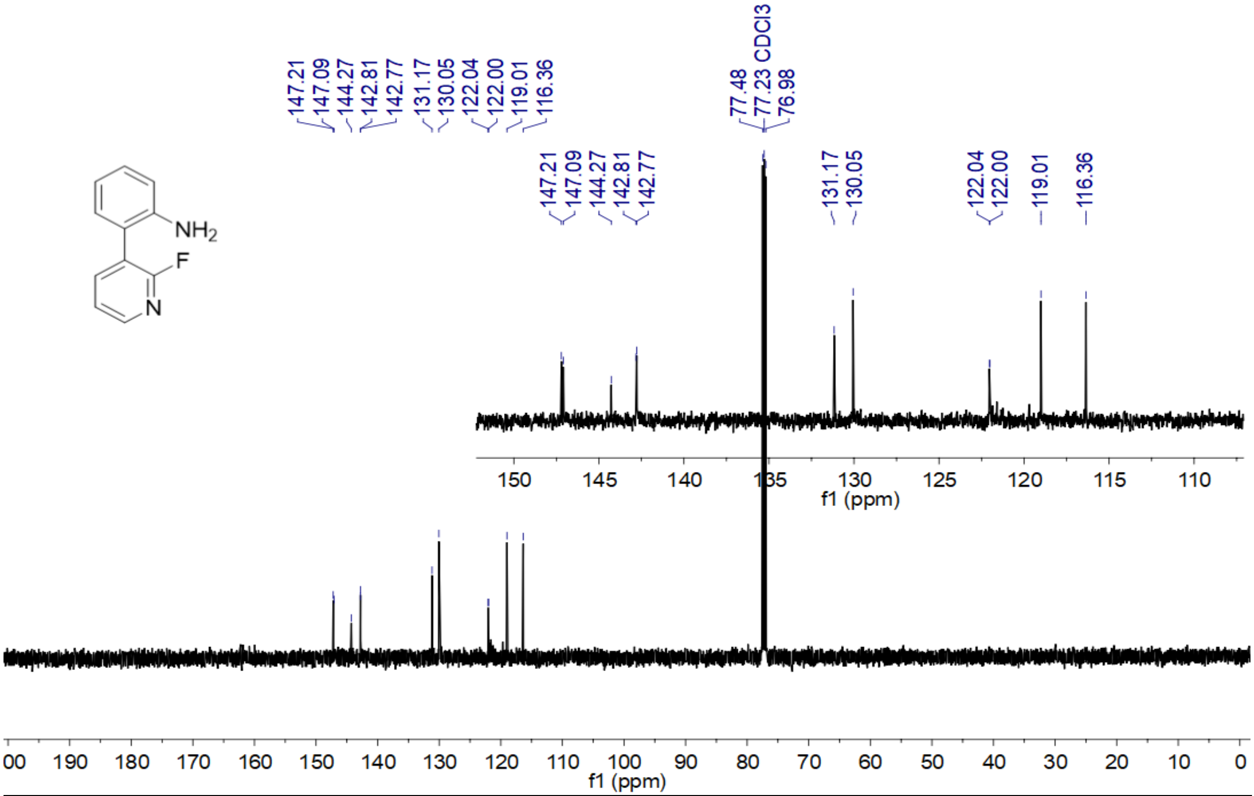

2-(6-fluoropyridin-3-yl)aniline **(TDG6)**

^{1}H NMR

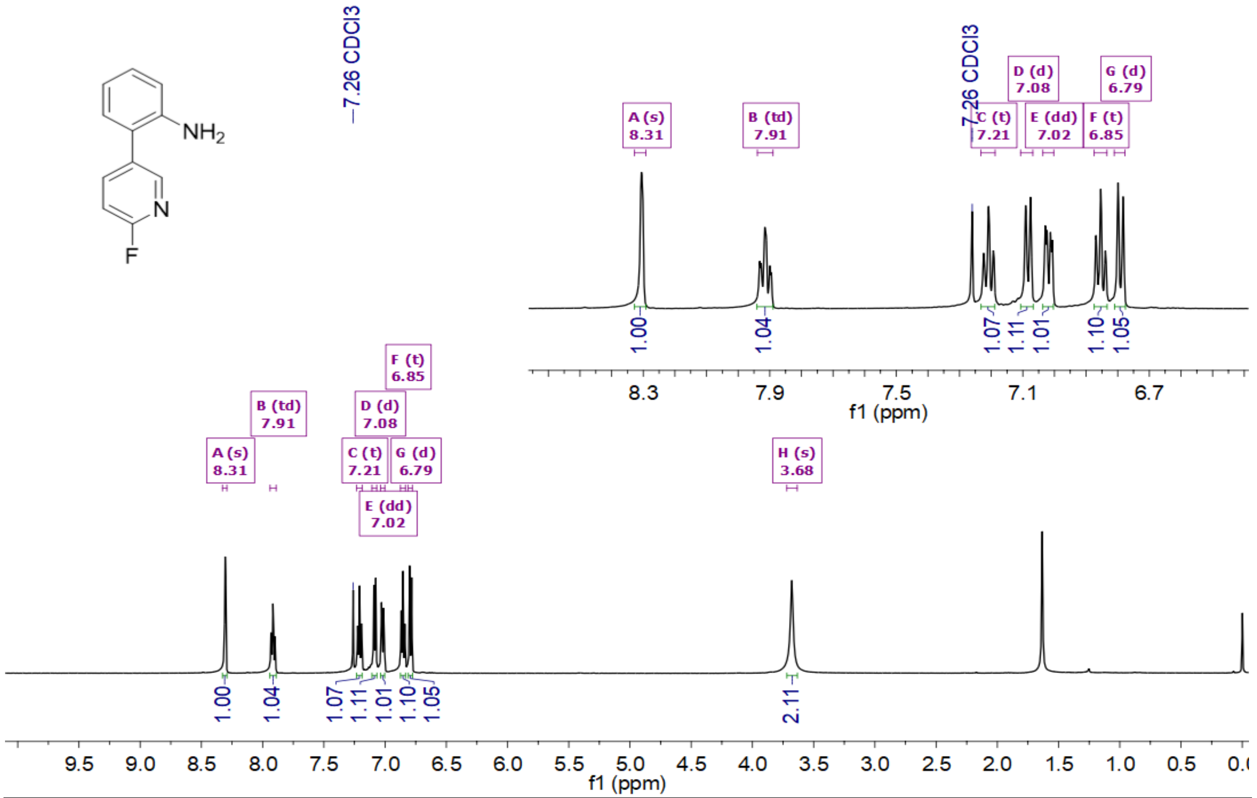

^{13}C NMR

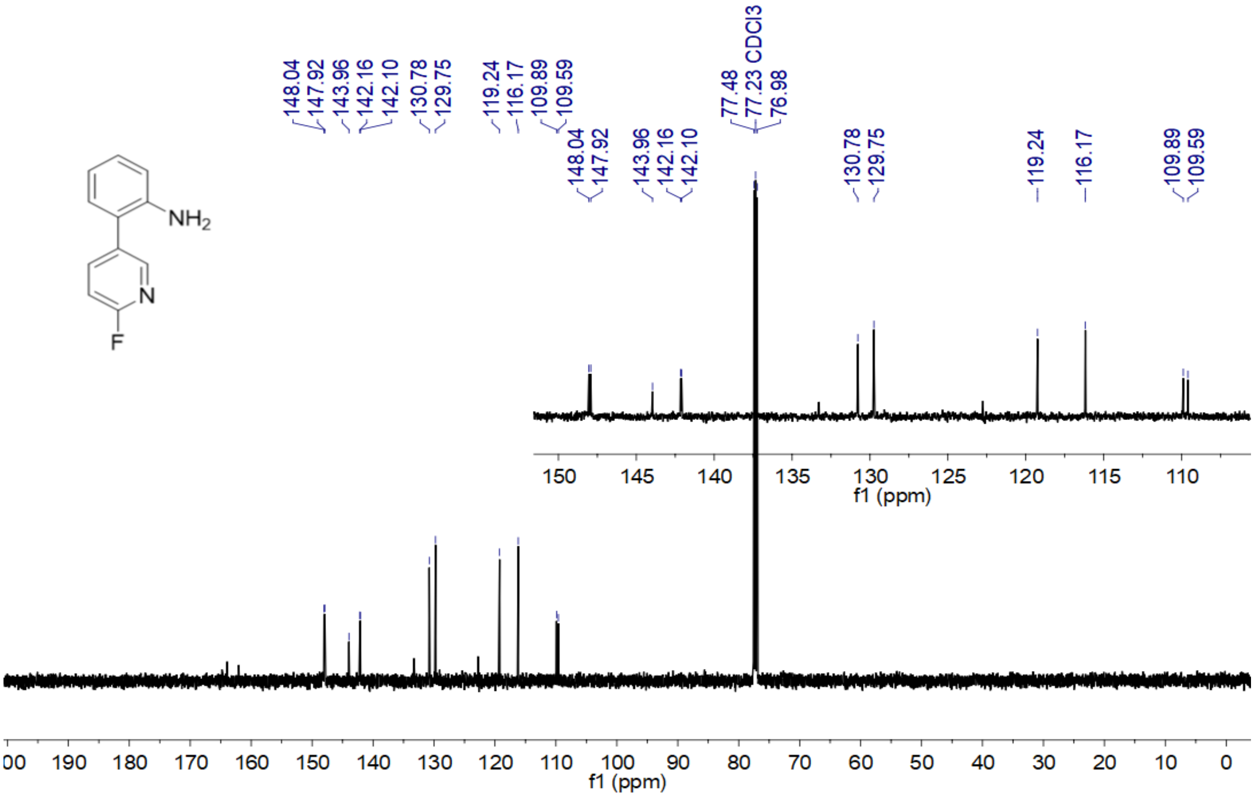

***2-(2-fluoro-5-methylpyridin-3-yl)aniline* (TDG7)**

^{1}H NMR

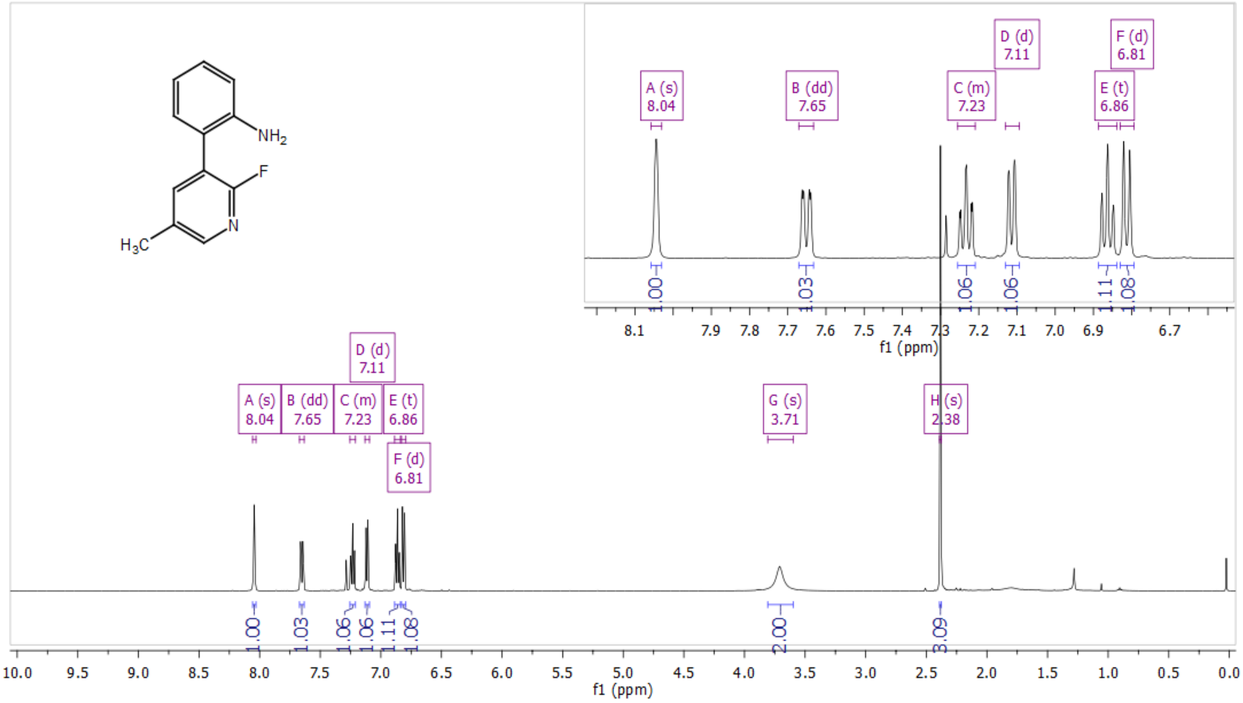

^{13}C NMR

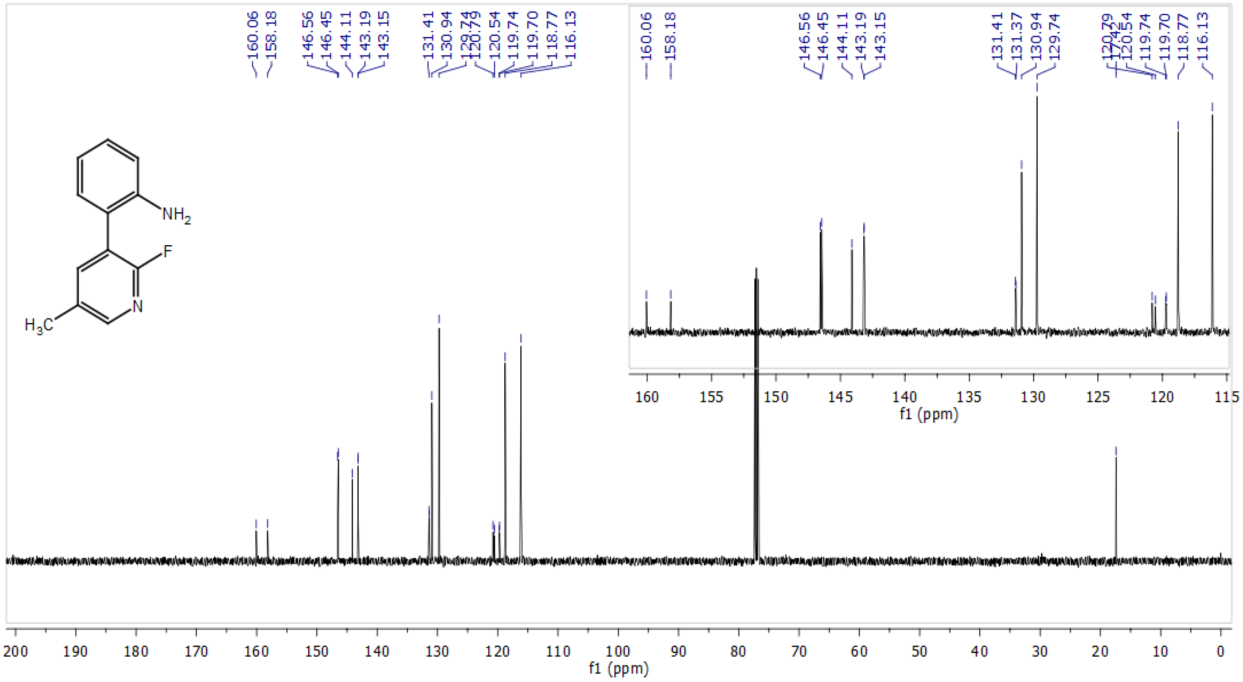

2-(6-chloropyridin-3-yl)aniline **(TDG8)**

^{1}H NMR

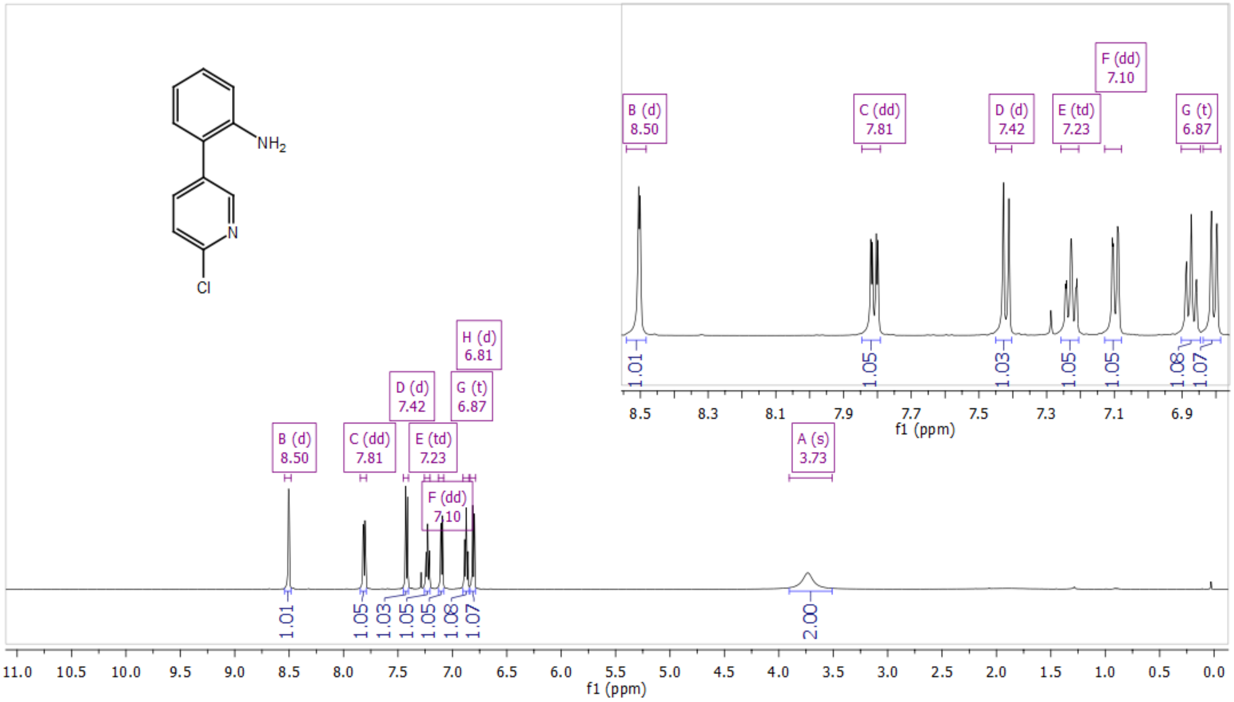

^{13}C NMR

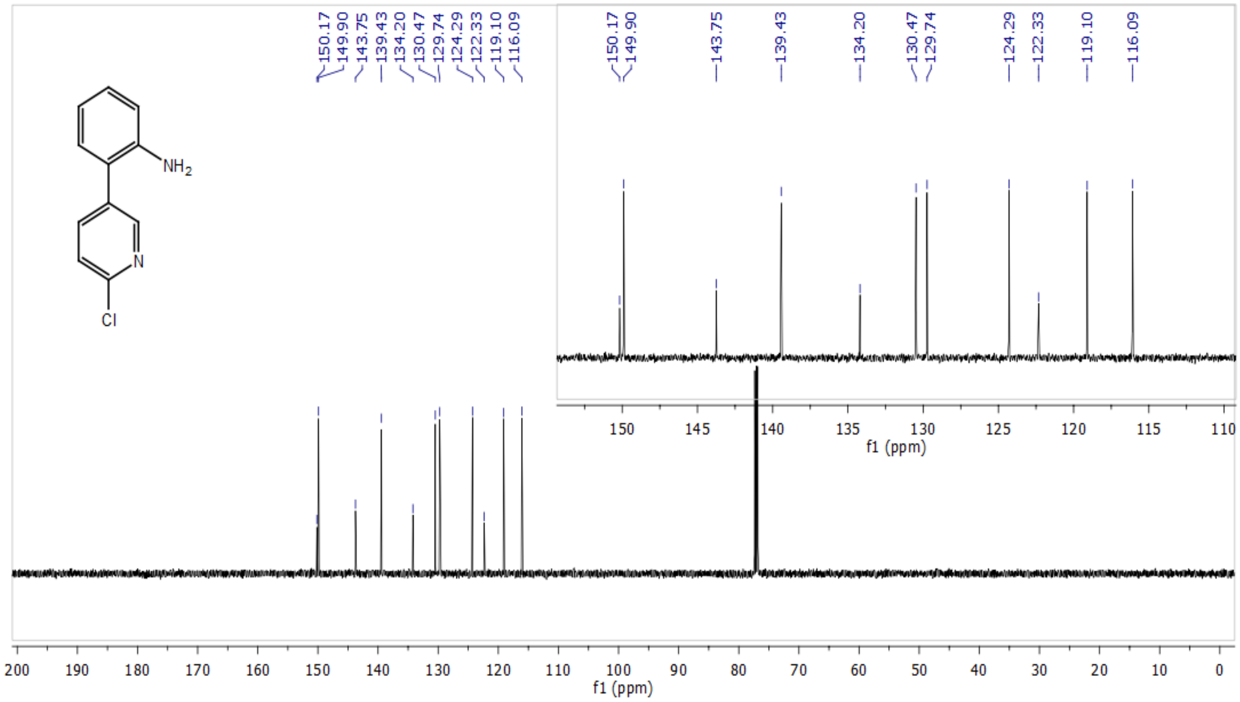

5-(2-aminophenyl)picolinonitrile **(TDG 9)**

^{1}H NMR

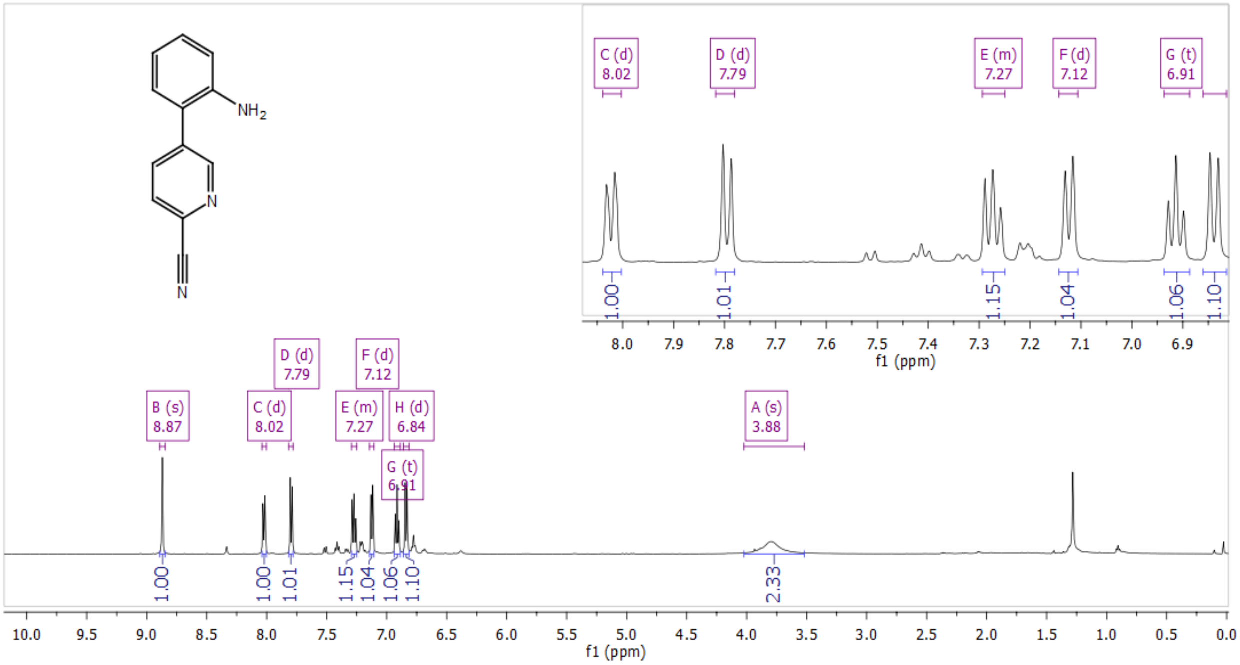

^{13}C NMR

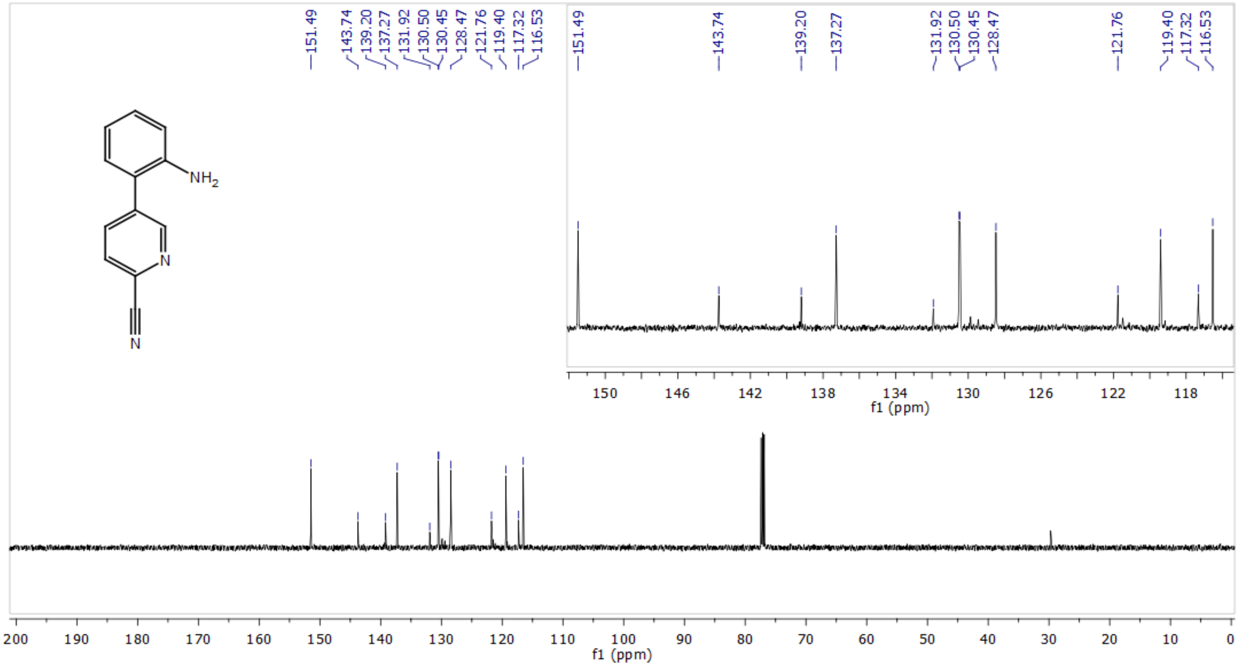

***Methyl-5-(2-aminophenyl)picolinate* (TDG10)**
^{1}H NMR

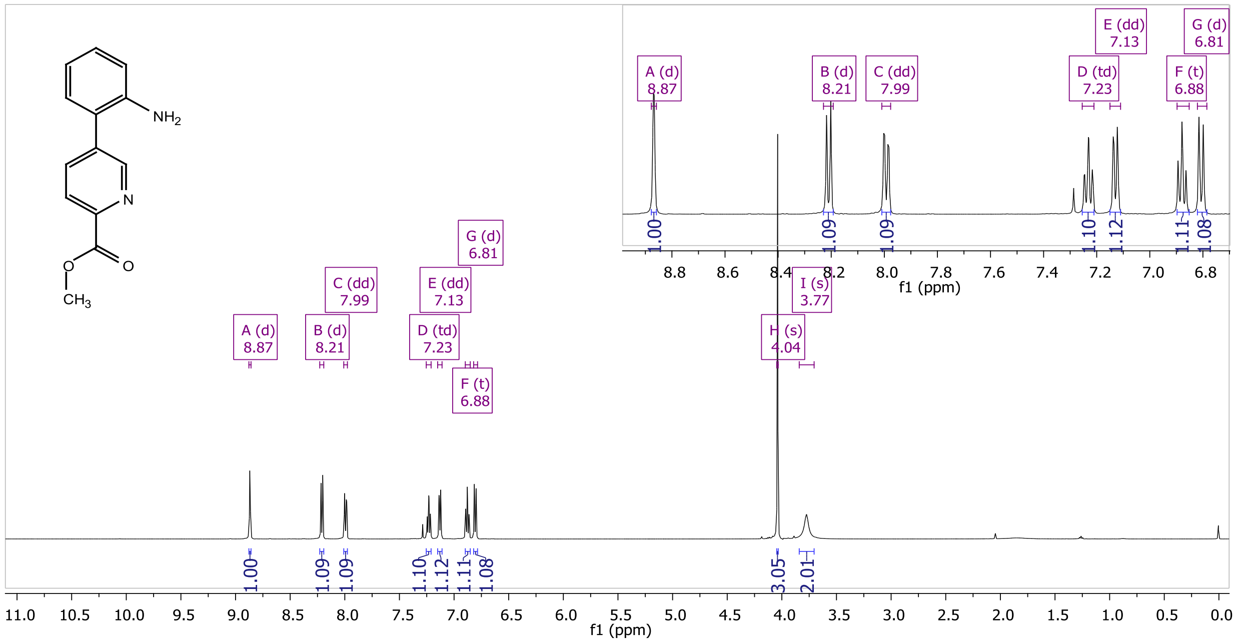

^{13}C NMR

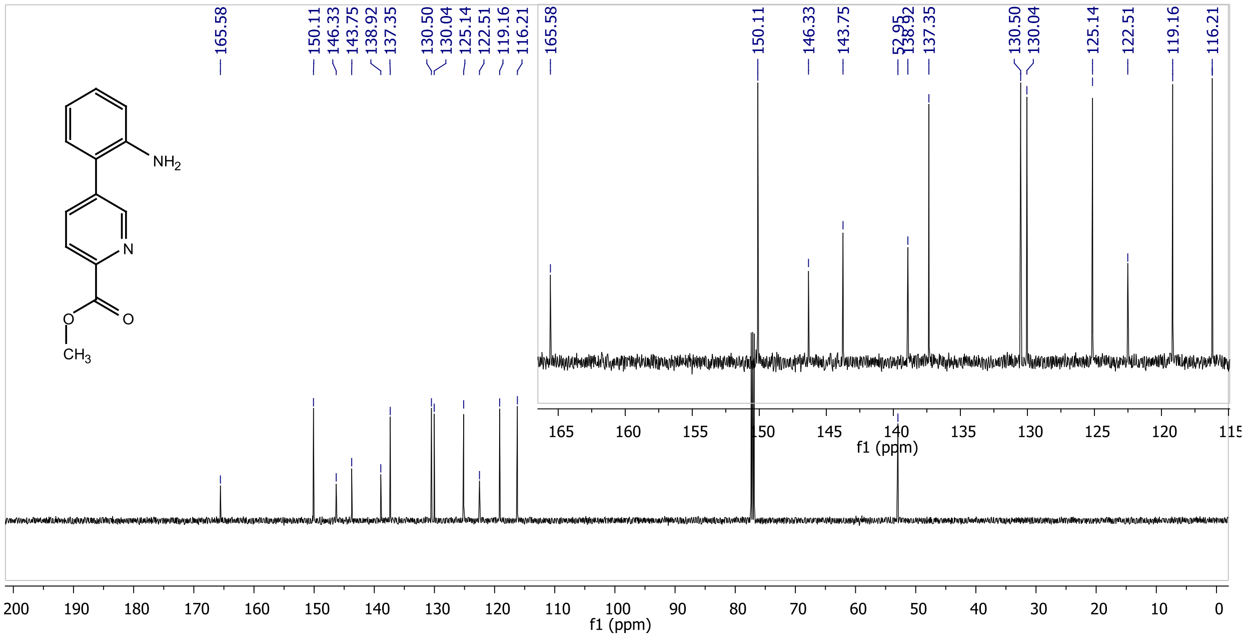

Isopropyl 5-(2-aminophenyl)picolinate **(TDG11)**

^{1}H NMR

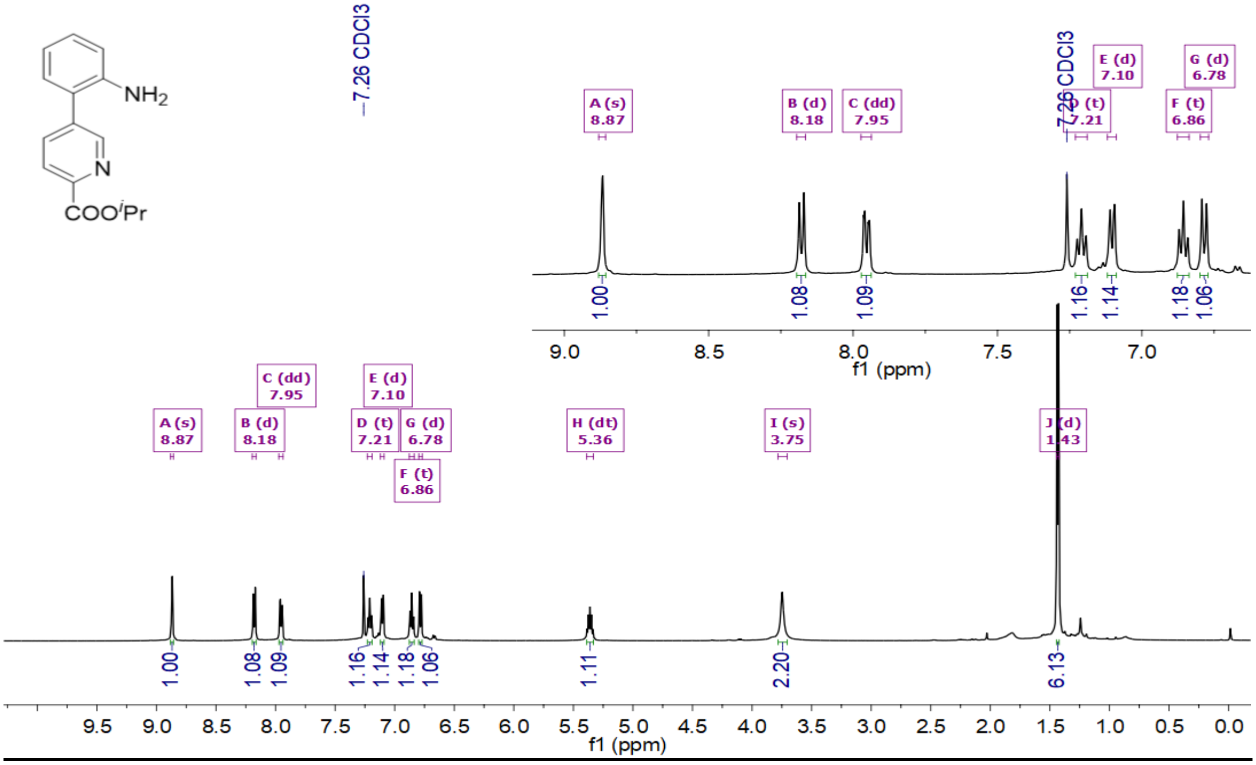

^{13}C NMR

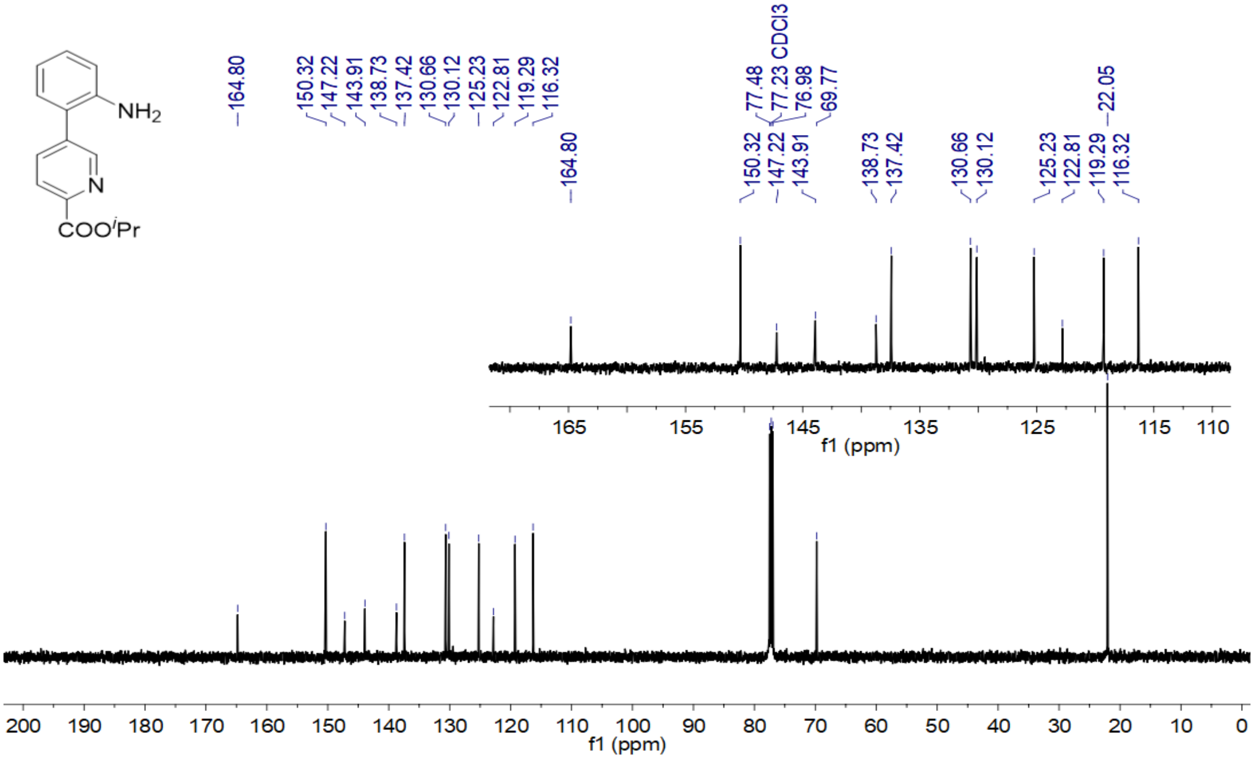

***2-(quinolin-3-yl)aniline* (TDG12)**

^{1}H NMR

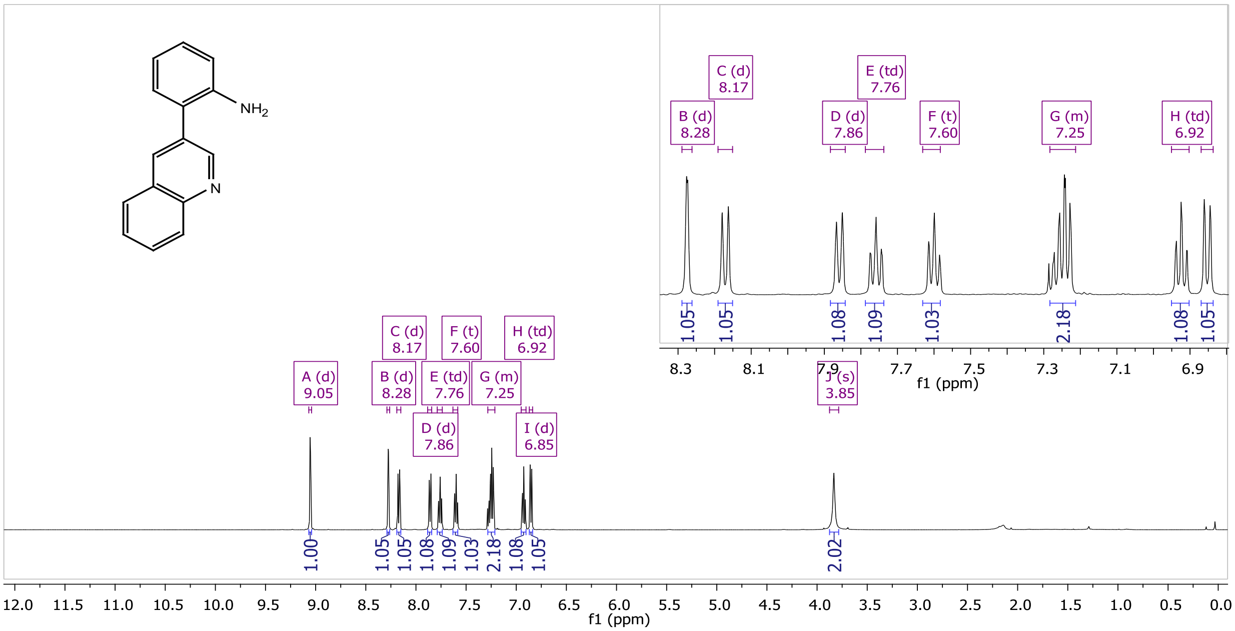

^{13}C NMR

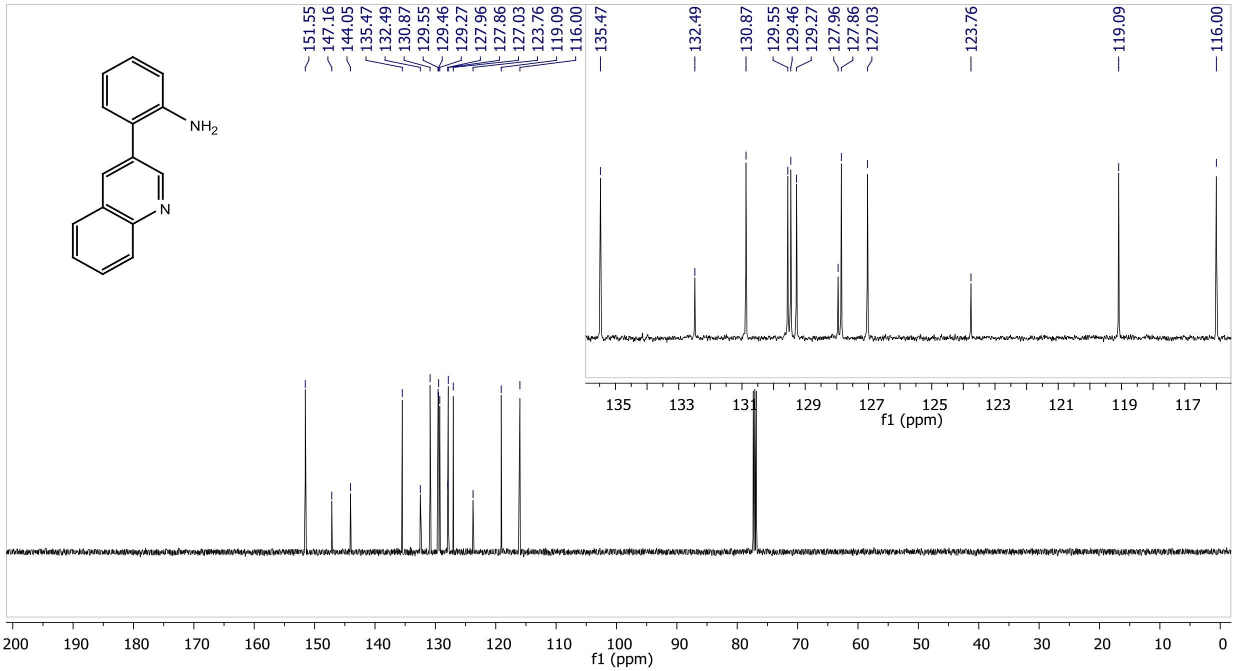

2-(8-nitroquinolin-3-yl)aniline **(TDG13)**

^{1}H NMR

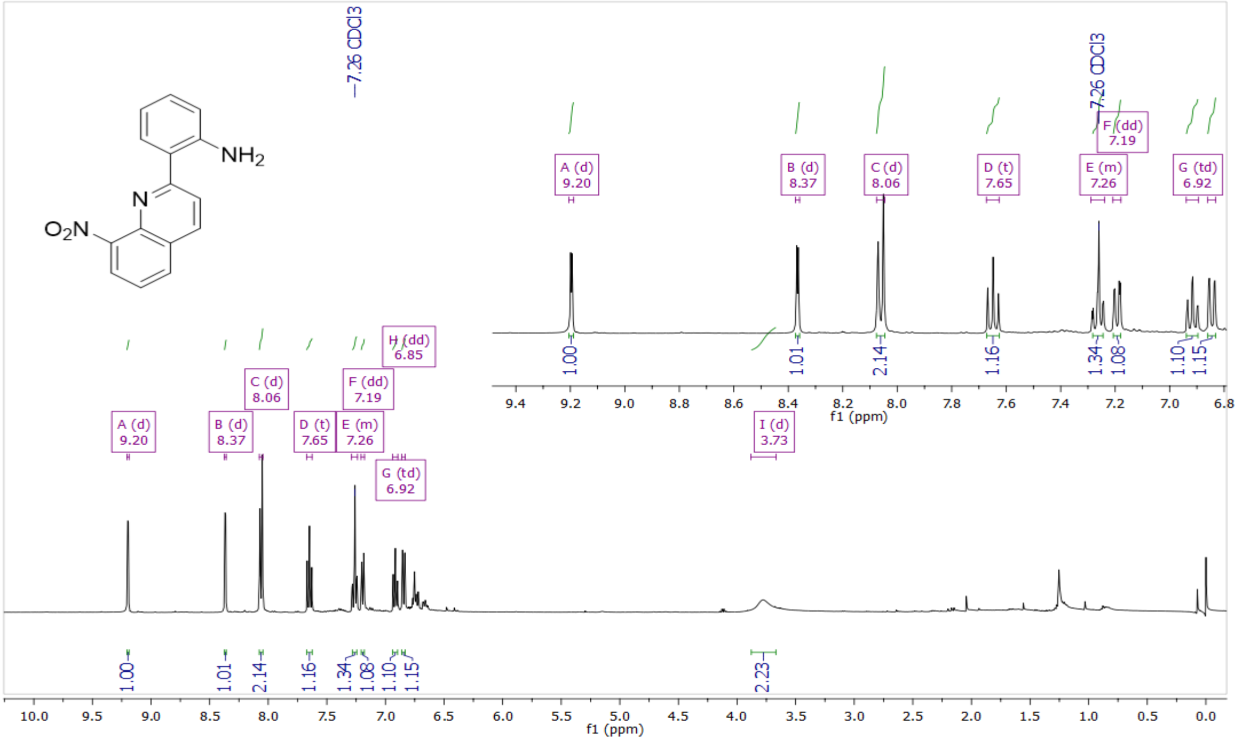

^{13}C NMR

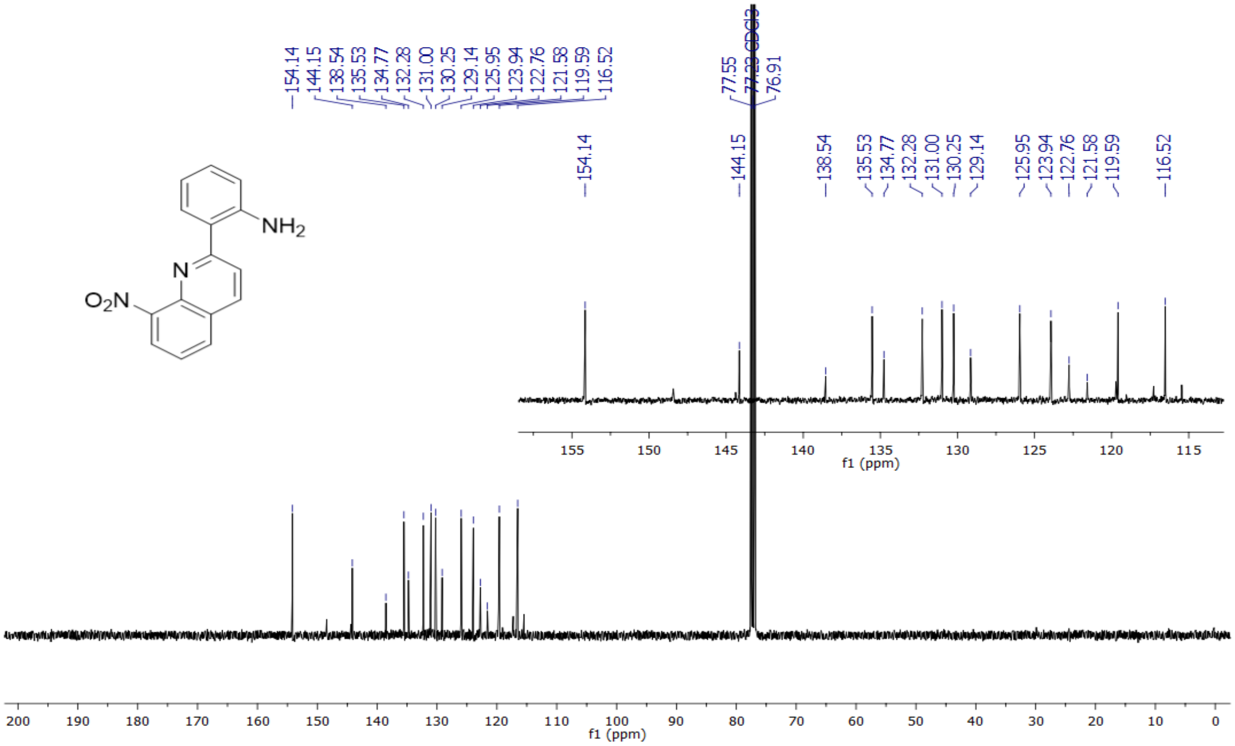

***2-fluoro-6-(pyridin-3-yl)aniline* (TDG14)**

^{1}H NMR

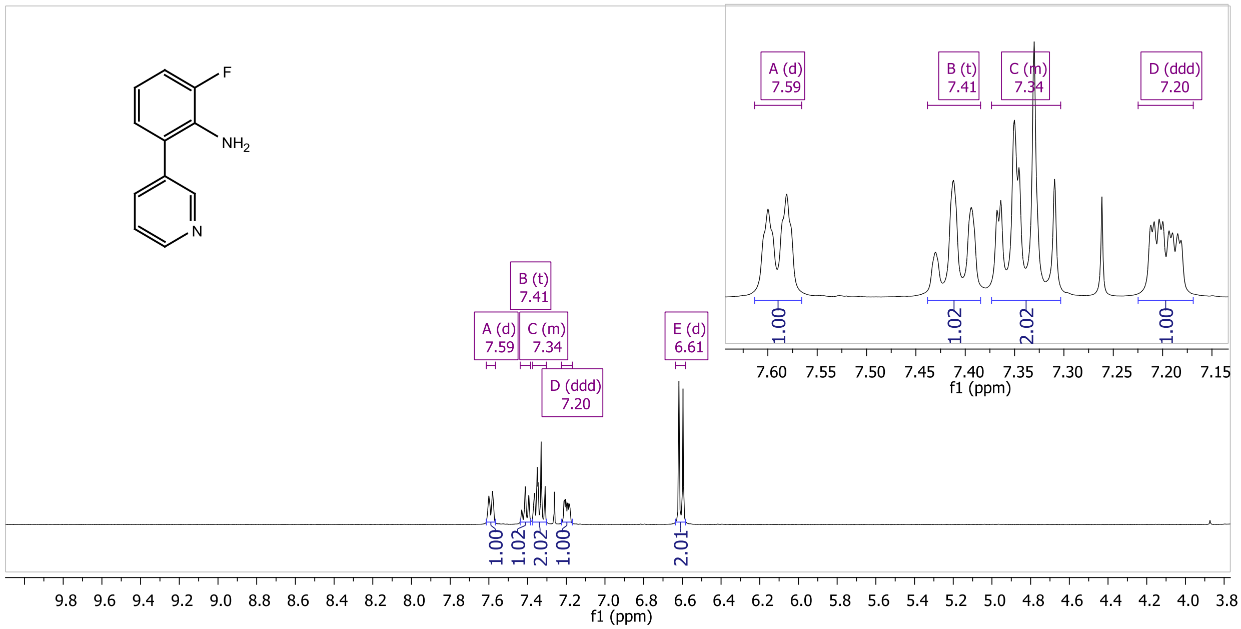

^{13}C NMR

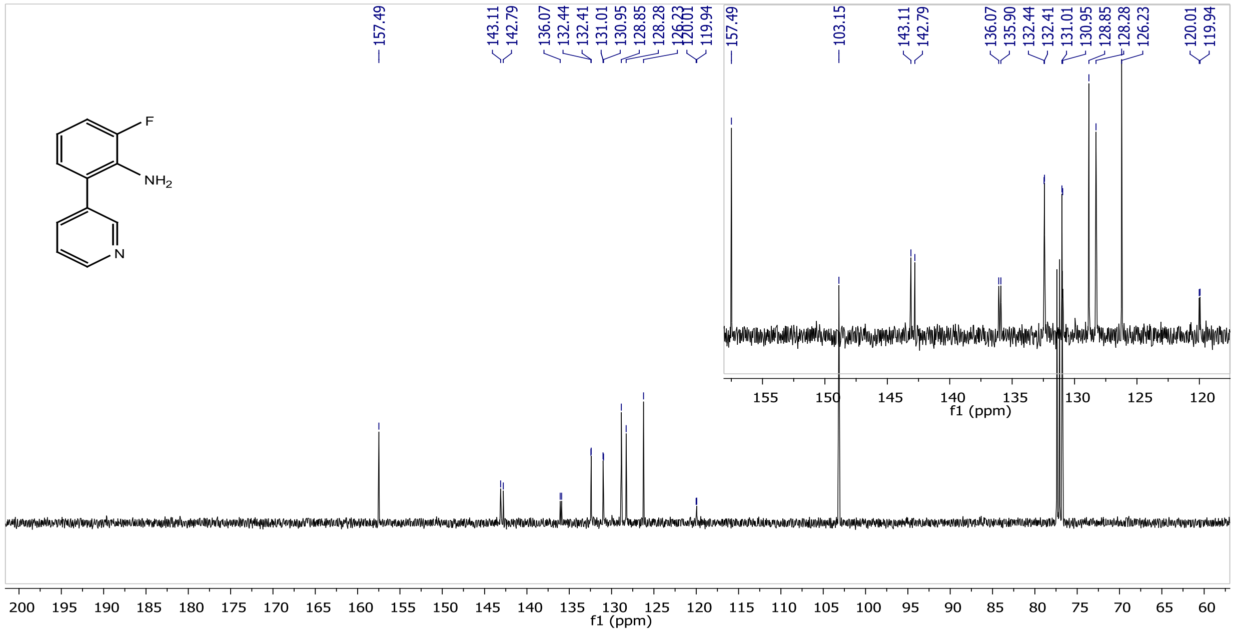

2-fluoro-6-(2-fluoropyridin-3-yl)aniline **(TDG15)**

^{1}H NMR

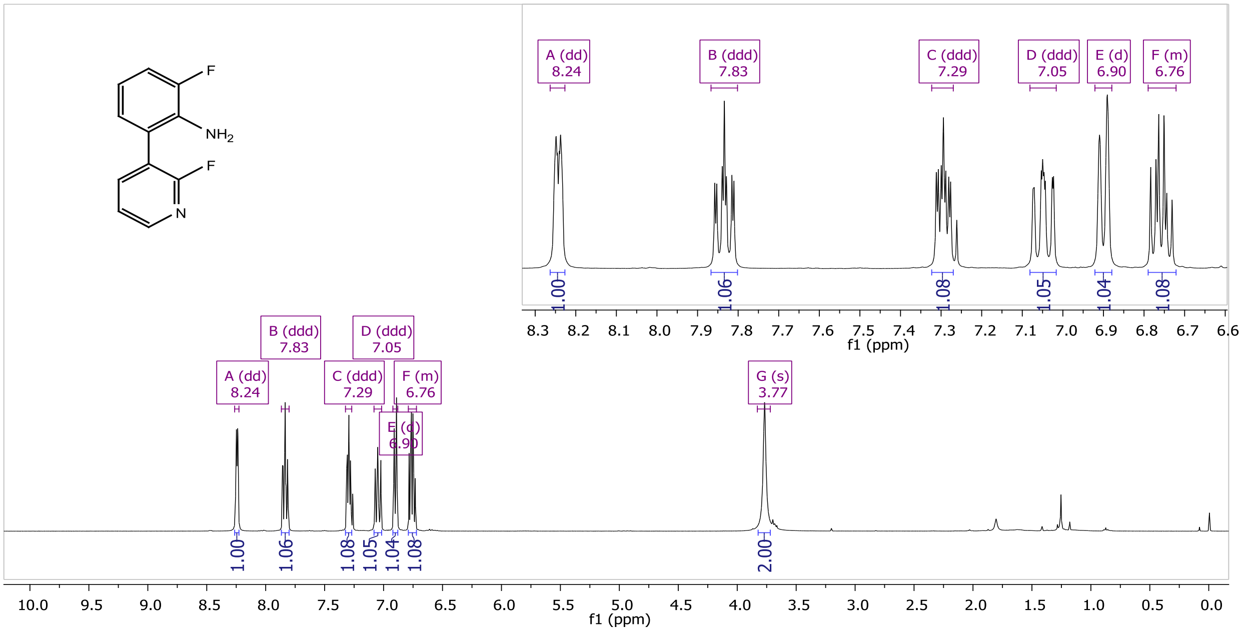

^{13}C NMR

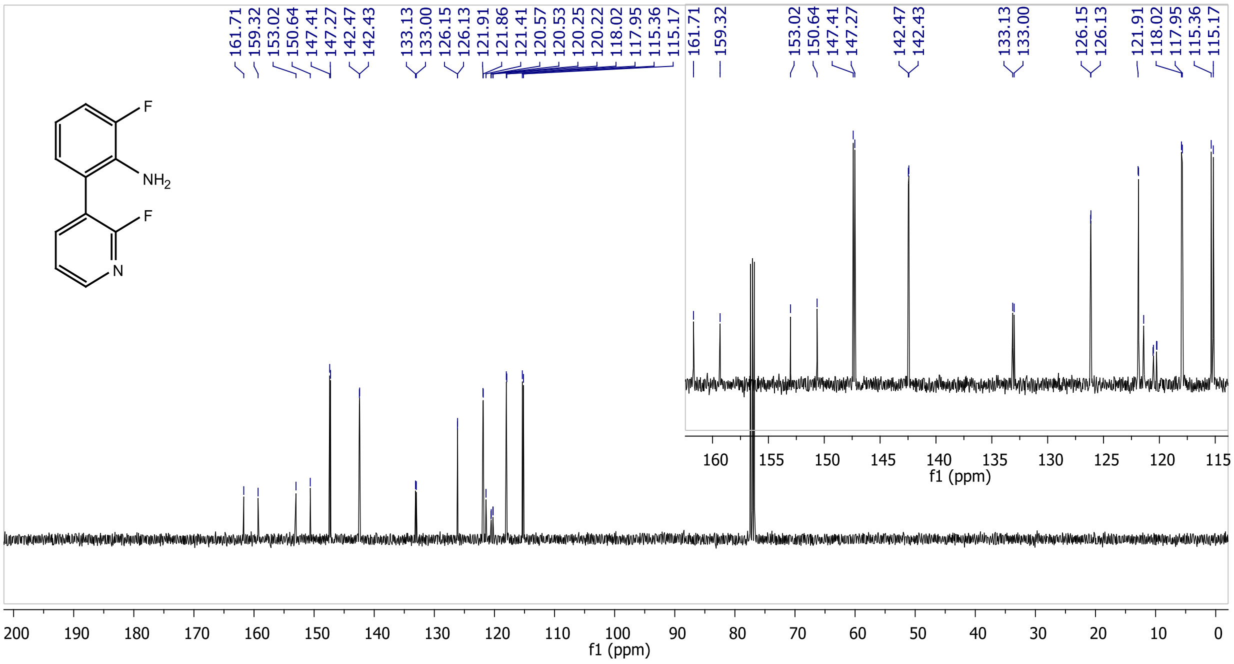

***2-(pyrimidin-5-yl)aniline* (TDG16)**

^{1}H NMR

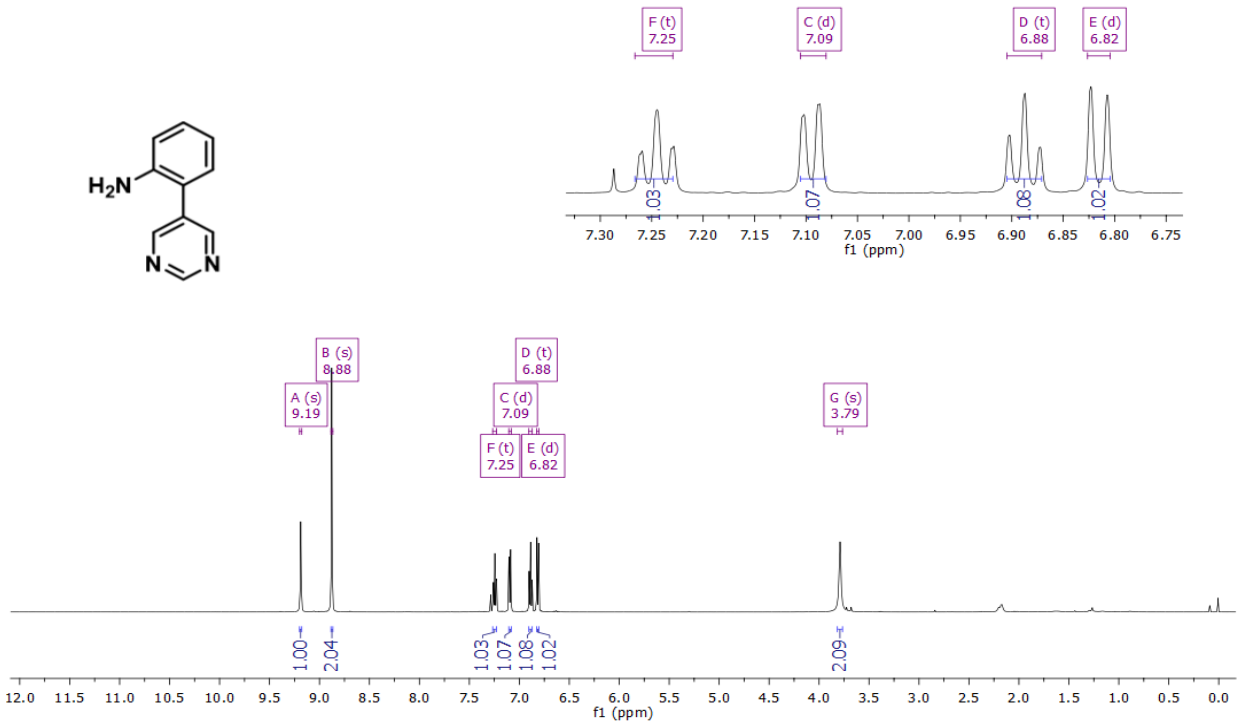

^{13}C NMR

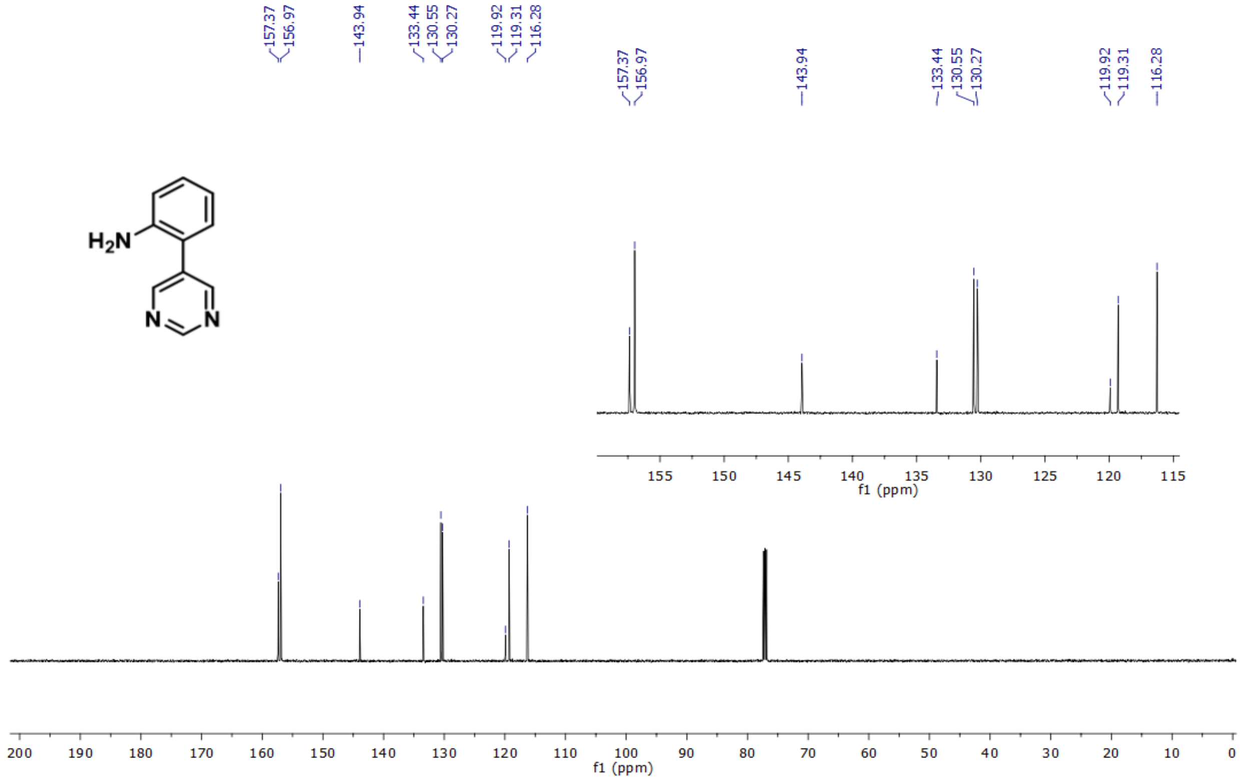

2,4-dimethyl-6-(pyrimidin-5-yl)aniline **(TDG17)**

^{1}H NMR

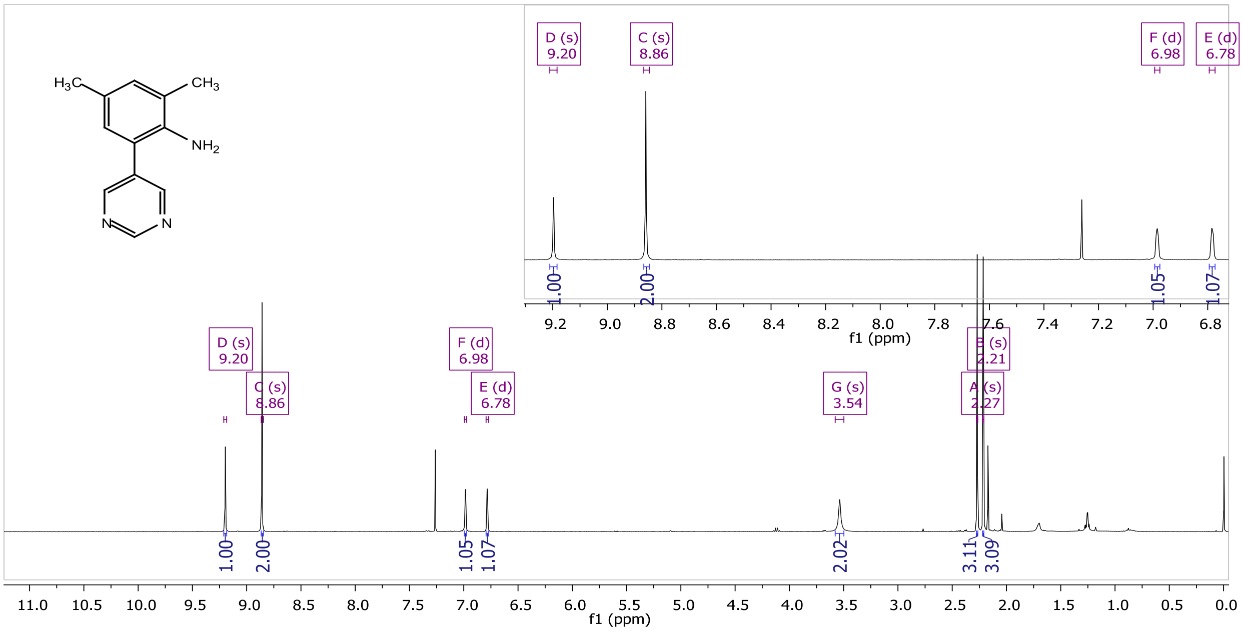

^{13}C NMR

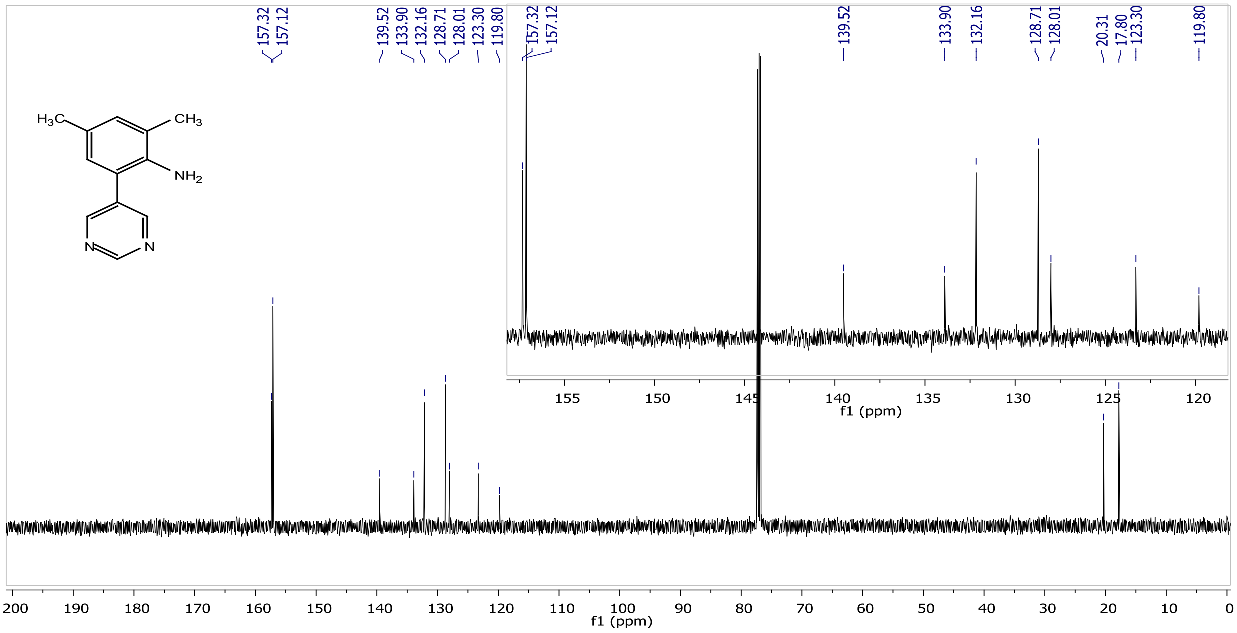

4-chloro-2-(pyrimidin-5-yl)-6-(trifluoromethyl)aniline **(TDG18)**

^{1}H NMR

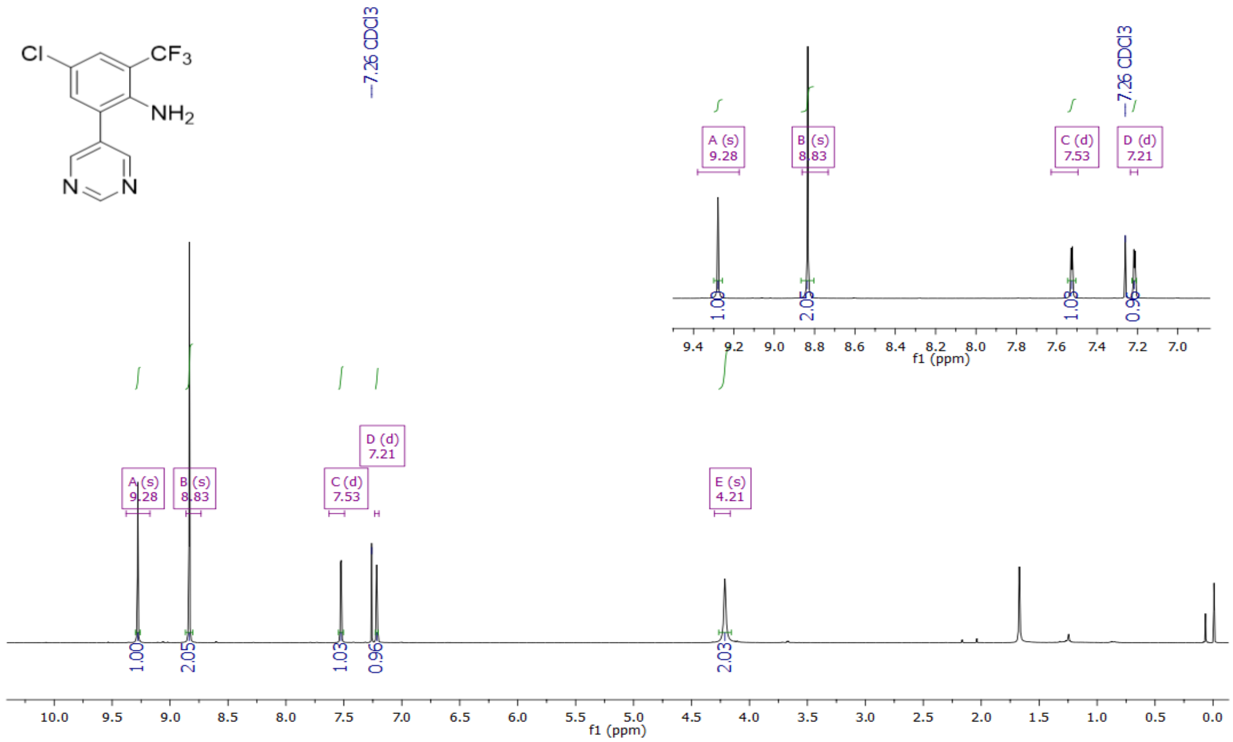

^{13}C NMR

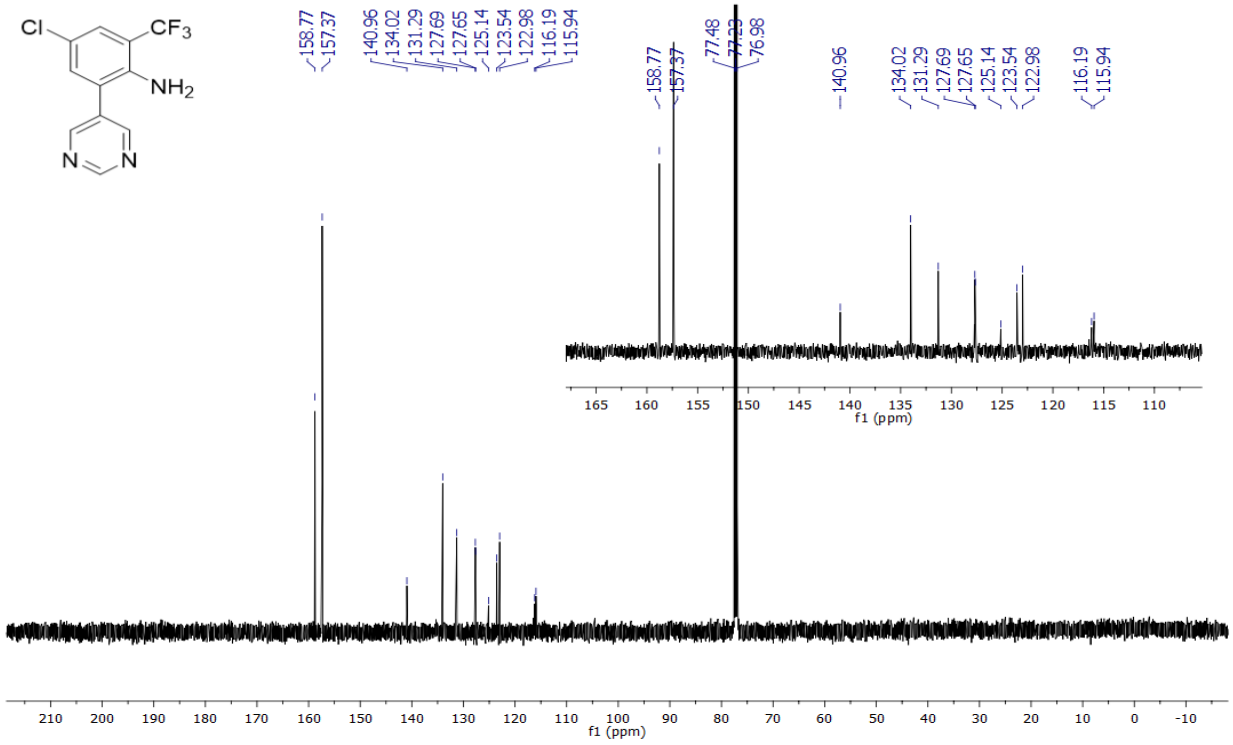

2-fluoro-6-(pyrimidin-5-yl)aniline (TDG19)

^{1}H NMR

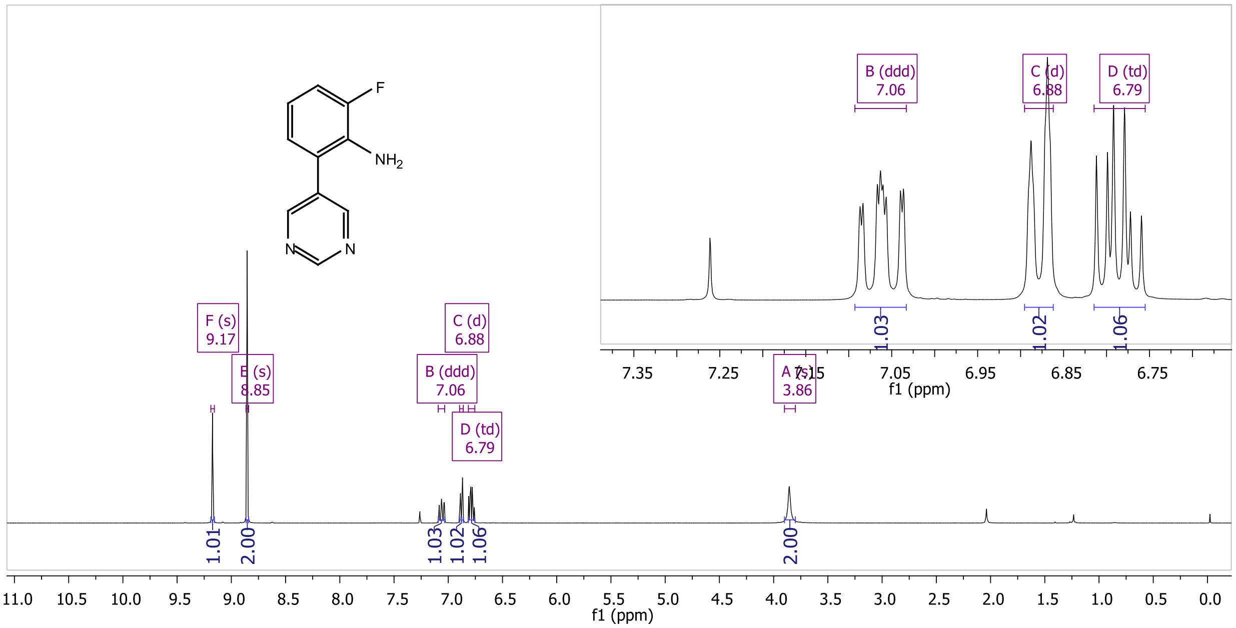

^{13}C NMR

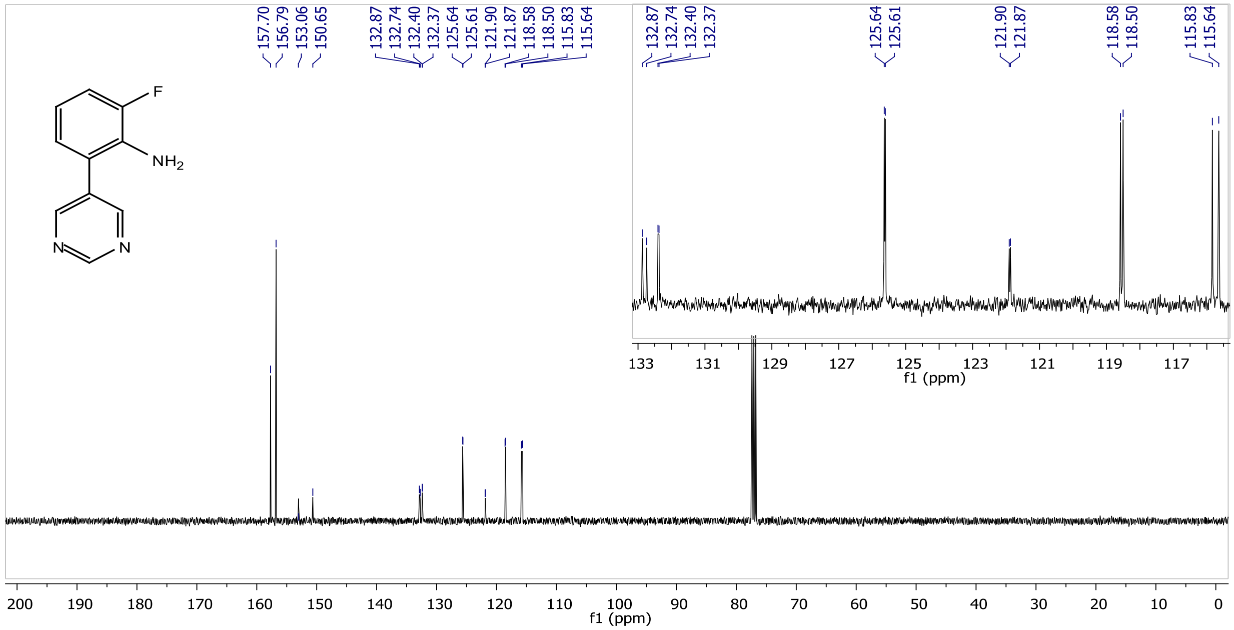

2-(pyrimidin-5-yl)benzaldehyde **(TDG20)**

^{1}H NMR

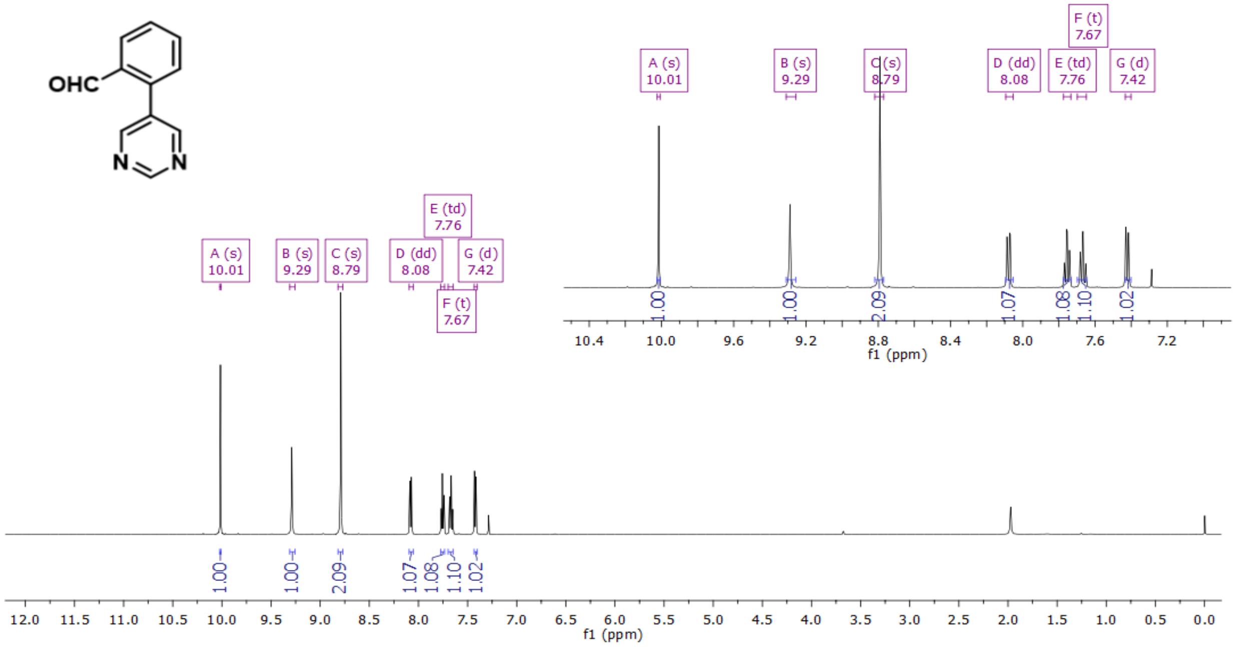

^{13}C NMR

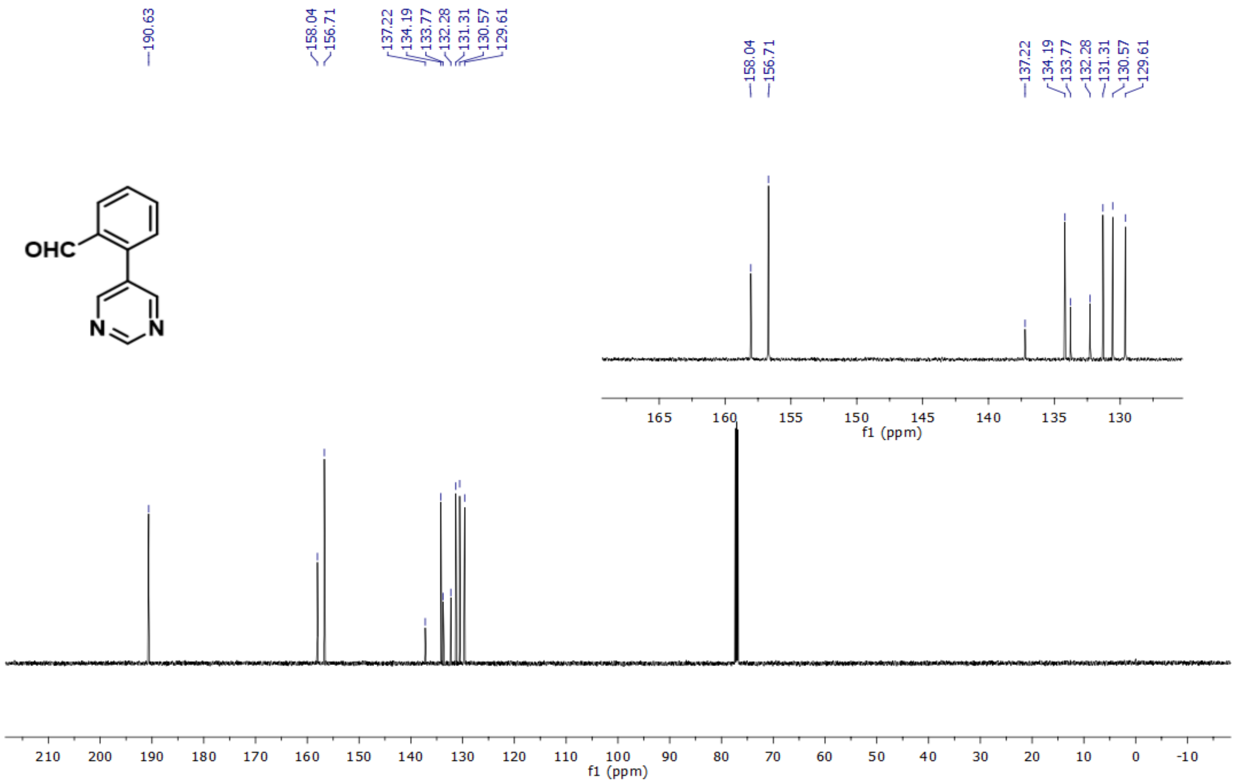

2-phenylbenzyldehyde derivatives

***3'-methyl-[1,1'-biphenyl]-2-carbaldehyde* (1a)**

^{1}H NMR

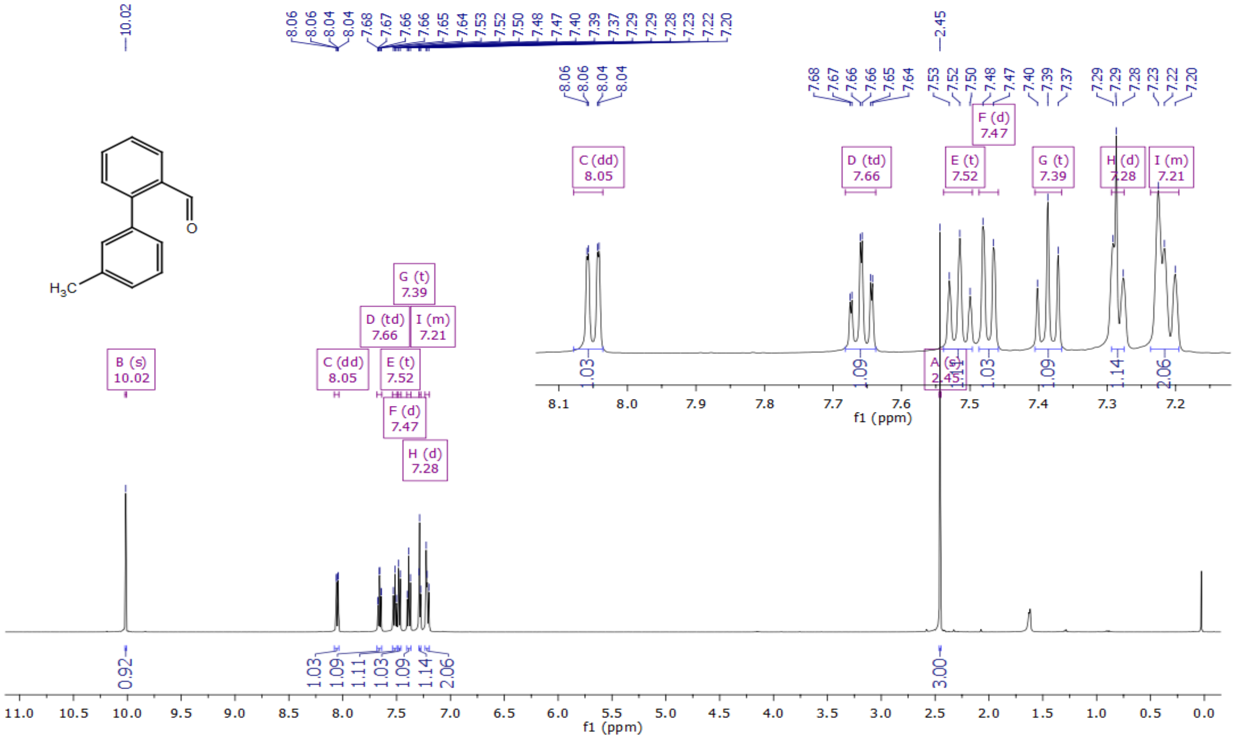

^{13}C NMR

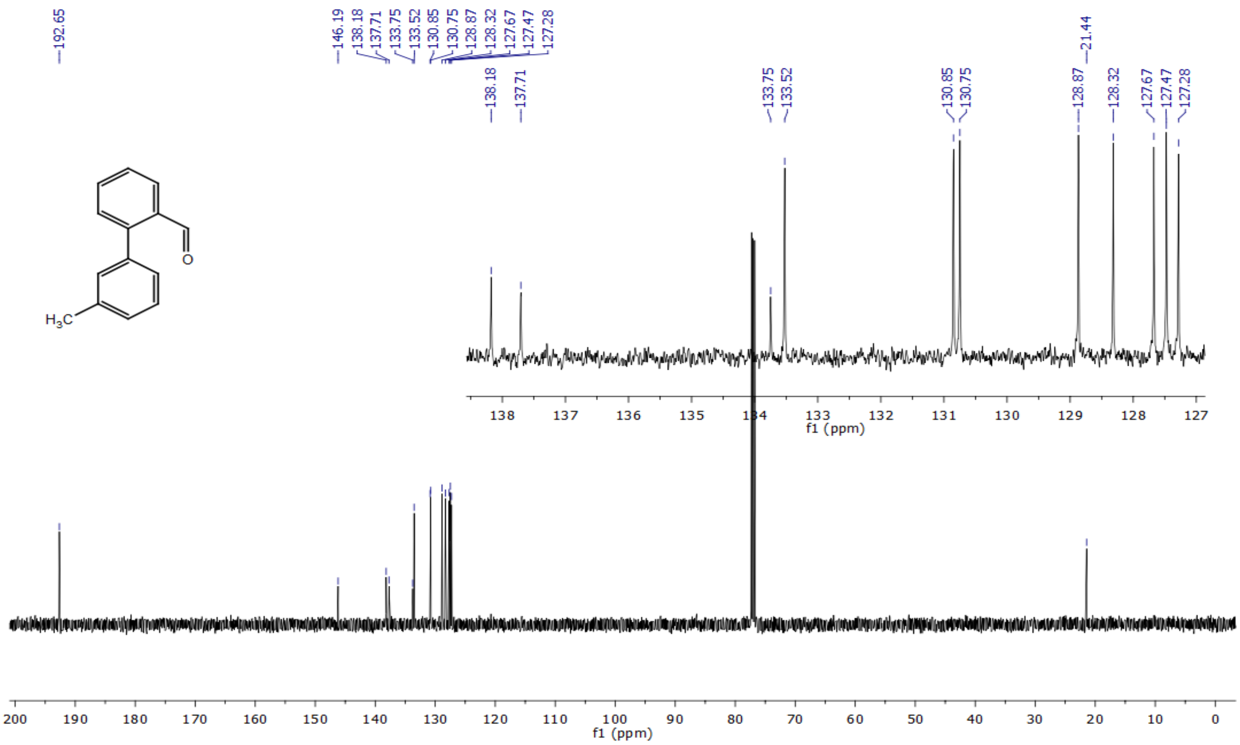

[1,1'-biphenyl]-2-carbaldehyde (1b)

^{1}H NMR

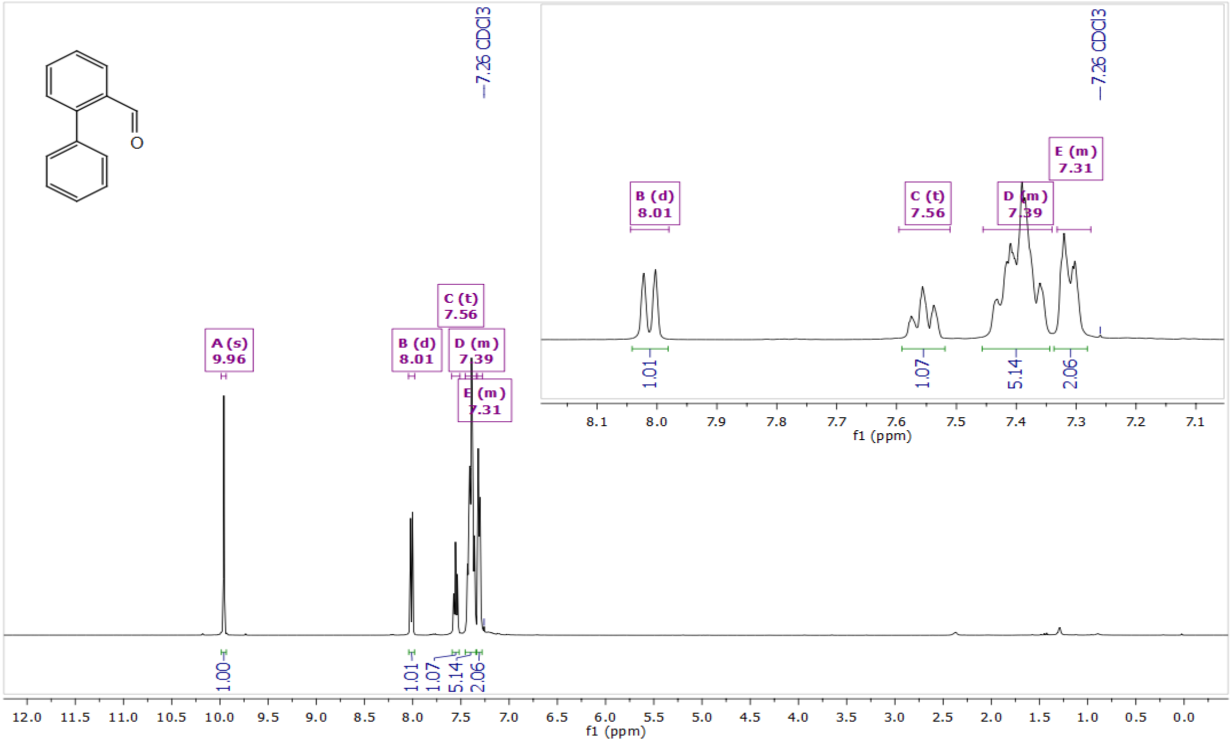

^{13}C NMR

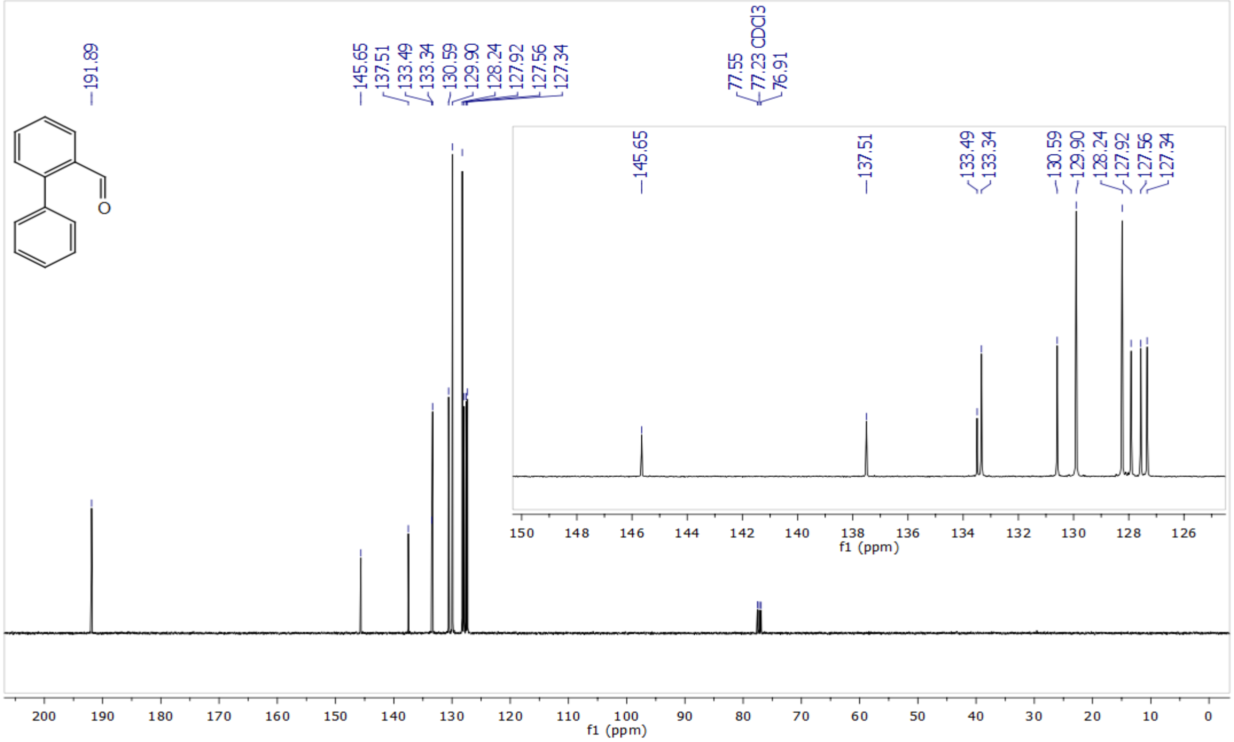

***3'-chloro-[1,1'-biphenyl]-2-carbaldehyde* (1c)**

^{1}H NMR

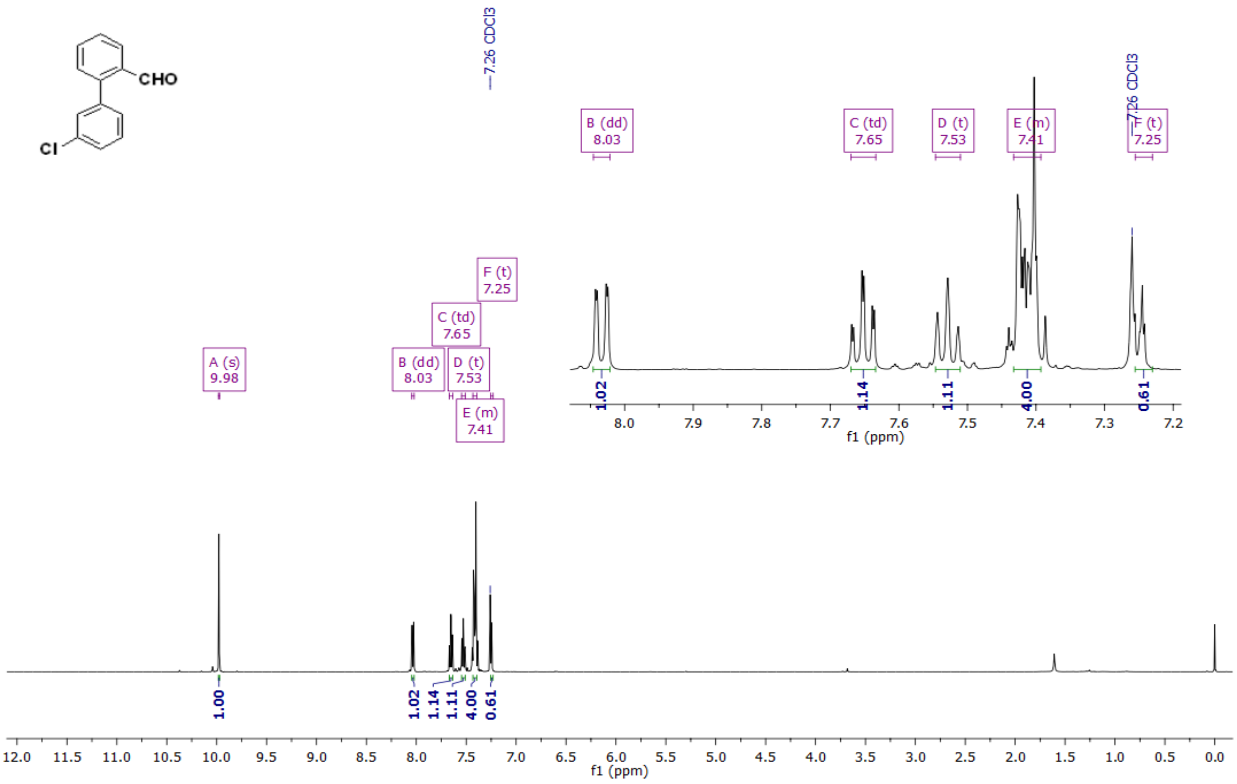

^{13}CNMR

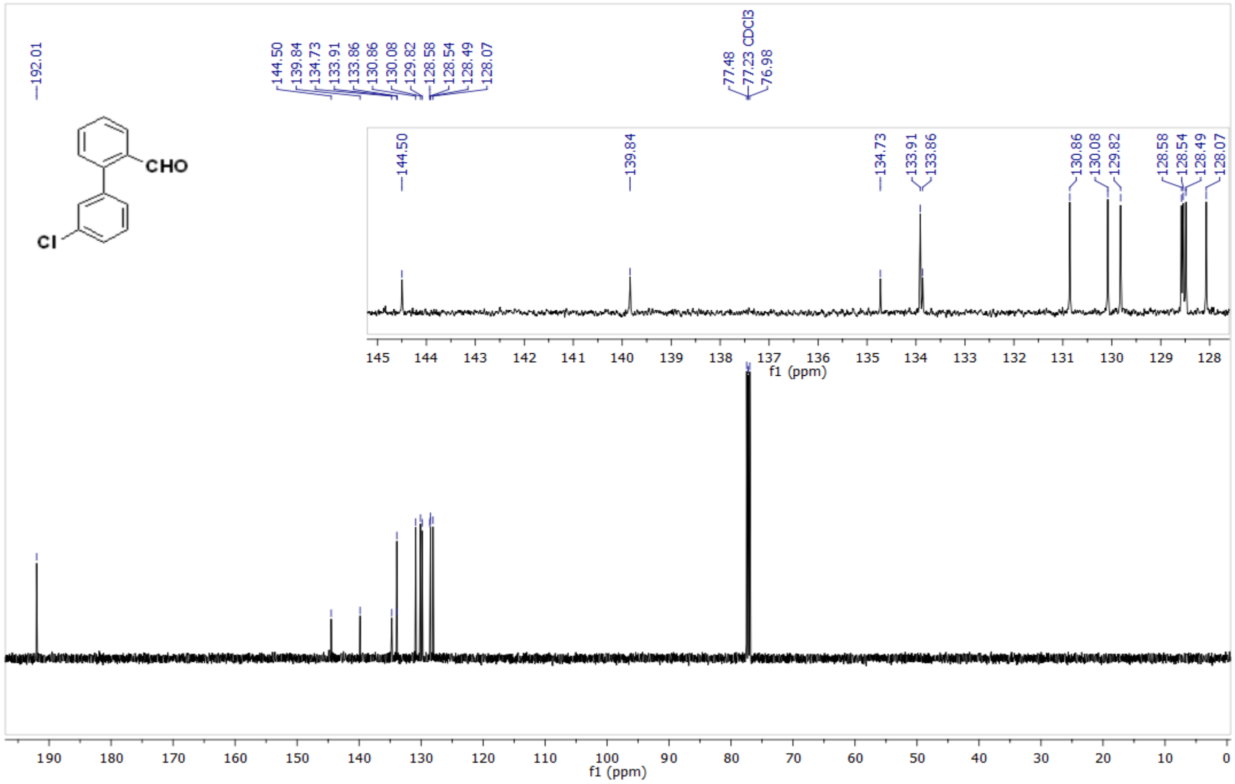

***4'-methyl-[1,1'-biphenyl]-2-carbaldehyde* (1d)**

^{1}H NMR

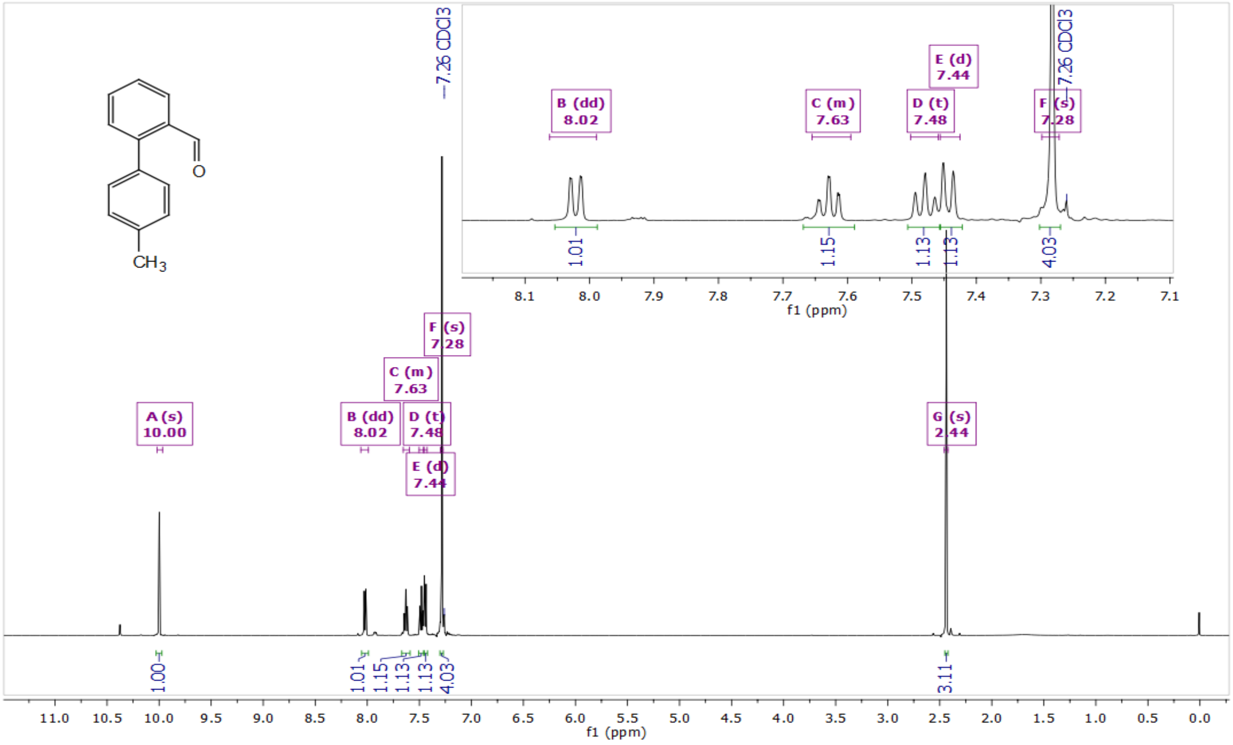

^{13}C NMR

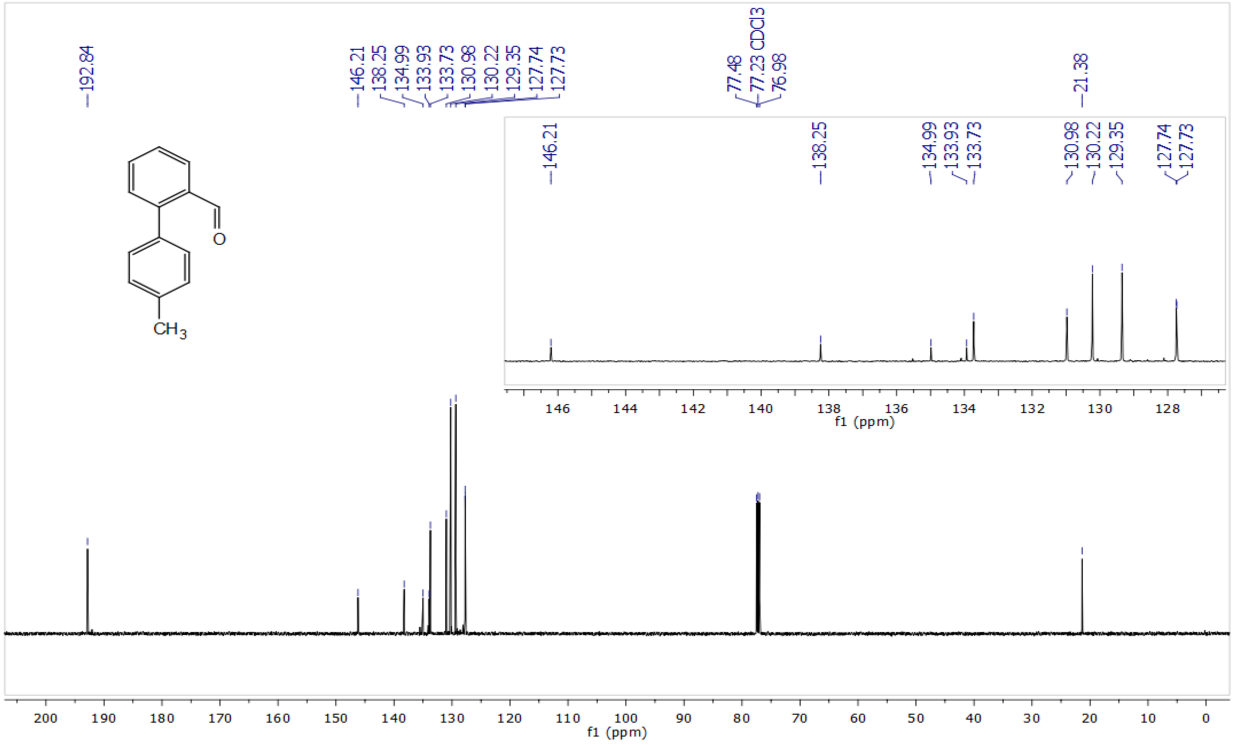

4'-chloro-[1,1'-biphenyl]-2-carbaldehyde (1e)

^{1}H NMR

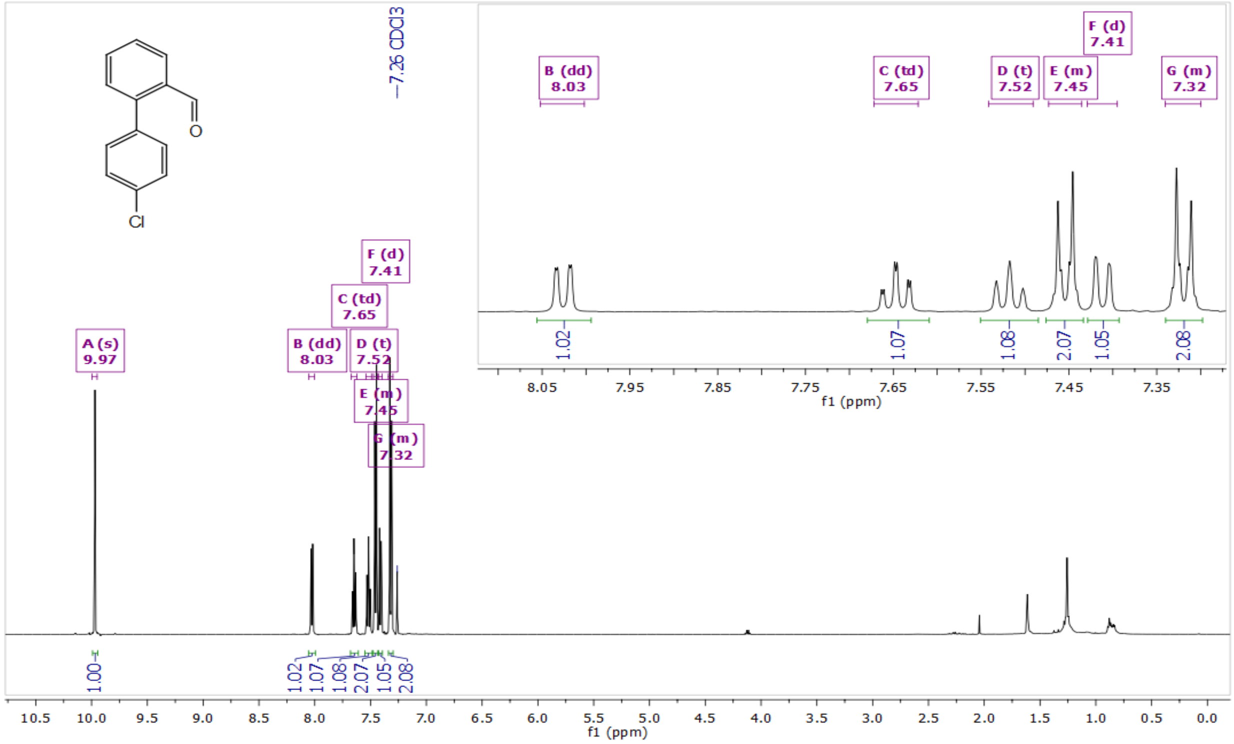

^{13}C NMR

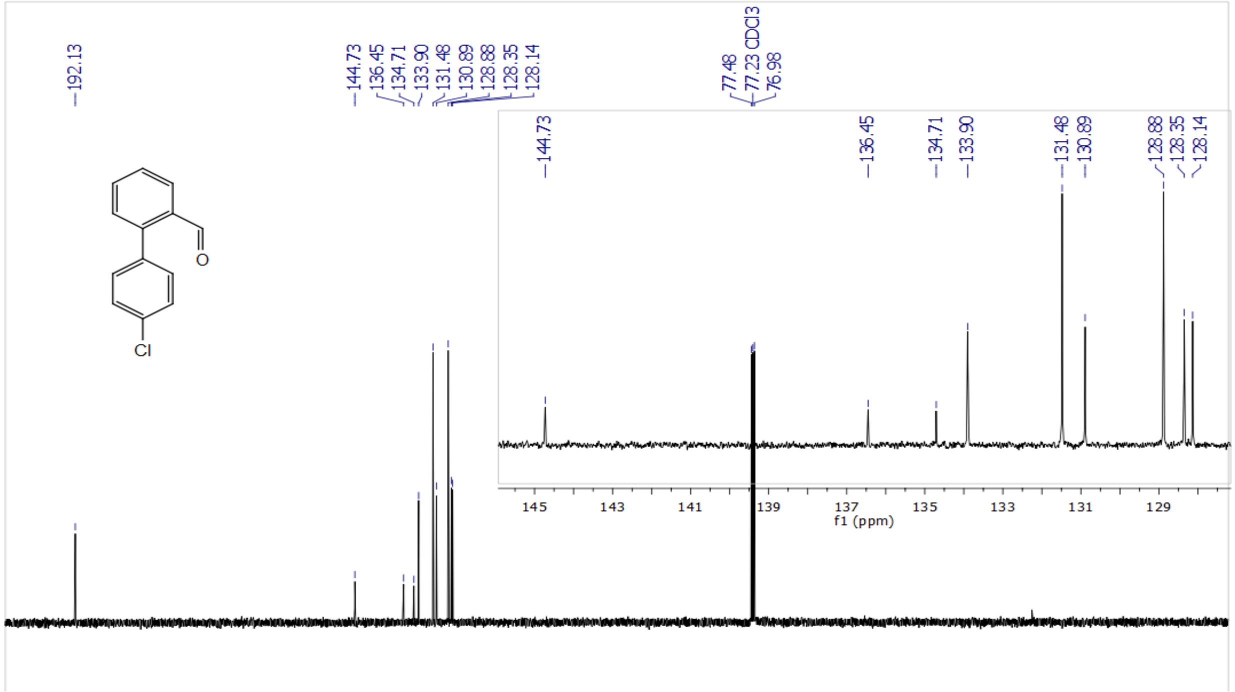

***2'-methyl-[1,1'-biphenyl]-2-carbaldehyde* (1f)**

^{1}H NMR

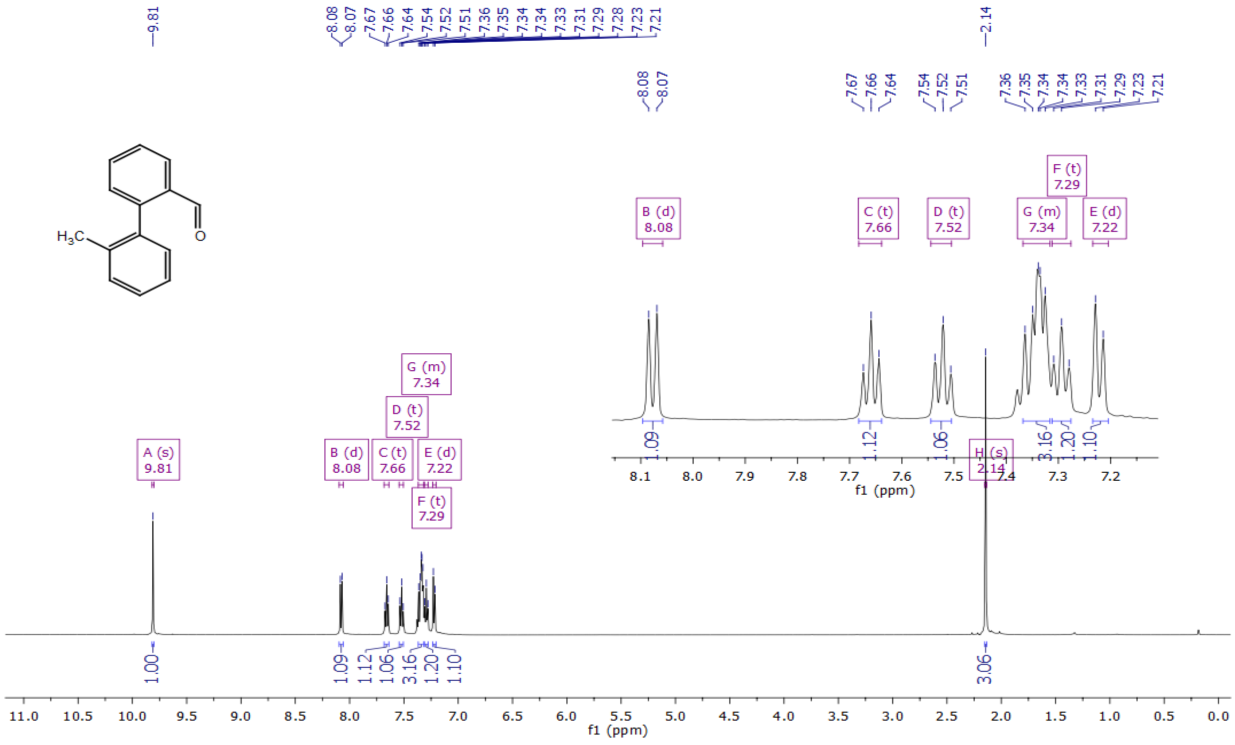

^{13}C NMR

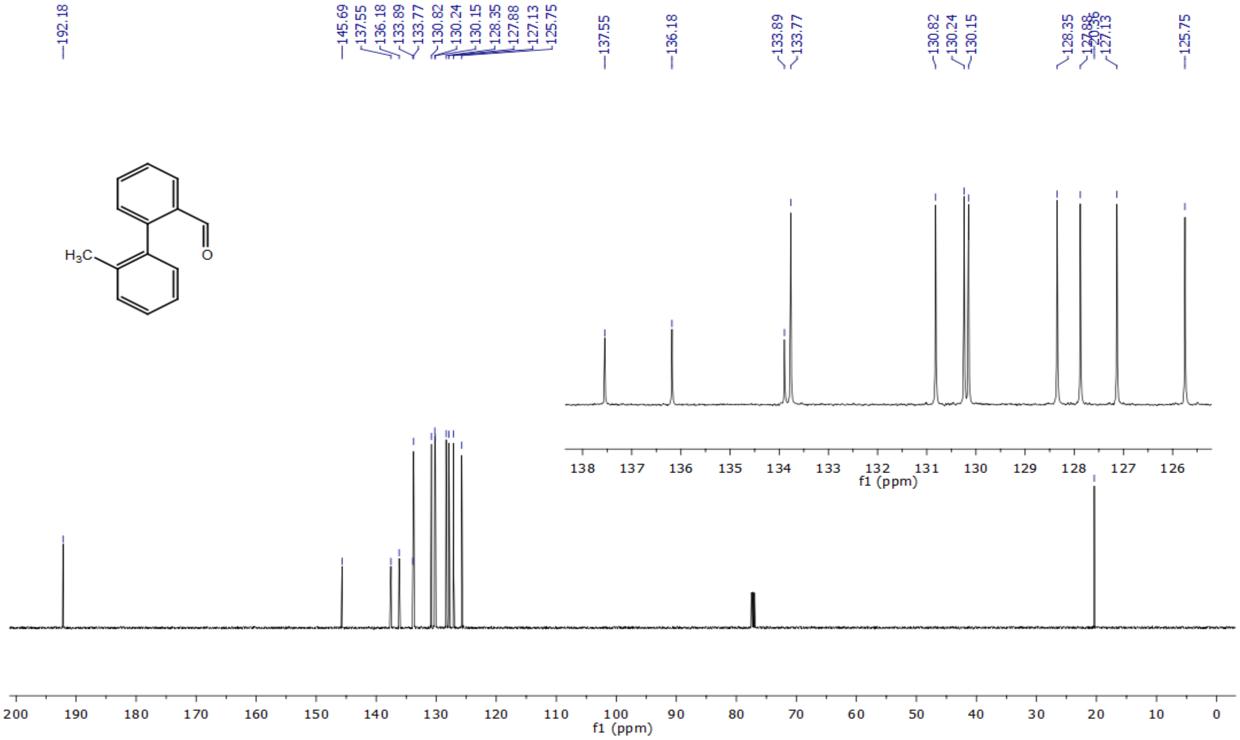

2',6'-dimethyl-[1,1'-biphenyl]-2-carbaldehyde (**1g**)

^{1}H NMR

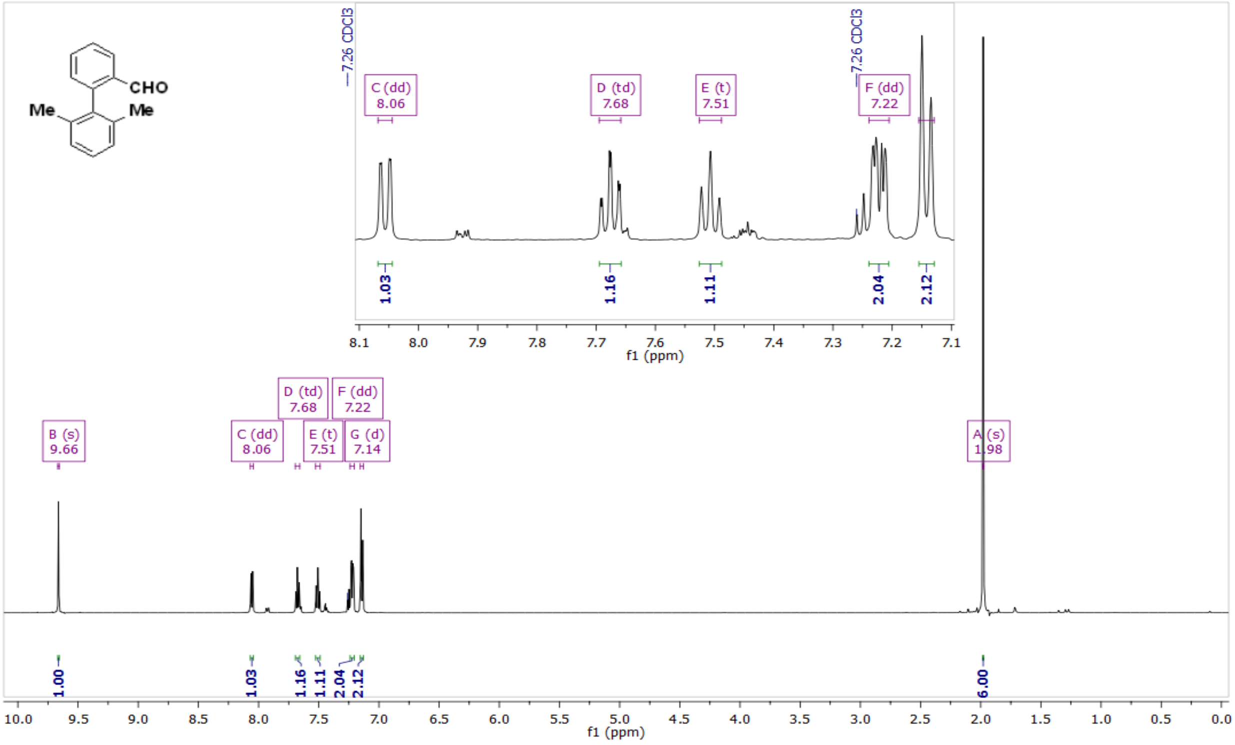

^{13}C NMR

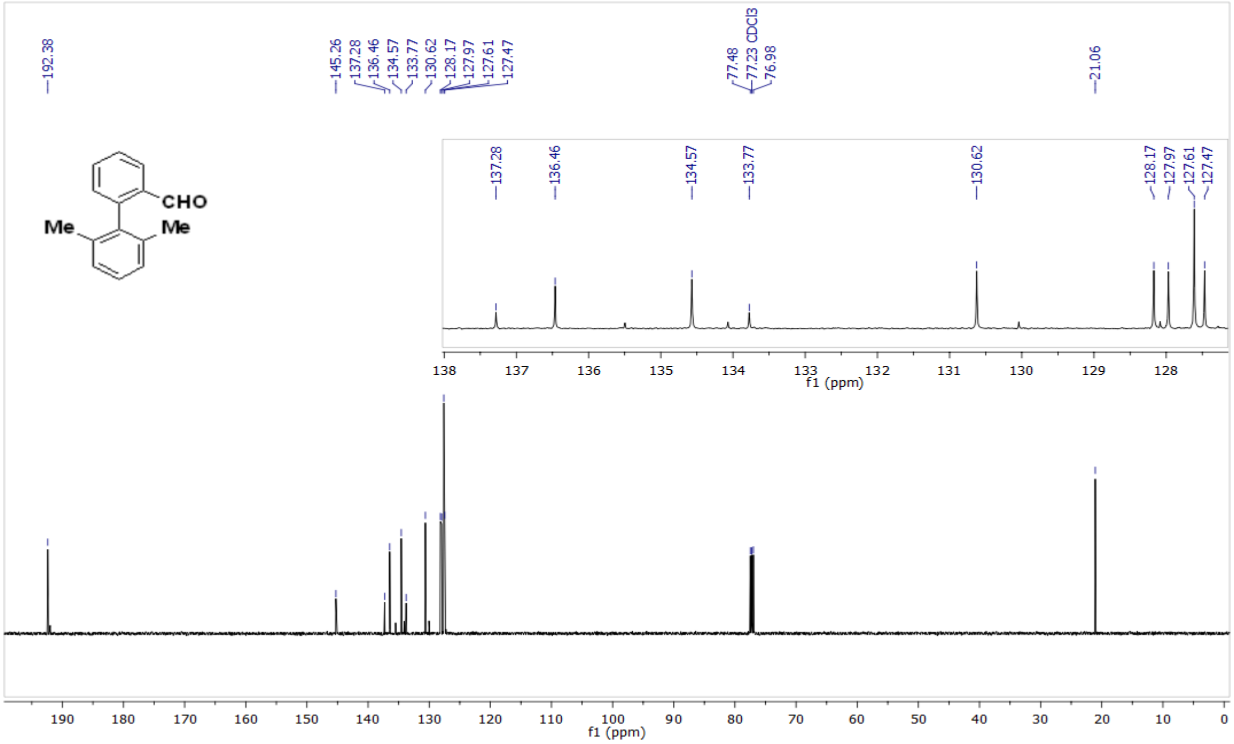

4'-methoxy-2',5'-dimethyl-[1,1'-biphenyl]-2-carbaldehyde **(1h)**

^{1}H NMR

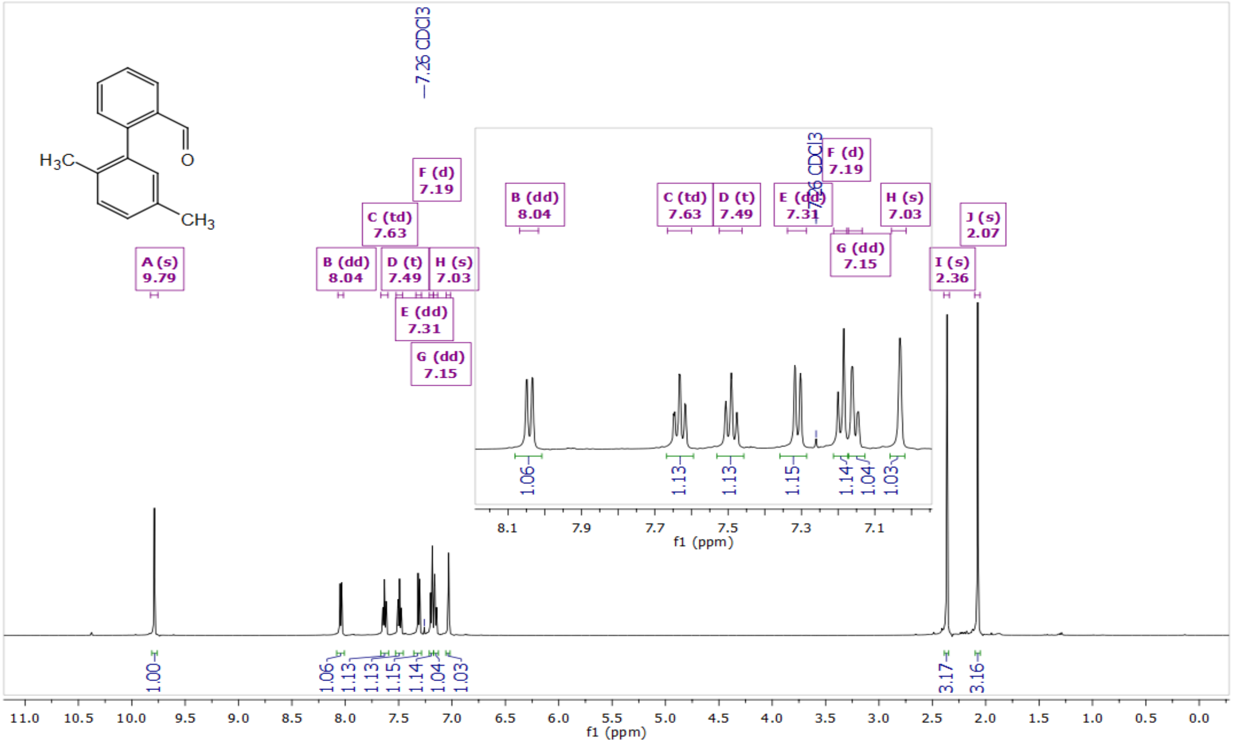

^{13}C NMR

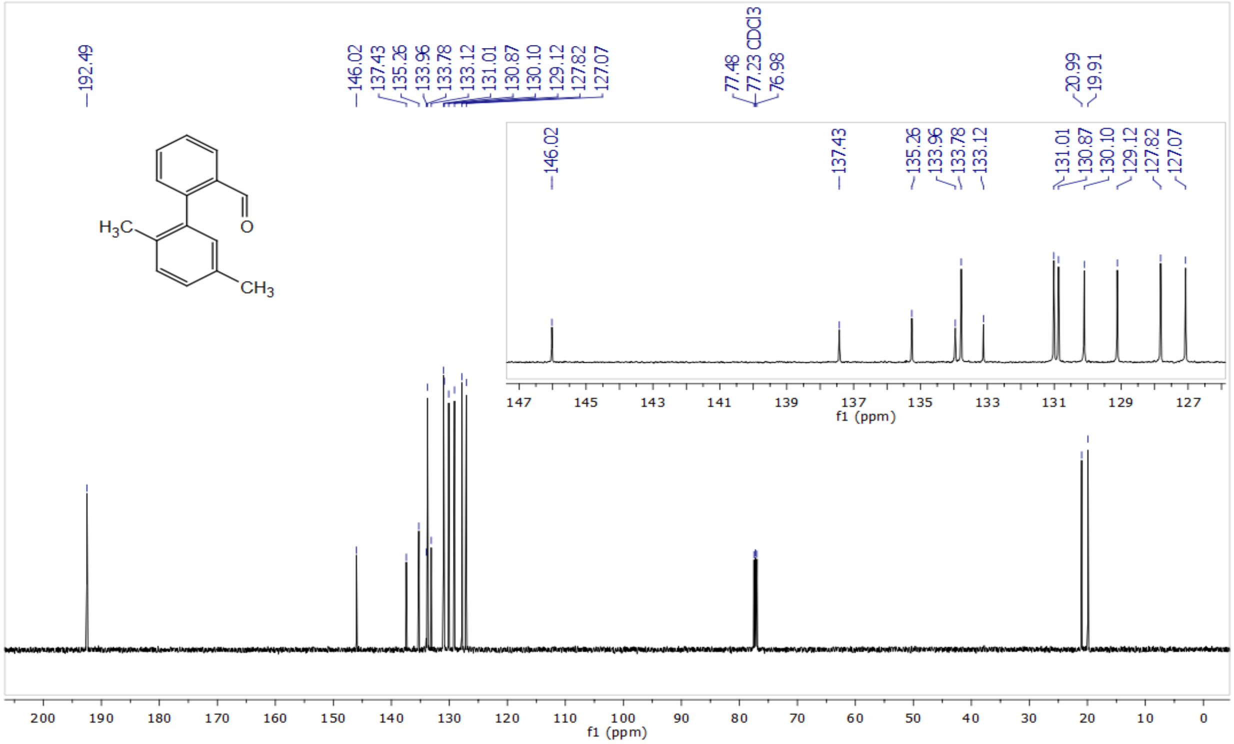

2-(benzo[d][1,3]dioxol-5-yl)benzaldehyde (1i)

^{1}H NMR

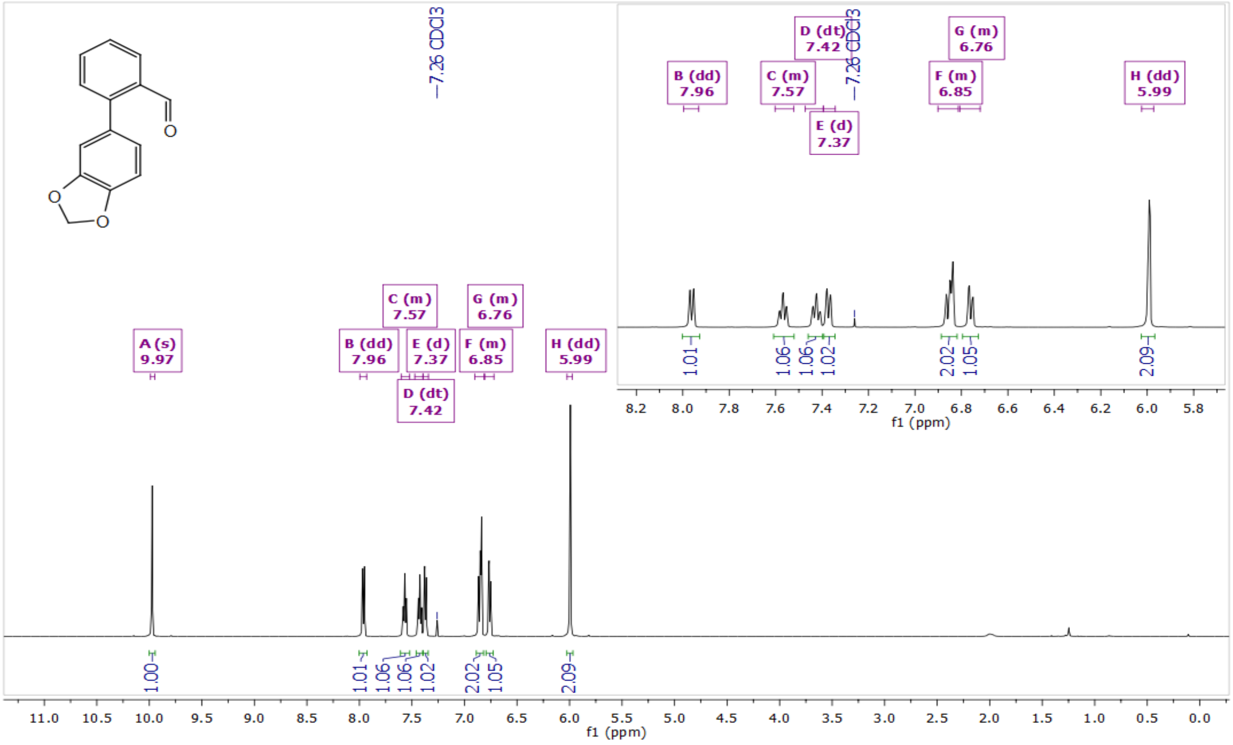

^{13}C NMR

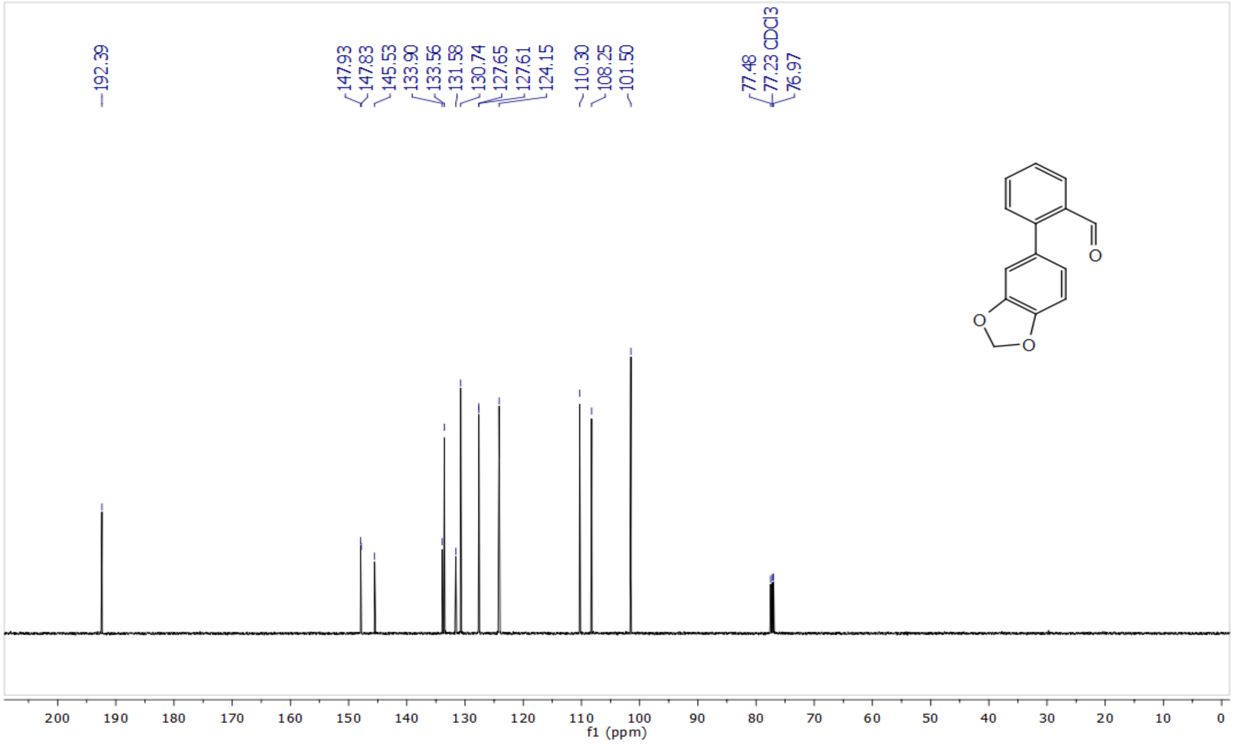

2-(2,3-dihydrobenzo[b][1,4]dioxin-6-yl)benzaldehyde **(1j)**

^{1}H NMR

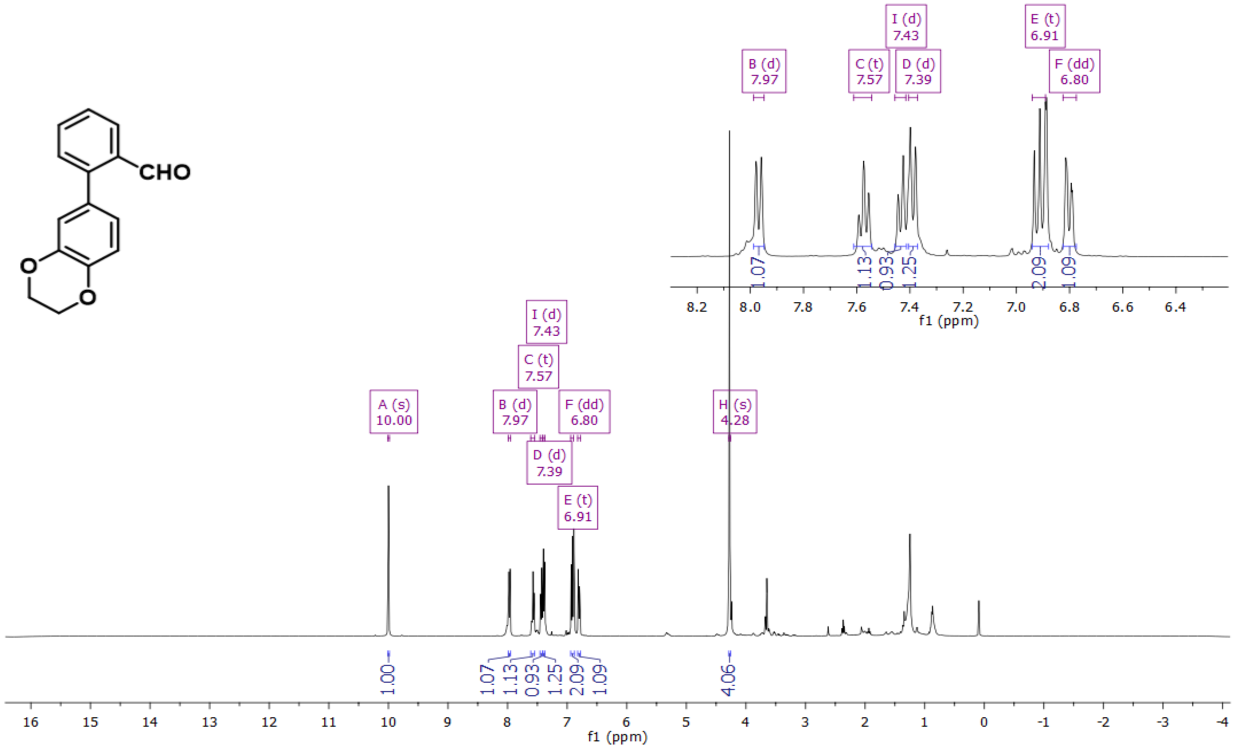

^{13}C NMR

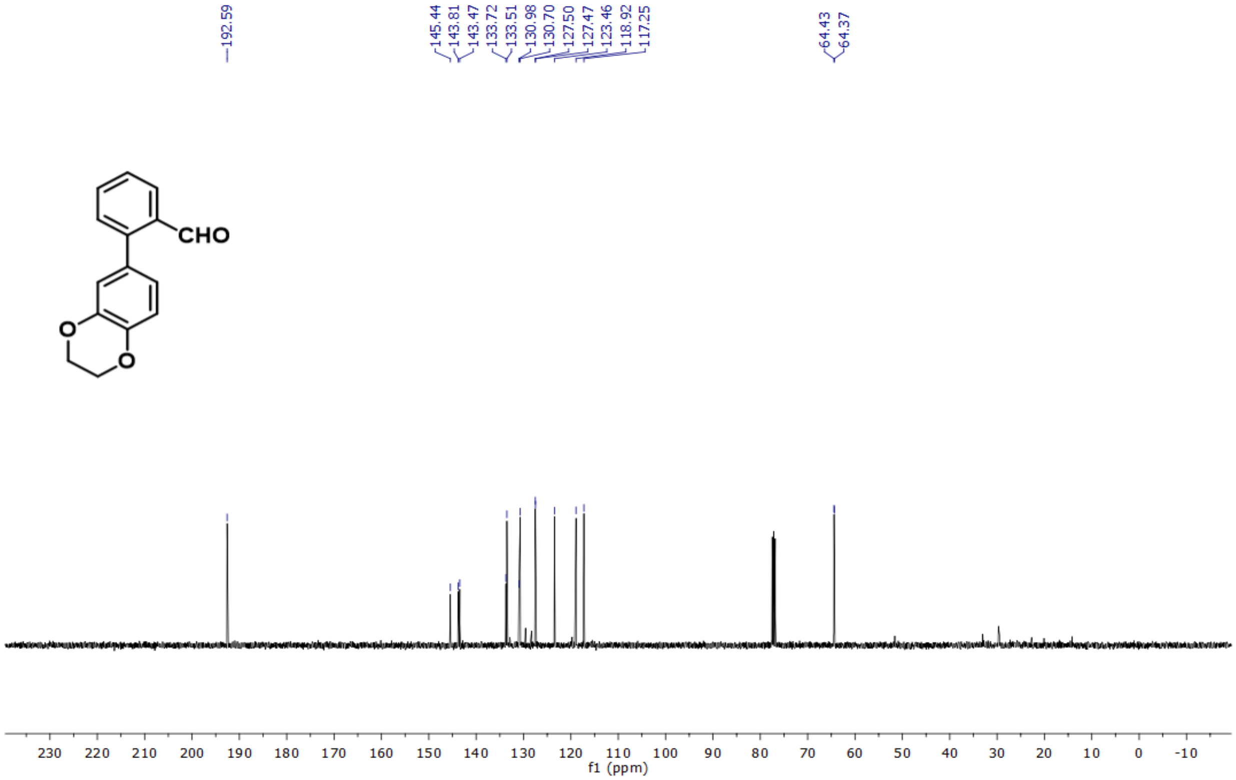

***2',5-dimethyl-[1,1'-biphenyl]-2-carbaldehyde* (1k)**

^{1}H NMR

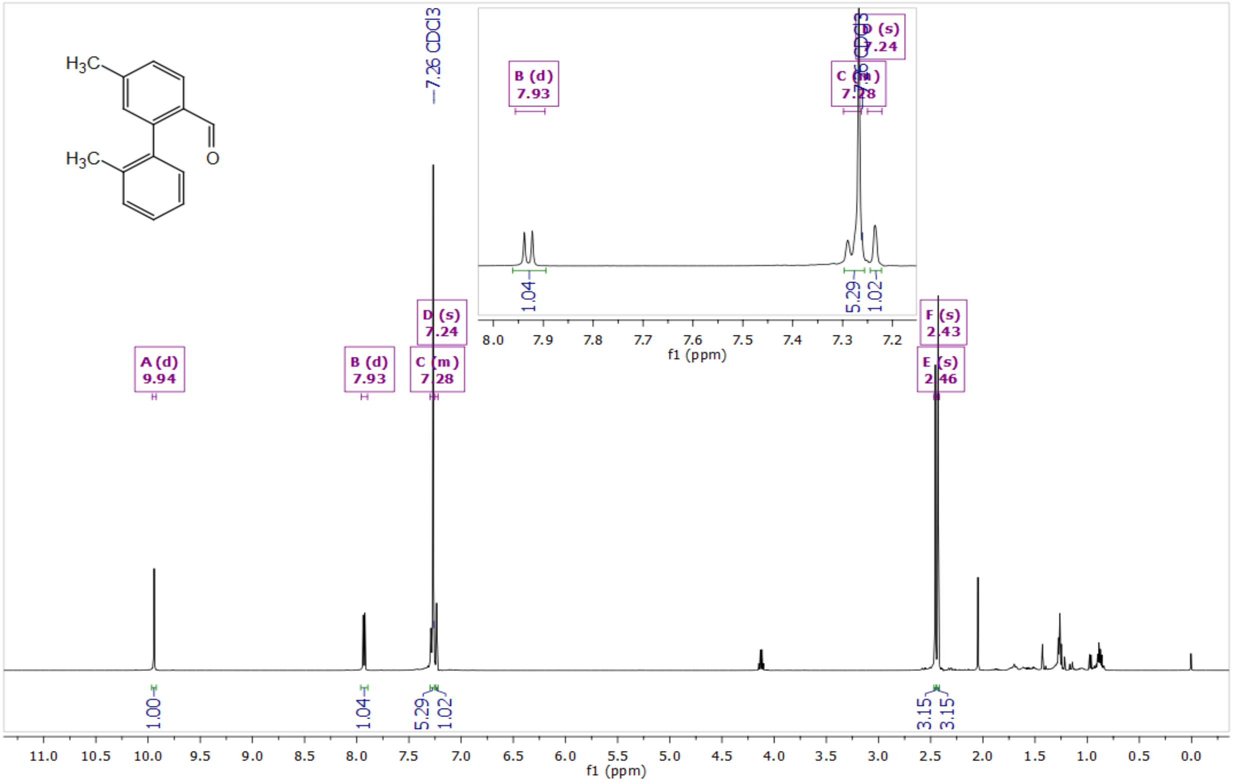

^{13}C NMR

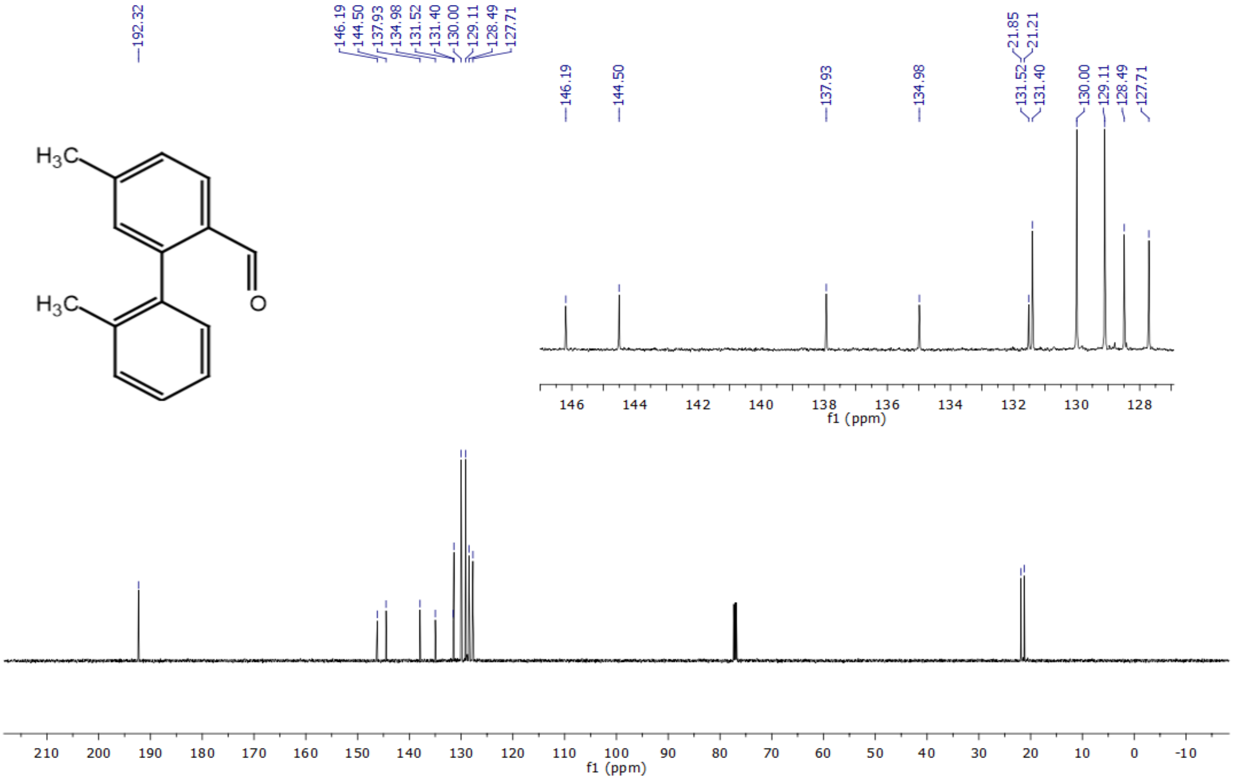

***4'-methoxy-5-methyl-[1,1'-biphenyl]-2-carbaldehyde* (1l)**

^{1}H NMR

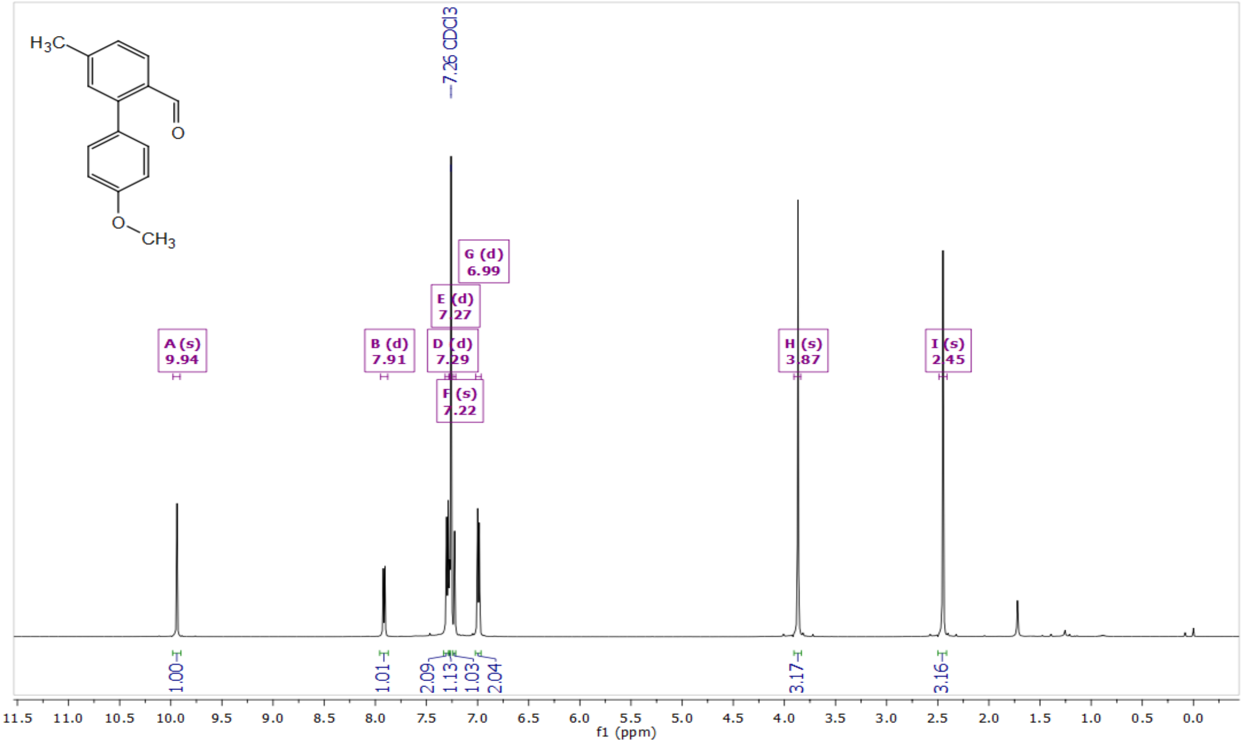

^{13}C NMR

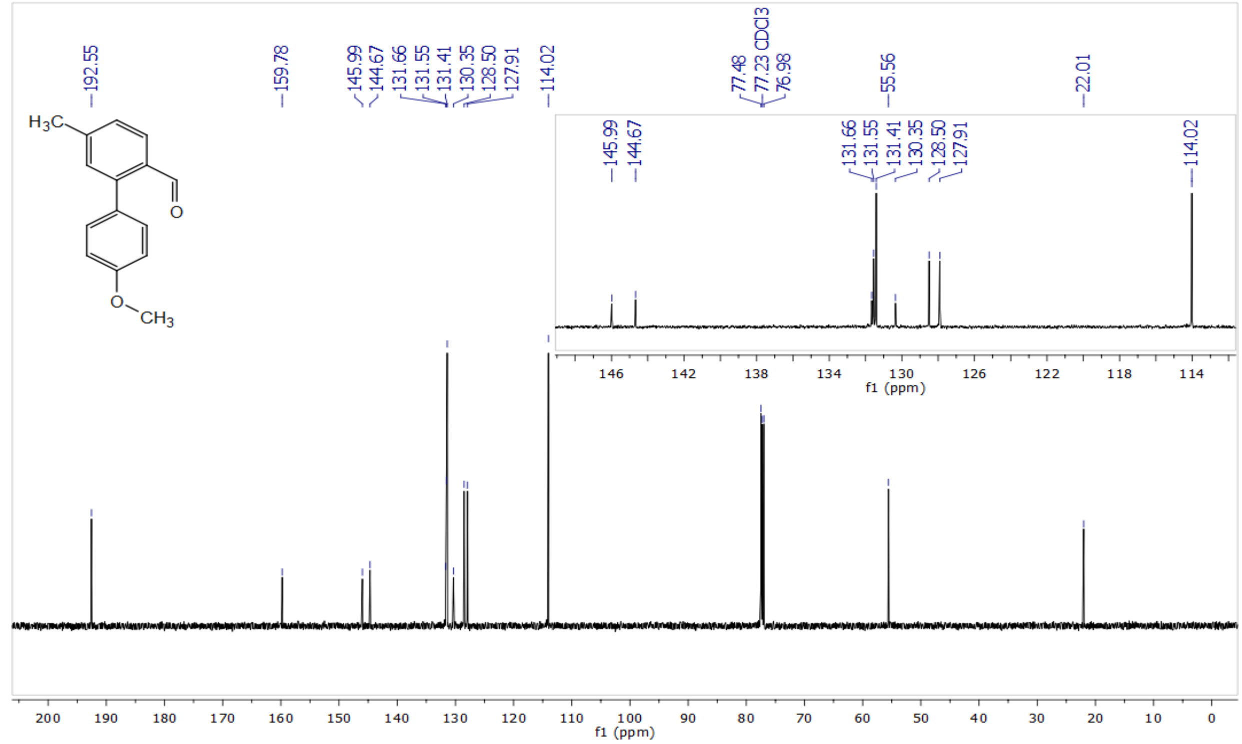

3',4-dimethoxy-[1,1'-biphenyl]-2-carbaldehyde **(1m)**

^{1}H NMR

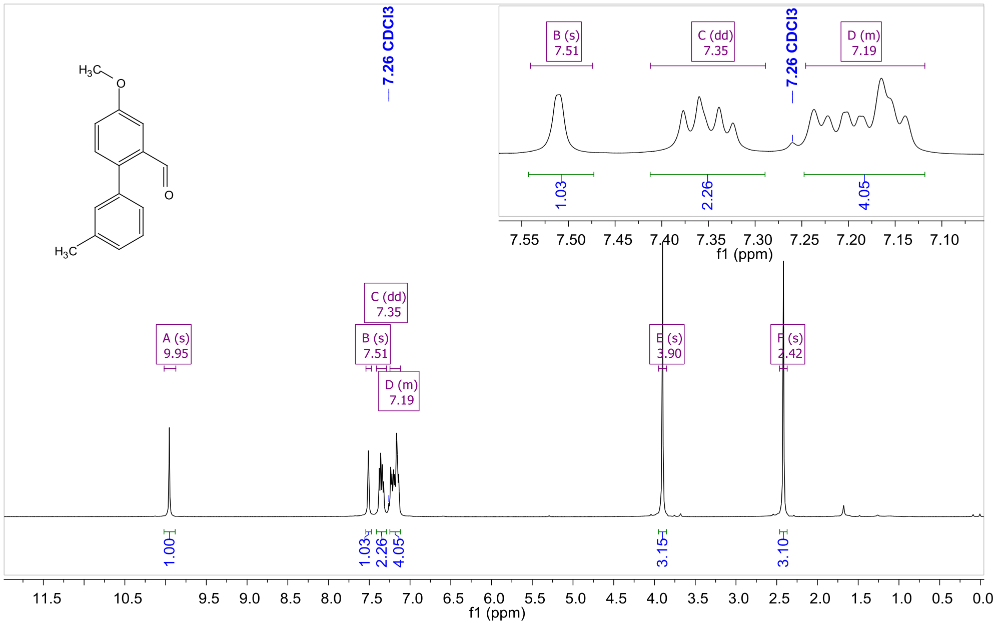

^{13}C NMR

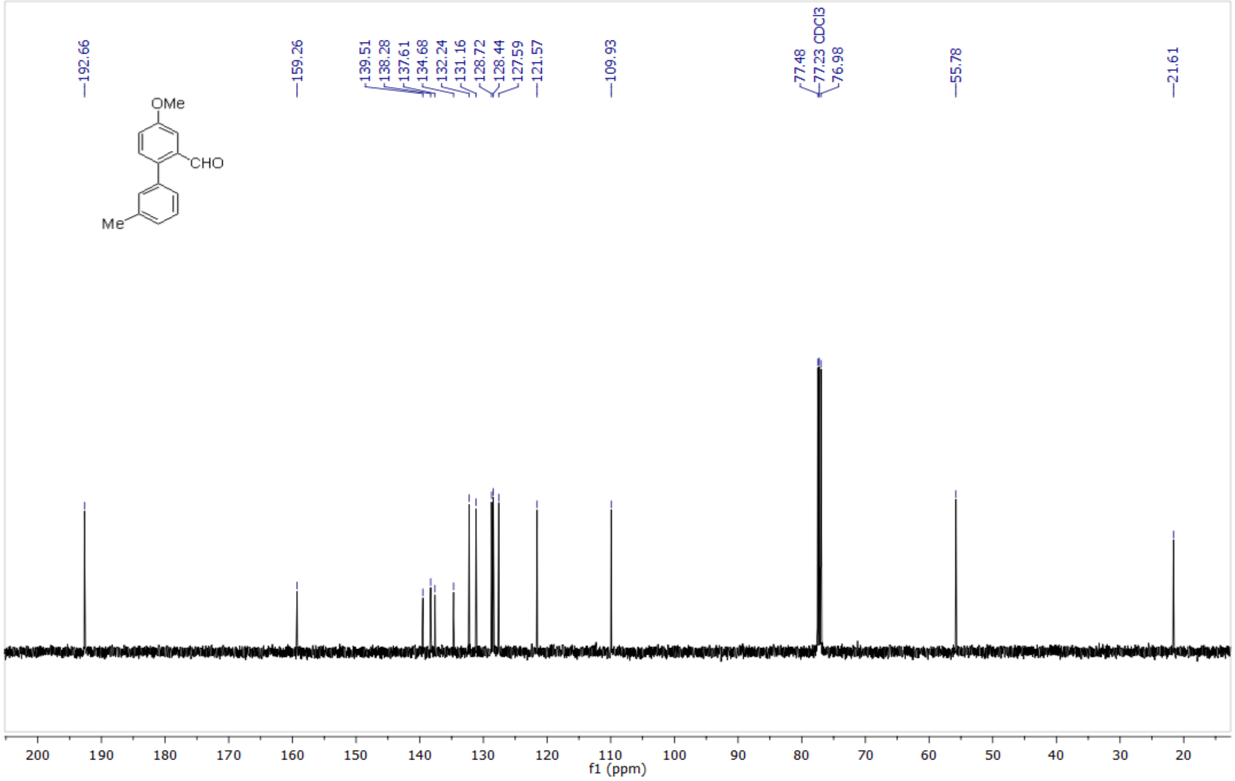

4,4',5-trimethoxy-[1,1'-biphenyl]-2-carbaldehyde **(1n)**

^{1}H NMR

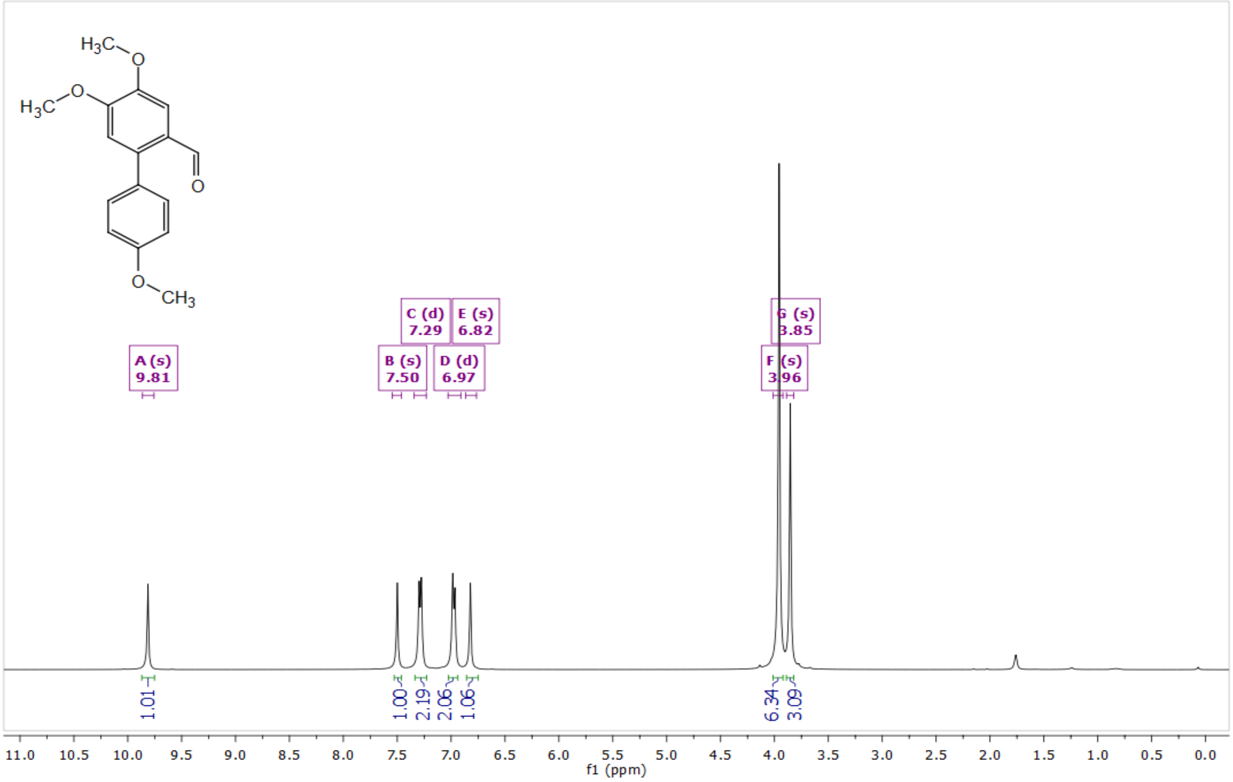

^{13}C NMR

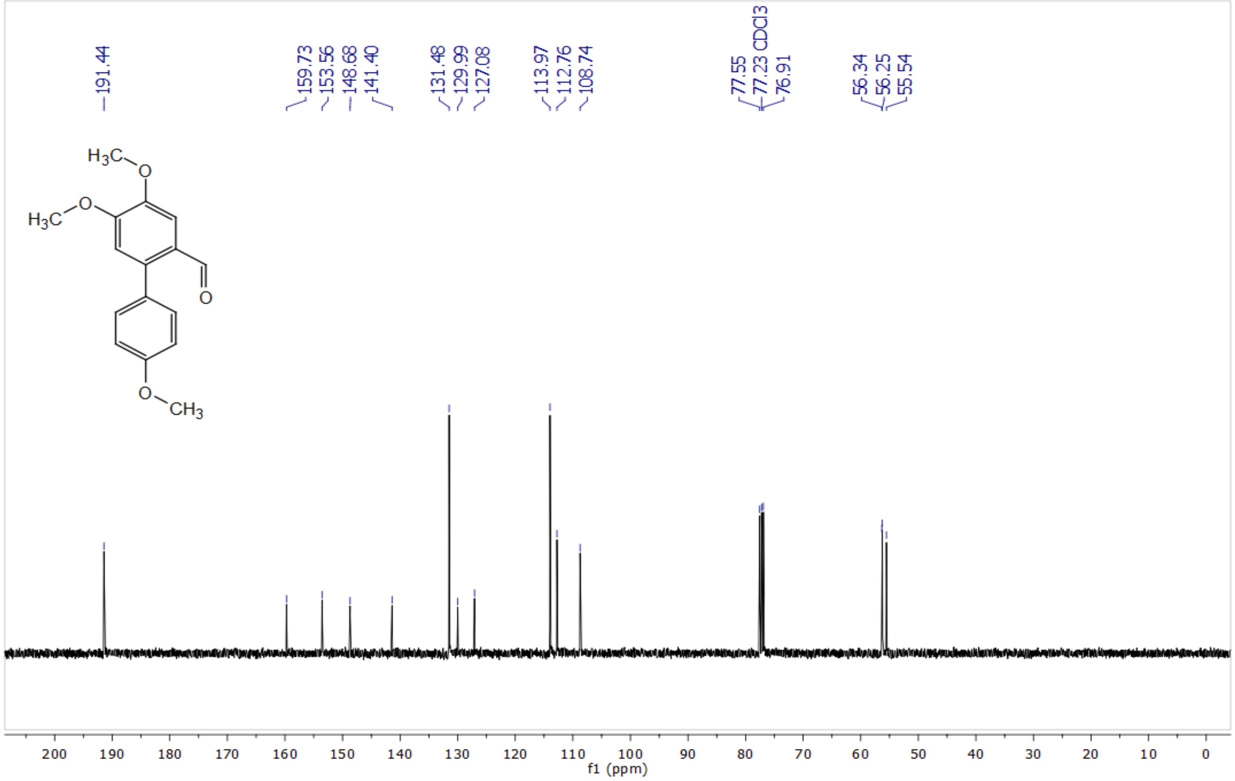

2-(benzo[d][1,3]dioxol-5-yl)-4-methoxybenzaldehyde (**1o**)

^{1}H NMR

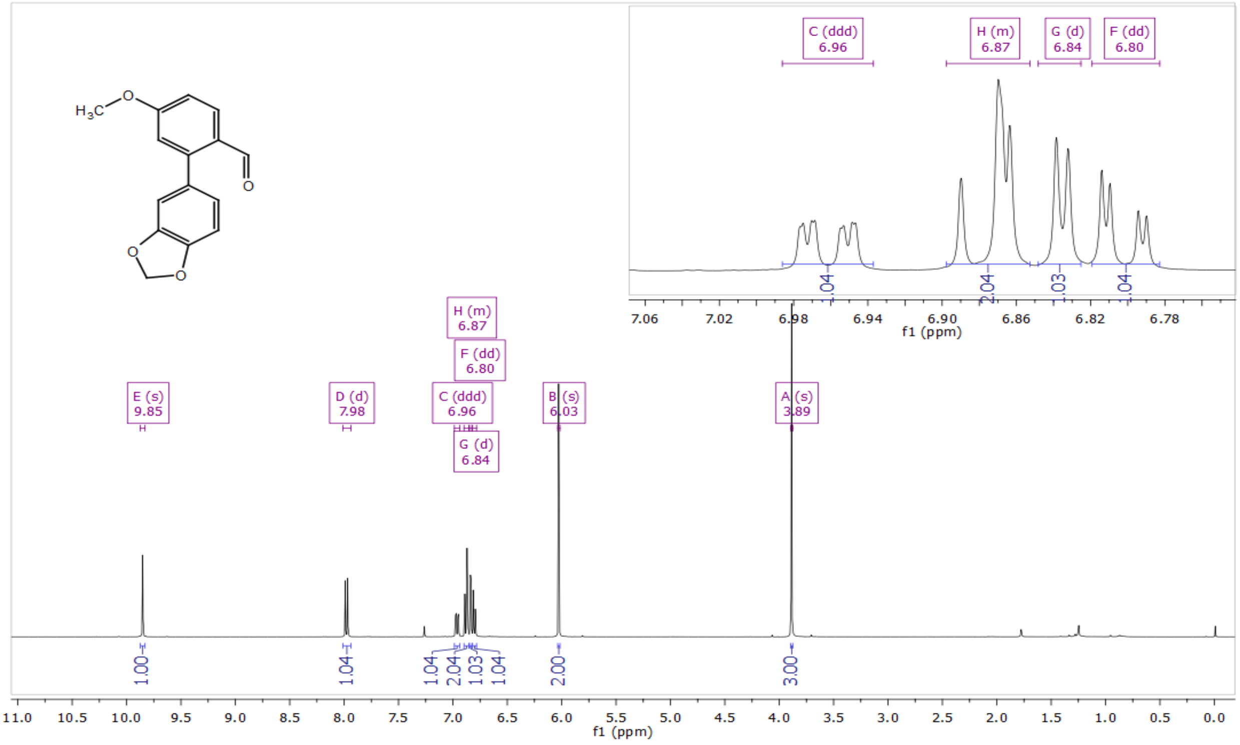

^{13}C NMR

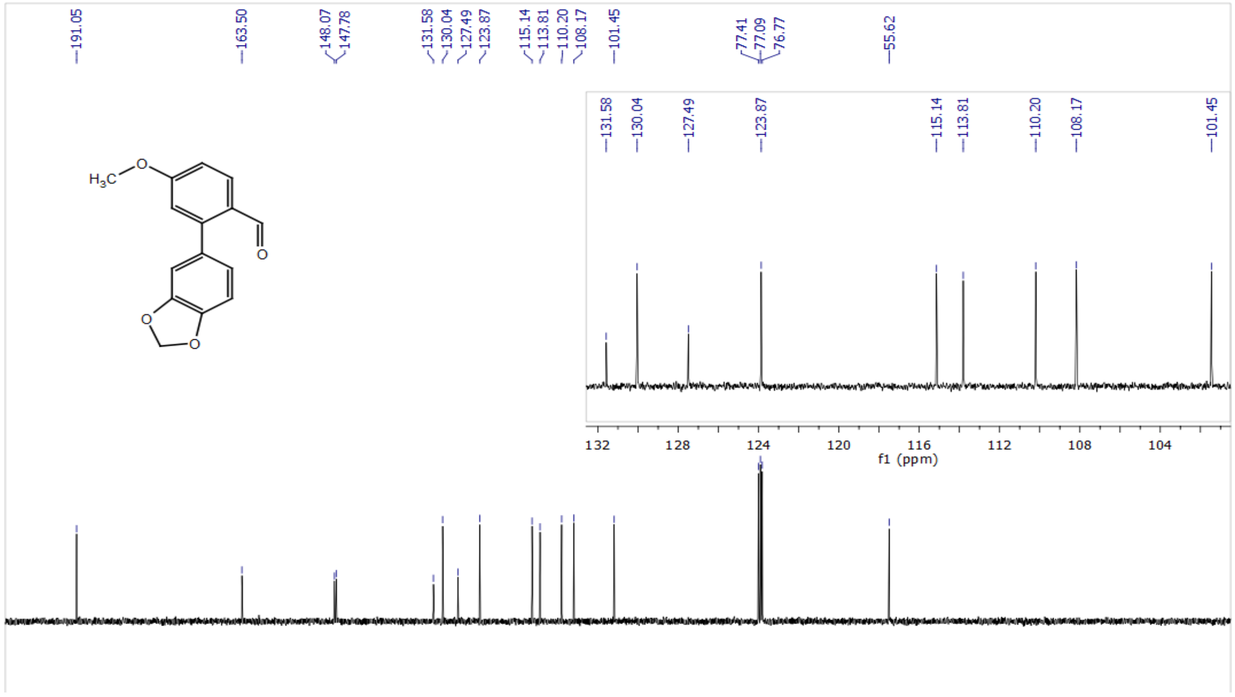

***[1,1'-biphenyl]-2',3',4',5',6'-d5-2-carbaldehyde* (1b-d5)**

^{1}H NMR

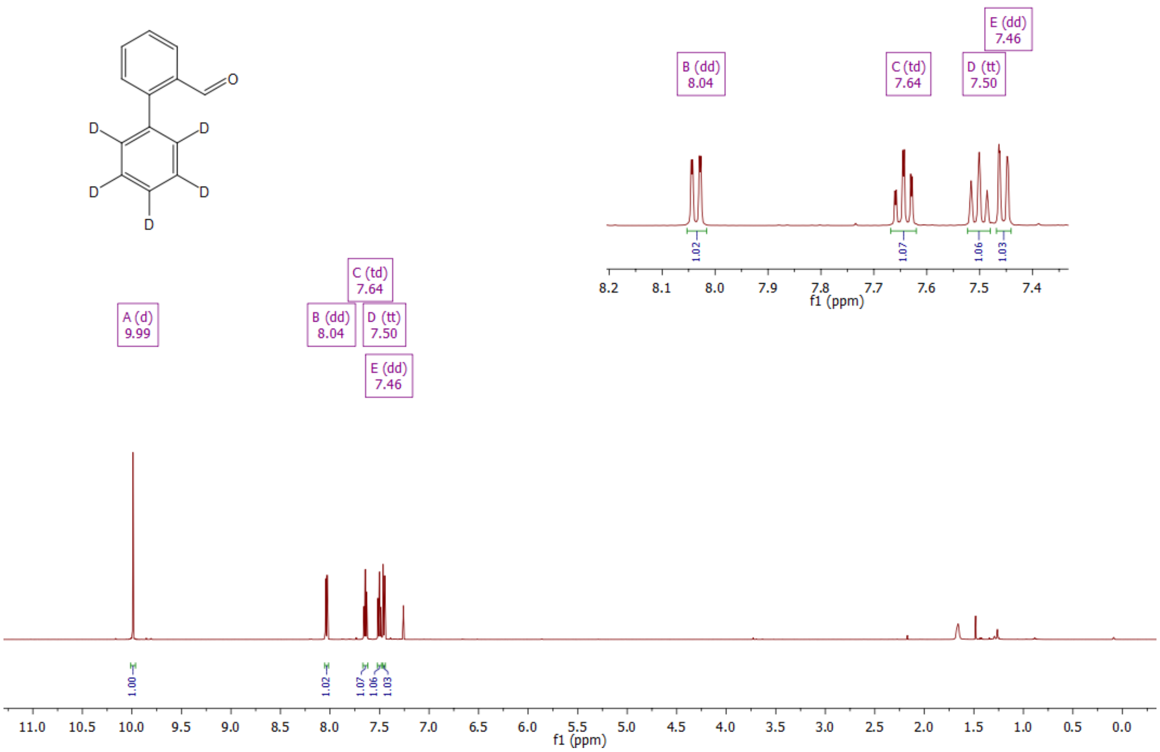

^{13}C NMR

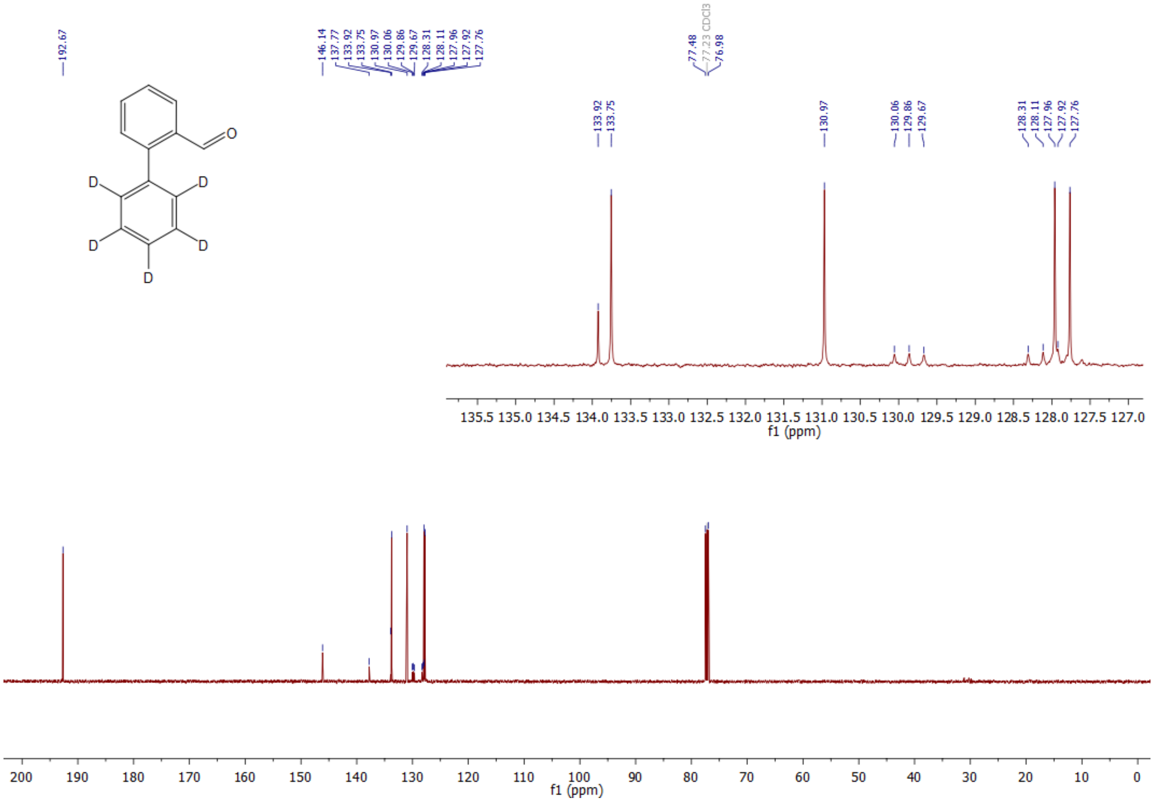

2-phenylaniline derivatives: *[1,1'-biphenyl]-2-amine* (2a)

^{1}H NMR

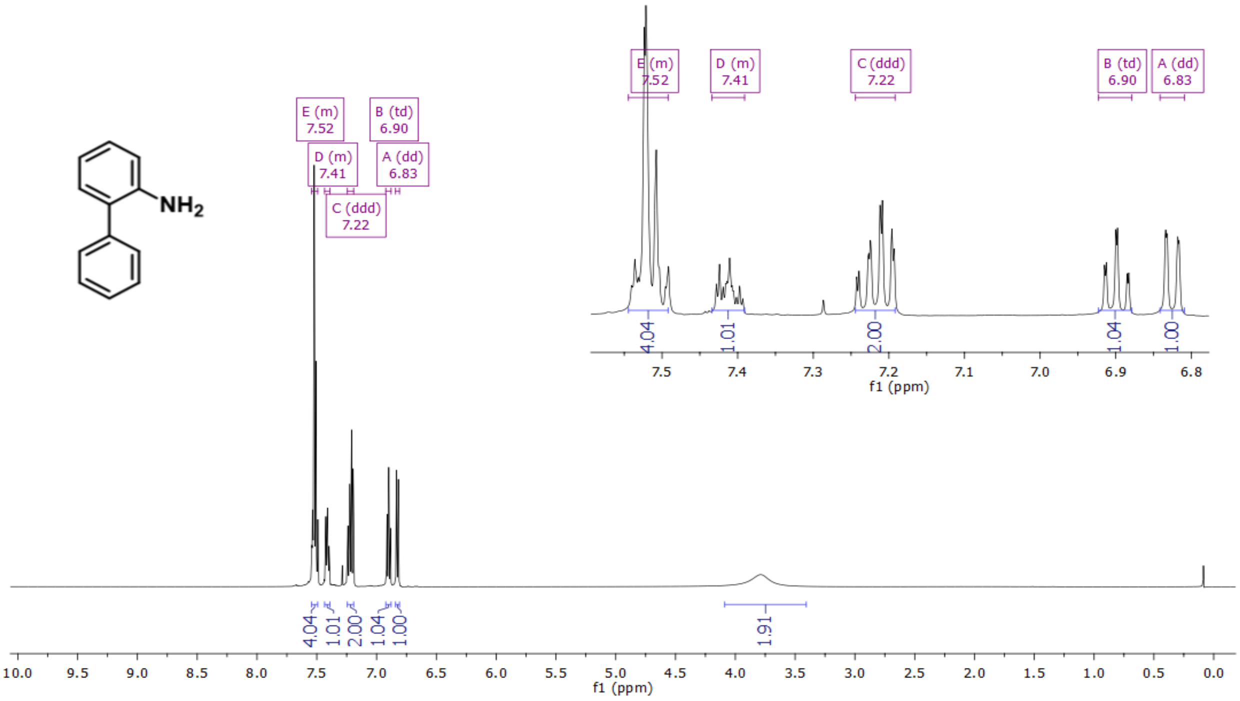

^{13}C NMR

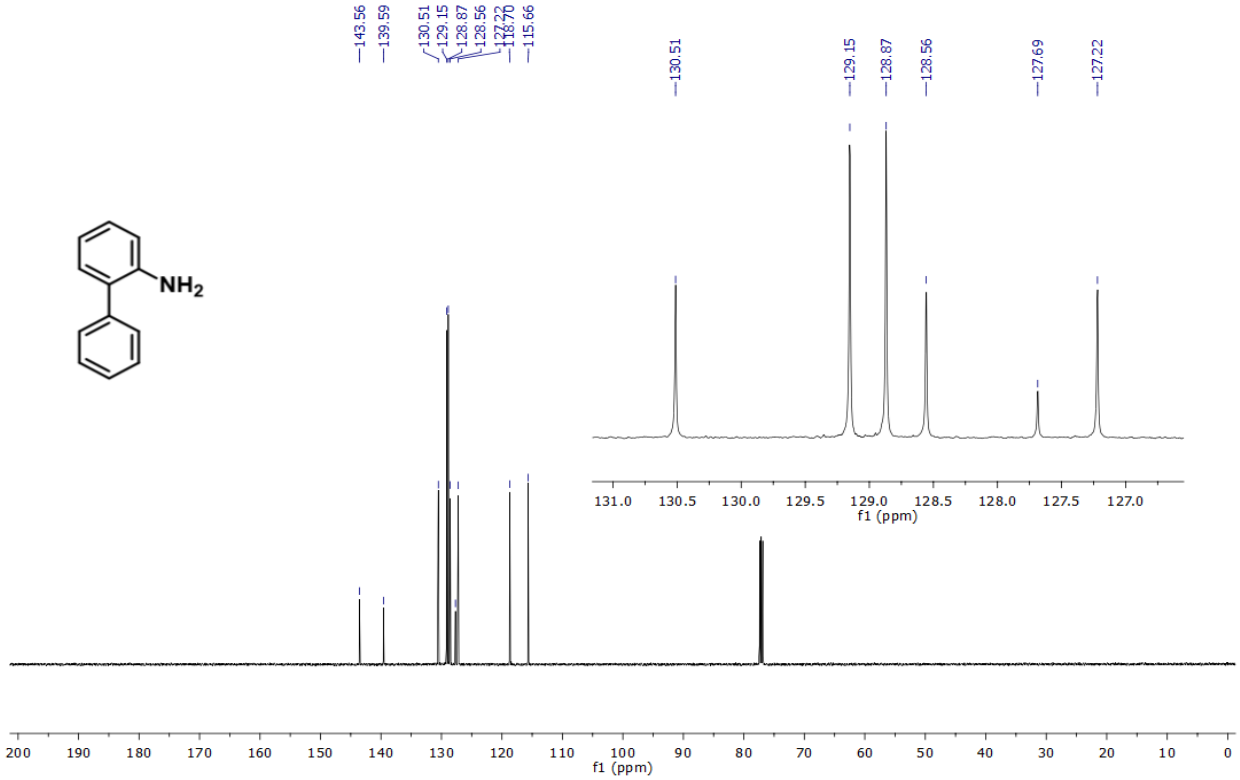

3'-methyl-[1,1'-biphenyl]-2-amine **(2b)**

^{1}H NMR

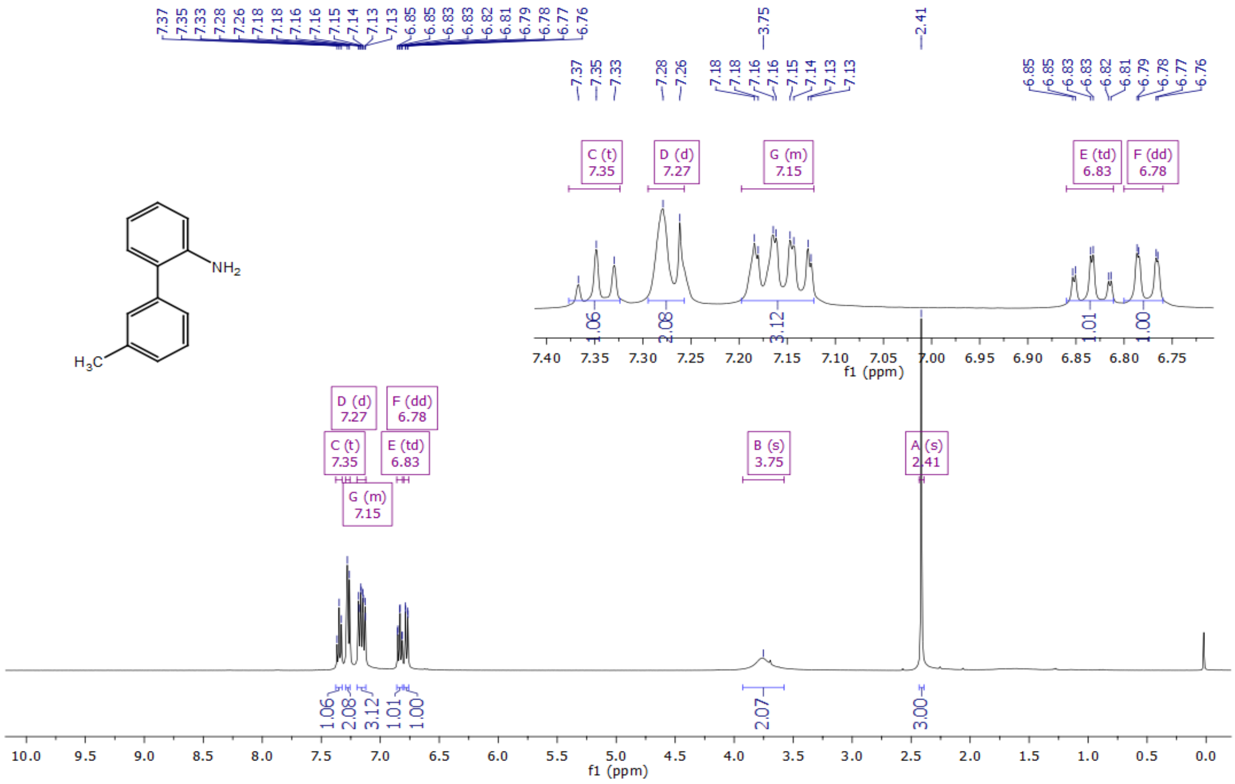

^{13}C NMR

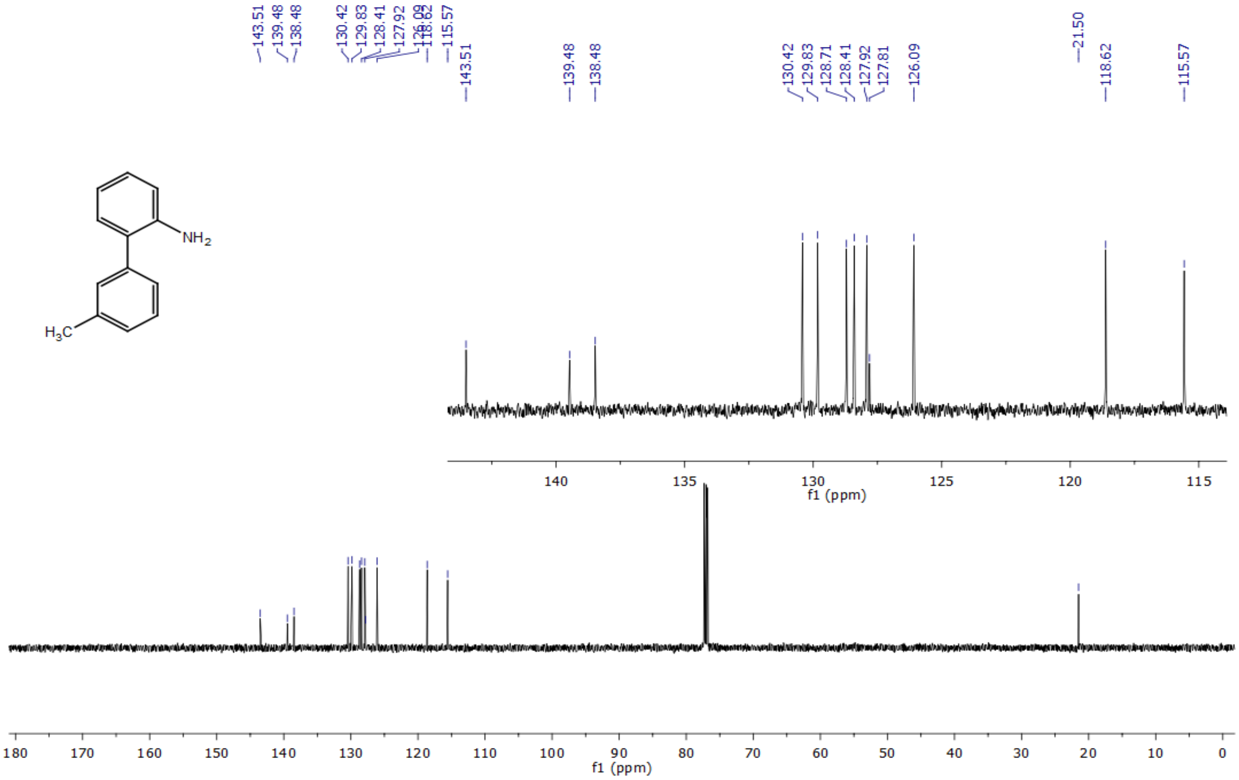

3'-chloro-[1,1'-biphenyl]-2-amine **(2c)**

^{1}H NMR

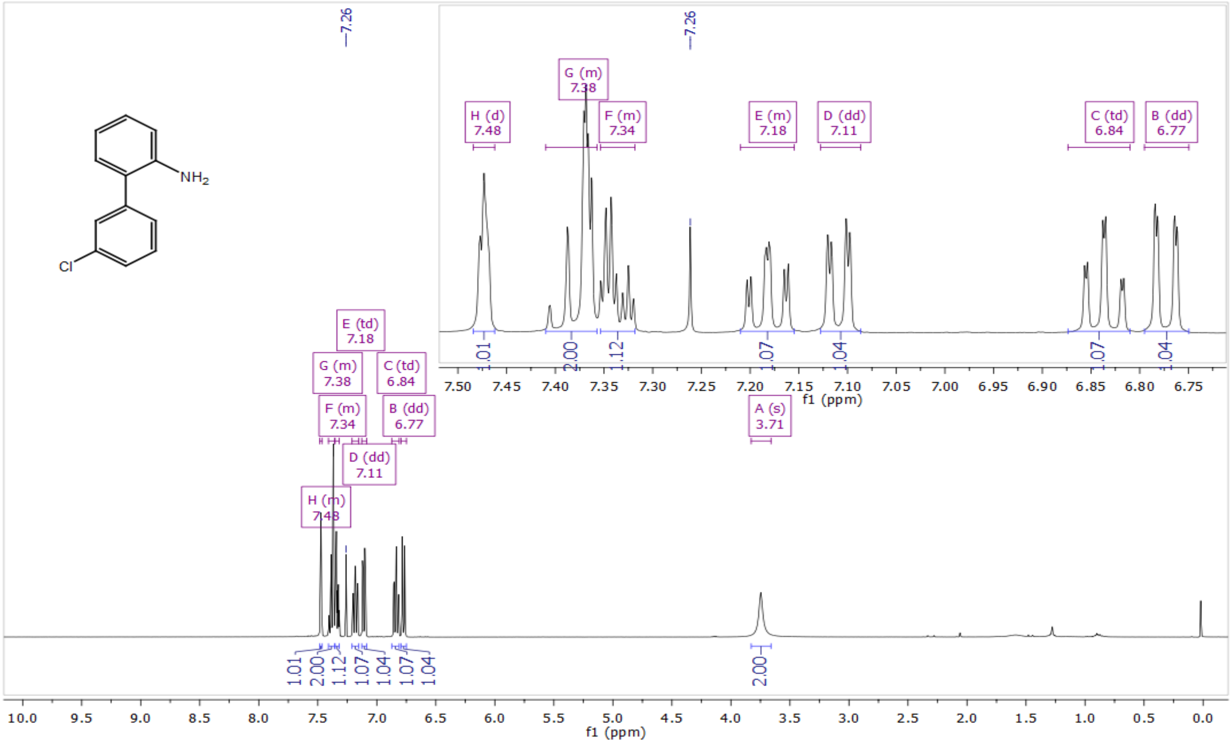

^{13}C NMR

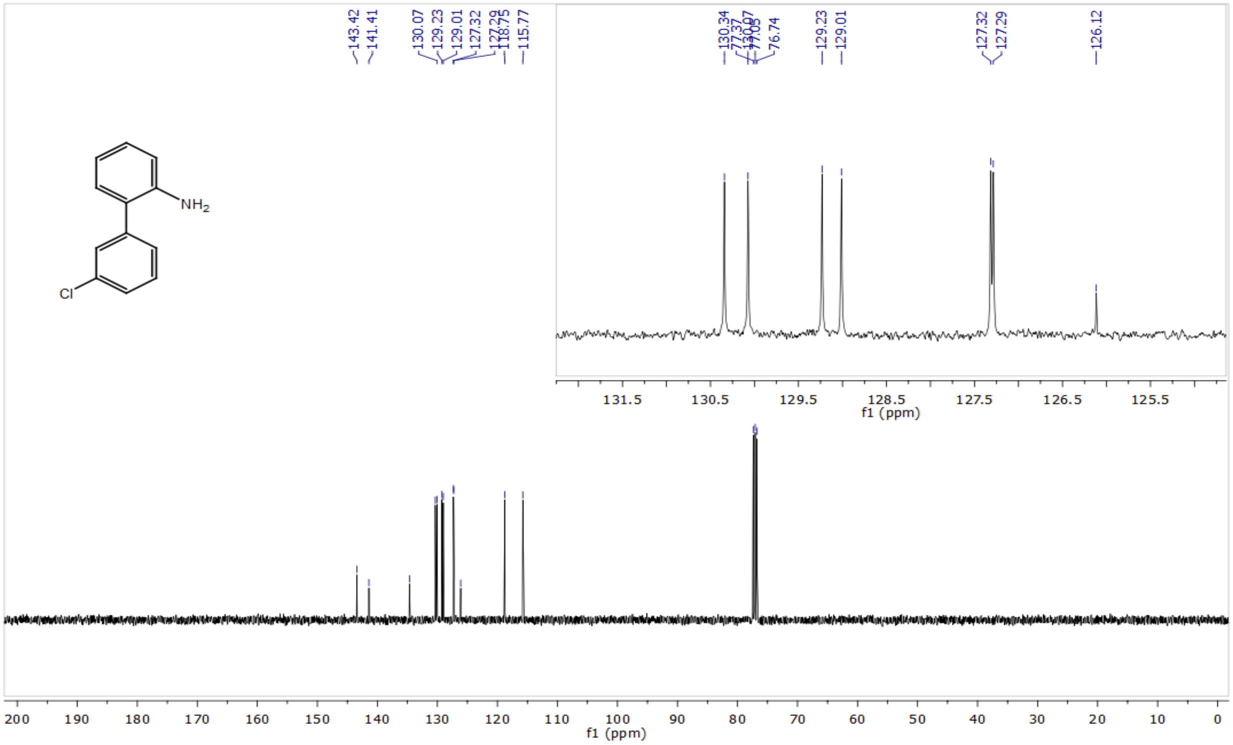

4'-fluoro-[1,1'-biphenyl]-2-amine (2d)

^{1}H NMR

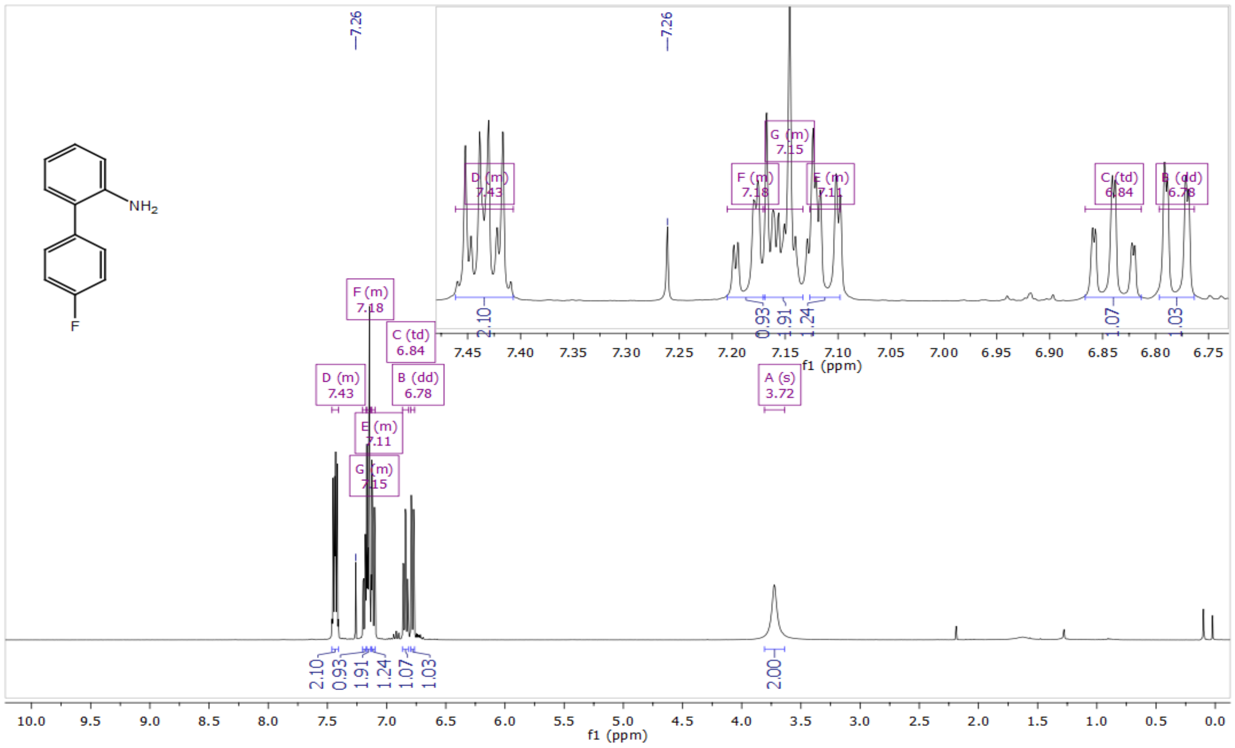

^{13}C NMR

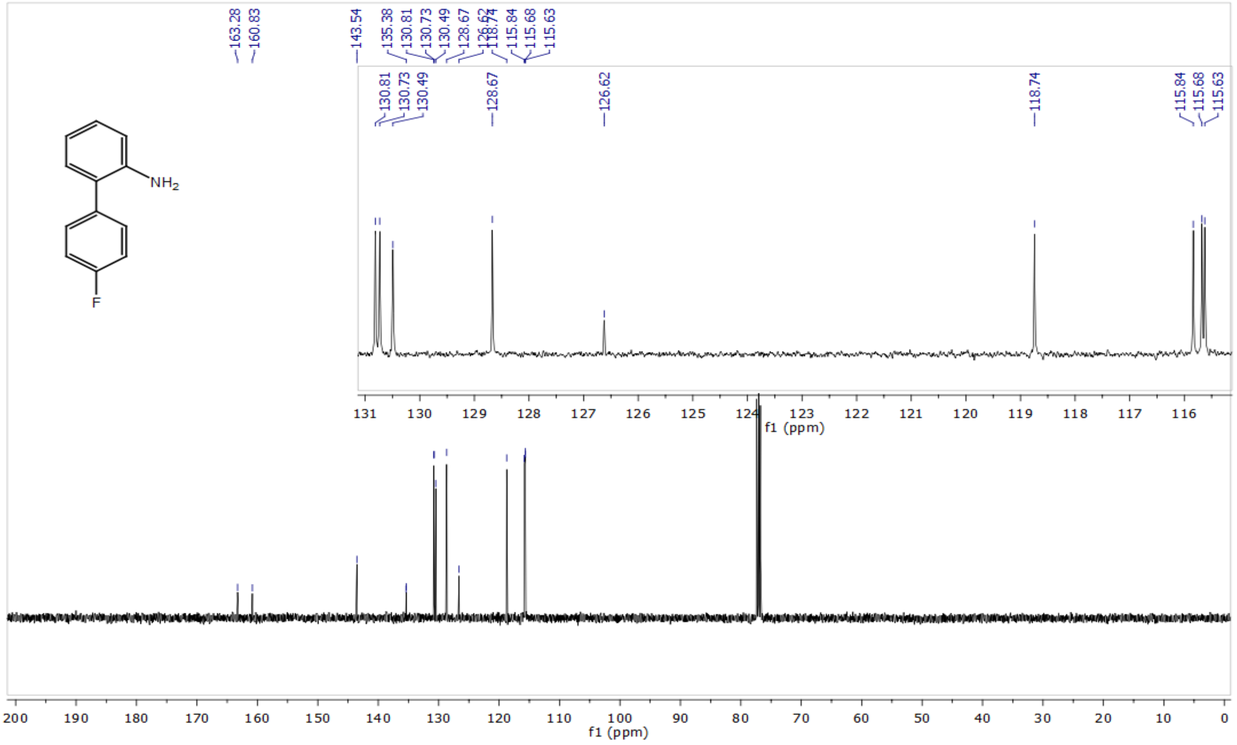

2'-fluoro-[1,1'-biphenyl]-2-amine (2e)

^{1}H NMR

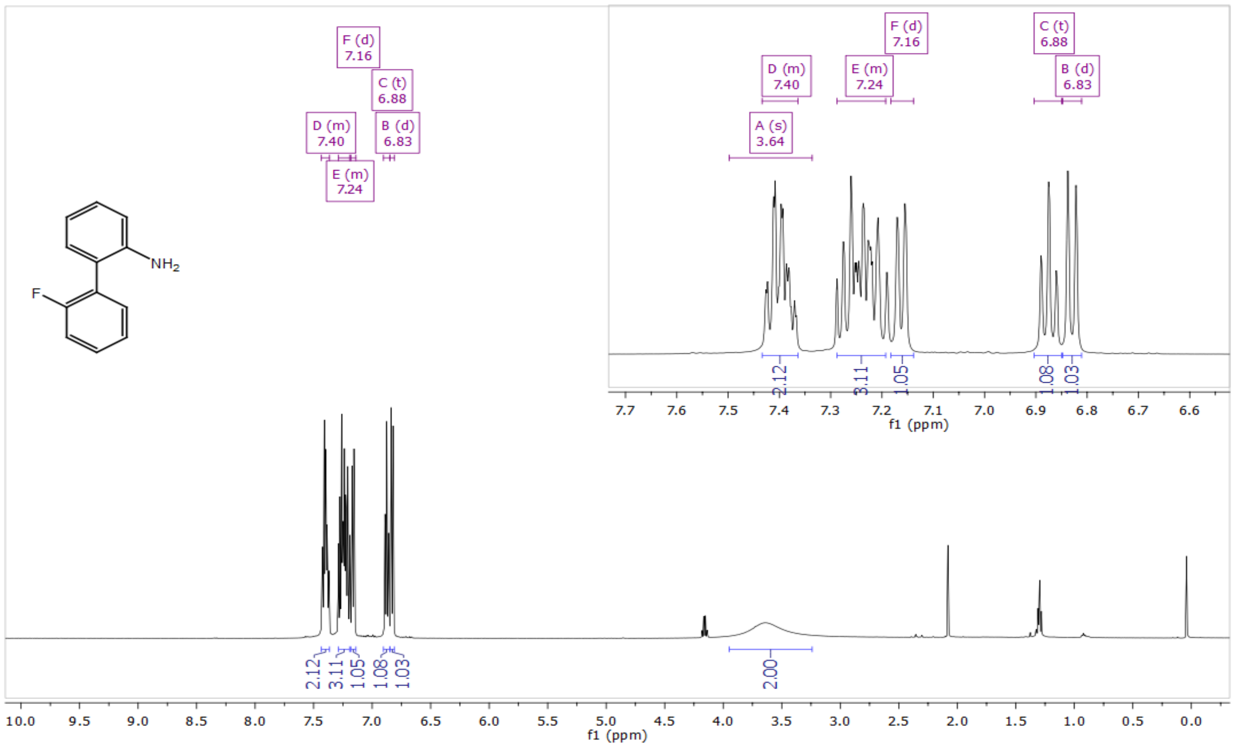

^{13}C NMR

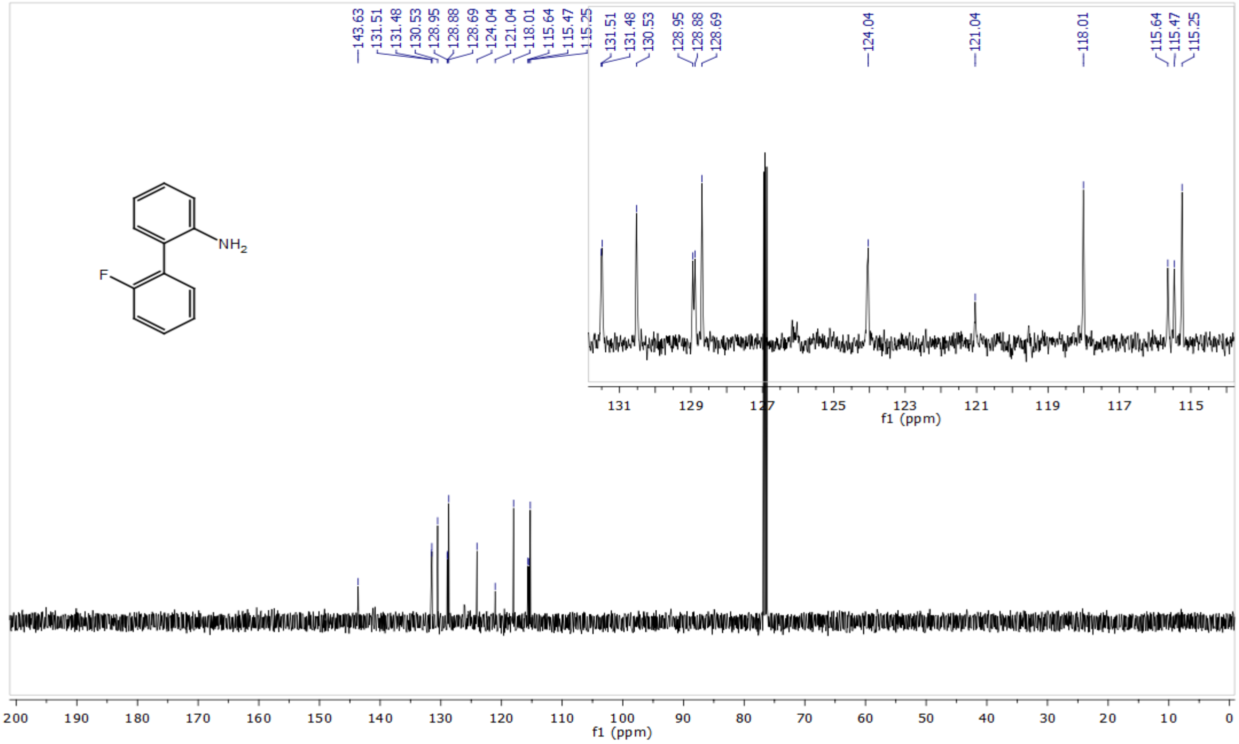

4'-fluoro-3'-methyl-[1,1'-biphenyl]-2-amine (2f)

^{1}H NMR

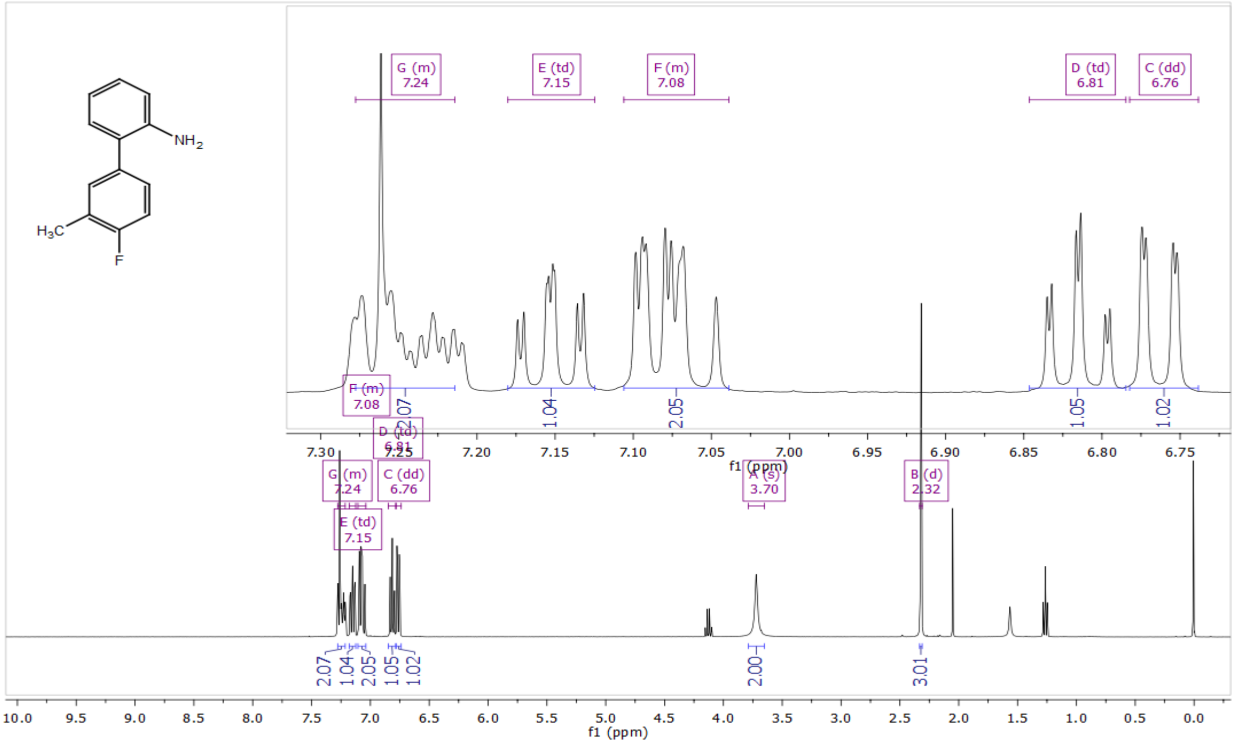

^{13}C NMR

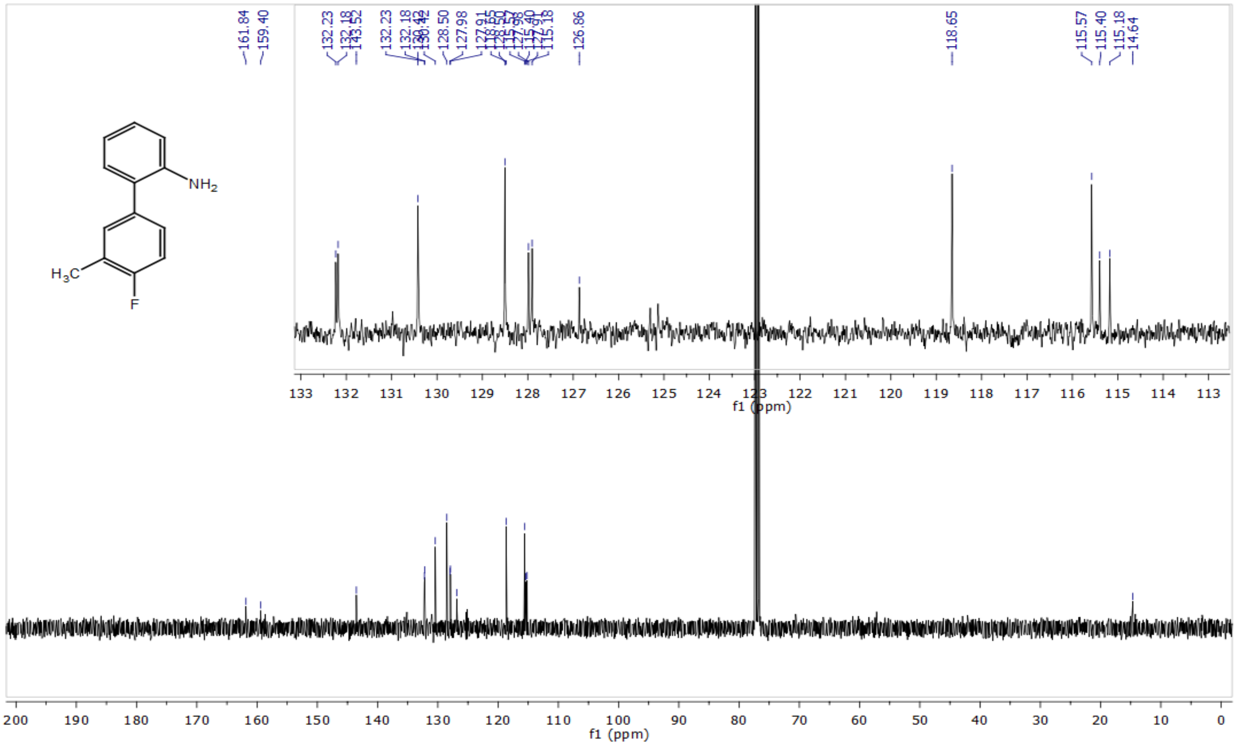

***2',6'-dimethyl-[1,1'-biphenyl]-2-amine* (2g)**

^{1}H NMR

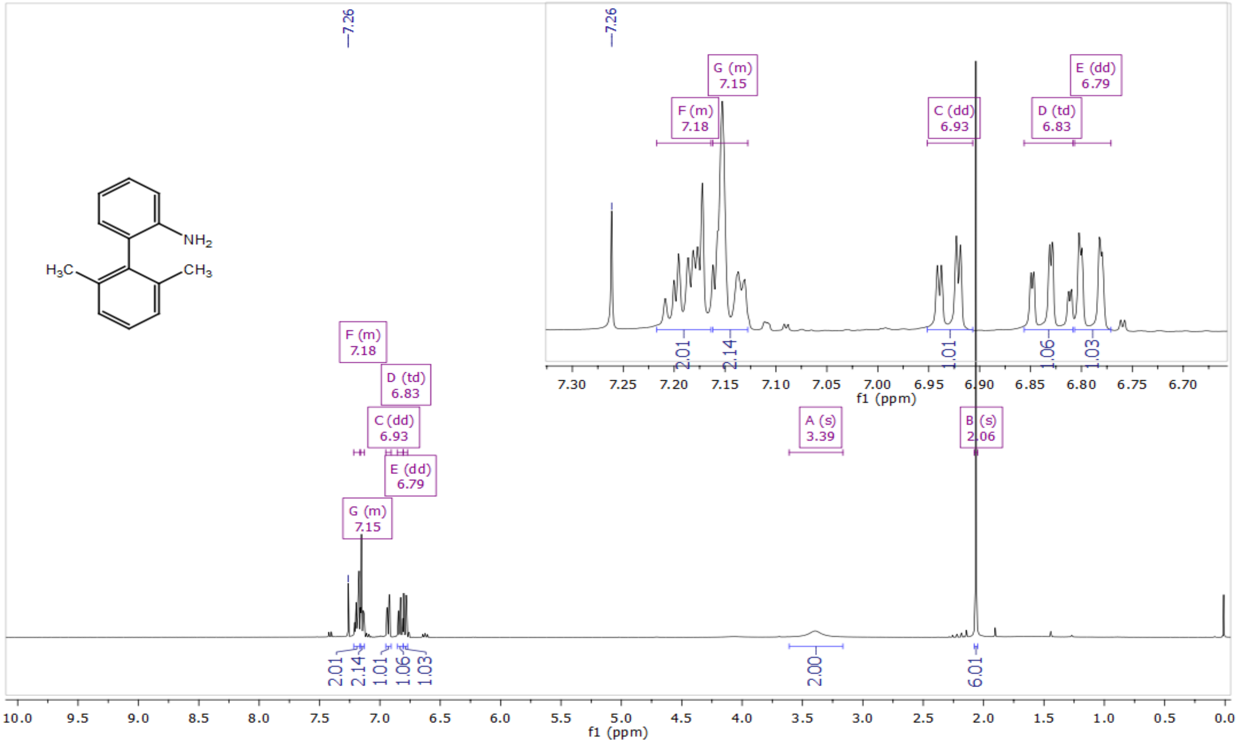

^{13}C NMR

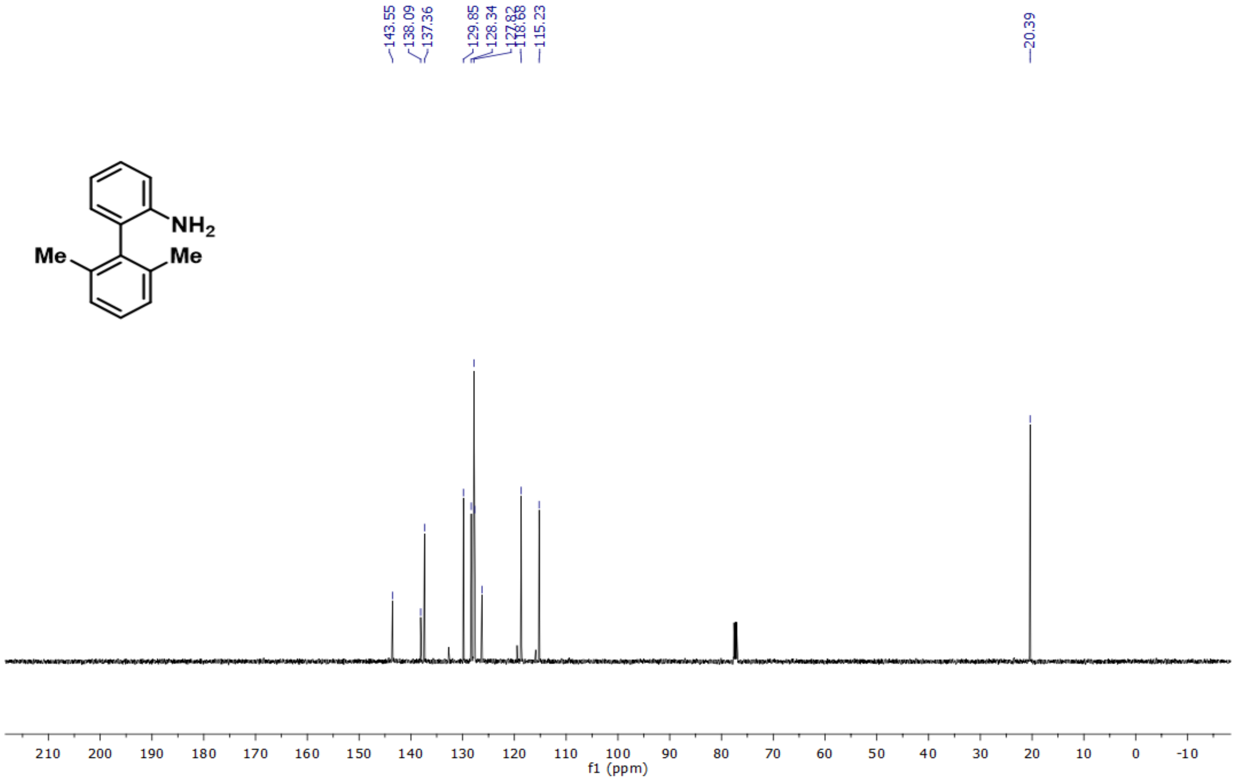

***2',5'-dimethyl-[1,1'-biphenyl]-2-amine* (2h)**

^{1}H NMR

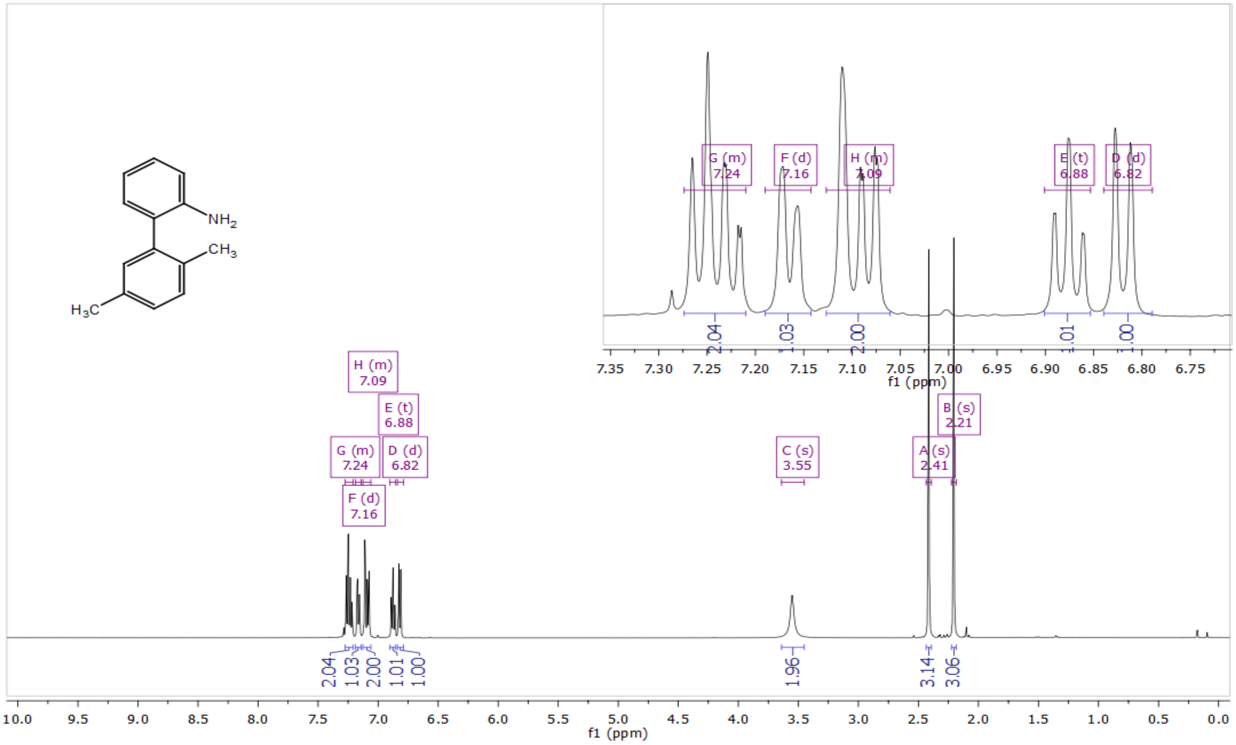

^{13}C NMR

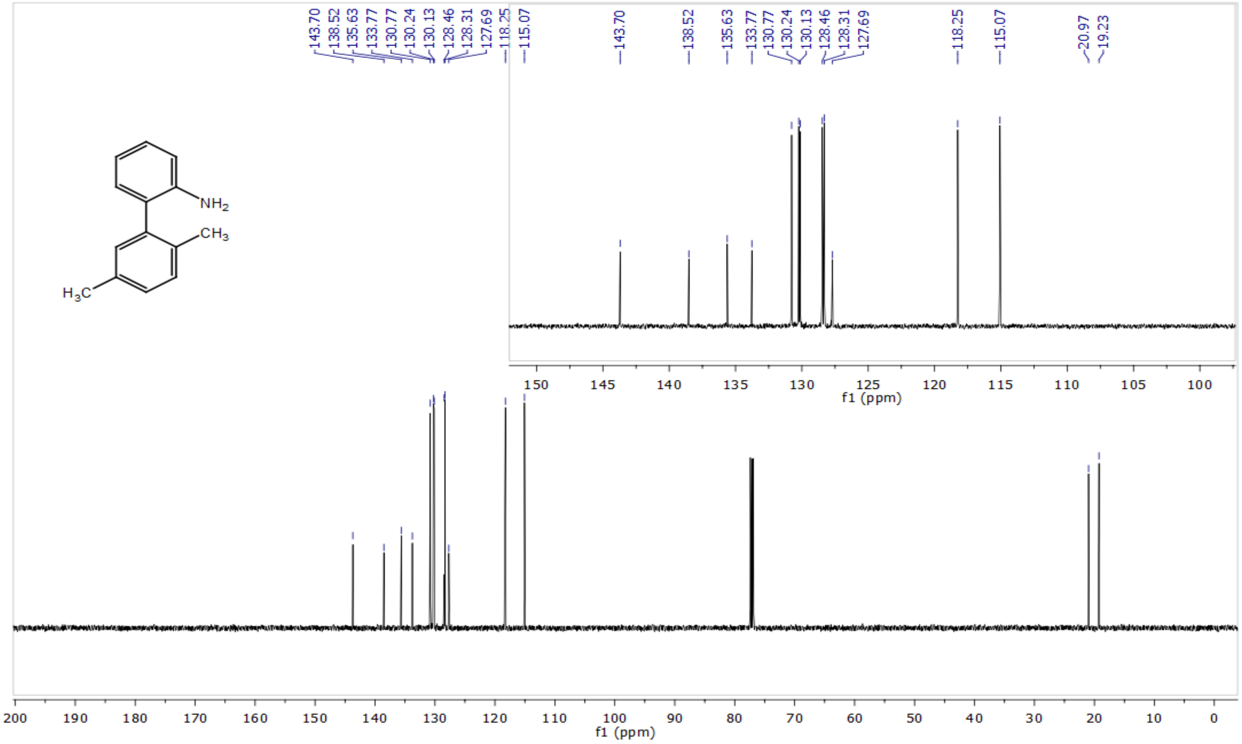

2',5'-difluoro-[1,1'-biphenyl]-2-amine **(2i)**

^{1}H NMR

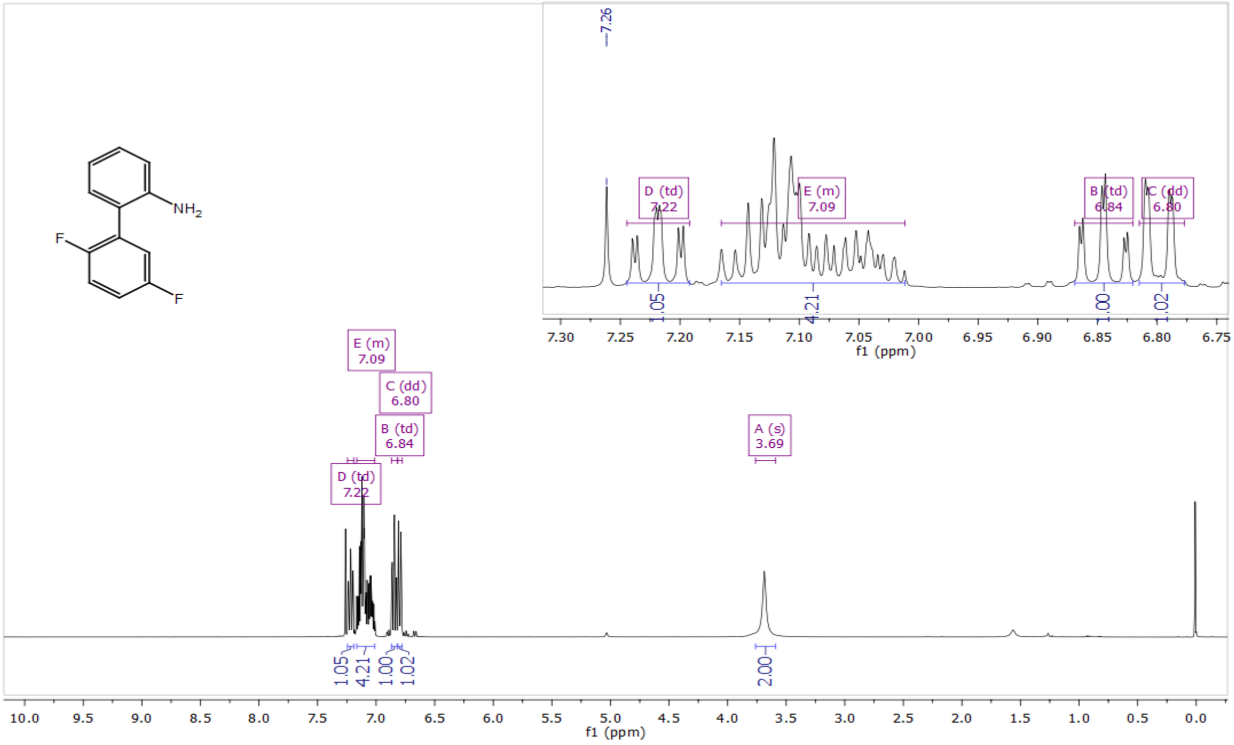

^{13}C NMR

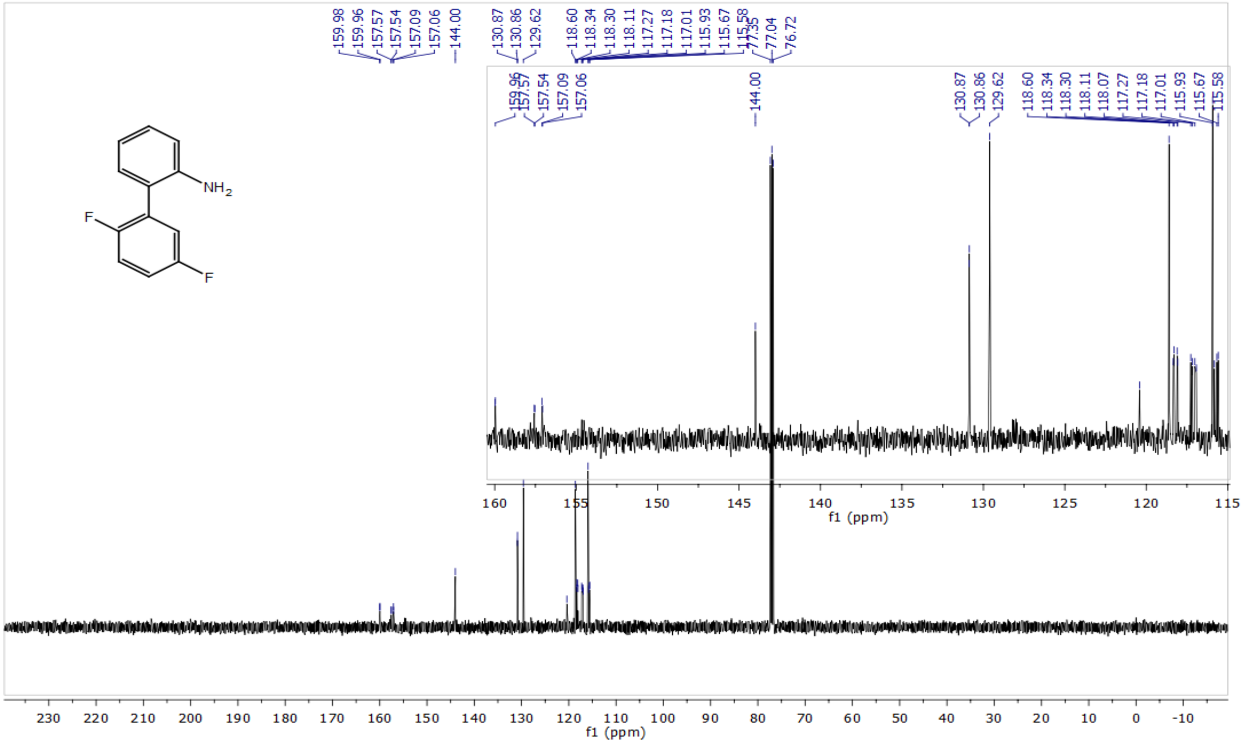

***3-fluoro-3'-methyl-[1,1'-biphenyl]-2-amine* (2j)**

^{1}H NMR

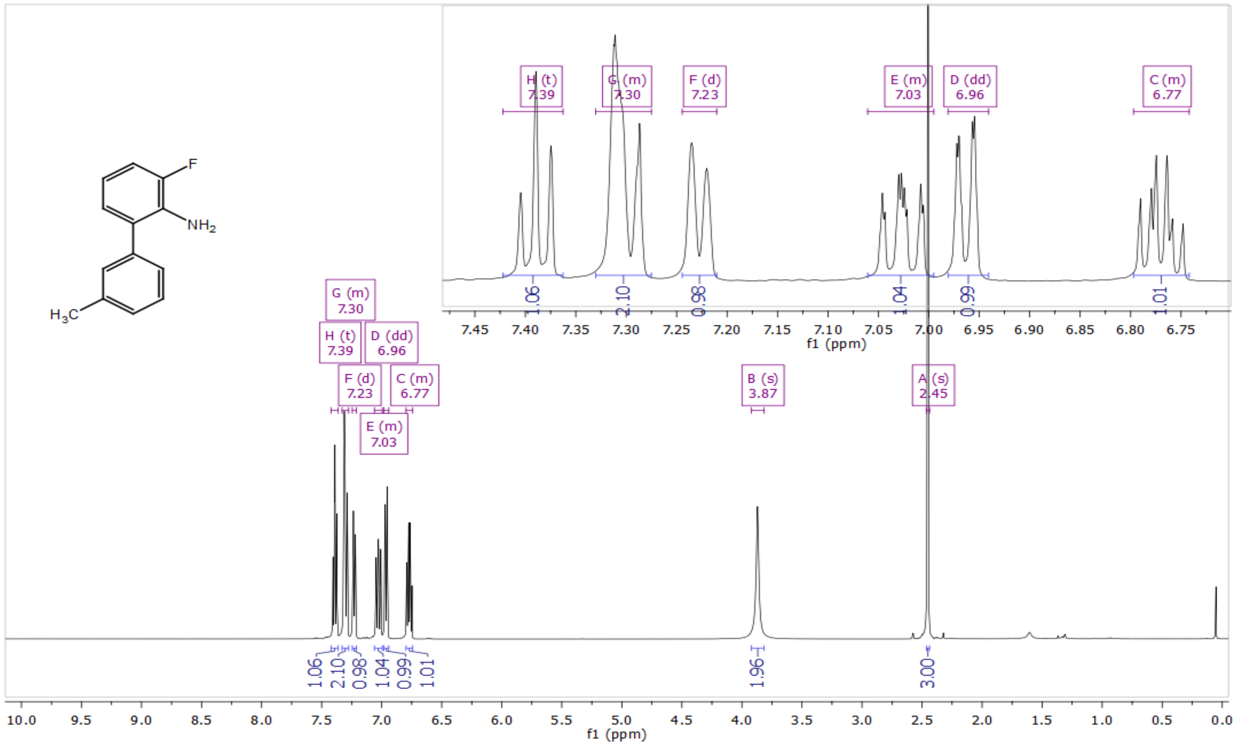

^{13}C NMR

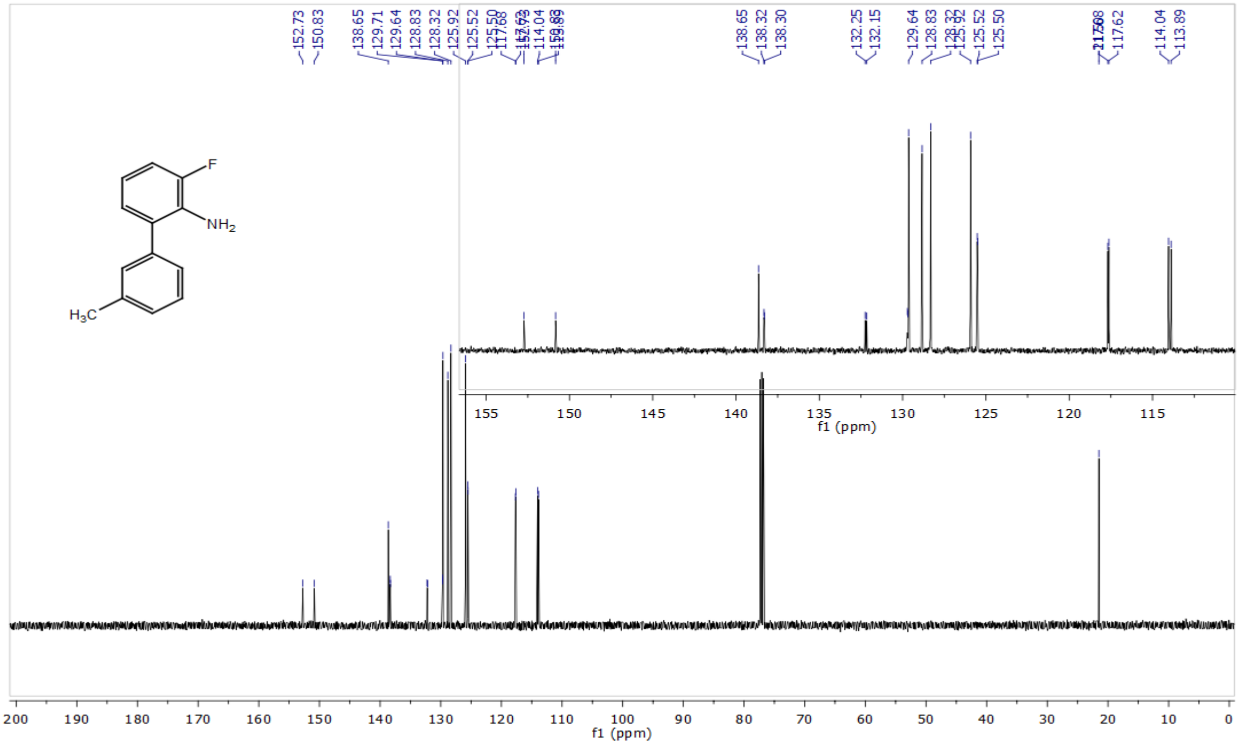

2-(1H-indol-1-yl)aniline (2k)

^{1}H NMR

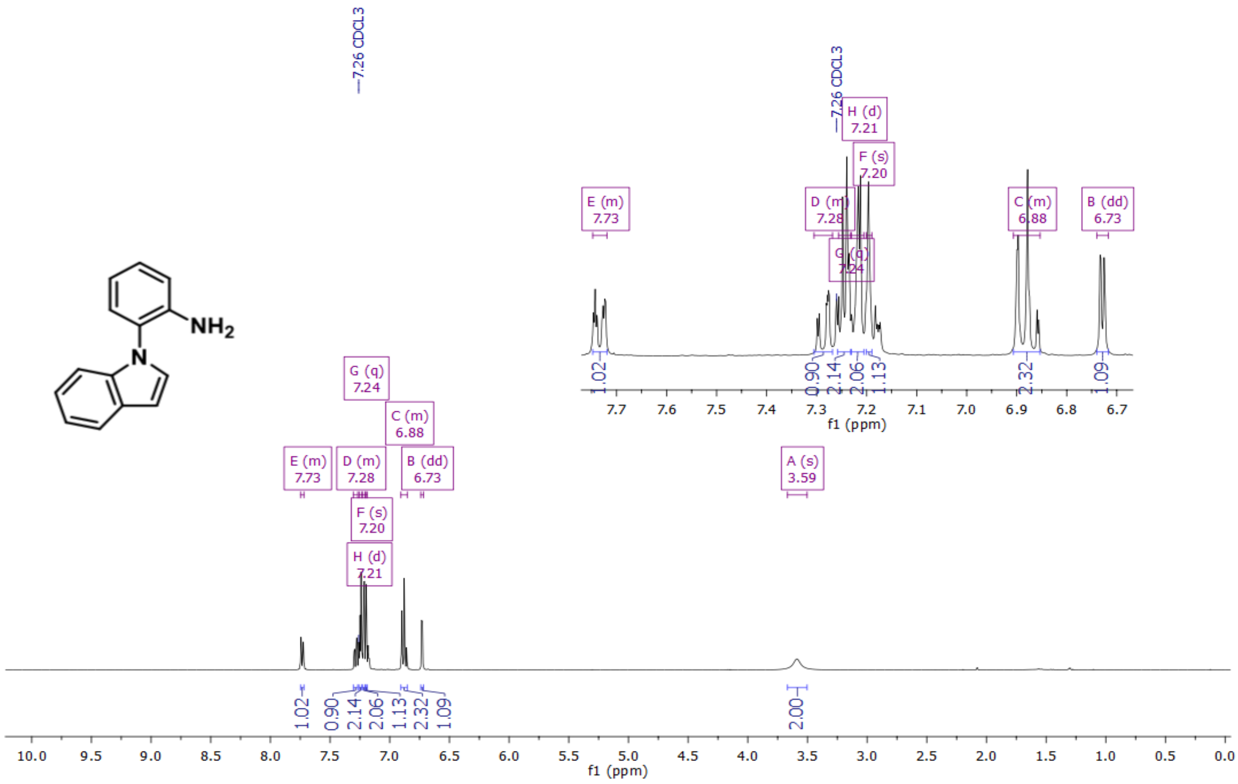

^{13}C NMR

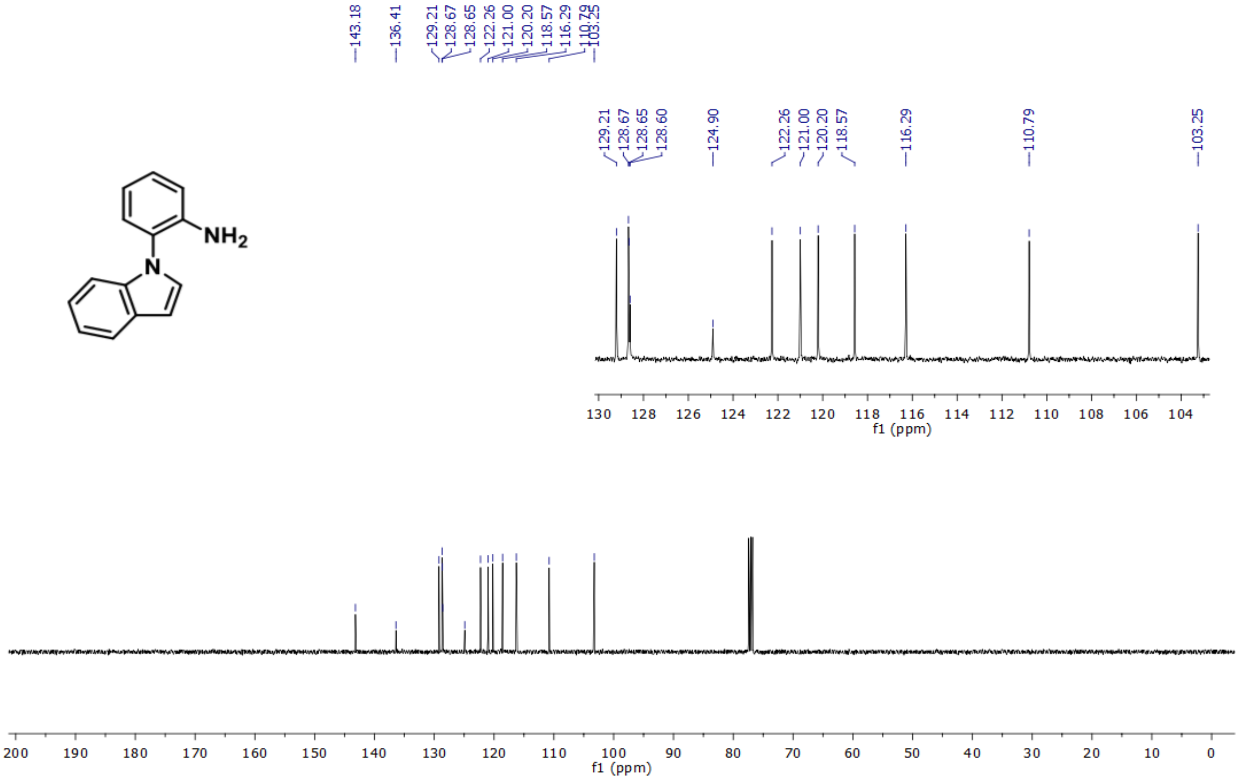

***Testosterone acrylate*:**

^{1}H NMR

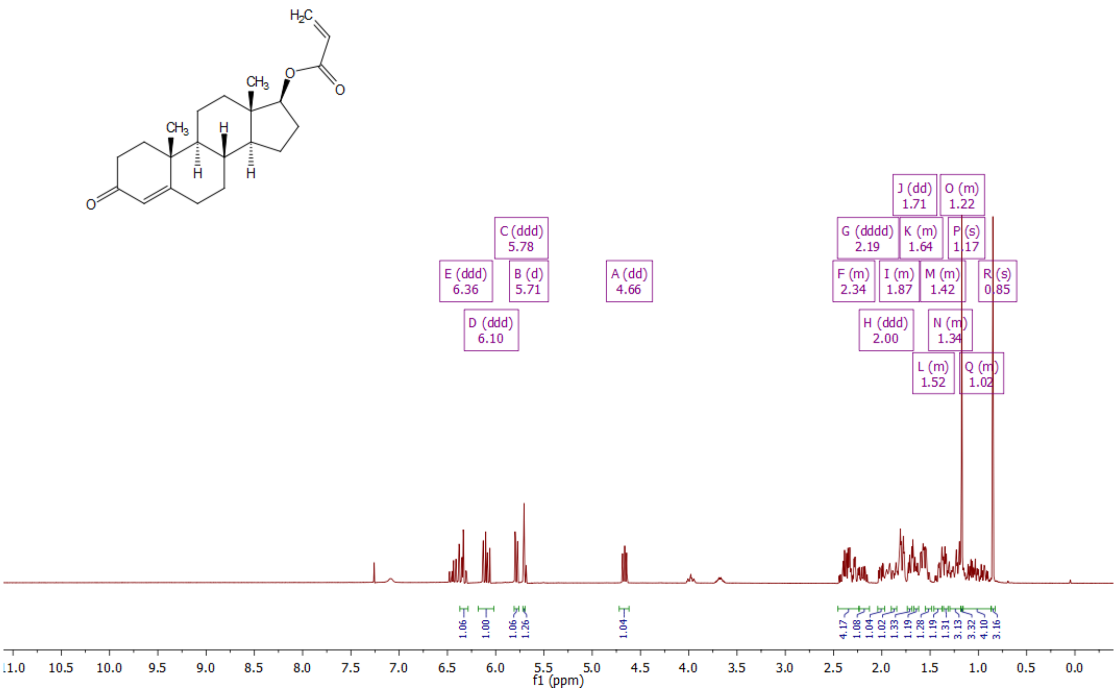

^{13}C NMR

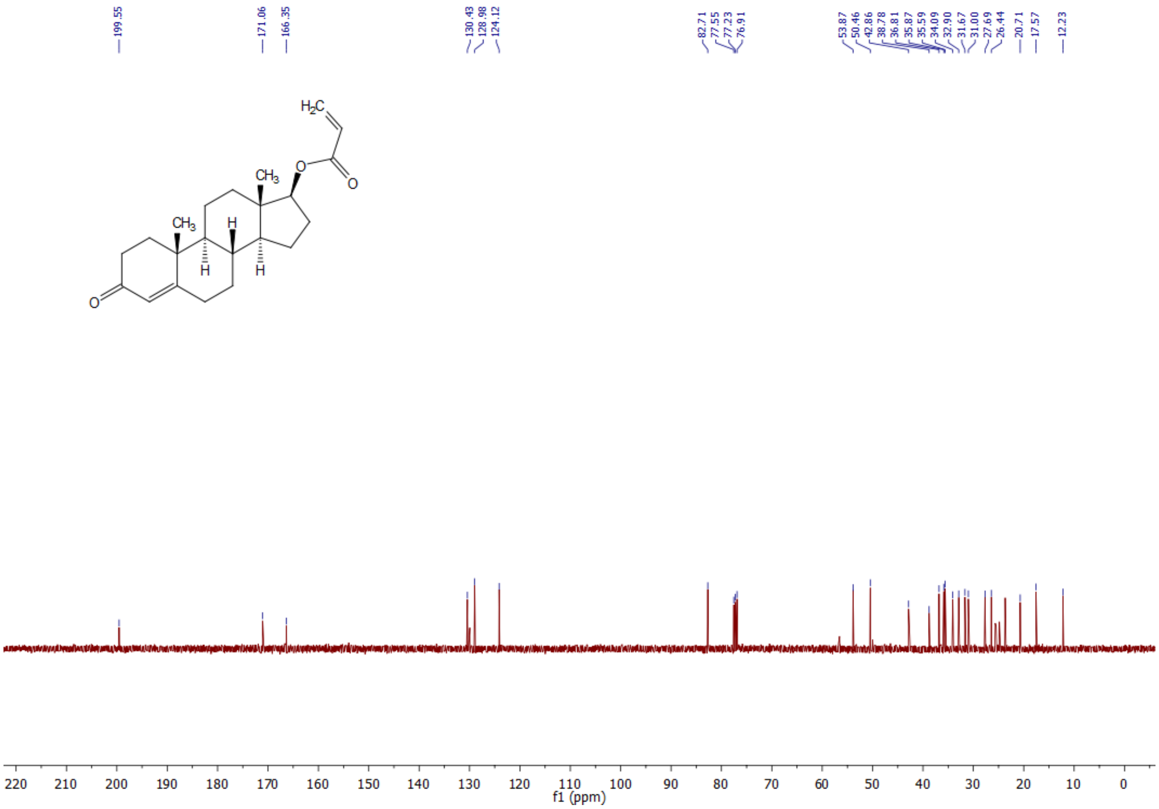

Ergosterol acrylate

^{1}H NMR

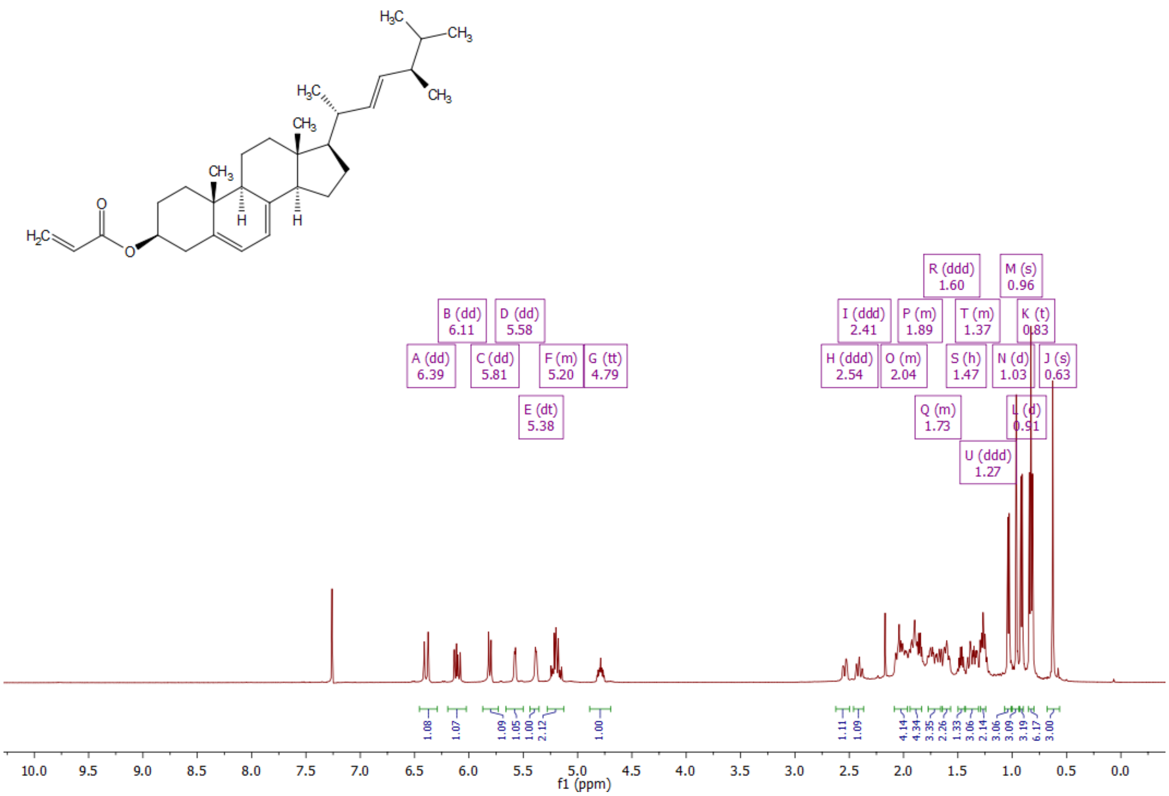

^{13}C NMR

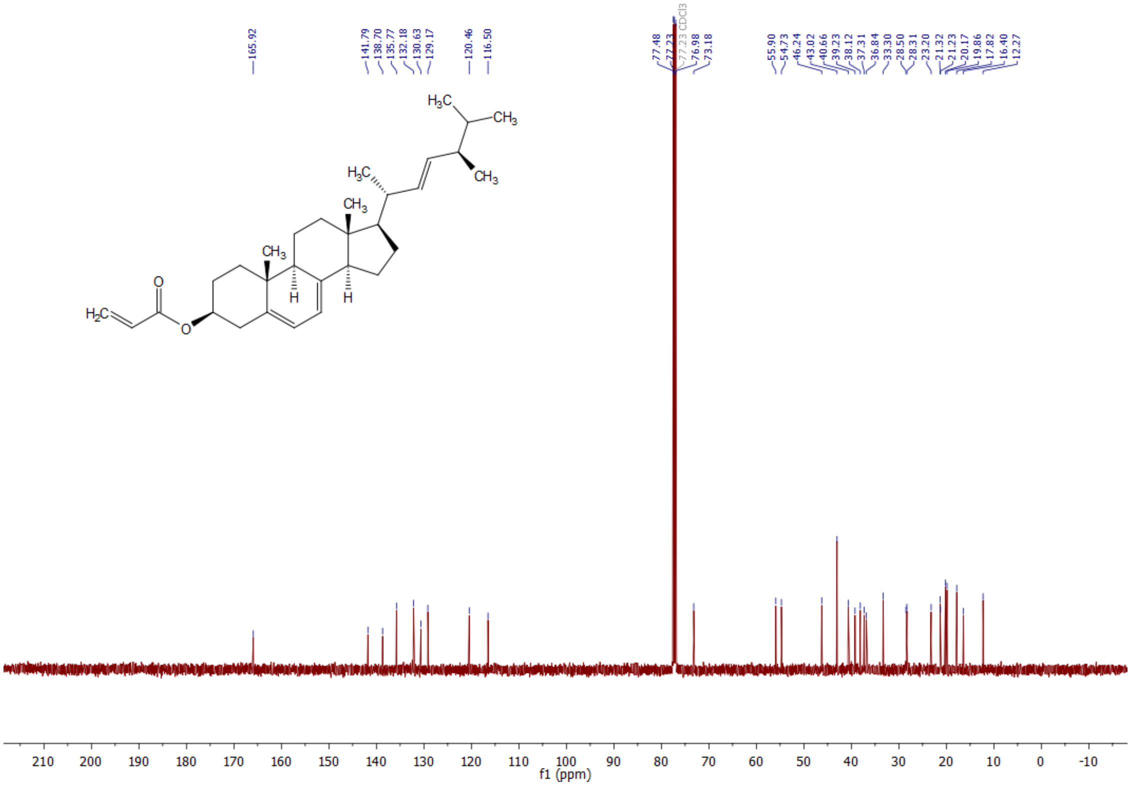

***Oleyl acrylate*:**

^{1}H NMR

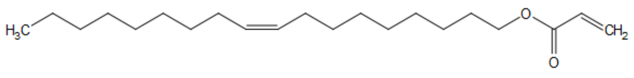

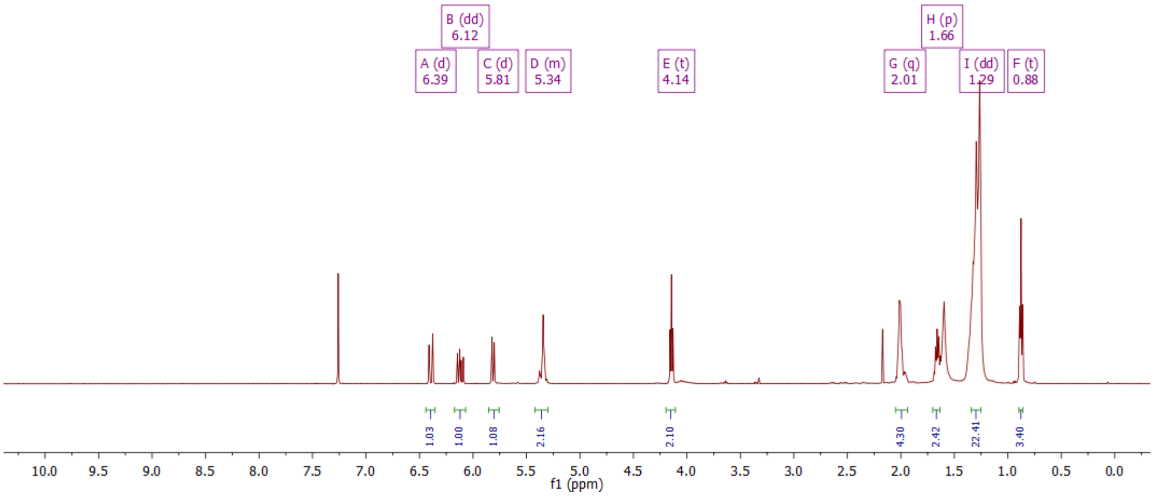

^{13}C NMR

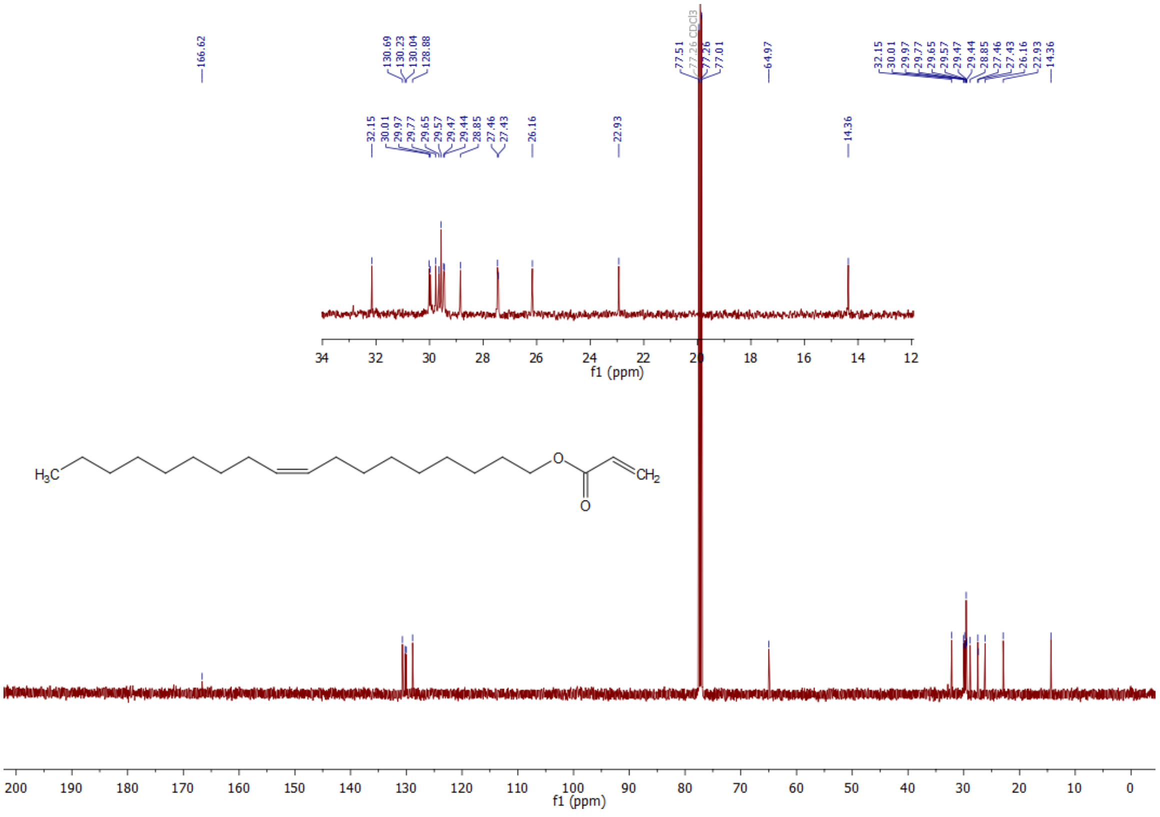

Ethyl (E)-3-(2'-formyl-5-methyl-[1,1'-biphenyl]-3-yl)acrylate **(3)**

^{1}H NMR

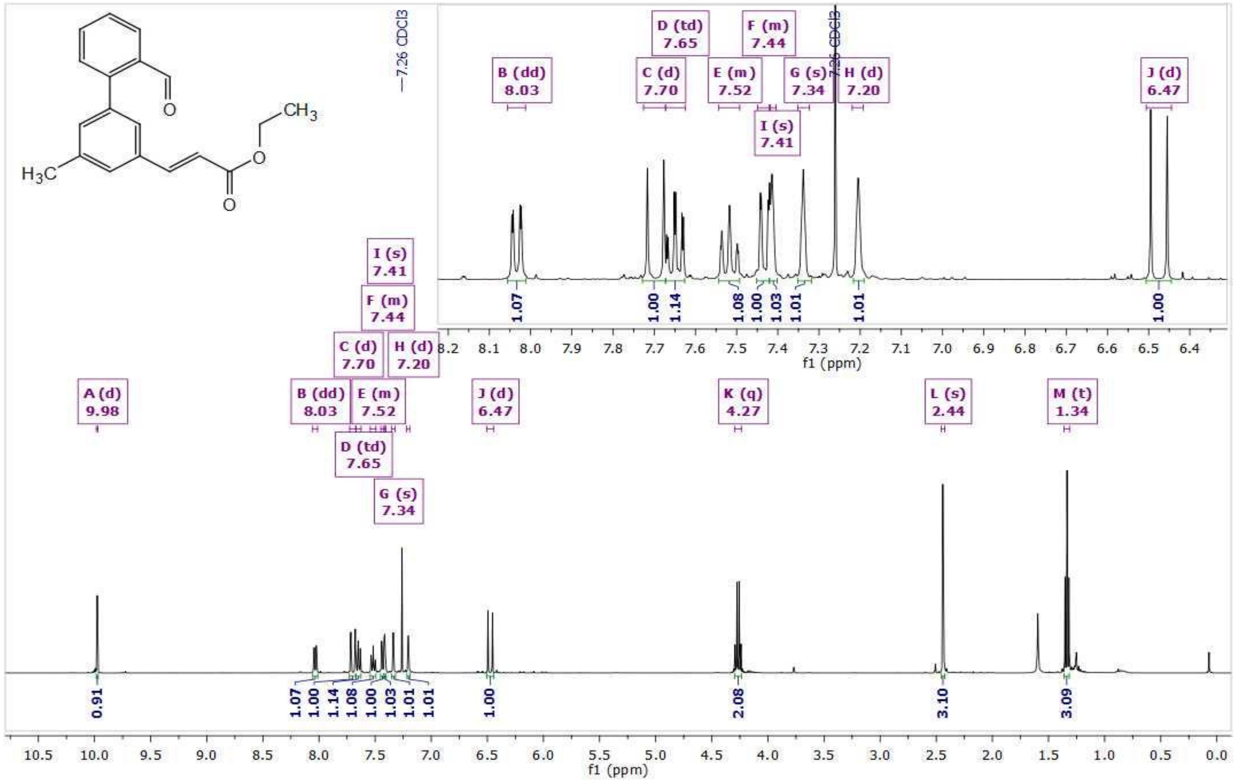

^{13}C NMR

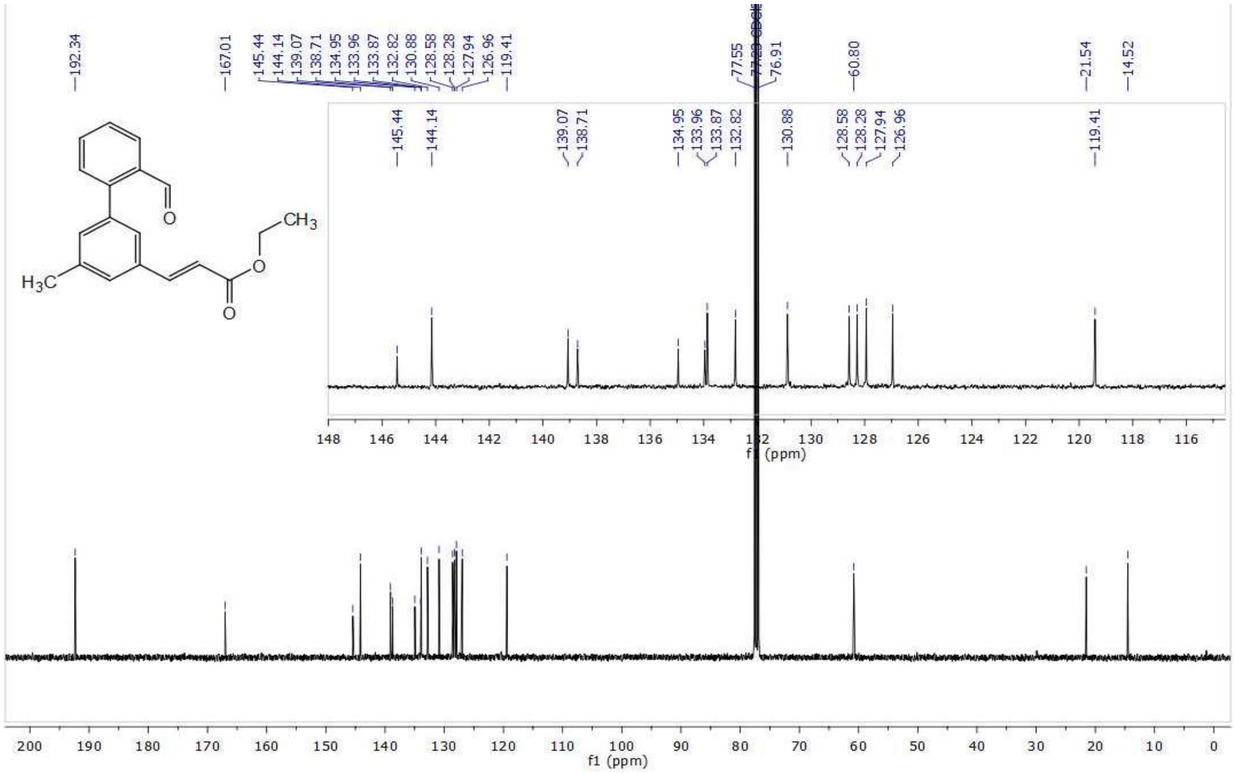

Methyl (E)-3-(2'-formyl-5-methyl-[1,1'-biphenyl]-3-yl)acrylate (**4**)

^{1}H NMR

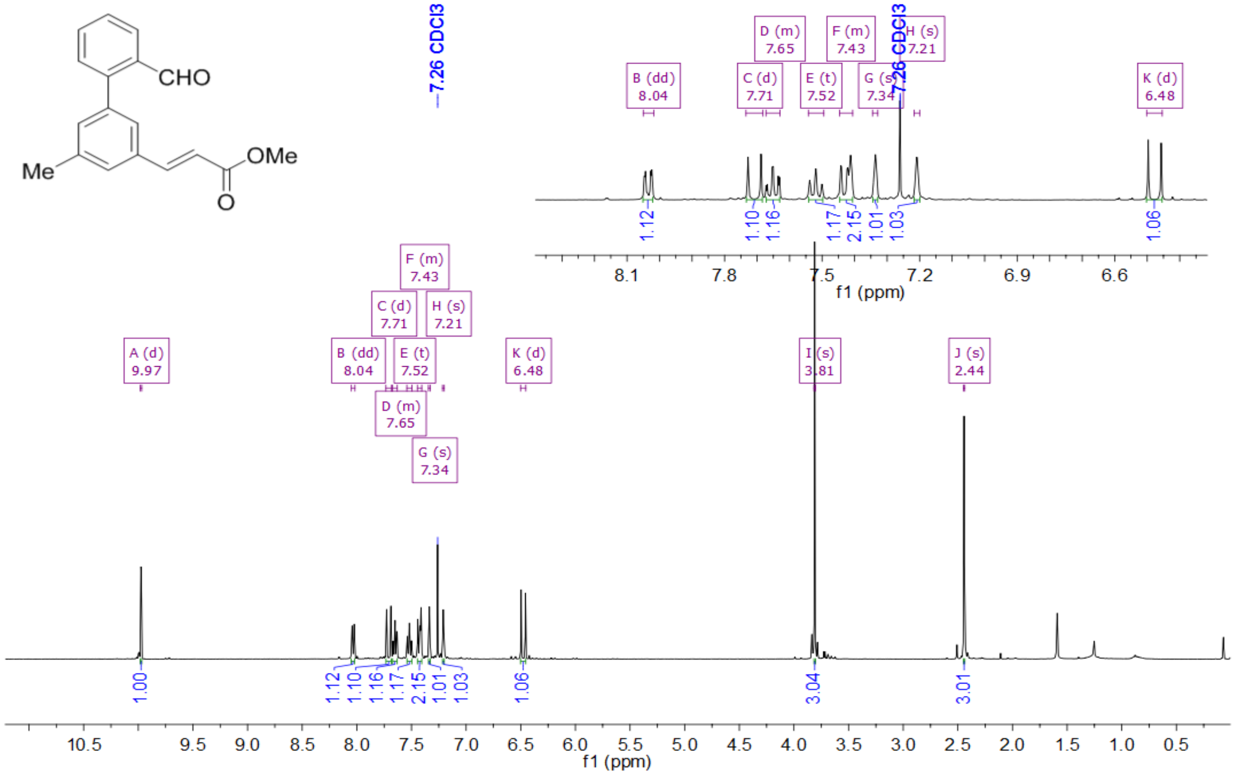

^{13}C NMR

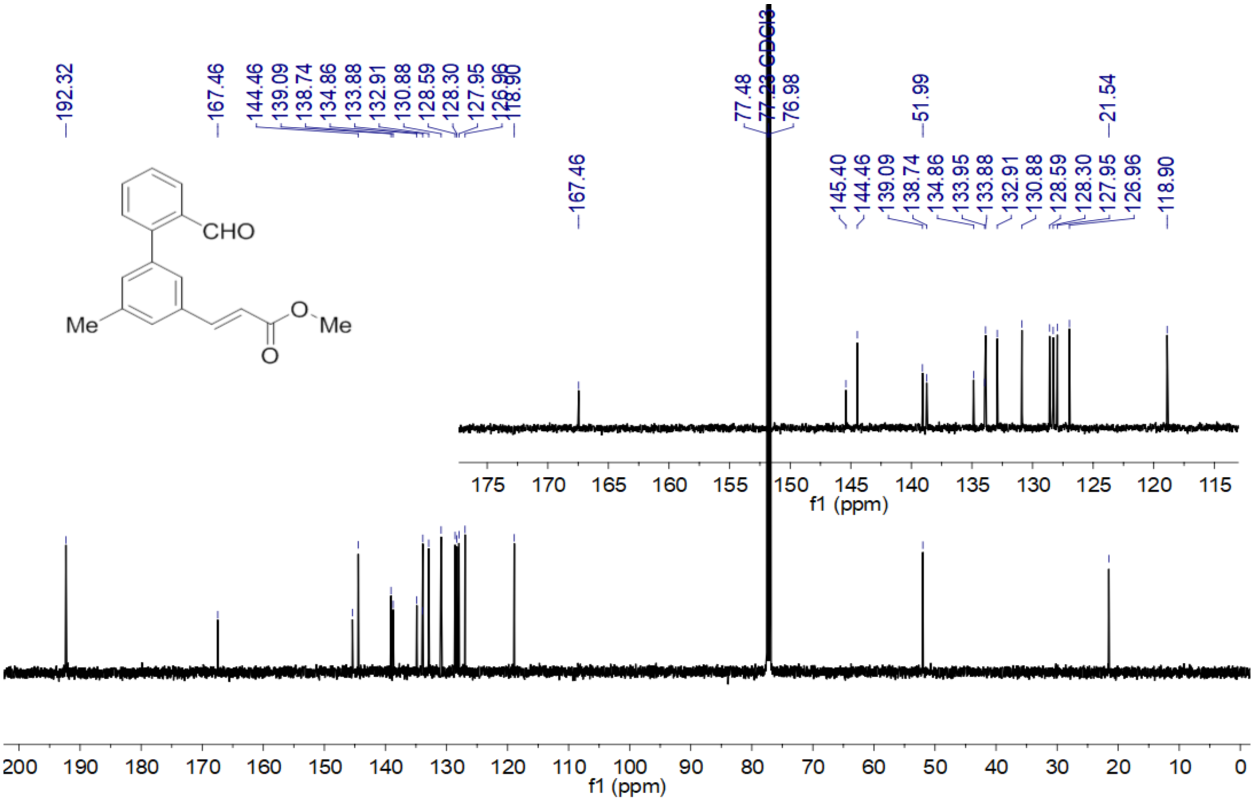

***2,2,2-trifluoroethyl (E)-3-(2'-formyl-5-methyl-[1,1'-biphenyl]-3-yl)acrylate* (5)**

^{1}H NMR

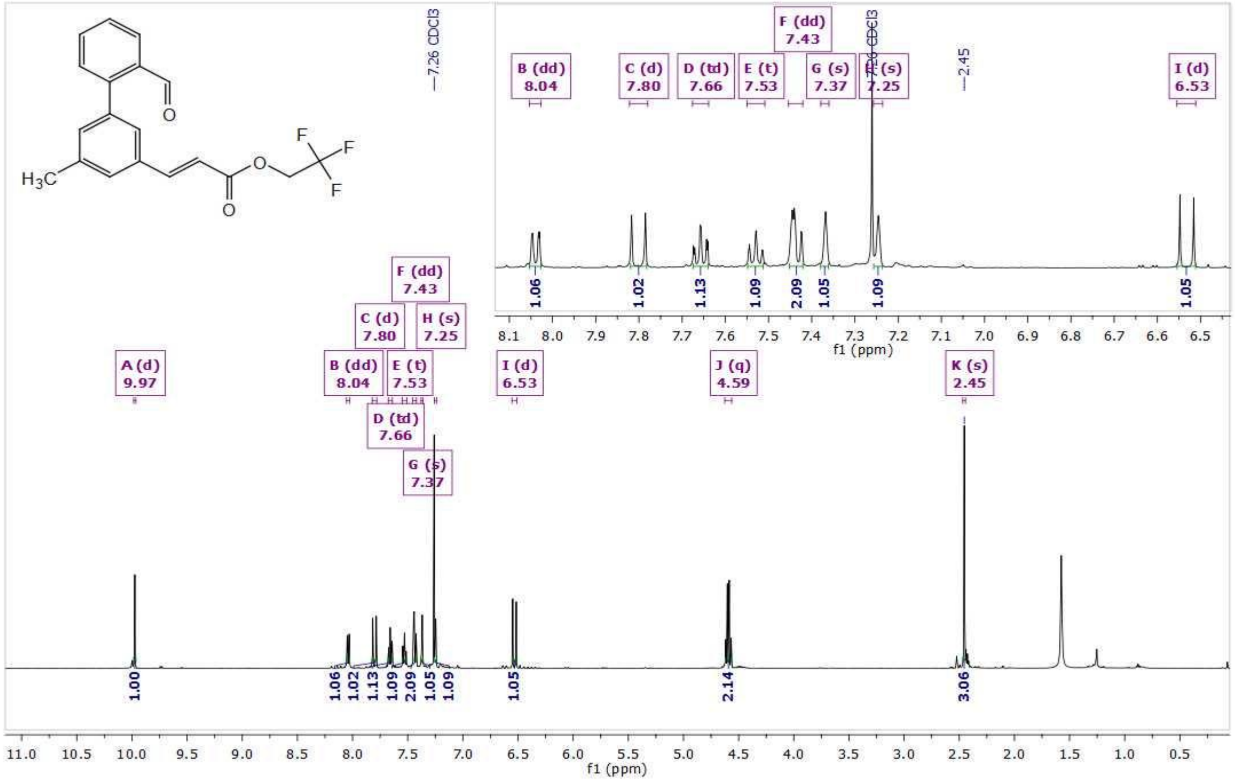

^{13}C NMR

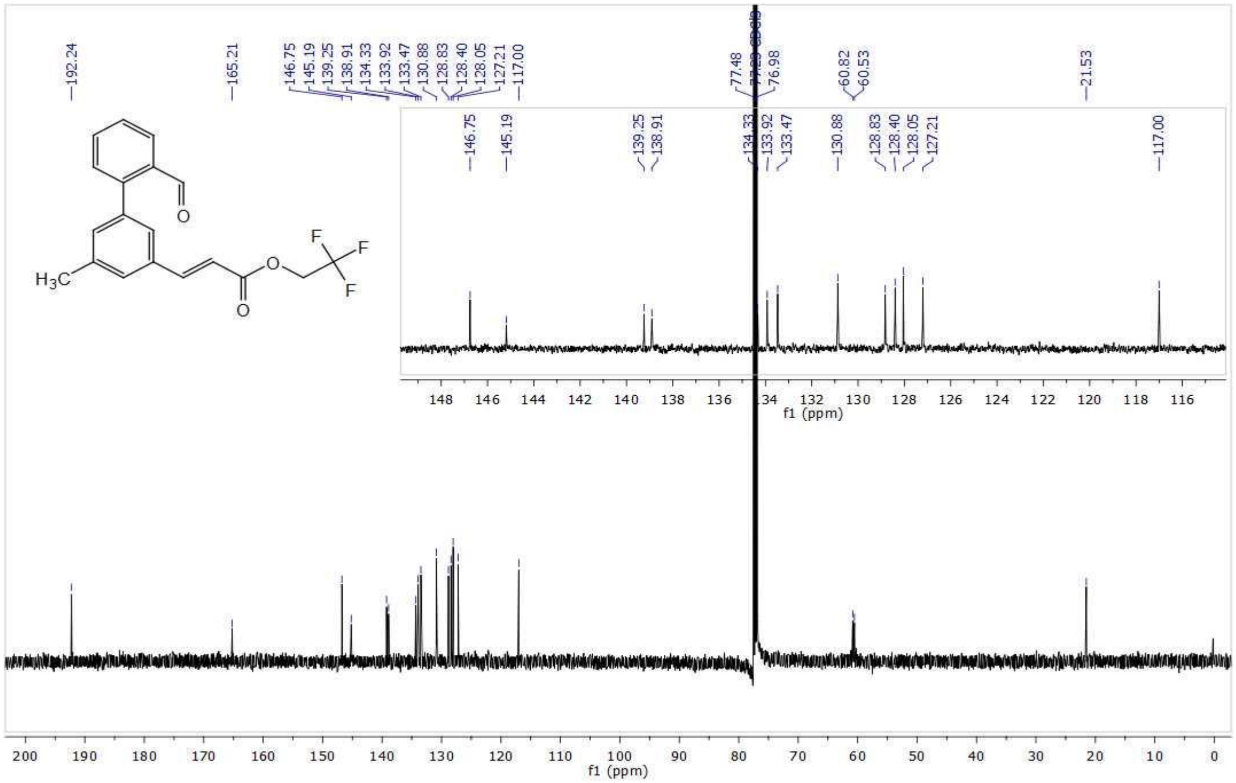

^{19}F NMR

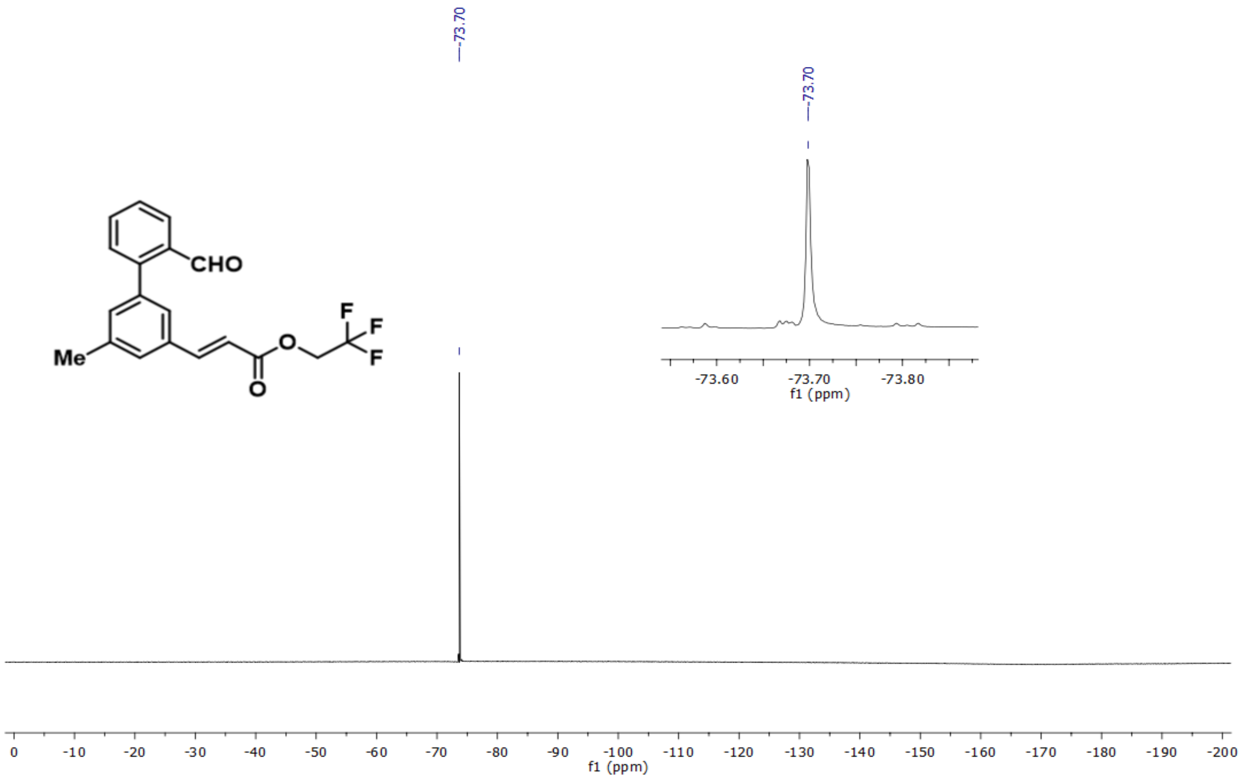
-73.70
CHO
Me
O
O
F
F
F
-73.70
-73.60
-73.70
-73.80
f1 (ppm)
0
-10
-20
-30
-40
-50
-60
-70
-80
-90
-100
-110
-120
-130
-140
-150
-160
-170
-180
-190
-200
f1 (ppm)

***Butyl (E)-3-(2'-formyl-5-methyl-[1,1'-biphenyl]-3-yl)acrylate* (6)**

^{1}H NMR

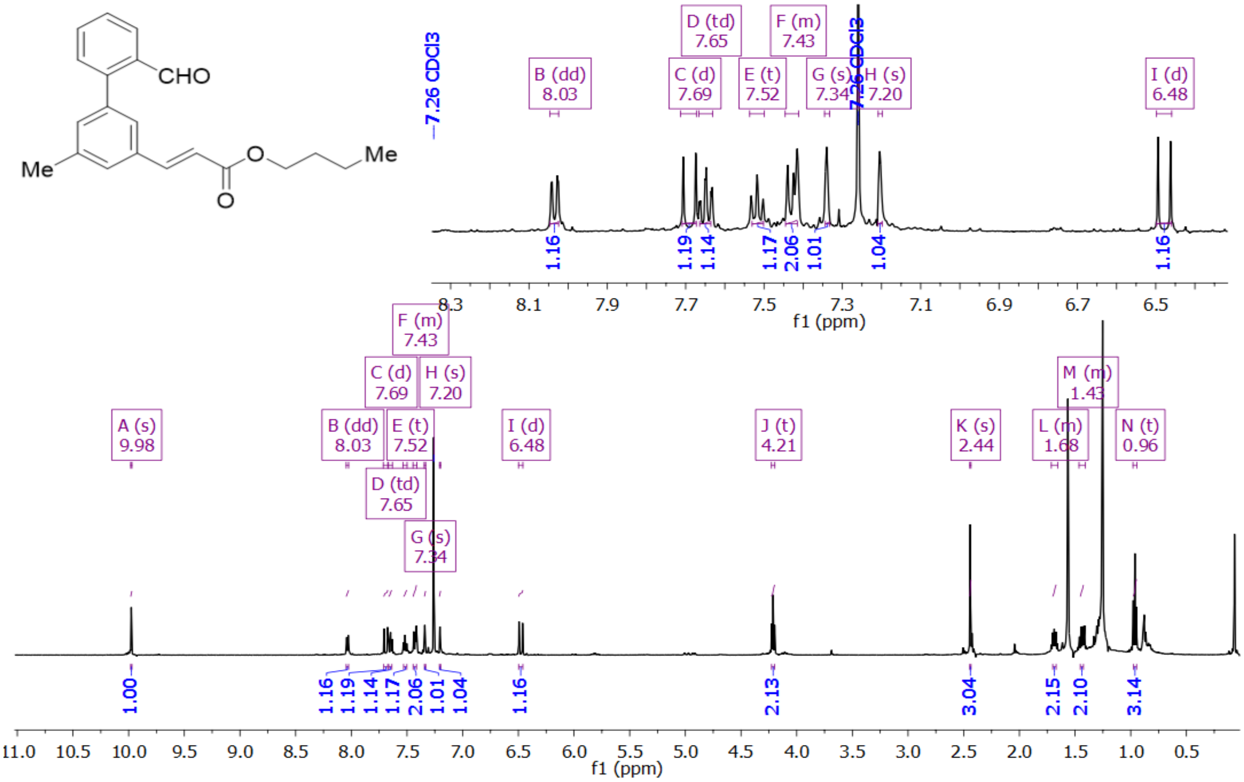

^{13}C NMR

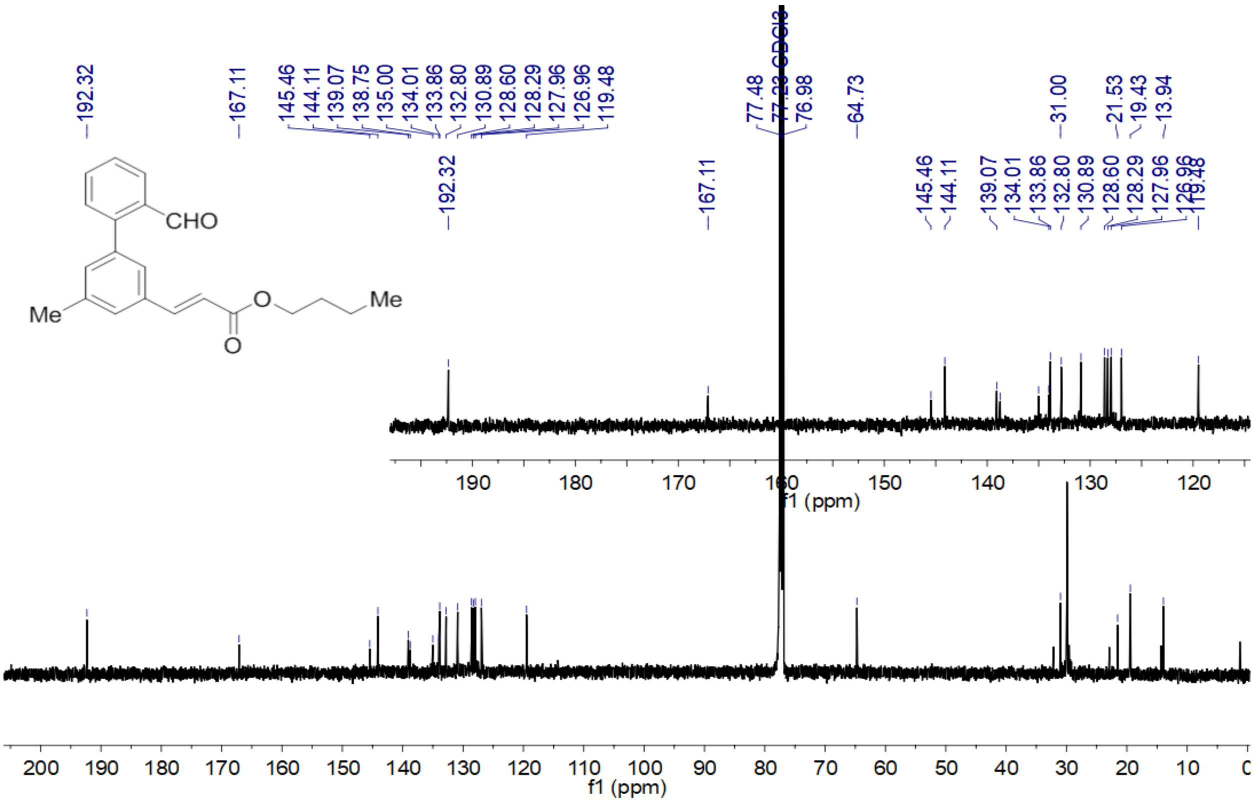

***Tert-butyl (E)-3-(2'-formyl-5-methyl-[1,1'-biphenyl]-3-yl)acrylate* (7)**

^{1}H NMR

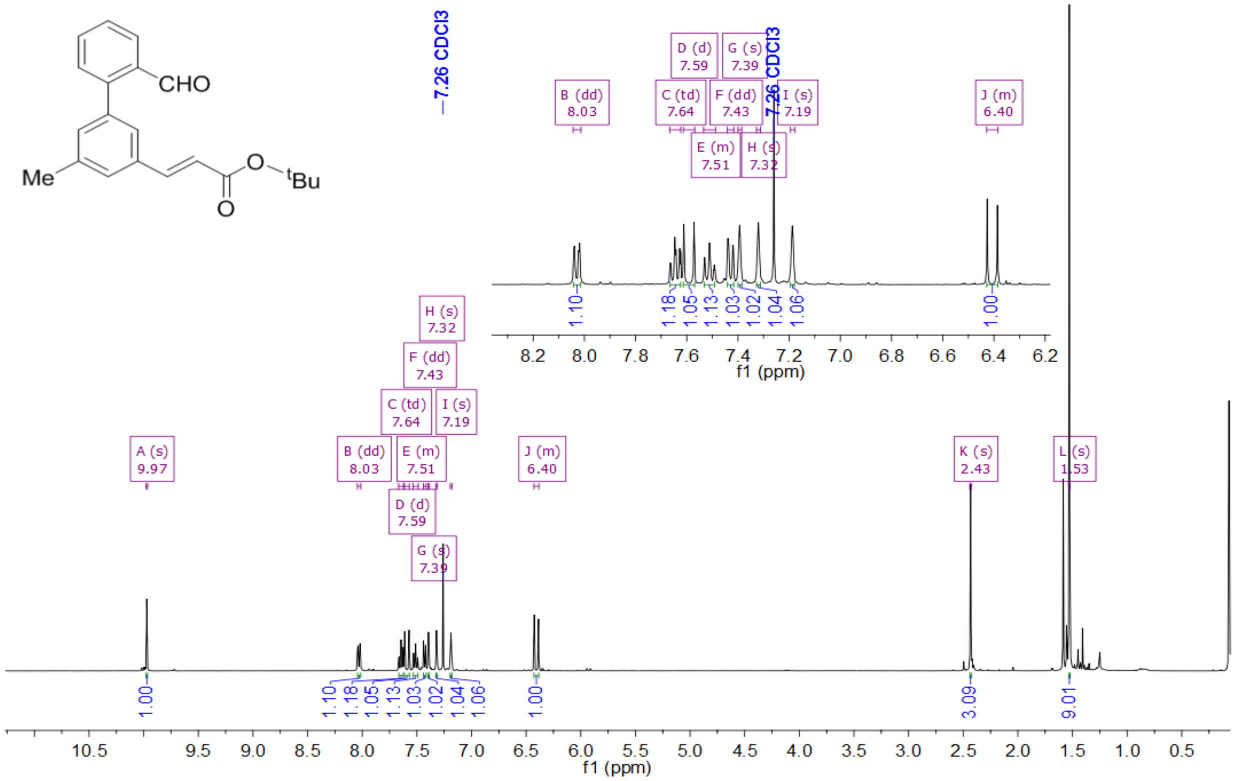

^{13}C NMR

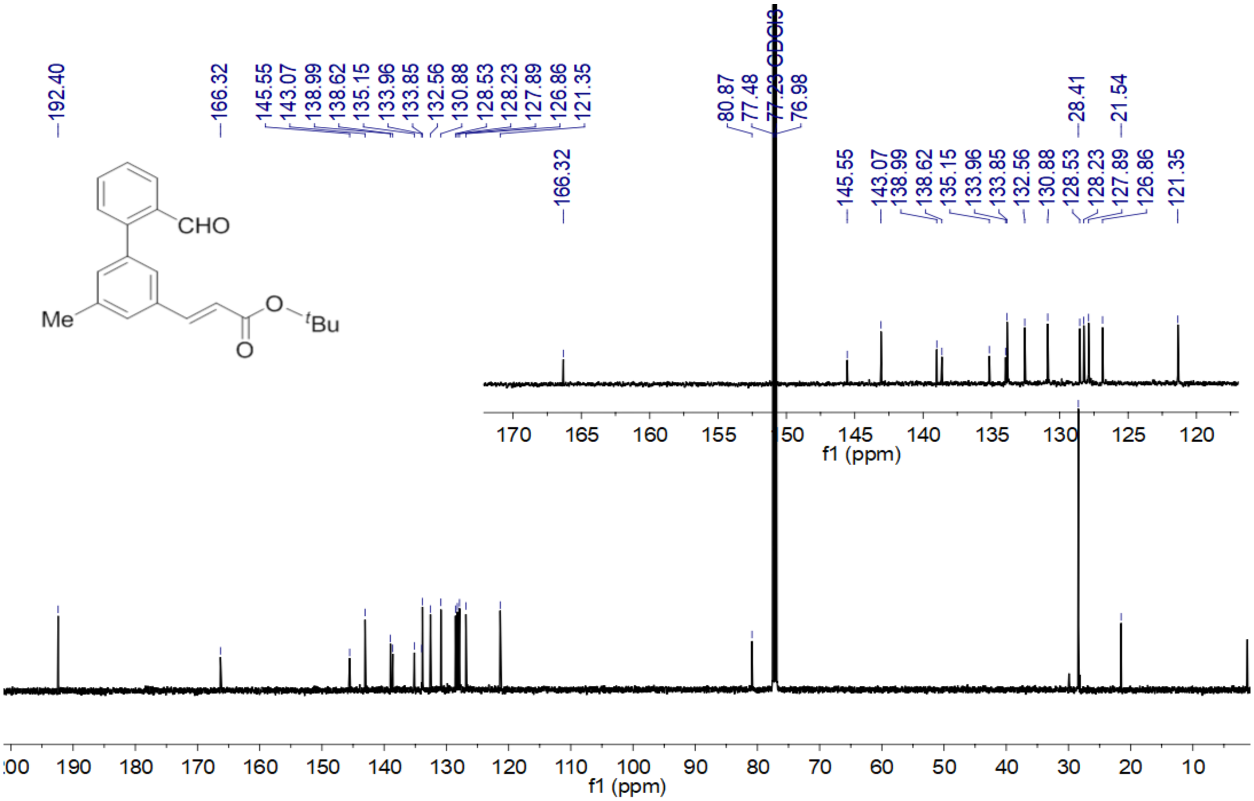

***Dodecyl (E)-3-(2'-formyl-5-methyl-[1,1'-biphenyl]-3-yl)acrylate* (8)**

^{1}H NMR

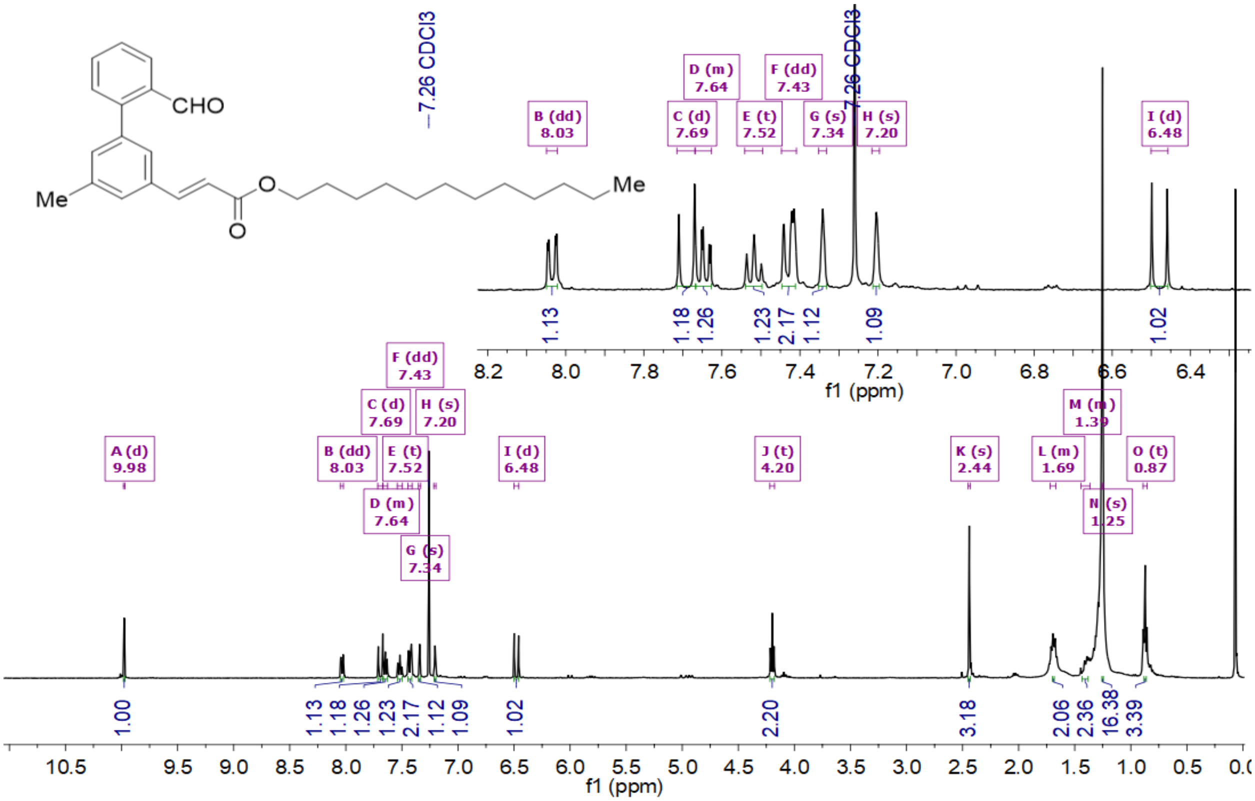

^{13}C NMR

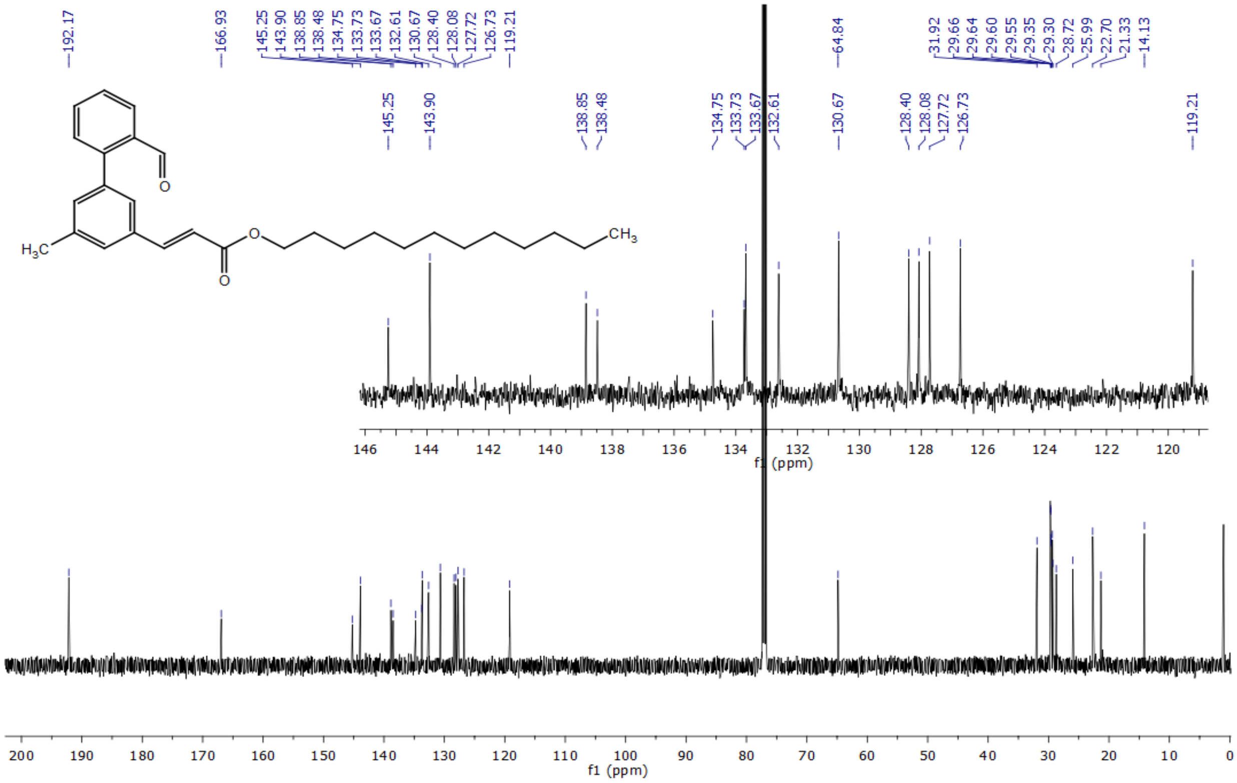

***Methyl (E)-3-(2'-formyl-5-methyl-[1,1'-biphenyl]-3-yl)but-2-enoate* (9)**

^{1}H NMR

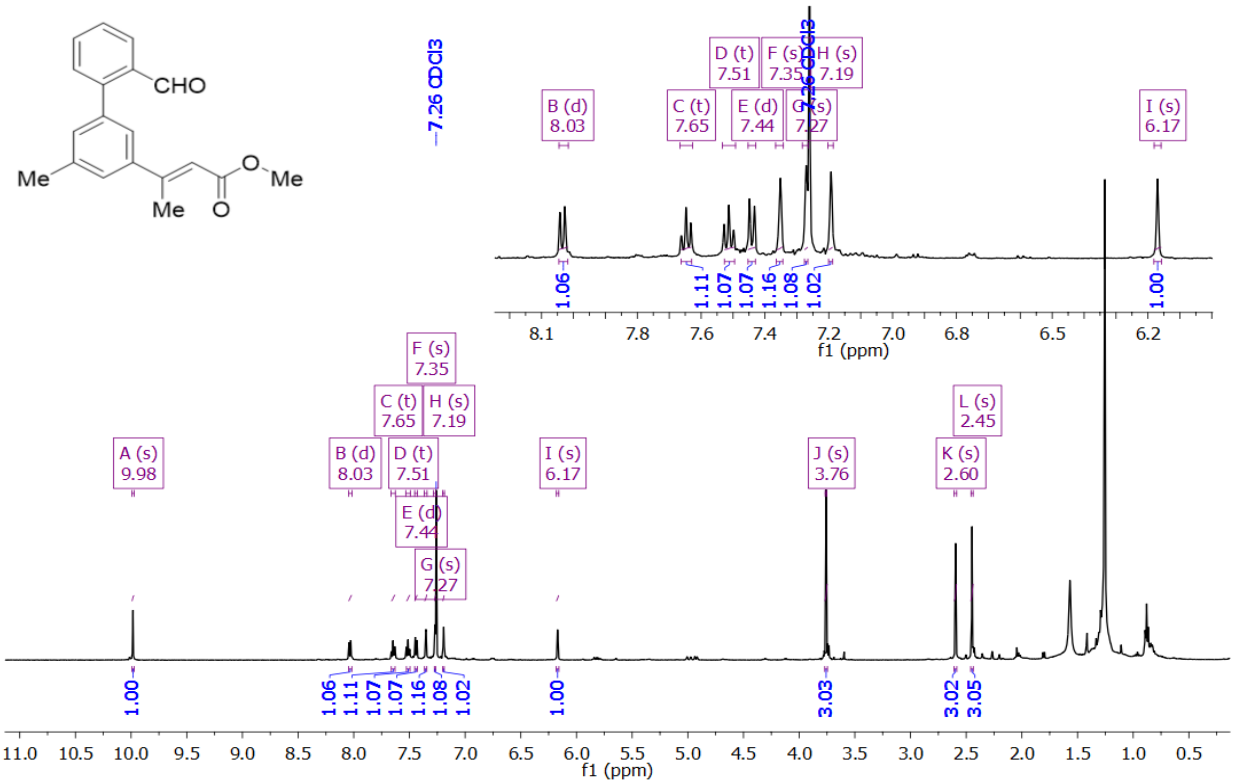

^{13}C NMR

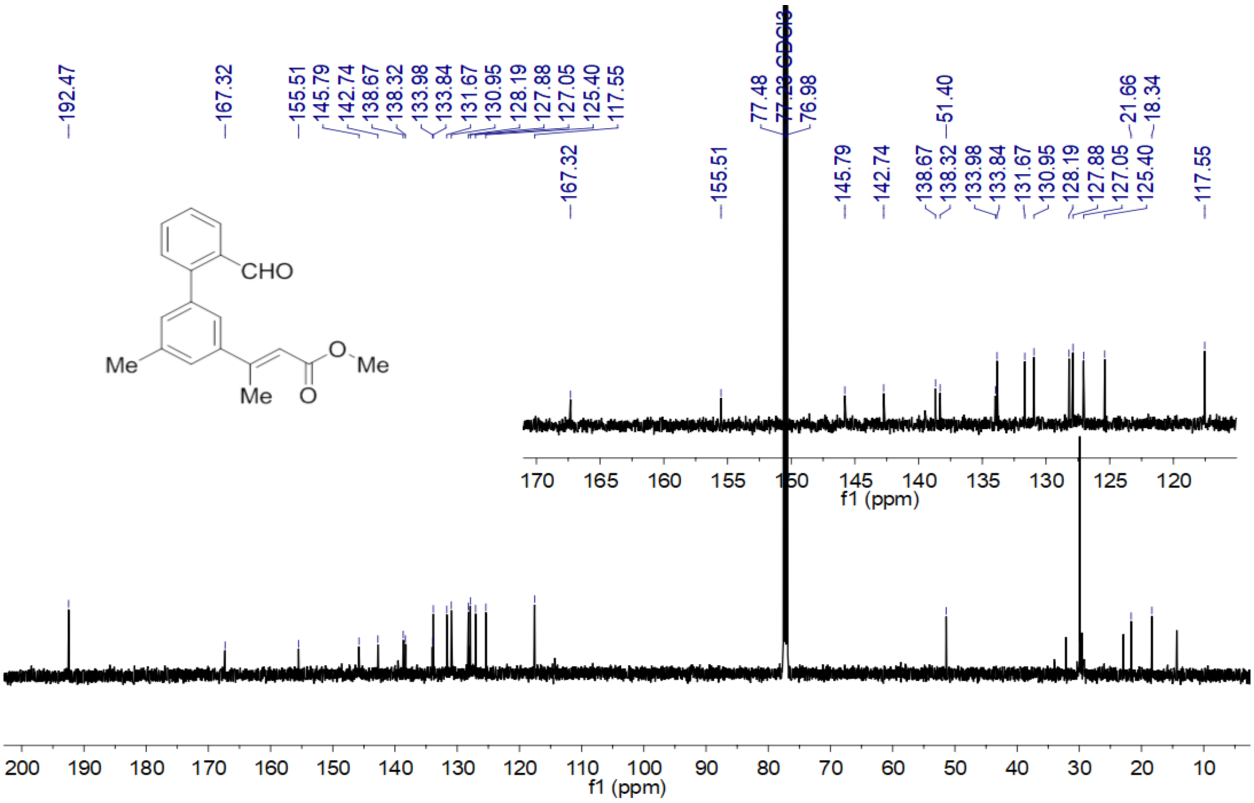

***Benzyl (E)-3-(2'-formyl-5-methyl-[1,1'-biphenyl]-3-yl)-3-phenylacrylate* (10)**

^{1}H NMR

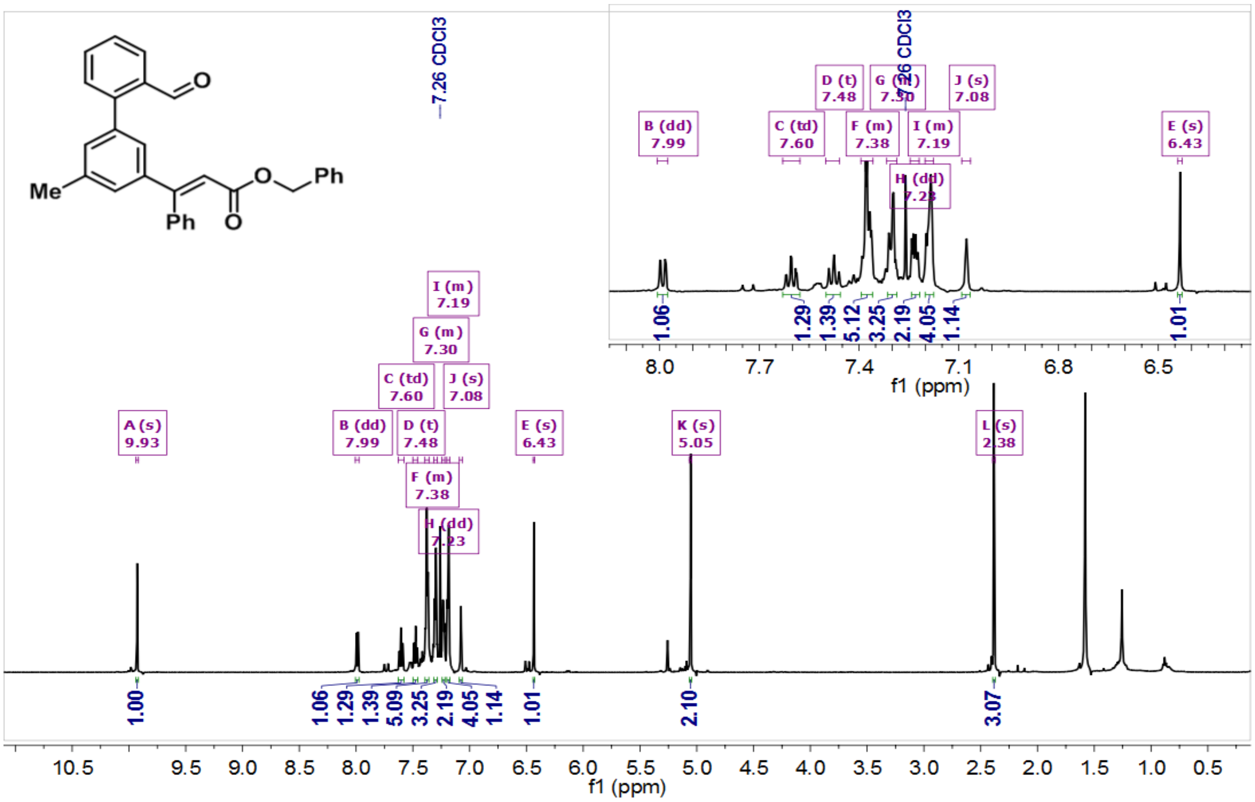

^{13}C NMR

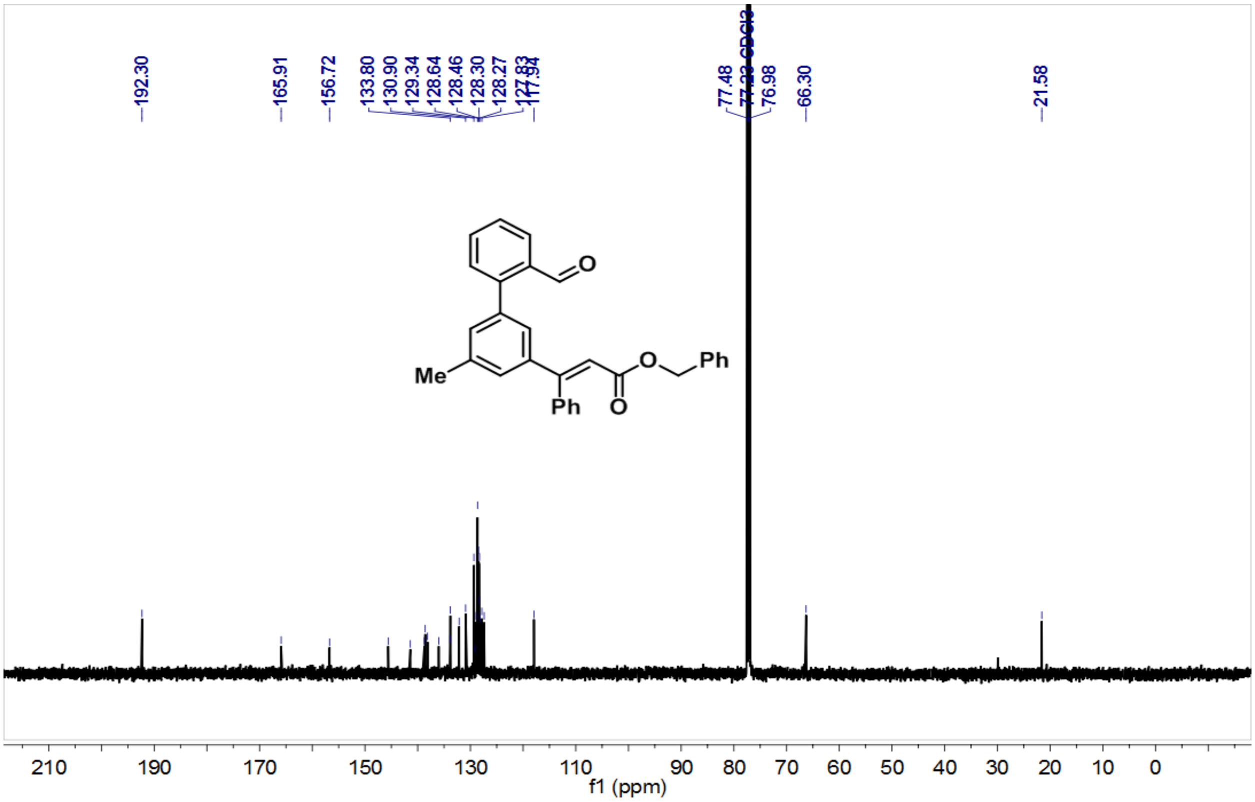

***Dimethyl 2-(2'-formyl-5-methyl-[1,1'-biphenyl]-3-yl)fumarate* (11)**

^{1}H NMR

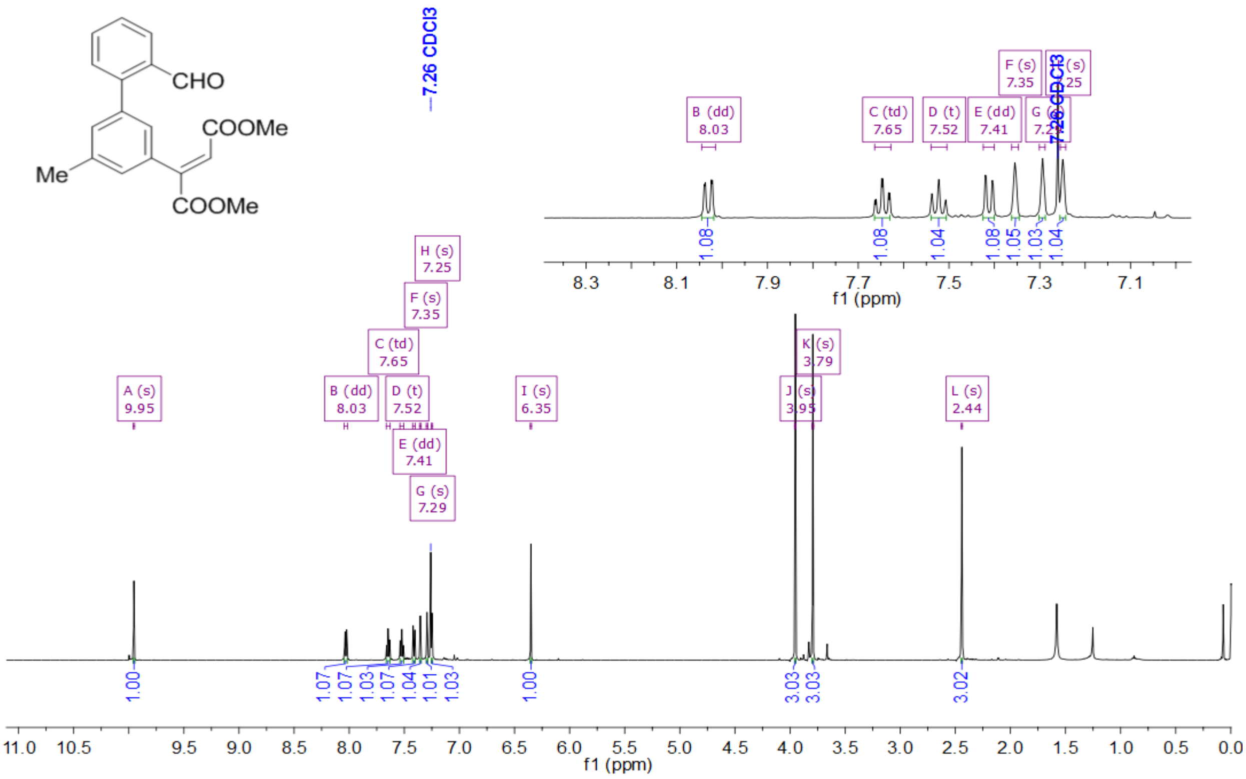

^{13}C NMR

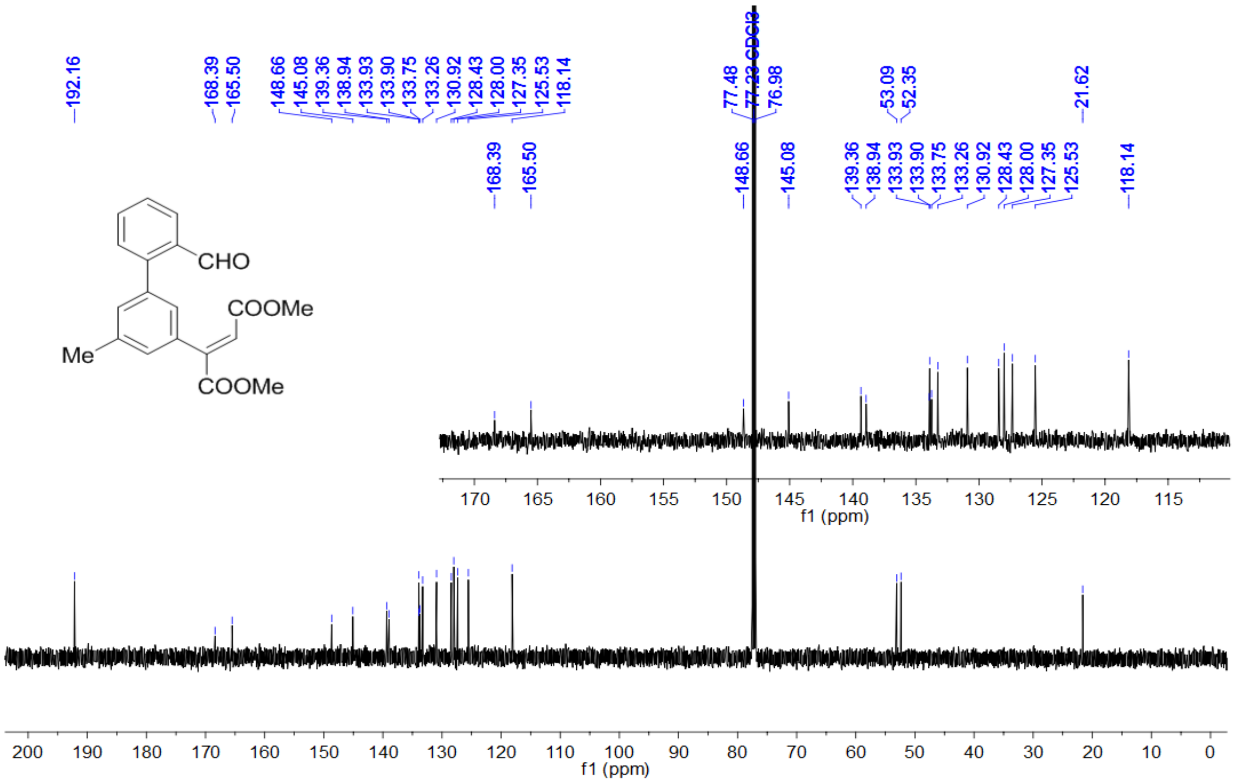

(E)-3'-methyl-5'-(3-oxobut-1-en-1-yl)-[1,1'-biphenyl]-2-carbaldehyde (**12**)

^{1}H NMR

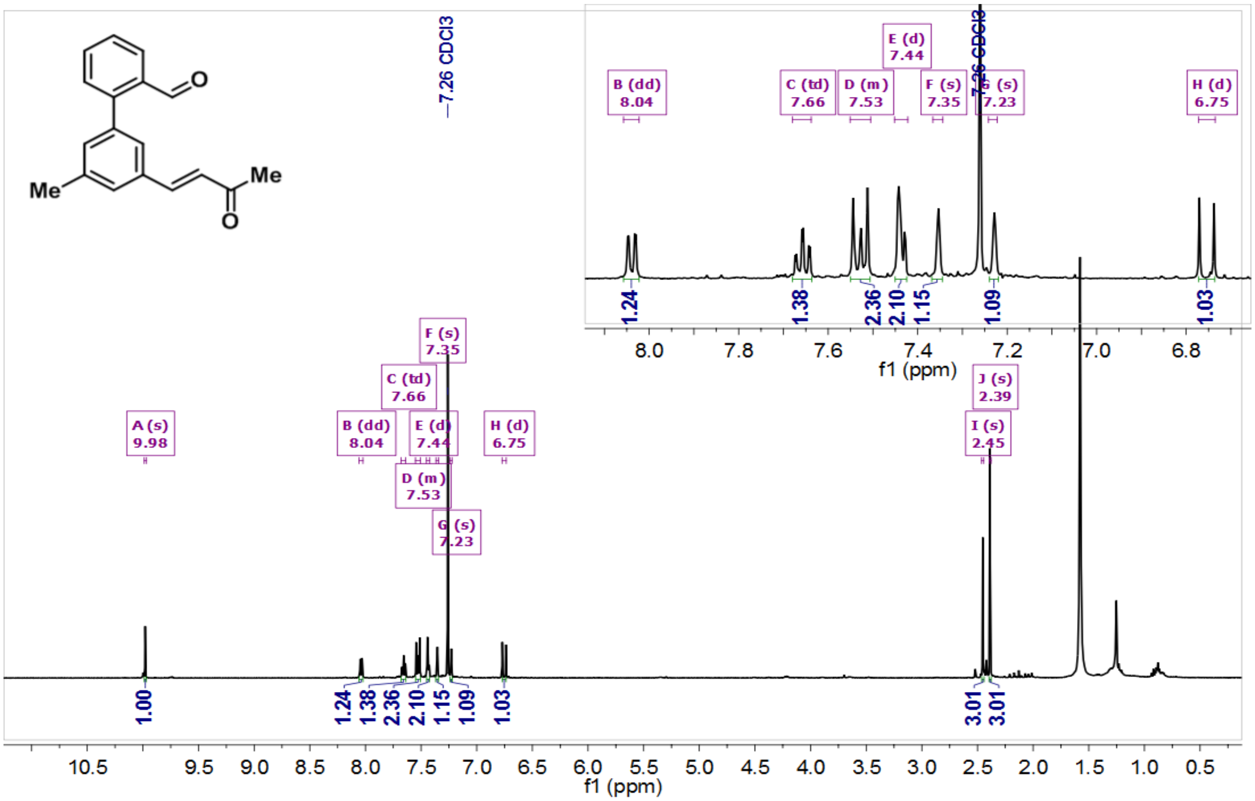

^{13}C NMR

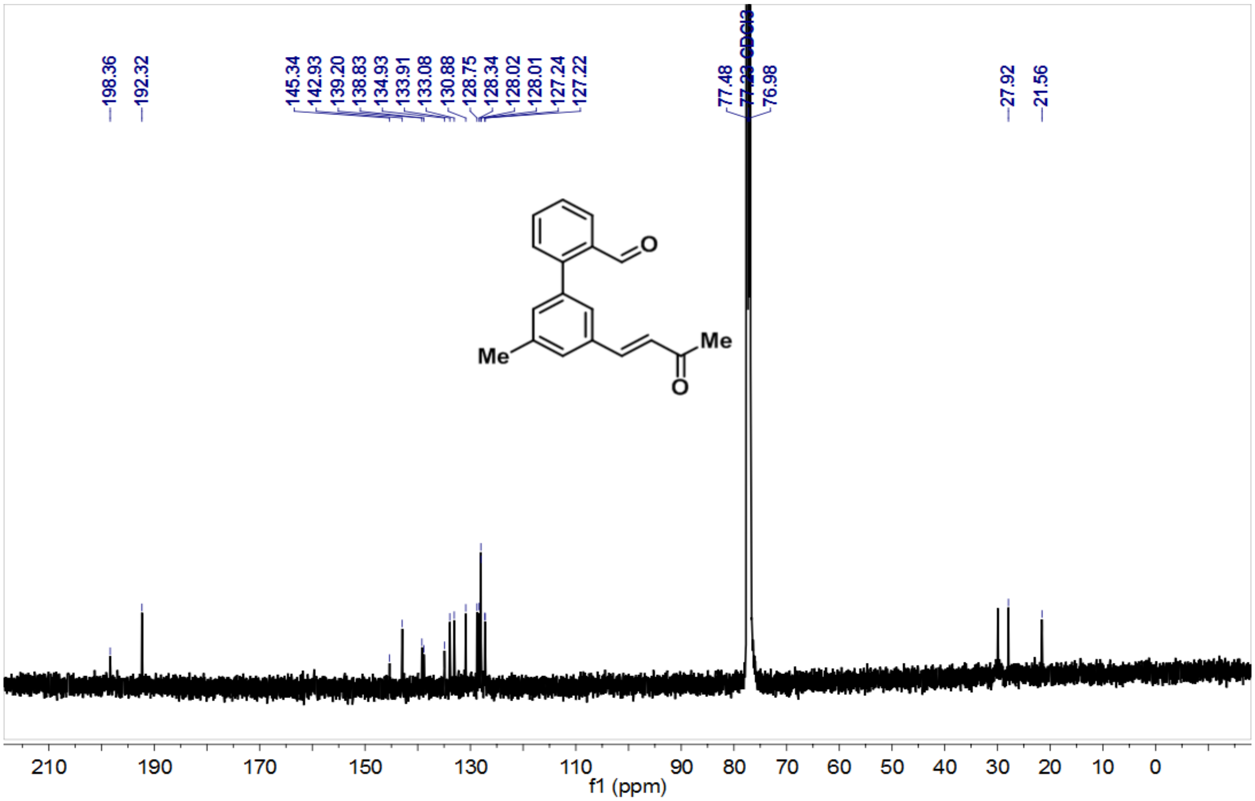

(E)-3'-methyl-5'-(3-oxopent-1-en-1-yl)-[1,1'-biphenyl]-2-carbaldehyde (**13**)

^{1}H NMR

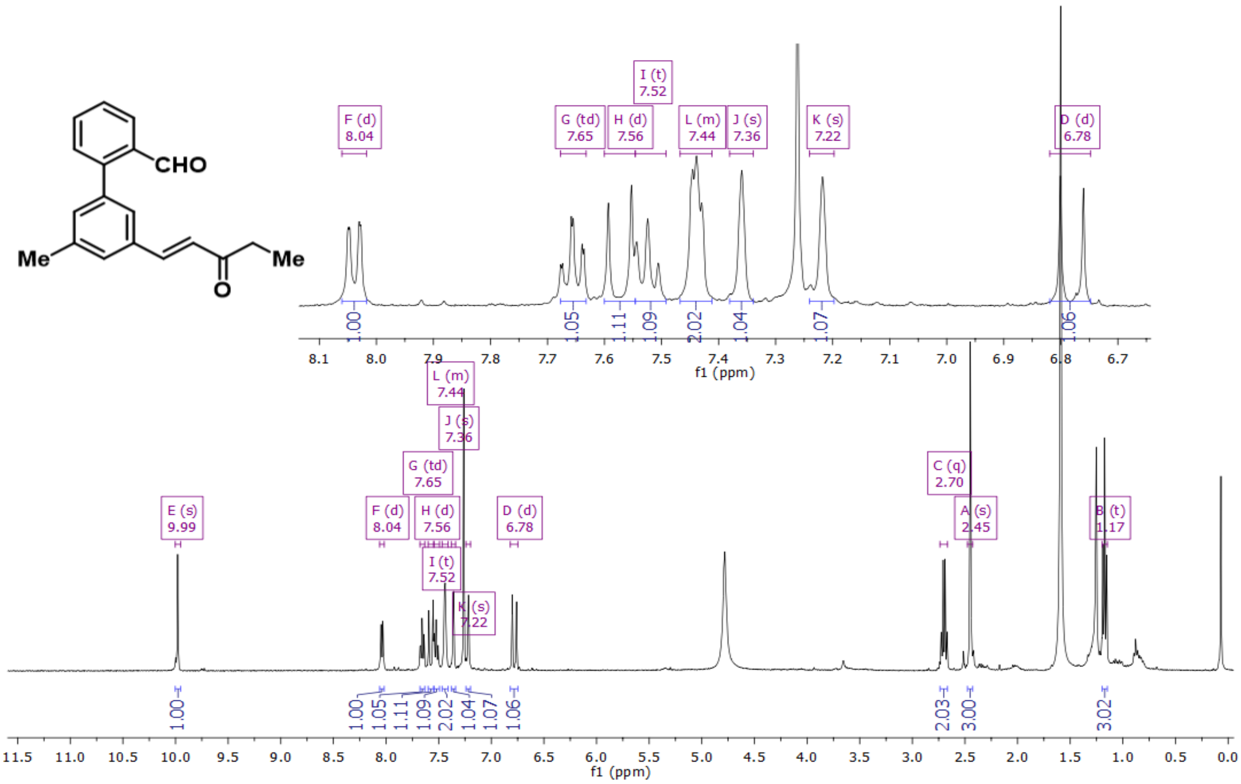

^{13}C NMR

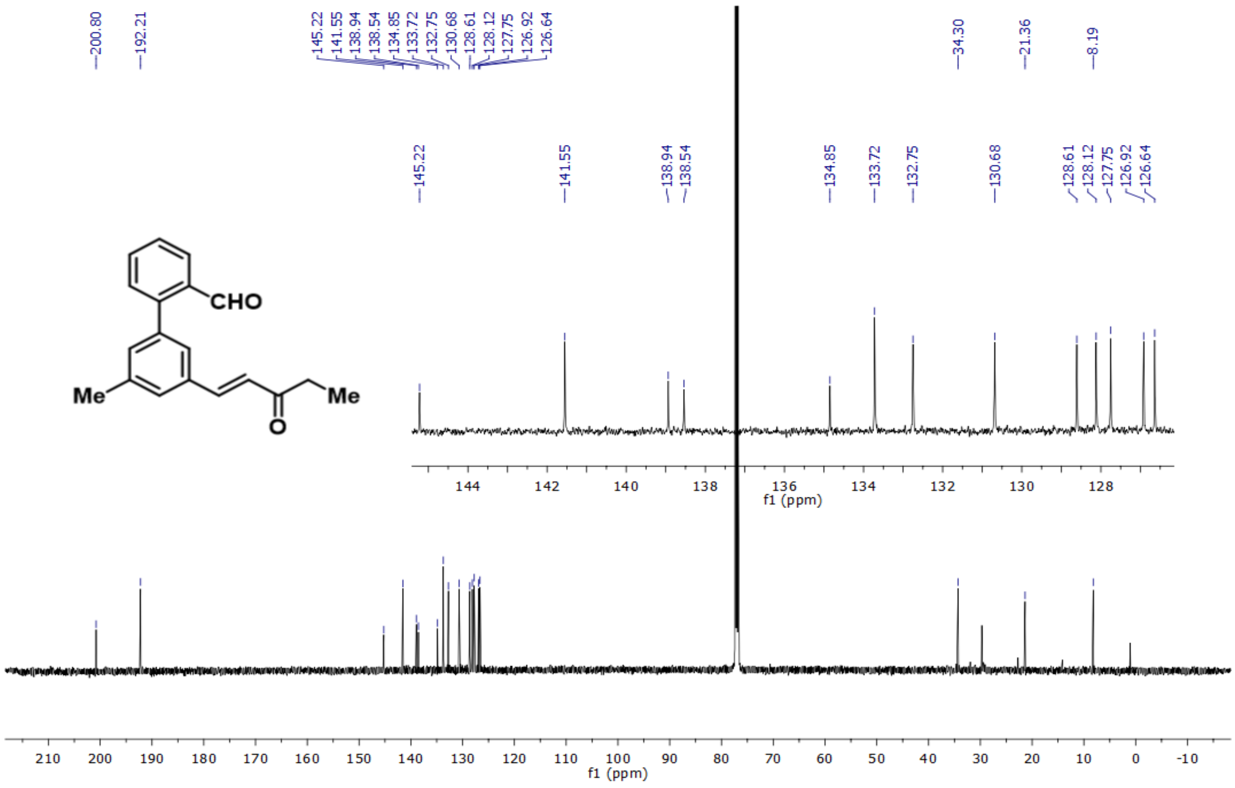

(E)-3-(2'-formyl-5-methyl-[1,1'-biphenyl]-3-yl)acrylonitrile (14)

^{1}H NMR

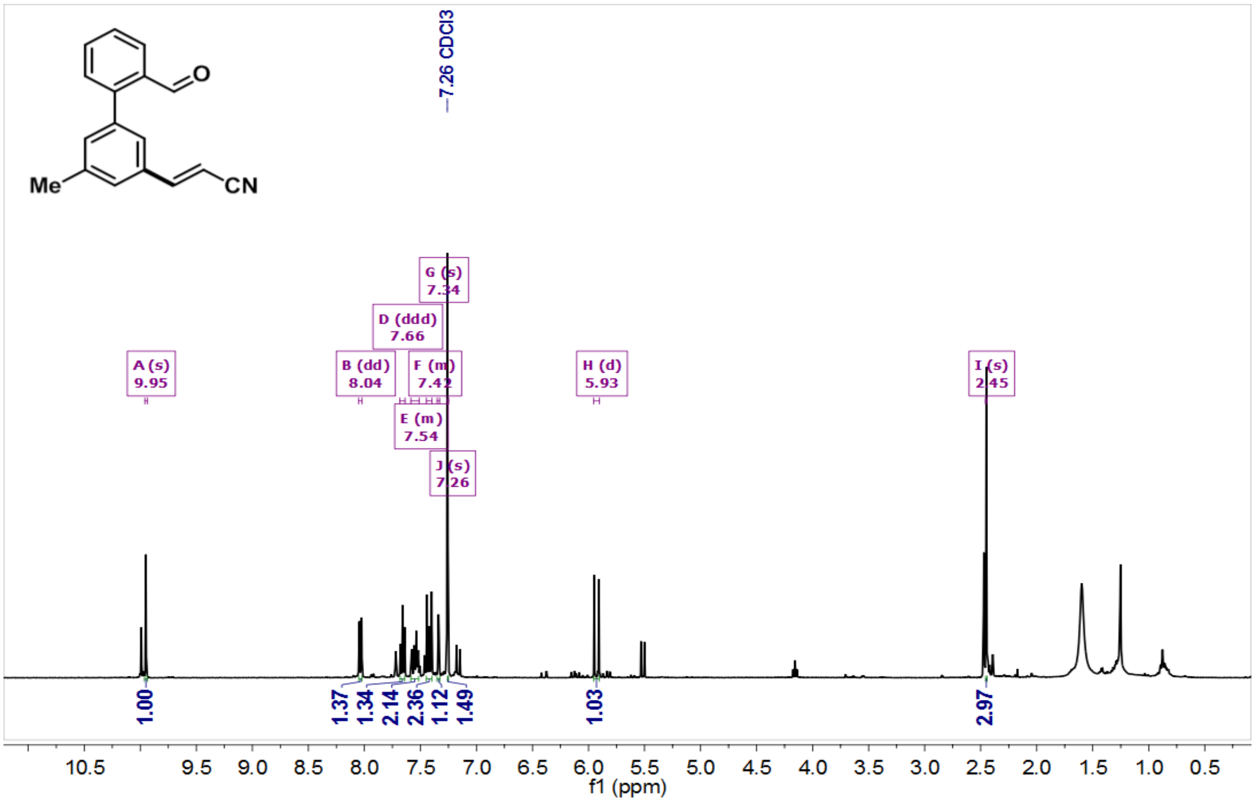

^{13}C NMR

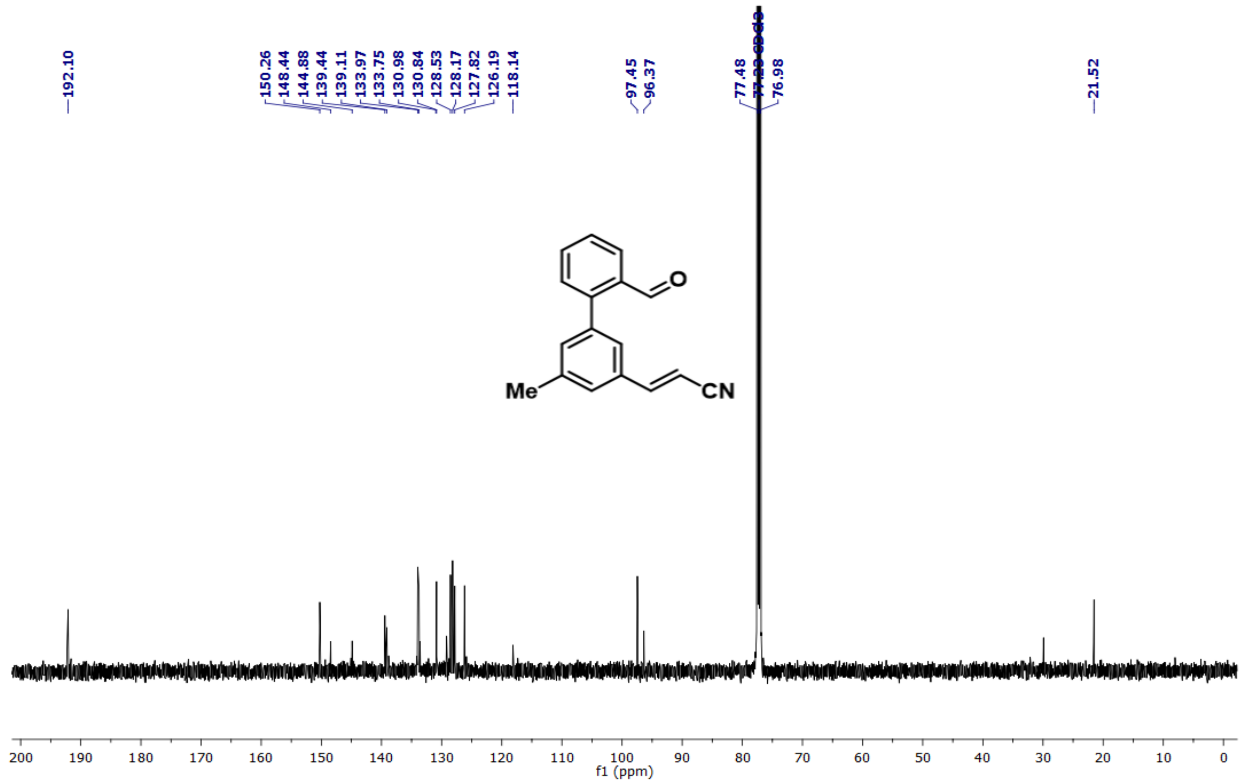

(8R,9S,10R,13S,14S,17S)-10,13-dimethyl-3-oxo-2,3,6,7,8,9,10,11,12,13,14,15,16,17-tetradecahydro-1H-cyclopenta[a]phenanthren-17-yl (E)-3-(2'-formyl-[1,1'-biphenyl]-3-yl)acrylate (15);^{1}H NMR

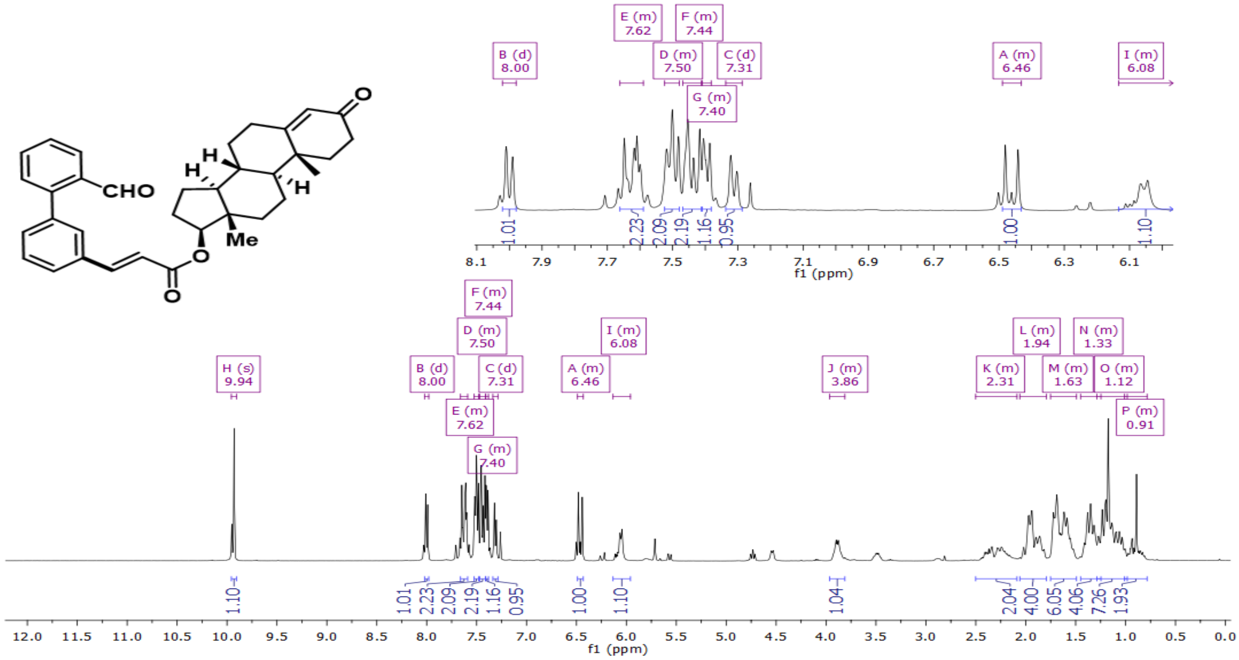

^{13}C NMR

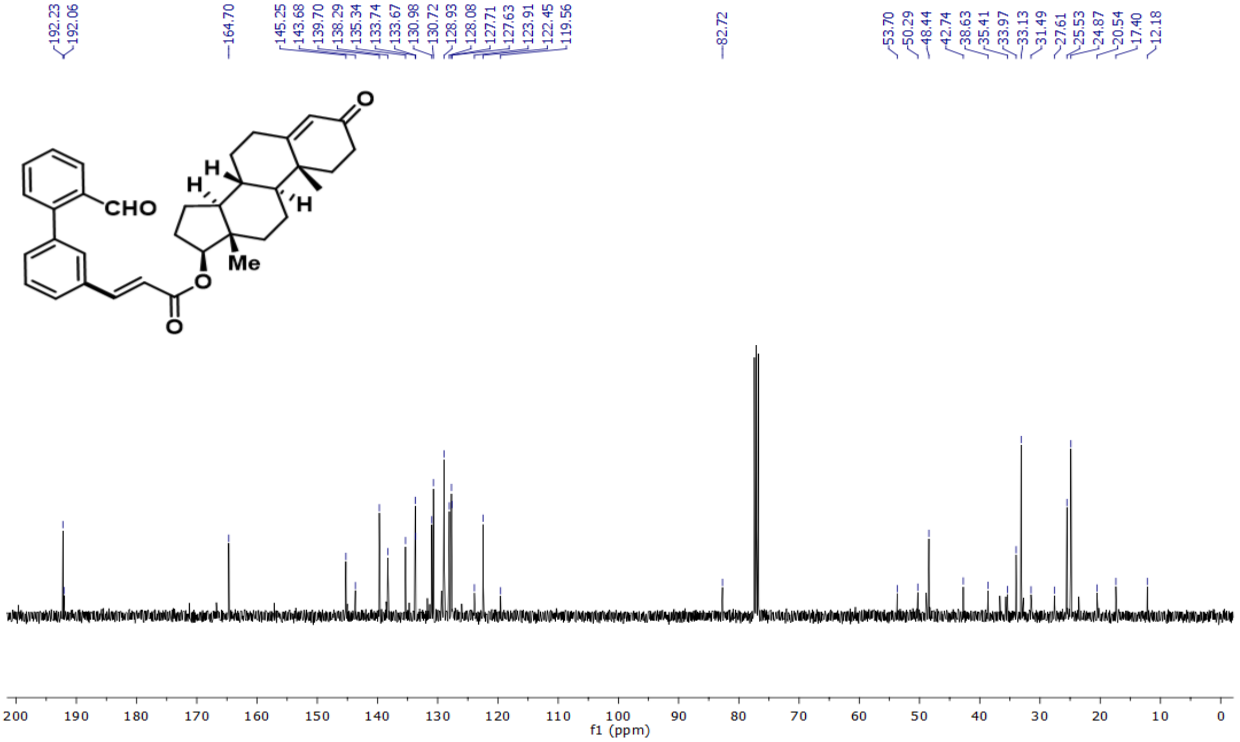

***3R,9R,10S,13S,14S,17S)-17-((2S,5S,E)-5,6-dimethylhept-3-en-2-yl)-10,13-dimethyl-2,3,4,9,10,11,12,13,14,15,16,17-dodecahydro-1H-cyclopenta[a]phenanthren-3-yl (E)-3-(2'-formyl-[1,1'-biphenyl]-3-yl)acrylate* (16); ^{1}H NMR**

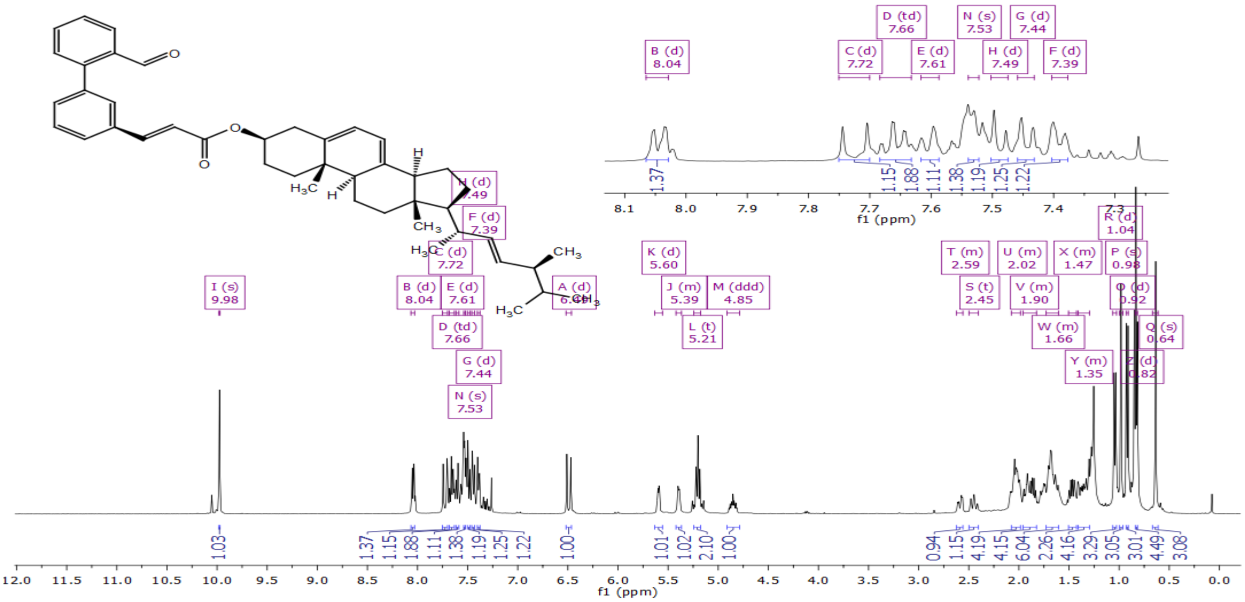

^{13}C NMR

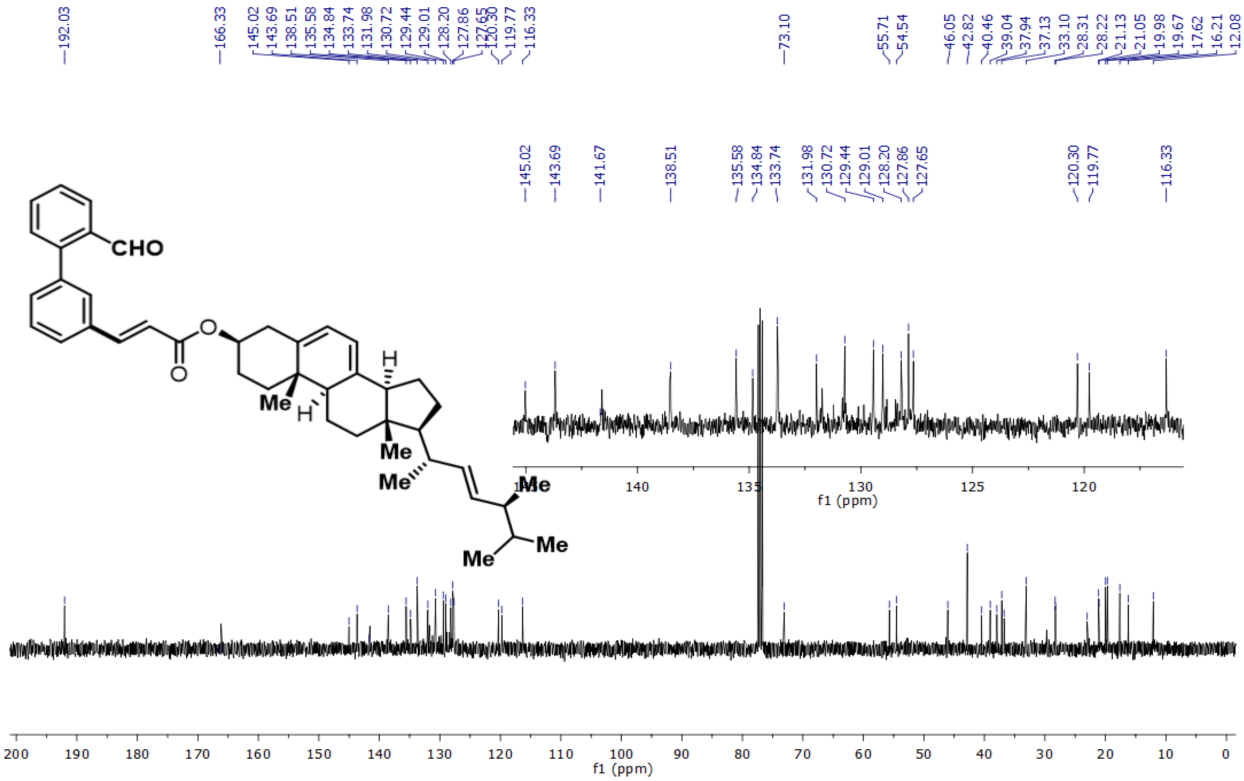

***(4R)-1,7,7-trimethylbicyclo[2.2.1]heptan-2-yl (E)-3-(2'-formyl-5',6-dimethyl-[1,1'-biphenyl]-3-yl)acrylate* (17); ^{1}H NMR**

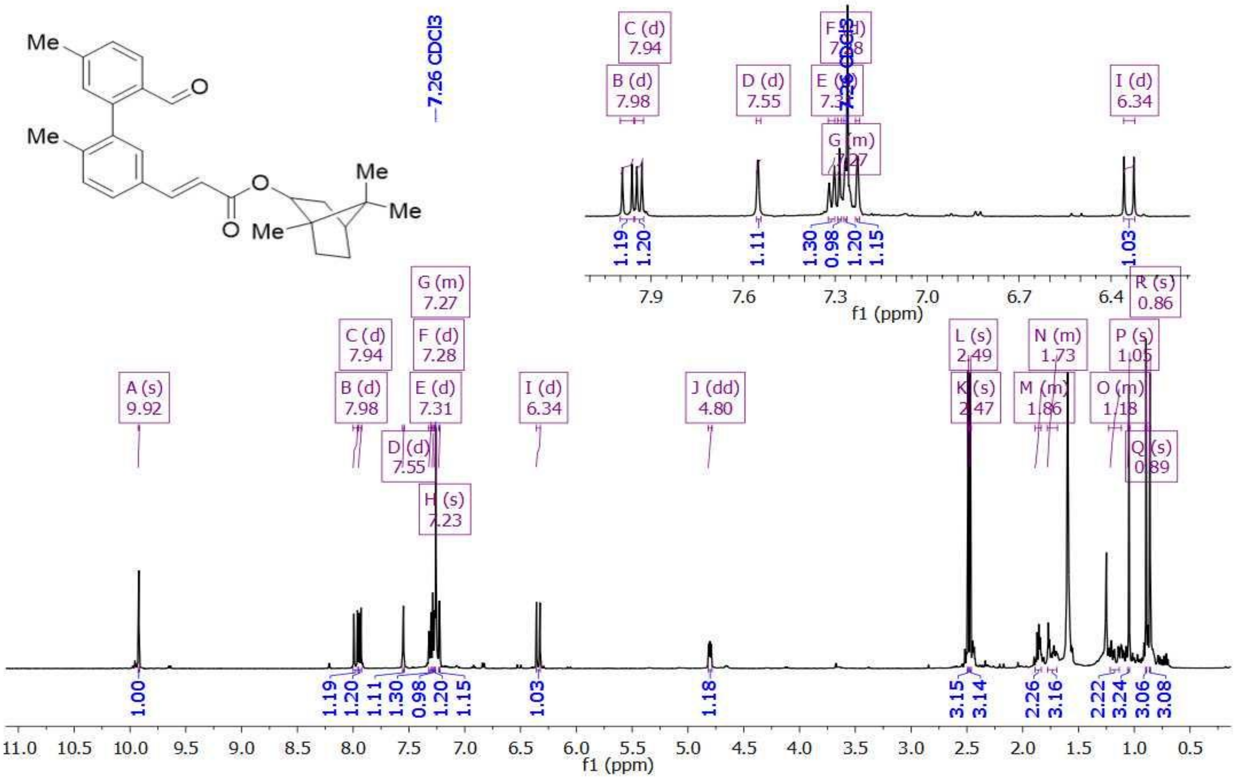

^{13}C NMR

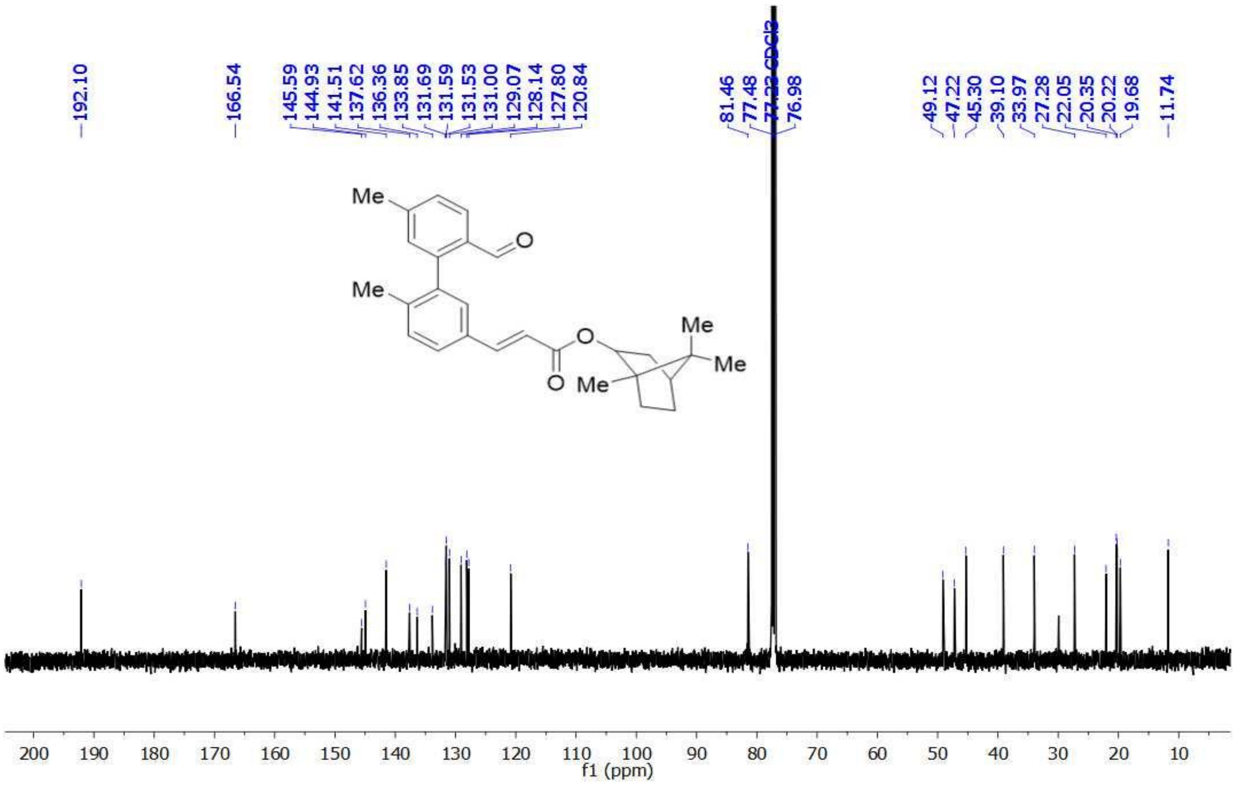

***(Z)-octadec-9-en-1-yl (E)-3-(2'-formyl-[1,1'-biphenyl]-3-yl)acrylate* (18)**

^{1}H NMR

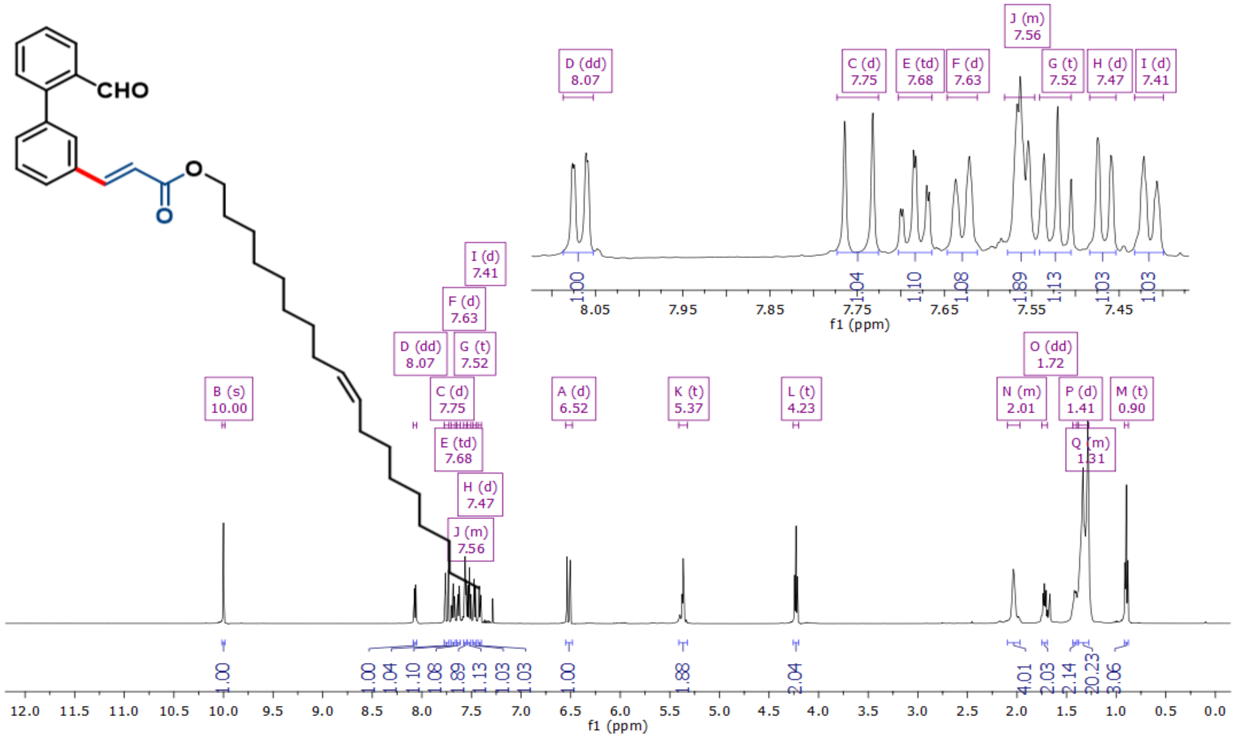

^{13}C NMR

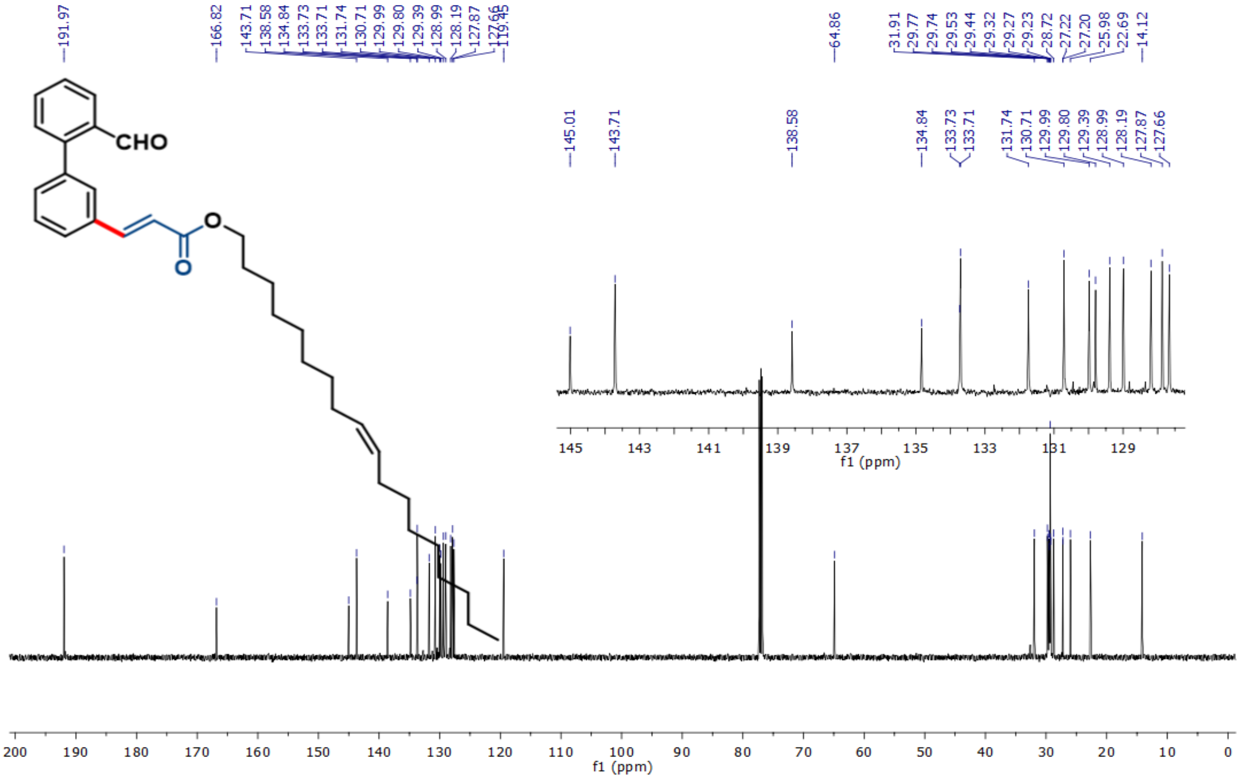

***1,1,2,2,3,3,4,4,5,5,6,6,7,7-tetradecafluoroheptyl (E)-3-(2'-formyl-5-methyl-[1,1'-biphenyl]-3-yl)acrylate* (19); ^{1}H NMR**

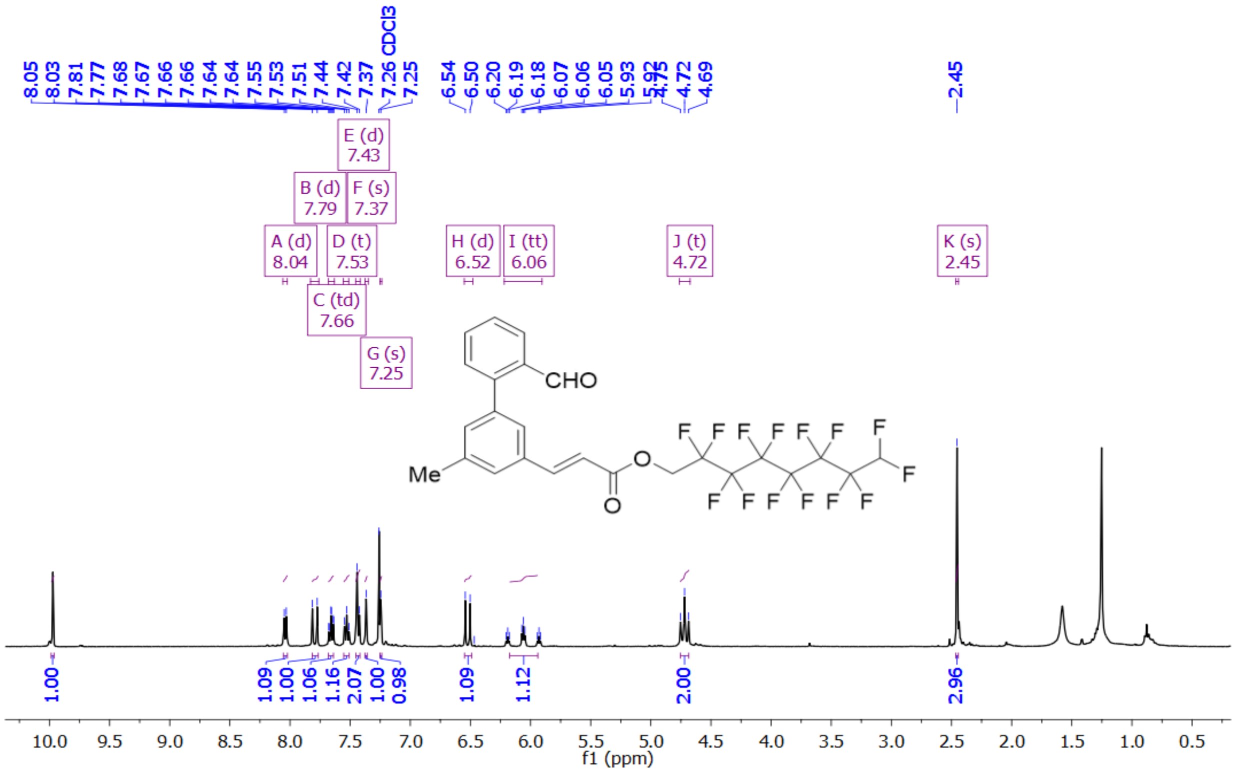

^{13}C NMR

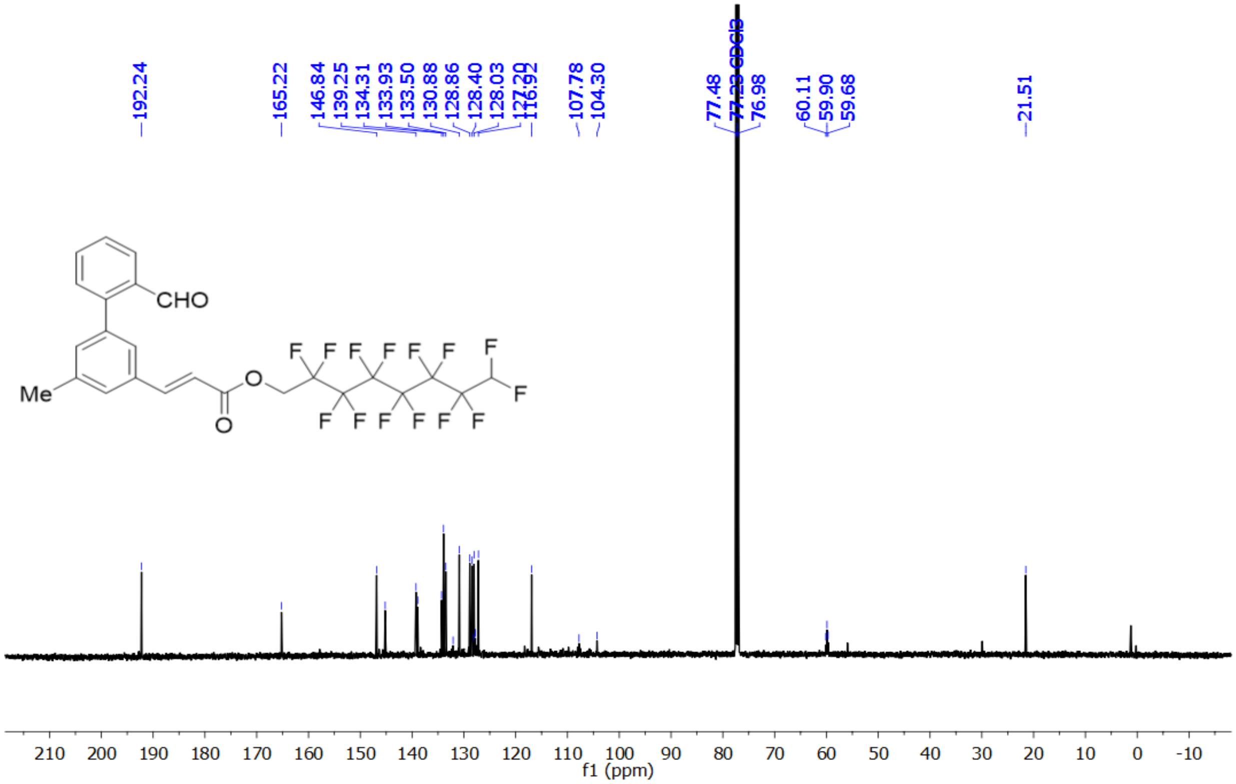

^{19}F NMR

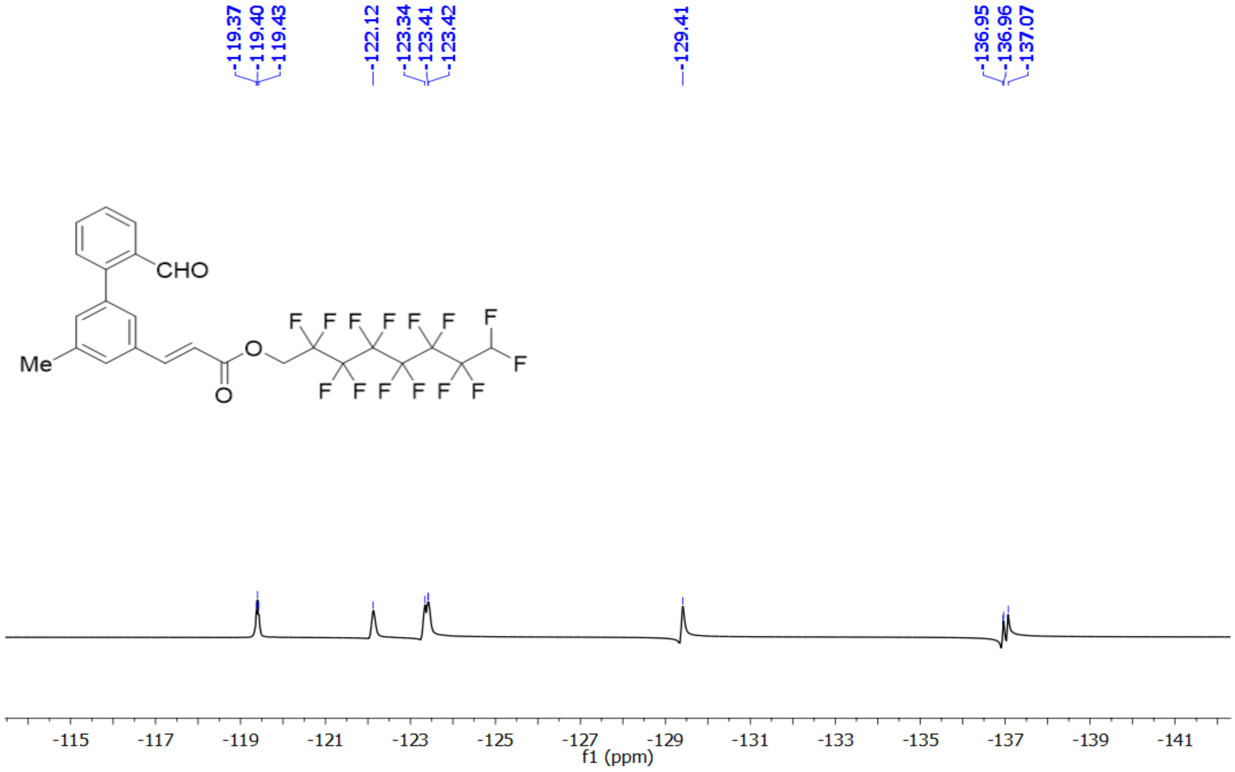
-119.37
-119.40
-119.43
-122.12
-123.34
-123.41
-123.42
-129.41
-136.95
-136.96
-137.07
CHO
Me
O
F
-115
-117
-119
-121
-123
-125
-127
-129
-131
-133
-135
-137
-139
-141
f1 (ppm)

(E)-3-(2'-formyl-5-methyl-[1,1'-biphenyl]-3-yl)allyl acetate (**20**)

^{1}H NMR

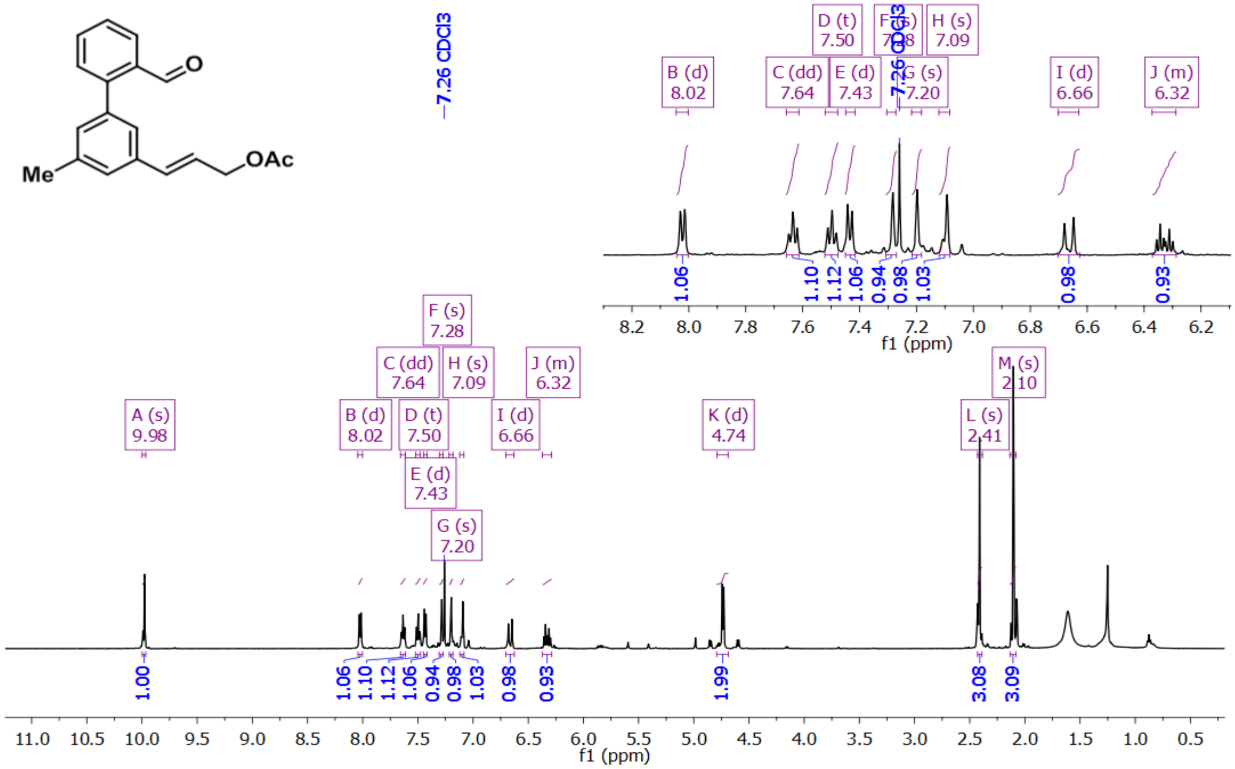

^{13}C NMR

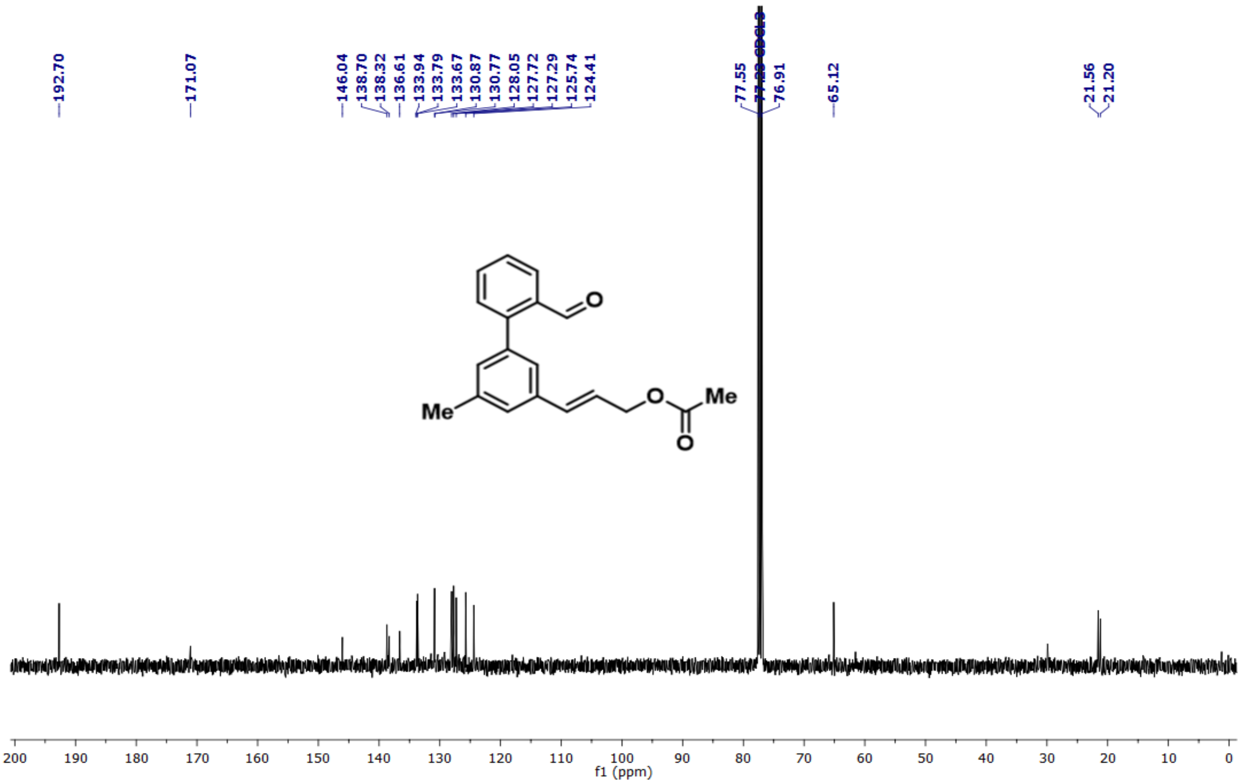

Methyl (E)-3-(2'-formyl-[1,1'-biphenyl]-3-yl)acrylate (**21**)

^{1}H NMR

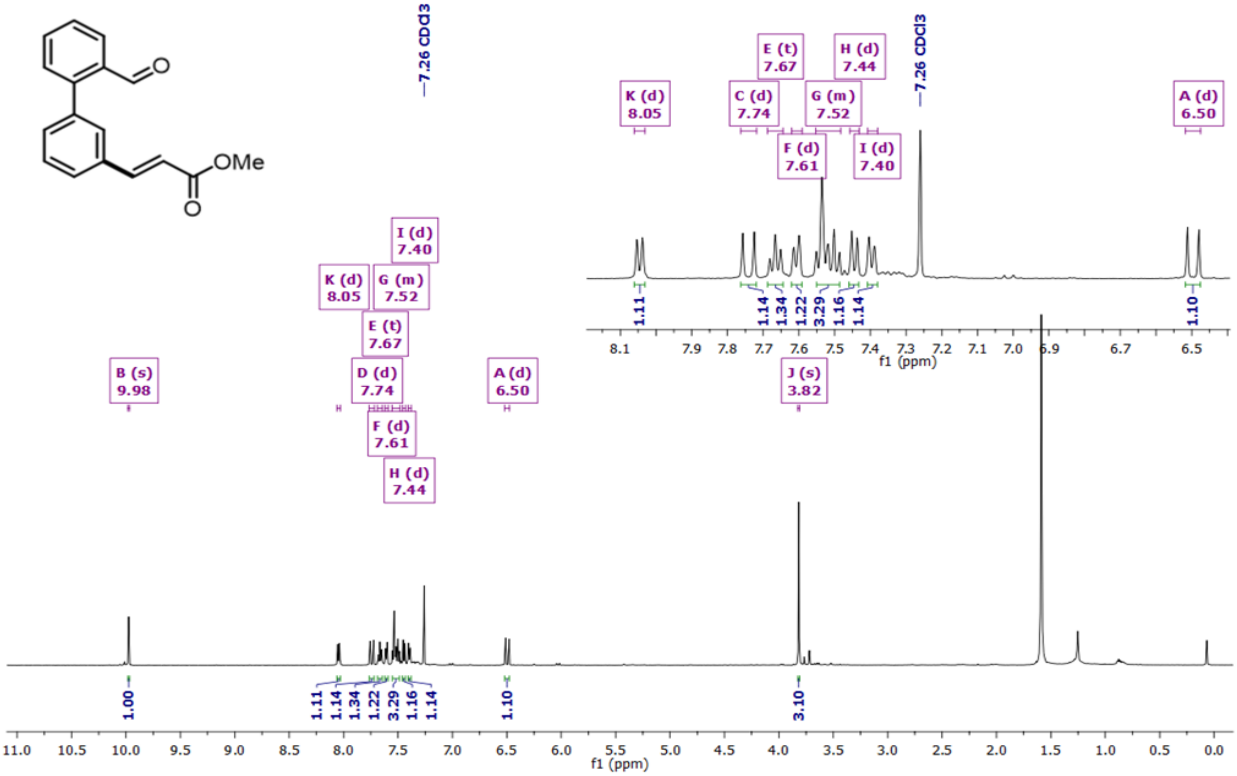

^{13}C NMR

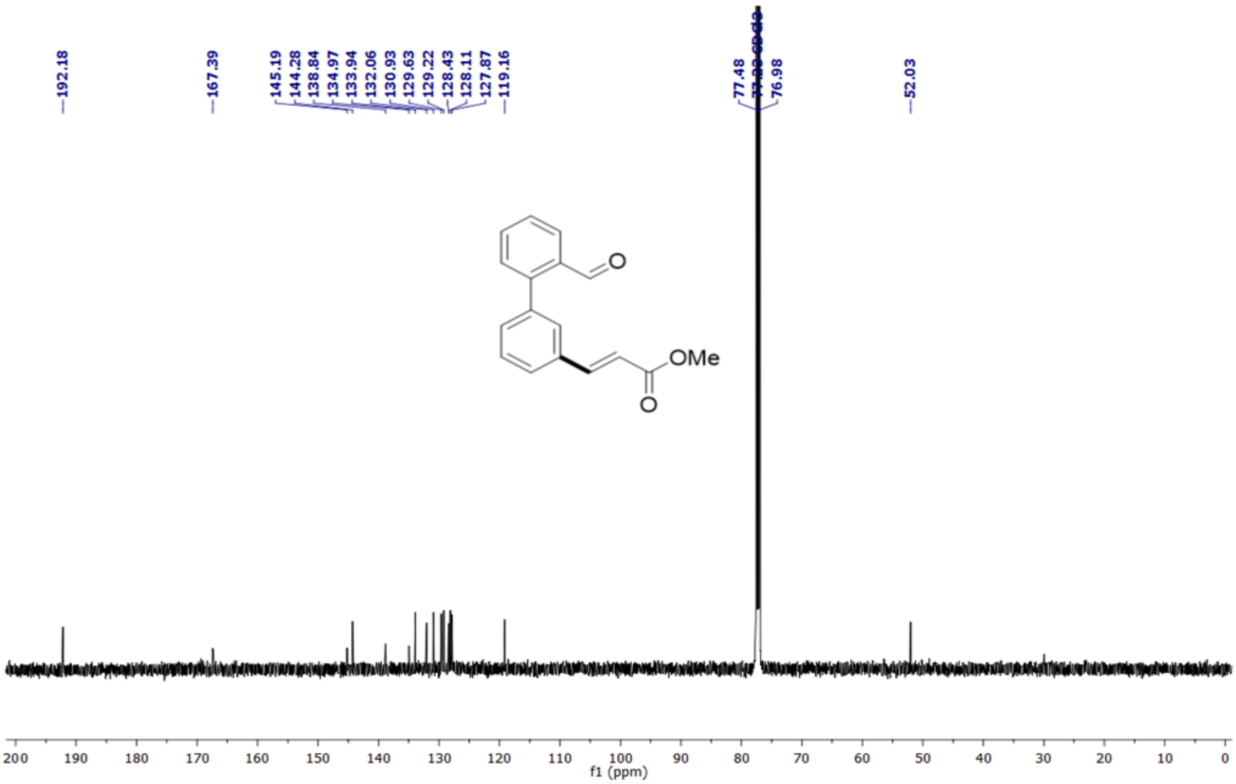

Butyl (E)-3-(2'-formyl-[1,1'-biphenyl]-3-yl)acrylate (22)

^{1}H NMR

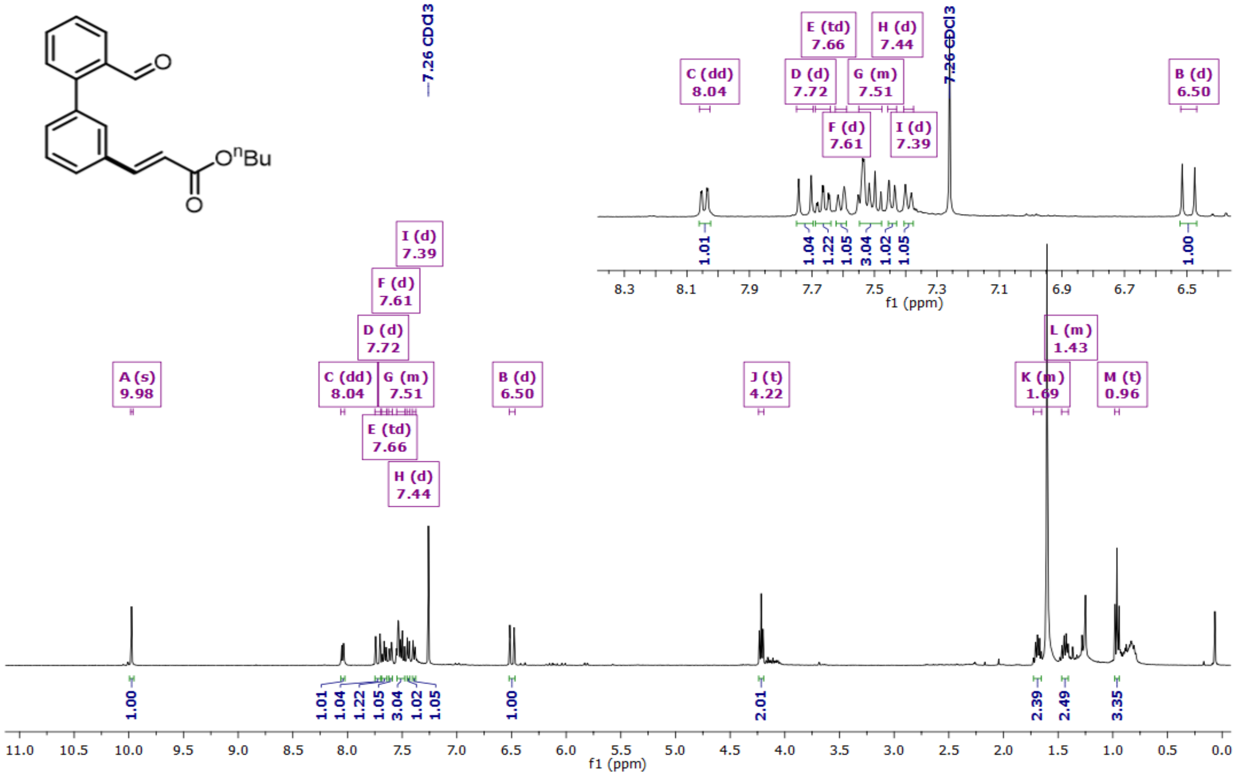

^{13}C NMR

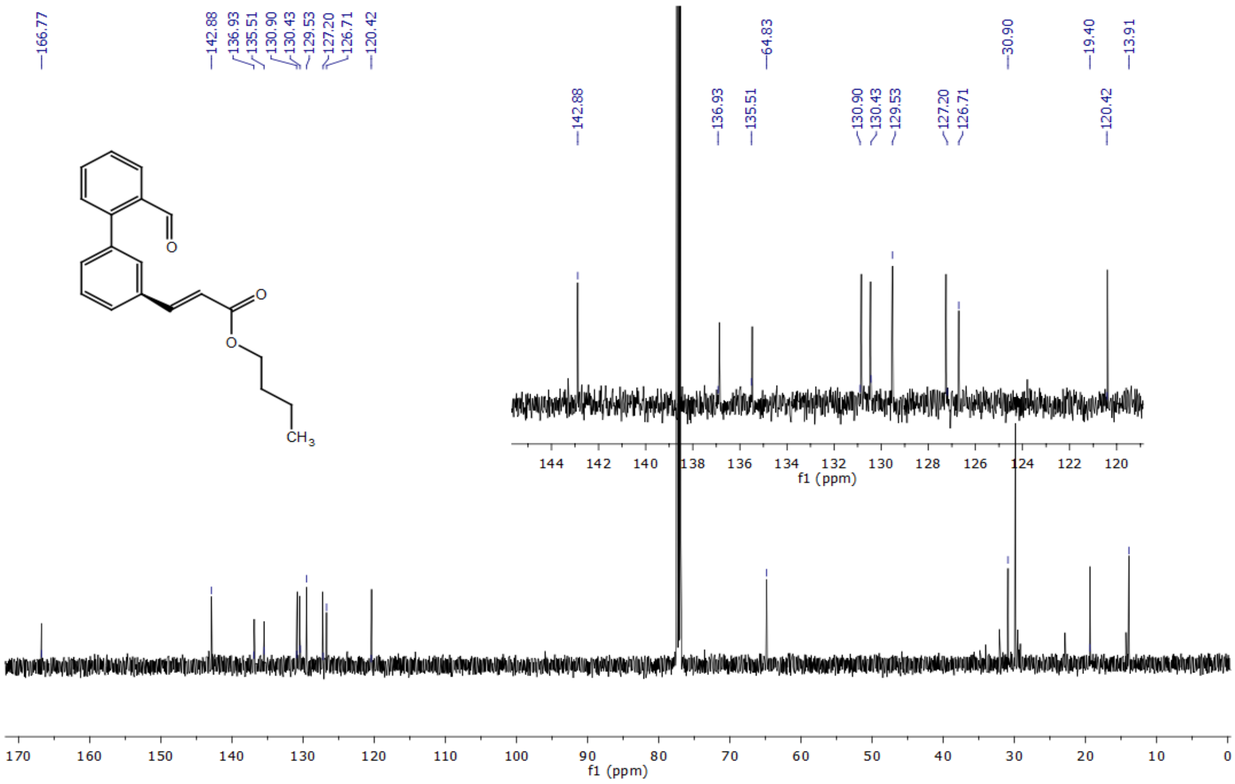

Butyl (E)-3-(2'-formyl-[1,1'-biphenyl]-3-yl)acrylate (23)

^{1}H NMR

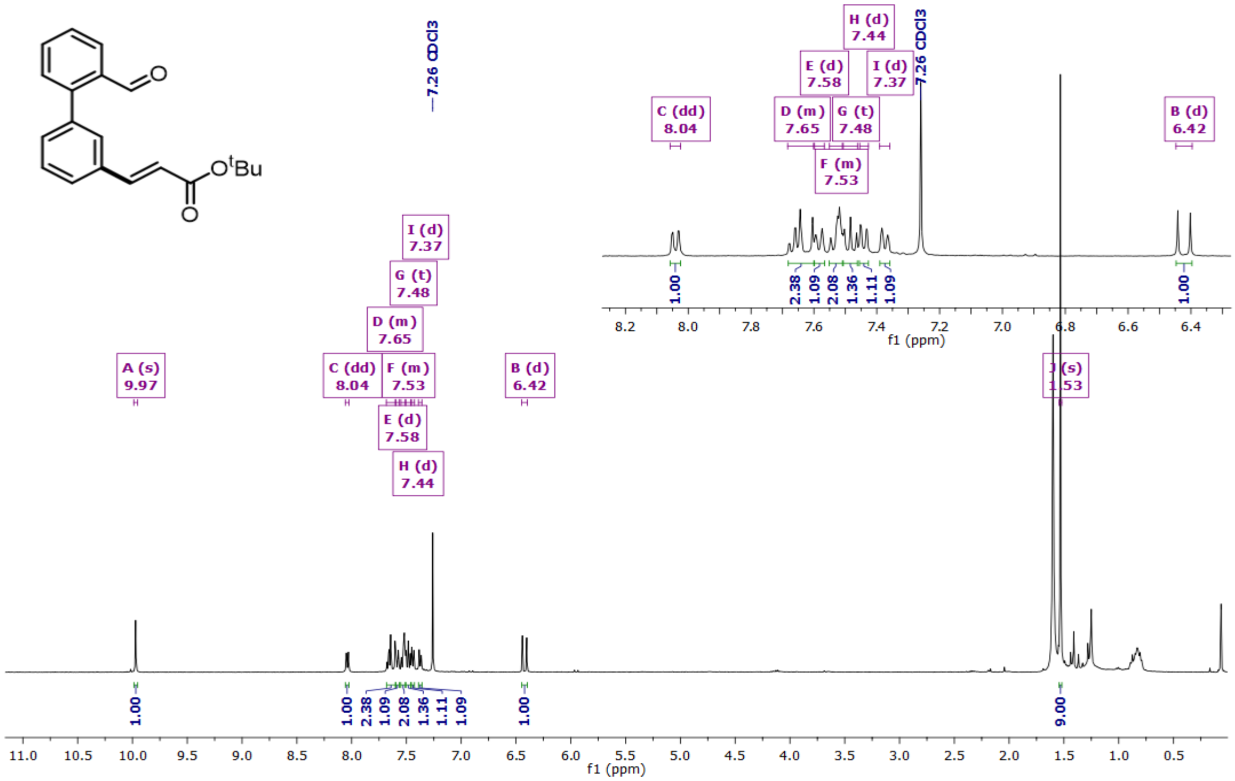

^{13}C NMR

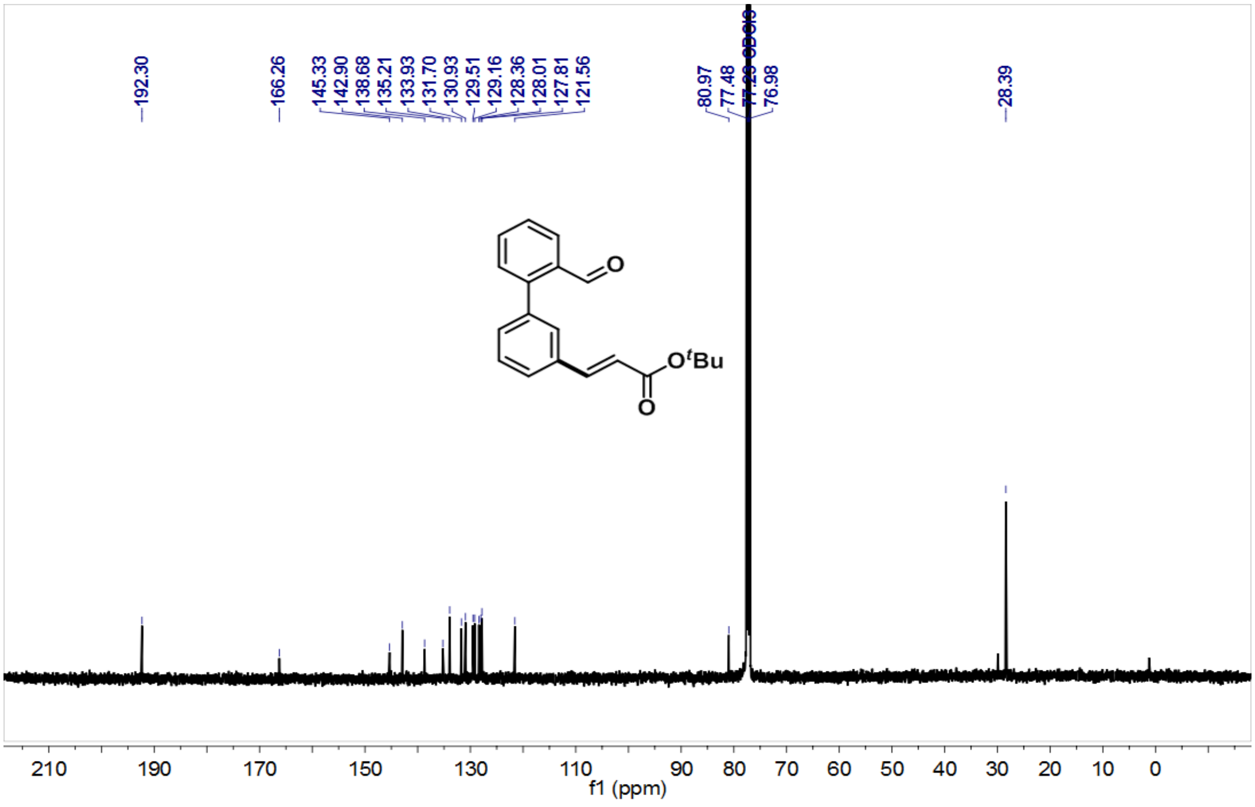

***2,2,3,3,4,4,5,5,6,6,7,7,8,8-tetradecafluorooctyl (E)-3-(2'-formyl-[1,1'-biphenyl]-3-yl)acrylate* (24); ^{1}H NMR**

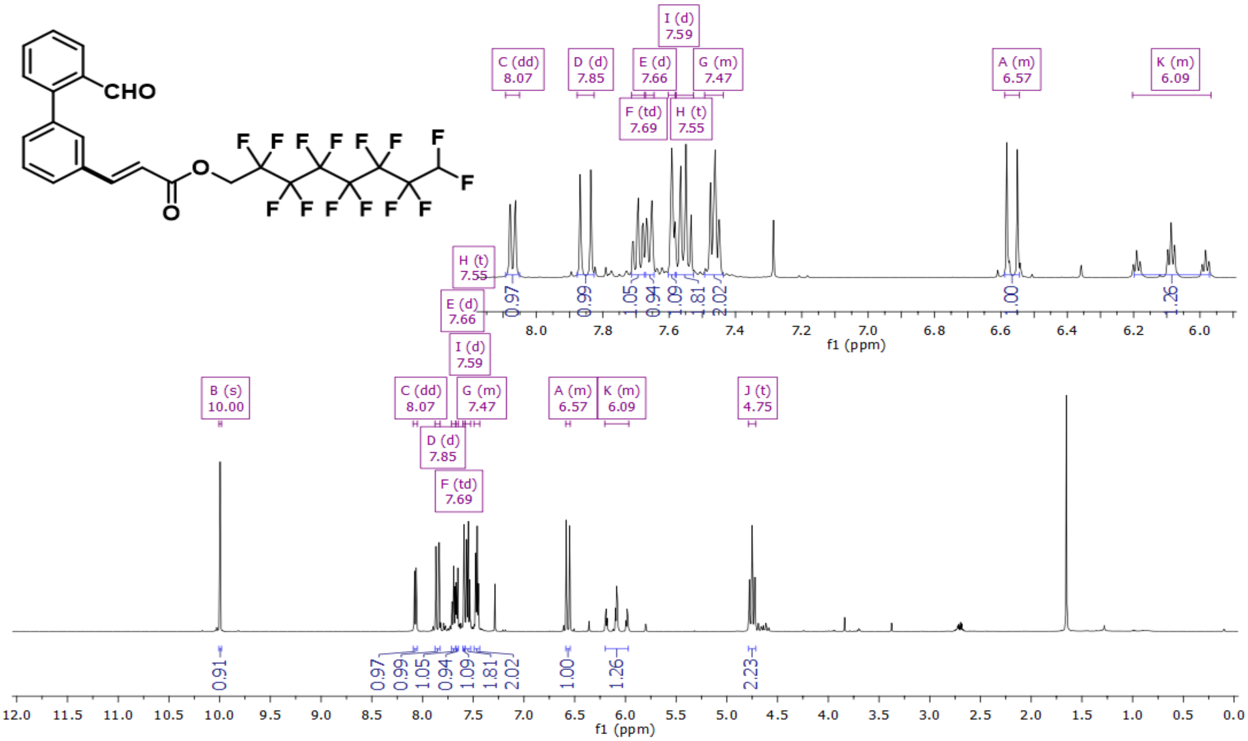

^{13}C NMR

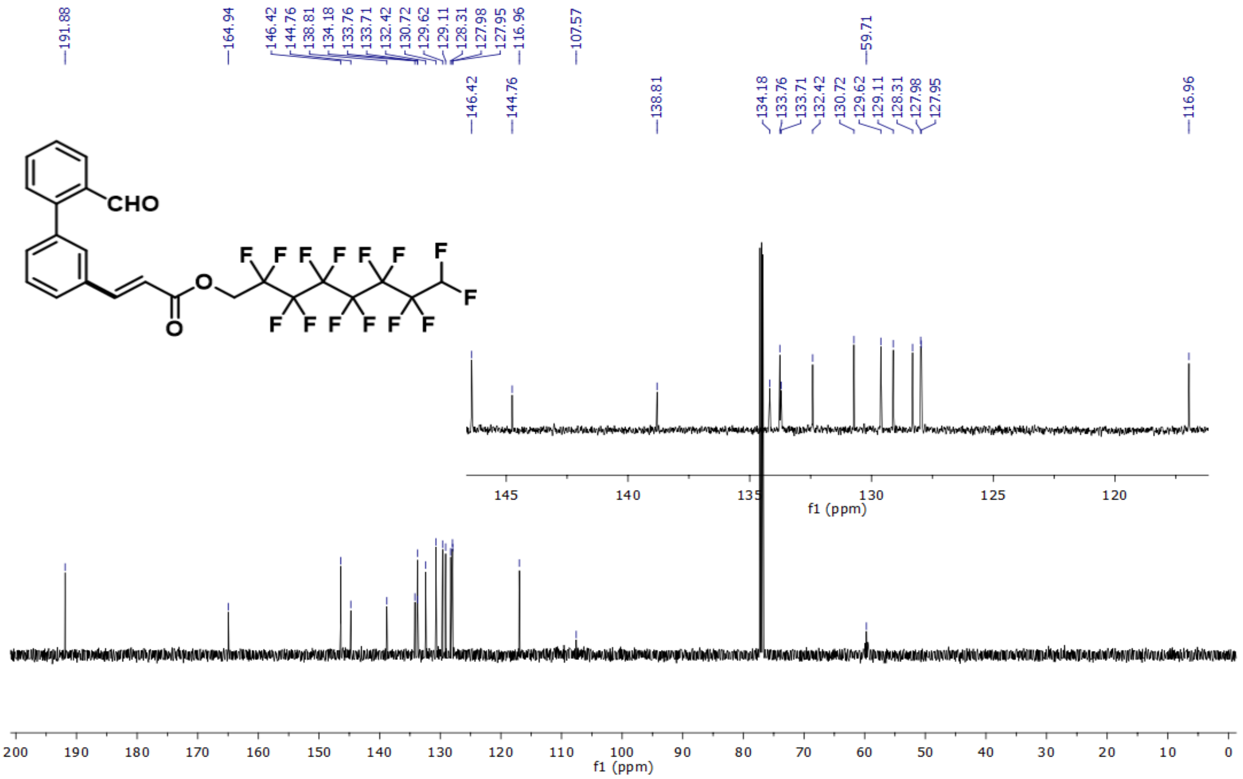

^{19}F NMR

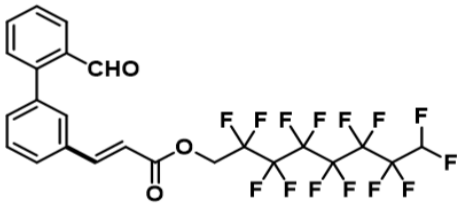
CHO
F
O

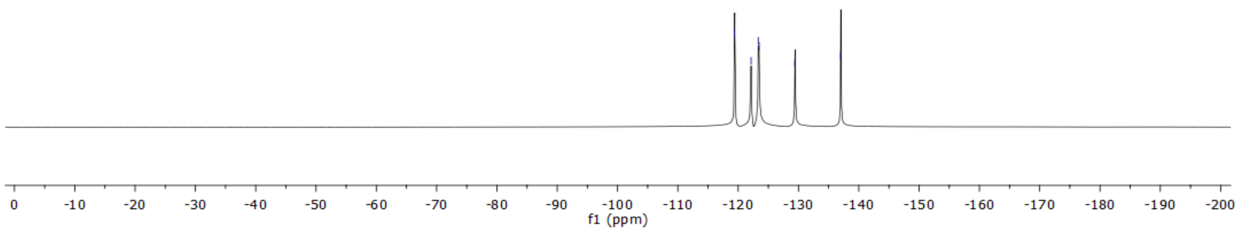
-119.39
-122.15
-123.33
-123.47
-129.39
-137.00
0
-10
-20
-30
-40
-50
-60
-70
-80
-90
-100
-110
-120
-130
-140
-150
-160
-170
-180
-190
-200
f1 (ppm)

***(E)-3'-(3-oxobut-1-en-1-yl)-[1,1'-biphenyl]-2-carbaldehyde* (25)**

^{1}H NMR

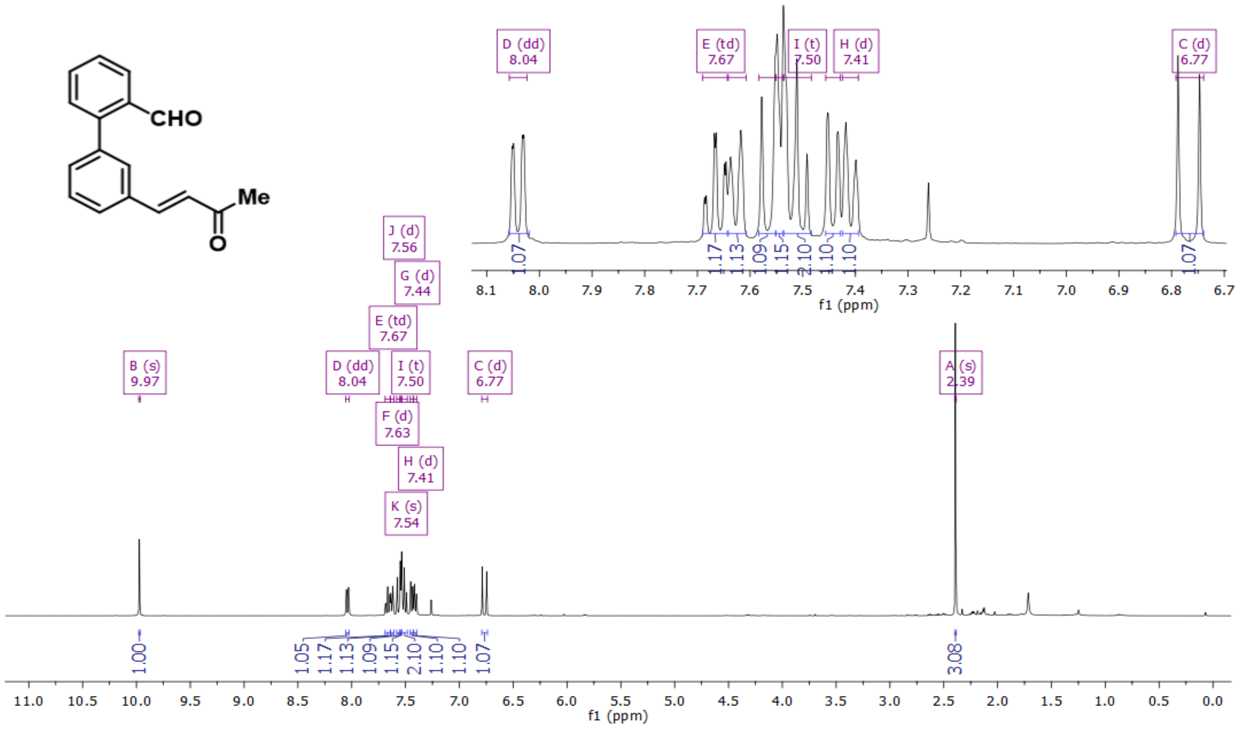

^{13}C NMR

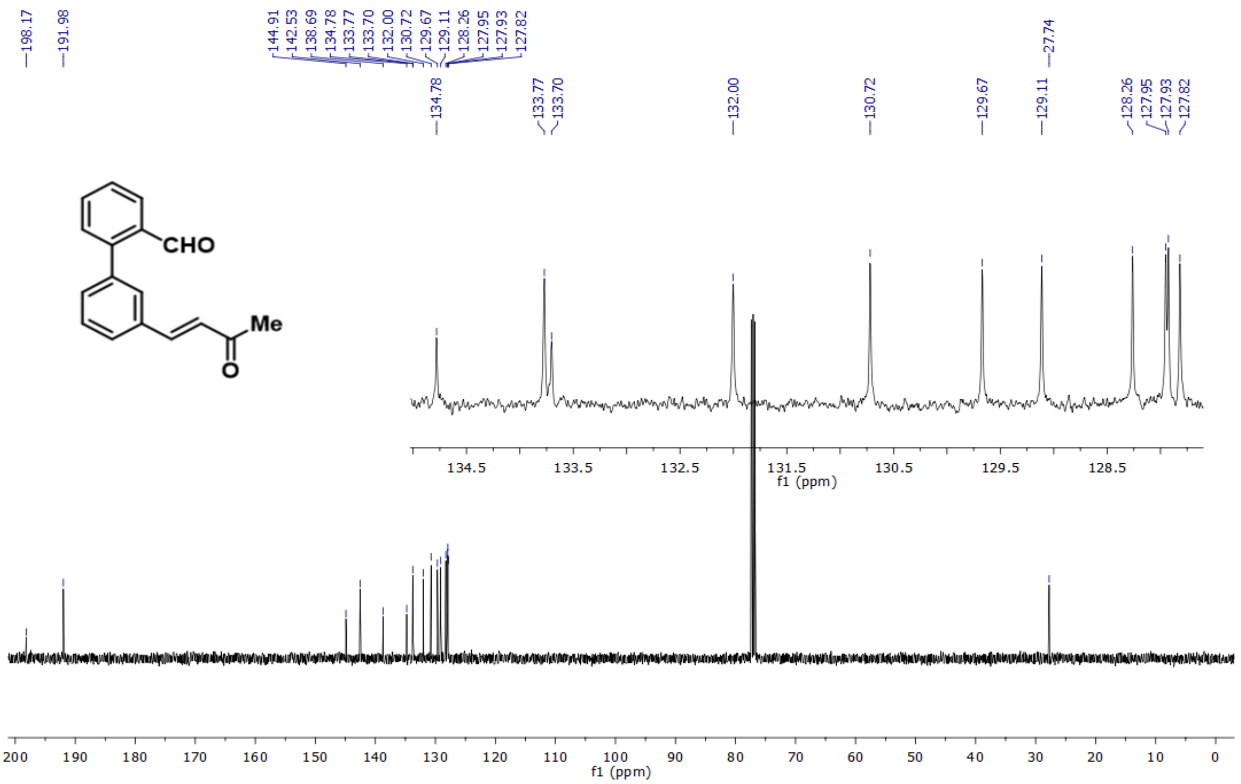

***(E)-4'-chloro-3'-(3-oxopentadec-1-en-1-yl)-[1,1'-biphenyl]-2-carbaldehyde* (26)**

^{1}H NMR

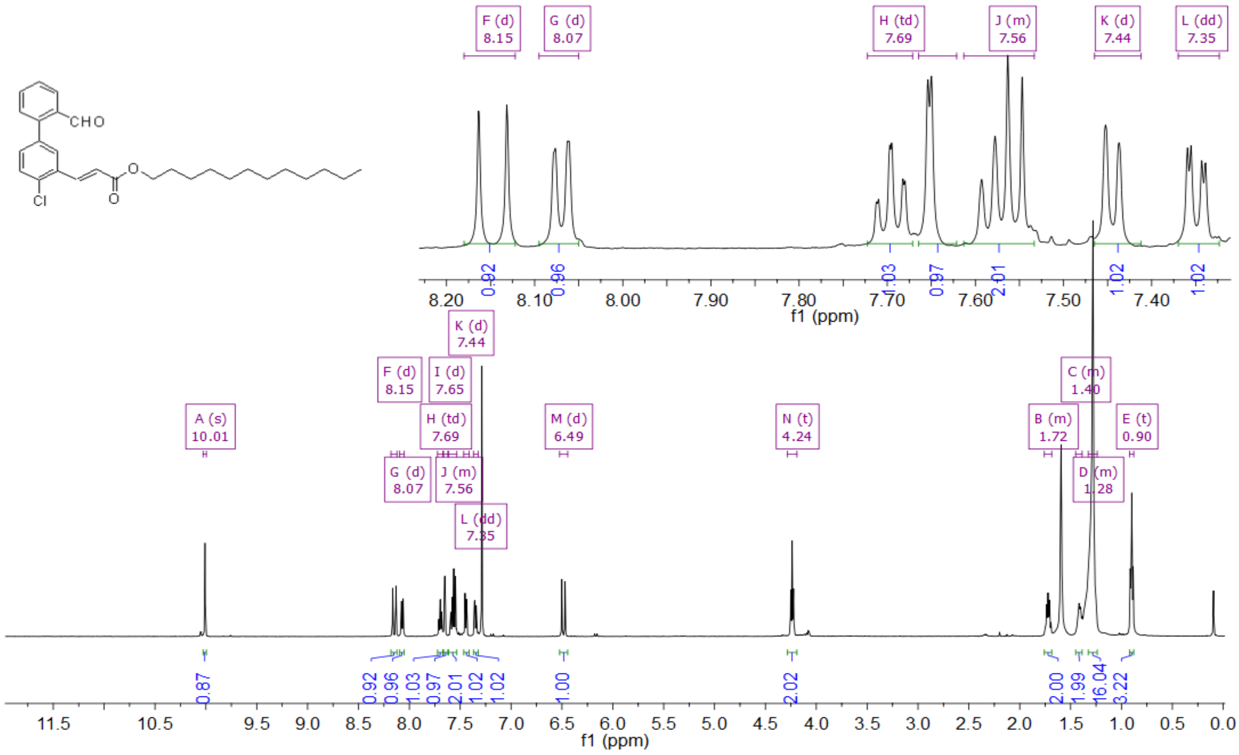

^{13}C NMR

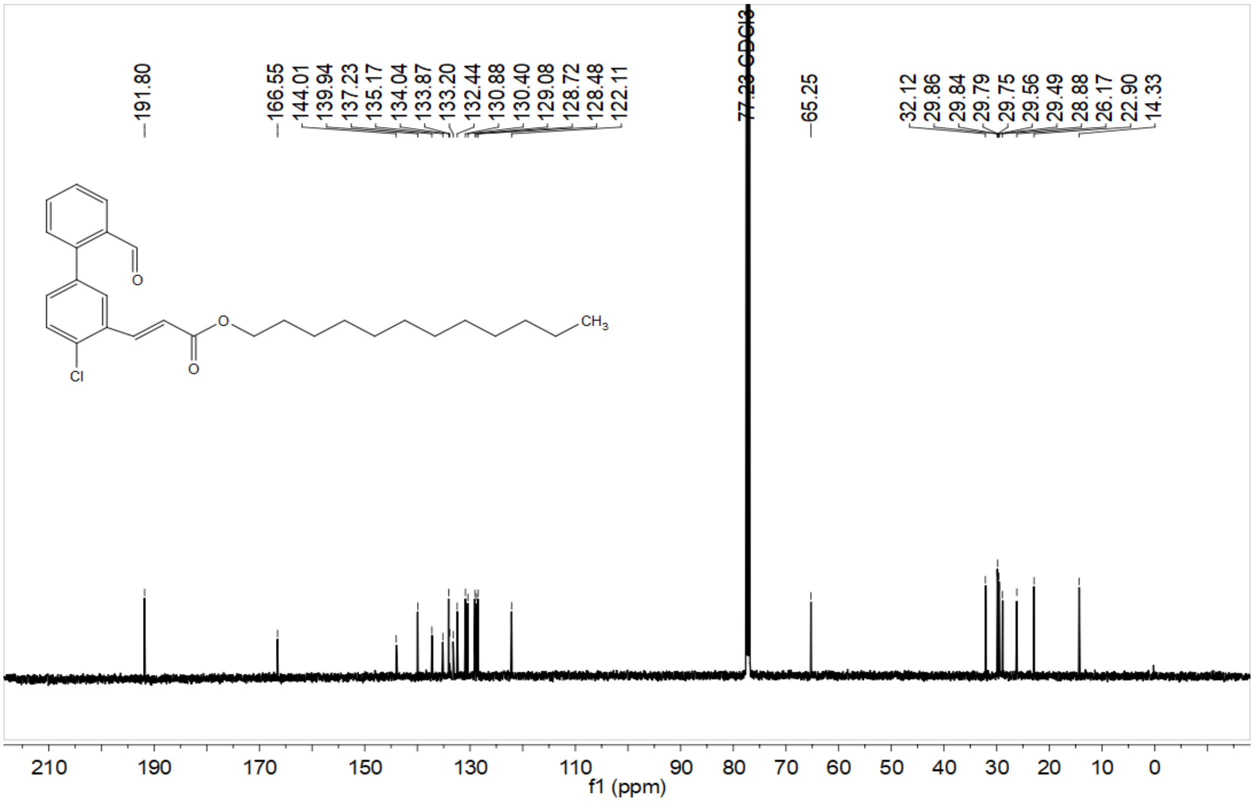

***(E)-2',6'-dimethyl-3'-(3-oxobut-1-en-1-yl)-[1,1'-biphenyl]-2-carbaldehyde* (27)**

^{1}H NMR

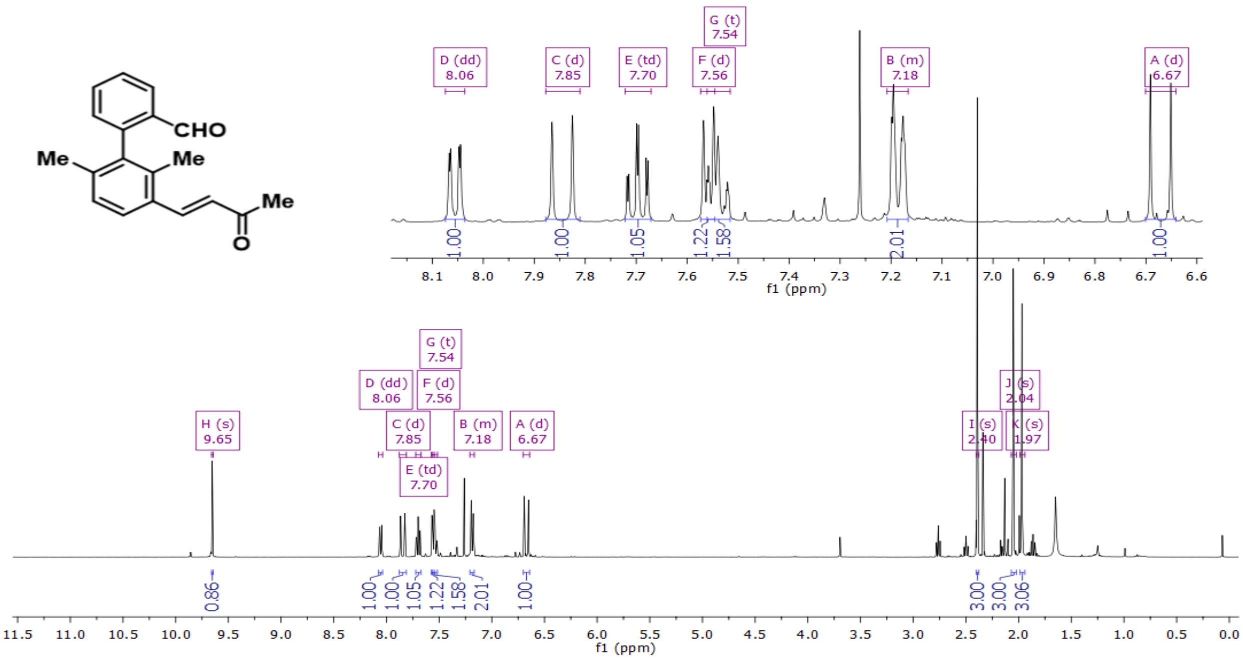

^{13}C NMR

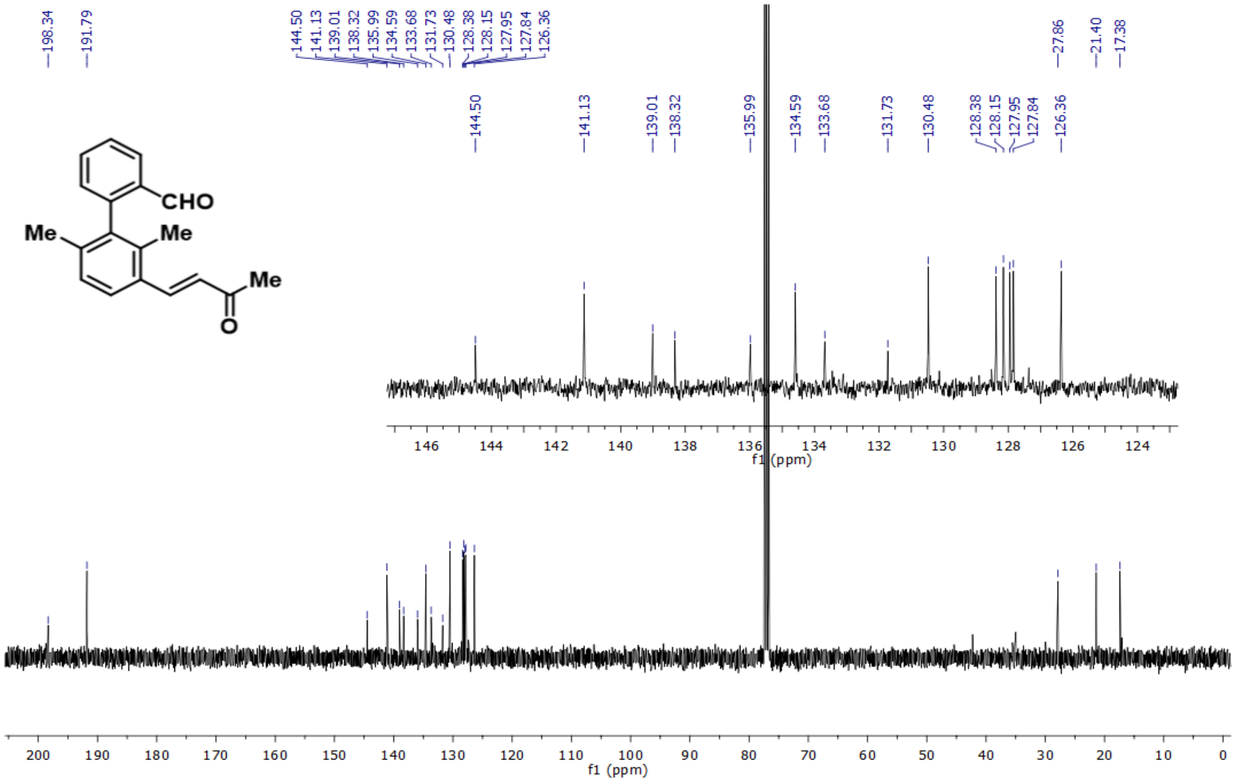

***(E)-2',6'-dimethyl-3'-(3-oxopent-1-en-1-yl)-[1,1'-biphenyl]-2-carbaldehyde* (28)**

^{1}H NMR

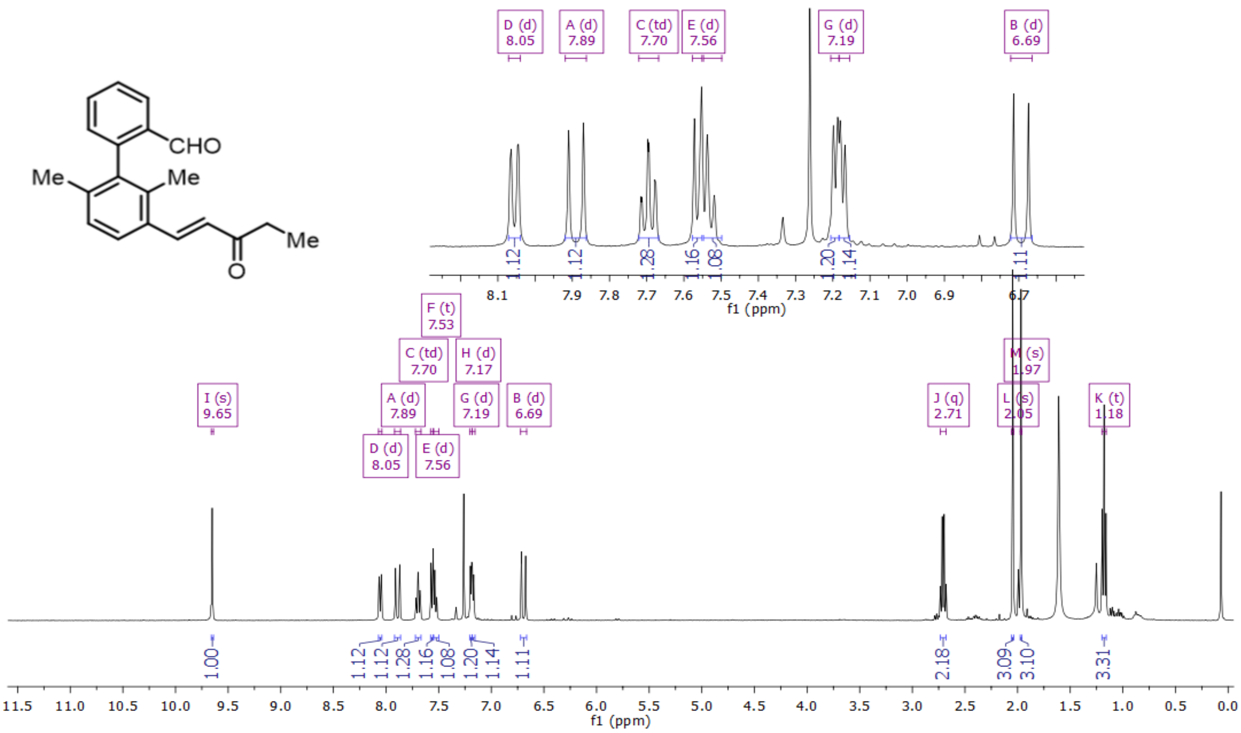

^{13}C NMR

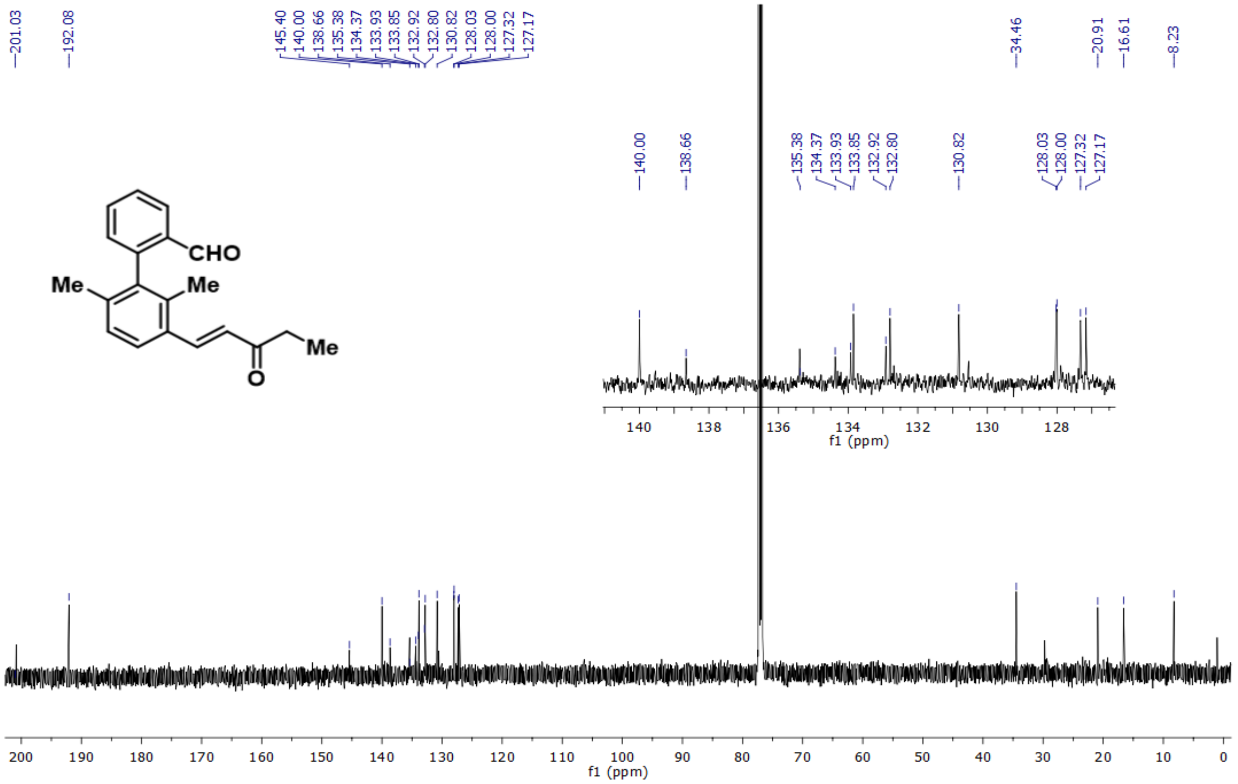

***(E)-2',5'-dimethyl-3'-(3-oxopent-1-en-1-yl)-[1,1'-biphenyl]-2-carbaldehyde* (29)**

^{1}H NMR

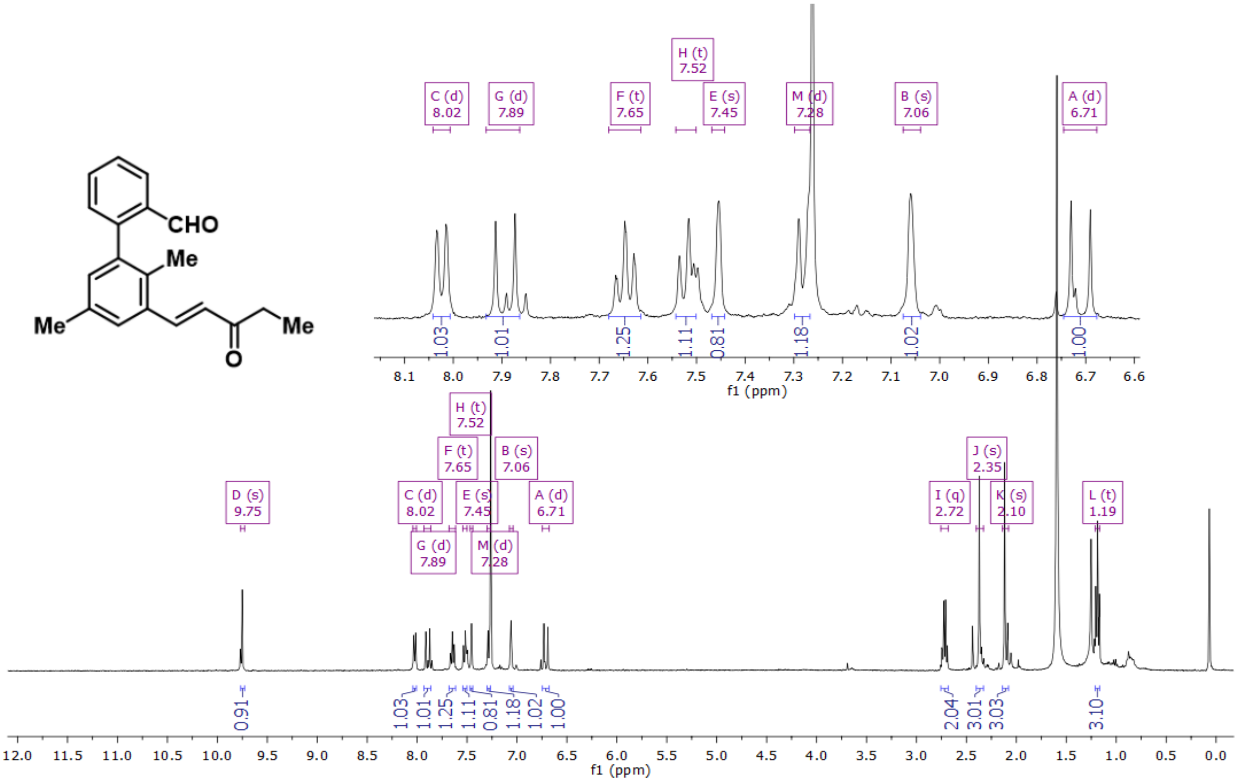

^{13}C NMR

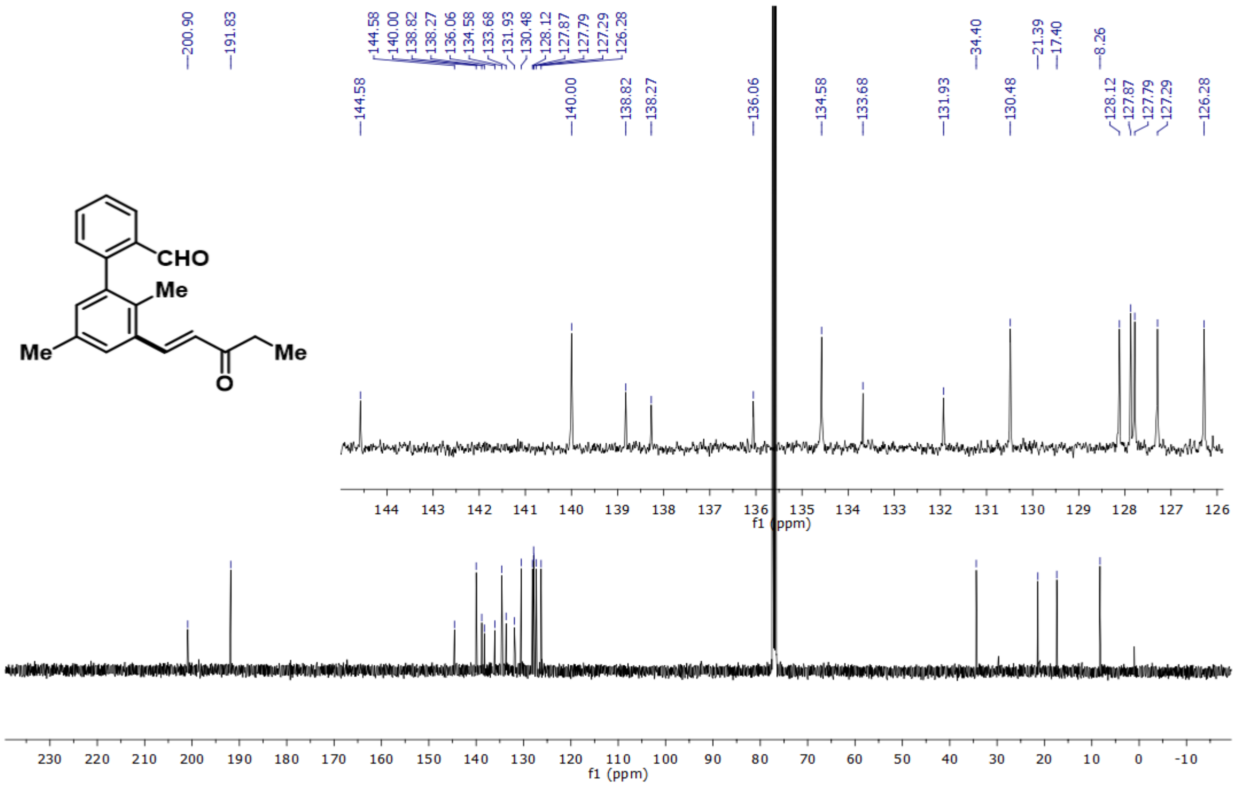

(E)-2-(7-(3-oxobut-1-en-1-yl)benzo[d][1,3]dioxol-5-yl)benzaldehyde (**30**)

^{1}H NMR

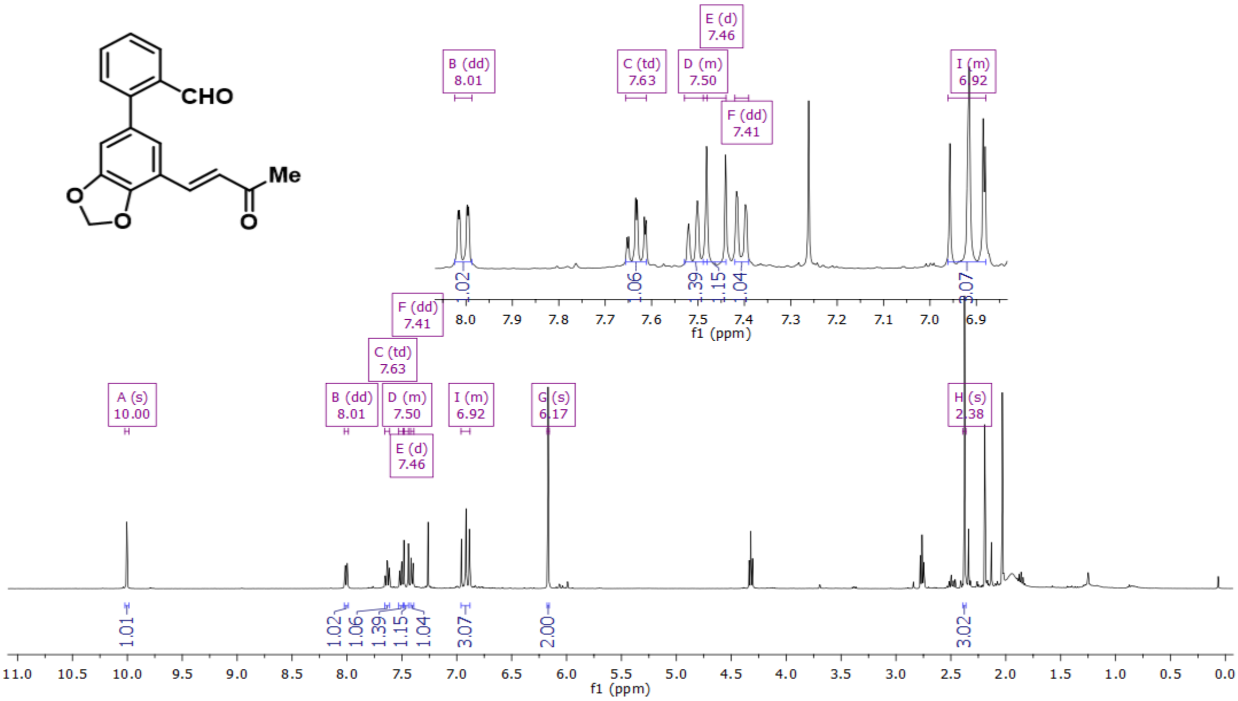

^{13}C NMR

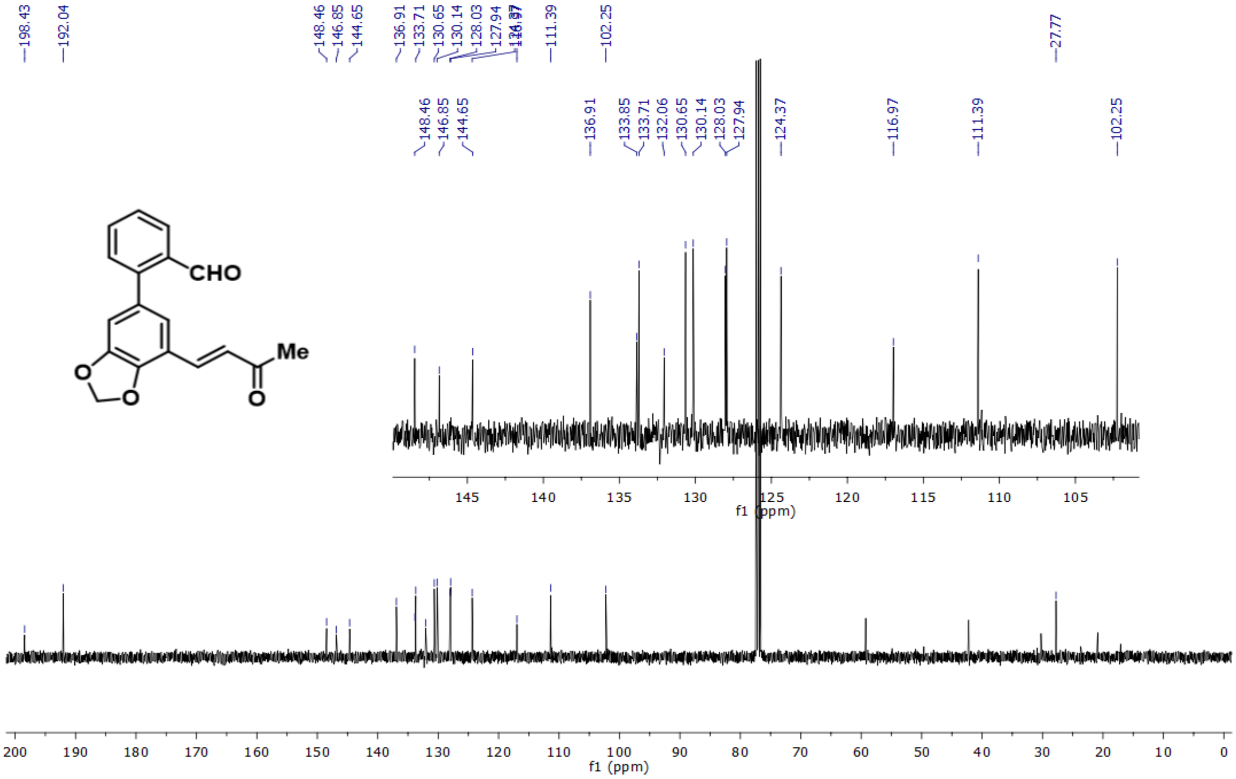

Dodecyl (E)-3-(6-(2-formylphenyl)benzo[d][1,3]dioxol-4-yl)acrylate **(31)**

^{1}H NMR

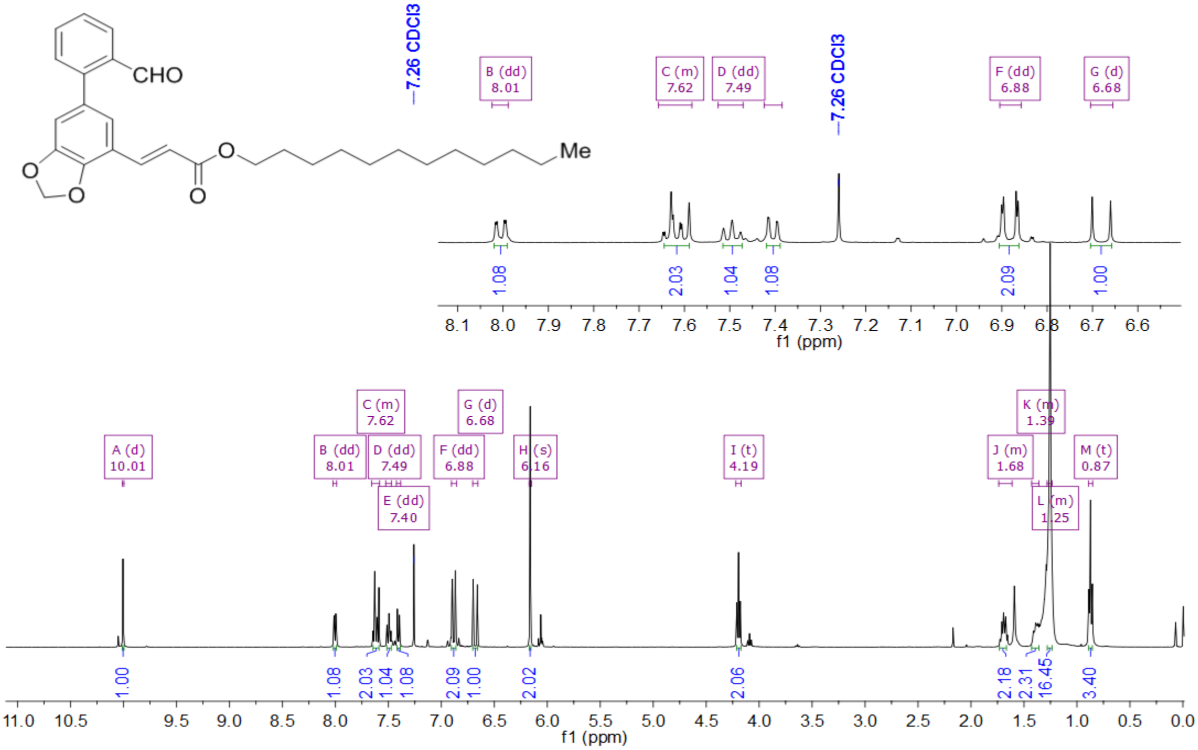

^{13}C NMR

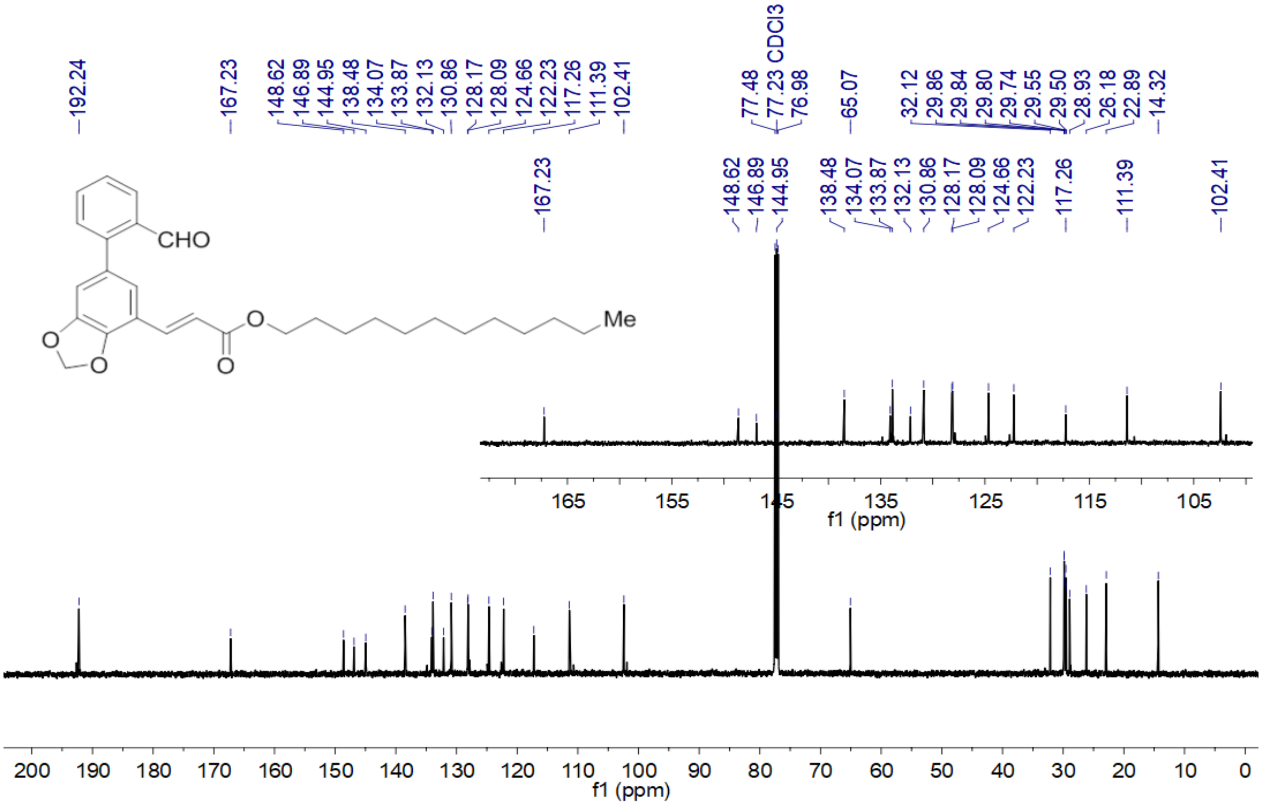

***(E)*-3-(2'-formyl-5',6-dimethyl-[1,1'-biphenyl]-3-yl)acrylonitrile** (32)

^{1}H NMR

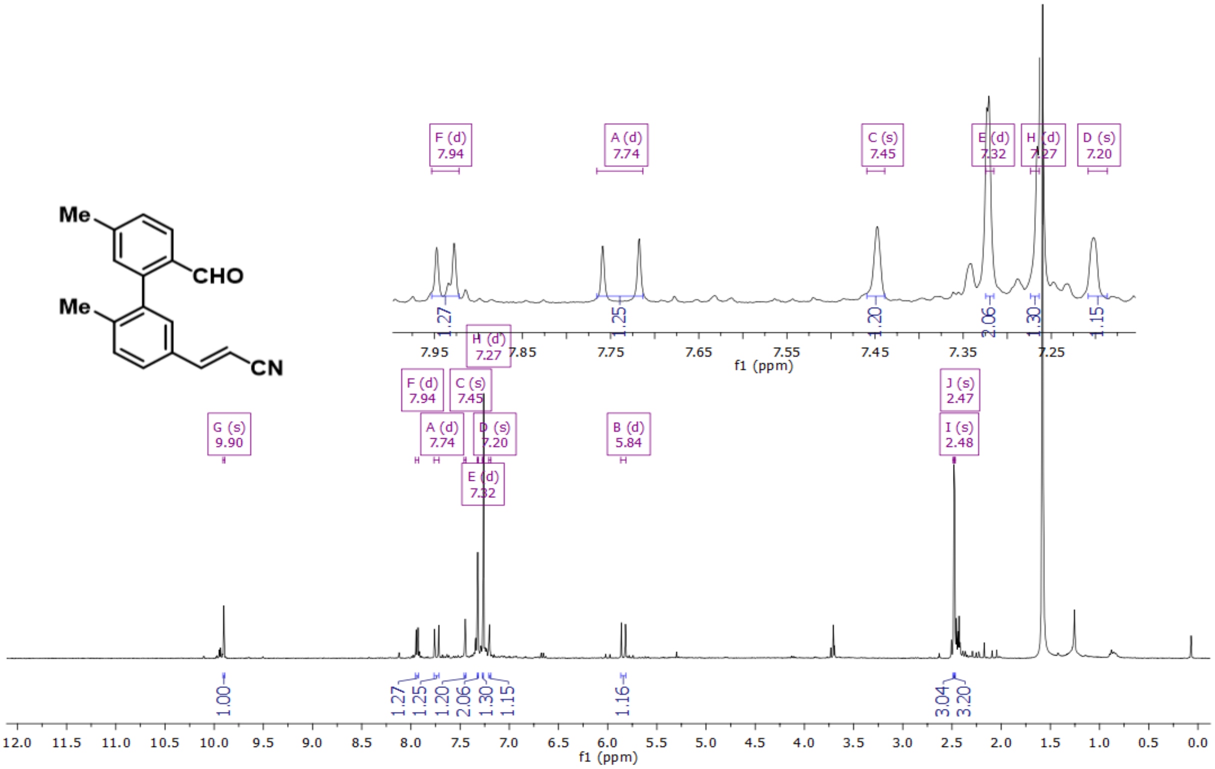

^{13}C NMR

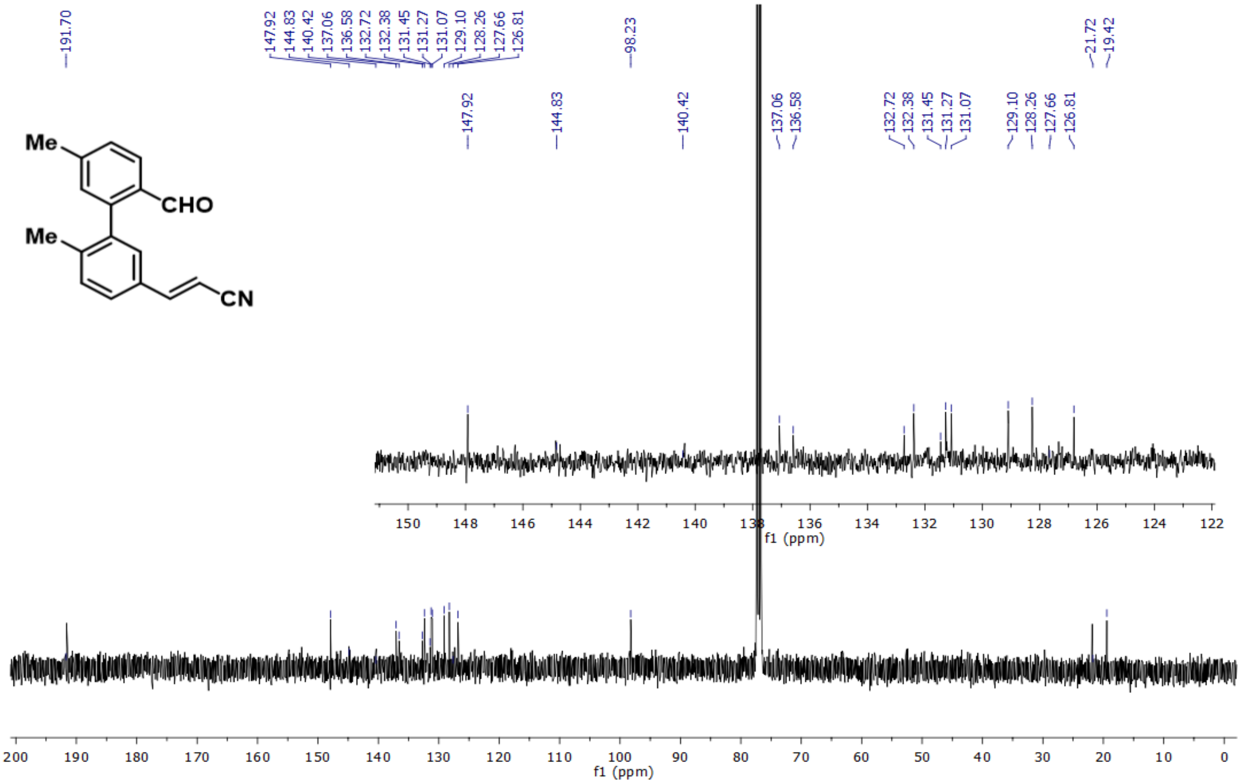

***(E)-4-methoxy-3'-methyl-5'-(3-oxopent-1-en-1-yl)-[1,1'-biphenyl]-2-carbaldehyde* (33)**

^{1}H NMR

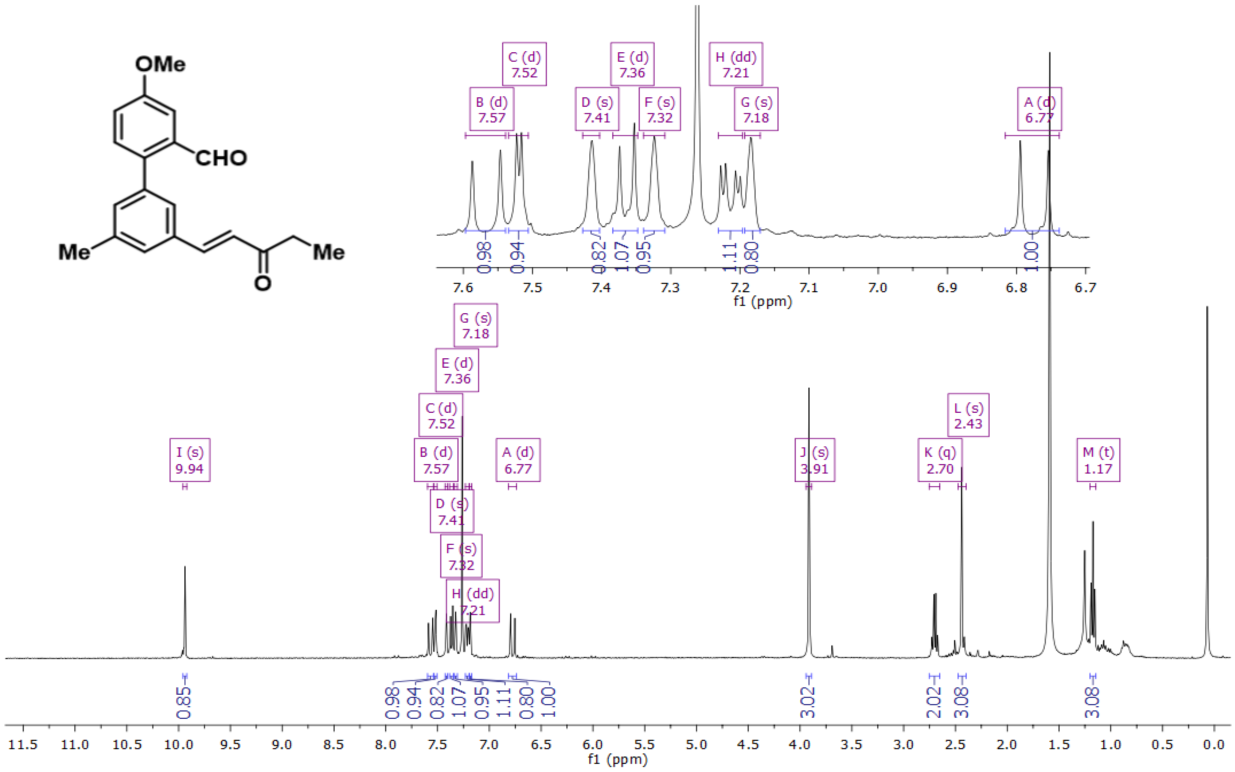

^{13}C NMR

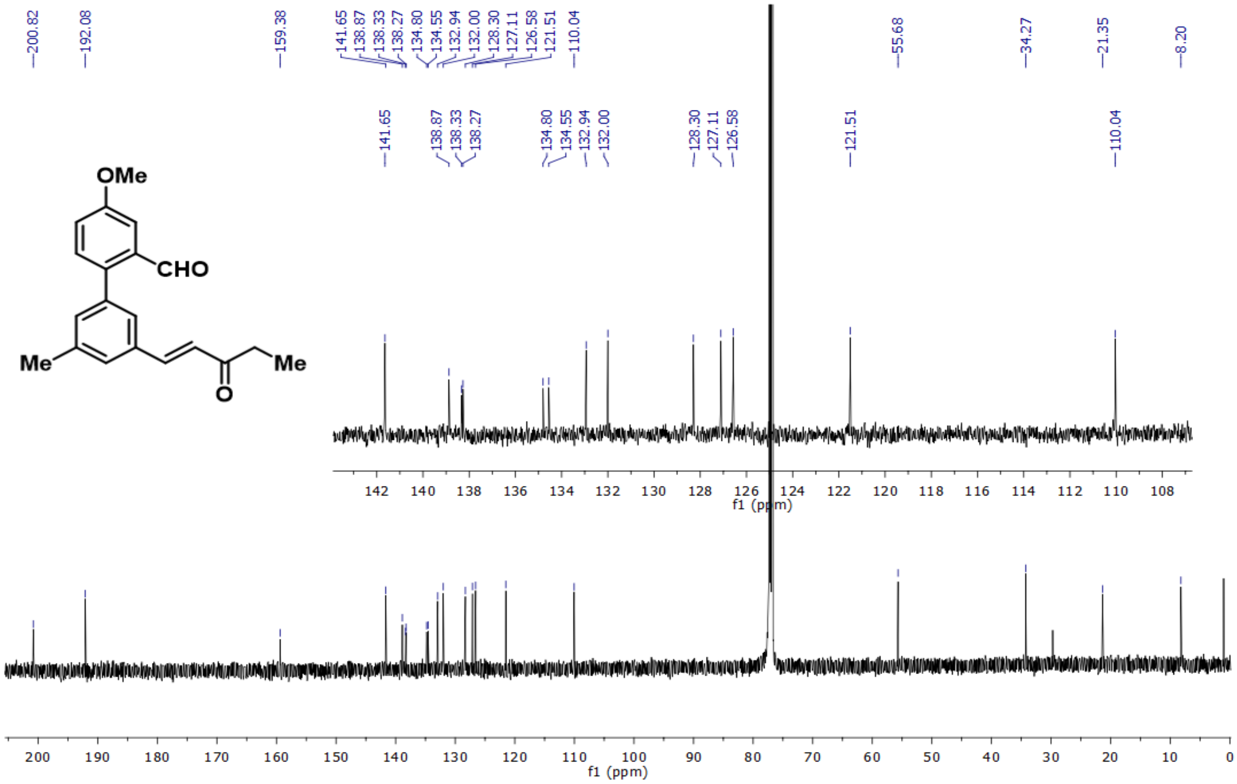

(E)-3-(2'-formyl-4'-methoxy-5-methyl-[1,1'-biphenyl]-3-yl)acrylonitrile (**34**)

^{1}H NMR

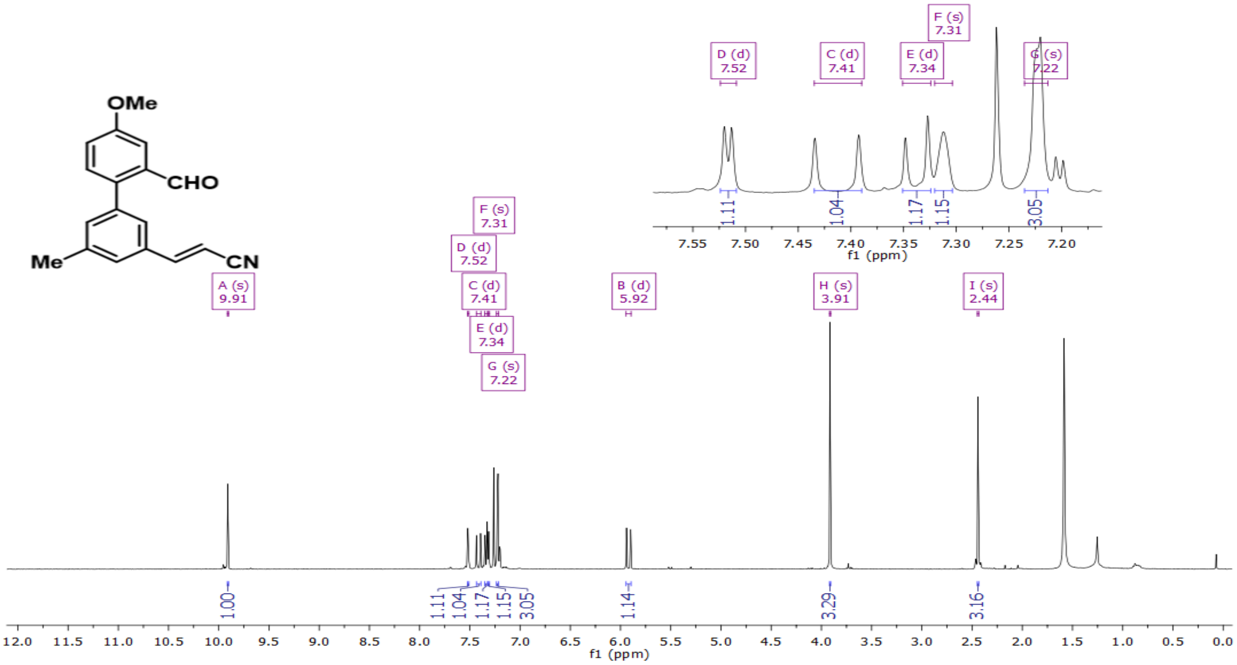

^{13}C NMR

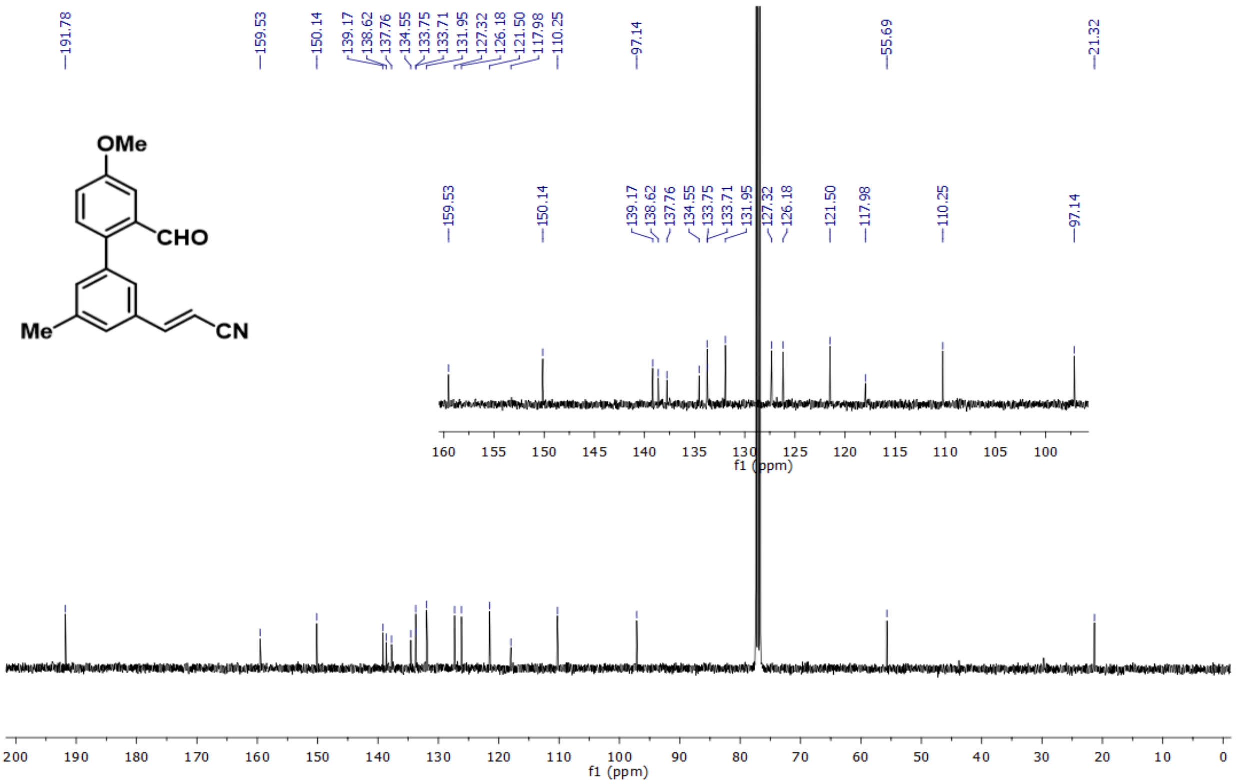

***(E)-3-(6-(2-formyl-5-methoxyphenyl)benzo[d][1,3]dioxol-4-yl)acrylonitrile* (35)**

^{1}H NMR

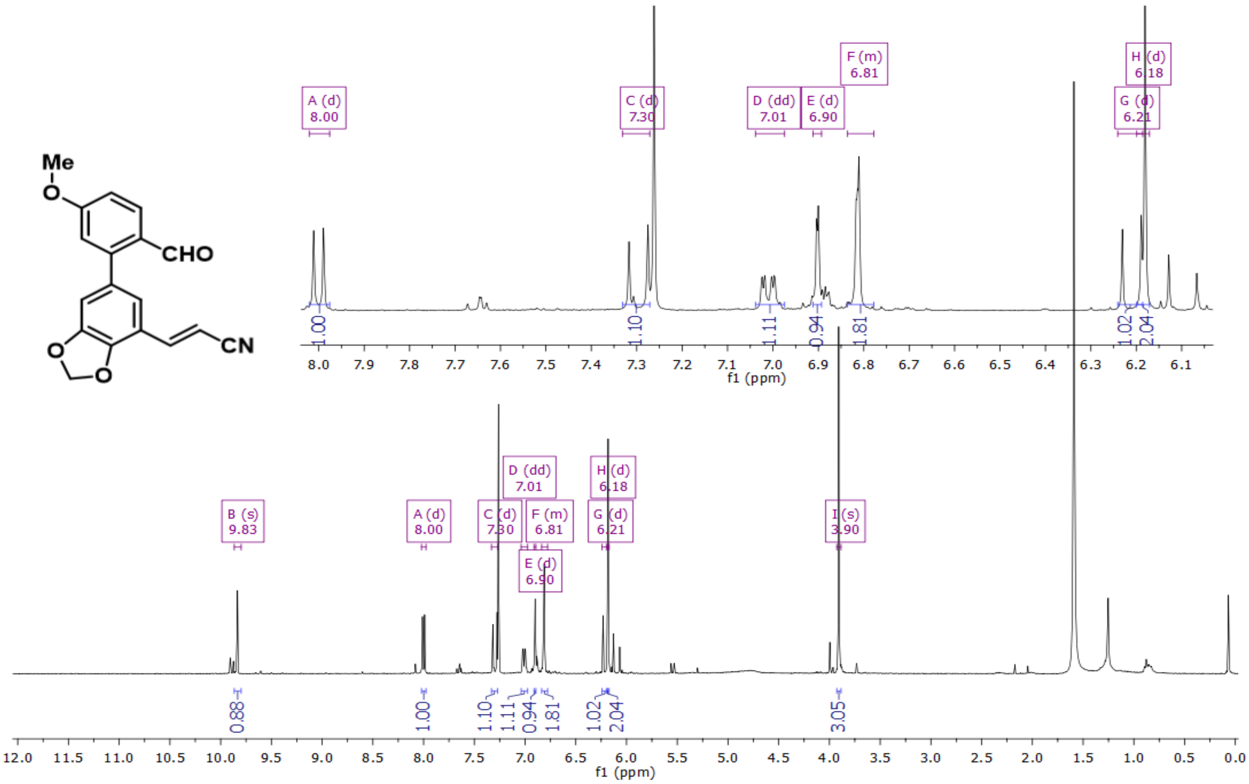

^{13}C NMR

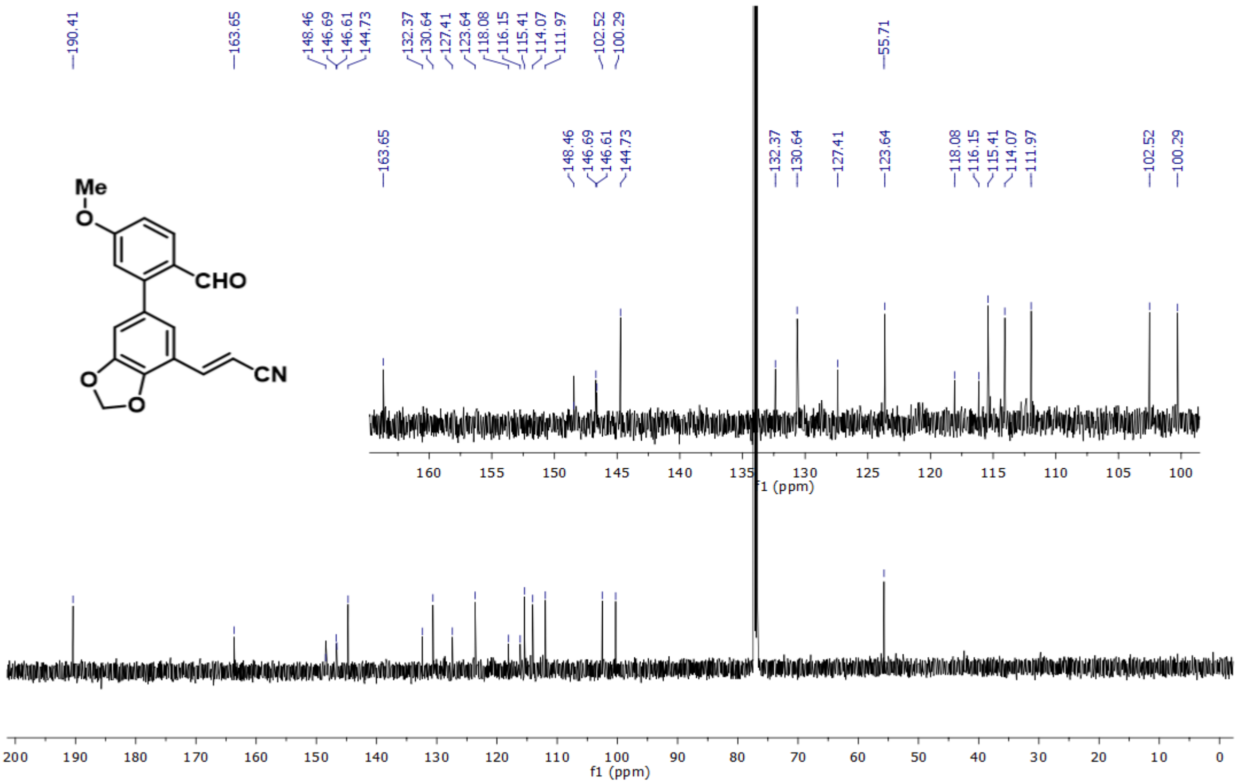

Ethyl (E)-3-(2'-formyl-[1,1'-biphenyl]-3-yl)acrylate (**36**)

^{1}H NMR

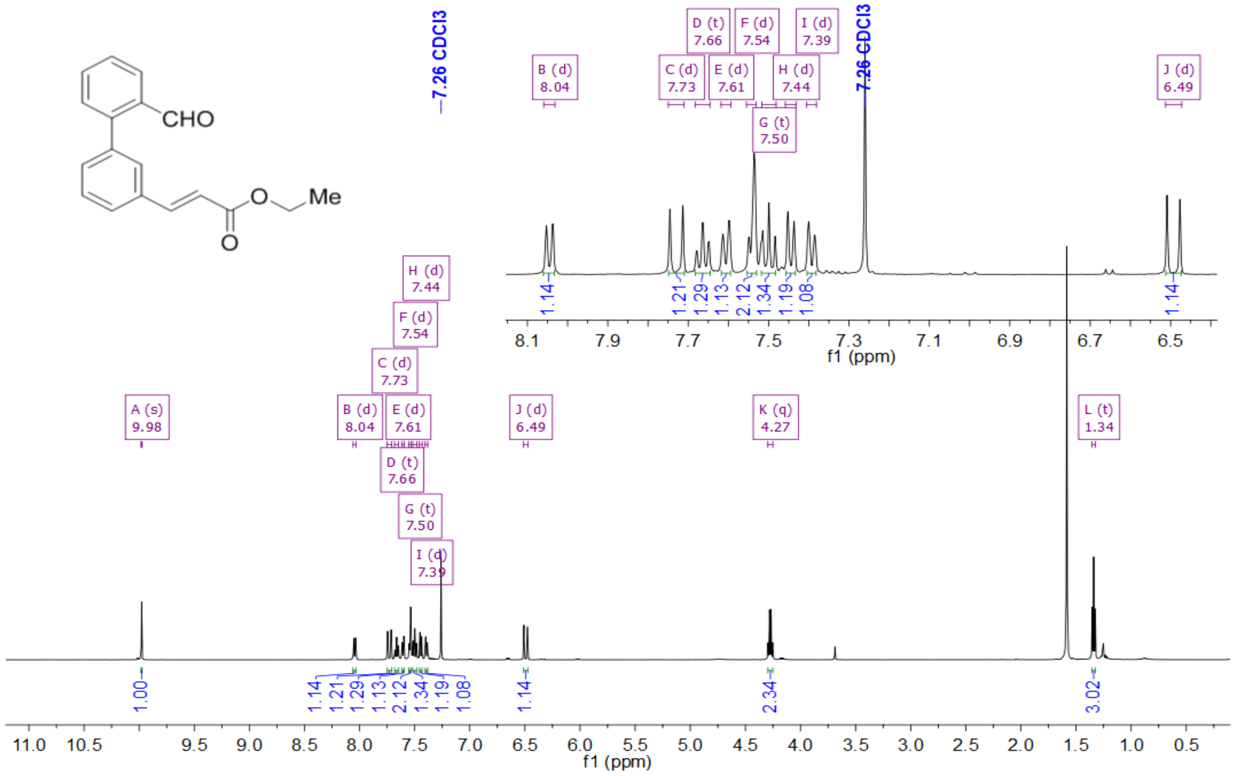

^{13}C NMR

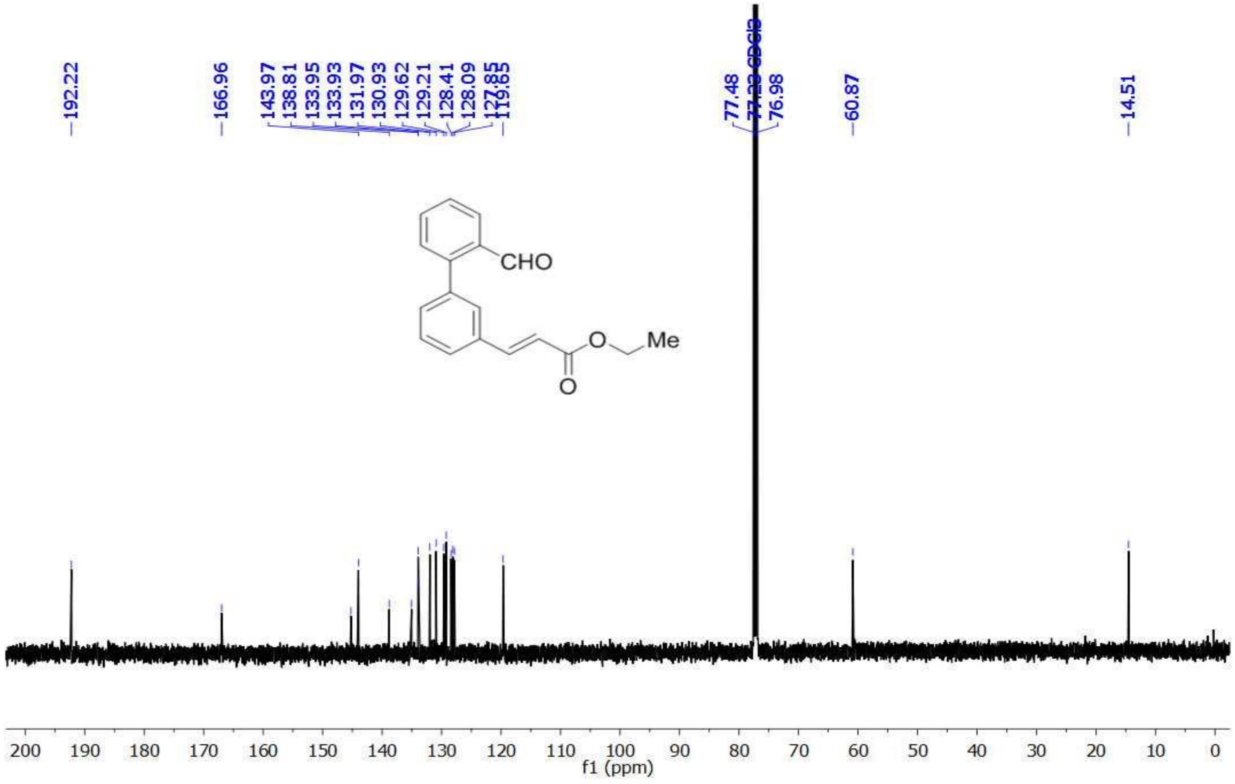

***Ethyl (E)-3-(5-chloro-2'-formyl-[1,1'-biphenyl]-3-yl)acrylate* (37)**

^{1}H NMR

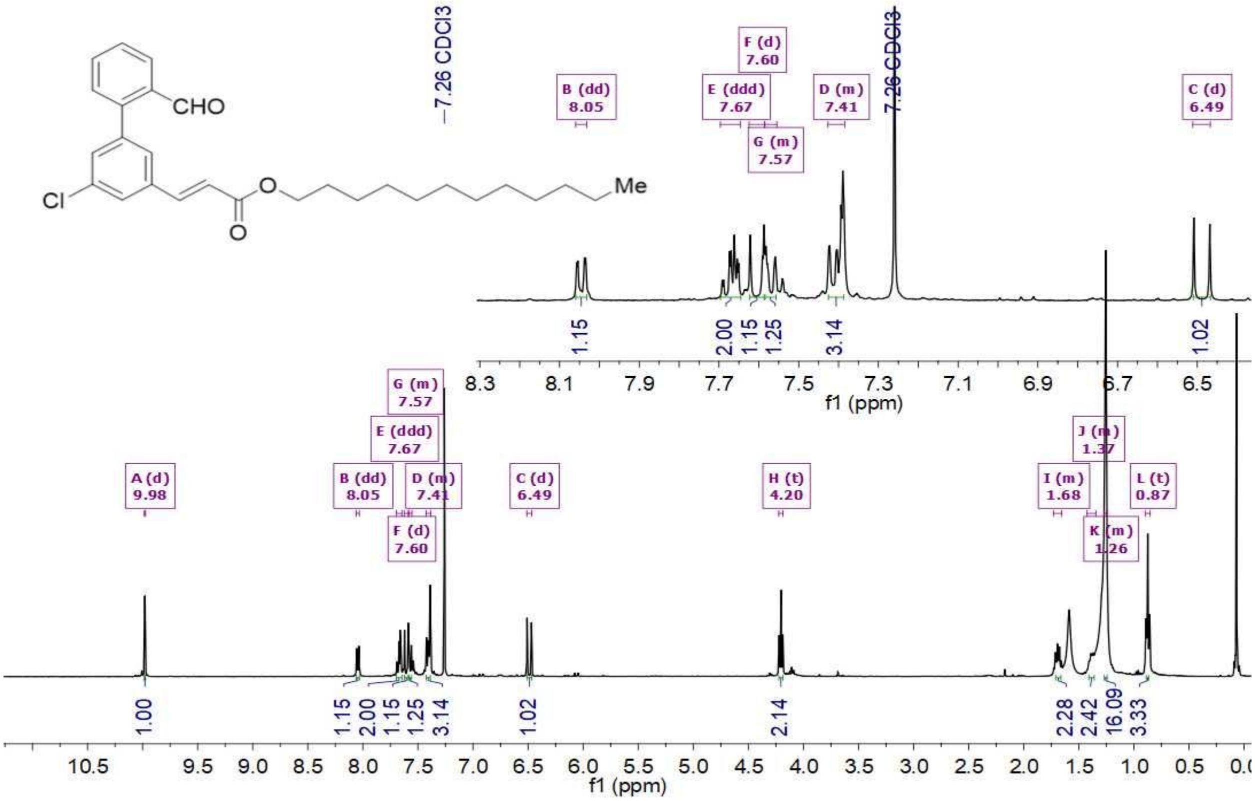

^{13}C NMR

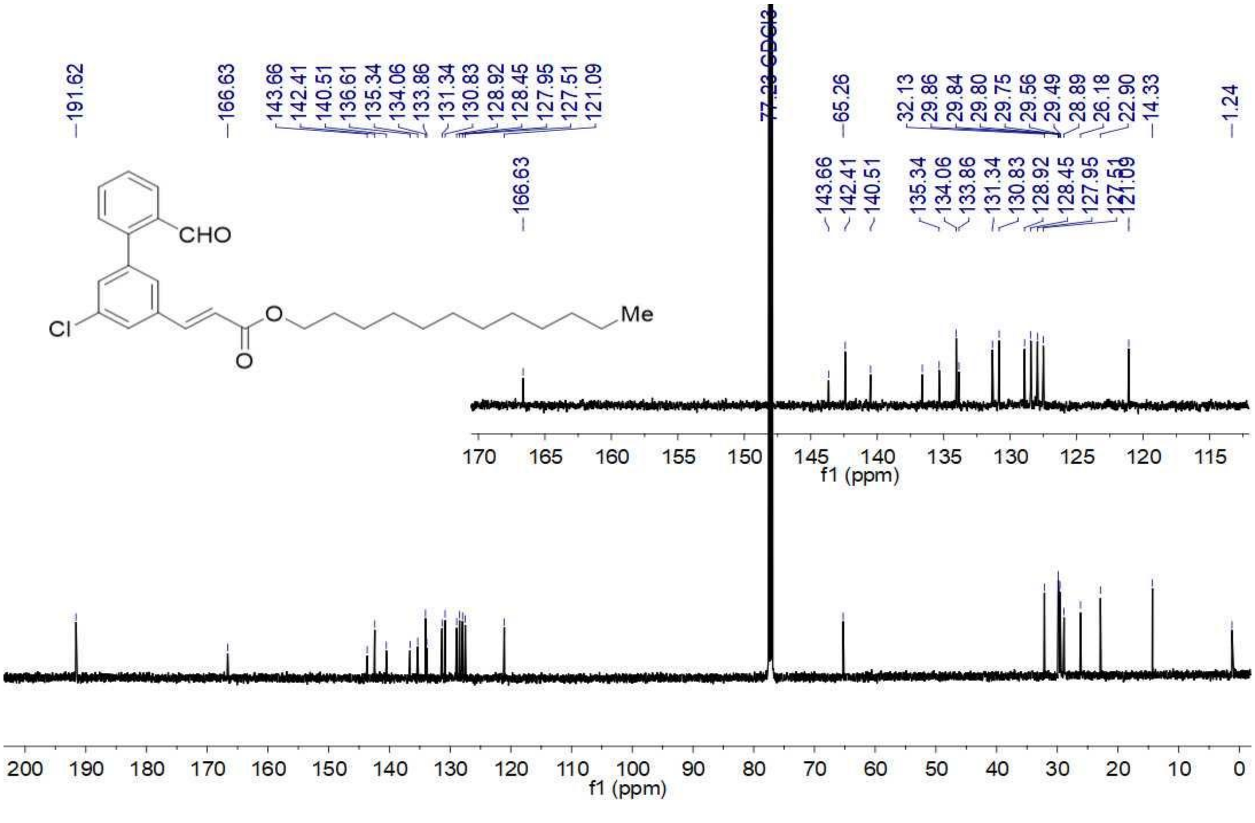

Methyl (E)-5-(3-ethoxy-3-oxoprop-1-en-1-yl)-2'-formyl-[1,1'-biphenyl]-3-carboxylate **(38)**

^{1}H NMR

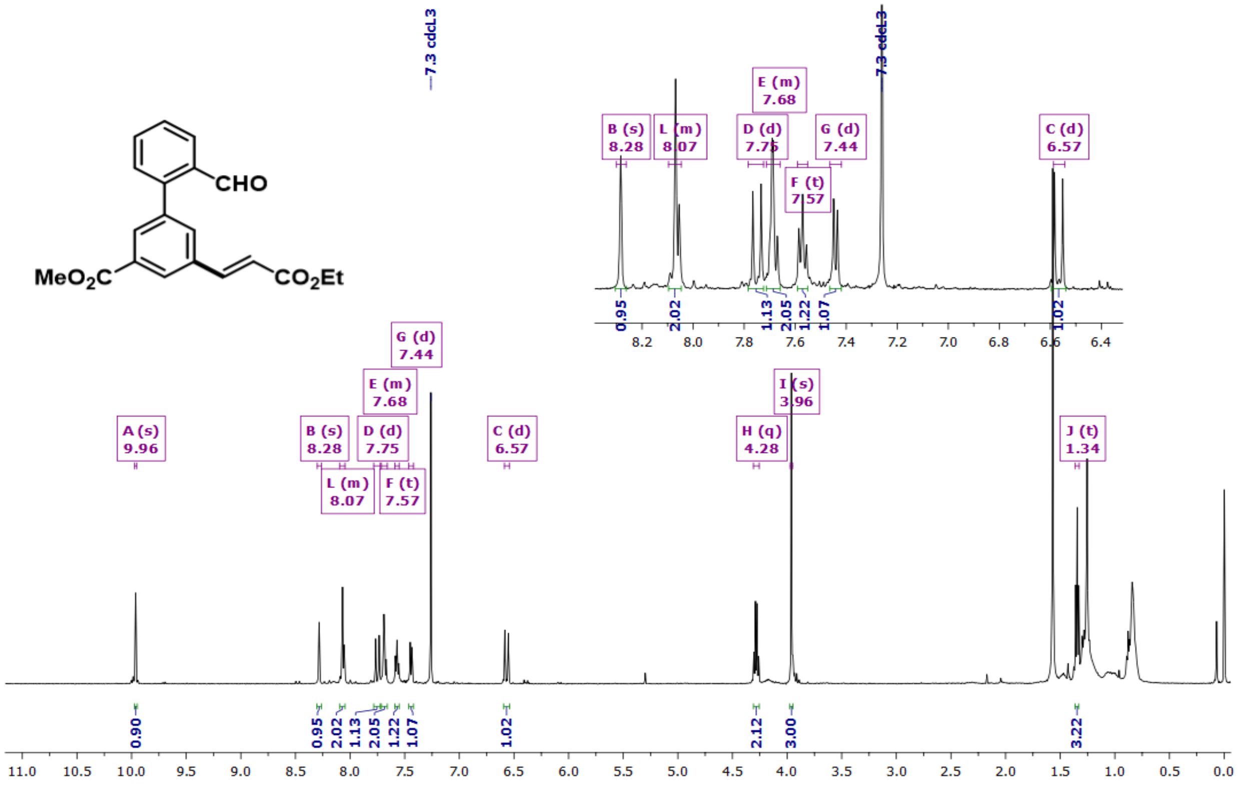

^{13}C NMR

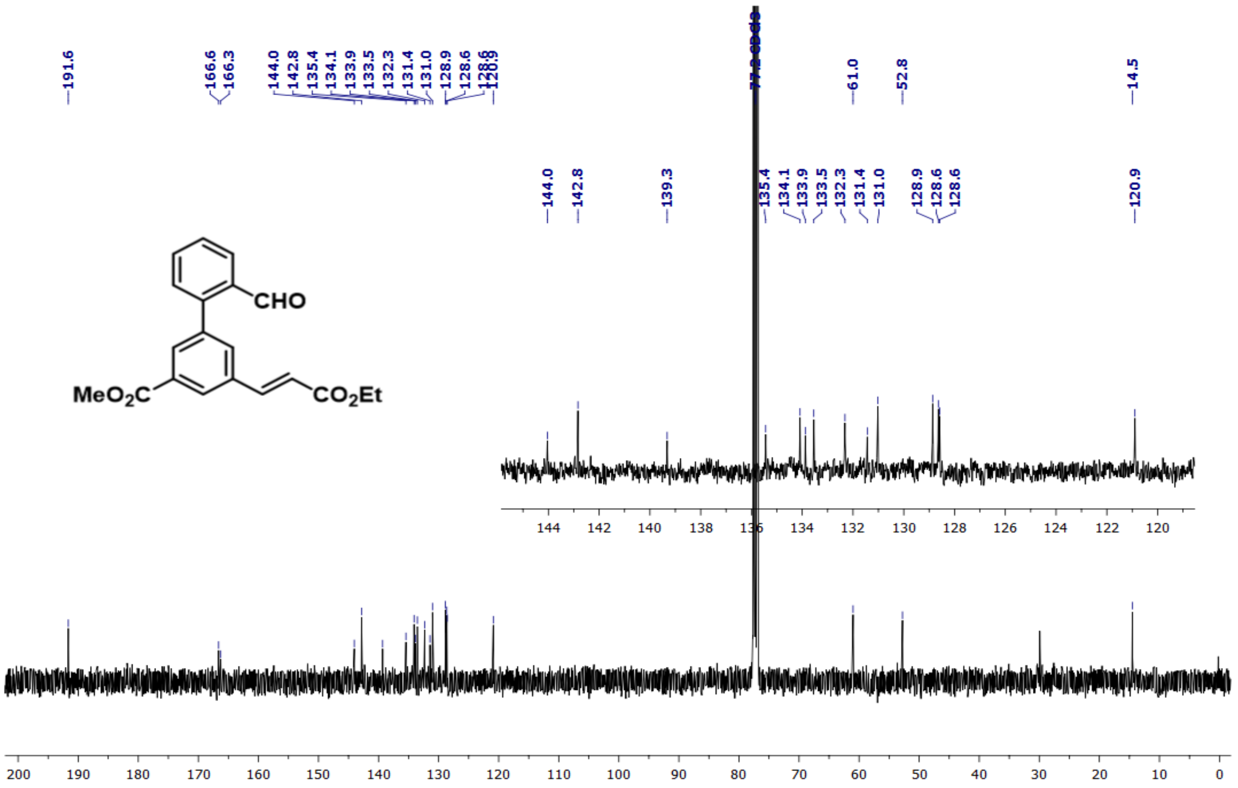

Dodecyl (E)-3-(2'-formyl-4-methyl-[1,1'-biphenyl]-3-yl)acrylate (**39**)

^{1}H NMR

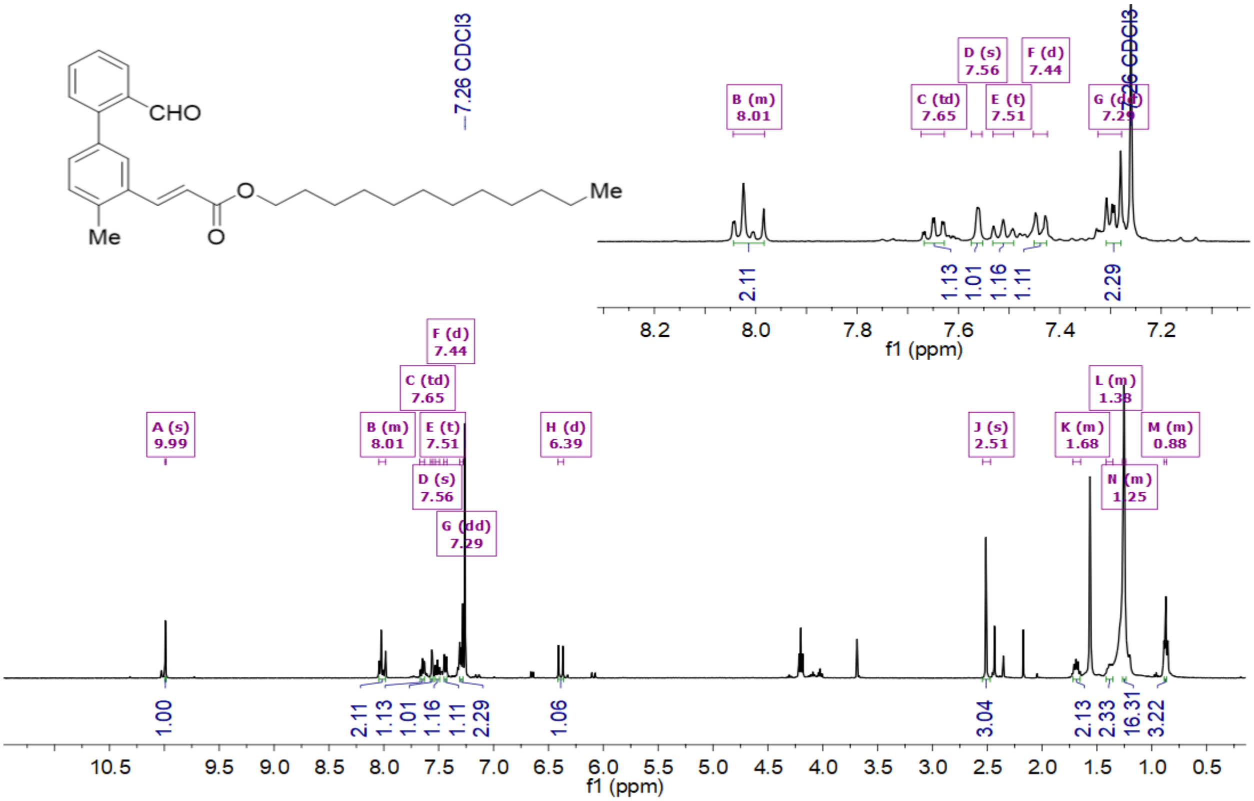

^{13}C NMR

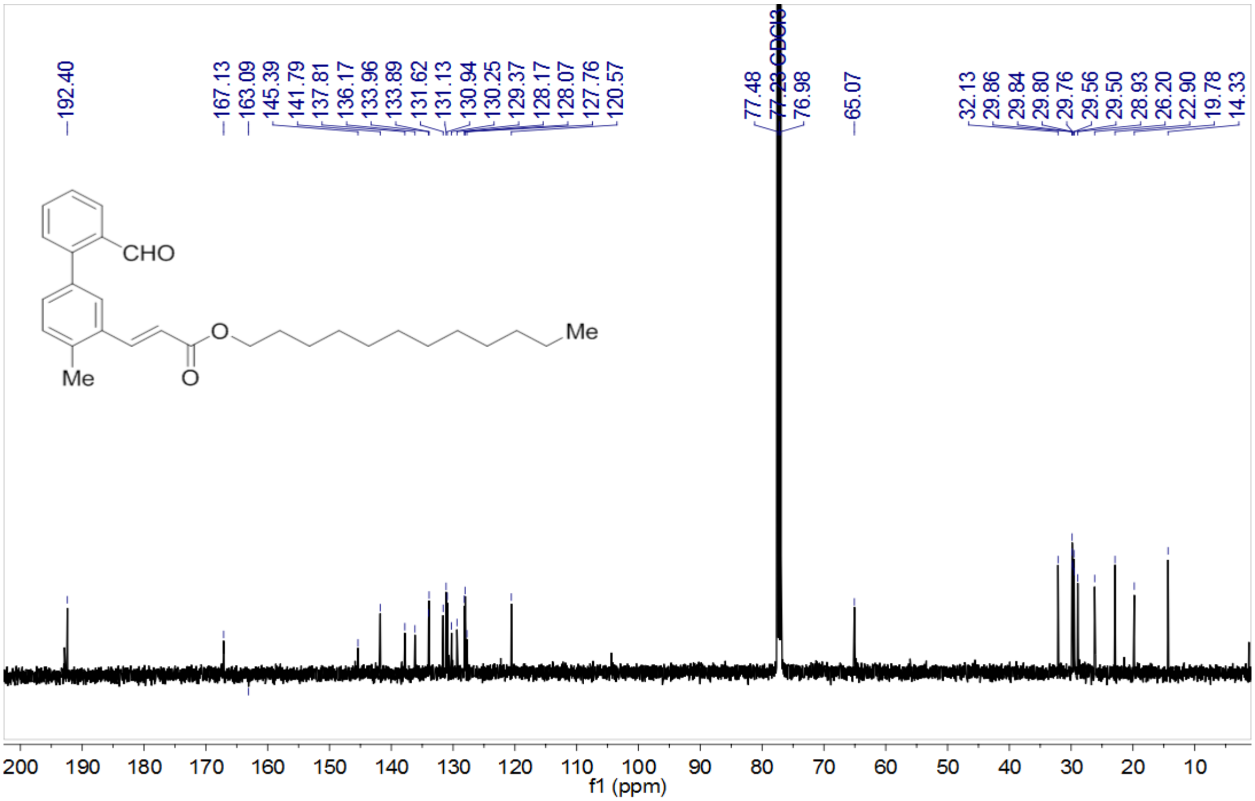

***Ethyl (E)-3-(4-chloro-2'-formyl-[1,1'-biphenyl]-3-yl)acrylate* (40)**

^{1}H NMR

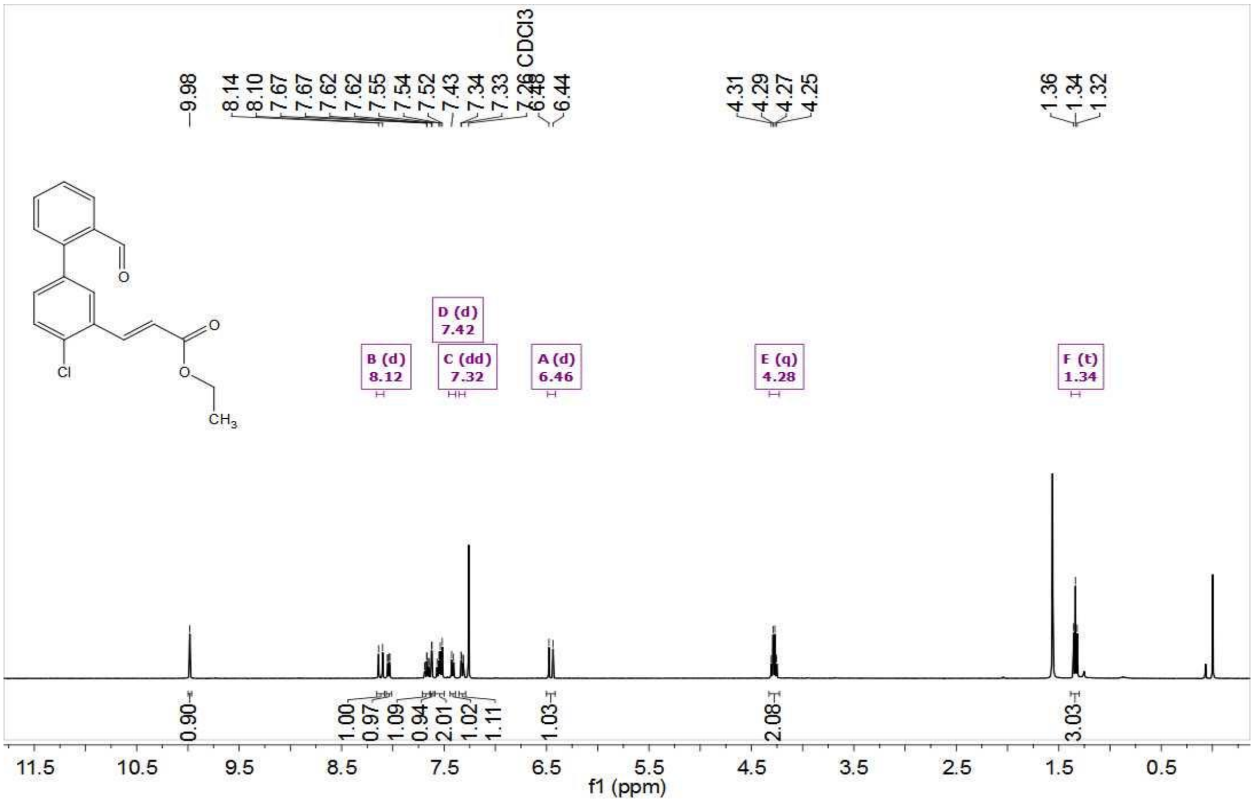

^{13}C NMR

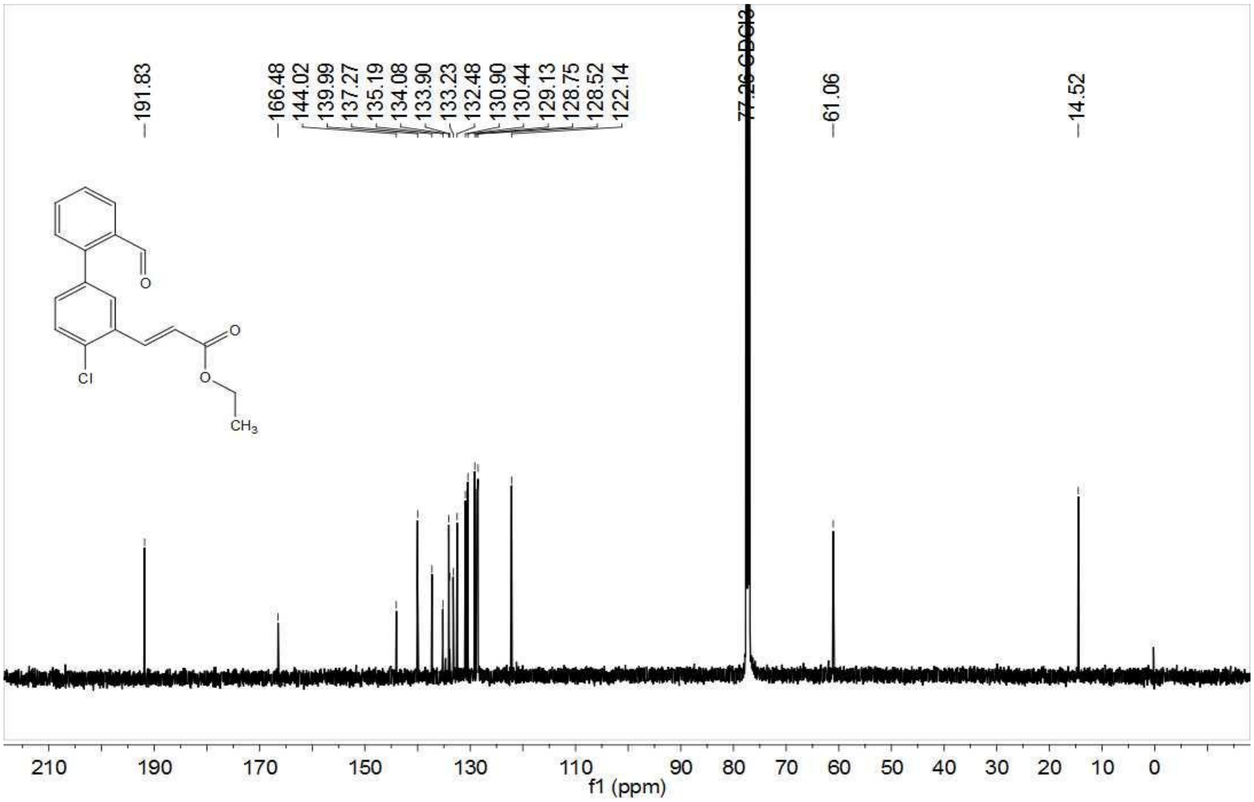

***Ethyl (E)-3-(2'-formyl-6-methyl-[1,1'-biphenyl]-3-yl)acrylate* (41)**

^{1}H NMR

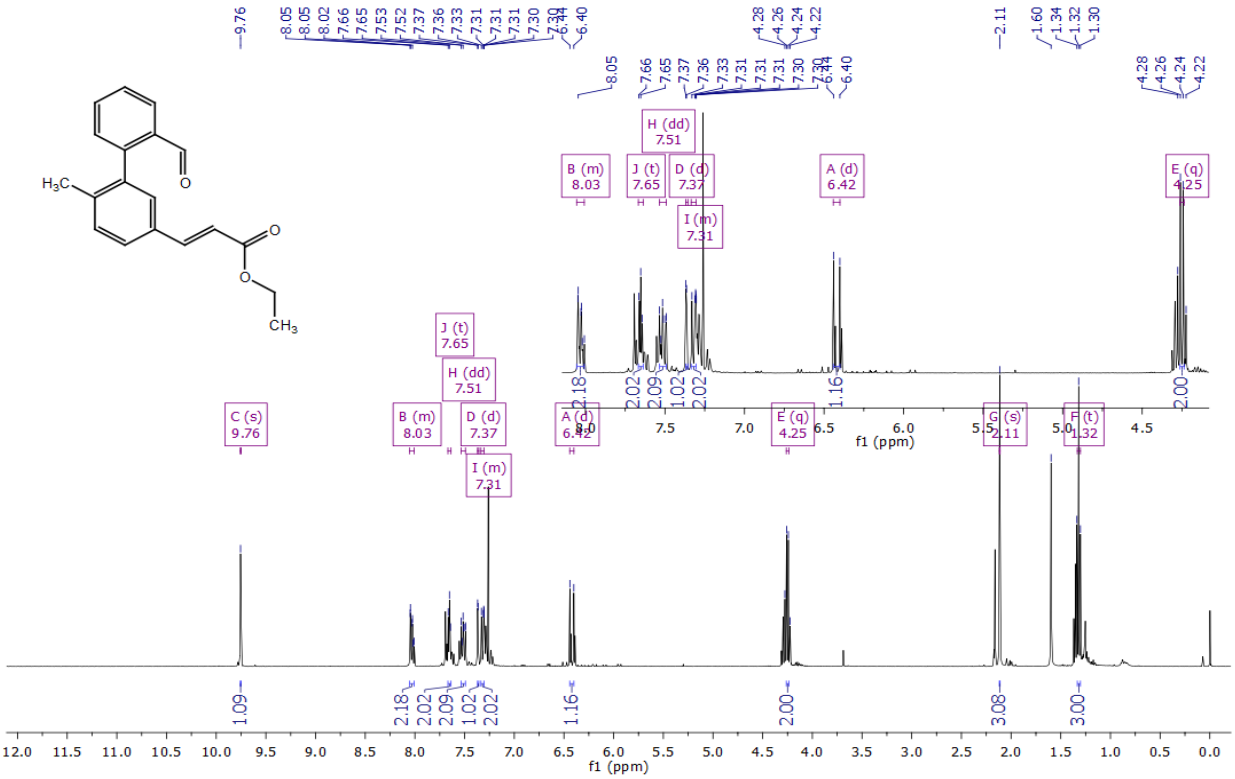

^{13}C NMR

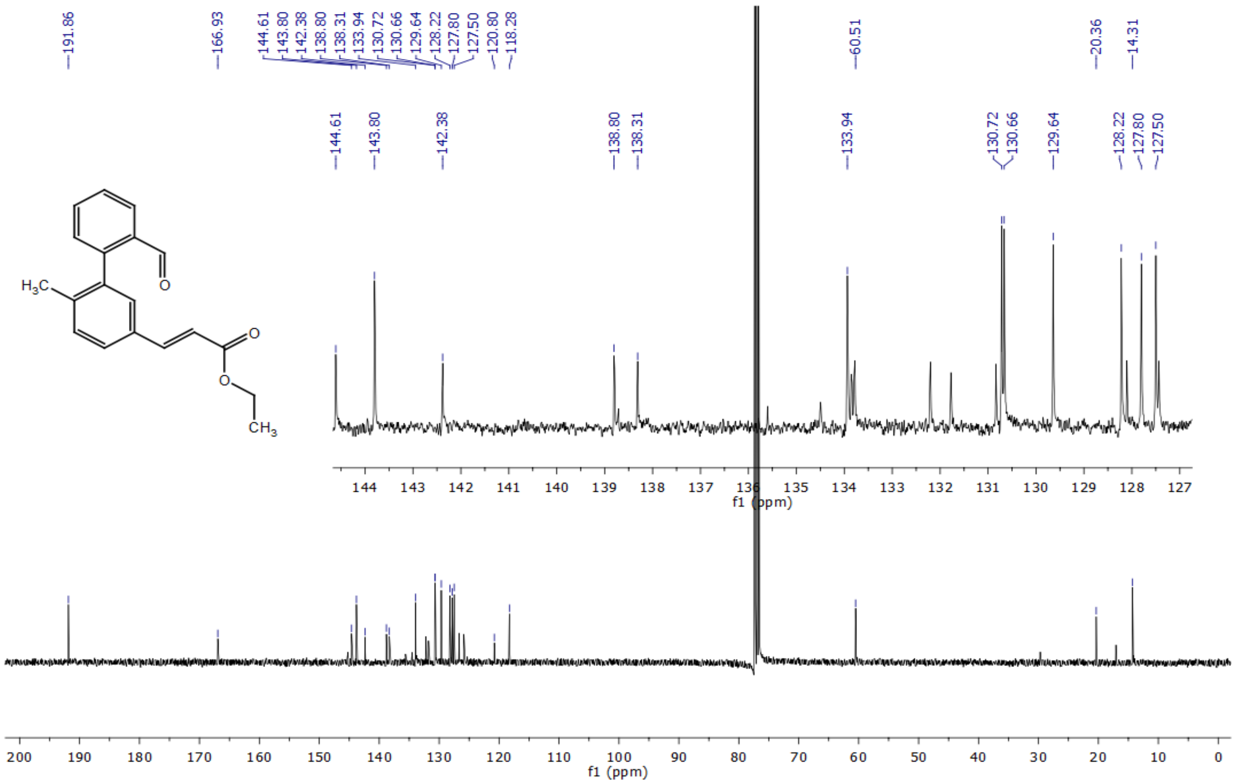

Ethyl (E)-3-(2'-formyl-2,6-dimethyl-[1,1'-biphenyl]-3-yl)acrylate (**42**)

^{1}H NMR

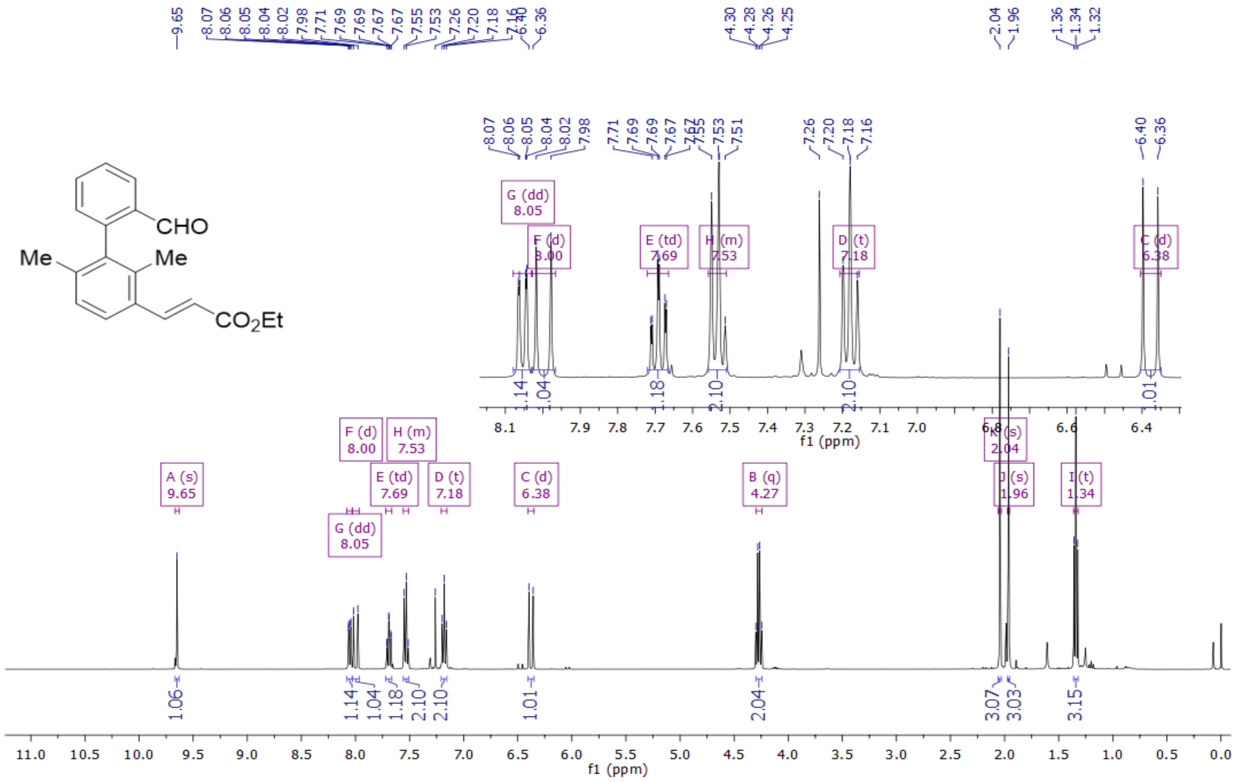

^{13}C NMR

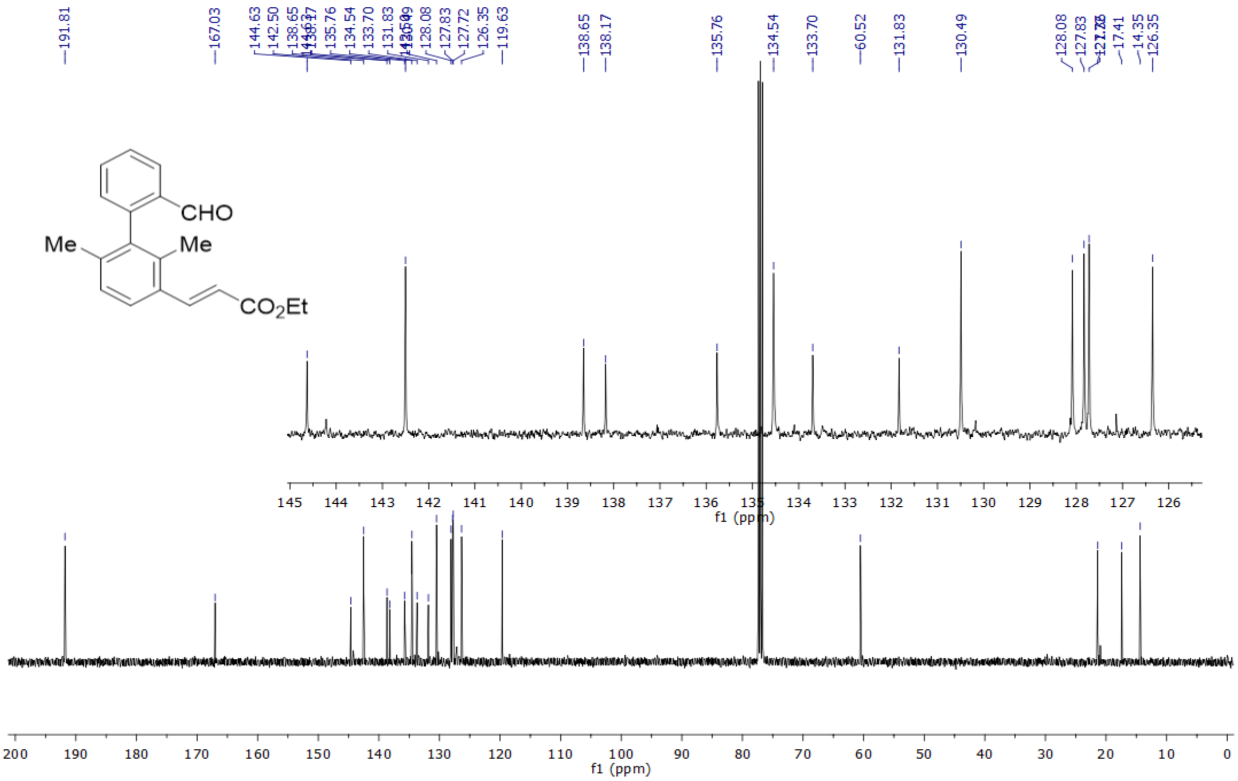

***Ethyl (E)-3-(2'-formyl-2,5-dimethyl-[1,1'-biphenyl]-3-yl)acrylate* (43)**

^{1}H NMR

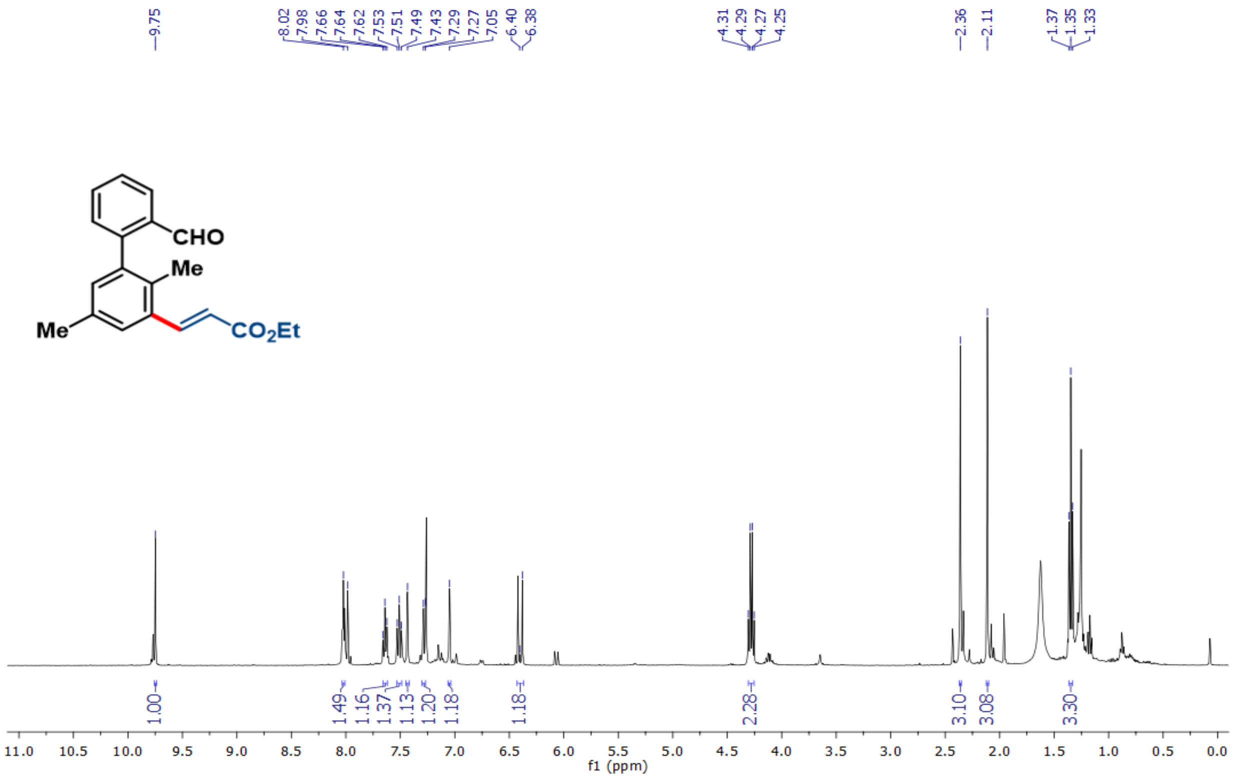

^{13}C NMR

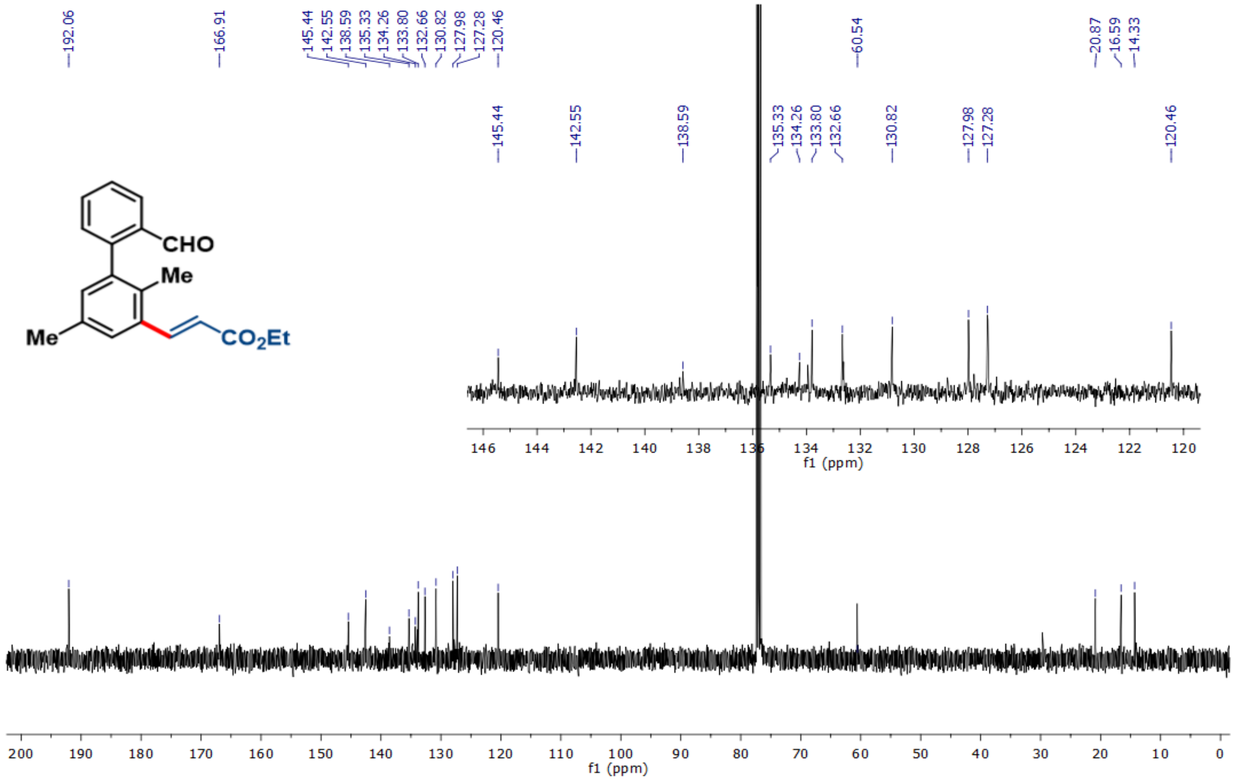

***Ethyl (E)-3-(6-(2-formylphenyl)benzo[d][1,3]dioxol-4-yl)acrylate* (44)**

^{1}H NMR

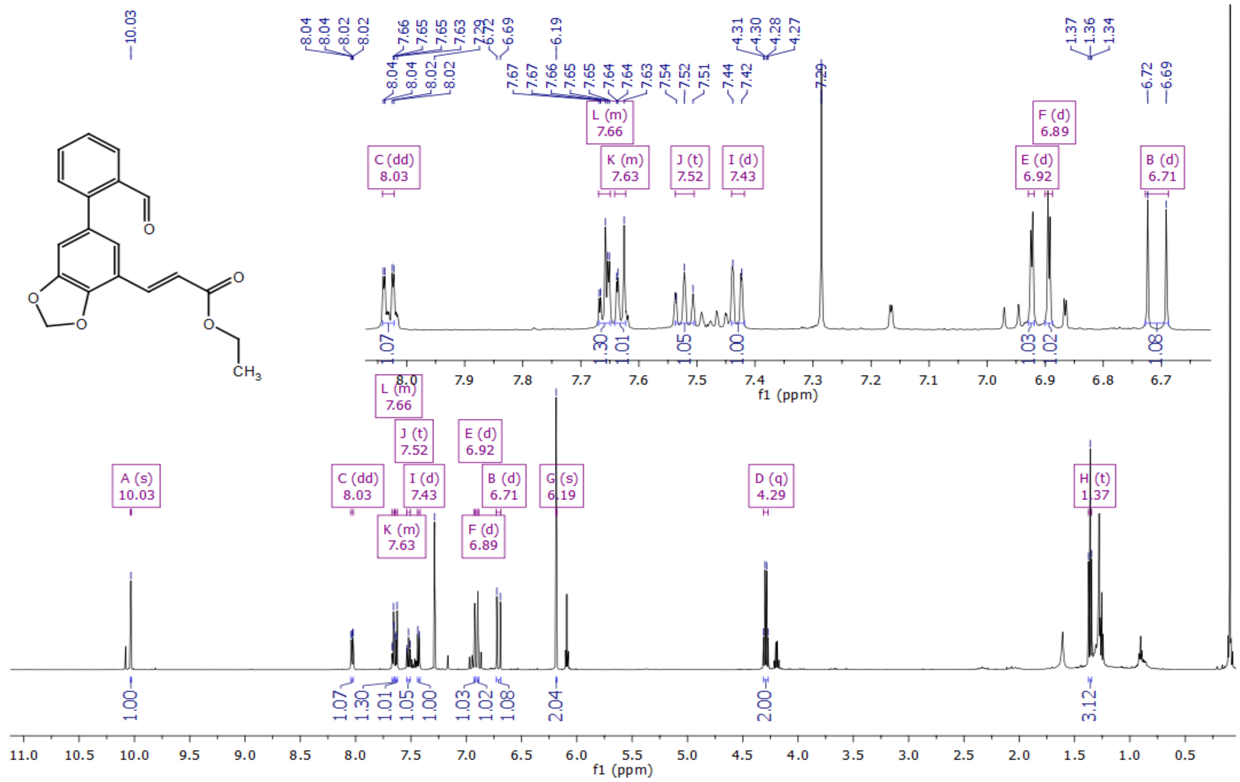

^{13}C NMR

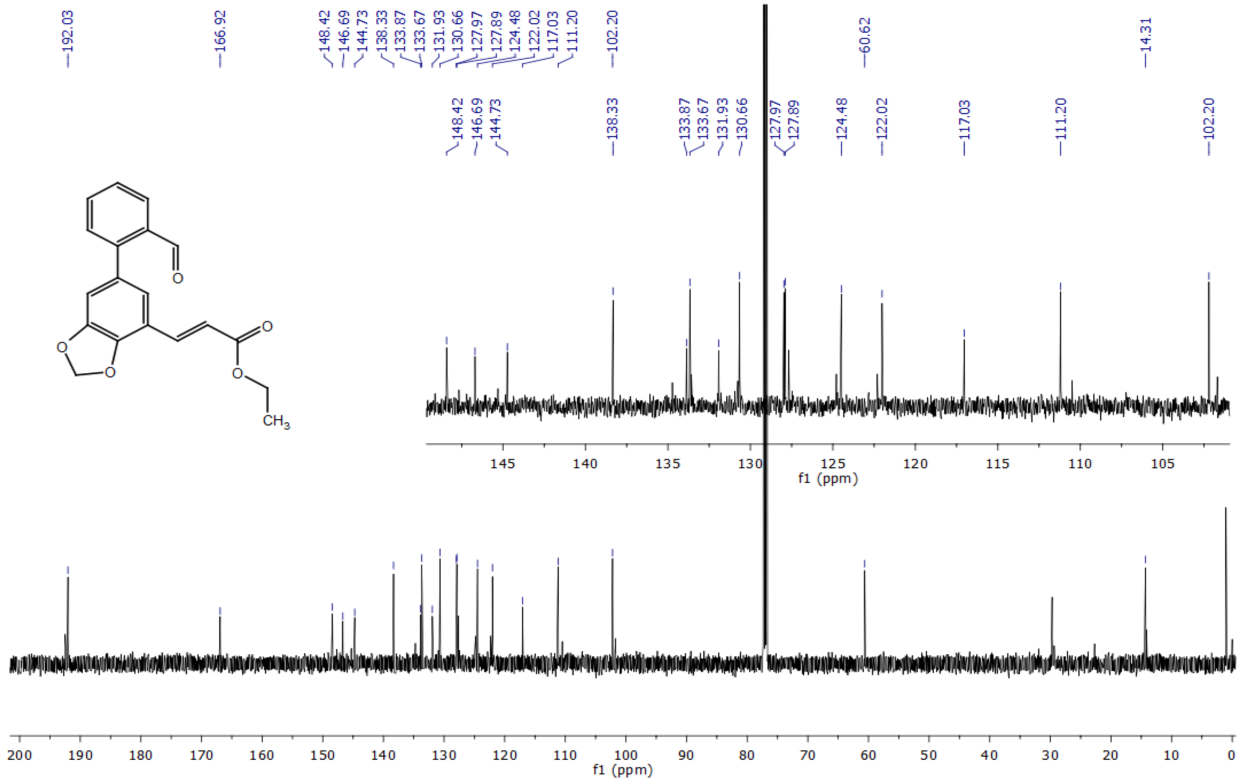

***Ethyl (E)-3-(7-(2-formylphenyl)-2,3-dihydrobenzo[b][1,4]dioxin-5-yl)acrylate* (45)**

^{1}H NMR

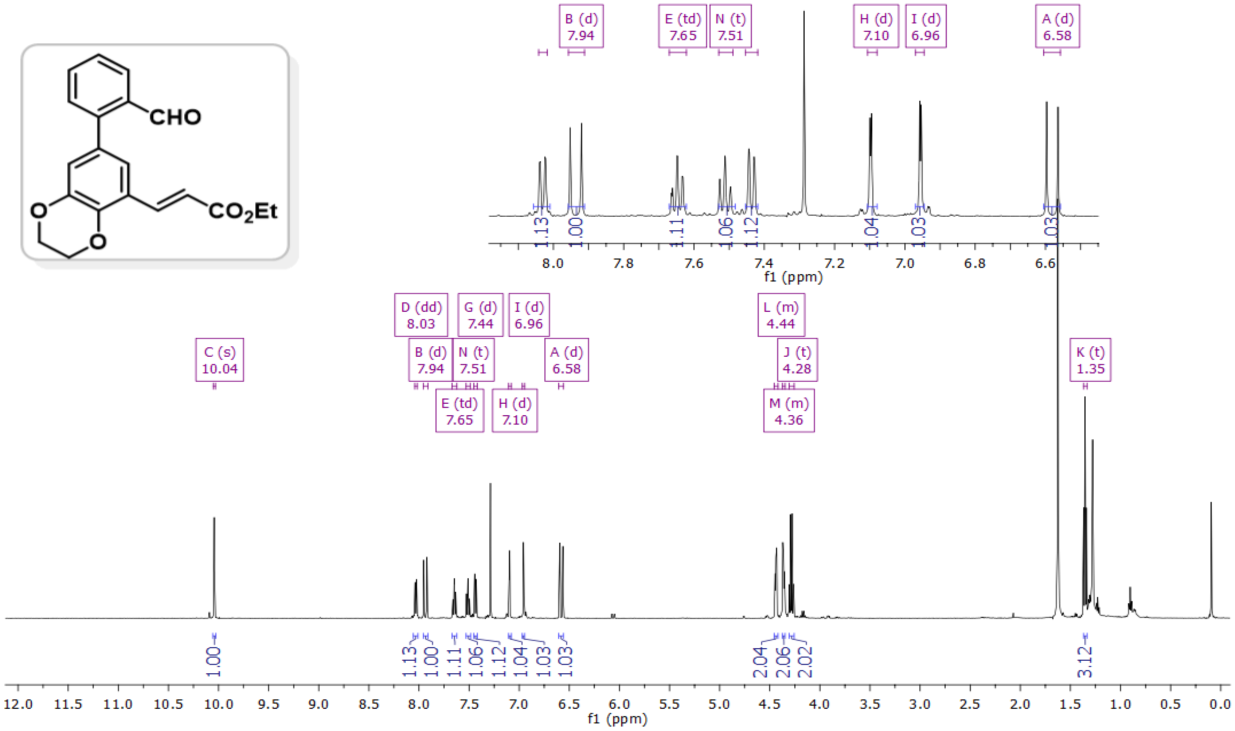

^{13}C NMR

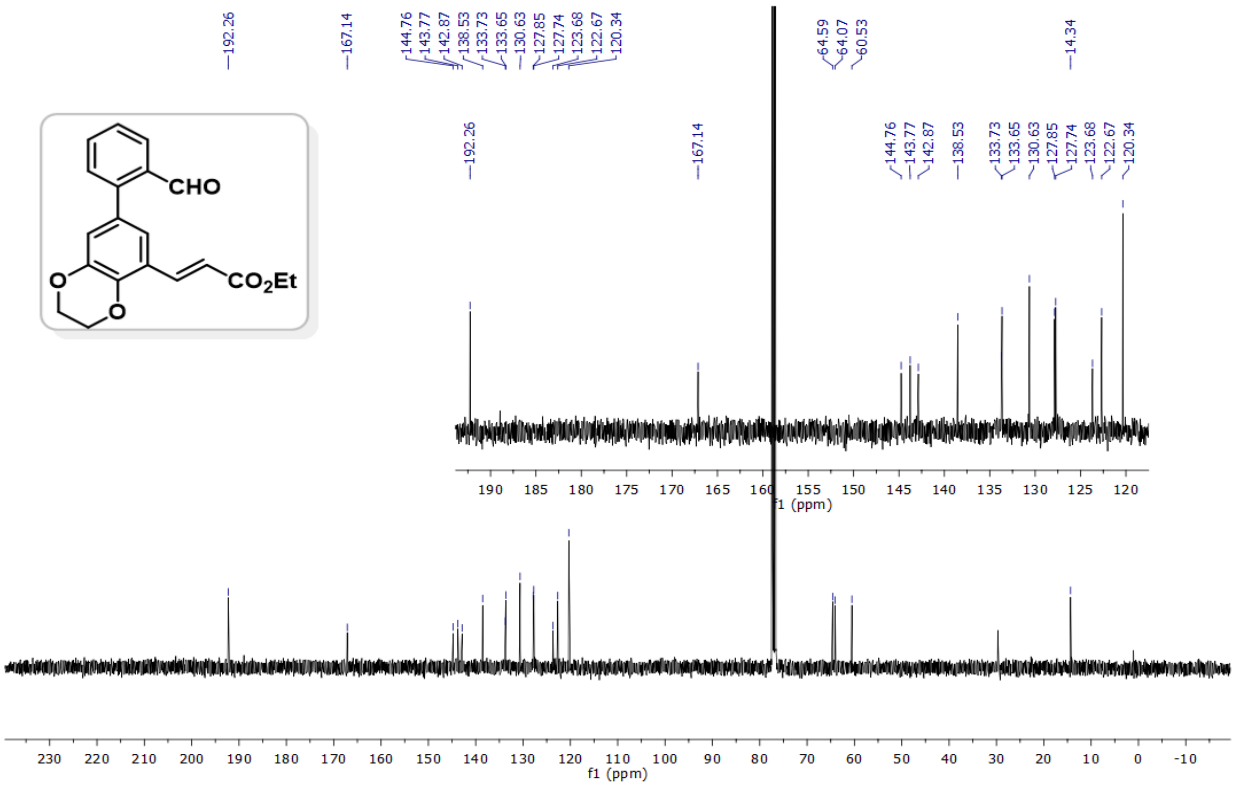

***(E)*-2',5-dimethyl-5'-(3-oxopent-1-en-1-yl)-[1,1'-biphenyl]-2-carbaldehyde** (**46**)

^{1}H NMR

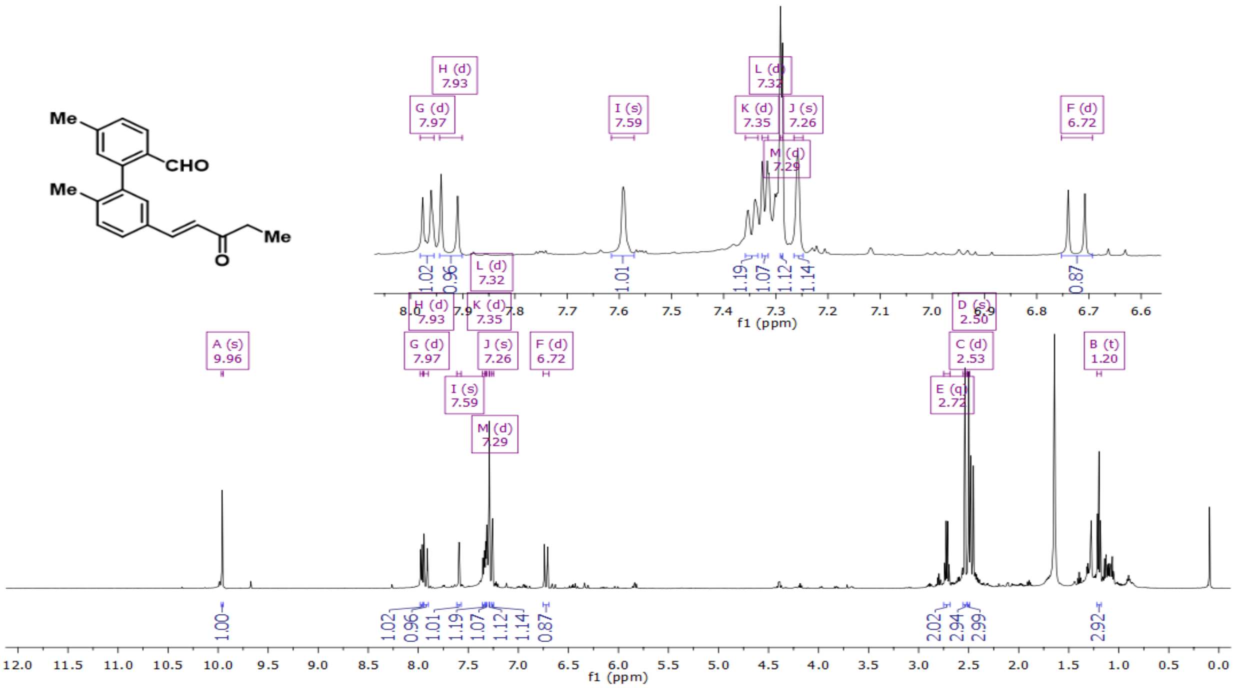

^{13}C NMR

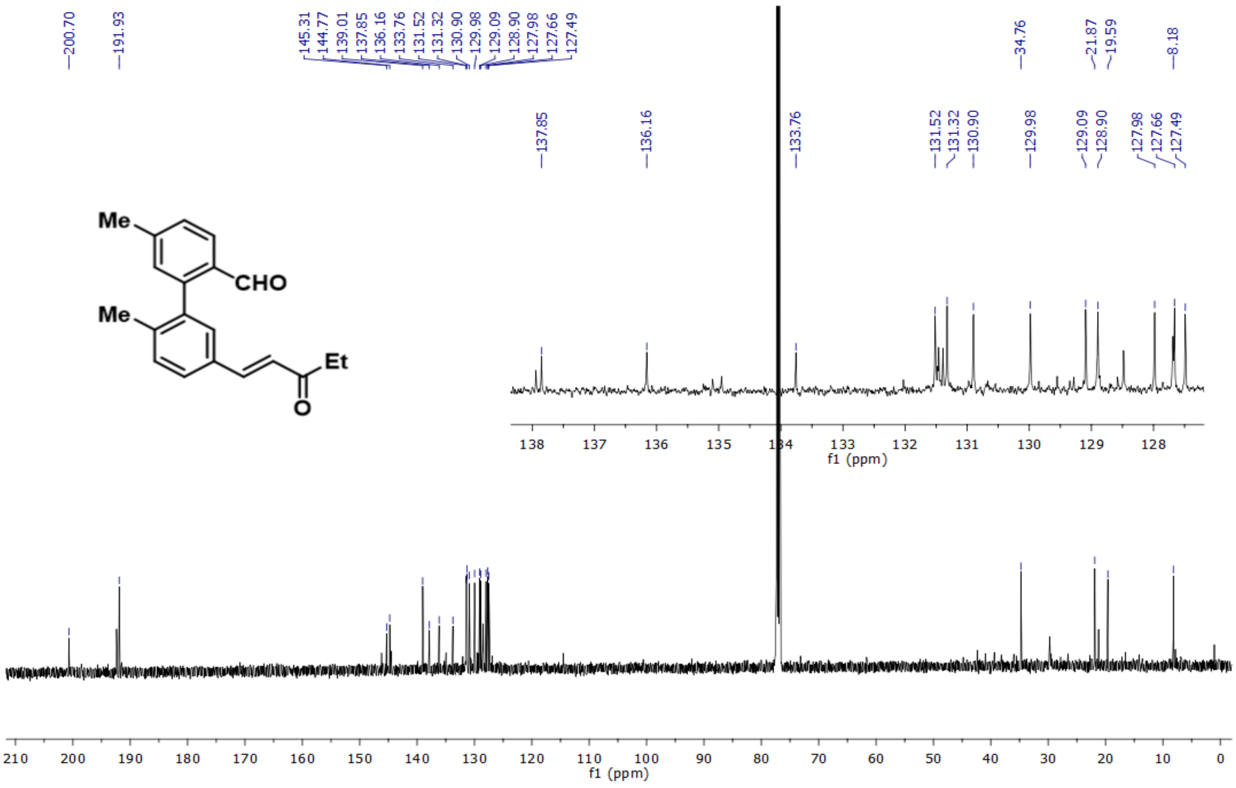

***Dodecyl (E)-3-(2'-formyl-4-methoxy-5'-methyl-[1,1'-biphenyl]-3-yl)acrylate* (47)**

^{1}H NMR

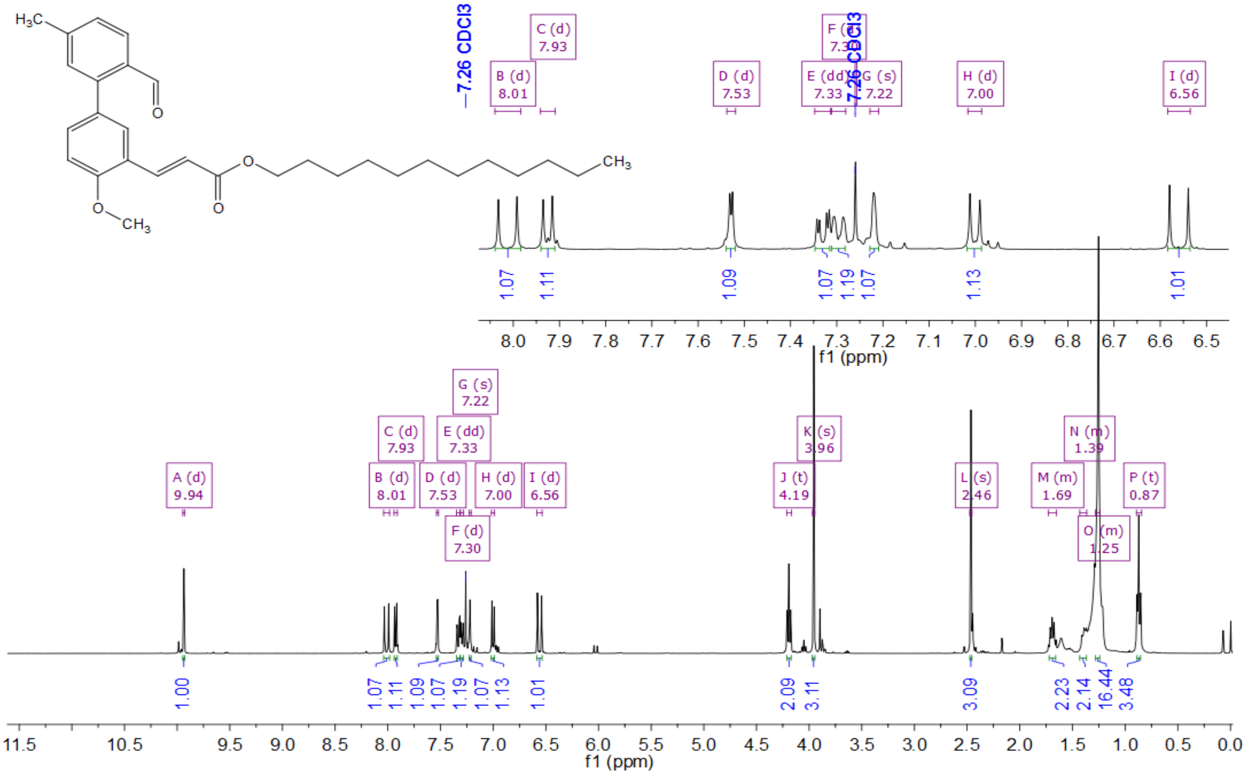

^{13}C NMR

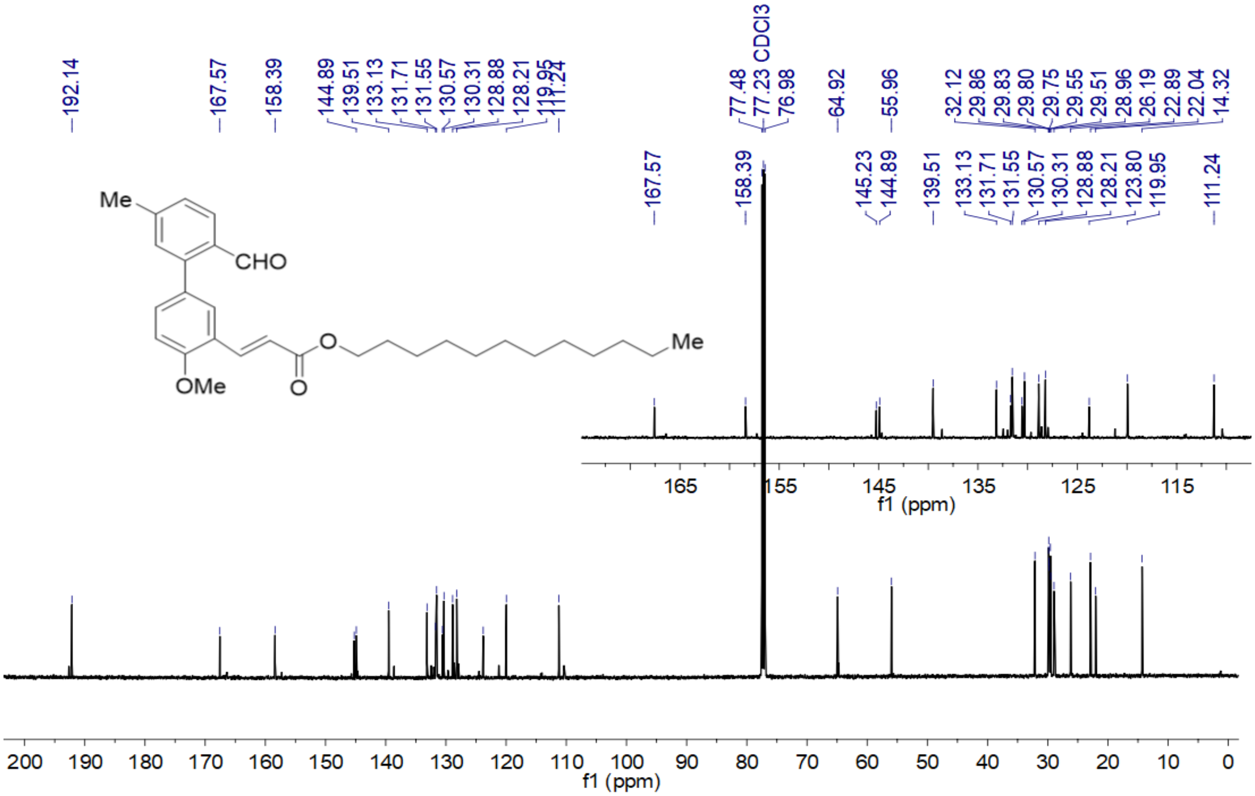

(E)-4-methoxy-3'-methyl-5'-(3-oxobut-1-en-1-yl)-[1,1'-biphenyl]-2-carbaldehyde (**48**)

^{1}H NMR

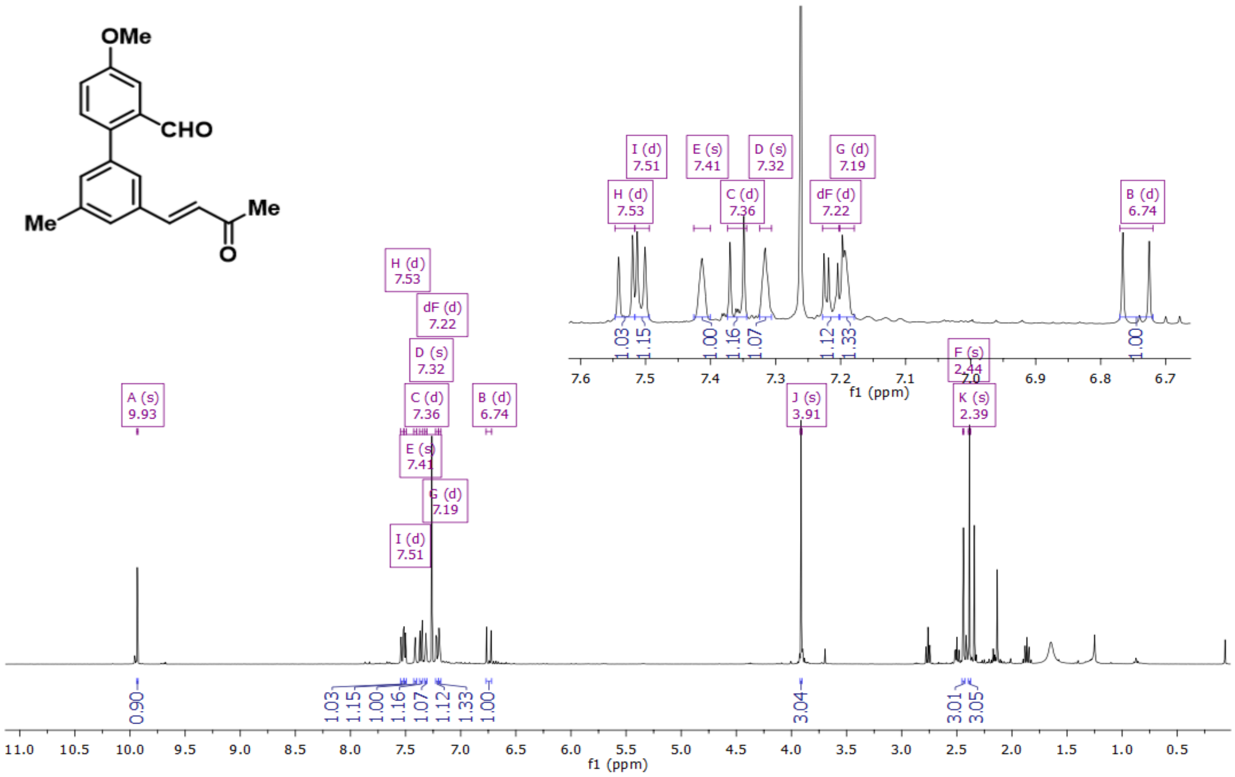

^{13}C NMR

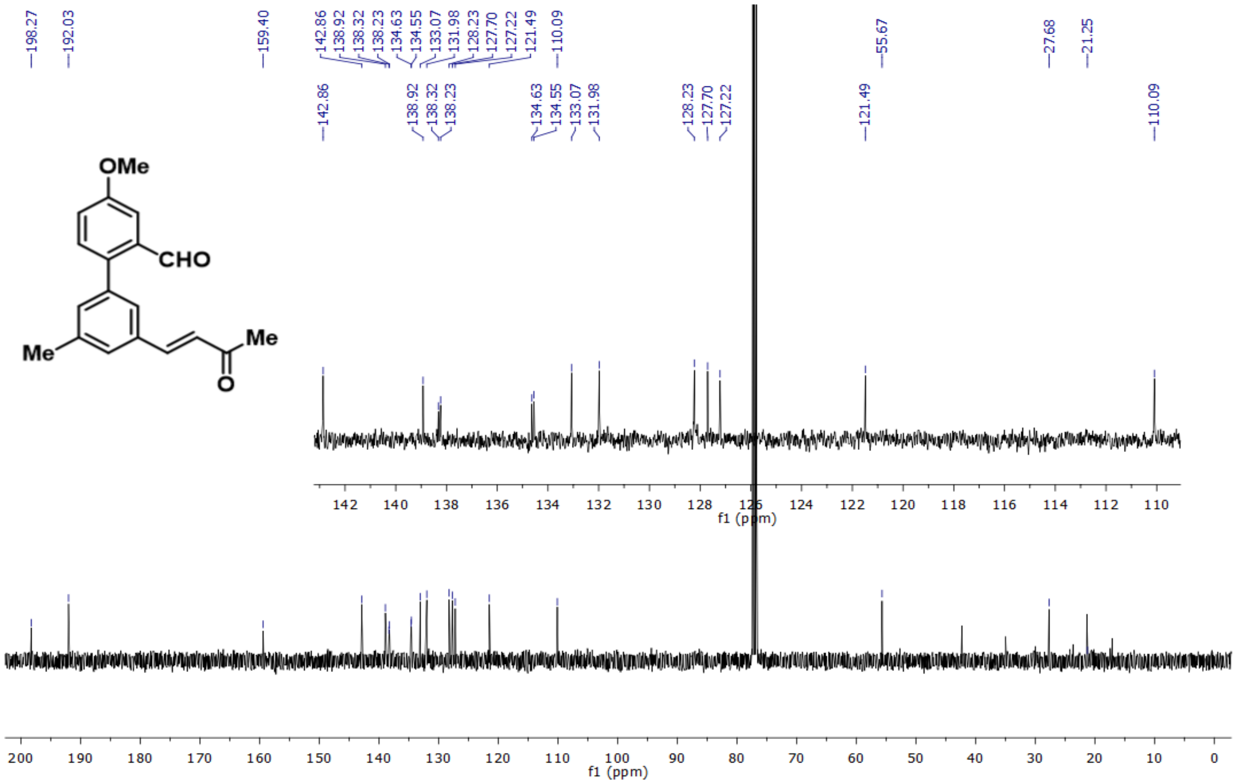

***Ethyl (E)-3-(2'-formyl-4,4',5'-trimethoxy-[1,1'-biphenyl]-3-yl)acrylate* (49)**

^{1}H NMR

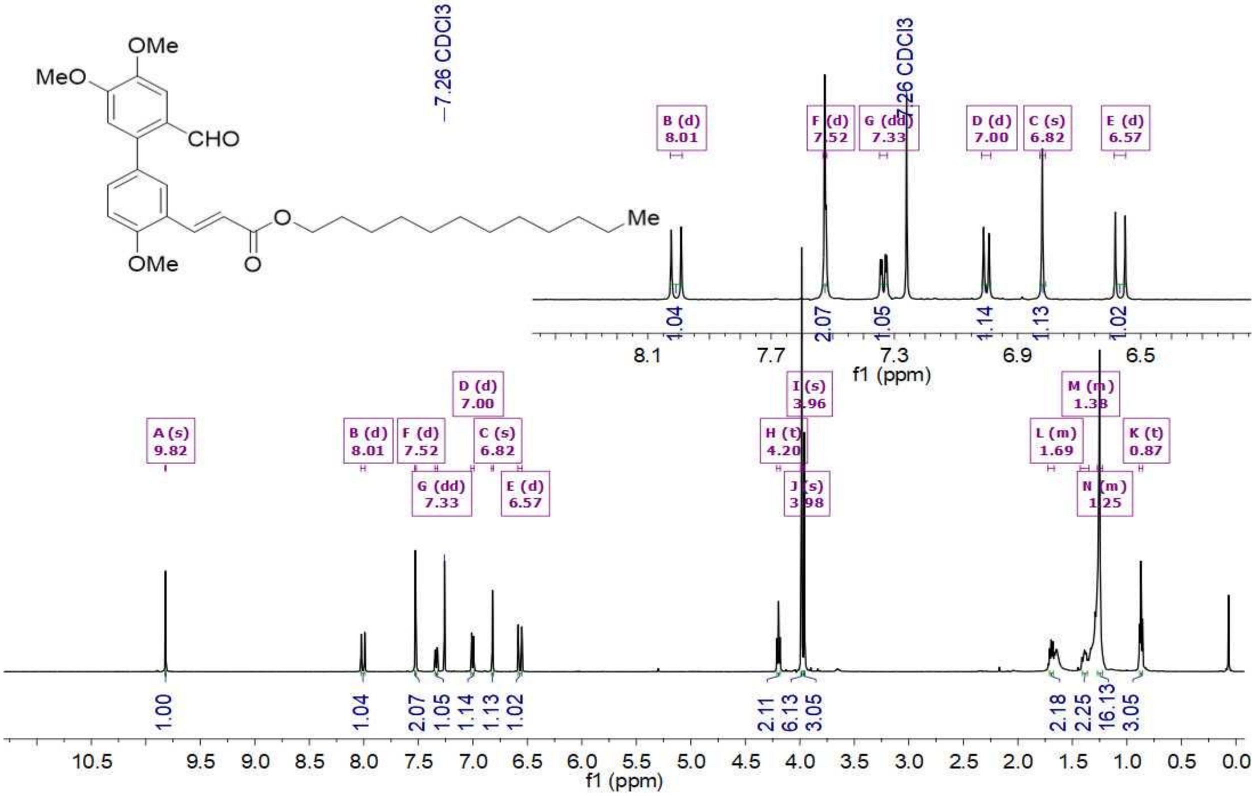

^{13}C NMR

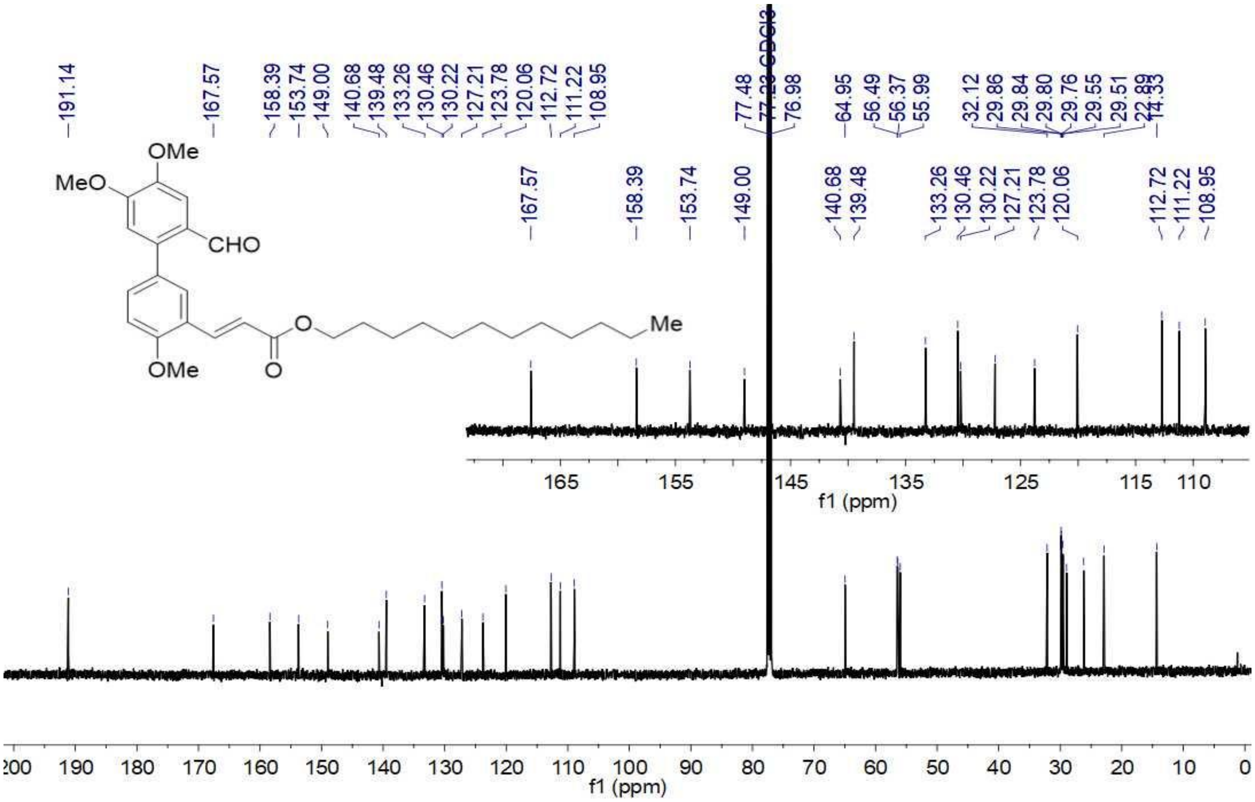

***Dodecyl (E)-3-(6-(2-formyl-5-methoxyphenyl)benzo[d][1,3]dioxol-4-yl)acrylate* (50)**

^{1}H NMR

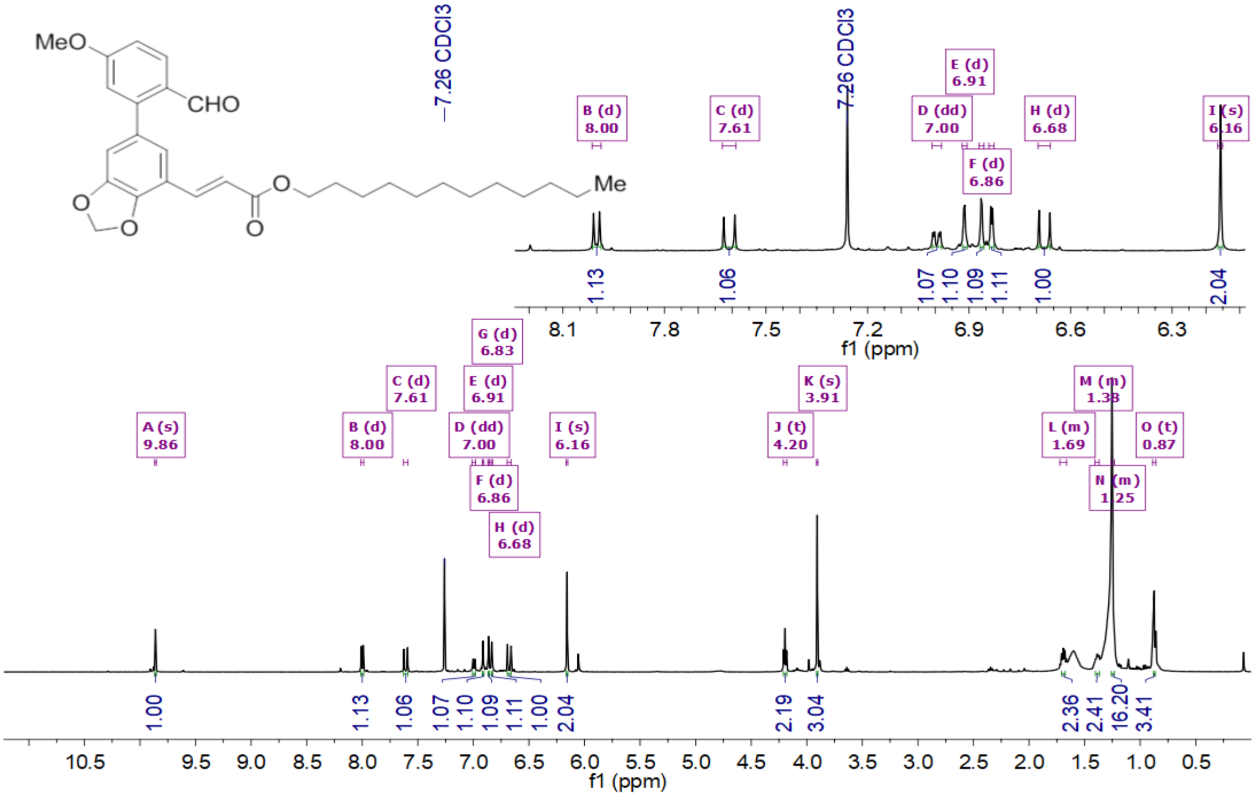

^{13}C NMR

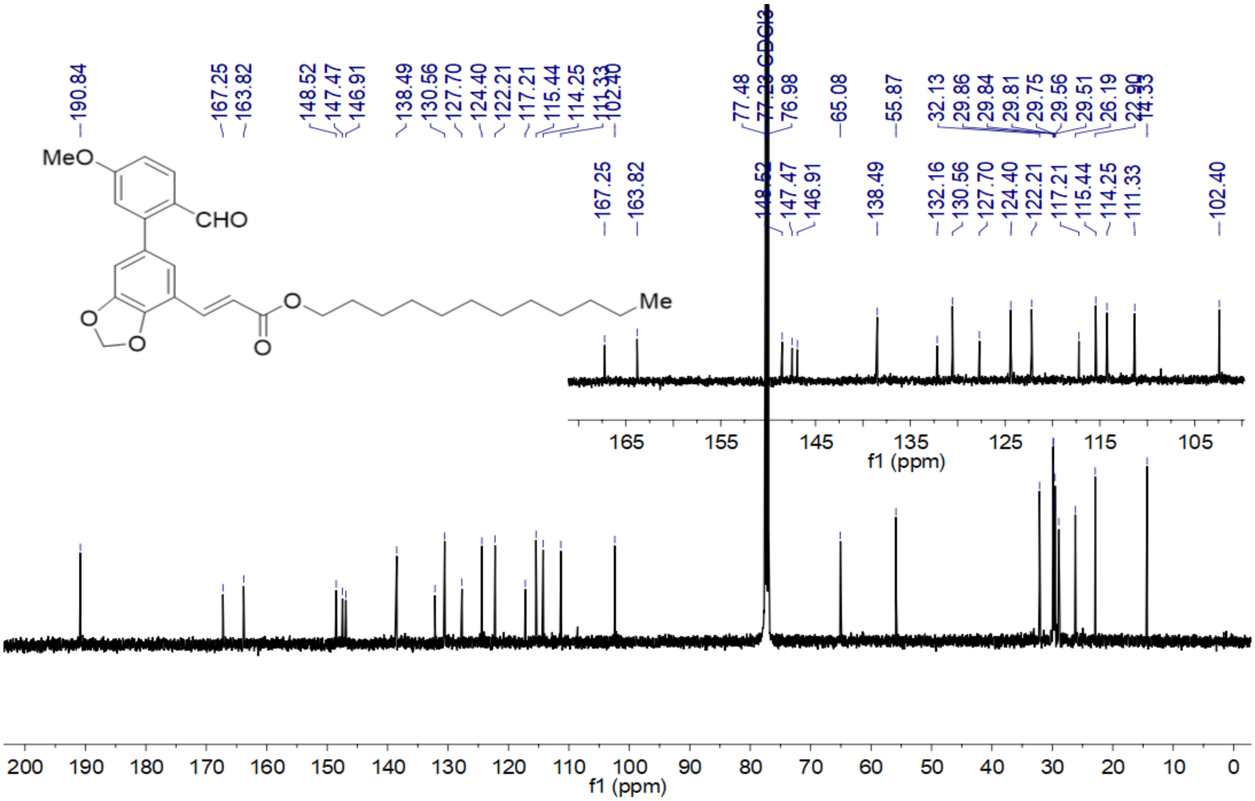

***Ethyl (E)-3-(3-(2-formylnaphthalen-1-yl)phenyl)acrylate* (51)**

^{1}H NMR

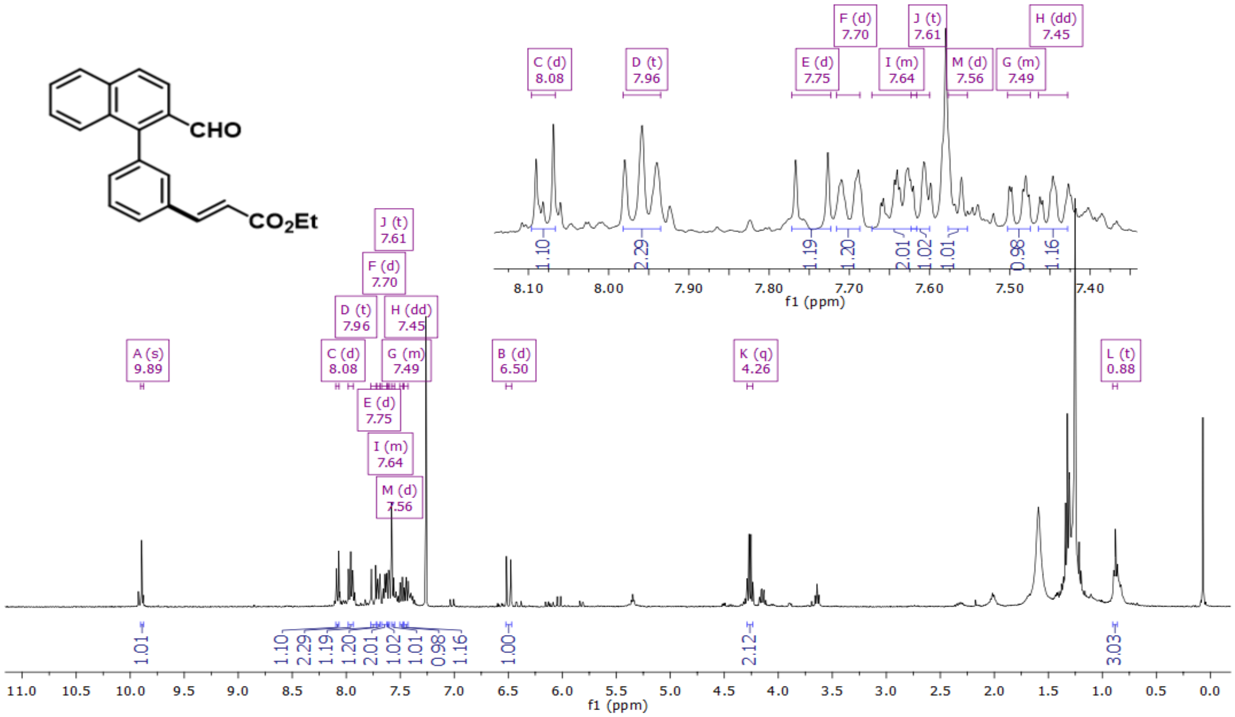

^{13}C NMR

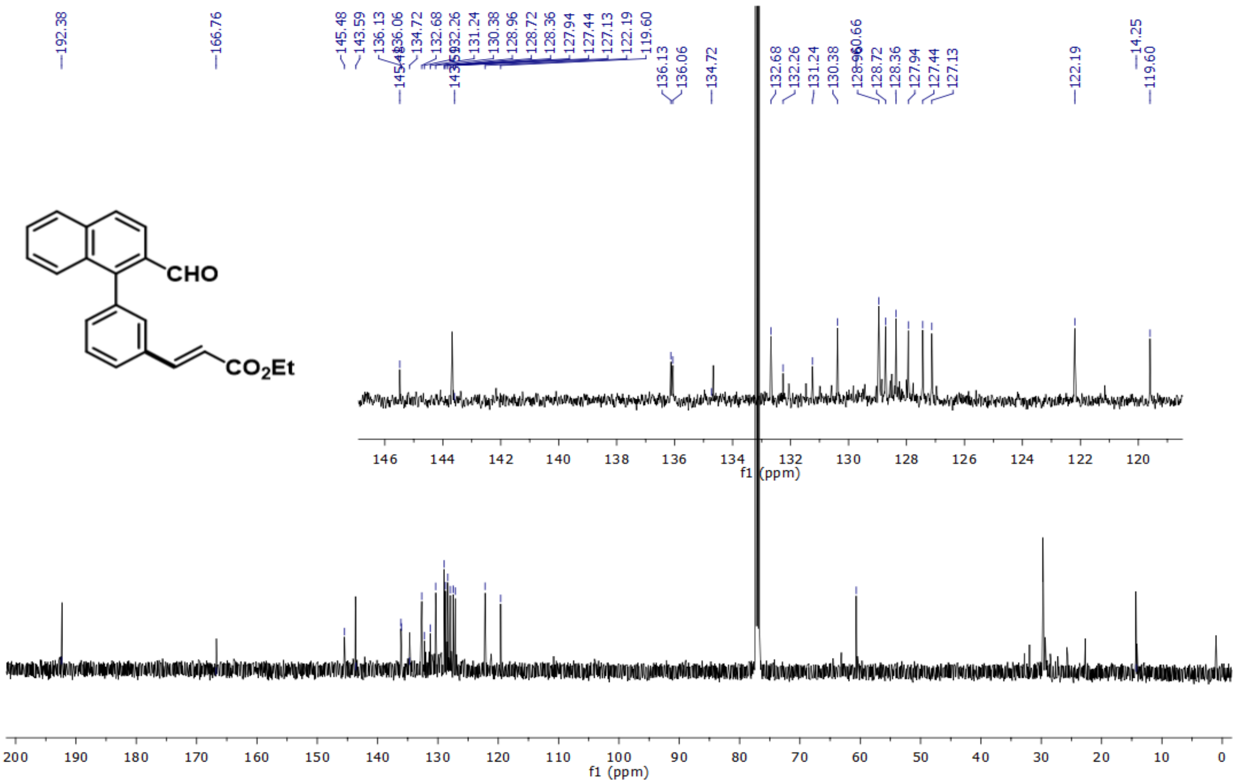

***Ethyl (E)-3-(2'-amino-[1,1'-biphenyl]-3-yl)acrylate* (52)**

^{1}H NMR

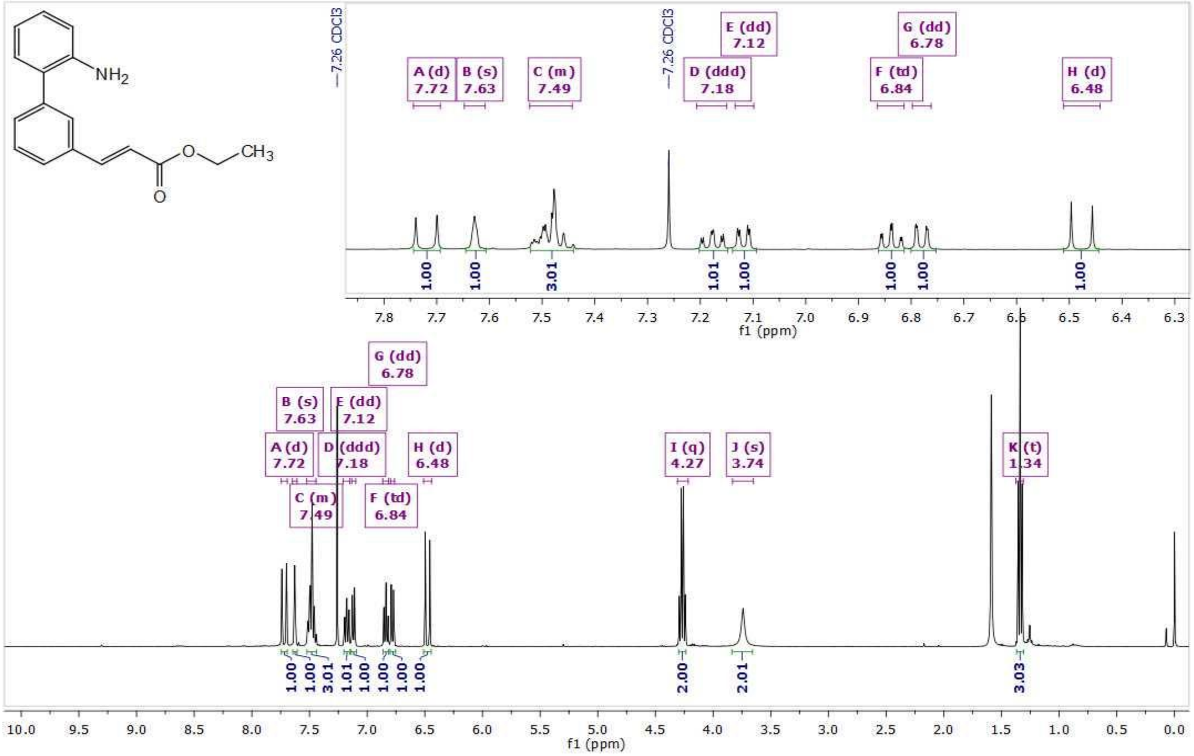

^{13}C NMR

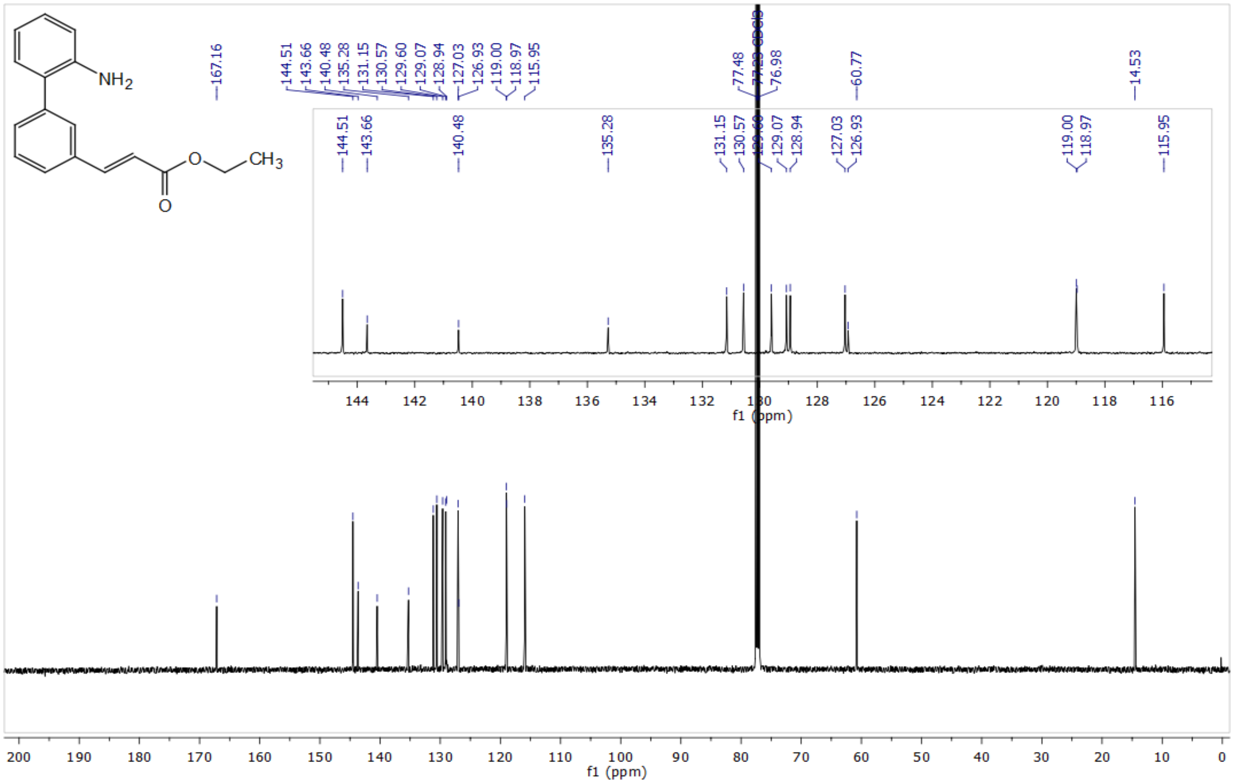

Methyl (E)-3-(2'-amino-[1,1'-biphenyl]-3-yl)acrylate (**53**)

^{1}H NMR

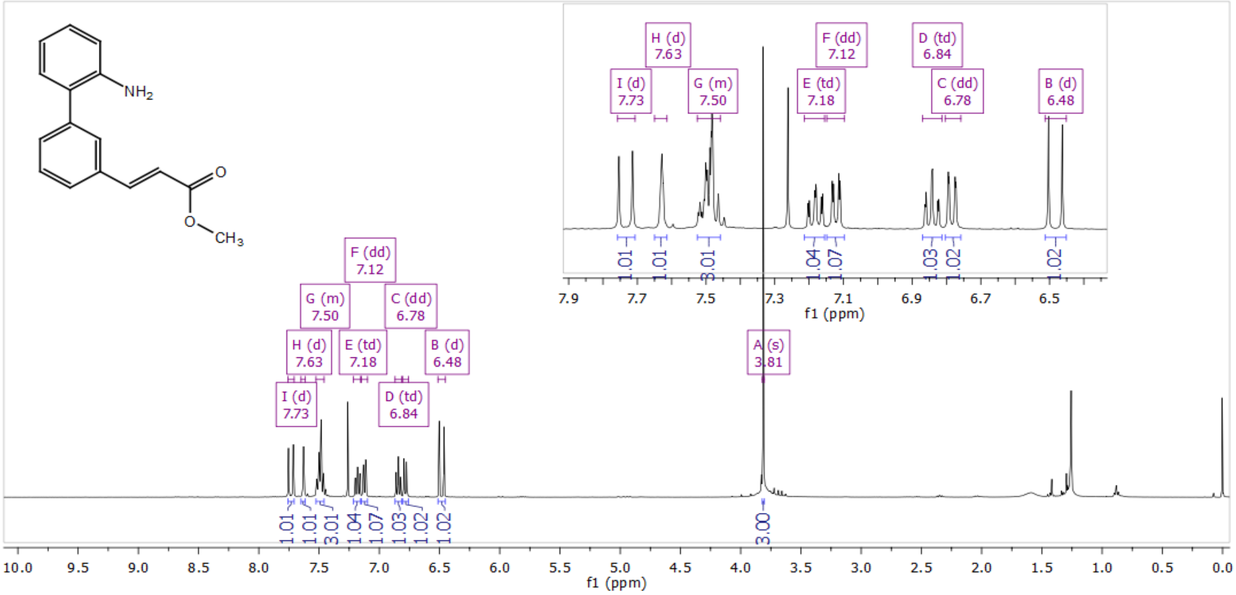

^{13}C NMR

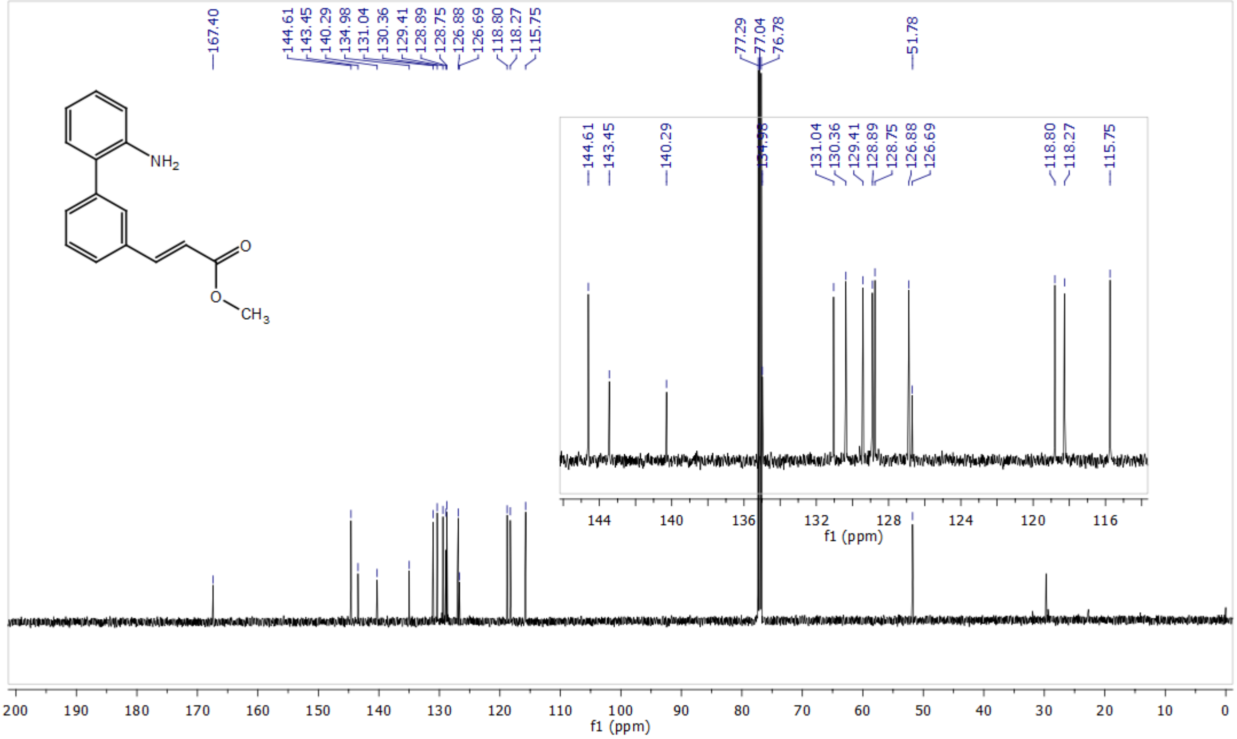

***(E)-3-(2'-amino-[1,1'-biphenyl]-3-yl)acrylonitrile* (54)**

^{1}H NMR

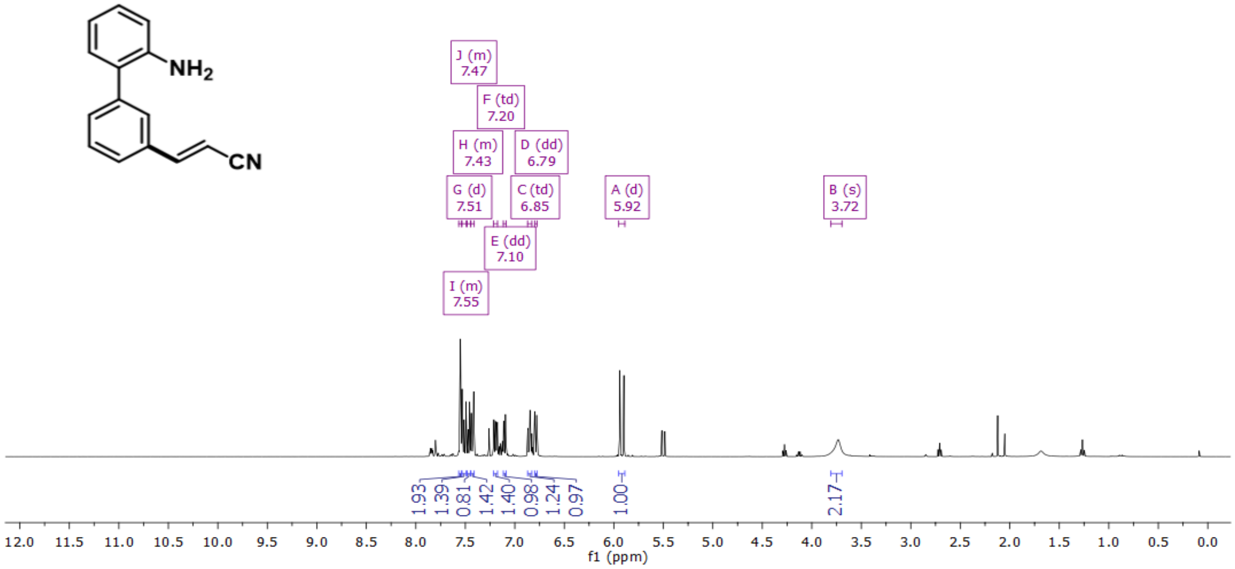

^{13}C NMR

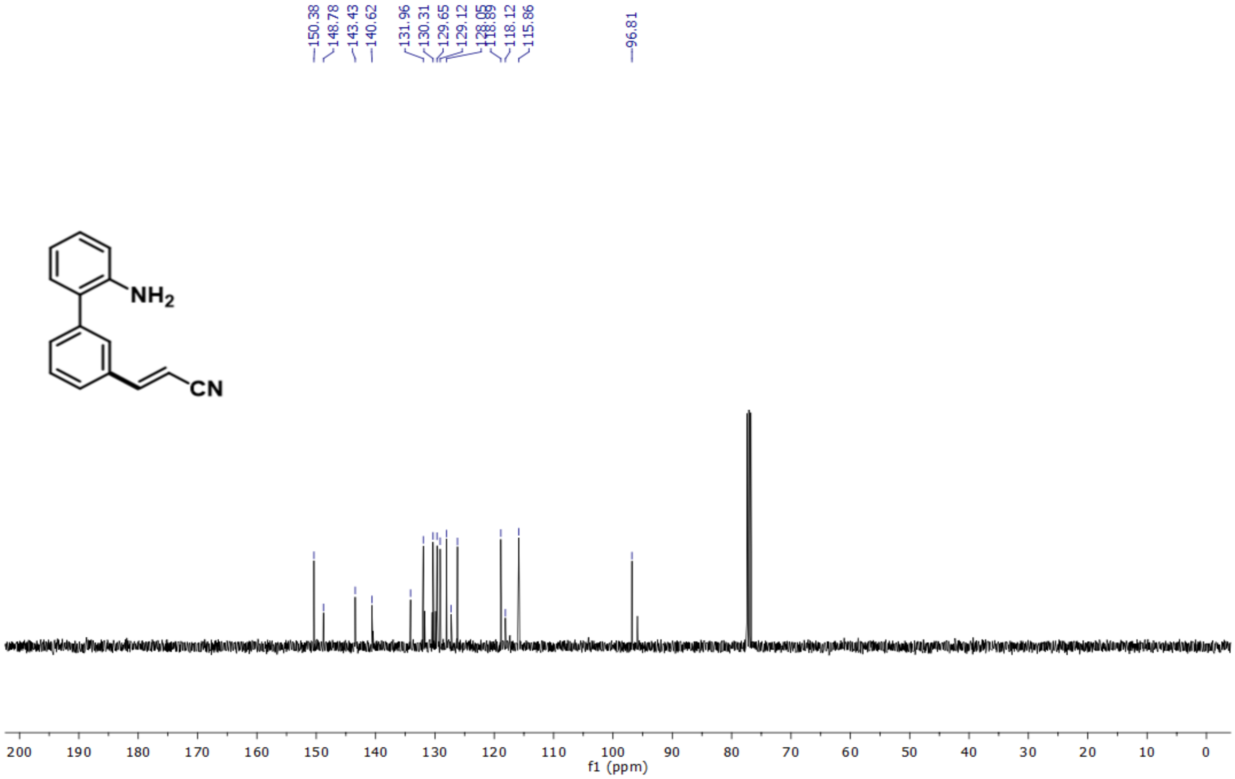

***Ethyl (E)-3-(2'-amino-5-methyl-[1,1'-biphenyl]-3-yl)acrylate* (55)**

^{1}H NMR

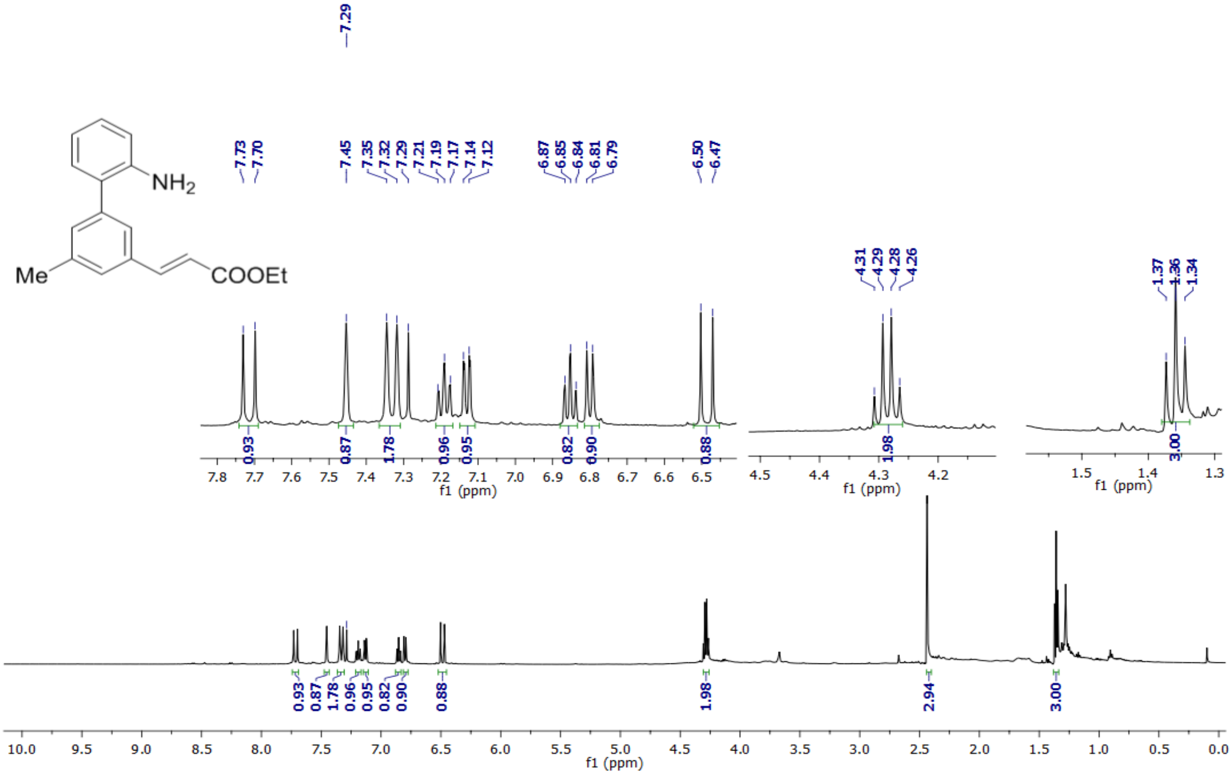

^{13}C NMR

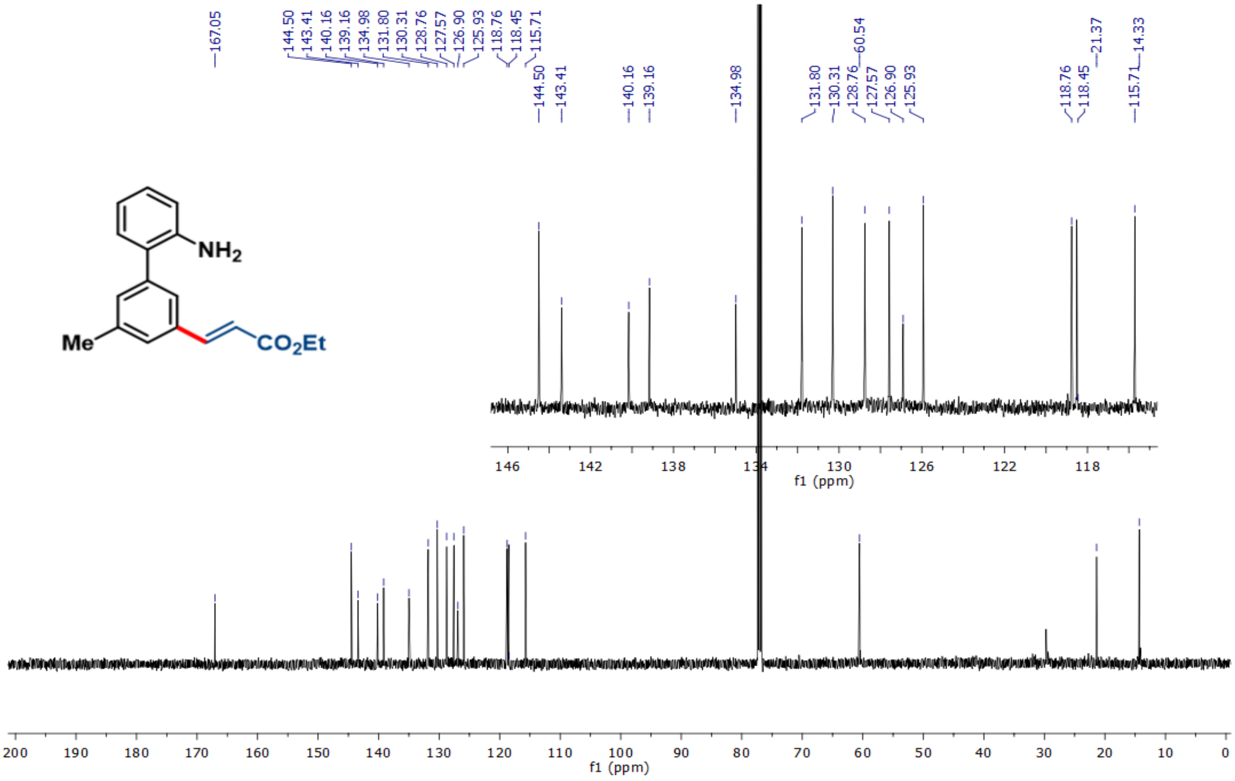

***Methyl (E)-3-(2'-amino-5-methyl-[1,1'-biphenyl]-3-yl)acrylate* (56)**

^{1}H NMR

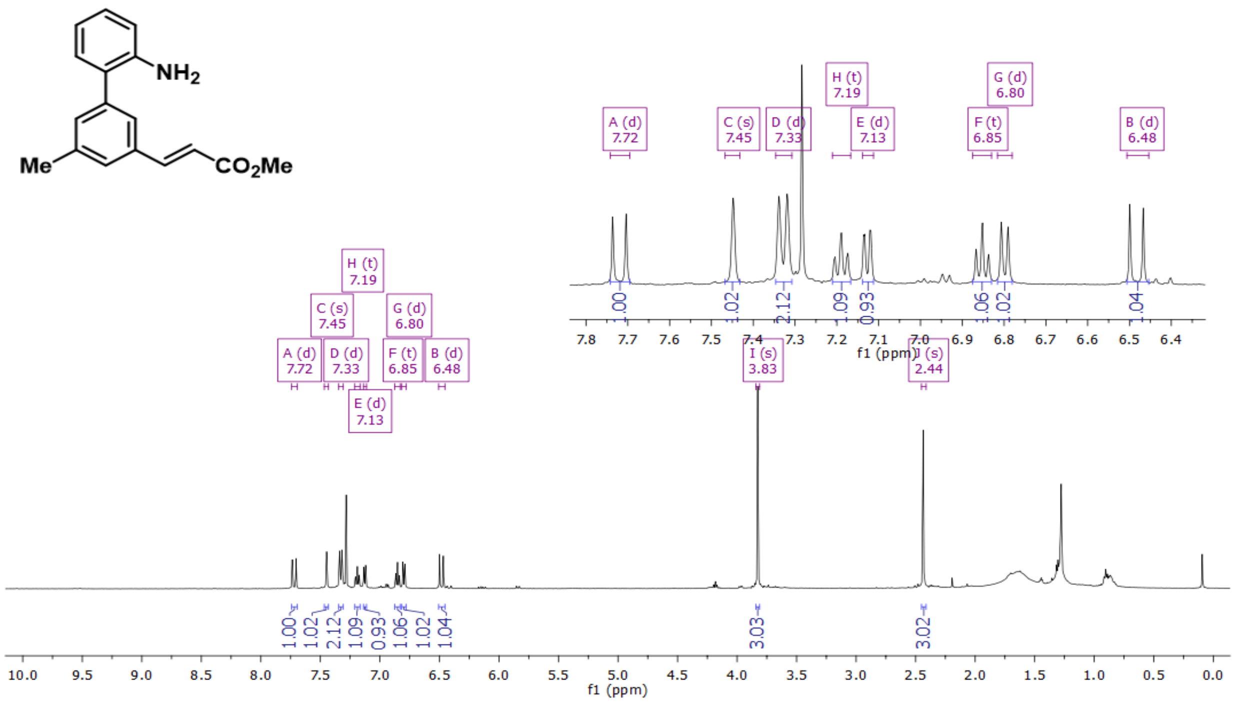

^{13}C NMR

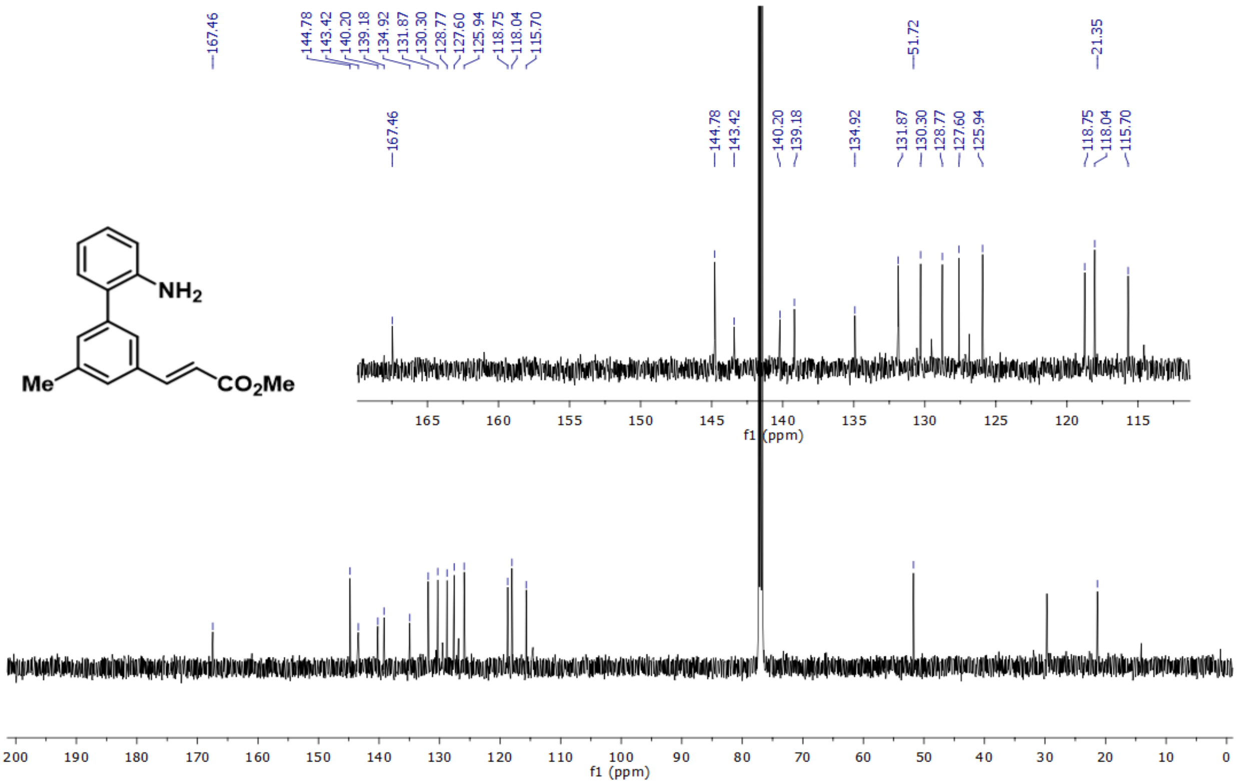

***Ethyl (E)-3-(2'-amino-5-chloro-[1,1'-biphenyl]-3-yl)acrylate* (57)**

^{1}H NMR

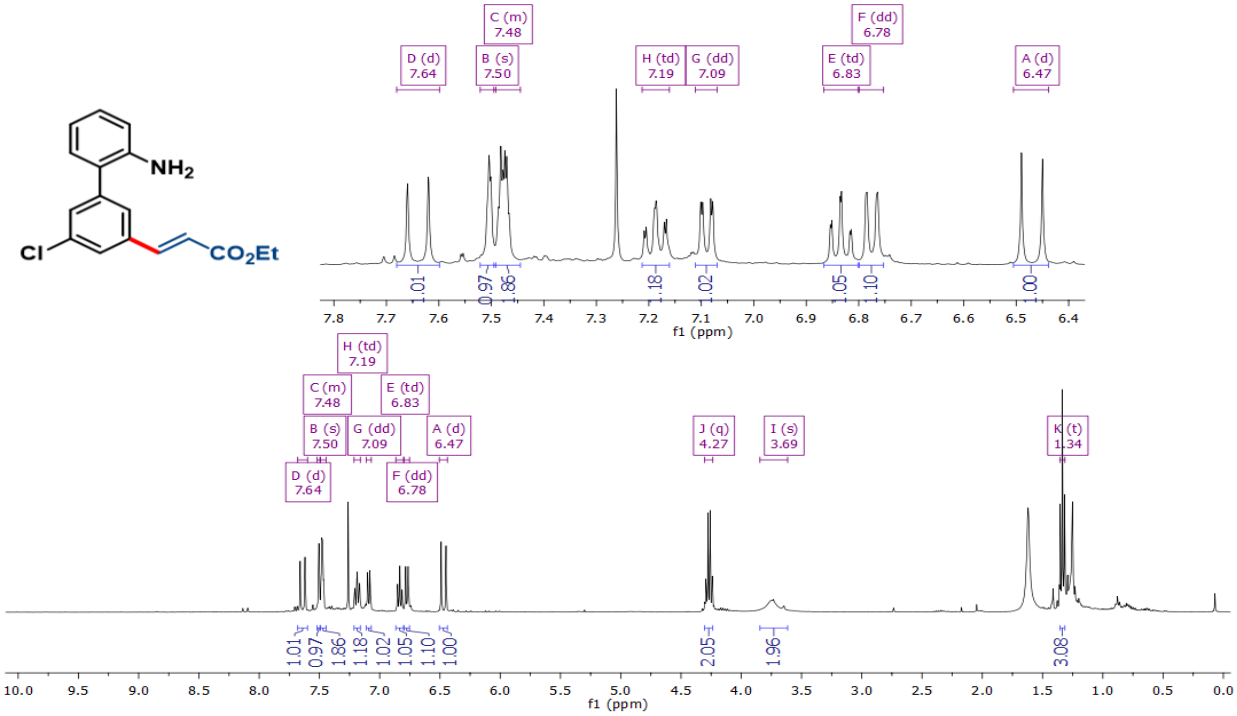

^{13}C NMR

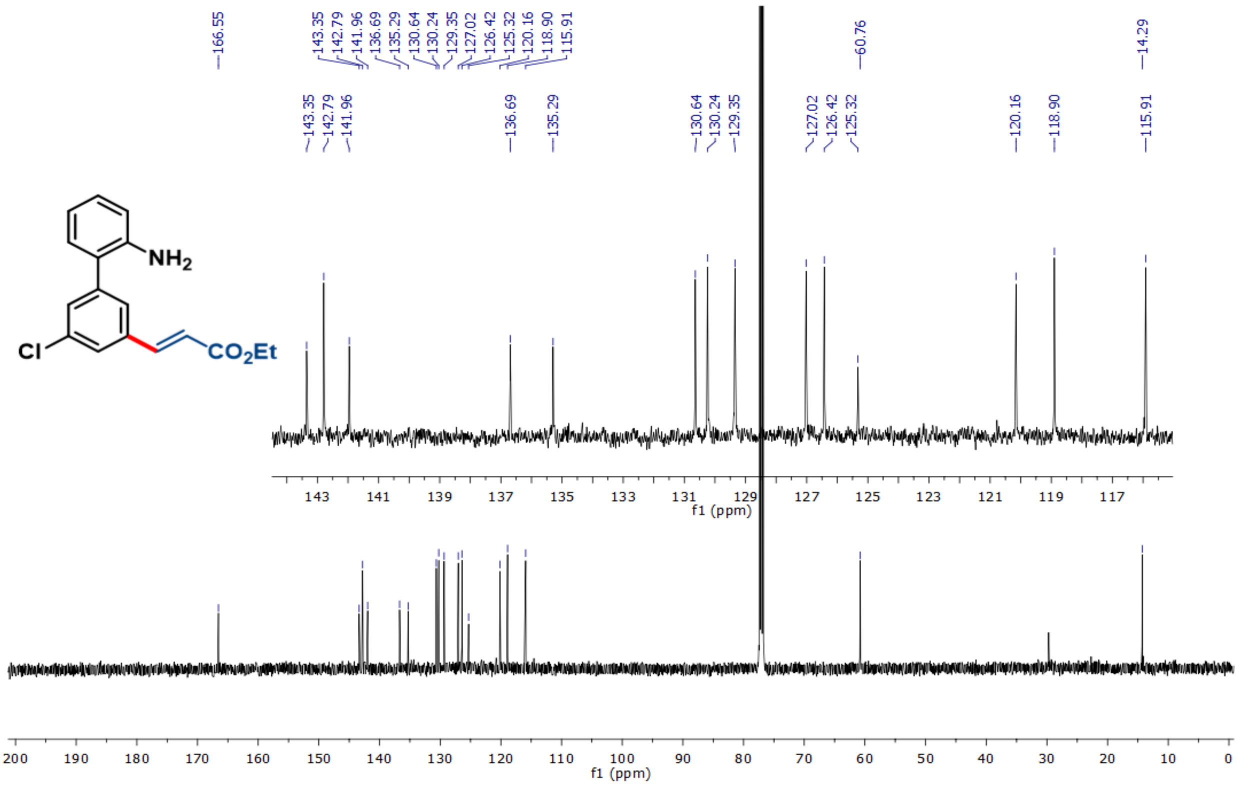

***Butyl (E)-3-(2'-amino-5-chloro-[1,1'-biphenyl]-3-yl)acrylate* (58)**

¹H NMR

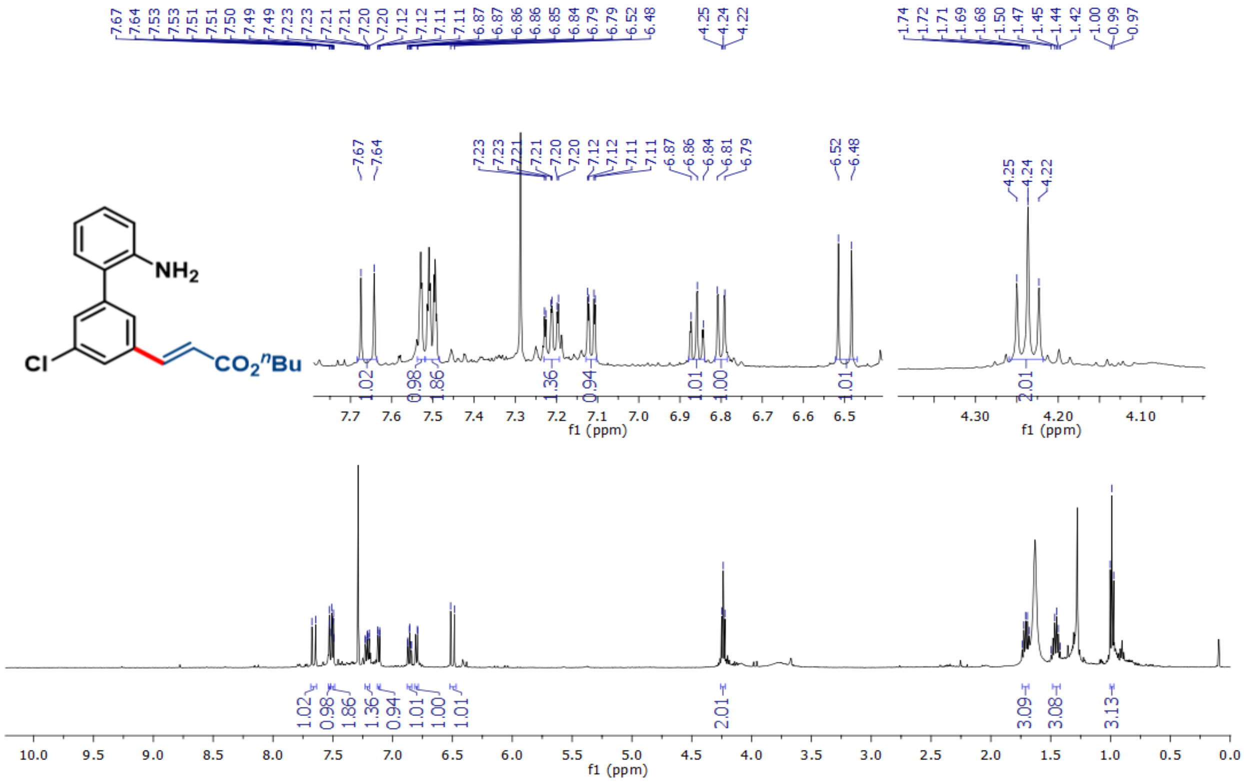

¹³C NMR

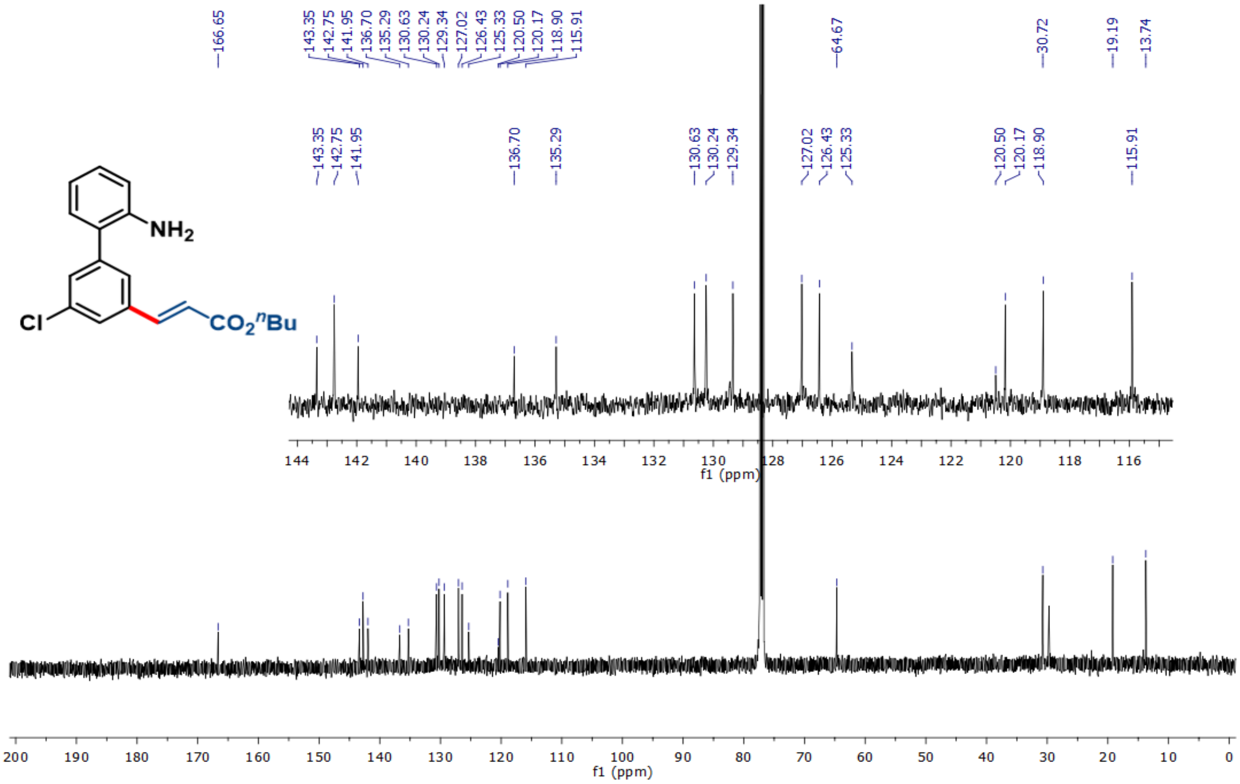

Ethyl (E)-3-(2'-amino-4-fluoro-[1,1'-biphenyl]-3-yl)acrylate (59)

^{1}H NMR

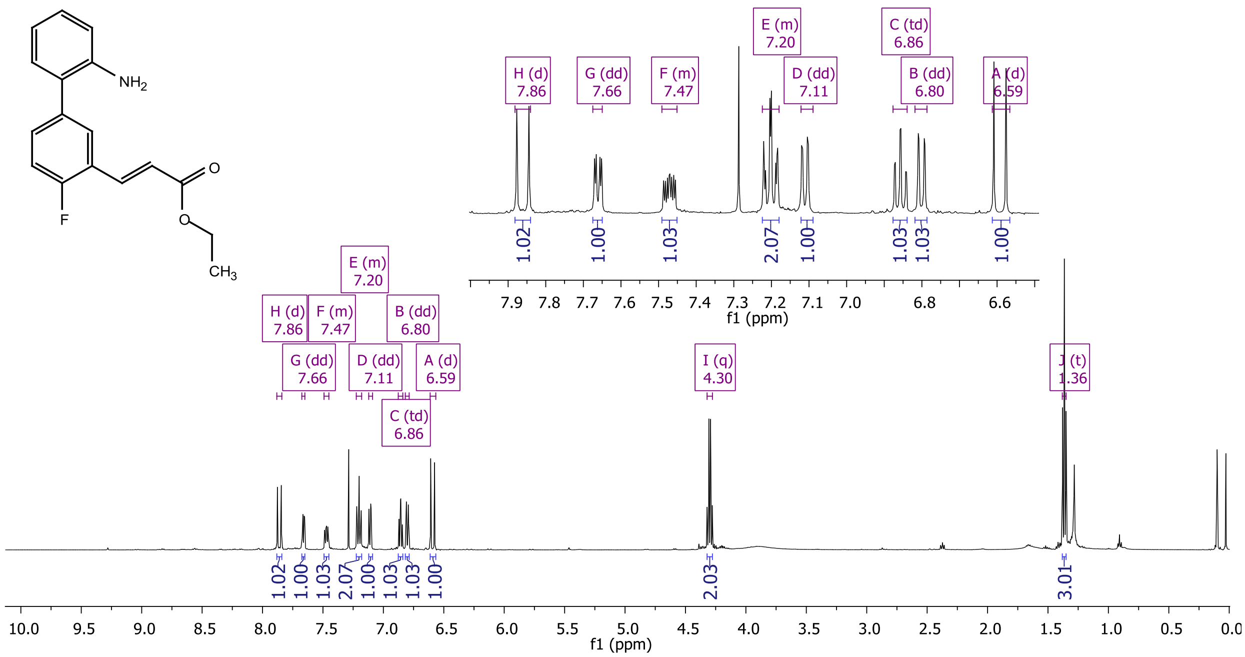

^{13}C NMR

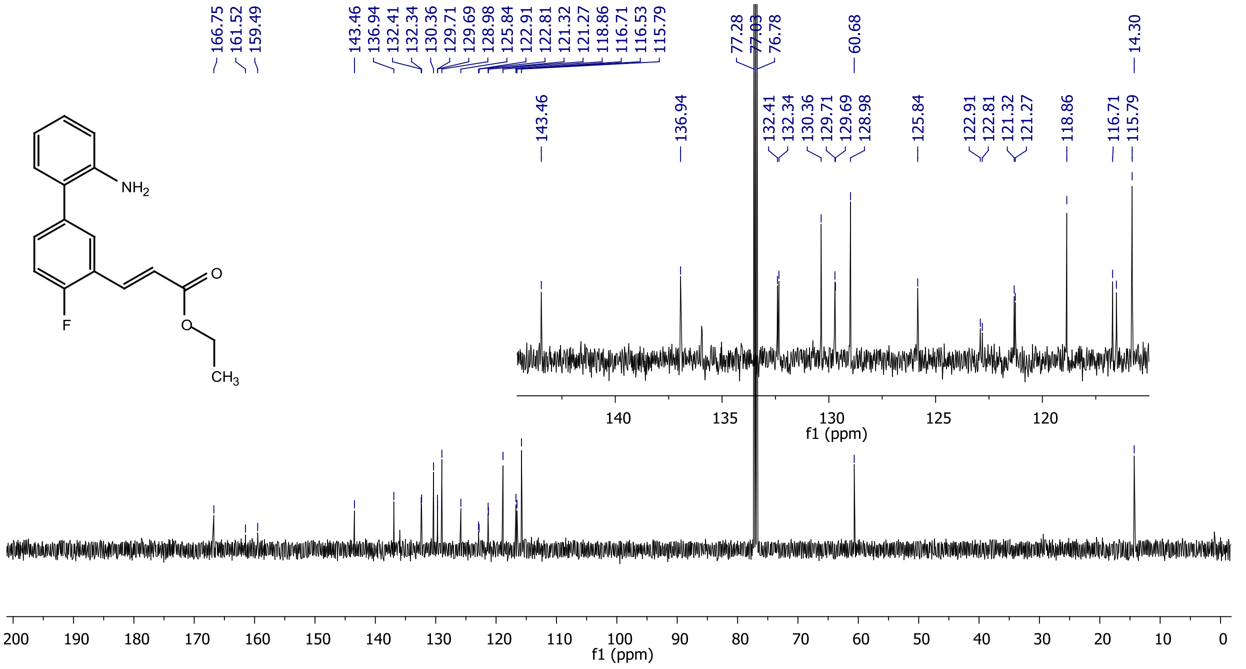

^{19}F NMR

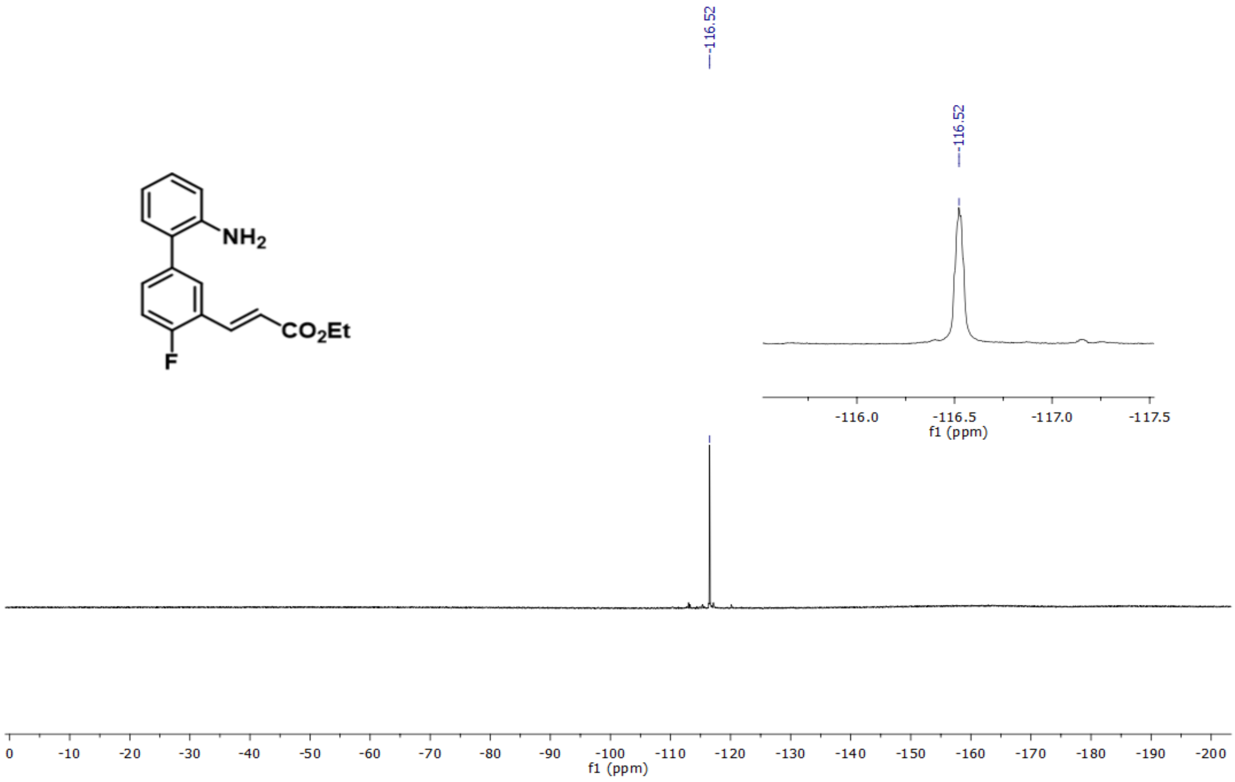
NH2
CO2Et
F
-116.52
-116.52
-116.0
-116.5
-117.0
-117.5
f1 (ppm)
0
-10
-20
-30
-40
-50
-60
-70
-80
-90
-100
-110
-120
-130
-140
-150
-160
-170
-180
-190
-200
f1 (ppm)

Ethyl (E)-3-(2'-amino-4-fluoro-5-methyl-[1,1'-biphenyl]-3-yl)but-2-enoate (**60**)

^{1}H NMR

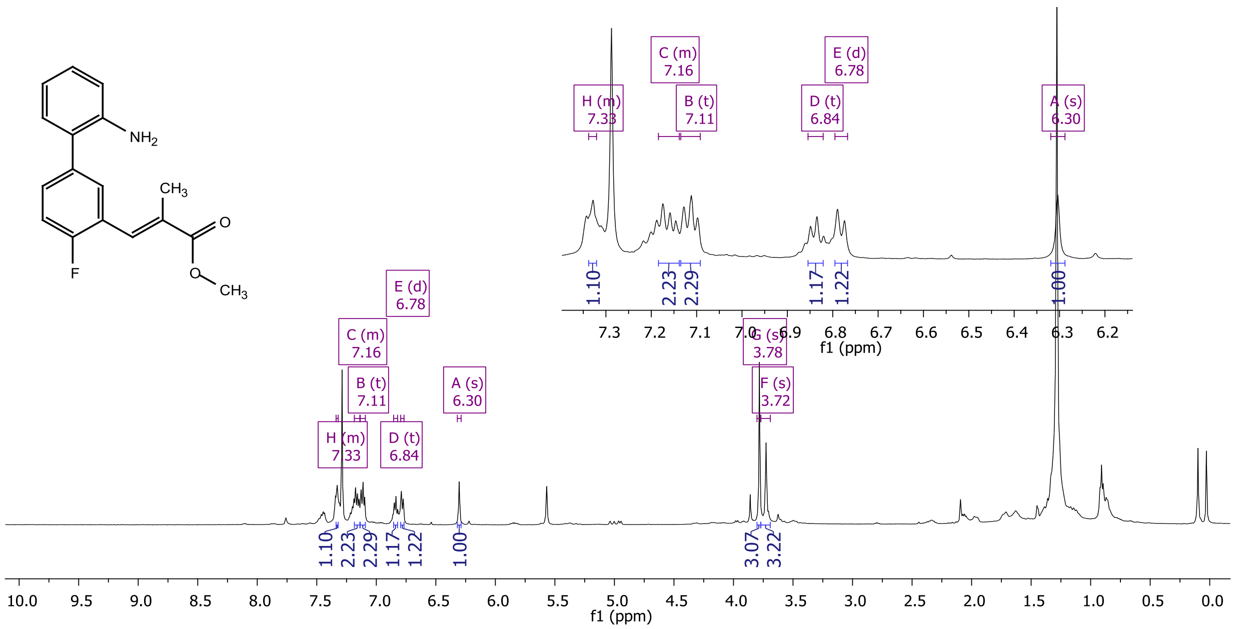

^{13}C NMR

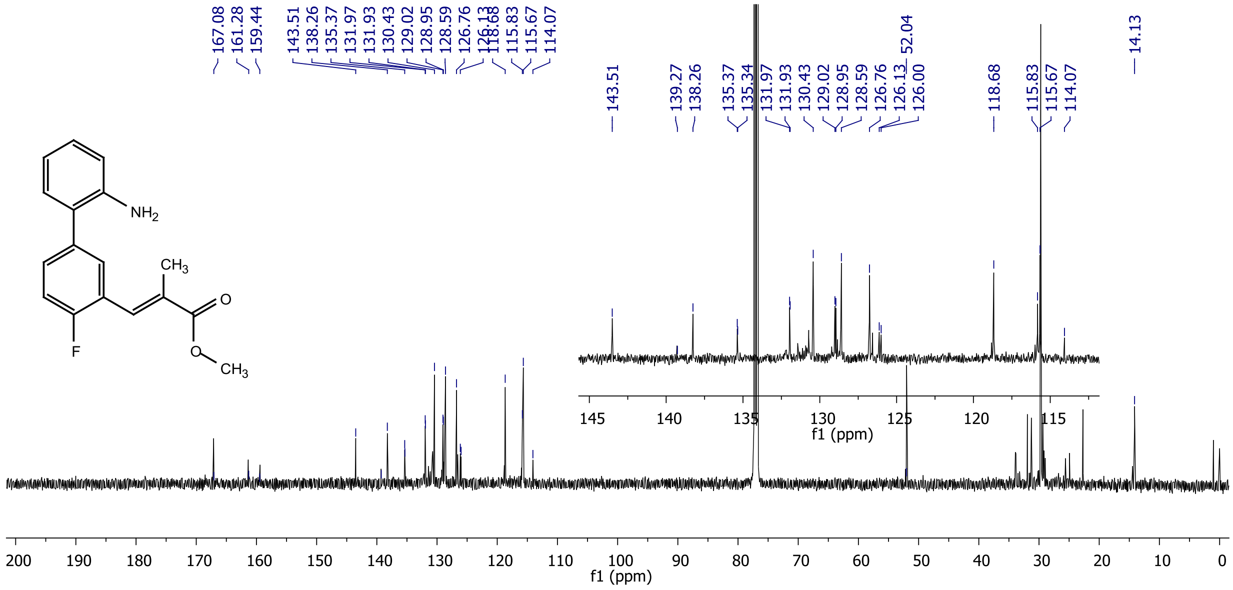

^{19}F NMR

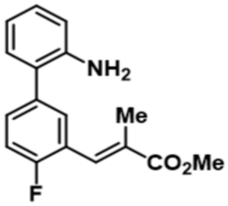

NH2
Me
CO2Me
F

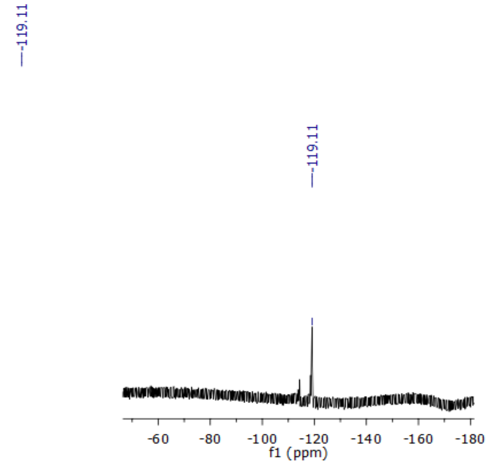

-119.11
-119.11
-60 -80 -100 -120 -140 -160 -180
f1 (ppm)

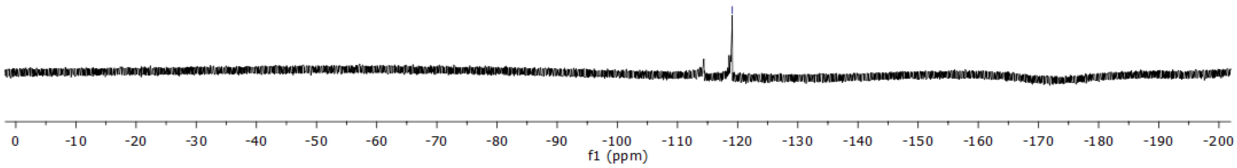

0 -10 -20 -30 -40 -50 -60 -70 -80 -90 -100 -110 -120 -130 -140 -150 -160 -170 -180 -190 -200
f1 (ppm)

***Ethyl (E)-3-(2'-amino-6-fluoro-[1,1'-biphenyl]-3-yl)acrylate* (61)**

^{1}H NMR

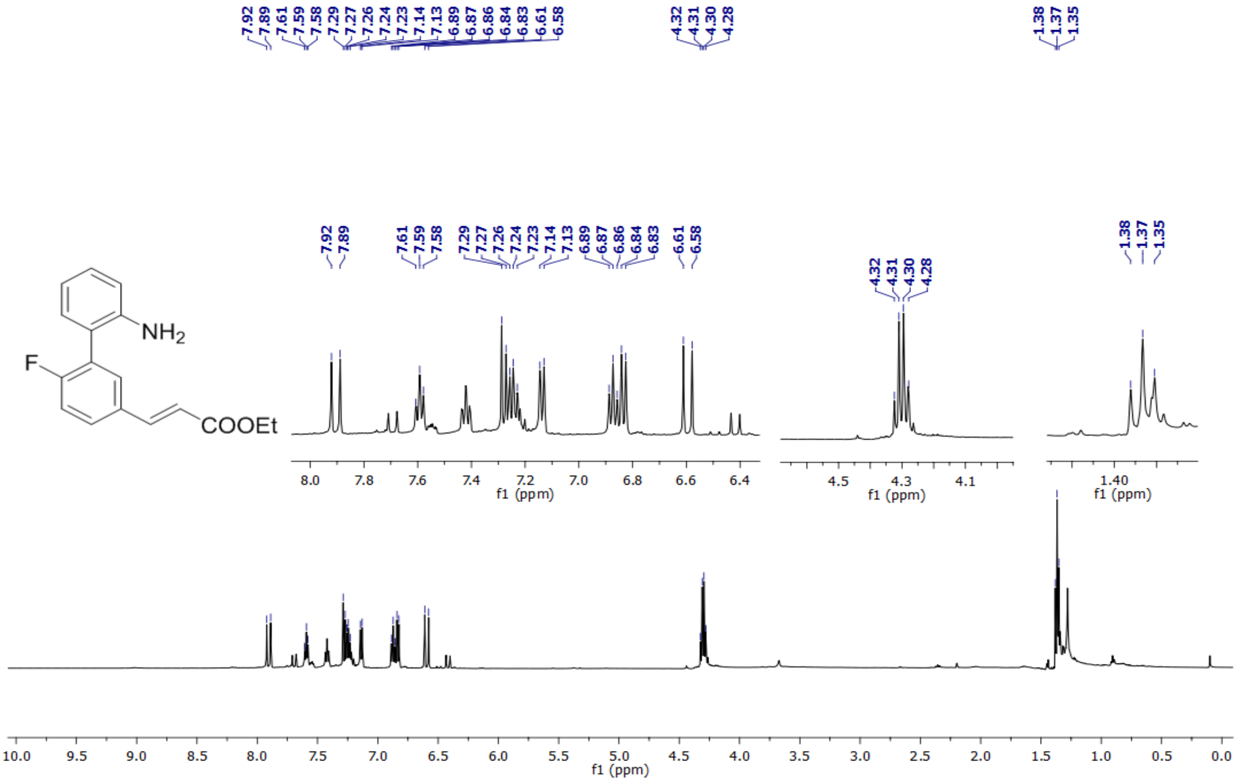

^{13}C NMR

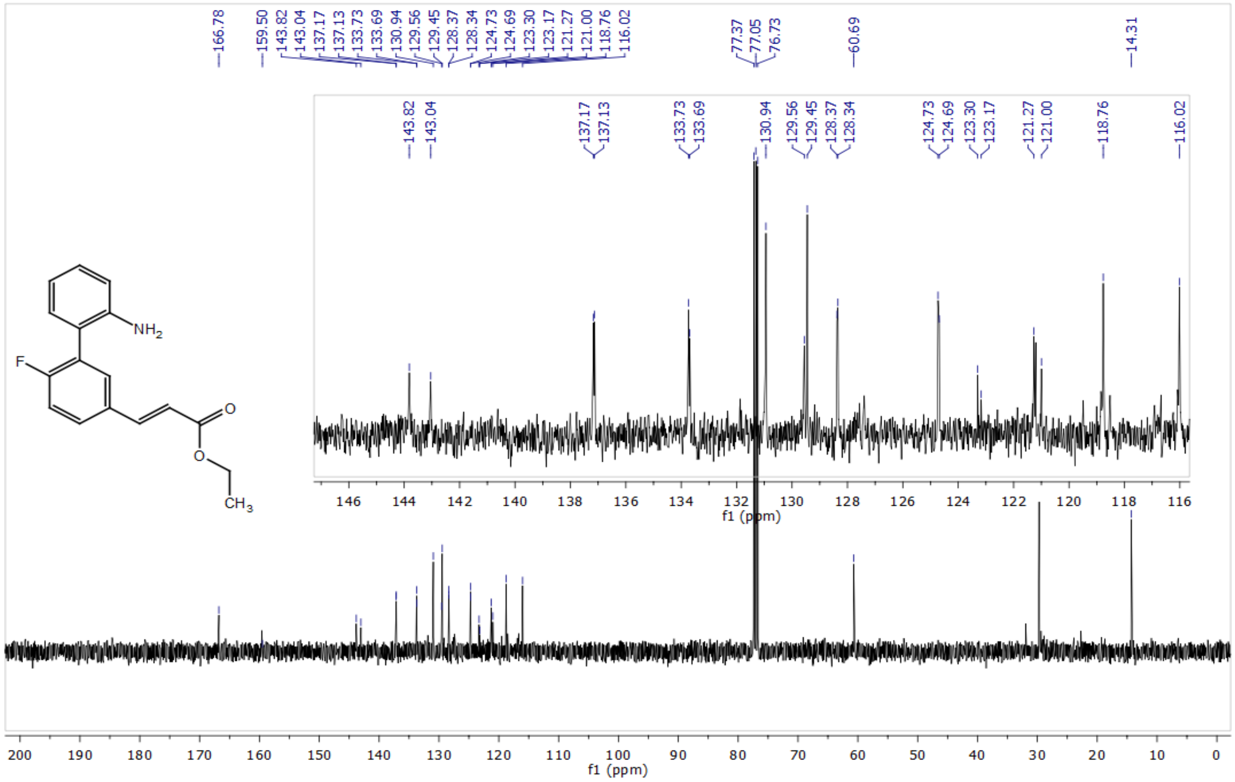

^{19}F NMR

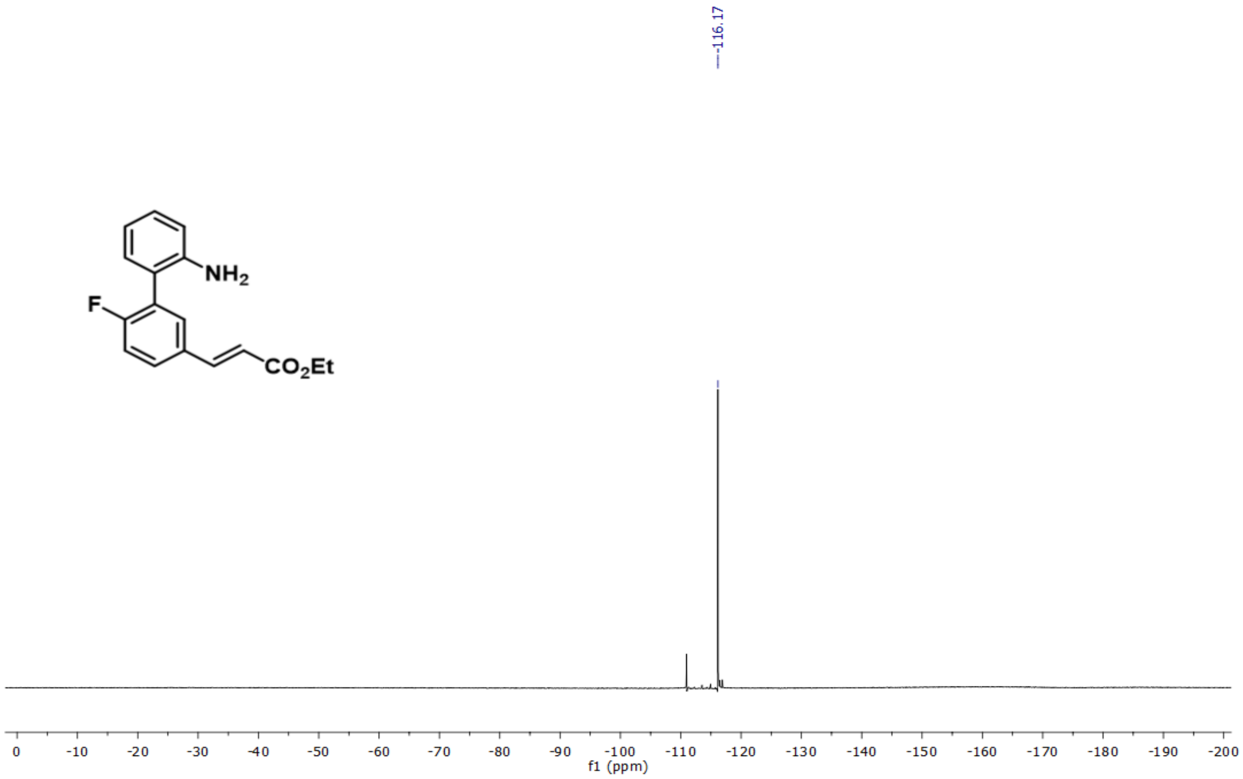

-116.17
NH2
F
CO2Et
0 -10 -20 -30 -40 -50 -60 -70 -80 -90 -100 -110 -120 -130 -140 -150 -160 -170 -180 -190 -200
f1 (ppm)

***Ethyl (E)-3-(2'-amino-4-fluoro-5-methyl-[1,1'-biphenyl]-3-yl)acrylate* (62)**

^{1}H NMR

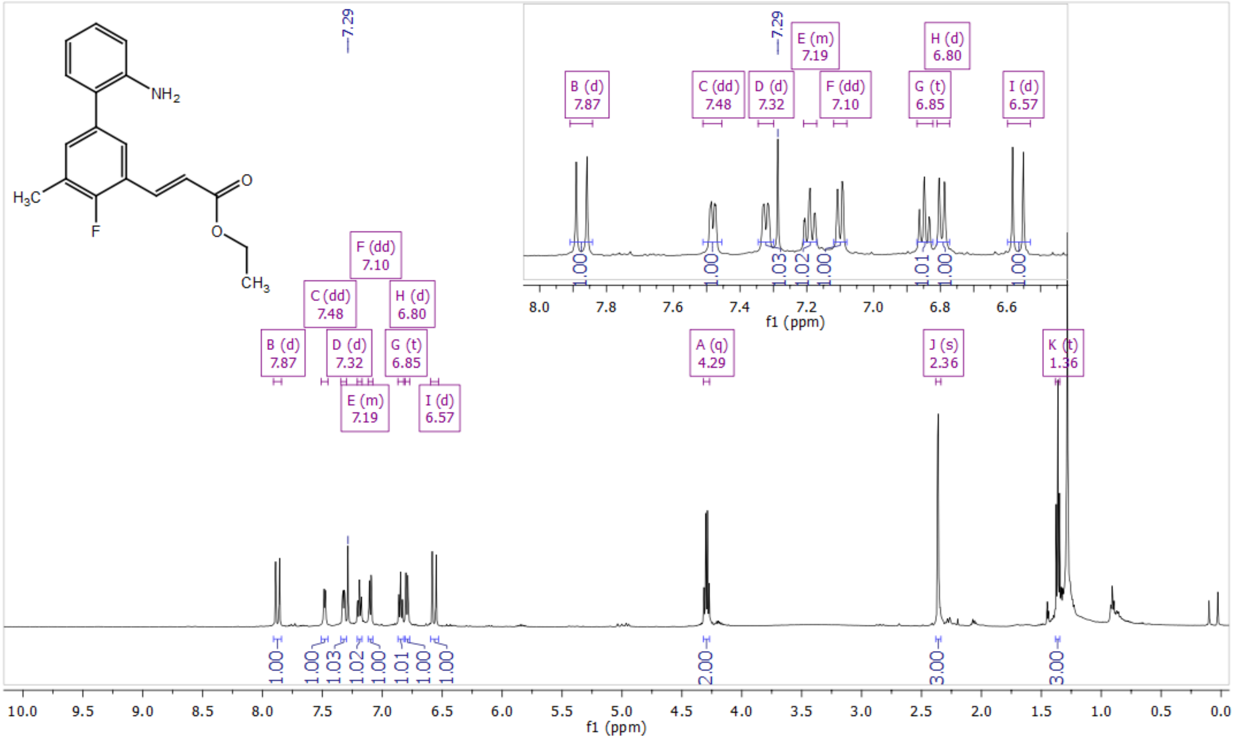

^{13}C NMR

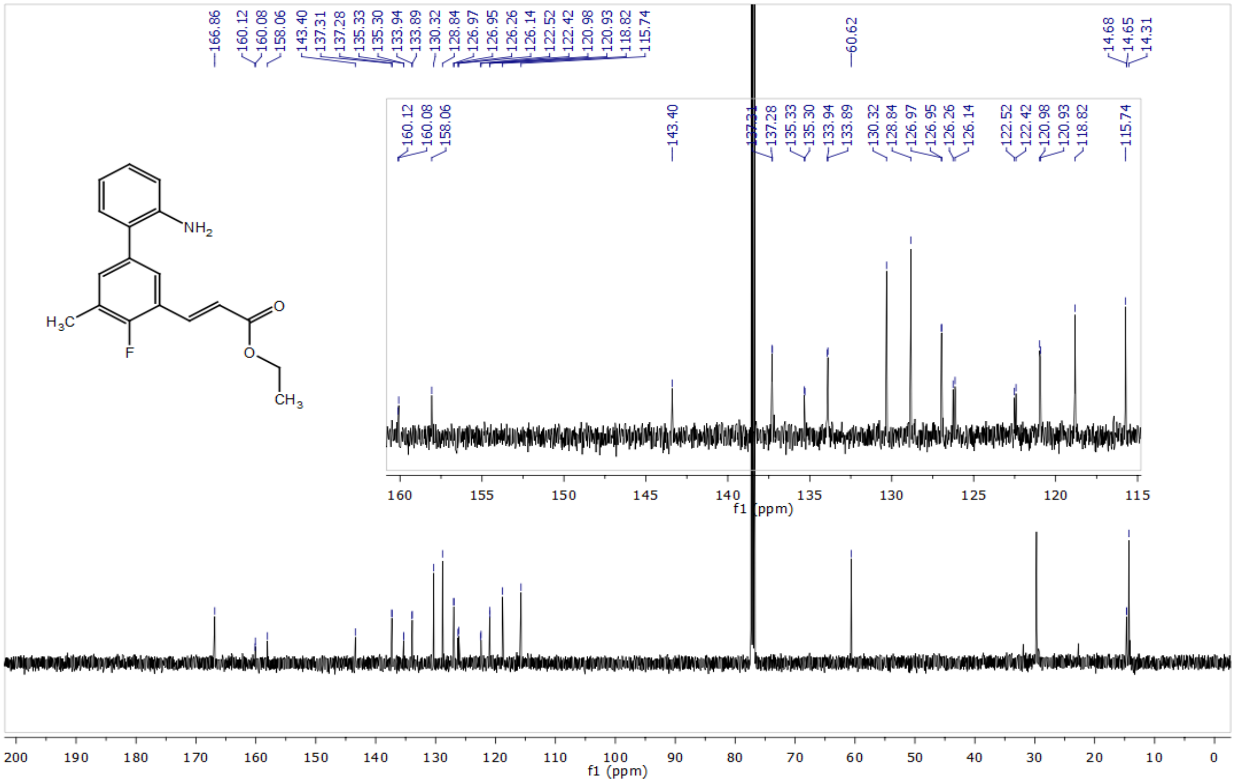

^{19}F NMR

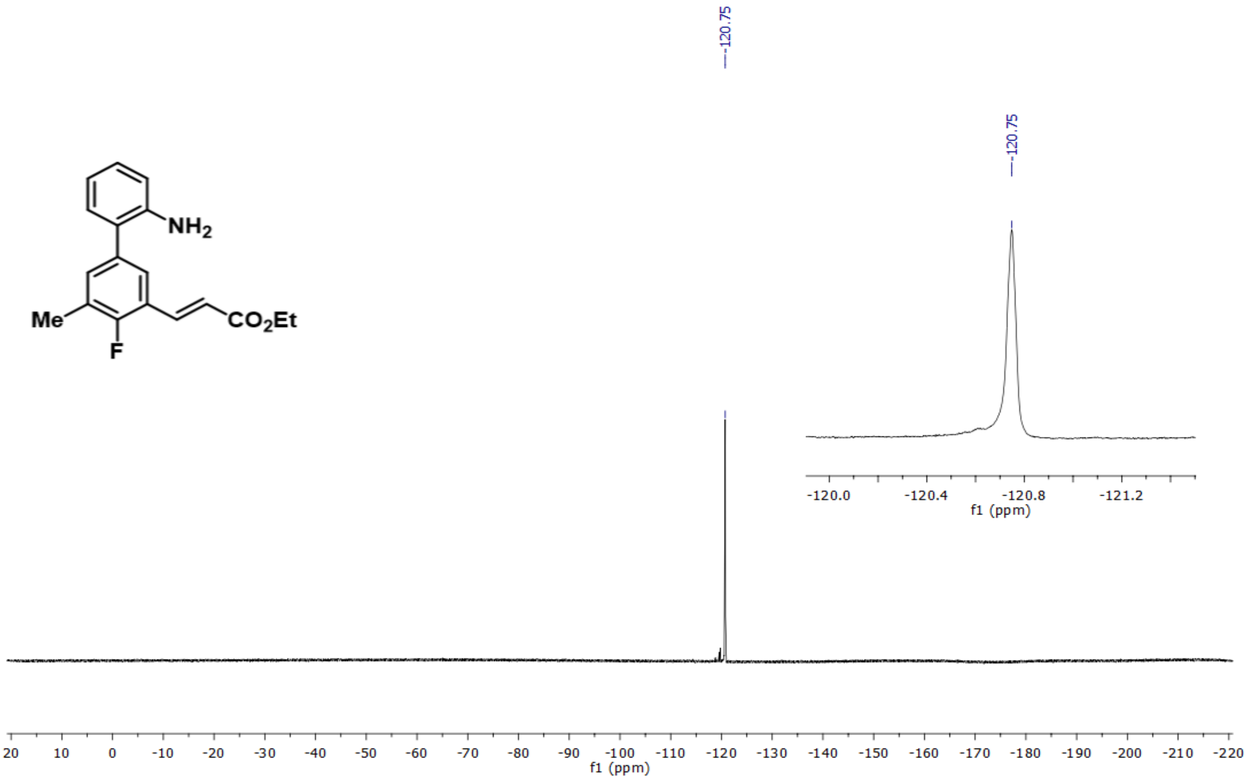

-120.75
-120.75
-120.0
-120.4
-120.8
-121.2
f1 (ppm)
20
10
0
-10
-20
-30
-40
-50
-60
-70
-80
-90
-100
-110
-120
-130
-140
-150
-160
-170
-180
-190
-200
-210
-220
f1 (ppm)

***Ethyl (E)-3-(2'-amino-4-fluoro-5-methyl-[1,1'-biphenyl]-3-yl)but-2-enoate* (63)**

^{1}H NMR

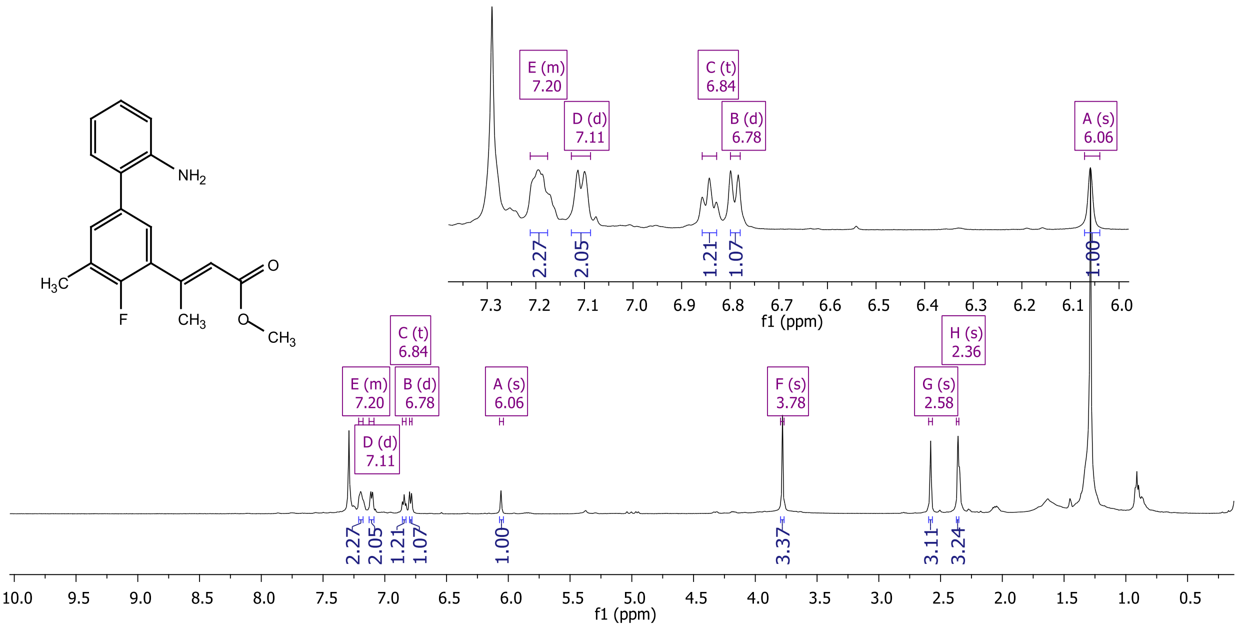

^{13}C NMR

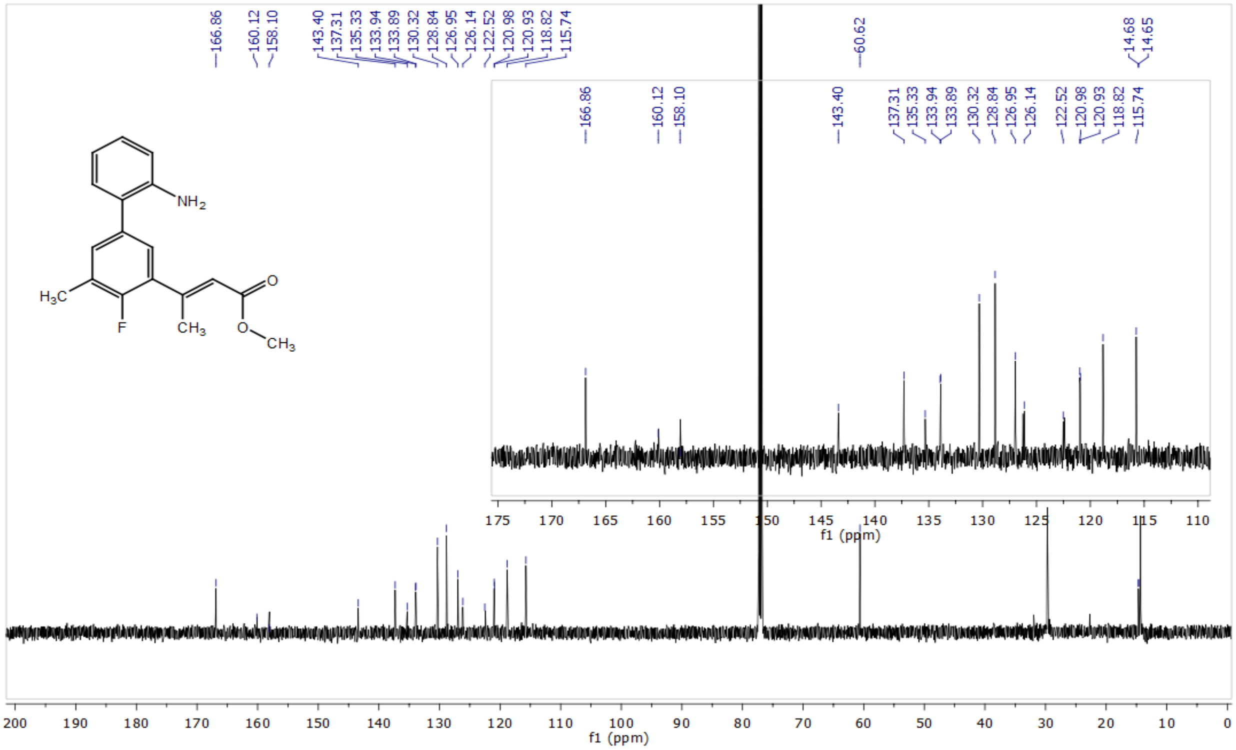

^{19}F NMR

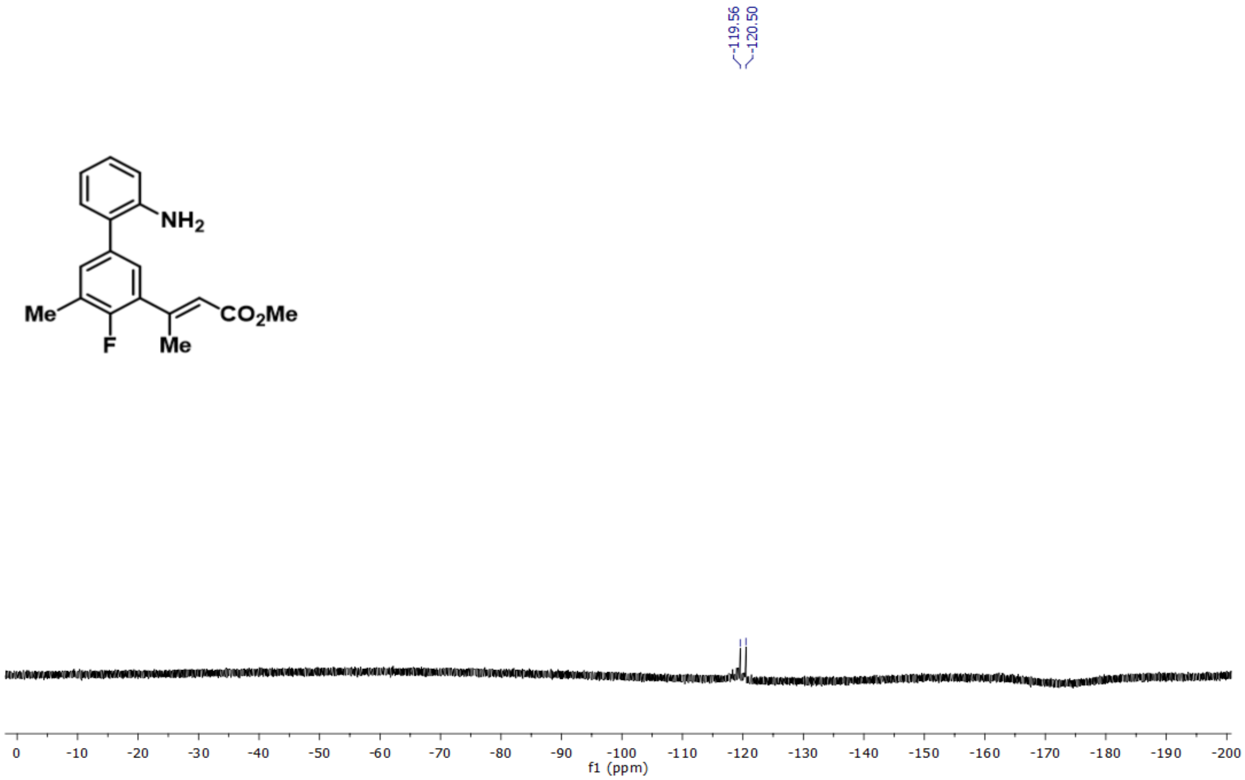

-119.56
-120.50
NH2
Me
F
Me
CO2Me
0 -10 -20 -30 -40 -50 -60 -70 -80 -90 -100 -110 -120 -130 -140 -150 -160 -170 -180 -190 -200
f1 (ppm)

***Tricosyl (E)-3-(2'-amino-4-fluoro-5-methyl-[1,1'-biphenyl]-3-yl)acrylate* (64)**

^{1}H NMR

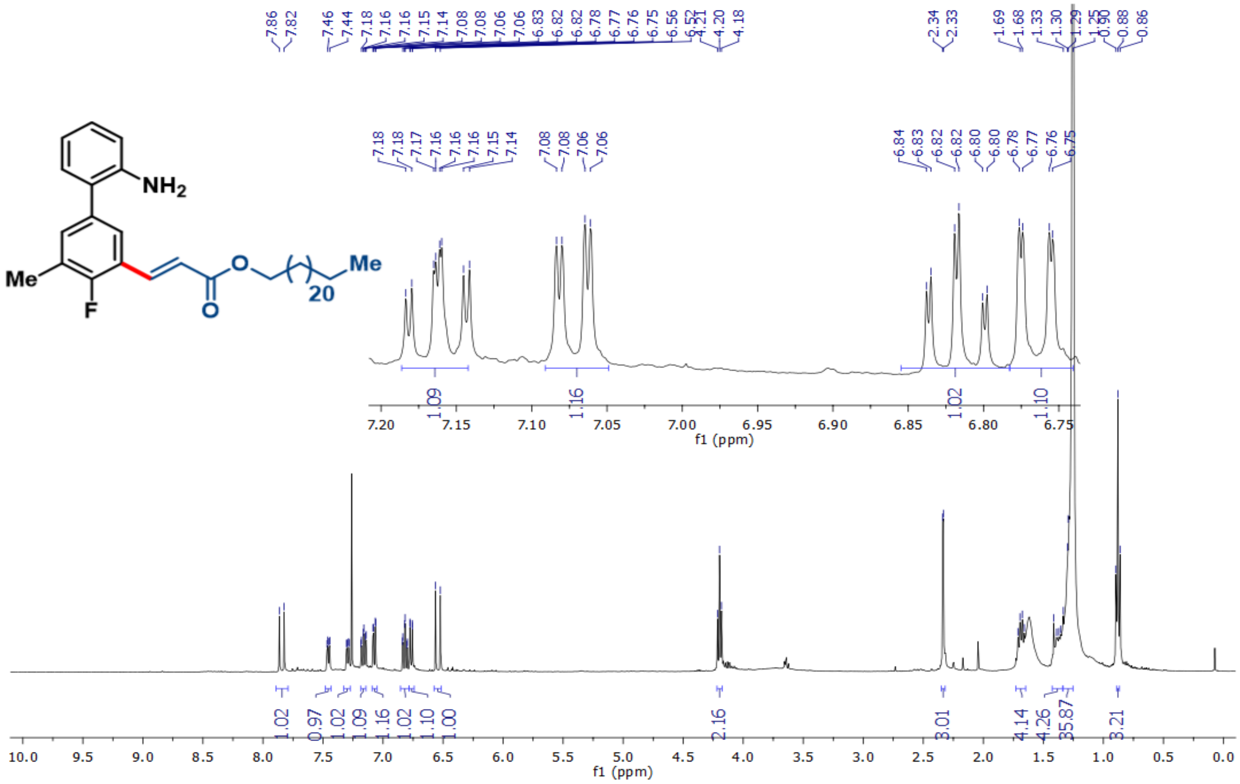

^{13}C NMR

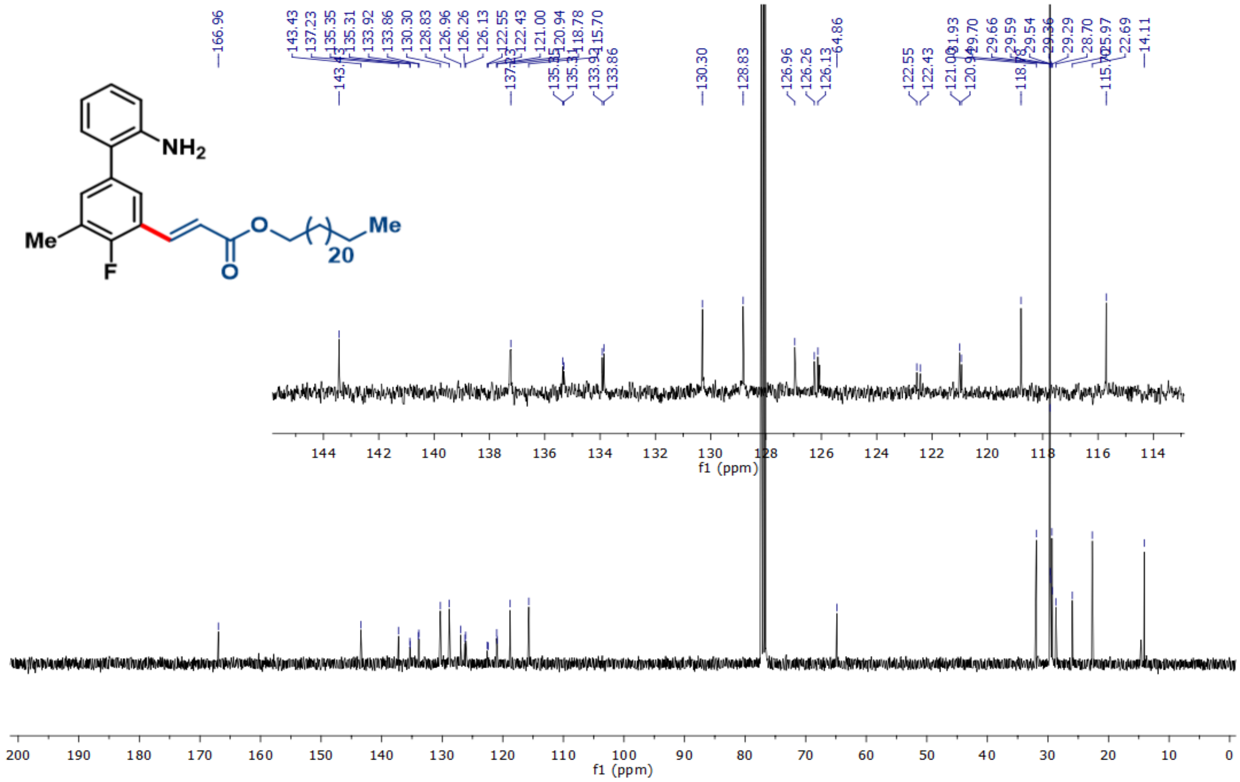

^{19}F NMR

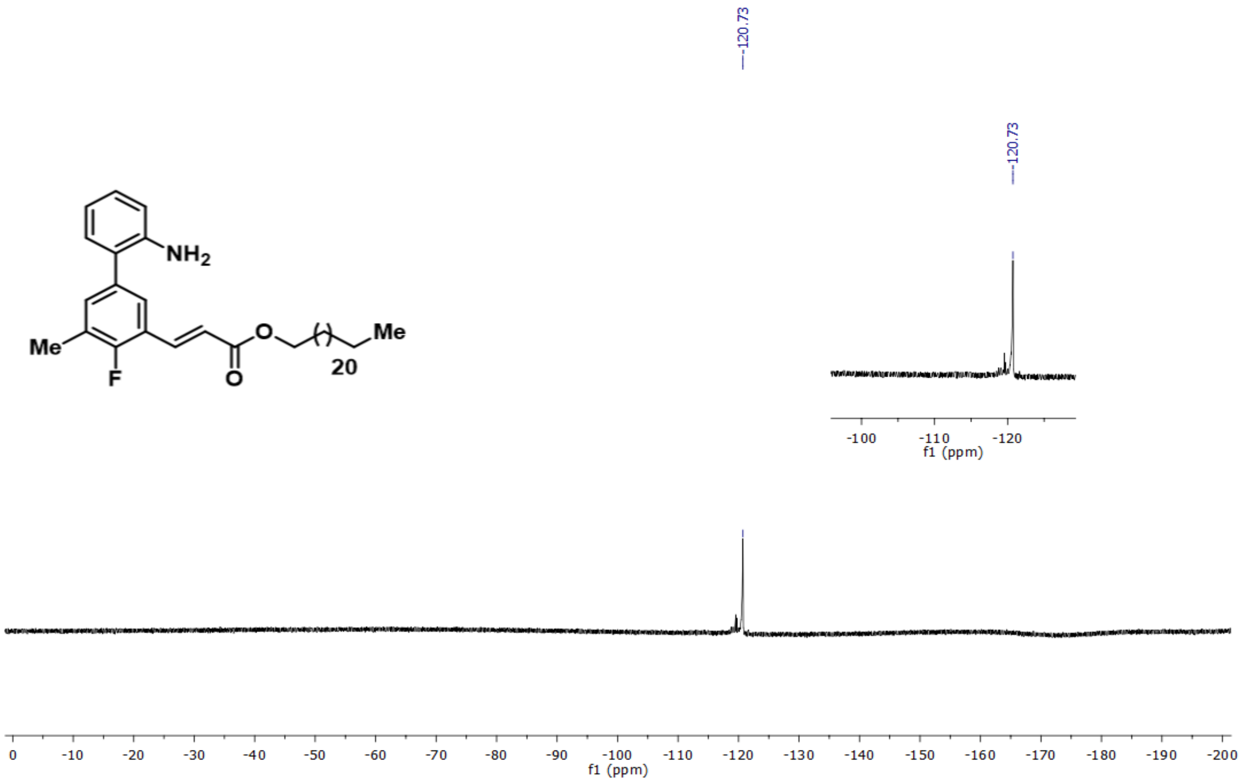

NH2
Me
F
O
O
Me
20
-120.73
-120.73
-100
-110
-120
f1 (ppm)
0
-10
-20
-30
-40
-50
-60
-70
-80
-90
-100
-110
-120
-130
-140
-150
-160
-170
-180
-190
-200
f1 (ppm)

***Dimethyl 2-(2'-amino-4-fluoro-2,6-dimethyl-[1,1'-biphenyl]-3-yl)maleate* (65)**

^{1}H NMR

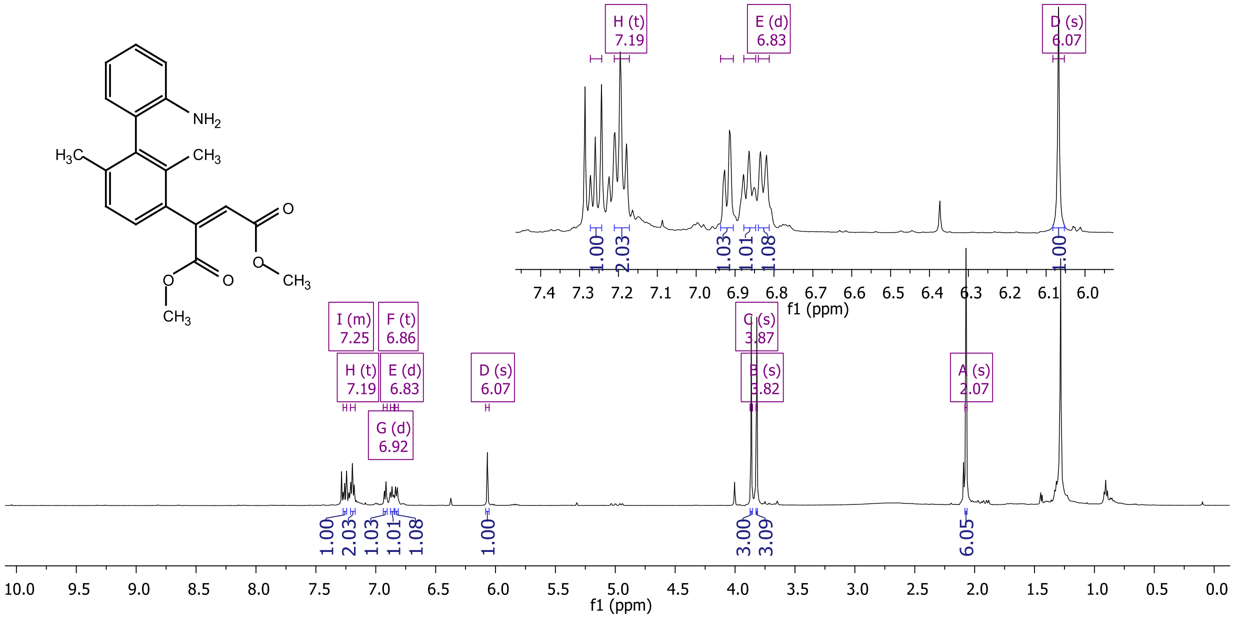

^{13}C NMR

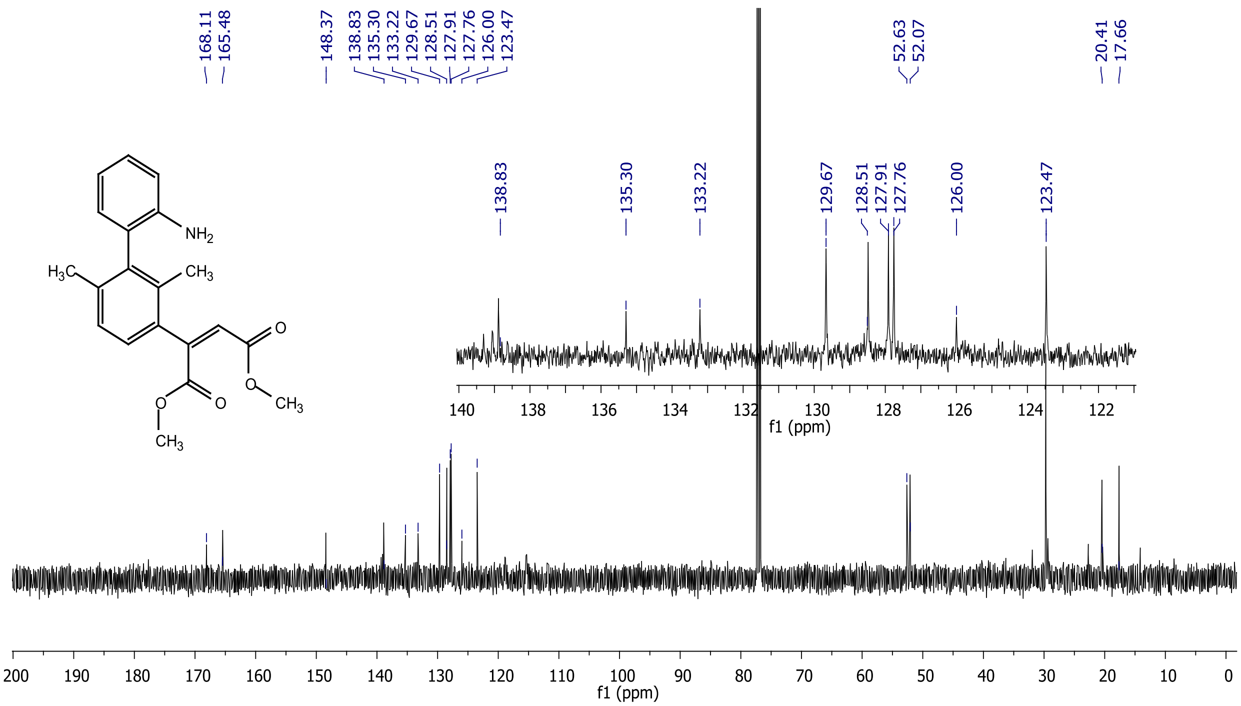

***Dimethyl 2-(2'-amino-2,5-dimethyl-[1,1'-biphenyl]-3-yl)maleate* (66)**

^{1}H NMR

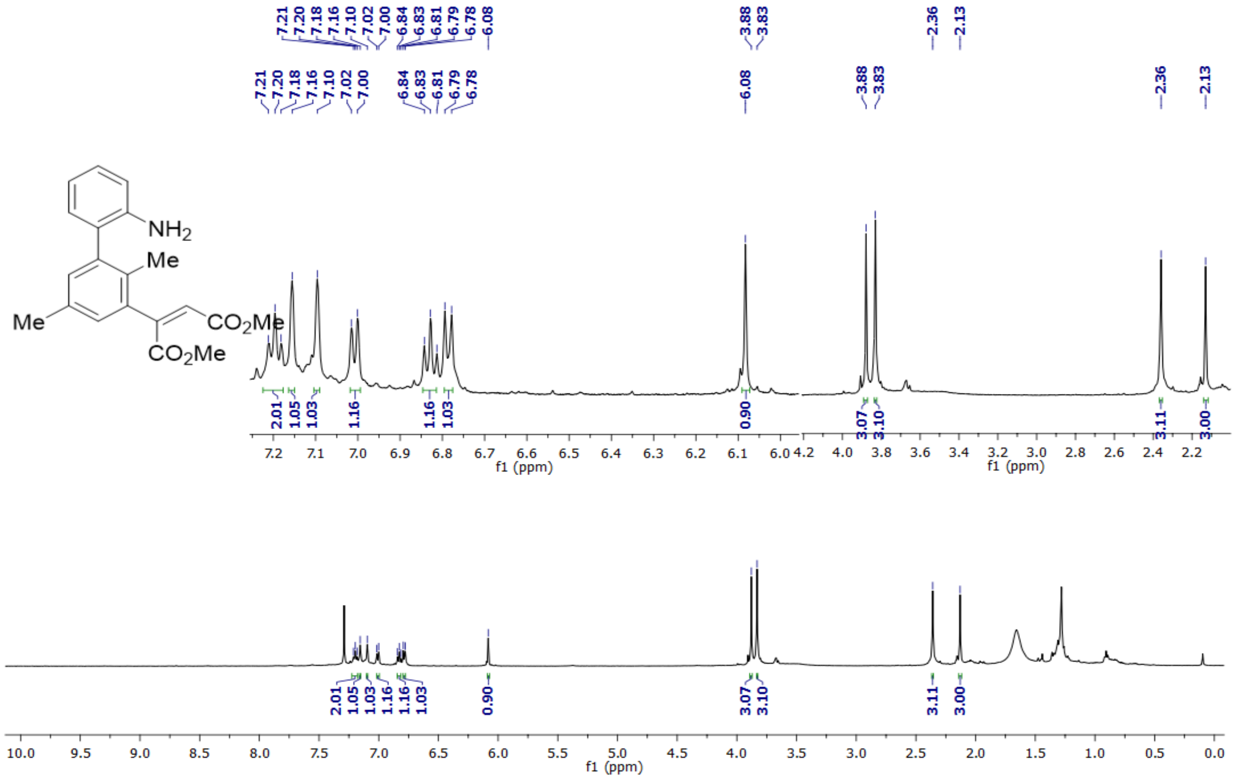

^{13}C NMR

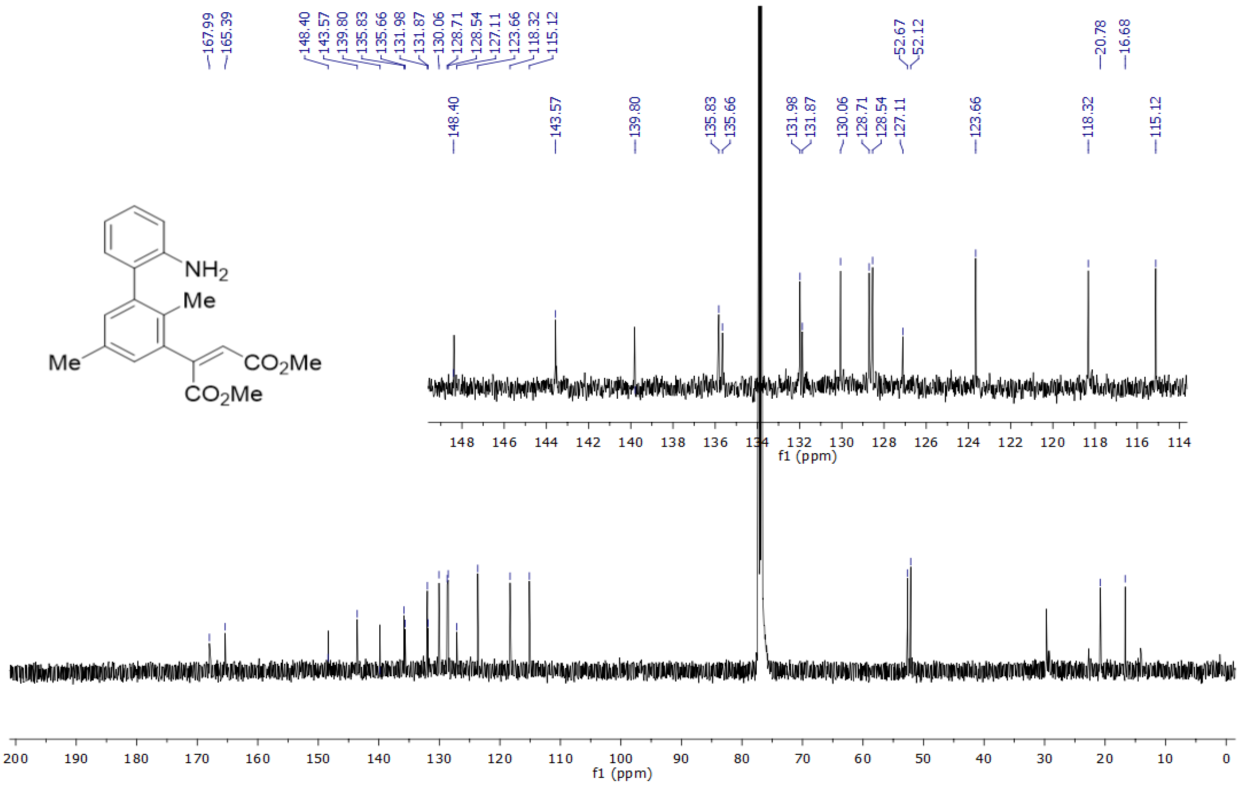

***Ethyl (E)-3-(2'-amino-2,5-difluoro-[1,1'-biphenyl]-3-yl)acrylate* (67)**

^{1}H NMR

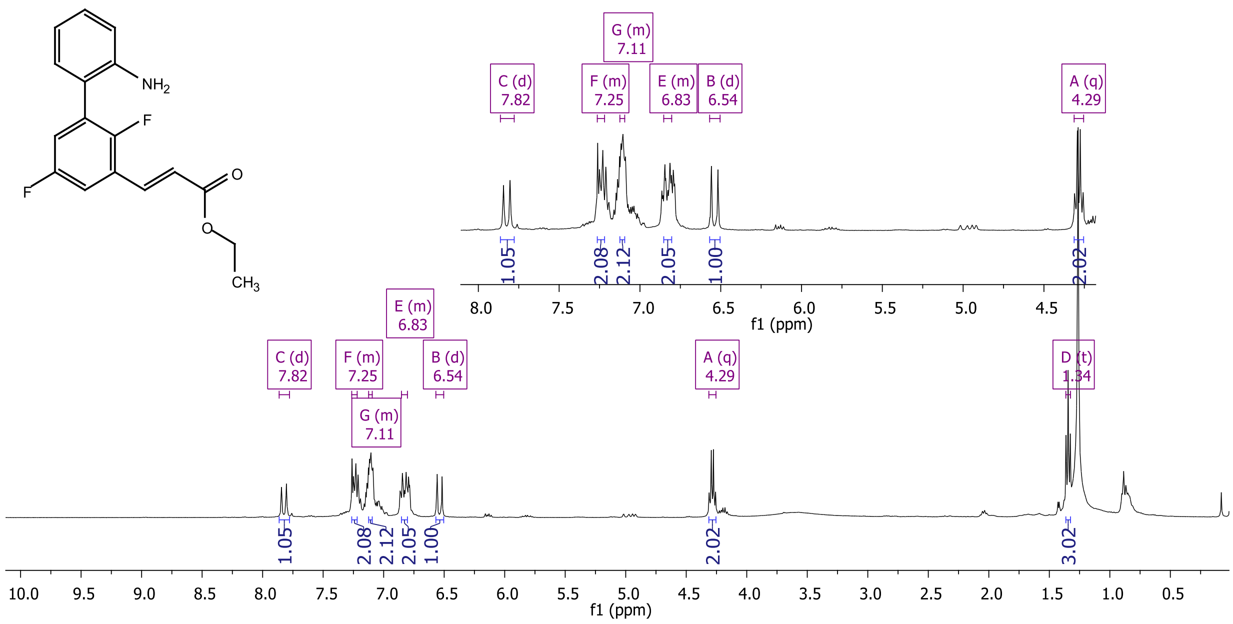

^{13}C NMR

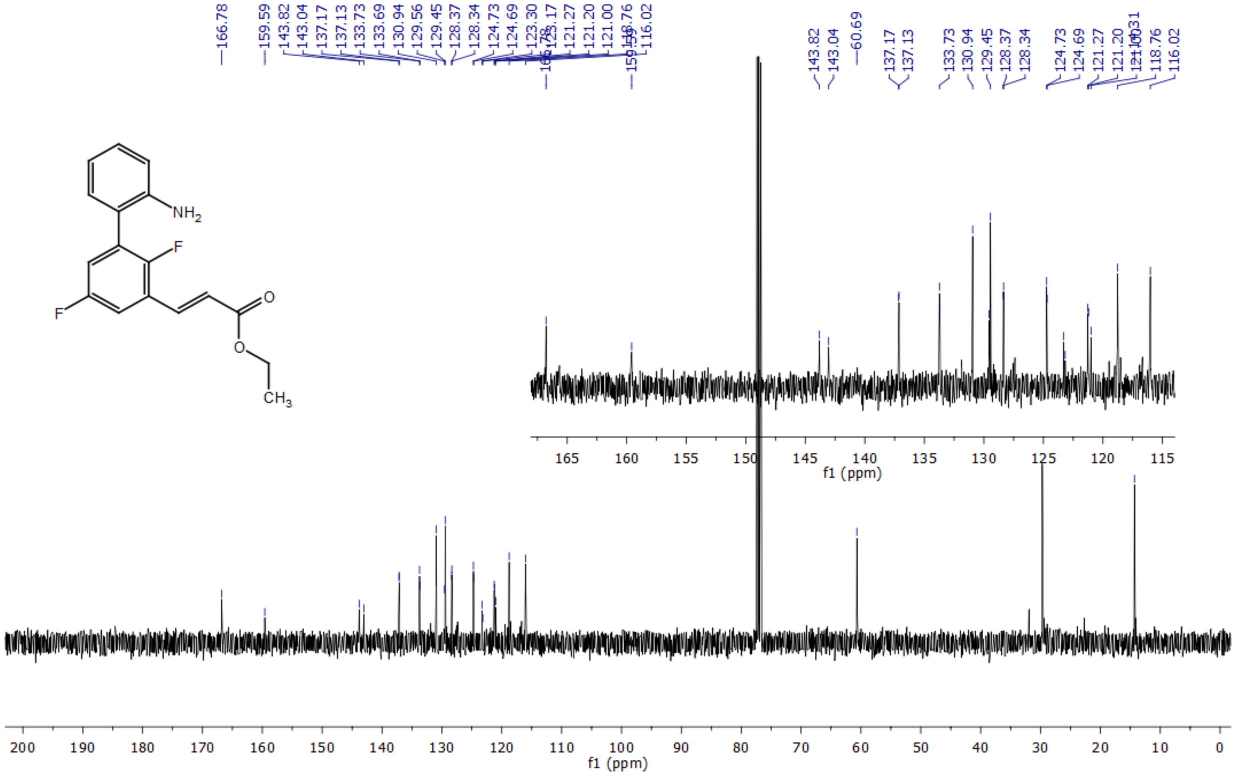

^{19}F NMR

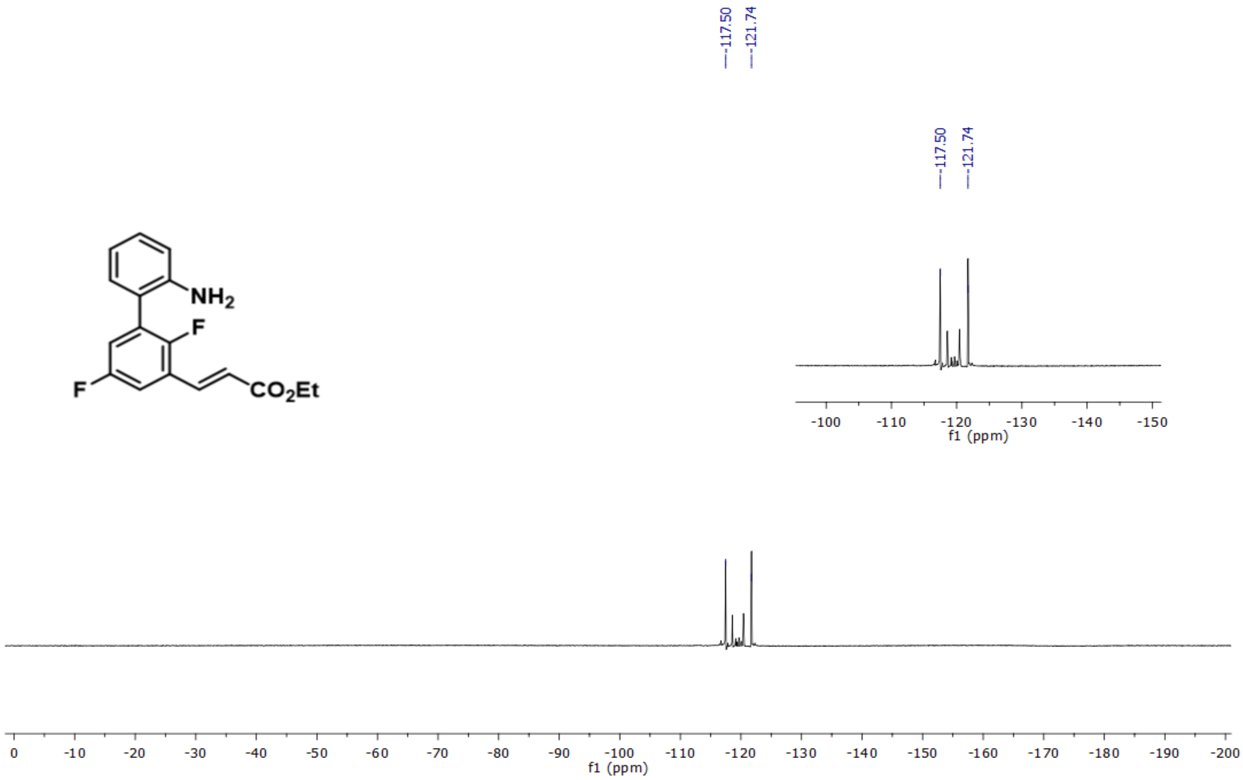

NH2
F
F
CO2Et
-117.50
-121.74
-117.50
-121.74
-100
-110
-120
-130
-140
-150
f1 (ppm)
0
-10
-20
-30
-40
-50
-60
-70
-80
-90
-100
-110
-120
-130
-140
-150
-160
-170
-180
-190
-200
f1 (ppm)

***Ethyl (E)-3-(2'-amino-3'-fluoro-5-methyl-[1,1'-biphenyl]-3-yl)acrylate* (68)**

^{1}H NMR

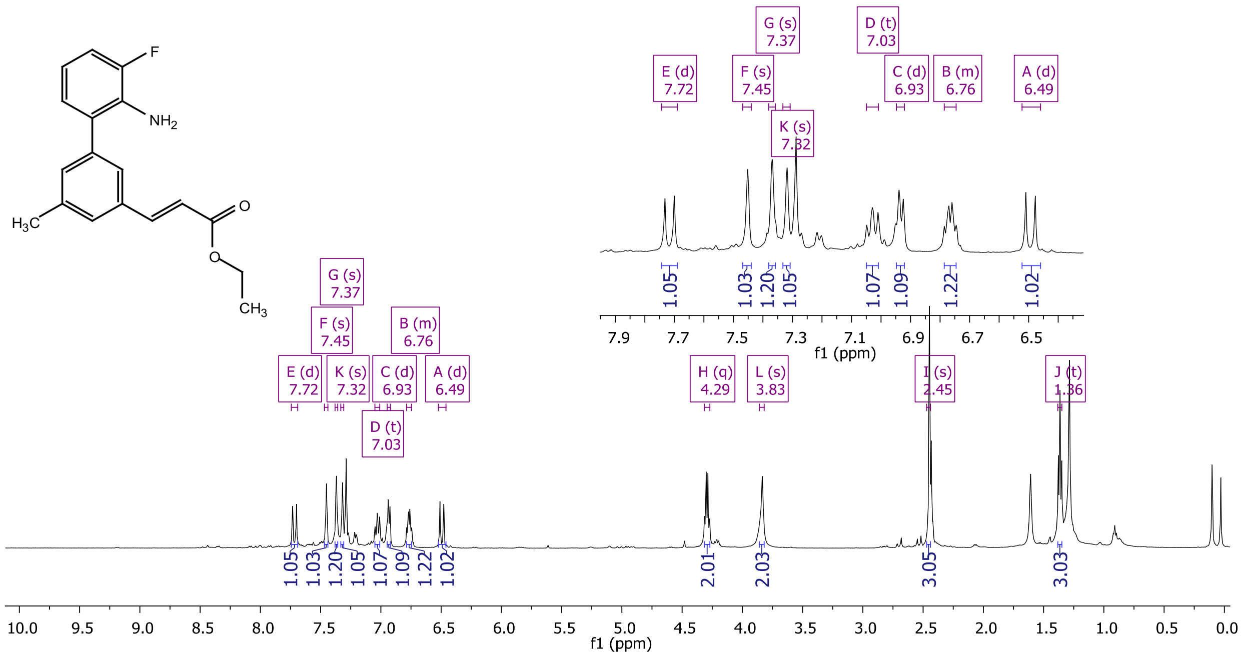

^{13}C NMR

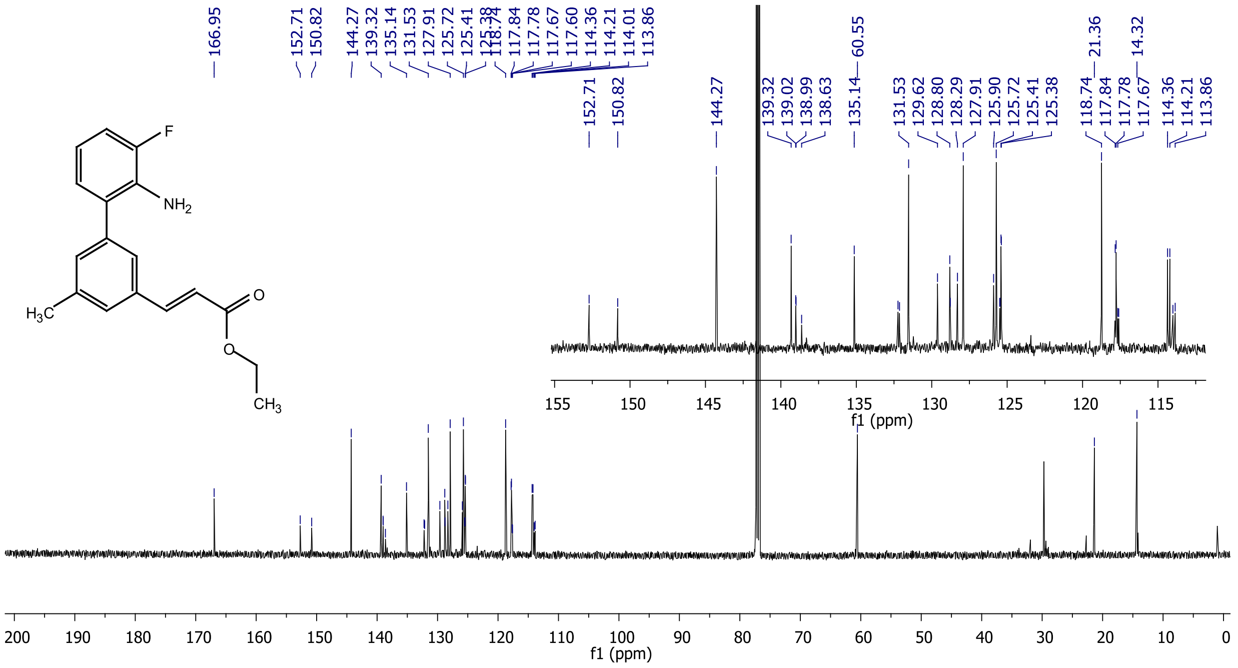

^{19}F NMR

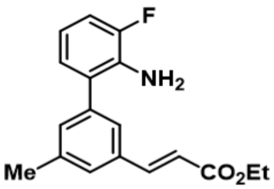
F
NH2
Me
CO2Et

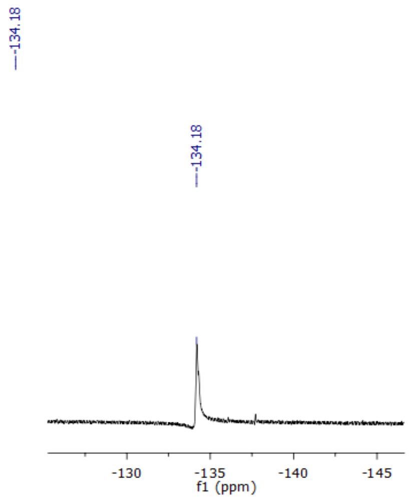
-134.18
-134.18
-130
-135
-140
-145
f1 (ppm)

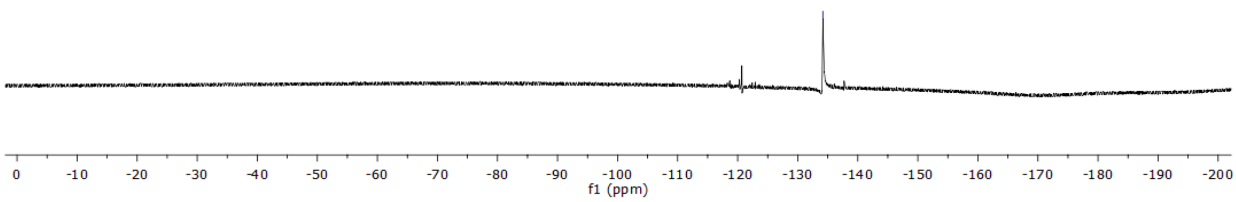
0
-10
-20
-30
-40
-50
-60
-70
-80
-90
-100
-110
-120
-130
-140
-150
-160
-170
-180
-190
-200
f1 (ppm)

(E)-4-(2'-amino-3'-fluoro-5-methyl-[1,1'-biphenyl]-3-yl)but-3-en-2-one (**69**)

^{1}H NMR

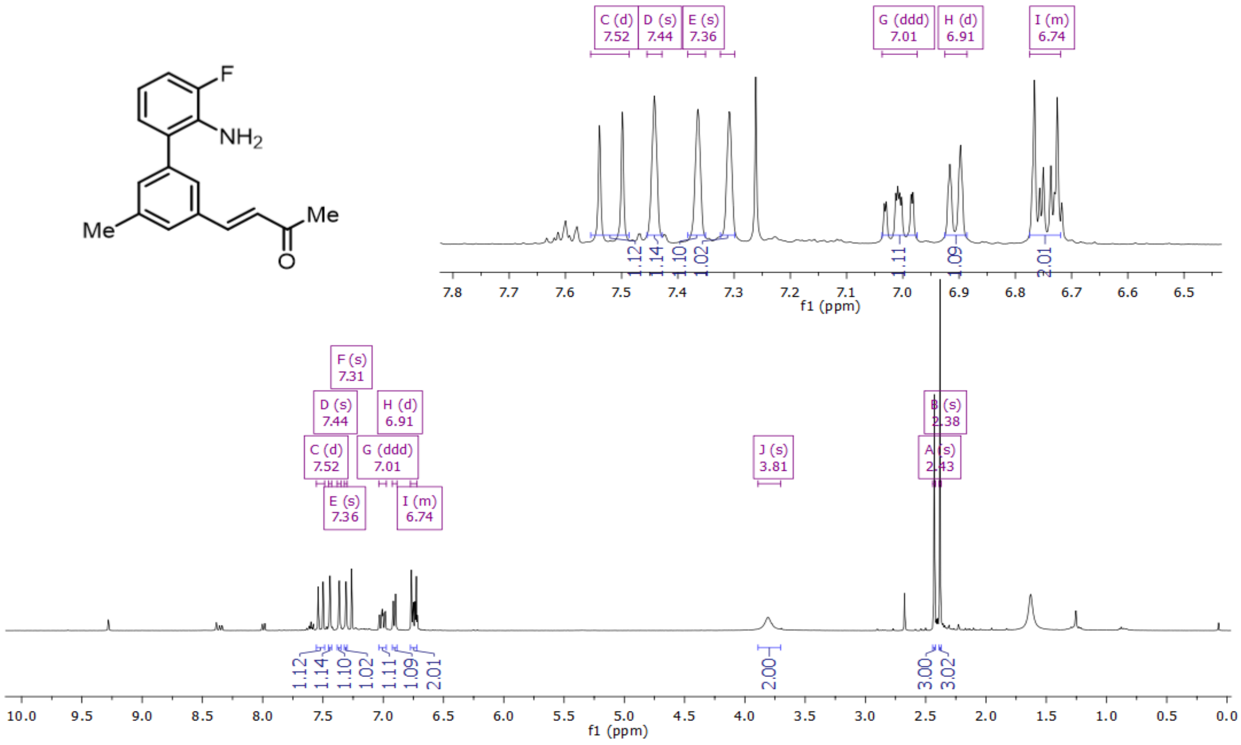

^{13}C NMR

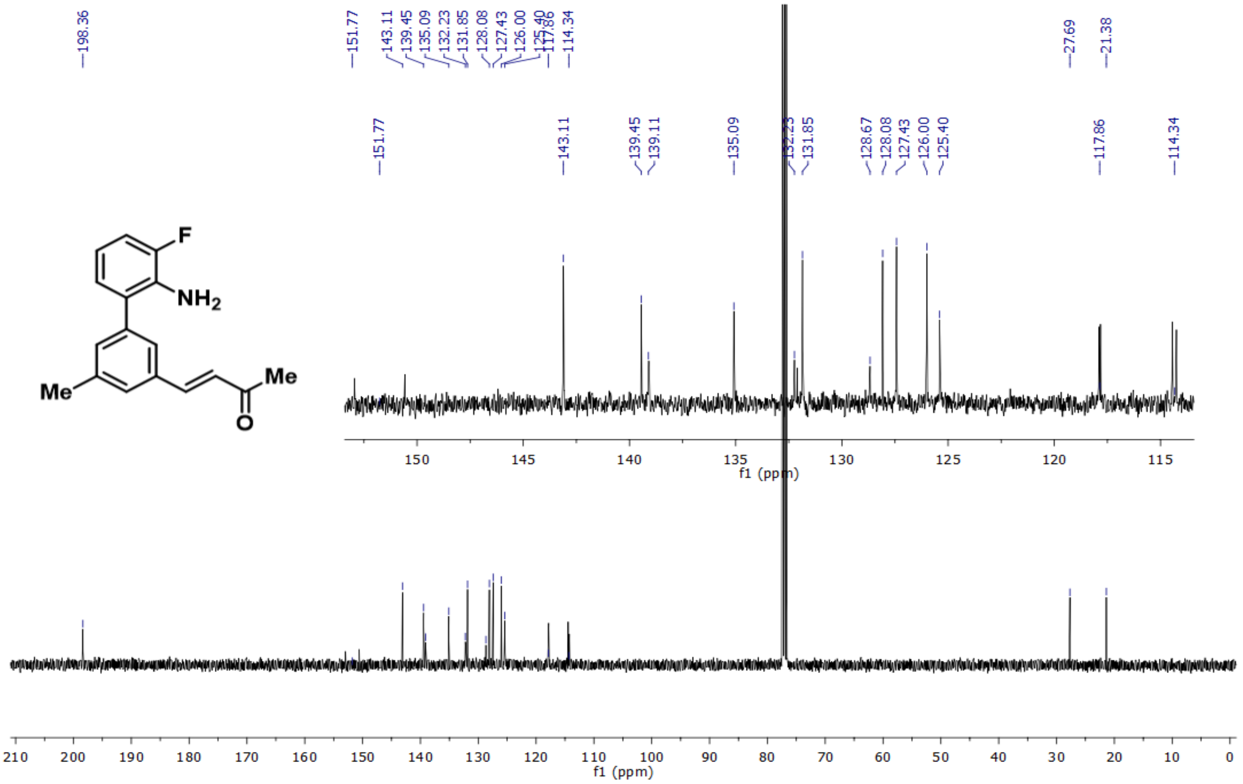

^{19}F NMR

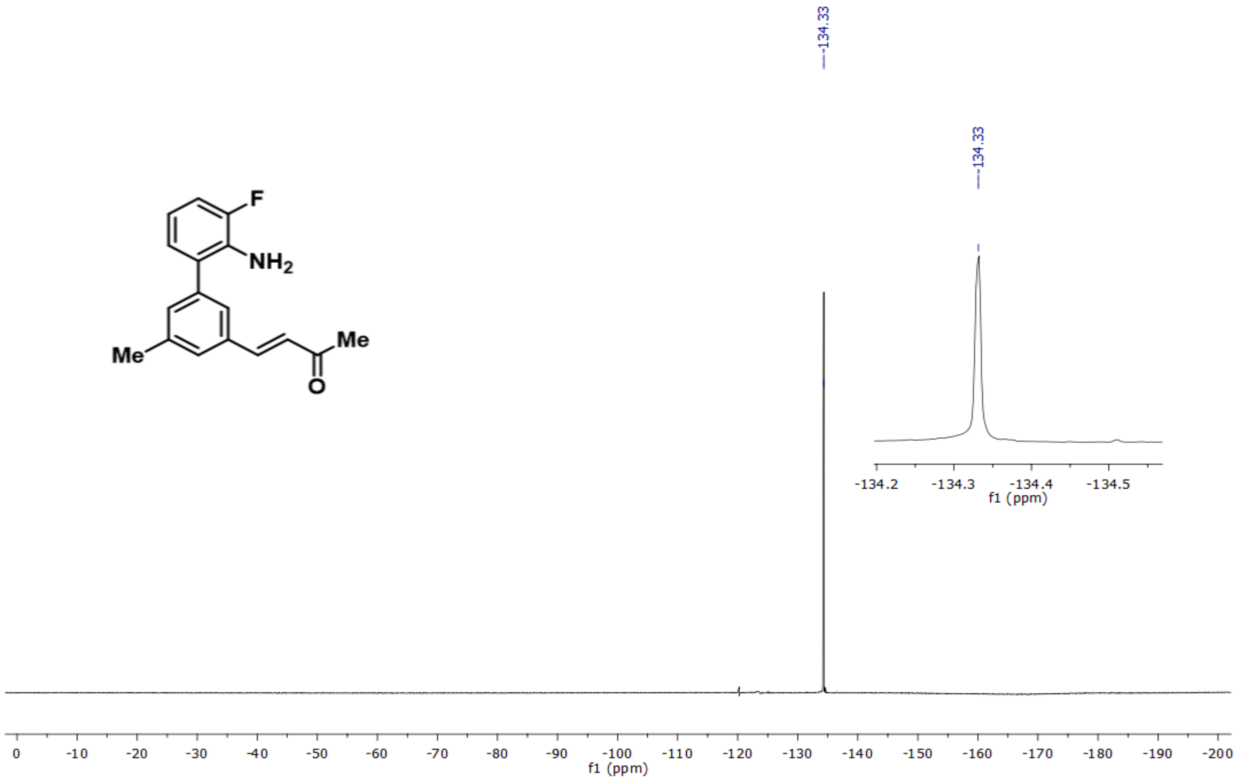
--134.33
--134.33
-134.2
-134.3
-134.4
-134.5
f1 (ppm)
0
-10
-20
-30
-40
-50
-60
-70
-80
-90
-100
-110
-120
-130
-140
-150
-160
-170
-180
-190
-200
f1 (ppm)

***Dmethyl 2-(2'-amino-3'-fluoro-5-methyl-[1,1'-biphenyl]-3-yl)maleate* (70)**

^{1}H NMR

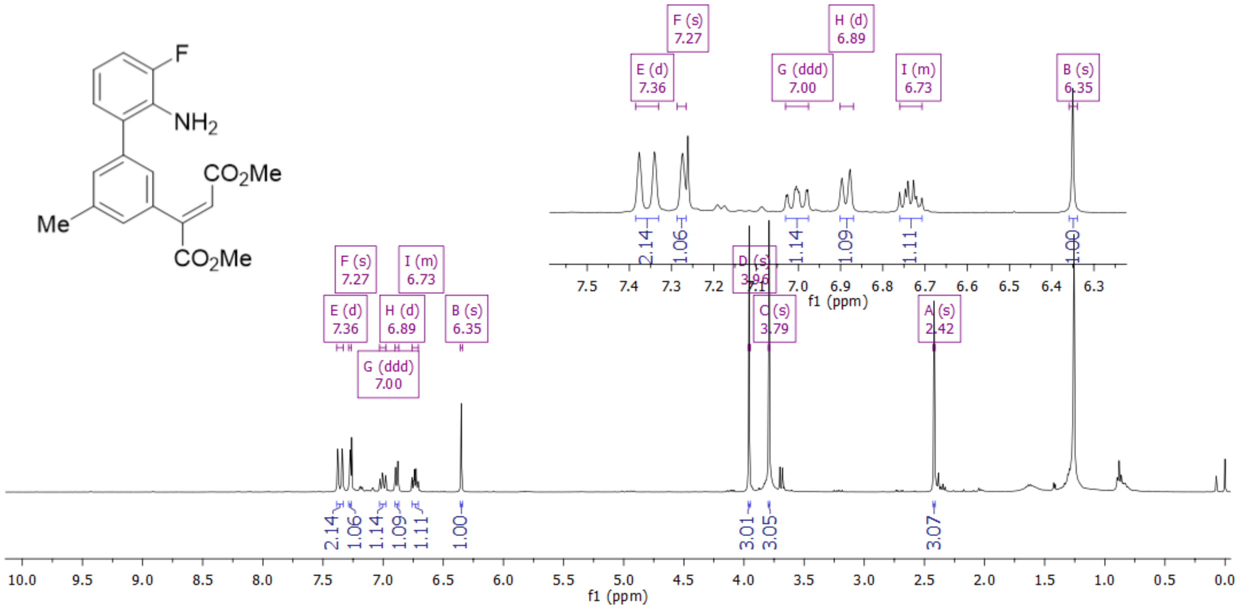

^{13}C NMR

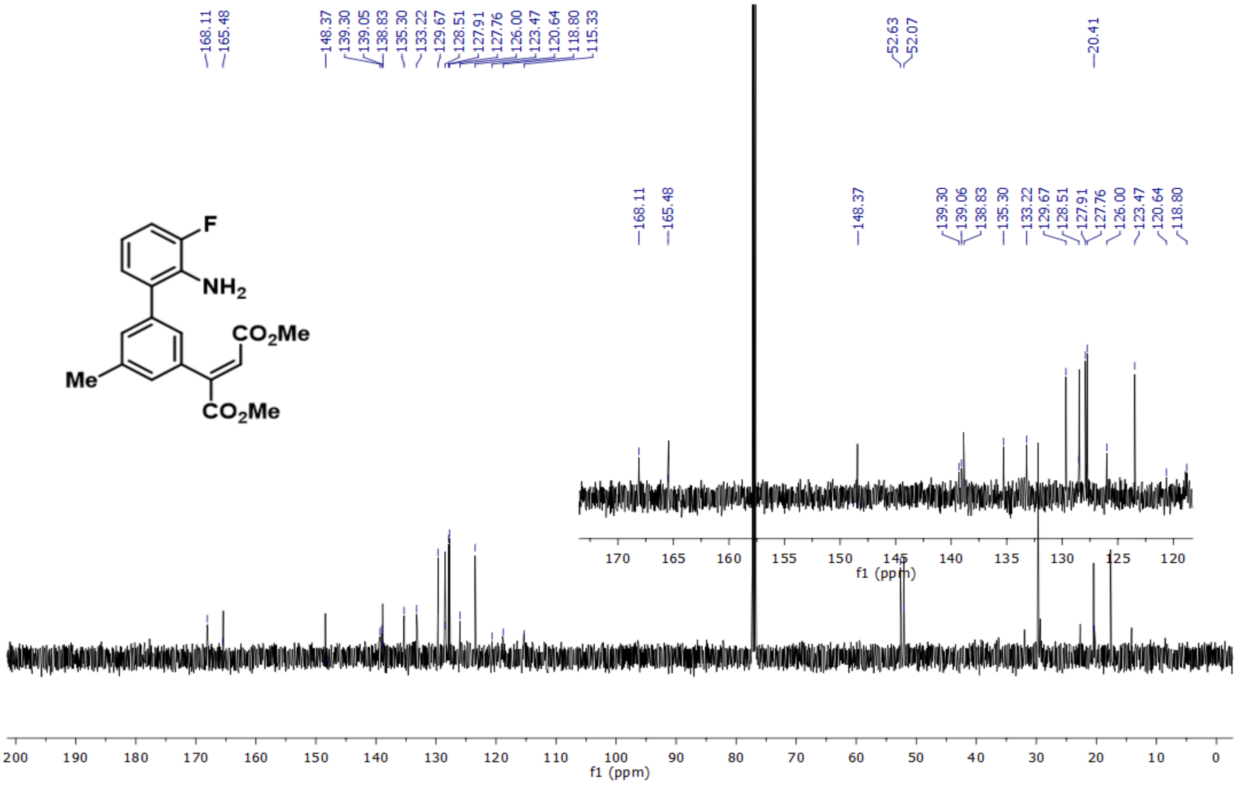

^{19}F NMR

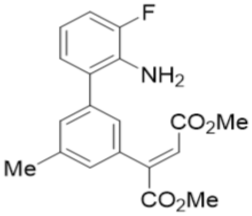
F
NH2
CO2Me
Me
CO2Me

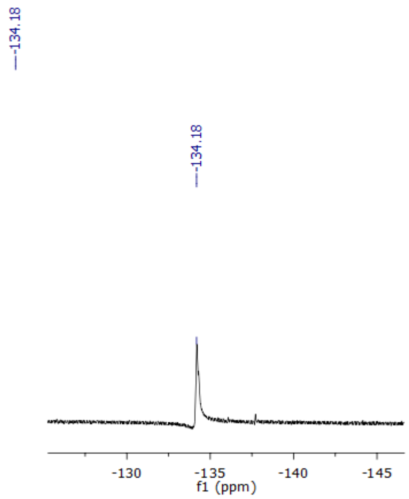
-134.18
-134.18
-130
-135
-140
-145
f1 (ppm)

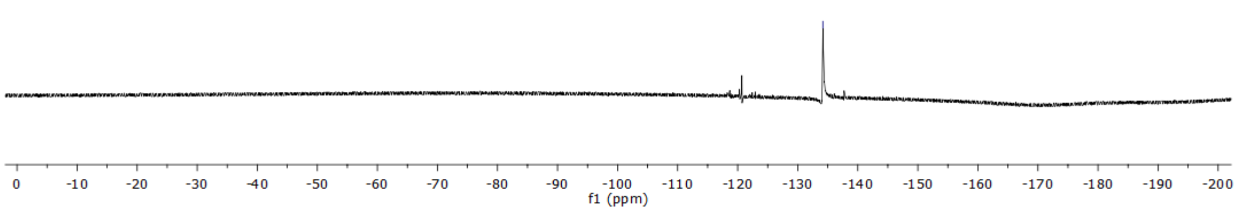
0
-10
-20
-30
-40
-50
-60
-70
-80
-90
-100
-110
-120
-130
-140
-150
-160
-170
-180
-190
-200
f1 (ppm)

***(E)*-3-(2'-amino-3'-fluoro-5-methyl-[1,1'-biphenyl]-3-yl)acrylonitrile** (**71**)

^{1}H NMR

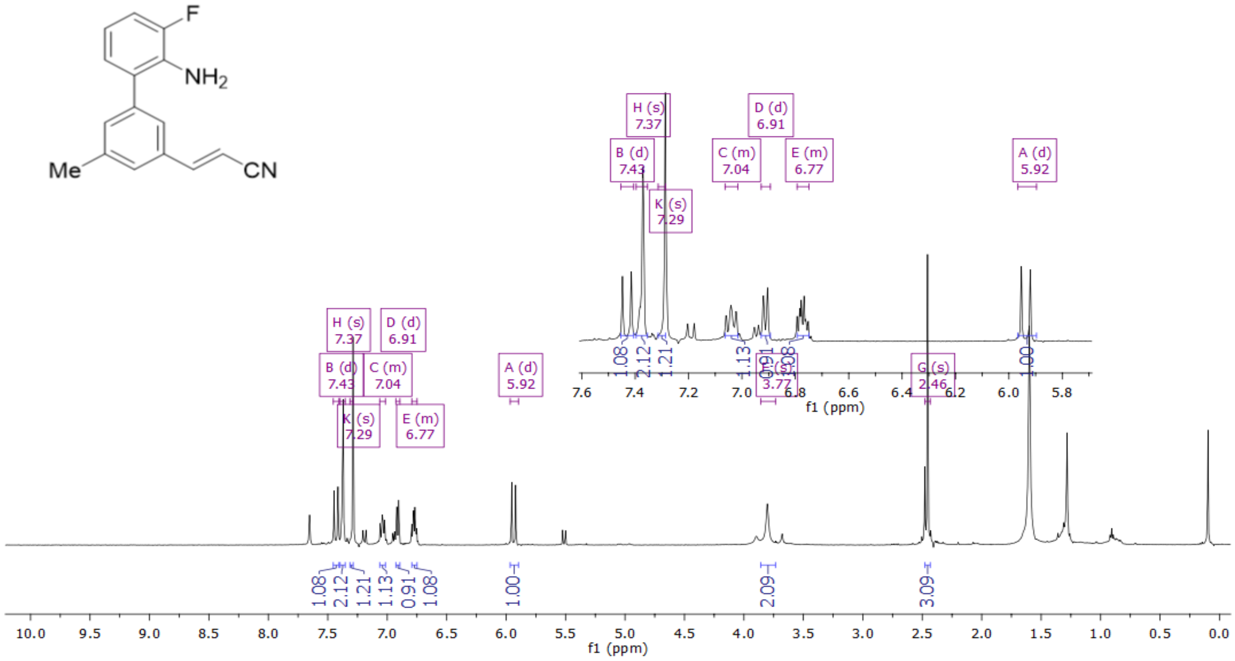

^{13}C NMR

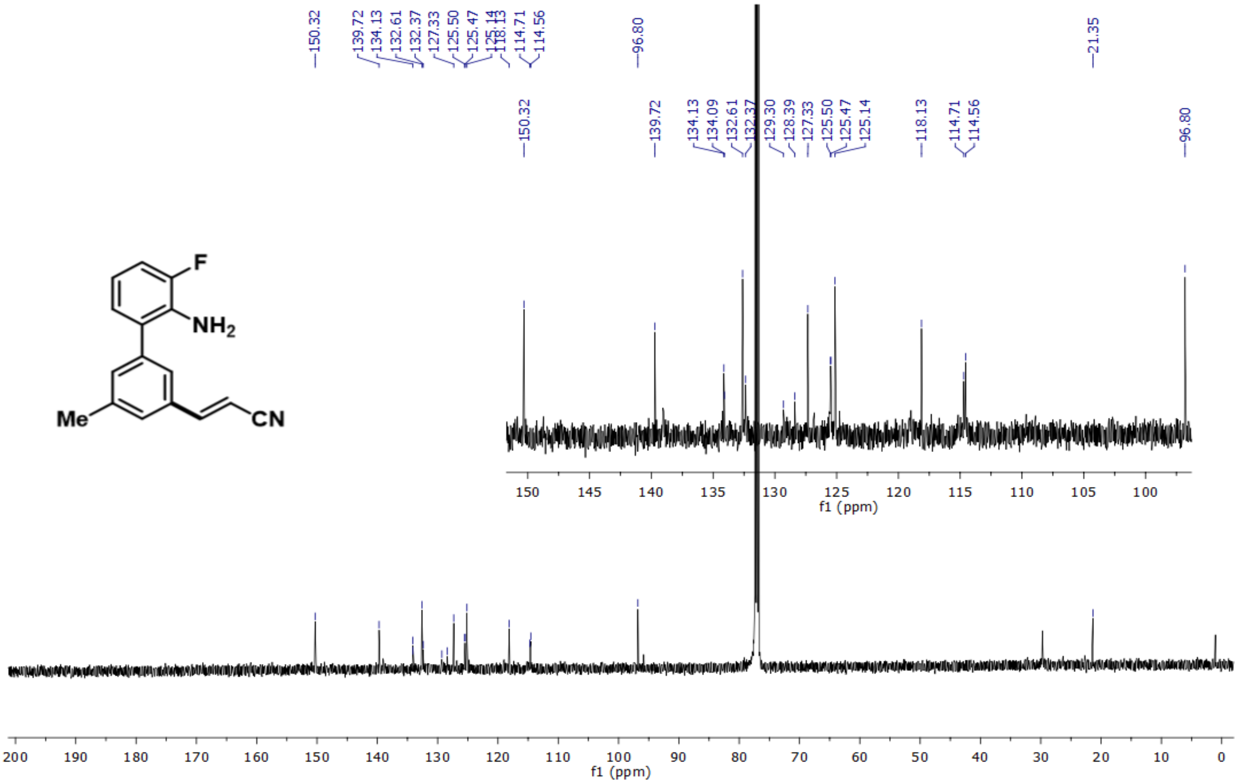

^{19}F NMR

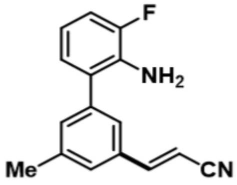
F
NH2
Me
CN

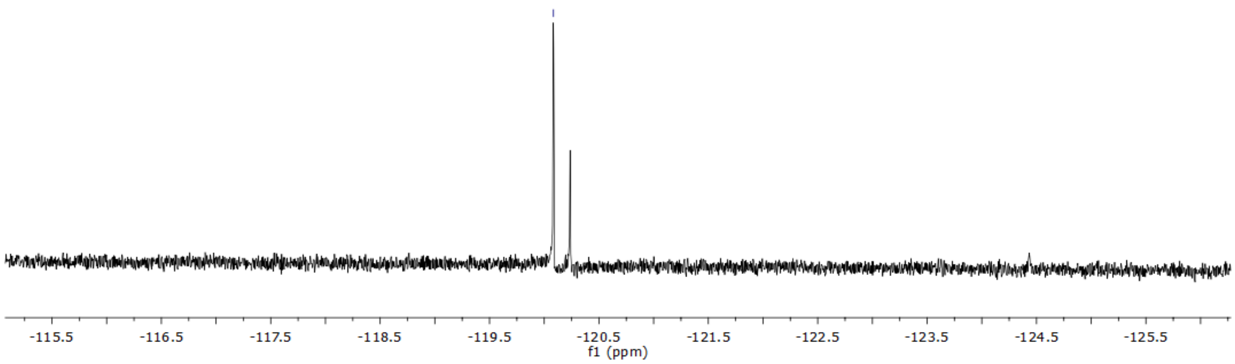
-120.08
-115.5
-116.5
-117.5
-118.5
-119.5
-120.5
-121.5
-122.5
-123.5
-124.5
-125.5
f1 (ppm)

***Ethyl (E)-3-(1-(2-aminophenyl)-1H-indol-3-yl)acrylate* (72)**

^{1}H NMR

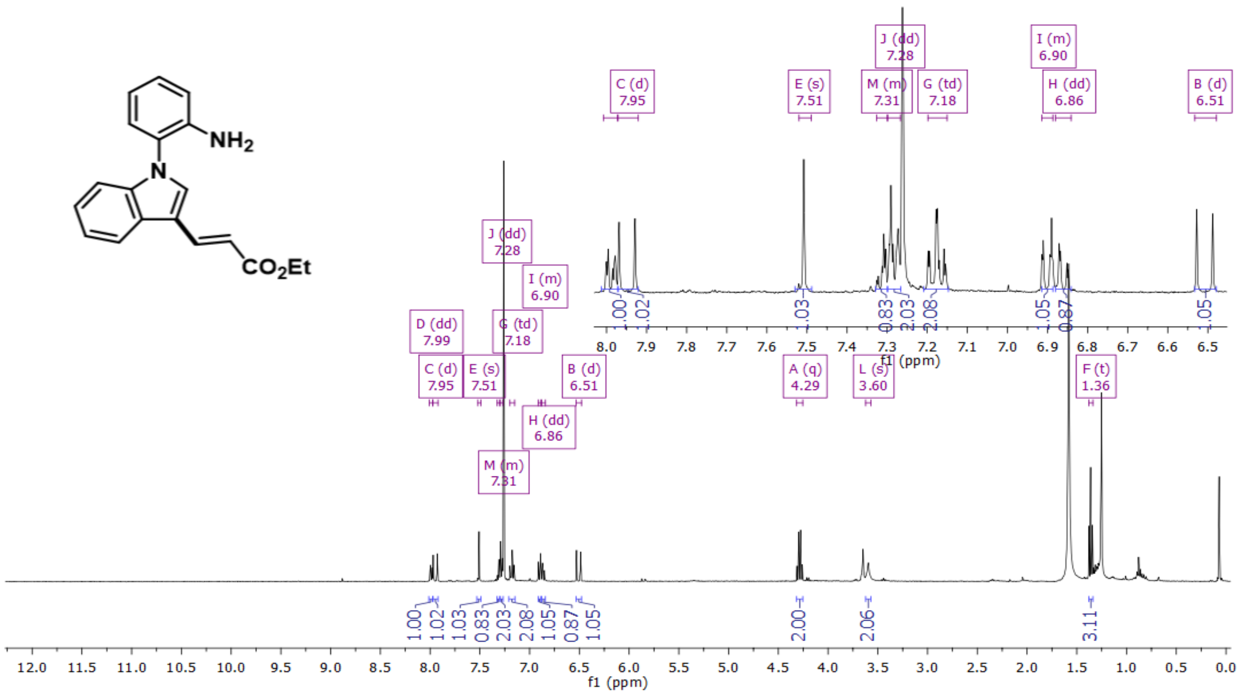

^{13}C NMR

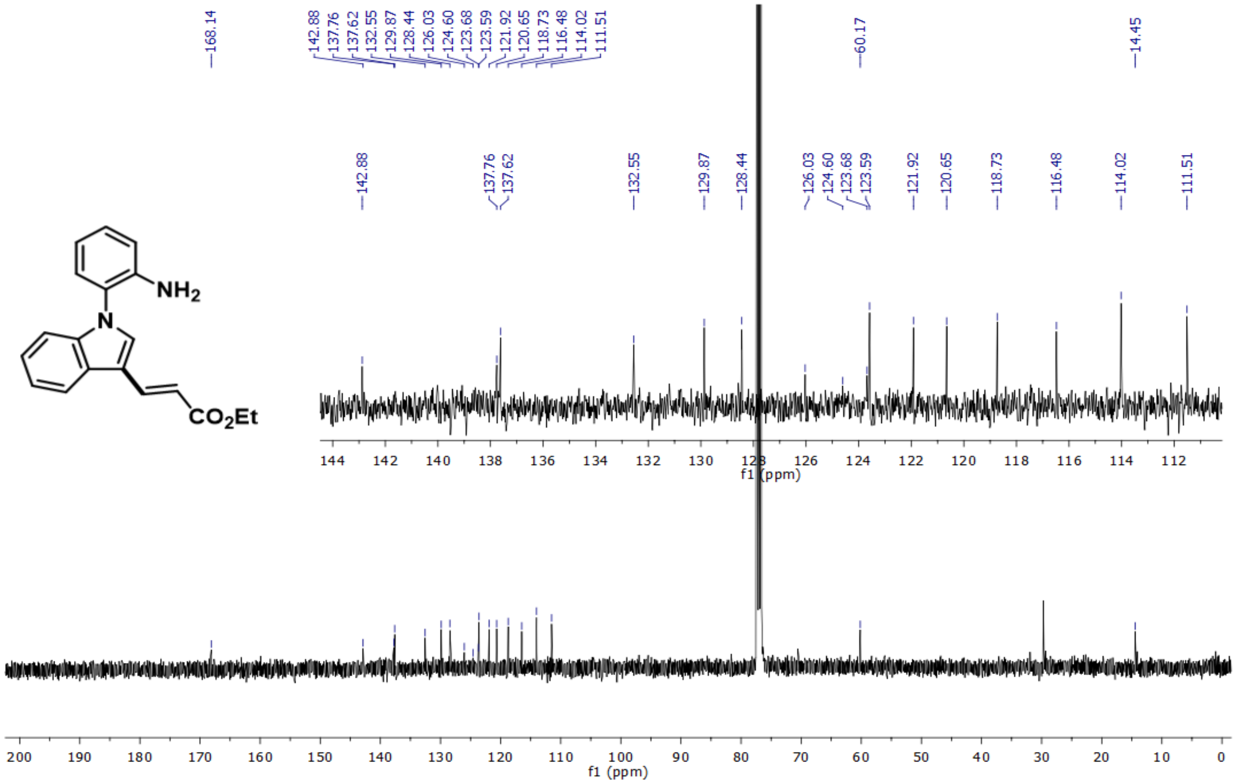

Ethyl (E)-3-(3'-chloro-2'-formyl-[1,1'-biphenyl]-3-yl)acrylate (**73**)

^{1}H NMR

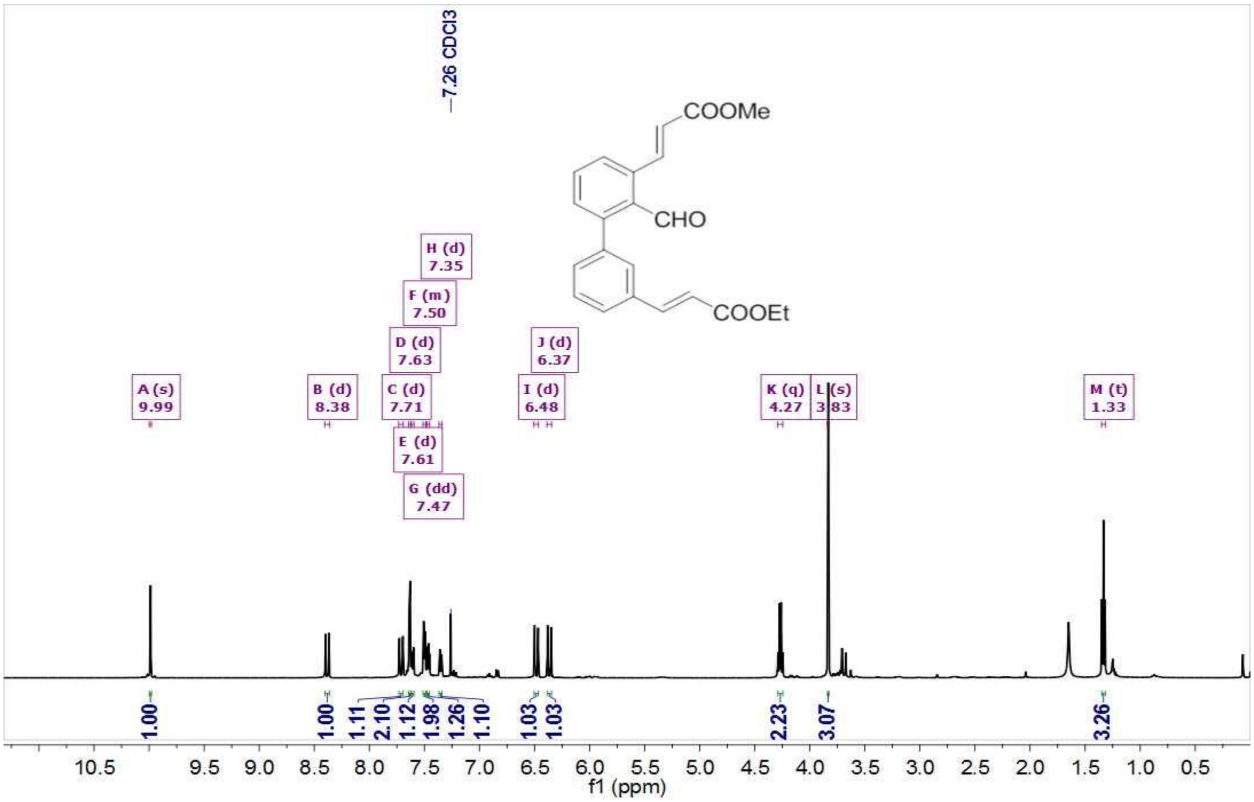

^{13}C NMR

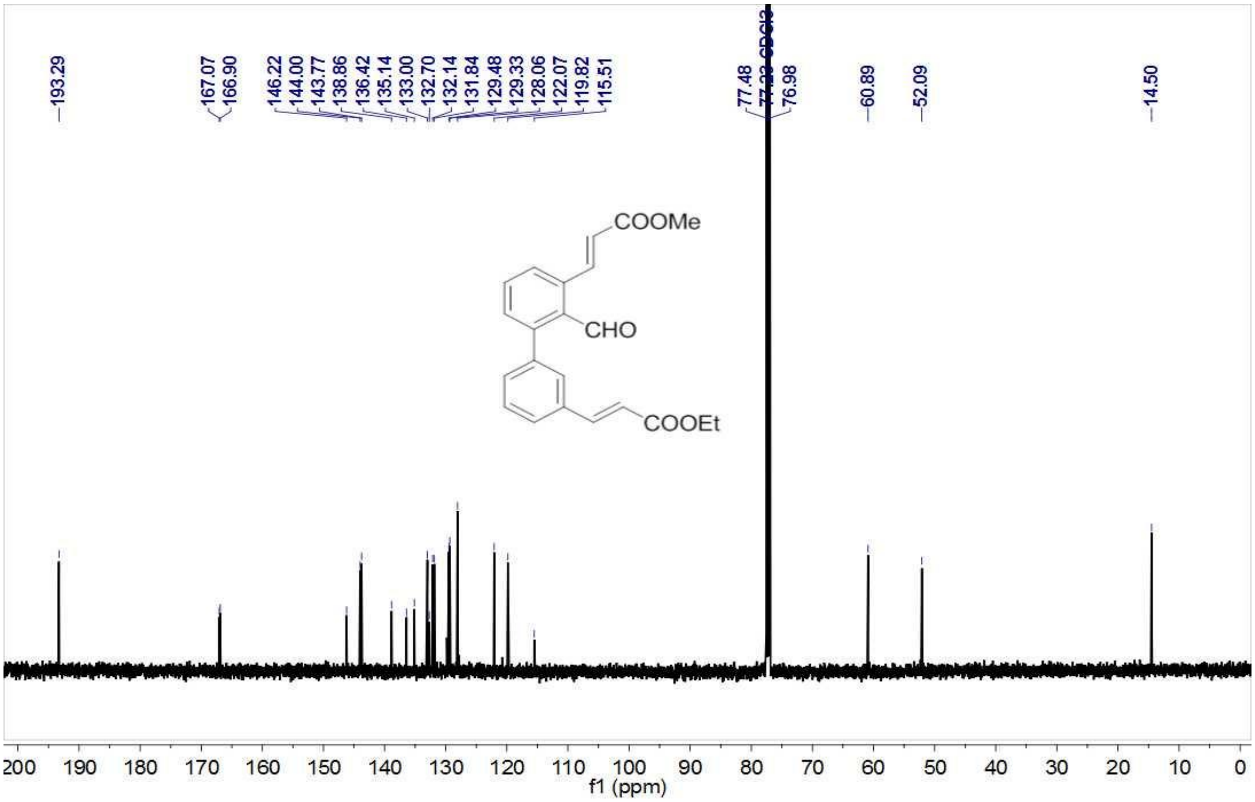

Ethyl (E)-3-(2'-formyl-3'-((4-methylphenyl)sulfonamido)-[1,1'-biphenyl]-3-yl)acrylate **(74)**; **^{1}H NMR**

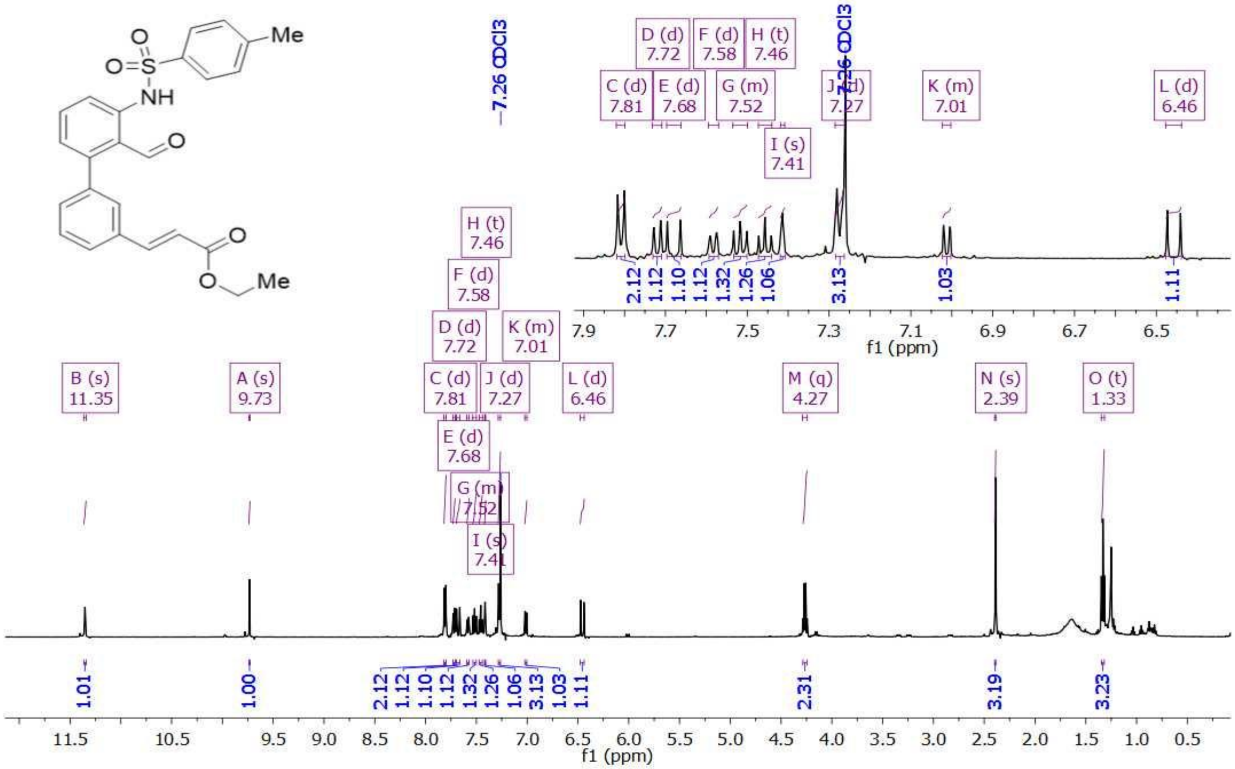

^{13}C NMR

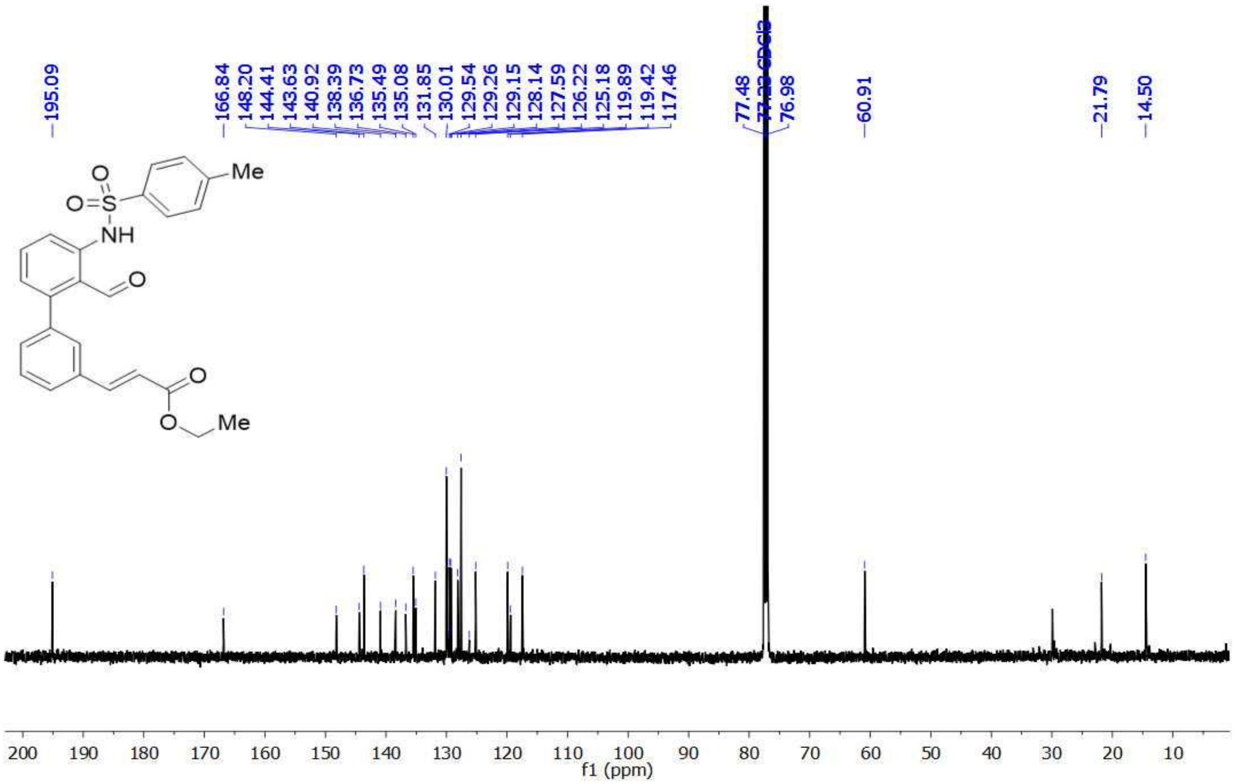

Methyl (E)-3-(9-oxo-9H-fluoren-1-yl)acrylate **(75)**

^{1}H NMR

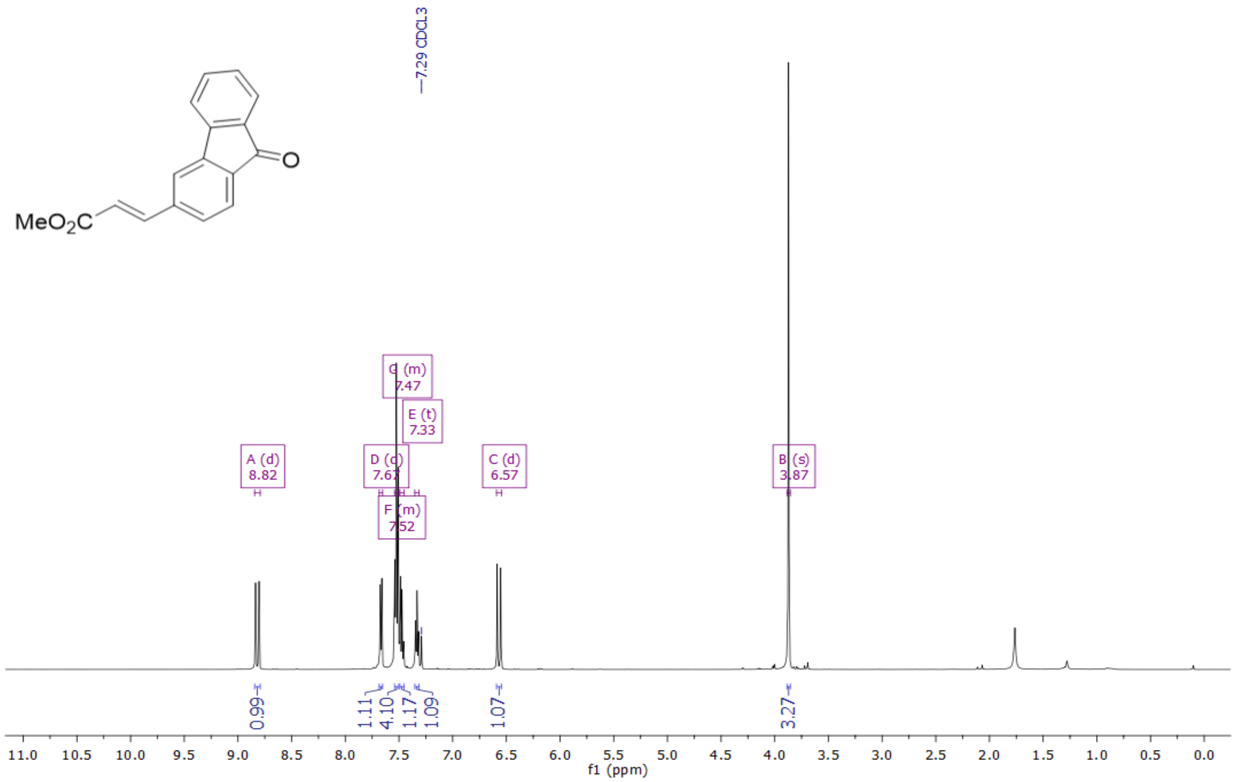

^{13}C NMR

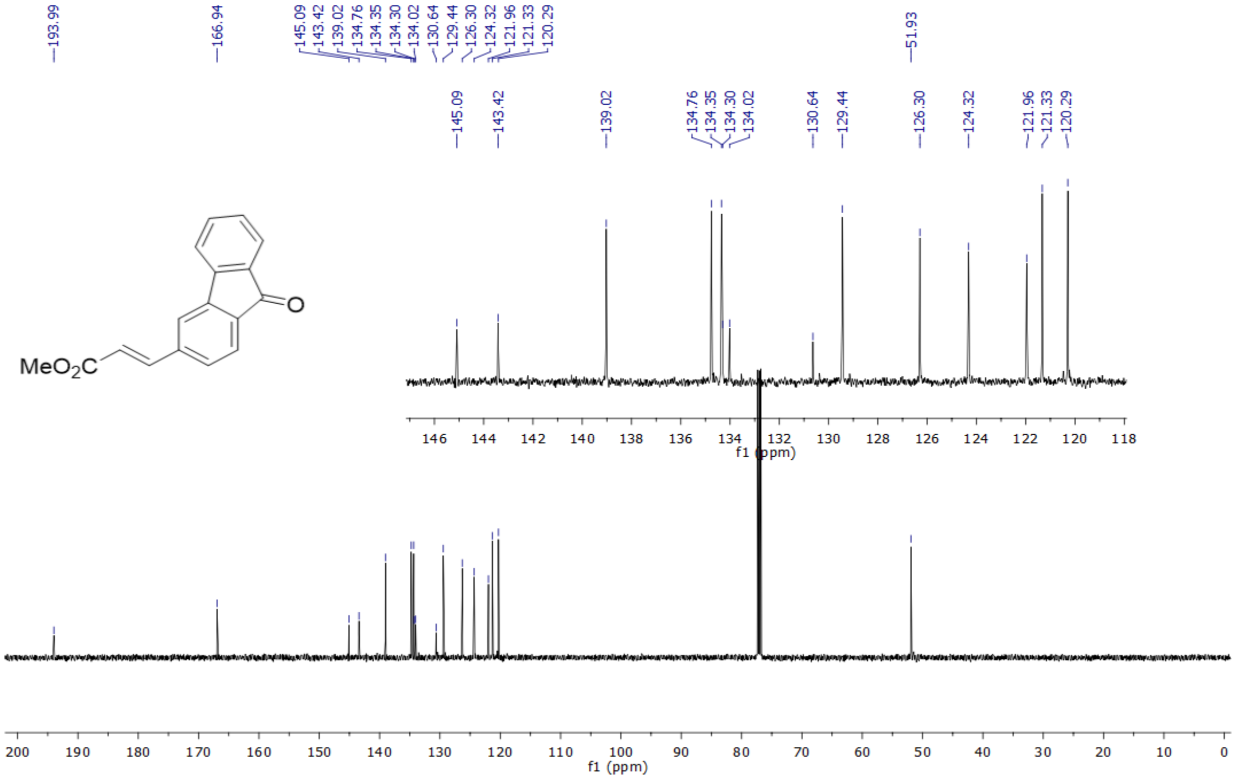

Ethyl (E)-3-(9-oxo-9H-fluoren-3-yl)acrylate **(76)**

^{1}H NMR

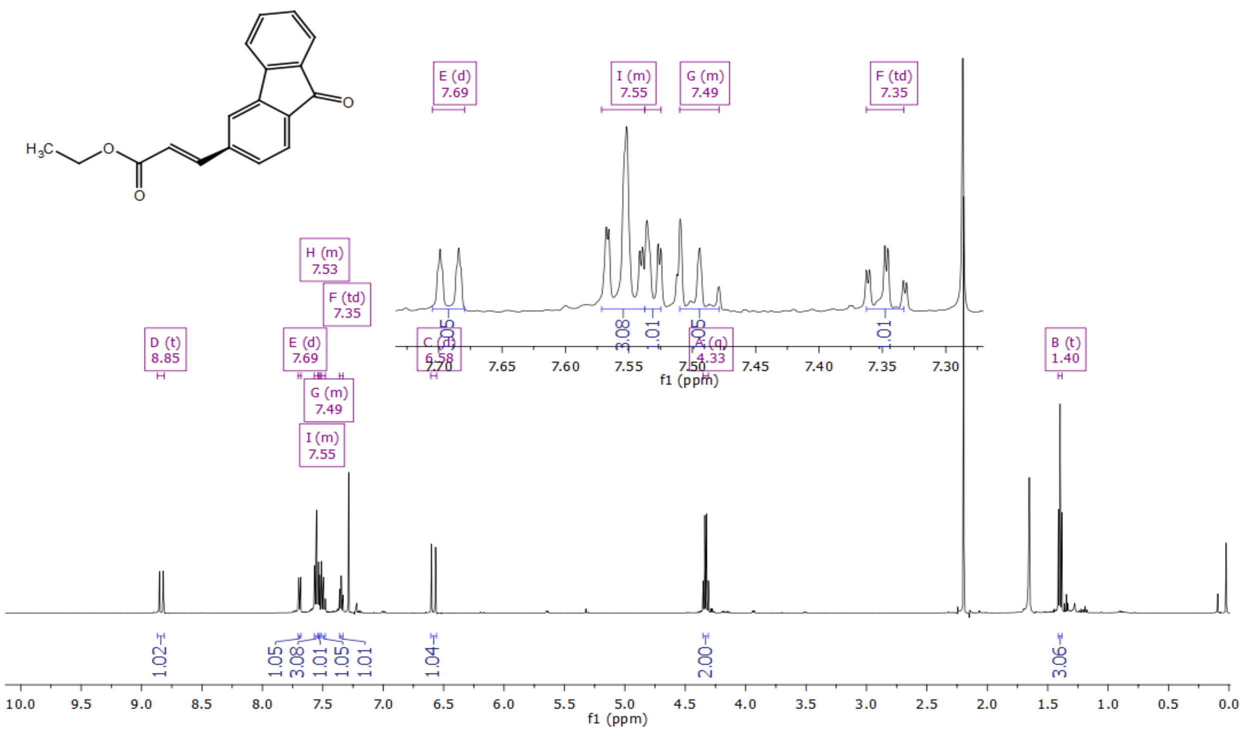

^{13}C NMR

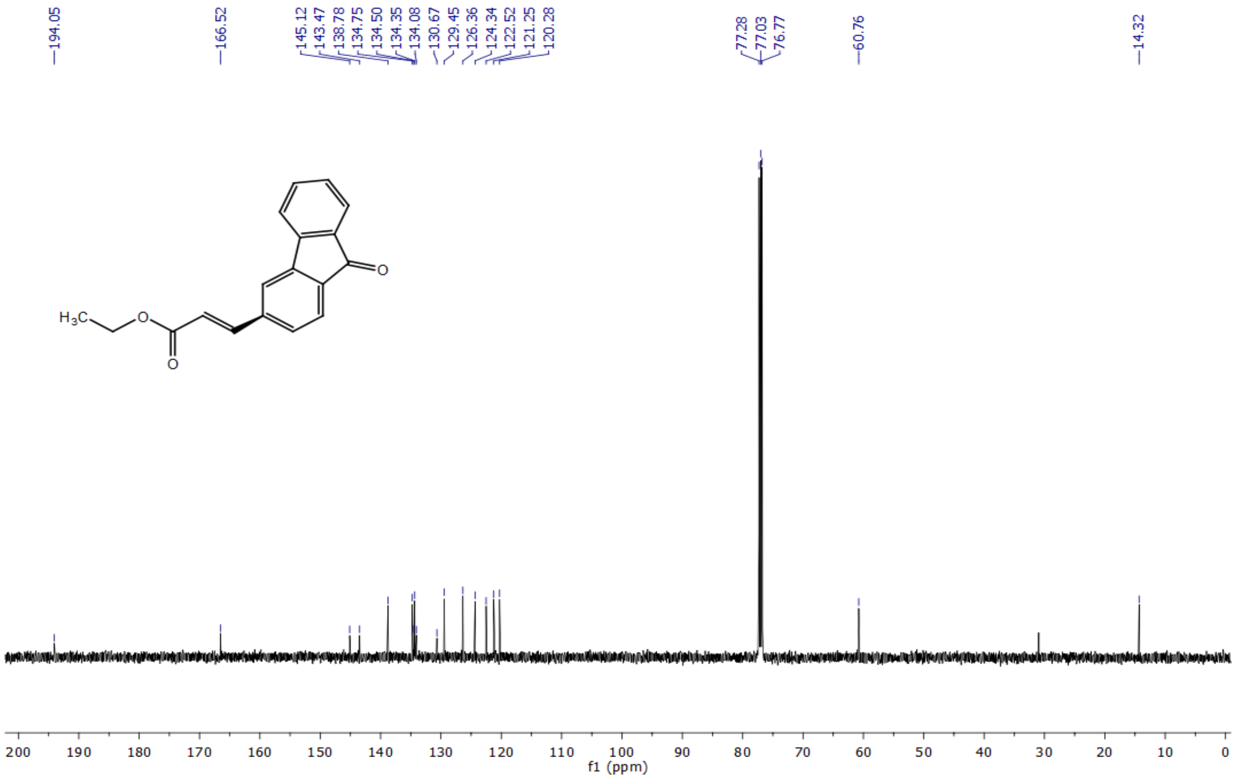

***Butyl (E)-3-(9-oxo-9H-fluoren-3-yl)acrylate* (77)**

^{1}H NMR

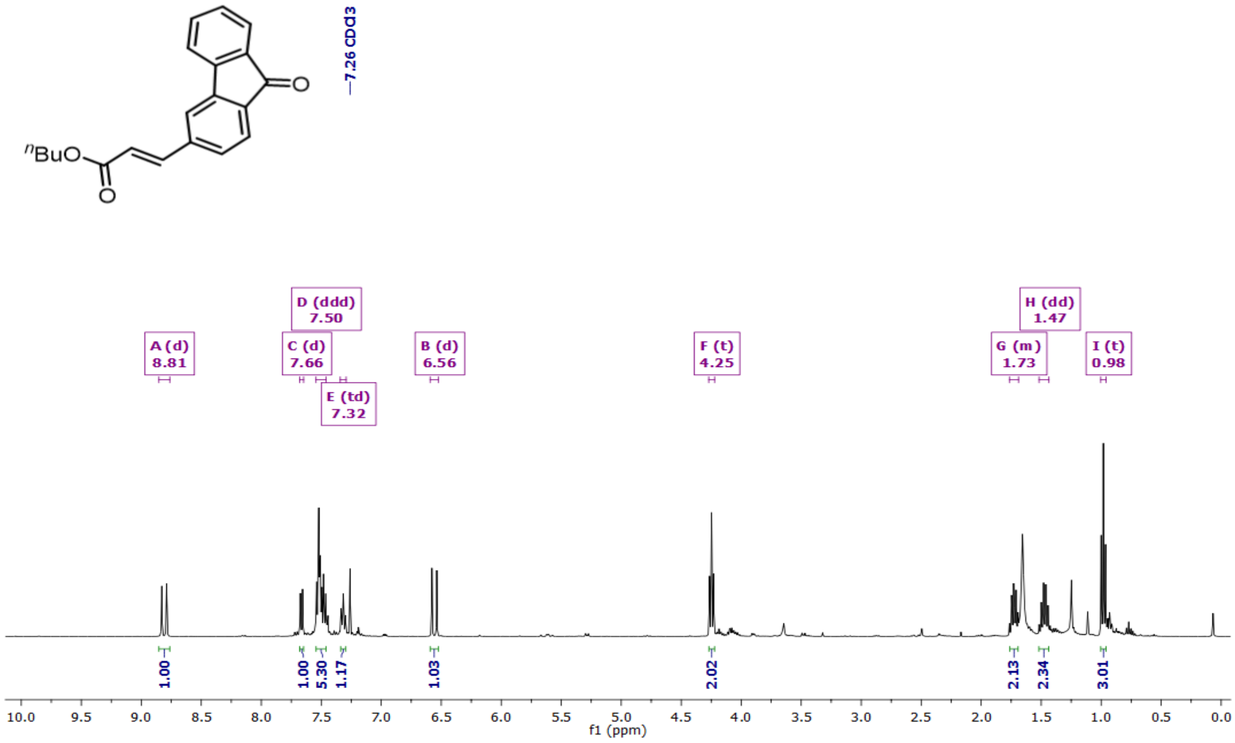

^{13}C NMR

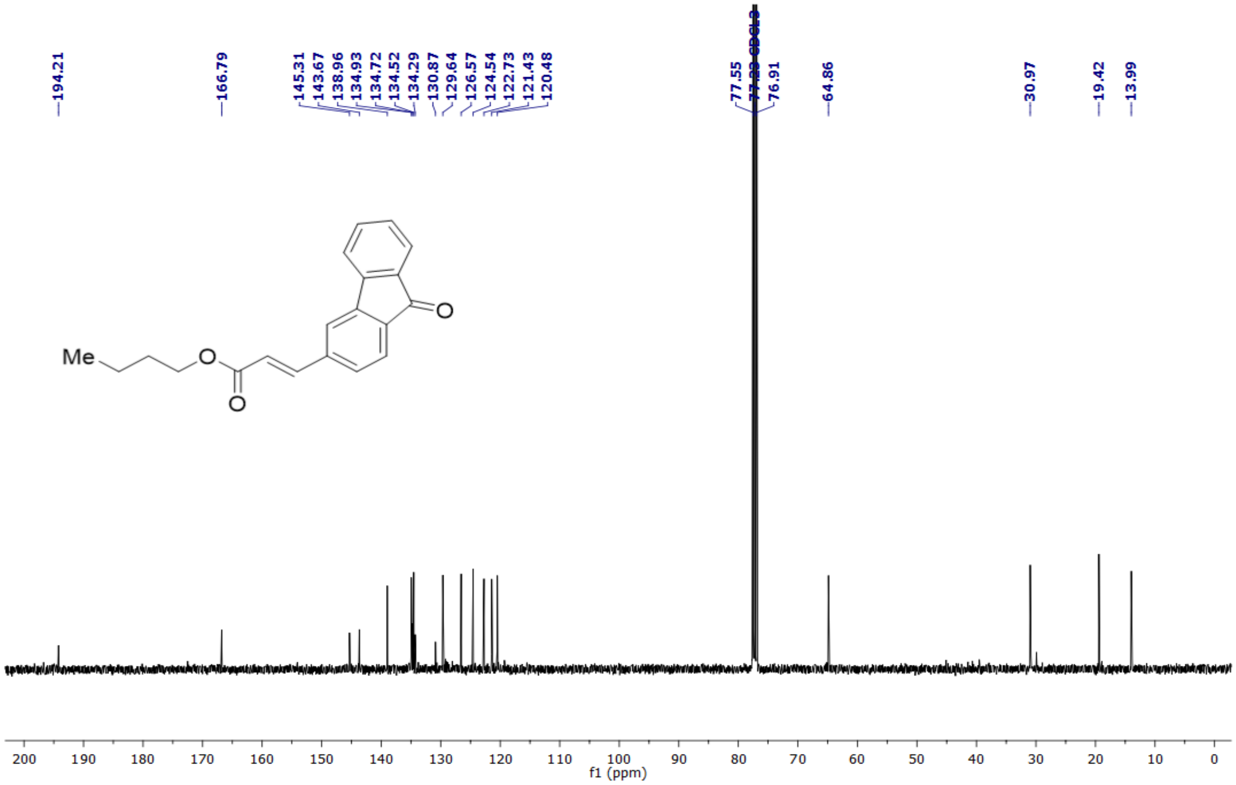

***Ethyl (E)-3-(6-oxo-6H-benzo[c]chromen-2-yl)acrylate* (78)**

^{1}H NMR

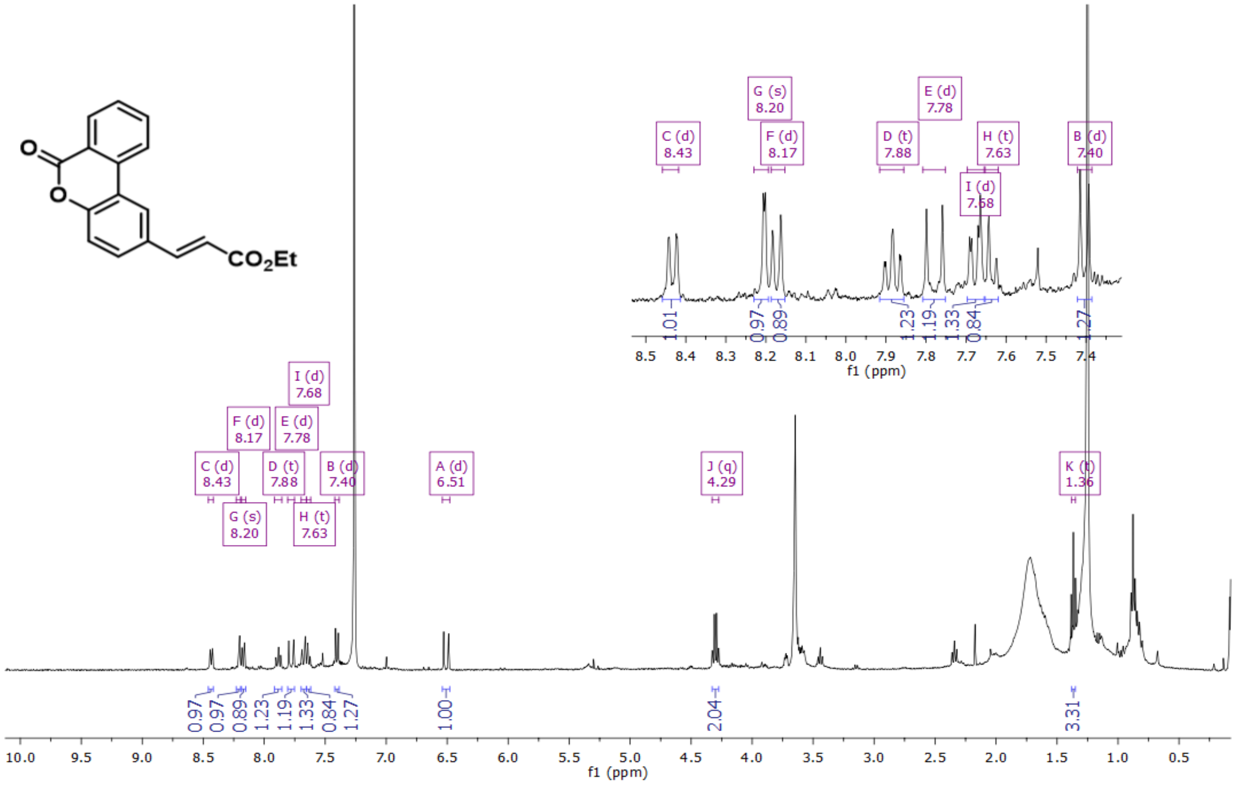

^{13}C NMR

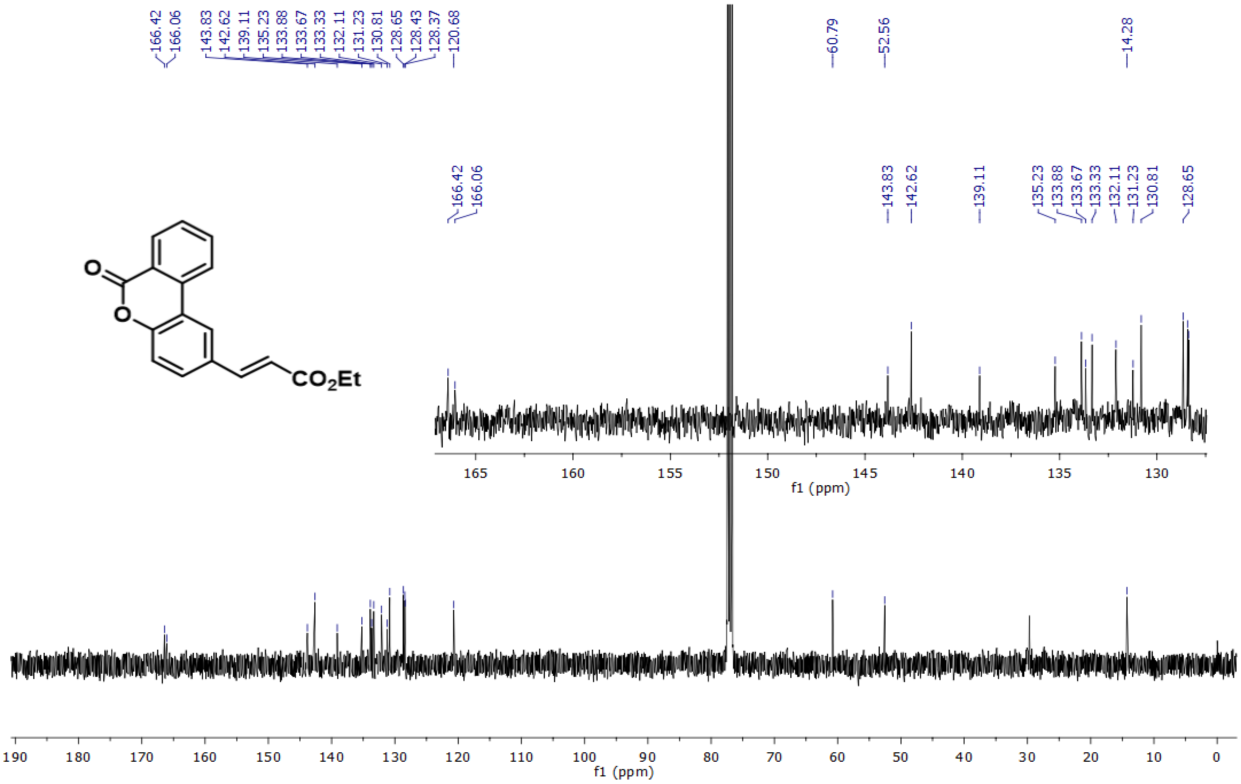

Ethyl (E)-3-(2'-(3-ethoxy-1-hydroxy-3-oxopropyl)-[1,1'-biphenyl]-3-yl)acrylate (79)

^{1}H NMR

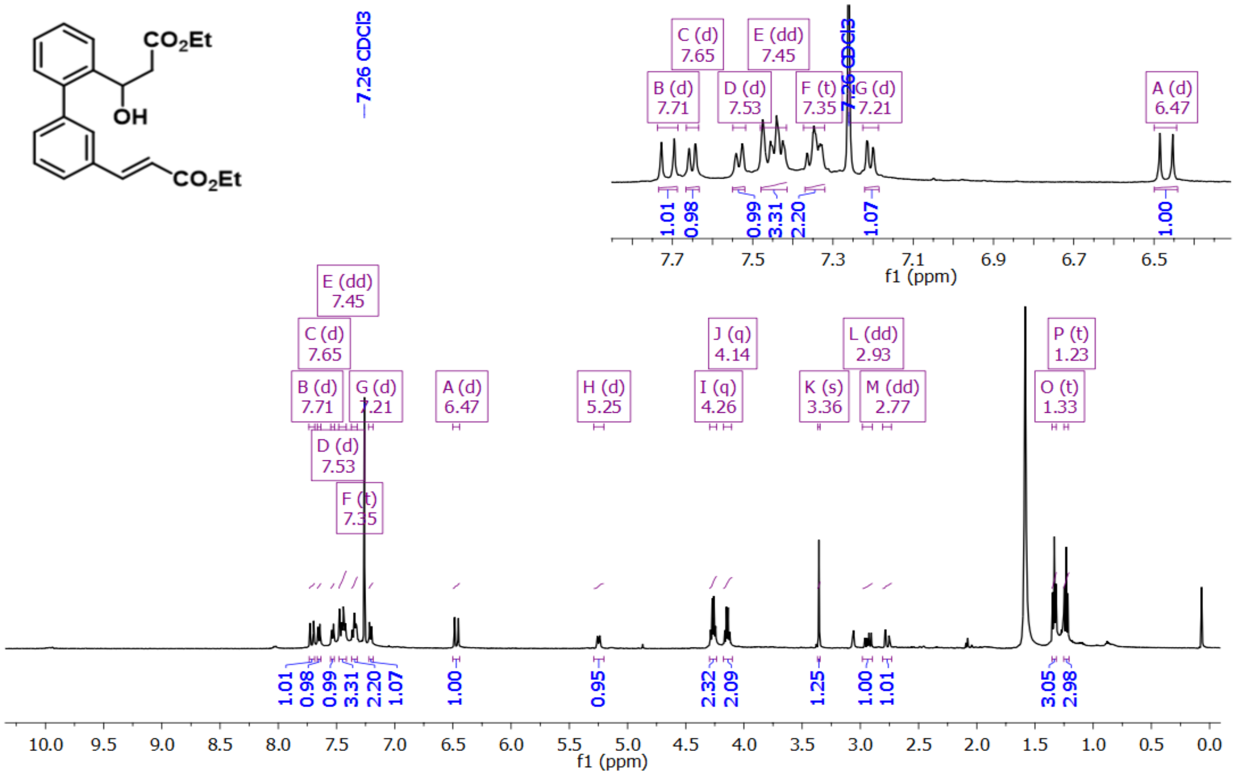

^{13}C NMR

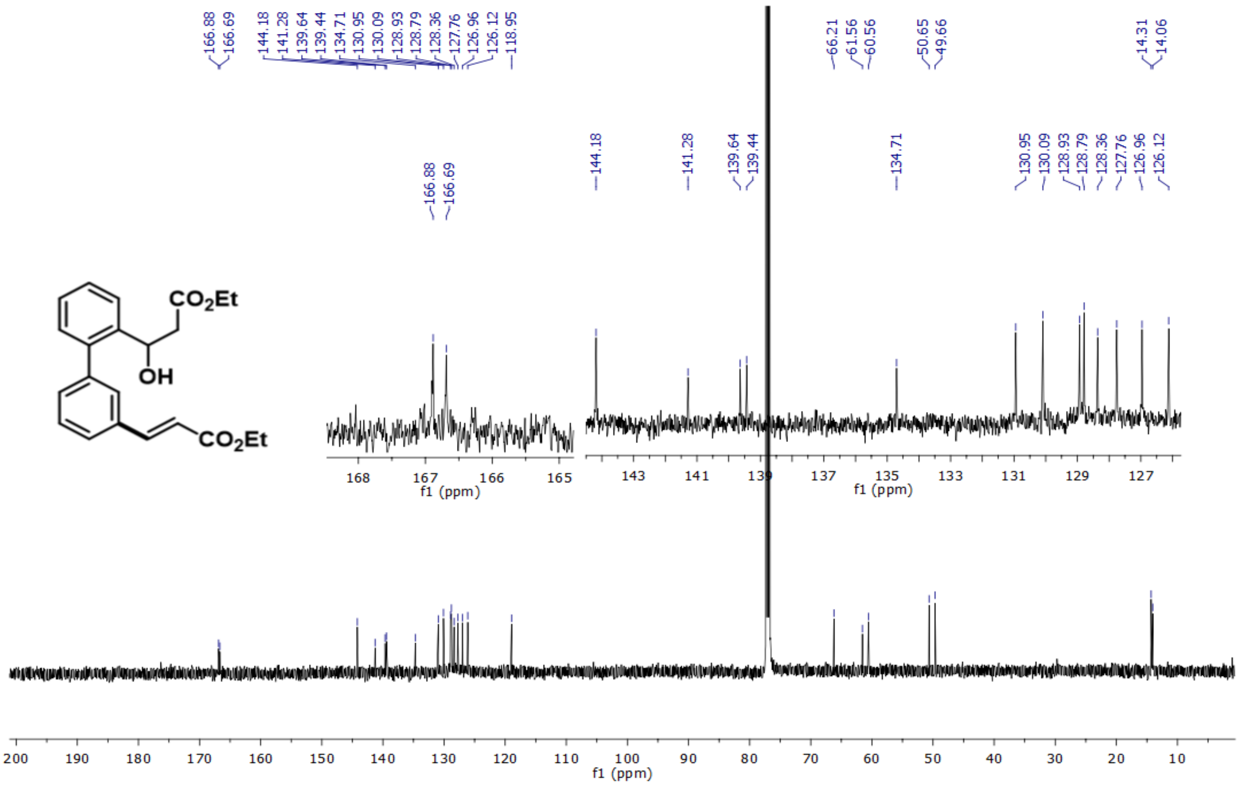

***Ethyl (E)-3-(2'-(1-hydroxy-3-oxobutyl)-[1,1'-biphenyl]-3-yl)acrylate* (80)**

^{1}H NMR

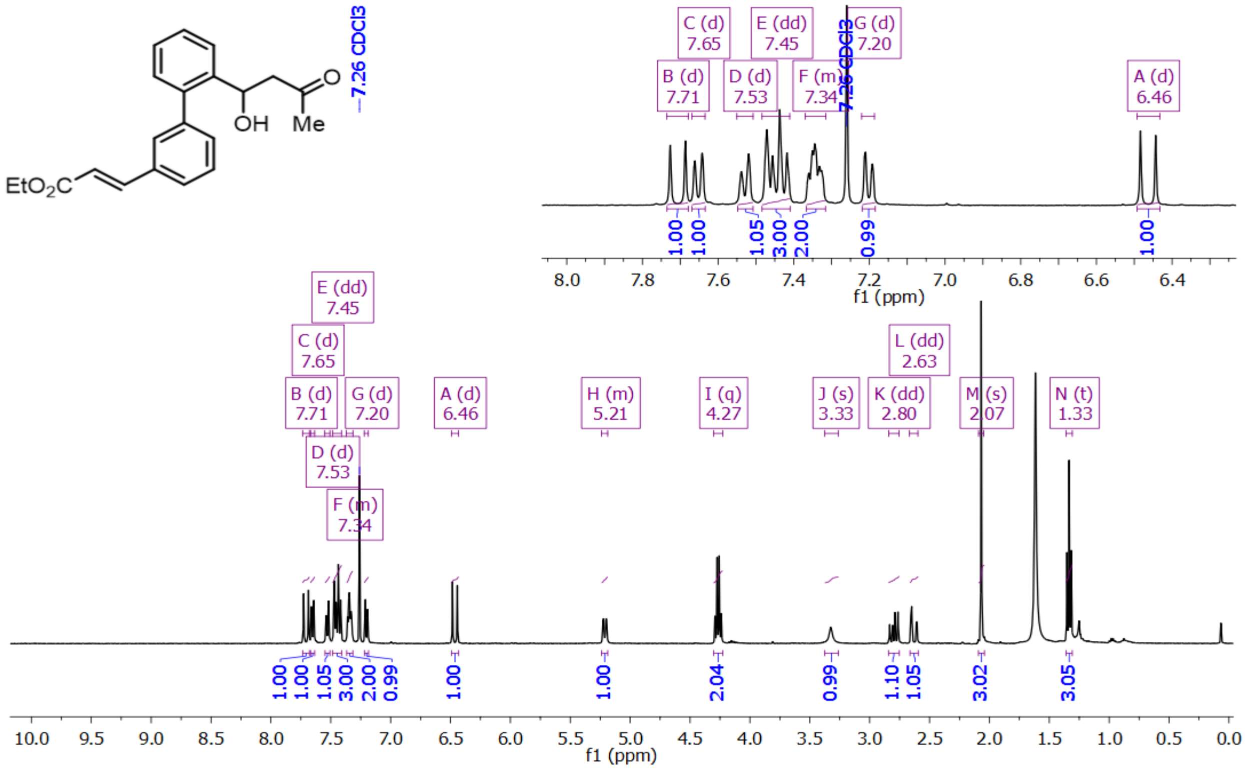

^{13}C NMR

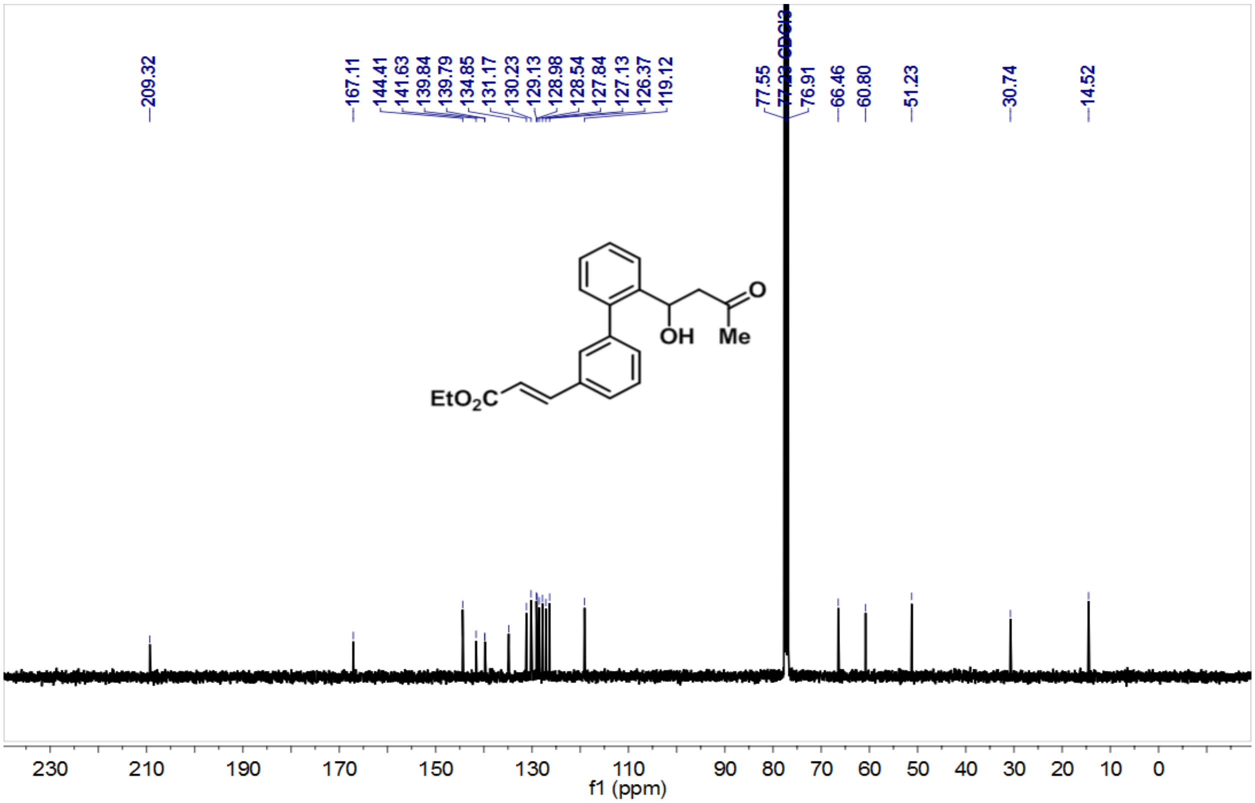

Ethyl (E)-3-(2'-vinyl-[1,1'-biphenyl]-3-yl)acrylate (**81**)

^{1}H NMR

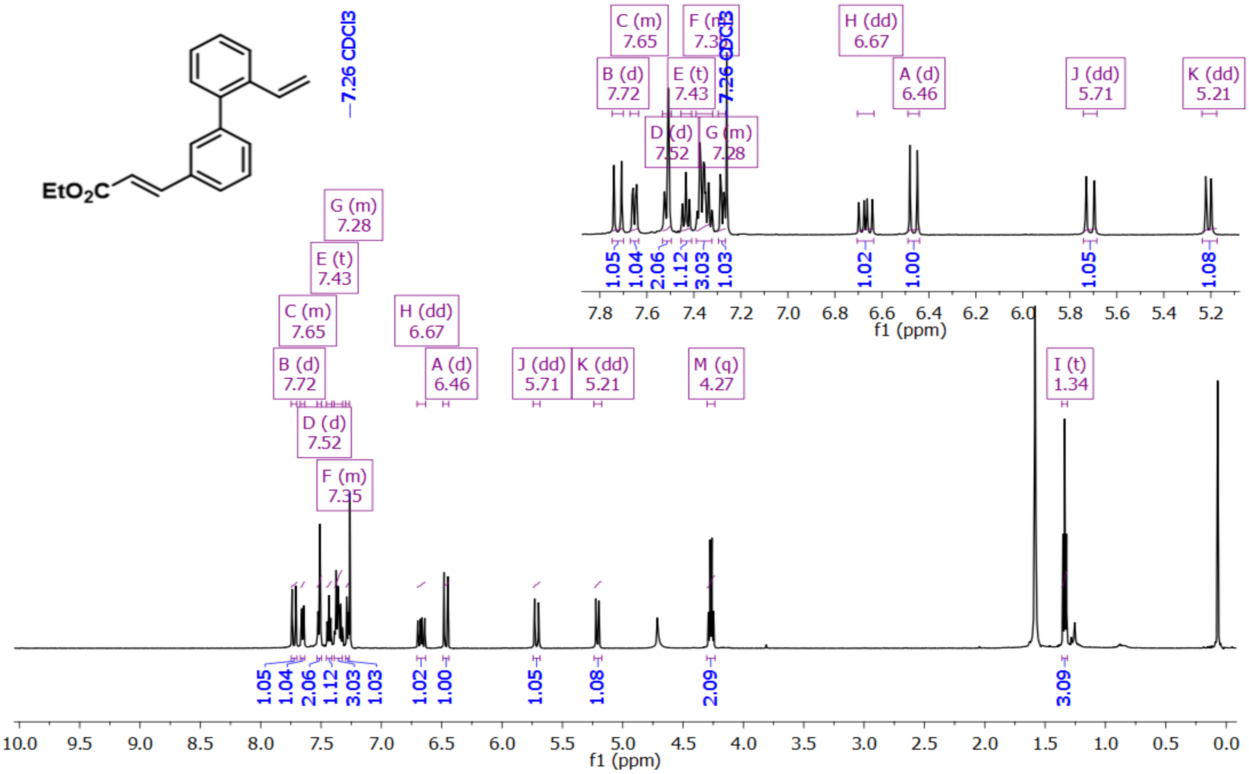

^{13}C NMR

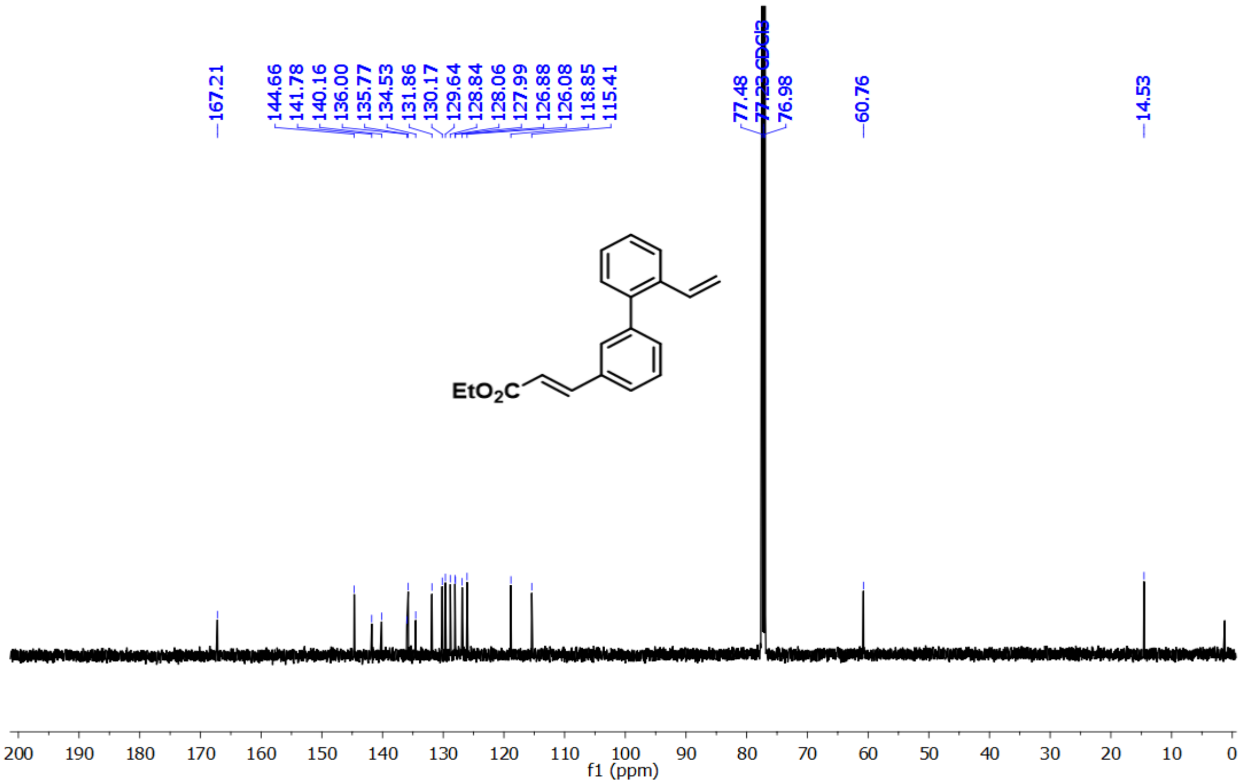

Ethyl (E)-3-([1,1'-biphenyl]-3-yl)acrylate **(82)**

^{1}H NMR

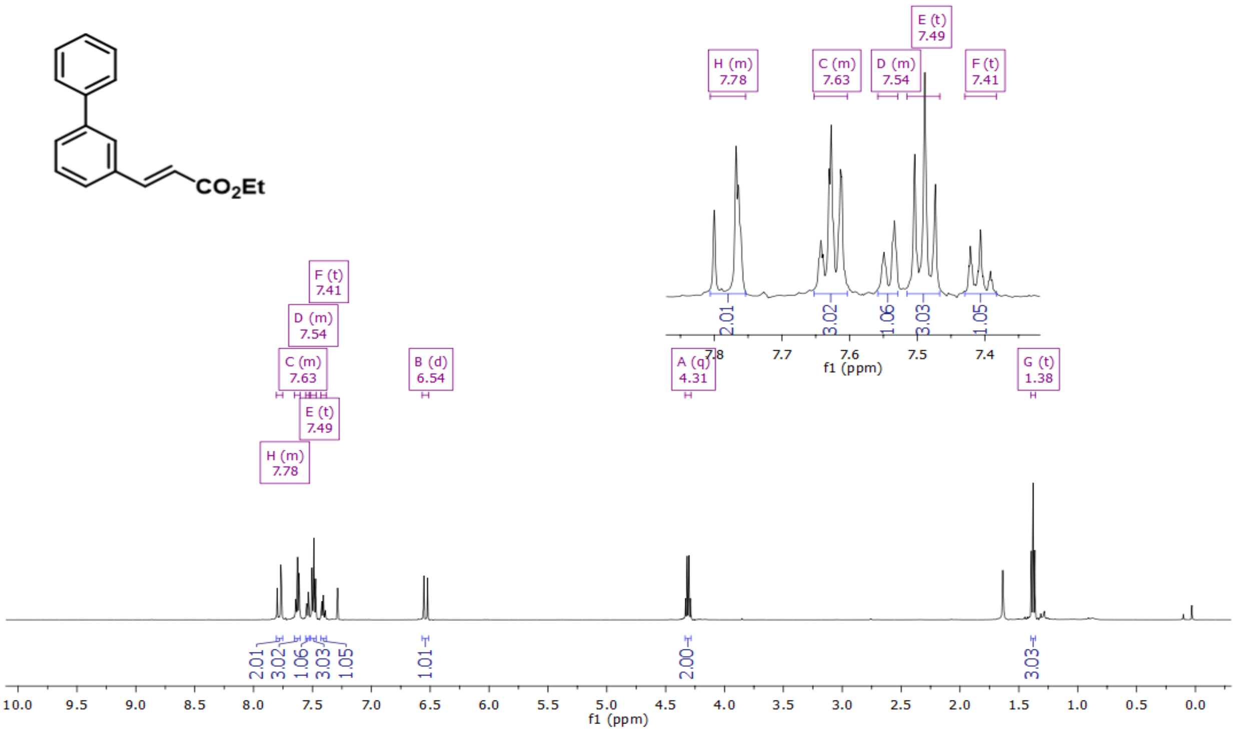

^{13}C NMR

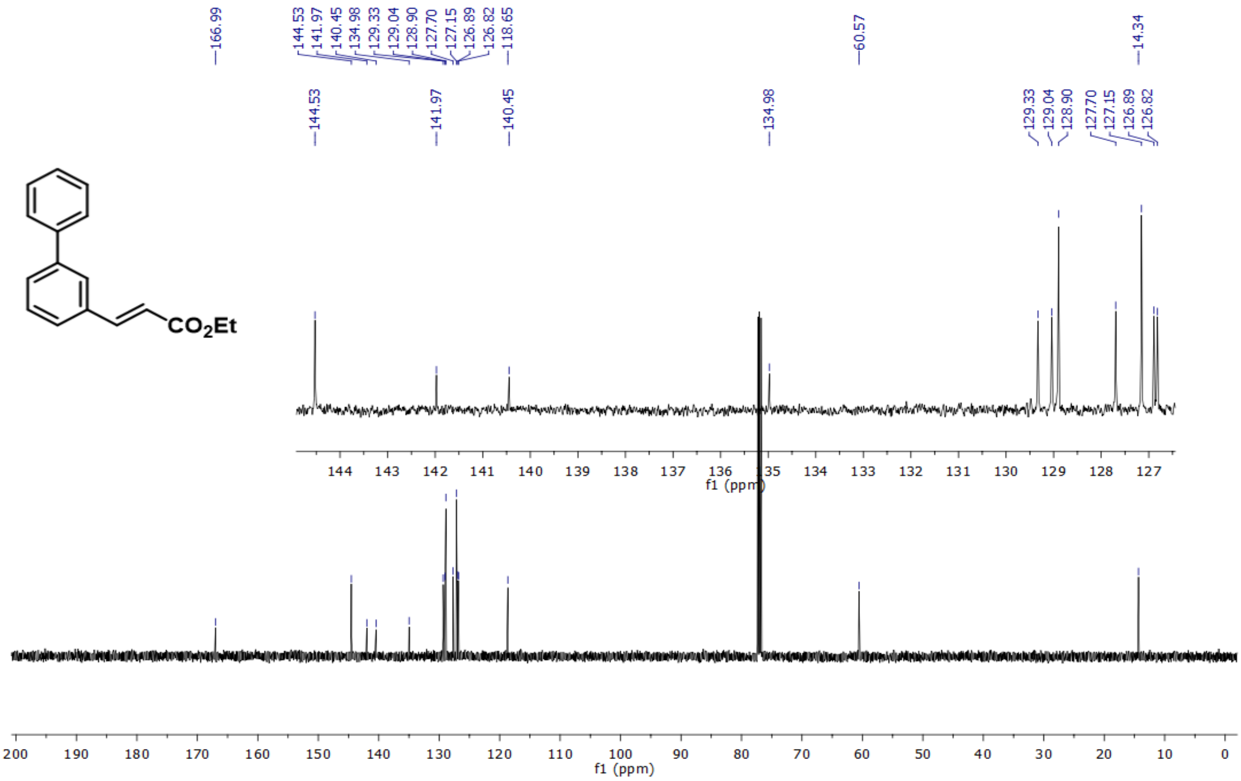

Ethyl (E)-3-([1,1'-biphenyl]-3-yl)acrylate **(83)**

^{1}H NMR

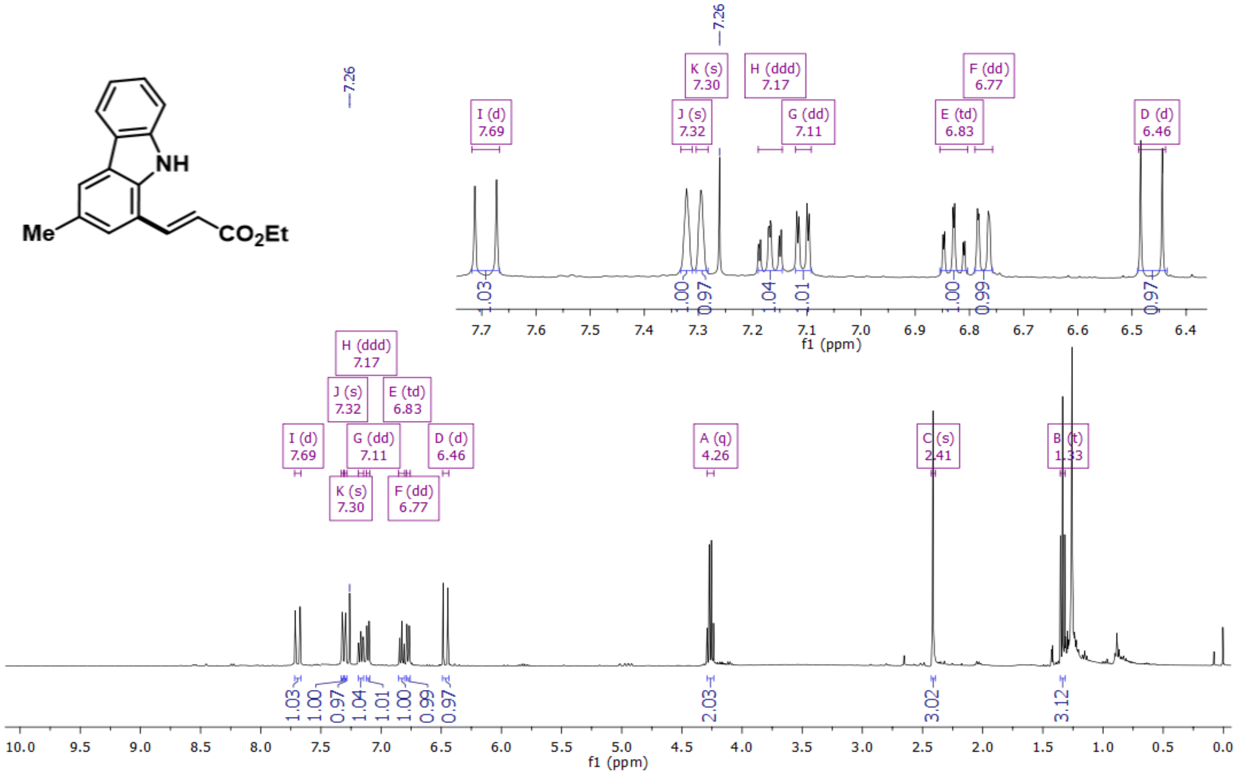

^{13}C NMR

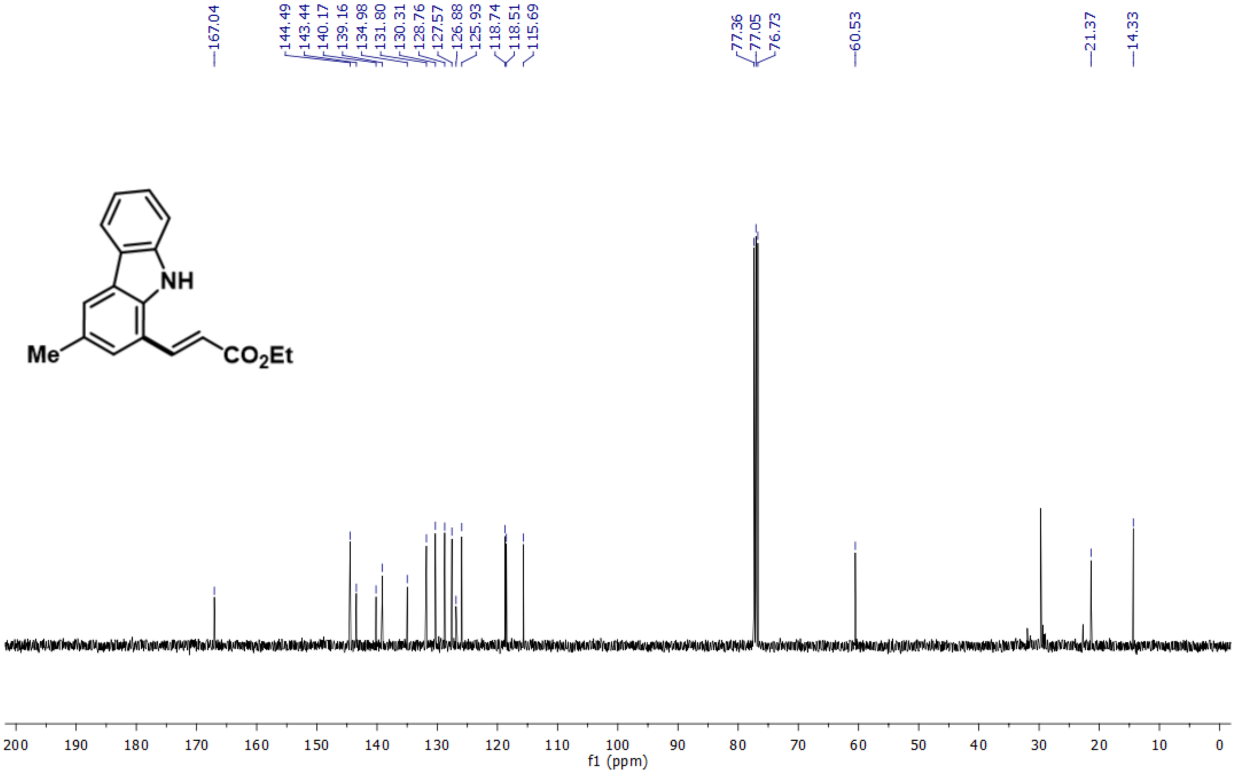

4. Supplementary References

1. Zhang, Z. et al. I_2-catalyzed one-pot synthesis of pyrrolo[1,2-a]quinoxaline and imidazo[1,5-a]quinoxaline derivatives via sp3 and sp2 C–H cross-dehydrogenative coupling. *Org. Chem. Front*. **2**, 942-946 (2015).
2. Padala, K. & Jeganmohan, M. Highly Regio- and Stereoselective Ruthenium(II)-Catalyzed Direct ortho-Alkenylation of Aromatic and Heteroaromatic Aldehydes with Activated Alkenes under Open Atmosphere. *Org. Lett.* **14**, 1134-1137 (2012).
3. Liu, X.-H., Park, H., Hu, J.-H., Hu, Y., Zhang, Q.-L., Wang, B.-L., Sun, B., Yeung, K.-S., Zhang, F.-L. & Yu, J.-Q. Diverse *ortho*-C(sp^2)–H Functionalization of Benzaldehydes Using Transient Directing Groups. *J. Am. Chem. Soc.* **139**, 888-896 (2017).
4. Shi, Z. & Glorius, F. Synthesis of fluorenones via quaternary ammonium salt-promoted intramolecular dehydrogenative arylation of aldehydes. *Chem. Sci.* **4**, 829-833 (2013).
5. Singha, R., Dhara, S., Ghosh, M. & Ray, J. K. Copper catalyzed room temperature lactonization of aromatic C–H bond: a novel and efficient approach for the synthesis of dibenzopyranones. *RSC Adv.* **5**, 8801-8805 (2015).
6. Xie, S., Li, S., Ma, W., Xu, X. & Jin, Z. Chelation-directed remote meta-C–H functionalization of aromatic aldehydes and ketones. *Chem. Commun.* **55**, 12408-12411 (2019).
7. Modak, A., Rana, S., Phukan, A. K. & Maiti, D. Palladium-Catalyzed Deformylation Reactions with Detailed Experimental and in Silico Mechanistic Studies. *Eur. J. Org. Chem.* **2017**, 4168-4174 (2017).
8. Suzuki, C., Hirano, K., Satoh, T. & Miura, M. Direct Synthesis of N-H Carbazoles via Iridium(III)-Catalyzed Intramolecular C–H Amination. *Org. Lett.* **17**, 1597-1600 (2015).
